# Proteomic-based identification of maternal proteins in mature mouse oocytes

**DOI:** 10.1186/1471-2164-10-348

**Published:** 2009-08-03

**Authors:** Ping Zhang, Xiaojian Ni, Ying Guo, Xuejiang Guo, Yufeng Wang, Zuomin Zhou, Ran Huo, Jiahao Sha

**Affiliations:** 1 Laboratory of Reproductive Medicine, Department of Histology and Embryology, Nanjing Medical University, Nanjing, PR China

## Abstract

**Background:**

The mature mouse oocyte contains the full complement of maternal proteins required for fertilization, reprogramming, zygotic gene activation (ZGA), and the early stages of embryogenesis. However, due to limitations of traditional proteomics strategies, only a few abundantly expressed proteins have yet been identified. Our laboratory applied a more effective strategy: one-dimensional sodium dodecyl sulfate polyacrylamide gel electrophoresis (1D SDS-PAGE) and reverse-phase liquid chromatography tandem mass spectrometry (RP-LC-MS/MS) were employed to analyze the mature oocyte proteome in depth.

**Results:**

Using this high-performance proteomic approach, we successfully identified 625 different proteins from 2700 mature mouse oocytes lacking zona pellucidae. This is the largest catalog of mature mouse oocyte proteins compiled to date. According to their pattern of expression, we screened 76 maternal proteins with high levels of mRNA expression both in oocytes and fertilized eggs. Many well-known maternal effect proteins were included in this subset, including MATER and NPM2. In addition, our mouse oocyte proteome was compared with a recently published mouse embryonic stem cell (ESC) proteome and 371 overlapping proteins were identified.

**Conclusion:**

This proteomics analysis will be a valuable resource to aid in the characterization of important maternal proteins involved in oogenesis, fertilization, early embryonic development and in revealing their mechanisms of action.

## Background

Mammalian reproduction is a complicated physiological process involving many important events, such as generation of mature gametes, fertilization, zygotic gene activation (ZGA), and embryonic development. Thus far, the key molecules and mechanisms involved in these events remain poorly characterized. Mammalian oocytes, a highly specialized cell type, play unique roles in reproduction because only in these cells are maternal proteins and transcripts crucial for the above-mentioned processes.

During oogenesis, oocytes synthesize and accumulate a number of maternal proteins. Some of them function in the formation of follicles and/or the growth of the oocytes, including, Figα, GDF9, and BMP15 [1-3]. However, many maternal proteins stored in oocytes play significant roles in later stages, namely fertilization and early embryogenesis. The corresponding genes are called maternal effect genes [4], and we call the proteins they code for maternal effect proteins. Maternal effect genes/proteins have been shown to be important in early embryonic development of *Drosophila melanogaster and Xenoupus laevis *[5,6]. Several maternal-effect genes/proteins have recently been identified in mammals, and their importance in embryonic development has also been demonstrated. MATER (Maternal antigen that embryos require; official name Nlrp5) is one of the first characterized maternal effect proteins in mice, the absence of which precludes embryonic progression beyond the 2-cell stage [7]. Npm2 is another well characterized maternal effect protein, which is required for nuclear and nucleolar organization during embryonic development [8]. Much research has been done to identify maternal effect genes or proteins essential for preimplantation or postimplantation mouse embryo development. Dppa3, Padi6, Tle6 and Floped were successfully identified in individual studies [9-11], but there remain many unknown players. Therefore, the identification and molecular characterization of novel maternal proteins will be of great significance and novel proteomic technologies can potentially deduce most of the maternal proteins in mature oocytes.

There are several recent reports utilizing proteomics approaches to the study of ooctyes, including the exploration of the bovine, pig and mouse oocyte proteomes [12-16]. For example, Calvert et al. identified 8 highly abundant heat shock proteins (HSPs) and related chaperones in the mature mouse egg by two-dimensional electrophoresis (2DE) [15]. Vitale et al. used 2DE and mass spectrometry (MS) to identify 12 proteins that appeared to be differentially expressed between germinal vesicle (GV) and metaphase II (MII) murine oocytes [16]. In our previous work that demonstrated post-translational modifications of maternal proteins, we used a similar approach to perform large-scale protein identification in mature mouse oocytes, and we successfully identified a total of 380 different proteins corresponding to 869 proteins spots [17]. The 2DE platform is valuable to analyze heterogeneity of proteins in the forms of alternative splicing, post-translation modifications, etc [18,19]. Although 2DE continues to be a very popular tool for studying the proteome, it has some limitations in identifying proteins that have either high or low molecular masses, those with extreme isoelectric points (pIs), those are highly hydrophobic, and those of low abundance [20]. 1D SDS-PAGE liquid chromatography tandem mass spectrometry (LC-MS/MS)-a combination of 1DE protein separation and LC-MS/MS analysis-has been used widely and is generally accepted as a more effective method of studying the proteome [21,22]. It is technically simple and combines improved protein separation capability that also captures those proteins typically not accessible via 2D PAGE (notably large proteins and those with transmembrane domains) with the well-established sensitivity of gel-based protein identification using MS for less complex samples [23]. For the purpose of identifying novel maternal proteins, we employed this high-performance proteomic approach to analyze proteins extracted from 2700 mature mouse oocytes lacking zona pellucidae and we successfully identified 625 different proteins. The maternal protein compilation provided here is intended to serve as an important tool for expanding our knowledge of the regulation of multiple processes in mammalian reproduction.

## Results

### Identification of Mature Oocyte Proteins

**Figure 1:**
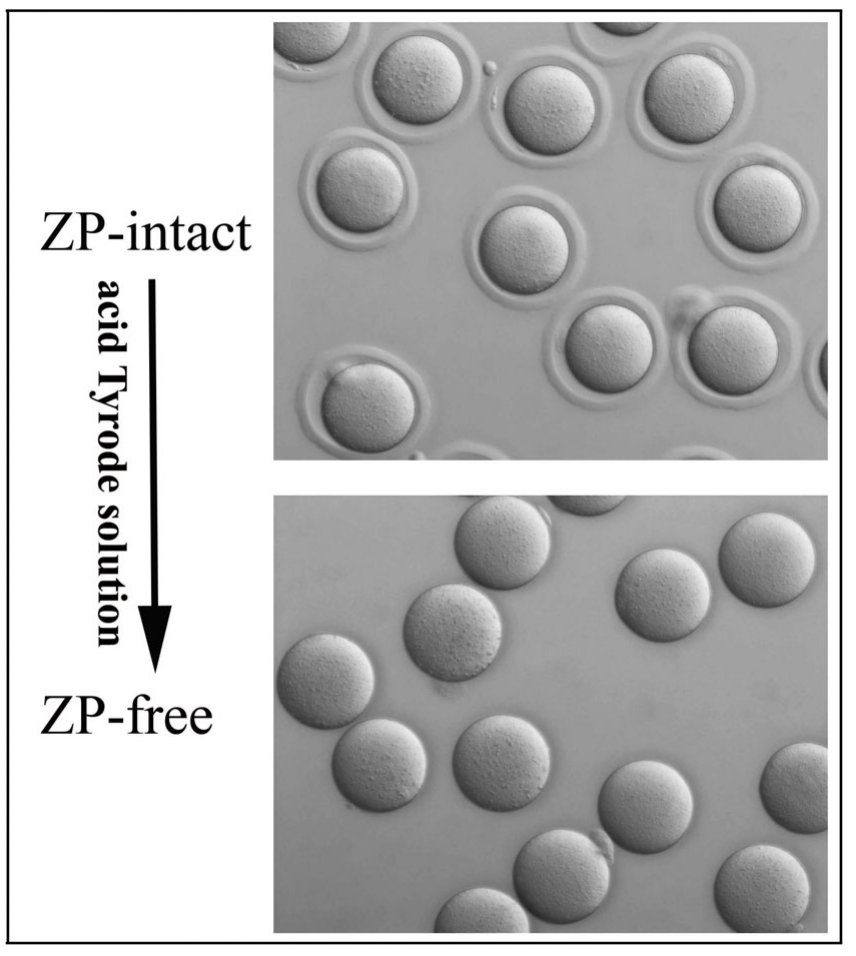
**A representative image of a ZP-free MII oocyte selected for proteomic analysis**. During the process of removing zona pellucidae, the status of oocyte was checked rigorously, and only healthy denuded oocytes were chosen for further study.

The mouse oocyte zona pellucida (ZP) is a thick extracellular coat containing, on average, ~3.5 ng of glycoprotein which contributes approximately 15% of the total egg protein [24]. Given that high-abundance proteins may interfere with the identification of other proteins, the ZP was removed by treating with acid Tyrode solution [25]. In total, 2700 ZP-free MII oocytes were collected and the integrity of the oocytes was checked rigorously according to the criteria outlined in the Materials and Methods to ensure only morphologically normal oocytes were chosen for further research (Figure 1). Oocytes passing these selection criteria were lysed and their proteins were separated by 1D SDS-PAGE. The gel was then cut into 29 slices, proteins were in-gel digested with trypsin, and the resulting peptides extracted from each gel slice were analyzed by automated reverse phase LC (RP-LC) coupled with MS/MS. From 29 LC-MS/MS runs, a total of 42711 MS/MS spectra were acquired and searched against the IPI Mouse database v3.30 by SEQUEST. To experimentally verify the false discovery rate (FDR) in our dataset, all output files were searched against the reversed IPI Mouse database, yielding an FDR of ~1.8%. We applied additional filter criteria to exclude proteins identified with low probabilities. The confidence score of the protein was calculated by applying the PeptideProphet algorithm using Scaffold software (v01_07_00; Proteome Software) [26], and only proteins with confidence scores of more than 90% were included in our dataset. Identical peptide sequences are occasionally shared by biologically distinct proteins, thus it is occasionally difficult to determine the identities of proteins based on sequenced peptides unless unique peptides are identified. In our analysis, proteins with shared peptides were organized into a single group (protein group) in Scaffold. If a protein group comprised only isoforms or overlapping database entries indistinguishable by MS/MS analysis, then the proteins in this group were counted as a single protein. If these proteins were the products of distinct genes, then all the proteins in the group were discarded from our dataset. Consequently, the final number of identified proteins was lower than the actual number of proteins in the sample. In summary, our dataset included 625 proteins corresponding to 611 known genes and 11 proteins from uncharacterized genes [see Additional file 1]. MS/MS spectra and fragment assignments of single peptide-based identifications are provided [see Additional files 2, 3 and 4].

**Figure 2:**
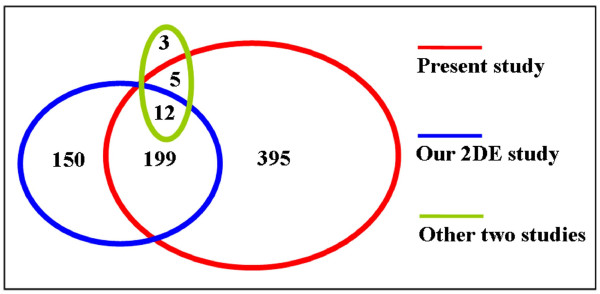
**Diagram of proteins identified in different studies**. Maternal proteins identified in the present study were compared with three recently published proteomics datasets, including the 2DE study conducted in our lab [15] and two other published reports [16,17]. Numbers represent the number of shared proteins in the respective overlapping area.

We were interested to compare the mature oocyte proteins identified in the present study with those discovered by other analyses, including the 2DE study conducted in our lab as well as two other published reports [15-17]. Protein IDs in the datasets from each study were converted to gene symbols. As shown in Figure 2, a sum of 369 unique gene products were reported previously. Of these, 216 (67.7%) were also found in our present dataset, whereas 395 gene products were found only in our present study. Therefore a total of 764 different maternal proteins have been identified from mature mouse oocytes thus far.

### GO and Pathway Analysis of the Identified Proteins

**Figure 3:**
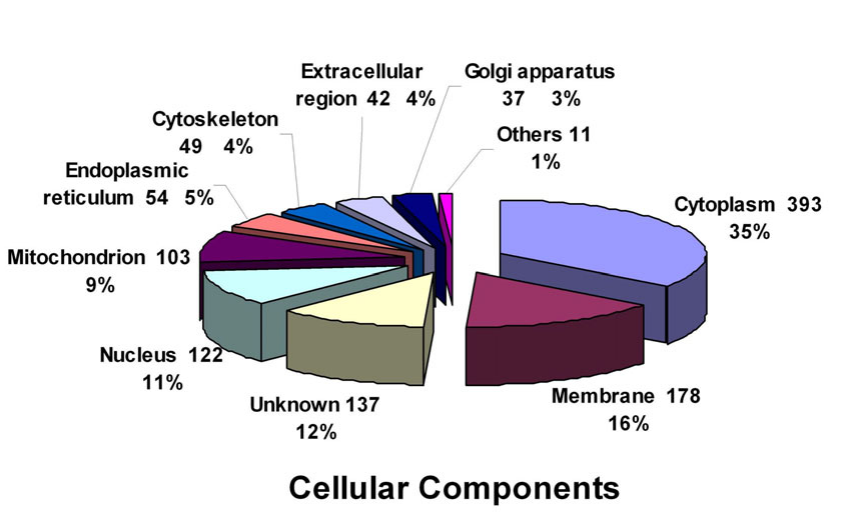
**Distribution of oocyte proteins among subcellular compartments**. The classification of the 625 identified maternal proteins was performed according to the gene ontology term "Cellular component".

In Figure 3 we have the categorized proteins identified in this study in terms of cellular components based on Gene Ontology (GO) analysis. A majority of proteins (393) were assigned to the cytoplasmic compartment, accounting for 35% of the identified proteins. Among the proteins classified by GO annotation, 16% (178) were membrane proteins, followed by proteins of unknown localization (12%), nuclear (11%), and mitochondrial (5%) proteins.

**Figure 4:**
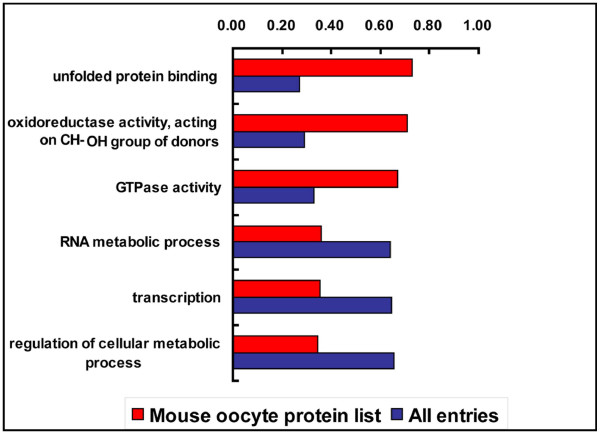
**Over- and underrepresented annotations of the set of identified maternal proteins**. The set of identified maternal proteins was compared with the combined database of multiple mouse tissues, and significantly GO molecular function and biological process terms (p < 0,05) are illustrated.

To examine what biologically importantly entries are enriched in the mouse oocyte, we compared our dataset with a combined database of multiple mouse tissues as a reference. Generation of this combined proteomic database was based on the avaliable proteomic data from different tissues or cells created with the same or similar LC-MS/MS exprimental approach. This strategy can overcome the biases caused by the experimental approach [27]. As a result, ~28,600 genes with duplicates were included in the combined dataset. Babelomics http://babelomics.bioinfo.cipf.es/EntryPoint?loadForm=fatigo, an advanced set of tools for the functional profiling of high-throughput transcriptomic, genomic and proteomic data [28], was used to carry out the calculation. When compared to the pooled proteomic database, 3 GO terms (unfolded protein binding, oxidoreductase activity, acting on CH-OH group of donors, GTPase activity) for molecular function appeared to be significantly overrepresented, and none significantly underrepresented. In the biological process category, 3 GO terms (RNA metabolic process, transcription, regulation of cellular metabolic process) appeared to be significantly underrepresented, and none significantly overrepresented (Figure 4). We found that transcription in the mouse oocyte was also underrepresented. Recent studies have demonstrated that oocytes undergo large-scale chromatin modifications in the process of maturation, including acetylation and methylation of the histone proteins, and finally global transcriptional repression appears [29]. So, the global transcriptional activity of the MII oocytes is very low, and this is consistent with our results.

**Figure 5:**
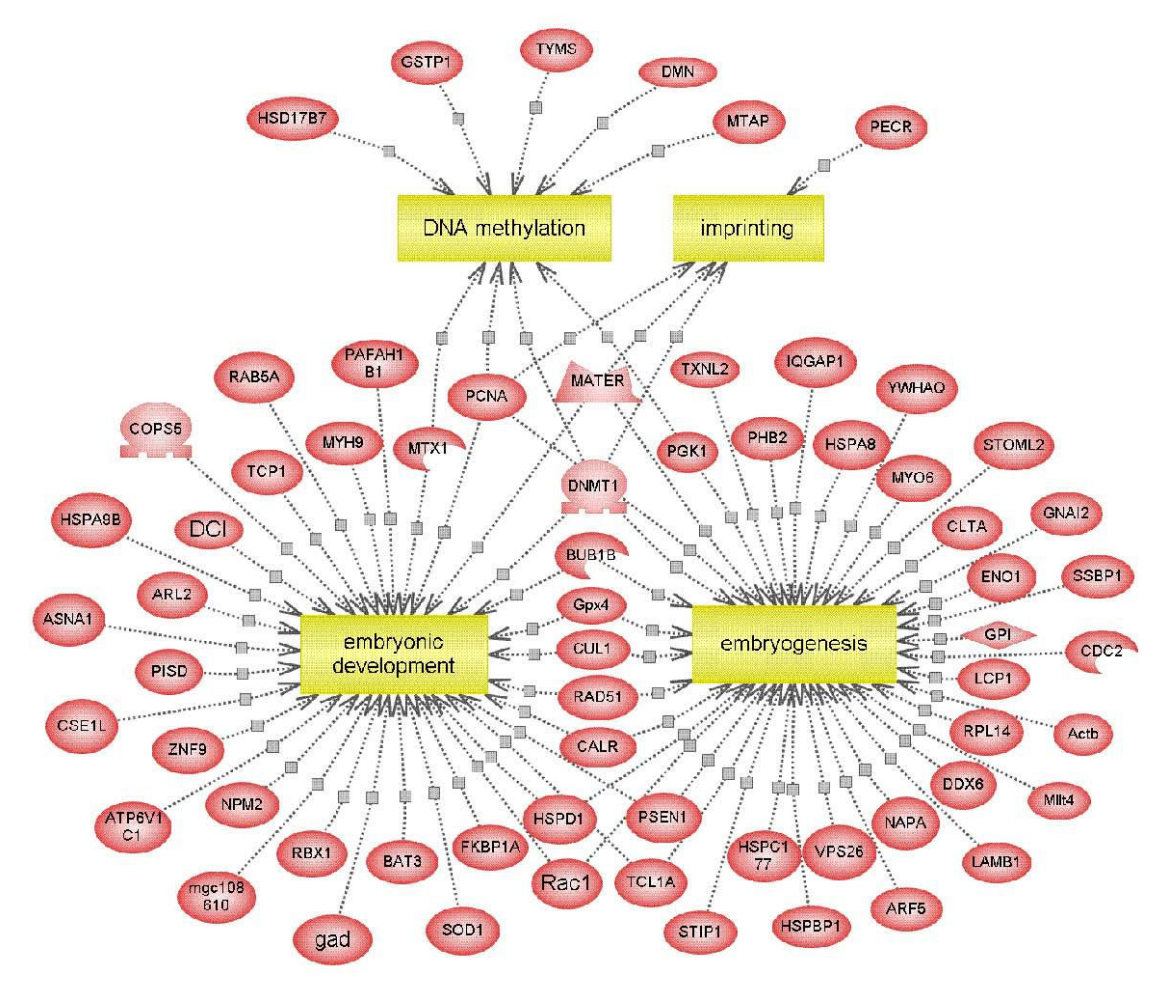
**Biological pathways deduced for the identified maternal proteins using Pathway Studio software**. Proteins involved in early embryogenesis events are shown. Proteins are indicated as red ovals and regulated processes are represented by yellow squares. Regulation events are displayed with arrows and documented by literature citations.

A key function of maternal proteins accumulated in mature oocyte is the regulation of early embryogenesis. A detailed analysis of embryonic development influenced by the protein profile of the mouse oocyte was performed using Pathway Studio, an automated text-mining tool which enables the software to generate pathways from entries in the PubMed database as well as other public sources. Pathway analysis revealed that 65 (11%) unique gene products were involved in early embryogenesis events (Figure 5).

### Analysis of High Abundance Proteins

The number of unique peptides identified is generally accepted as a semiquantitative measure of protein abundance [30,31]. We sorted the 625 proteins in our dataset according to the number of unique peptides identified. The highest ranked 23 proteins represented by more than 10 unique peptides are listed [see Additional file 5]. Among the high abundance proteins, some members have already been well characterized in oocytes. For example, DNA methyltransferase 1 (Dnmt1), is the predominant form of DNA (cytosine-5-)-methyltransferase in mammals and is essential for embryo development [32]. This protein was represented by 25 unique peptides and was the second-most abundant protein in our dataset.

### Screening for Proteins with Special mRNA Expression Patterns

Unique or atypical mRNA expression patterns of maternal proteins suggest their key roles in oogenesis or early embryo development and this notion has been validated by other functional research on maternal effect proteins [7-11]. We adopted an *in silico *approach to investigate the expression patterns of proteins in our dataset by examining their expression in the mouse transcriptome database SymAtlas http://symatlas.gnf.org/SymAtlas/, which describes gene expression patterns in 45 mouse tissues [33]. Surprisingly, many of the proteins in our dataset exhibited patterns worthy of note. We were particularly interested in genes highly expressed in oocytes and fertilized eggs (where expression was 10-fold higher than the corresponding median values). In total, 76 proteins, representing approximately 12% of the entire dataset, were included in this subset [see Additional file 5]. As expected, many well known maternal effect proteins in mice were found in this subset. Included in this subset were the earliest identified maternal protein MATER, as well as the recently identified TLE6 [11]. In addition to these well-known maternal effect proteins, many of the proteins we identified as highly expressed in oocytes and fertilized eggs have not been characterized.

To explore the functions of the 76 proteins with abundant mRNA expression levels, we analyzed their domain composition with Babelomics. Statistical analysis indicated that the most significantly overrepresented domain was the F-box domain (Pfam: 6 proteins, p = 3.9E-05) and the 6 maternal proteins containing F-box domains are listed [see Additional file 5]. Different F-box proteins, as components of the Skp1-Cullin1-F-box (SCF) complex, can recruit particular substrates for ubiquitination and play central roles in cell-cycle regulation [34].

### Intersection Between the Oocyte and ESC Proteome

Somatic cells can be reprogrammed by transferring their nuclear contents into oocytes [35] or by fusion with embryonic stem (ES) cells [36,37]. This fact indicates that unfertilized eggs and ES cells probably contain similar factors that can confer totipotency or pluripotency to somatic cells. In order to identify a common set proteins shared between oocytes and ES cells, mouse MII oocyte proteins were compared with recently published data for proteins expressed in mouse ES cells [31]. This analysis resulted in an overlap of 371 proteins [see Additional file 5]. As expected, developmental pluripotency associated 5 (DPPA5), which has been implicated in cell pluripotency, was included in this set. Of the 371 proteins, 108 (29%) were either exclusively found in mouse ES cells or highly enriched in ES cells compared to differentiated ES cells [see Additional file 5] [31]. It is likely that novel factors associated with reprogramming are included in this subset.

## Discussion

Activation of the embryonic genome in mice begins late in one-cell zygote and is fully underway by the two-cell cleavage stage [38]. The silencing of nuclear transcription occurring between meiotic maturation in oocytes and activation of the embryonic genome implies critical roles for preexisting stores of proteins and transcripts [39]. Through knockout and knockdown strategies, individual maternal proteins have been demonstrated as essential for cleavage stage development in mice. In the present study, we identified many new and unknown maternal proteins in mice by constructing an MII oocyte proteome. In-depth analysis of these maternal proteins will assist us in screening for a proportion of great interest.

Interestingly, many maternal transcripts deposited in mammalian oocytes are not polyadenylated and therefore not translated into proteins [40]. Independent confirmation of the protein expression of maternal genes is therefore necessary. This was also an important reason for us to construct the oocyte proteome. As a case in point, NALP14, NALP5 (MATER), and NALP4f were included in our subset of abundantly expressed maternal proteins. These three proteins belong to the multifunctional NACHT nucleoside triphosphatase (NTPase) family. NALP14 and NALP5 were previously reported as maternal effect proteins and play significant roles in mouse preimplantation embryo development [7,41,42]. NALP4f was represented by 14 unique peptides in our present study and a previous analysis demonstrated that *NALP4f *was an oocyte-specific gene [42]. Our research has independently confirmed the high protein expression level of NALP4f in mature oocytes. Assuming that NALP4f has similar roles to NALP14 and NALP5, it is highly likely that NALP4f is an important factor necessary for normal embryogenesis and is a good candidate to be a maternal effect protein. In addition, we identified NALP2, NALP4b, and NALP9b in our oocyte proteome. Although the precise functions of NACHT NTPase family members remain to be determined, we speculate that these members play significant roles in early embryo development based on their homology to NALP14 and NALP5.A distinguishing characteristic of maternal effect proteins identified to date is that the majority of them have an abundant mRNA expression in oocytes and many are expressed only in oocytes [7-11]. This fact led us to filter maternal products in our protein list by analyzing their corresponding mRNA expression patterns. As a result, 76 maternal proteins with high mRNA expression levels in oocytes and fertilized eggs were selected out. Of these proteins, we discovered that 9 previously described maternal effect proteins (MATER, STELLA, DNMT1, ZAR1, NPM2, PADI6, TLE6, TCL1, FILIA) were enriched in this subset. These maternal effect proteins have been reported to be absolutely necessary for oogenesis, fertilization or early embryo development. Indeed, apart from these well-known examples, the majority of proteins in this subset have not been previously studied or reported in oocytes. We suggest that these proteins are excellent candidates as maternal effect proteins.

**Figure 6:**
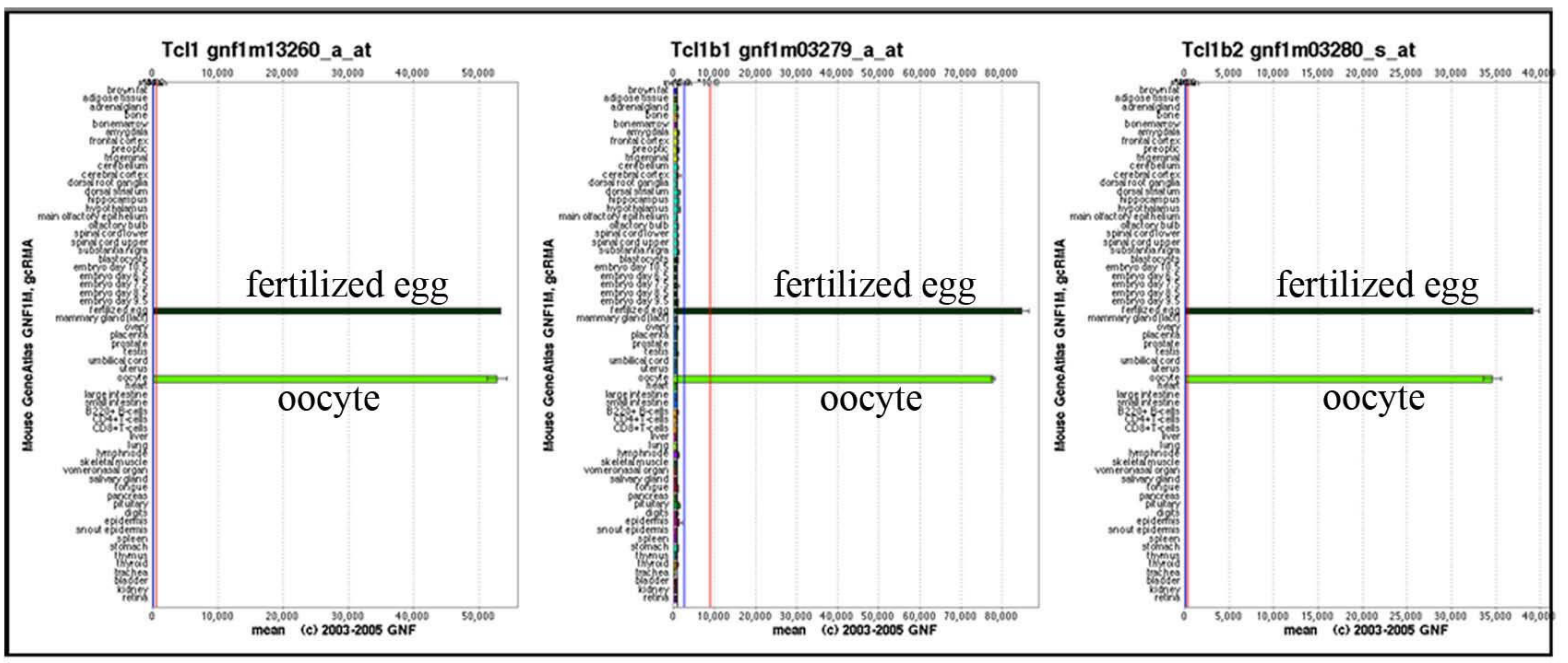
**mRNA expression patterns of three maternal proteins**. GNF SymAtlas database analysis shows that *TCL1, TCLB1 *and *TCLB2 *are almost solely expressed in the mouse oocyte and fertilized egg.

A group of proteins belonging to the T-cell leukemia/lymphoma 1 (TCL1) protein family was of particular interest because their corresponding genes had dramatically similar mRNA expression patterns. Figure 6 demonstrates that *TCL1, TCLB1, TCLB2 *are almost oocyte-specific genes. *TCL1 *was initially identified as a gene involved in recurrent chromosomal translocation in human prolymphocytic leukemia (T-PLL) and overexpression of *TCL1 *played a causative role in T cell leukemias of humans and mice [43]. However, in *TCL1*-deficient mice, a female fertility defect was observed. *TCL1*-deficient females display normal oogenesis and rates of oocyte maturation/ovulation and fertilization, but the lack of maternally derived TCL1 impairs the embryo's ability to undergo normal cleavage and develop to the morula stage, especially under *in vitro *culture conditions [44]. The *TCL1 *loss-of-function phenotype indicates that maternal protein TCL1 plays a significant role in early embryo development. Unfortunately the functions of TCLB1 and TCLB2 have not yet been investigated and we can speculate that the two proteins may play similar or complementary roles in embryogenesis.

Domain composition analysis is an effective way to predict the functions of proteins identified in a proteomics analysis. Among 76 proteins singled out because their corresponding genes are highly expressed in oocytes, 6 proteins (FBXL10, FBXW14, FBXW16, FBXW19, EG382106, E330009P21Rik) contained an F-box domain, which was first described as a sequence motif in cyclin-F that interacts with the SKP1 protein. Different F-box proteins, as substrate-specific adaptor subunits of the Skp1-Cullin1-F-box (SCF) complexes, recruit particular substrates for ubiquitination via specific protein-protein interaction domains. Coincidentally, three core protein subunits (SKP1, RBX1, CUL1) of the SCF complex were all definitively identified in our proteome. As one of the major classes of ubiquitin ligases, the SCF complex plays a central role in cell-cycle regulation [34]. In early stages of embryo development, degradation of maternal proteins is crucial for the oocyte-to-embryo transition [45]. Our results suggest that the maternal SCF complex probably exists in oocytes and may be important for the oocyte-to-embryo transition by recruiting specific substrates for degradation.

Pluripotent stem cells are of considerable current interest as they can proliferate indefinitely *in vitro *and give rise to many adult cell types, serving as a potentially unlimited source for tissue replacement in regenerative medicine. Recently, Takahashi et al. demonstrated that pluripotent stem cells can be induced from mouse fibroblasts by retroviral introduction of Oct3/4, Sox2, c-Myc and Klf4 [46], indicating that the combination of these four factors can induce reprogramming of somatic cells to a pluripotent state. However, the use of retrovirus-transduced oncogenes represents a serious barrier to the eventual use of reprogrammed cells for therapeutic application because of tumor formation by c-myc reactivation [47]. Therefore it is necessary to discover factors responsible for reprogramming that would be safer for therapeutic use. We compared the maternal proteins in our oocyte proteome with a recently published mouse ES cell proteome and identified an overlap of 371 proteins. In addition to some pluripotency markers, this group included many uncharacterized proteins, some of which may be good candidates for studying the mechanism of reprogramming. A good example is translationally-controlled tumor protein (Tpt1), which facilitates the first step of somatic cell reprogramming [48]. Recent studies on Tpt1 demonstrate that this protein activates transcription of *oct4 *and *nanog *in transplanted somatic nuclei [49]. We believe that further analysis of these candidate proteins at the functional level will uncover novel proteins that are essential for reprogramming and indirectly promote the application of therapeutic cloning.

## Conclusion

In this study, we used 1D SDS-PAGE and RP-LC-MS/MS to investigate the maternal proteins stored in mature mouse oocytes. This high-performance strategy allowed us to define a set of 625 different mouse MII oocyte proteins. This is the largest catalog of mature mouse oocyte proteins compiled to date. We believe that this study will help us to understand the diverse biological processes occurring in mouse oocytes and during early embryo development. However, compared with proteomic analyses of other cells and tissues, such as embryonic stem cells and liver, the proteins identified in mature mouse oocytes were limited. This was mainly because of the fact that mature oocytes obtained from each mouse were very limited. We believe that the catalog of maternal proteins present in this article is a starting point and we anticipate that more research on the oocyte proteome will deduce most of the maternal proteins.

## Methods

All experiments requiring the use of animals received prior approval from Nanjng Medical University and were performed according to USDA-approved protocols.

### Reagents

Urea (Cat. No. 17-1319-01), 3-[(3-Cholamidopropyl) dimethylammonio]-1-propanesulfonate (CHAPS) (Cat. No. 17-1314-01), iodoacetamide (Cat. No. RPN6302), and Dithiothreitol (DTT) (Cat. No.17-1318-02) were from GE Healthcare (Uppsala, Sweden); Thiourea (Cat. No. T7875), acetonitrile (ACN) (Cat. No. 34851), ammonium bicarbonate (NH4HCO3) (Cat. No. A6141), and trifluoracetic acid (TFA) (Cat. No. T0274) were from Sigma Chemical (St. Louis, MO); Protease inhibitor cocktail (Cat. No.78437) was purchased from Pierce Biotechnology (Rockford, IL).

### Oocyte collection and protein extraction

Mature oocytes were obtained from ICR female mice weighing 25-30 g. The mice were superovulated by intraperitoneal injection of 10 IU pregnant mare serum gonadotropin followed by 10 IU human chorionic gonadotropin after 48 h. After 14-16 h, oocyte-cumulus cell complexes were collected from the ampulla of the oviduct, and the cumulus cells were removed by brief exposure to 1 mg/ml hyaluronidase (Sigma Chemical, St. Louis, MO, USA). Zona pellucidae (ZP) were removed by treating oocytes for a few seconds with acid Tyrode solution (pH 2.5) followed by mechanical shearing. During this process, oocyte morphology was monitored rigorously and the oocytes with shape abnormalities or with cytoplasmic abnormalities (dark cytoplasm, granular cytoplasm, and refractile body) were discarded. Only the denuded oocytes with normal morphology were selected for further investigation. ZP-free oocytes were washed 3 times in 0.01 M PBS, and stored in lysis buffer at -80°C until needed. The lysis buffer consisted of 7 M urea, 2 M thiourea, 4% (w/v) 3-[(3-cholamidopropyl)dimethylammonio]-1-propanesulfonate (CHAPS), 65 mM dithiothreitol (DTT), and 1% (v/v) protease inhibitor cocktail.

### One-dimensional SDS-PAGE and in-gel digestion

In brief, proteins extracted from the 2700 MII oocytes were dissolved in SDS-PAGE loading buffer, boiled for 5 min, and loaded in a single lane on a 1-mm-thick 10% polyacrylamide gel. After separation, the gel was visualized by silver staining according to a published procedure [50], except that glutaraldehyde was omitted in the sensitizing solution. Thereafter, the gel was cut into 29 slices, and each slice was cut into 1-mm^3 ^gel particles for in-gel digestion. In-gel digestion was performed as follows: gel particles were washed 3 times in deionized water and subsequently dehydrated with 100% acetonitrile (ACN) for 10 min. The particles were incubated with 10 mM DTT in 25 mM ammonium bicarbonate for 1 h at 56°C for protein reduction. The resulting free thiol (-SH) groups were subsequently alkylated by incubating the samples with 55 mM iodoacetamide in 25 mM ammonium bicarbonate for 45 min in the dark. Gels were washed with 25 mM ammonium bicarbonate and 50% ACN solution and dehydrated with 100% ACN sequentially. The gel pieces were rehydrated with 10 ng/μl trypsin (Promega, Madison, WI, USA) in 25 mM ammonium bicarbonate and incubated for 12 h at 37°C for protein digestion. Supernatants were transferred to fresh tubes, and the remaining peptides were extracted by incubating the gel pieces twice with 30% ACN in 3% trifluoroacetic acid (TFA), followed by dehydration with 100% ACN. The extracts were combined and lyophilized to dryness, and the resulting peptides were used for mass spectrometric analysis.

### Online reverse-phase LC-MS/MS

For capillary reverse-phase LC (cLC) and mass spectrometric analysis, 29 fractions were sequentially loaded onto a Michrom peptide CapTrap (MW 0.5-50 kD, 0.5 × 2 mm; Michrom BioResources, Inc., Auburn, CA) at a flow rate of 50 μl/min with buffer A (see below). The trap column effluent was then transferred to a reverse-phase microcapillary column (0.1 × 150 mm, packed with Magic C18, 5 μm, 100 Å; Michrom Bioresources, Auburn, CA). The reverse-phase separation of peptides was performed using the following buffers: 5% ACN, 0.1% formic acid (buffer A) and 95% ACN, 0.1% formic acid (buffer B); a 56-min gradient (5-45% buffer B for 41 min, 90% buffer B for 5 min, and 5% buffer B for 10 min) was used. Peptide analysis was performed using Finnigan LTQ ORBitrap (ThermoFinnigan, San Jose, CA) coupled directly to an LC column. An MS survey scan was obtained for the m/z range 400-1800, and MS/MS spectra were acquired from the survey scan for the 10 most intense ions (as determined by Xcaliber mass spectrometer software in real time). Dynamic mass exclusion windows of 60 s were used, and siloxane (m/z 445.120025) was used as an internal standard.

### Database search and bioinformatics

DTA files (Bioworks version 3.3) in ASCII format for each MS/MS spectrum with a minimum ion count of 8 were generated from the raw data for the peptide mass range of 400-8,000. The resulting spectra were independently searched against the International Protein Index Mouse database (ipi.MOUSE.v3.30, downloaded from http://ftp.ebi.ac.uk/pub/databases/IPI) containing 56450 entries by using SEQUEST analysis software (Bioworks version 3.3, ThermoFinnigan). Carbamidomethylation of cysteine was set as a fixed modification, and oxidized methionine was sought as a variable modification. The initial mass tolerances for protein identification on MS and MS/MS peaks were 10 ppm and 0.6 Da, respectively. Two missed cleavages were permitted. The criteria used for filtering peptides with low confidence scores were the following: cross-correlation values (Xcorr) greater than 2.0 and 2.5 were used for doubly charged ions and triply or higher charged ions, respectively; ΔCn values (difference in Xcorr with the next highest value) less than 0.1 were removed from the matched sequences. Singly charged ions were discarded because they were small in number. All output files were searched against the forward and reversed IPI mouse database separately, and FDR for all peptide-to-spectrum matches was calculated as FDR = # of False peptides/(# of True peptides + # of False peptides).

For bioinformatics analysis, each IPI accession number was converted to an Entrez Gene ID according to the IPI protein cross-references file downloaded from http://ftp.ebi.ac.uk/pub/databases/IPI. We used Babelomics to find statistically over- and underrepresented GO categories in our oocyte proteome dataset. To compare the mouse oocyte proteome with other mouse tissue proteomes, we generated a combined database for the mouse based on the following mouse tissues and cell cultures characterized by LC-MS/MS: mouse heart [51], liver [51-53], brain [51,54], lung [51,55], kidney [51], spleen [56], placenta[51], cortical neurons cell culture[57], sperm [58], islet alpha-cell culture [59]. For enrichment analysis, our identified oocyte proteome was set as a test dataset and the combined mouse proteome was set as a reference. The enrichment analysis was done using 'fisher exact test', and all GO terms that were significant with adjusted *P *< 0.05 (after correcting for multiple term testing by using the FDR procedure of Bonferroni-Hochberg) were selected as overrepresented. An analysis of cellular processes influenced by the protein profile obtained was performed using PathwayStudio (v5.00) software (Ariadne Genomics, Inc., Rockville, MD). PathwayStudio includes an automated text-mining tool which enables the software to generate pathways from the PubMed database and other public sources. Each identified cellular process was confirmed through the PubMed/Medline hyperlink embedded in each node. The domain annotations were assigned using the Pfam database.

## Authors' contributions

PZ performed the LC-MS/MS experiments, data analysis, as well as data interpretation and contributed to the writing of the manuscript. NJ and YG contributed to the revision of the manuscript. XG contributed to the bioinformatic processing of the data. YW performed the sample preparation. ZZ, RH and JS supervised the experimental design and contributed to the data interpretation and manuscript evaluation. All authors read and approved the final manuscript.

## Supplementary Material

Additional file 1 **Identified mouse oocyte proteins**. Contains a list of all identified proteins from mouse MII oocytes in this study. Peptide information and spectrum information are also included. Click here for file

Additional file 2 **Data from single peptide-based identification of oocyte proteins**. Contains MS/MS spectra and fragment tables of single peptide-based identifications. Click here for file

Additional file 3 **Data from single peptide-based identification of oocyte proteins**. Contains MS/MS spectra and fragment tables of single peptide-based identifications. Click here for file

Additional file 4 **Data from single peptide-based identification of oocyte proteins**. Contains MS/MS spectra and fragment tables of single peptide-based identifications. Click here for file

Additional file 5 **Proteins of particular interest**. Contains a list of high-abundance proteins, proteins with high mRNA expression levels, and proteins common to both the oocyte and the ES cell proteome. Click here for file
