# Supplementary material for: Proteomic-based identification of maternal proteins in mature mouse oocytes
Source: BMC Genomics. 2009 Aug 3;10:348. doi: 10.1186/1471-2164-10-348 (PMC2730056; doi:10.1186/1471-2164-10-348)
Supplement: Additional file 2 — Data from single peptide-based identification of oocyte proteins. Contains MS/MS spectra and fragment tables of single peptide-based identifications. [file 1471-2164-10-348-S2.pdf]

MS/MS spectra and fragment assignments of single peptide-based identifications.

Gene symbol: -
Protein name: 22 kDa protein
Protein accession numbers: IPI00762428
Peptide sequence: (R)LLQCDPSSASQF
Exclusive (unique to this protein): TRUE
SEQUEST XCorr score: 2.13 SEQUEST DCn score: 0.662
Fix modifications: C4: Carbamidomethyl (+57.02)
Variable modifications: None
charge: 2 Observed m/z: 676.8119
Actual minus calculated peptide mass (AMU): 0.0002441

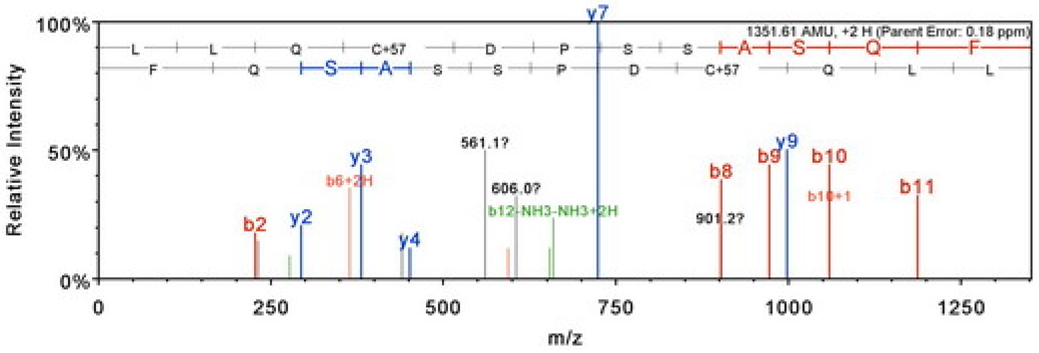

B	B Ions	B+2H	B-NH3	B-H2O	AA	Y Ions	Y+2H	Y-NH3	Y-H2O	Y
1	114.1				L	1352.6		1335.6	1334.6	12
2	227.2				L	1239.5		1222.5	1221.5	11
3	355.2		338.2		Q	1126.5		1109.4	1108.4	10
4	515.3		498.2		C+57	998.4		981.4	980.4	9
5	630.3		613.3	612.3	D	838.4		821.3	820.4	8
6	727.4	364.2	710.3	709.3	P	723.3		706.3	705.3	7
7	814.4	407.7	797.4	796.4	S	626.3		609.3	608.3	6
8	901.4	451.2	884.4	883.4	S	539.3		522.2	521.2	5
9	972.5	486.7	955.4	954.4	A	452.2		435.2	434.2	4
10	1059.5	530.2	1042.5	1041.5	S	381.2		364.2	363.2	3
11	1187.5	594.3	1170.5	1169.5	Q	294.2		277.1		2
12	1352.6	676.8	1335.6	1334.6	F	166.1				1

Gene symbol: -
Protein name: 23 kDa protein
Protein accession numbers: IPI00761220
Peptide sequence: (R)VLNSYWVGEDSTYK(V)
Exclusive (unique to this protein): TRUE

SEQUEST XCorr score: 2.25 SEQUEST DCn score: 0.681
Fix modifications: None
Variable modifications: None
charge: 2 Observed m/z: 830.8931
Actual minus calculated peptide mass (AMU): -0.0074454

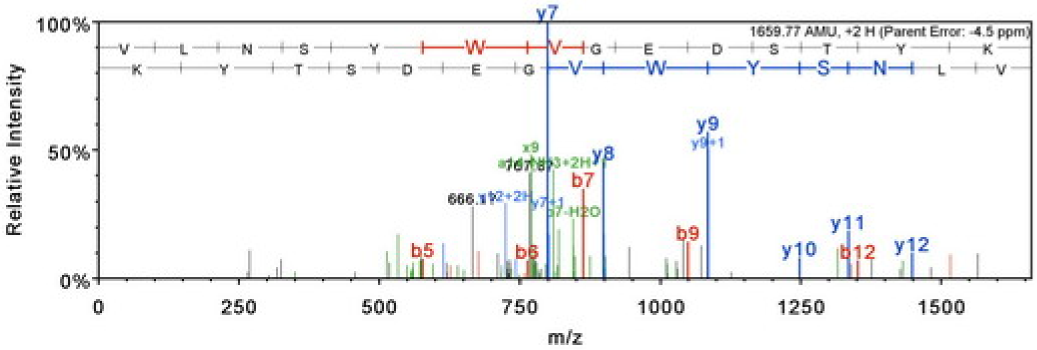

B	B Ions	B+2H	B-NH3	B-H2O	AA	Y Ions	Y+2H	Y-NH3	Y-H2O	Y
1	100.1				V	1660.8	830.9	1643.8	1642.8	14
2	213.2				L	1561.7	781.4	1544.7	1543.7	13
3	327.2		310.2		N	1448.6	724.8	1431.6	1430.6	12
4	414.2		397.2	396.2	S	1334.6	667.8	1317.6	1316.6	11
5	577.3		560.3	559.3	Y	1247.6	624.3	1230.5	1229.5	10
6	763.4	382.2	746.4	745.4	W	1084.5	542.8	1067.5	1066.5	9
7	862.5	431.7	845.4	844.4	V	898.4	449.7	881.4	880.4	8
8	919.5	460.2	902.4	901.5	G	799.4	400.2	782.3	781.3	7
9	1048.5	524.8	1031.5	1030.5	E	742.3	371.7	725.3	724.3	6
10	1163.5	582.3	1146.5	1145.5	D	613.3		596.3	595.3	5
11	1250.6	625.8	1233.5	1232.6	S	498.3		481.2	480.3	4
12	1351.6	676.3	1334.6	1333.6	T	411.2		394.2	393.2	3
13	1514.7	757.8	1497.7	1496.7	Y	310.2		293.2		2
14	1660.8	830.9	1643.8	1642.8	K	147.1		130.1		1

Gene symbol: -
Protein name: 27 kDa protein
Protein accession numbers: IPI00625569
Peptide sequence: (K)SPYQEFTDHLVK(T)
Exclusive (unique to this protein): TRUE
SEQUEST XCorr score: 2.47 SEQUEST DCn score: 0.527
Fix modifications: None
Variable modifications: None
charge: 2 Observed m/z: 732.3629
Actual minus calculated peptide mass (AMU): 0.0009766

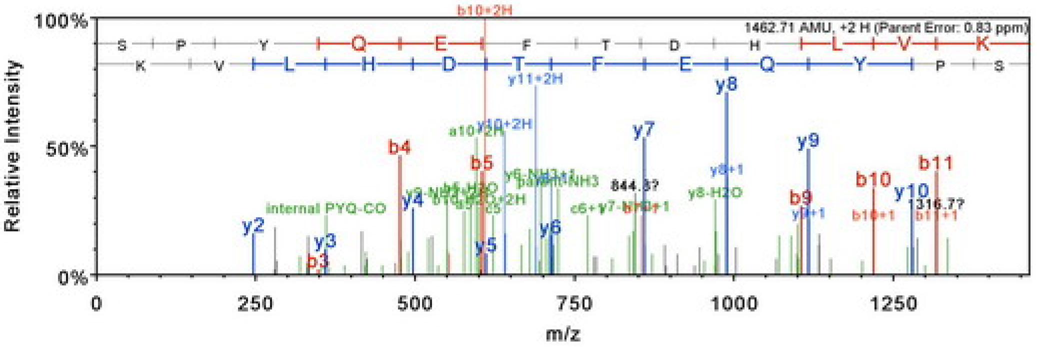

B	B Ions	B+2H	B-NH3	B-H2O	AA	Y Ions	Y+2H	Y-NH3	Y-H2O	Y
1	88.0			70.0	S	1463.7	732.4	1446.7	1445.7	12
2	185.1			167.1	P	1376.7	688.9	1359.7	1358.7	11
3	348.2			330.2	Y	1279.6	640.3	1262.6	1261.6	10
4	476.2		459.2	458.2	Q	1116.6	558.8	1099.5	1098.6	9
5	605.3		588.2	587.3	E	988.5	494.8	971.5	970.5	8
6	752.3	376.7	735.3	734.3	F	859.5	430.2	842.4	841.5	7
7	853.4	427.2	836.4	835.4	T	712.4	356.7	695.4	694.4	6
8	968.4	484.7	951.4	950.4	D	611.4	306.2	594.3	593.3	5
9	1105.5	553.2	1088.4	1087.5	H	496.3	248.7	479.3		4
10	1218.5	609.8	1201.5	1200.5	L	359.3		342.2		3
11	1317.6	659.3	1300.6	1299.6	V	246.2		229.2		2
12	1463.7	732.4	1446.7	1445.7	K	147.1		130.1		1

Gene symbol: -
Protein name: 29 kDa protein
Protein accession numbers: IPI00626848
Peptide sequence: (R)IVEPYIAWGYPNLK(S)
Exclusive (unique to this protein): TRUE
SEQUEST XCorr score: 2.11 SEQUEST DCn score: 0.52
Fix modifications: None
Variable modifications: None
charge: 2 Observed m/z: 831.9464
Actual minus calculated peptide mass (AMU): -0.004639

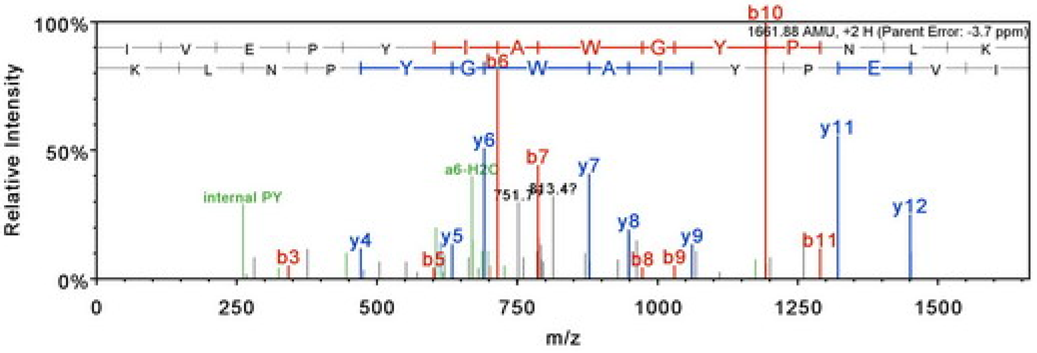

B	B Ions	B+2H	B-NH3	B-H2O	AA	Y Ions	Y+2H	Y-NH3	Y-H2O	Y
1	114.1				I	1662.9	832.0	1645.9	1644.9	14
2	213.2				V	1549.8	775.4	1532.8	1531.8	13
3	342.2			324.2	E	1450.7	725.9	1433.7	1432.7	12
4	439.3			421.3	P	1321.7	661.4	1304.7		11
5	602.3			584.3	Y	1224.6	612.8	1207.6		10
6	715.4	358.2		697.4	I	1061.6	531.3	1044.5		9
7	786.4	393.7		768.4	A	948.5	474.8	931.5		8
8	972.5	486.8		954.5	W	877.5	439.2	860.4		7
9	1029.5	515.3		1011.5	G	691.4	346.2	674.4		6
10	1192.6	596.8		1174.6	Y	634.4		617.3		5
11	1289.7	645.3		1271.7	P	471.3		454.3		4
12	1403.7	702.4	1386.7	1385.7	N	374.2		357.2		3
13	1516.8	758.9	1499.8	1498.8	L	260.2		243.2		2
14	1662.9	832.0	1645.9	1644.9	K	147.1		130.1		1

Gene symbol: -

Protein name: similar to Glyceraldehyde-3-phosphate dehydrogenase (GAPDH) isoform 1

Protein accession numbers: IPI00849530

Peptide sequence: (R)VPTPNVSIVDLTCR(L)

Exclusive (unique to this protein): TRUE

SEQUEST XCorr score: 2.03 SEQUEST DCn score: 0.442

Fix modifications: C13: Carbamidomethyl (+57.02)

Variable modifications: None

charge: 2 Observed m/z: 785.9143

Actual minus calculated peptide mass (AMU): -0.0056153

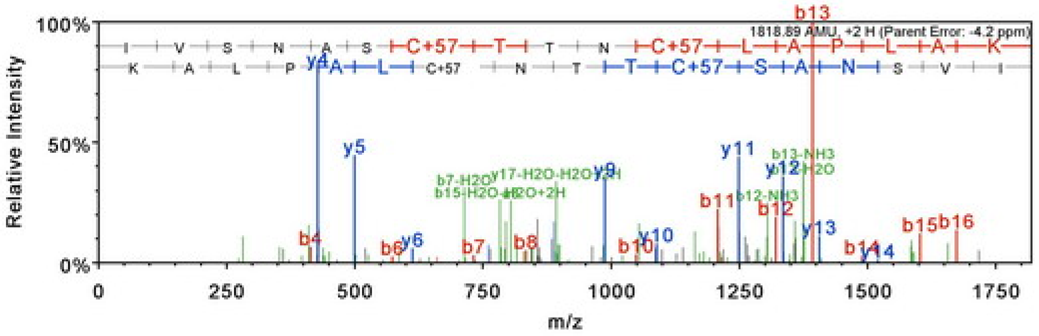

B	B Ions	B+2H	B-NH3	B-H2O	AA	Y Ions	Y+2H	Y-NH3	Y-H2O	Y
1	114.1				I	1819.9	910.5	1802.9	1801.9	17
2	213.2				V	1706.8	853.9	1689.8	1688.8	16
3	300.2			282.2	S	1607.8	804.4	1590.7	1589.7	15
4	414.2		397.2	396.2	N	1520.7	760.9	1503.7	1502.7	14
5	485.3		468.3	467.3	A	1406.7	703.8	1389.7	1388.7	13
6	572.3	286.7	555.3	554.3	S	1335.6	668.3	1318.6	1317.6	12
7	732.3	366.7	715.3	714.3	C+57	1248.6	624.8	1231.6	1230.6	11
8	833.4	417.2	816.4	815.4	T	1088.6	544.8	1071.5	1070.6	10
9	934.4	467.7	917.4	916.4	T	987.5	494.3	970.5	969.5	9
10	1048.5	524.7	1031.5	1030.5	N	886.5	443.8	869.5		8
11	1208.5	604.8	1191.5	1190.5	C+57	772.4	386.7	755.4		7
12	1321.6	661.3	1304.6	1303.6	L	612.4	306.7	595.4		6
13	1392.6	696.8	1375.6	1374.6	A	499.3		482.3		5
14	1489.7	745.3	1472.7	1471.7	P	428.3		411.3		4
15	1602.8	801.9	1585.7	1584.8	L	331.2		314.2		3
16	1673.8	837.4	1656.8	1655.8	A	218.2		201.1		2
17	1819.9	910.5	1802.9	1801.9	K	147.1		130.1		1

Gene symbol: -

Protein name: similar to ribosomal protein L21

Protein accession numbers: IPI00661977

Peptide sequence: (R)VYNVTQHAVGIIVNK(Q)

Exclusive (unique to this protein): TRUE

SEQUEST XCorr score: 2.01 SEQUEST DCn score: 0.682

Fix modifications: None

Variable modifications: None

charge: 2 Observed m/z: 827.9692

Actual minus calculated peptide mass (AMU): 0.002686

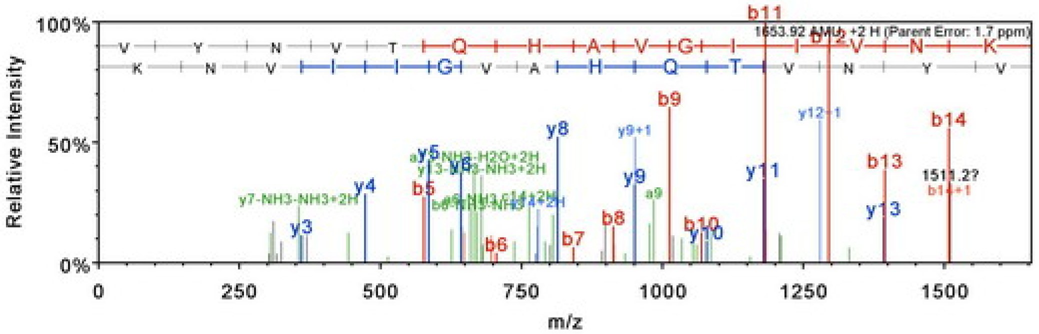

B	B Ions	B+2H	B-NH3	B-H2O	AA	Y Ions	Y+2H	Y-NH3	Y-H2O	Y
1	100.1				V	1654.9	828.0	1637.9	1636.9	15
2	263.1				Y	1555.9	778.4	1538.8	1537.9	14
3	377.2		360.2		N	1392.8	696.9	1375.8	1374.8	13
4	476.3		459.2		V	1278.8	639.9	1261.7	1260.7	12
5	577.3		560.3	559.3	T	1179.7	590.4	1162.7	1161.7	11
6	705.4	353.2	688.3	687.4	Q	1078.6	539.8	1061.6		10
7	842.4	421.7	825.4	824.4	H	950.6	475.8	933.6		9
8	913.5	457.2	896.4	895.4	A	813.5	407.3	796.5		8
9	1012.5	506.8	995.5	994.5	V	742.5	371.8	725.5		7
10	1069.5	535.3	1052.5	1051.5	G	643.4	322.2	626.4		6
11	1182.6	591.8	1165.6	1164.6	I	586.4		569.4		5
12	1295.7	648.4	1278.7	1277.7	I	473.3		456.3		4
13	1394.8	697.9	1377.8	1376.8	V	360.2		343.2		3
14	1508.8	754.9	1491.8	1490.8	N	261.2		244.1		2
15	1654.9	828.0	1637.9	1636.9	K	147.1		130.1		1

Gene symbol: 0610008C08Rik

Protein name: similar to Novel transmembrane domain containing protein

Protein accession numbers: IPI00807819

Peptide sequence: (R)TQLEENISQLR(H)

Exclusive (unique to this protein): TRUE

SEQUEST XCorr score: 2.47 SEQUEST DCn score: 0.442

Fix modifications: None

Variable modifications: None

charge: 2 Observed m/z: 665.8524

Actual minus calculated peptide mass (AMU): 0.0002441

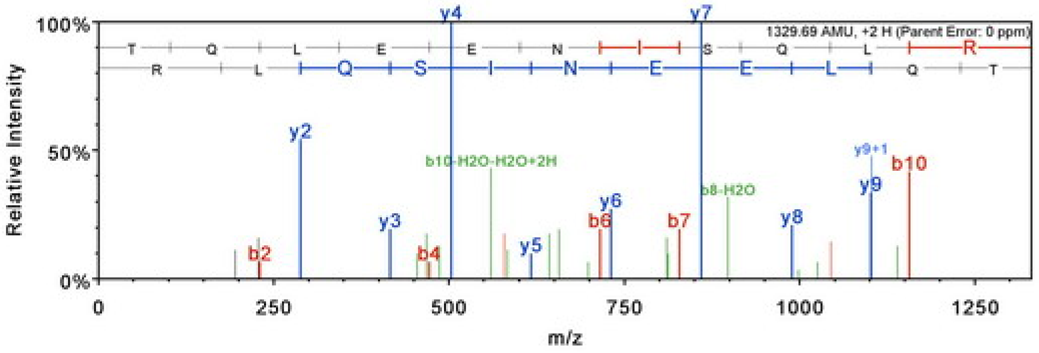

B	B Ions	B+2H	B-NH3	B-H2O	AA	Y Ions	Y+2H	Y-NH3	Y-H2O	Y
1	102.1			84.0	T	1330.7	665.9	1313.7	1312.7	11
2	230.1		213.1	212.1	Q	1229.7	615.3	1212.6	1211.6	10
3	343.2		326.2	325.2	L	1101.6	551.3	1084.6	1083.6	9
4	472.2		455.2	454.2	E	988.5	494.8	971.5	970.5	8
5	601.3		584.3	583.3	E	859.5	430.2	842.4	841.5	7
6	715.3	358.2	698.3	697.3	N	730.4	365.7	713.4	712.4	6
7	828.4	414.7	811.4	810.4	I	616.4		599.4	598.4	5
8	915.4	458.2	898.4	897.4	S	503.3		486.3	485.3	4
9	1043.5	522.3	1026.5	1025.5	Q	416.3		399.2		3
10	1156.6	578.8	1139.6	1138.6	L	288.2		271.2		2
11	1330.7	665.9	1313.7	1312.7	R	175.1		158.1		1

Gene symbol: 1110007L15Rik
Protein name: Isoform 1 of UPF0363 protein C7orf20 homolog
Protein accession numbers: IPI00272521,IPI00742325
Peptide sequence: (K)AEVDVADELLENLAK(V)
Exclusive (unique to this protein): TRUE
SEQUEST XCorr score: 2.28 SEQUEST DCn score: 0.725
Fix modifications: None
Variable modifications: None
charge: 2 Observed m/z: 814.9244
Actual minus calculated peptide mass (AMU): 0.002564

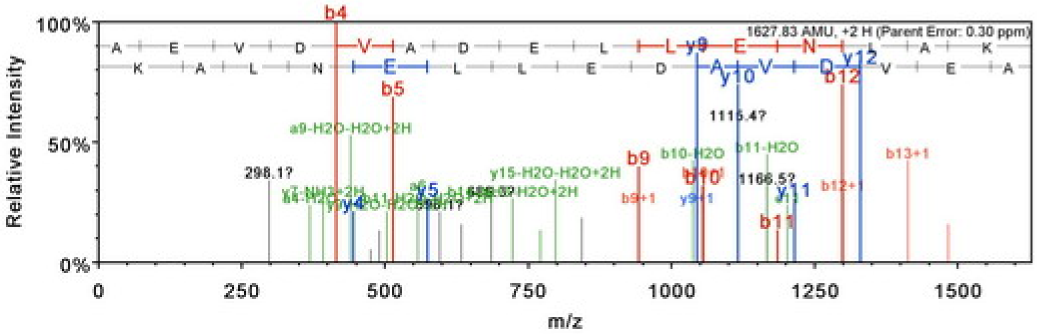

B	B Ions	B+2H	B-NH3	B-H2O	AA	Y Ions	Y+2H	Y-NH3	Y-H2O	Y
1	72.0				A	1628.8	814.9	1611.8	1610.8	15
2	201.1			183.1	E	1557.8	779.4	1540.8	1539.8	14
3	300.2			282.2	V	1428.8	714.9	1411.7	1410.8	13
4	415.2			397.2	D	1329.7	665.4	1312.7	1311.7	12
5	514.3			496.2	V	1214.7	607.8	1197.6	1196.7	11
6	585.3	293.2		567.3	A	1115.6	558.3	1098.6	1097.6	10
7	700.3	350.7		682.3	D	1044.6	522.8	1027.5	1026.5	9
8	829.4	415.2		811.4	E	929.5	465.3	912.5	911.5	8
9	942.4	471.7		924.4	L	800.5	400.8	783.5	782.5	7
10	1055.5	528.3		1037.5	L	687.4	344.2	670.4	669.4	6
11	1184.6	592.8		1166.6	E	574.3		557.3	556.3	5
12	1298.6	649.8	1281.6	1280.6	N	445.3		428.3		4
13	1411.7	706.4	1394.7	1393.7	L	331.2		314.2		3
14	1482.7	741.9	1465.7	1464.7	A	218.2		201.1		2
15	1628.8	814.9	1611.8	1610.8	K	147.1		130.1		1

Gene symbol: 1500019G21Rik
Protein name: Hsp70-binding protein 1
Protein accession numbers: IPI00120257
Peptide sequence: (R)EQEAGLLQFLR(L)
Exclusive (unique to this protein): TRUE
SEQUEST XCorr score: 2 SEQUEST DCn score: 0.49
Fix modifications: None
Variable modifications: None

charge: 2 Observed m/z: 652.3553

Actual minus calculated peptide mass (AMU): 0.001709

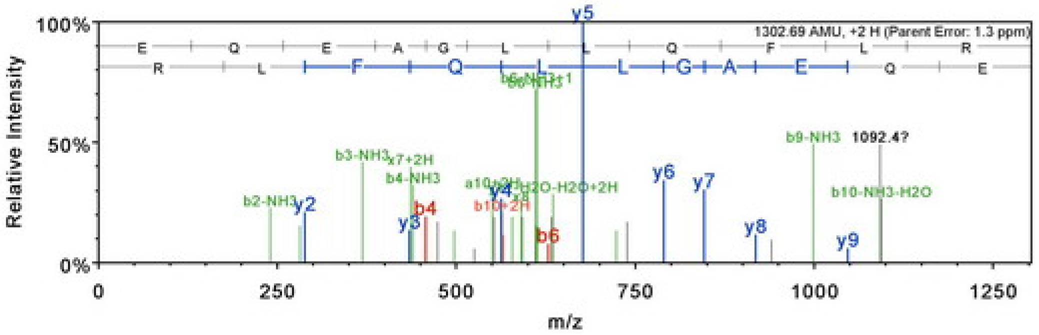

B	B Ions	B+2H	B-NH3	B-H2O	AA	Y Ions	Y+2H	Y-NH3	Y-H2O	Y
1	130.1			112.0	E	1303.7	652.4	1286.7	1285.7	11
2	258.1		241.1	240.1	Q	1174.7	587.8	1157.6	1156.7	10
3	387.2		370.1	369.1	E	1046.6	523.8	1029.6	1028.6	9
4	458.2		441.2	440.2	A	917.6	459.3	900.5		8
5	515.2		498.2	497.2	G	846.5	423.8	829.5		7
6	628.3	314.7	611.3	610.3	L	789.5	395.3	772.5		6
7	741.4	371.2	724.4	723.4	L	676.4		659.4		5
8	869.4	435.2	852.4	851.4	Q	563.3		546.3		4
9	1016.5	508.8	999.5	998.5	F	435.3		418.3		3
10	1129.6	565.3	1112.6	1111.6	L	288.2		271.2		2
11	1303.7	652.4	1286.7	1285.7	R	175.1		158.1		1

Gene symbol: 1700012G19Rik

Protein name: RIKEN cDNA 1700012G19 gene

Protein accession numbers: IPI00380195

Peptide sequence: (R)FIFDCVSQEYGINPER(T)

Exclusive (unique to this protein): TRUE

SEQUEST XCorr score: 2.31 SEQUEST DCn score: 0.71

Fix modifications: C5: Carbamidomethyl (+57.02)

Variable modifications: None

charge: 2 Observed m/z: 987.4552

Actual minus calculated peptide mass (AMU): -0.004273

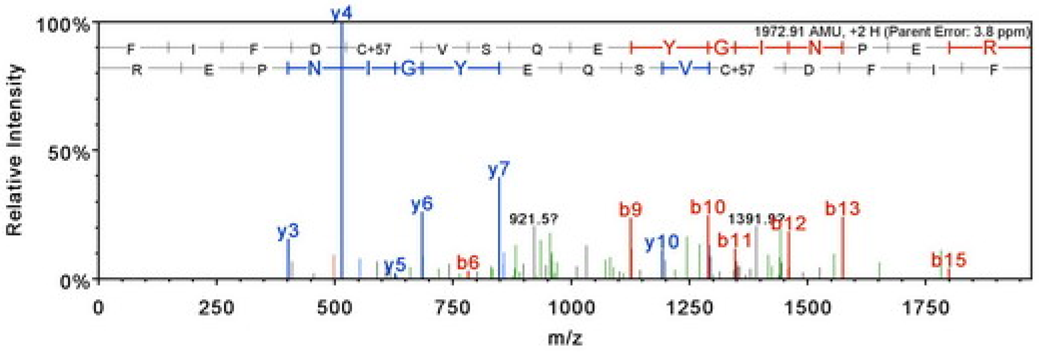

B	B Ions	B+2H	B-NH3	B-H2O	AA	Y Ions	Y+2H	Y-NH3	Y-H2O	Y
1	148.1				F	1973.9	987.5	1956.9	1955.9	16
2	261.2				I	1826.8	913.9	1809.8	1808.8	15
3	408.2				F	1713.8	857.4	1696.7	1695.7	14
4	523.3			505.3	D	1566.7	783.9	1549.7	1548.7	13
5	683.3			665.3	C+57	1451.7	726.3	1434.6	1433.7	12
6	782.4	391.7		764.3	V	1291.6	646.3	1274.6	1273.6	11
7	869.4	435.2		851.4	S	1192.6	596.8	1175.5	1174.5	10
8	997.5	499.2	980.4	979.4	Q	1105.5	553.3	1088.5	1087.5	9
9	1126.5	563.8	1109.5	1108.5	E	977.5	489.2	960.4	959.5	8
10	1289.5	645.3	1272.5	1271.5	Y	848.4	424.7	831.4	830.4	7
11	1346.6	673.8	1329.5	1328.6	G	685.4	343.2	668.3	667.4	6
12	1459.7	730.3	1442.6	1441.7	I	628.3		611.3	610.3	5
13	1573.7	787.4	1556.7	1555.7	N	515.3		498.2	497.3	4
14	1670.8	835.9	1653.7	1652.7	P	401.2		384.2	383.2	3
15	1799.8	900.4	1782.8	1781.8	E	304.2		287.1	286.2	2
16	1973.9	987.5	1956.9	1955.9	R	175.1		158.1		1

Gene symbol: 2410014A08Rik

Protein name: Uncharacterized protein KIAA0152 precursor

Protein accession numbers: IPI00312018

Peptide sequence: (R)STPEDQILYQTER(Y)

Exclusive (unique to this protein): TRUE

SEQUEST XCorr score: 3.15 SEQUEST DCn score: 0.657

Fix modifications: None

Variable modifications: None

charge: 2 Observed m/z: 790.3846

Actual minus calculated peptide mass (AMU): 0.0008545

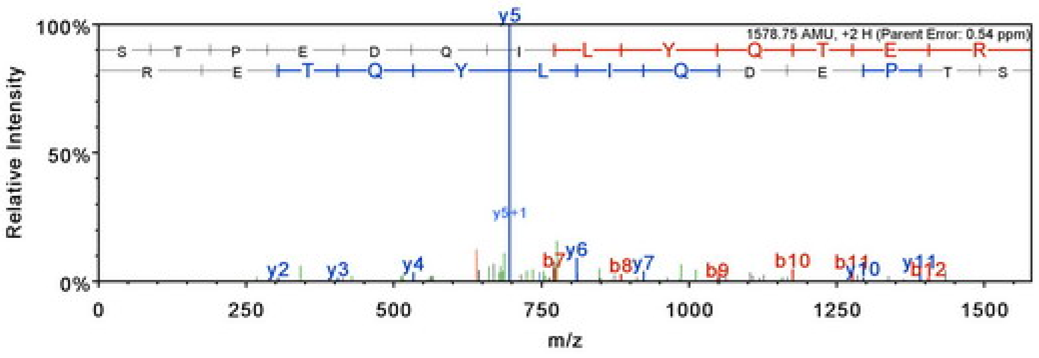

B	B Ions	B+2H	B-NH3	B-H2O	AA	Y Ions	Y+2H	Y-NH3	Y-H2O	Y
1	88.0			70.0	S	1579.8	790.4	1562.7	1561.8	13
2	189.1			171.1	T	1492.7	746.9	1475.7	1474.7	12
3	286.1			268.1	P	1391.7	696.3	1374.7	1373.7	11
4	415.2			397.2	E	1294.6	647.8	1277.6	1276.6	10
5	530.2			512.2	D	1165.6	583.3	1148.6	1147.6	9
6	658.3	329.6	641.2	640.3	Q	1050.6	525.8	1033.5	1032.5	8
7	771.4	386.2	754.3	753.3	I	922.5	461.8	905.5	904.5	7
8	884.4	442.7	867.4	866.4	L	809.4	405.2	792.4	791.4	6
9	1047.5	524.3	1030.5	1029.5	Y	696.3		679.3	678.3	5
10	1175.6	588.3	1158.5	1157.5	Q	533.3		516.2	515.3	4
11	1276.6	638.8	1259.6	1258.6	T	405.2		388.2	387.2	3
12	1405.7	703.3	1388.6	1387.6	E	304.2		287.1	286.2	2
13	1579.8	790.4	1562.7	1561.8	R	175.1		158.1		1

Gene symbol: 2610019P18Rik

Protein name: 10 days embryo whole body cDNA, RIKEN full-length enriched library, clone:2610019P18 product:hypothetical protein, full insert sequence

Protein accession numbers: IPI00224957

Peptide sequence: (K)ISDGQEEADDEEEEEEEEEITK(T)

Exclusive (unique to this protein): TRUE

SEQUEST XCorr score: 2.41 SEQUEST DCn score: 0.793

Fix modifications: None

Variable modifications: None

charge: 2 Observed m/z: 1227.0017

Actual minus calculated peptide mass (AMU): 0.010458

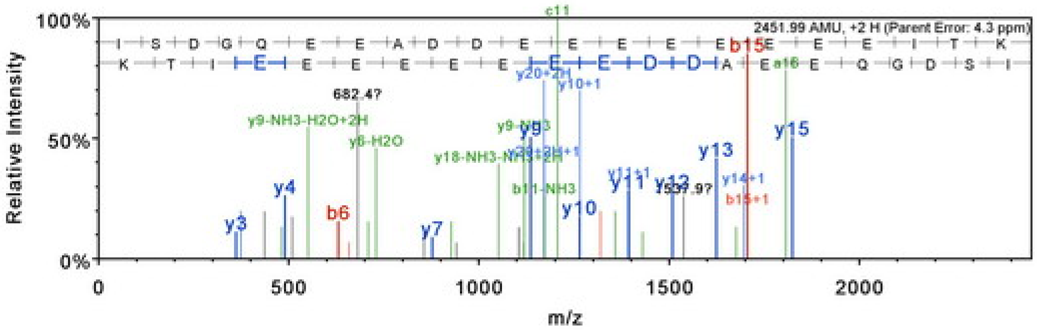

B	B Ions	B+2H	B-NH3	B-H2O	AA	Y Ions	Y+2H	Y-NH3	Y-H2O	Y
1	114.1				I	2453.0	1227.0	2436.0	2435.0	21
2	201.1			183.1	S	2339.9	1170.5	2322.9	2321.9	20
3	316.2			298.1	D	2252.9	1126.9	2235.8	2234.9	19
4	373.2			355.2	G	2137.8	1069.4	2120.8	2119.8	18
5	501.2		484.2	483.2	Q	2080.8	1040.9	2063.8	2062.8	17
6	630.3	315.6	613.3	612.3	E	1952.8	976.9	1935.7	1934.8	16
7	759.3	380.2	742.3	741.3	E	1823.7	912.4	1806.7	1805.7	15
8	830.4	415.7	813.3	812.3	A	1694.7	847.8	1677.7	1676.7	14
9	945.4	473.2	928.4	927.4	D	1623.6	812.3	1606.6	1605.6	13
10	1060.4	530.7	1043.4	1042.4	D	1508.6	754.8	1491.6	1490.6	12
11	1189.5	595.2	1172.4	1171.4	E	1393.6	697.3	1376.6	1375.6	11
12	1318.5	659.8	1301.5	1300.5	E	1264.5	632.8	1247.5	1246.5	10
13	1447.5	724.3	1430.5	1429.5	E	1135.5	568.3	1118.5	1117.5	9
14	1576.6	788.8	1559.5	1558.6	E	1006.5	503.7	989.4	988.5	8
15	1705.6	853.3	1688.6	1687.6	E	877.4	439.2	860.4	859.4	7
16	1834.7	917.8	1817.6	1816.7	E	748.4	374.7	731.4	730.4	6
17	1963.7	982.4	1946.7	1945.7	E	619.3		602.3	601.3	5
18	2092.8	1046.9	2075.7	2074.7	E	490.3		473.3	472.3	4
19	2205.8	1103.4	2188.8	2187.8	I	361.3		344.2	343.2	3
20	2306.9	1153.9	2289.8	2288.9	T	248.2		231.1	230.2	2
21	2453.0	1227.0	2436.0	2435.0	K	147.1		130.1		1

Gene symbol: 2610030H06Rik

Protein name: ES cells cDNA, RIKEN full-length enriched library, clone:C330020E23 product:similar to RNA BINDING PROTEIN

Protein accession numbers: IPI00132334,IPI00652117,IPI00762458

Peptide sequence: (K)AALNALQPPEFR(N)

Exclusive (unique to this protein): TRUE

SEQUEST XCorr score: 2.15 SEQUEST DCn score: 0.582

Fix modifications: None

Variable modifications: None

charge: 2 Observed m/z: 663.8586

Actual minus calculated peptide mass (AMU): -0.007568

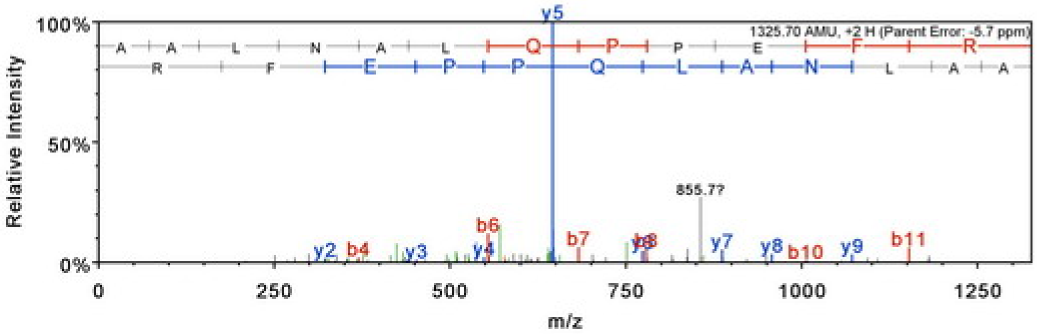

B	B Ions	B+2H	B-NH3	B-H2O	AA	Y Ions	Y+2H	Y-NH3	Y-H2O	Y
1	72.0				A	1326.7	663.9	1309.7	1308.7	12
2	143.1				A	1255.7	628.3	1238.7	1237.7	11
3	256.2				L	1184.6	592.8	1167.6	1166.6	10
4	370.2		353.2		N	1071.6	536.3	1054.5	1053.5	9
5	441.3		424.2		A	957.5	479.3	940.5	939.5	8
6	554.3	277.7	537.3		L	886.5	443.7	869.5	868.5	7
7	682.4	341.7	665.4		Q	773.4	387.2	756.4	755.4	6
8	779.4	390.2	762.4		P	645.3		628.3	627.3	5
9	876.5	438.8	859.5		P	548.3		531.3	530.3	4
10	1005.5	503.3	988.5	987.5	E	451.2		434.2	433.2	3
11	1152.6	576.8	1135.6	1134.6	F	322.2		305.2		2
12	1326.7	663.9	1309.7	1308.7	R	175.1		158.1		1

Gene symbol: 4930420K17Rik

Protein name: similar to chromosome 7 open reading frame 23 isoform 2

Protein accession numbers: IPI00752344,IPI00754821,IPI00755055

Peptide sequence: (R)TYGTSGLDNRPLFGETSAK(D)

Exclusive (unique to this protein): TRUE

SEQUEST XCorr score: 2.39 SEQUEST DCn score: 0.671

Fix modifications: None

Variable modifications: None

charge: 2 Observed m/z: 1007.5013

Actual minus calculated peptide mass (AMU): 0.0063456

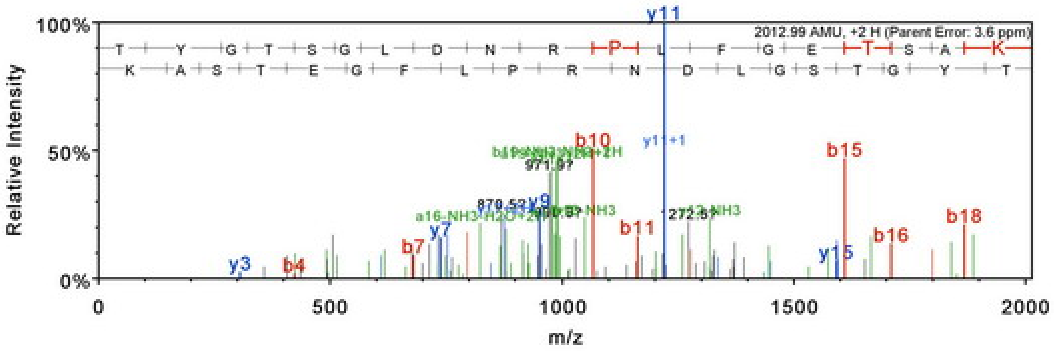

B	B Ions	B+2H	B-NH3	B-H2O	AA	Y Ions	Y+2H	Y-NH3	Y-H2O	Y
1	102.1			84.0	T	2014.0	1007.5	1997.0	1996.0	19
2	265.1			247.1	Y	1912.9	957.0	1895.9	1894.9	18
3	322.1			304.1	G	1749.9	875.4	1732.9	1731.9	17
4	423.2			405.2	T	1692.9	846.9	1675.8	1674.9	16
5	510.2			492.2	S	1591.8	796.4	1574.8	1573.8	15
6	567.2	284.1		549.2	G	1504.8	752.9	1487.8	1486.8	14
7	680.3	340.7		662.3	L	1447.8	724.4	1430.7	1429.7	13
8	795.4	398.2		777.3	D	1334.7	667.8	1317.6	1316.7	12
9	909.4	455.2	892.4	891.4	N	1219.6	610.3	1202.6	1201.6	11
10	1065.5	533.3	1048.5	1047.5	R	1105.6	553.3	1088.6	1087.6	10
11	1162.5	581.8	1145.5	1144.5	P	949.5	475.3	932.5	931.5	9
12	1275.6	638.3	1258.6	1257.6	L	852.5	426.7	835.4	834.4	8
13	1422.7	711.9	1405.7	1404.7	F	739.4	370.2	722.3	721.4	7
14	1479.7	740.4	1462.7	1461.7	G	592.3	296.7	575.3	574.3	6
15	1608.8	804.9	1591.7	1590.8	E	535.3		518.3	517.3	5
16	1709.8	855.4	1692.8	1691.8	T	406.2		389.2	388.2	4
17	1796.9	898.9	1779.8	1778.8	S	305.2		288.2	287.2	3
18	1867.9	934.5	1850.9	1849.9	A	218.2		201.1		2
19	2014.0	1007.5	1997.0	1996.0	K	147.1		130.1		1

Gene symbol: Aco1

Protein name: Cytoplasmic aconitase

Protein accession numbers: IPI00622780

Peptide sequence: (R)YQQAGLPLIVLAGK(E)

Exclusive (unique to this protein): TRUE

SEQUEST XCorr score: 2.1 SEQUEST DCn score: 0.446

Fix modifications: None

Variable modifications: None

charge: 2 Observed m/z: 735.935

Actual minus calculated peptide mass (AMU): -0.00647

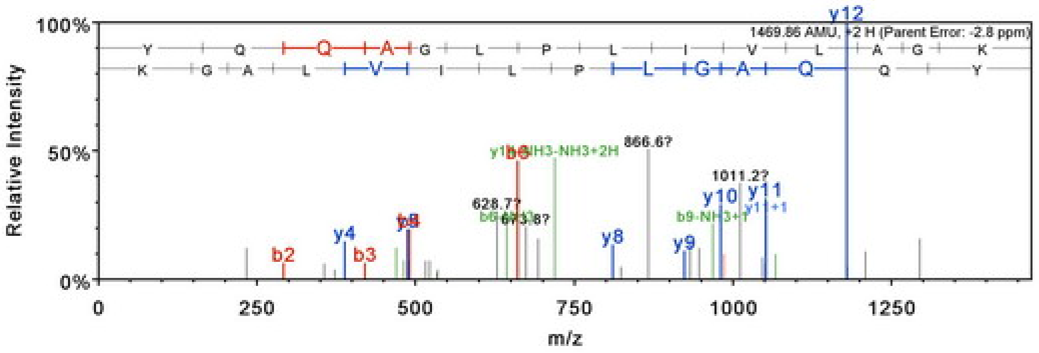

B	B Ions	B+2H	B-NH3	B-H2O	AA	Y Ions	Y+2H	Y-NH3	Y-H2O	Y
1	164.1				Y	1470.9	735.9	1453.8		14
2	292.1		275.1		Q	1307.8	654.4	1290.8		13
3	420.2		403.2		Q	1179.8	590.4	1162.7		12
4	491.2		474.2		A	1051.7	526.4	1034.7		11
5	548.3		531.2		G	980.7	490.8	963.6		10
6	661.3	331.2	644.3		L	923.6	462.3	906.6		9
7	758.4	379.7	741.4		P	810.6	405.8	793.5		8
8	871.5	436.2	854.4		L	713.5	357.3	696.5		7
9	984.6	492.8	967.5		I	600.4	300.7	583.4		6
10	1083.6	542.3	1066.6		V	487.3		470.3		5
11	1196.7	598.9	1179.7		L	388.3		371.2		4
12	1267.7	634.4	1250.7		A	275.2		258.1		3
13	1324.8	662.9	1307.7		G	204.1		187.1		2
14	1470.9	735.9	1453.8		K	147.1		130.1		1

Gene symbol: Acox3

Protein name: Acyl-coenzyme A oxidase 3, peroxisomal

Protein accession numbers: IPI00318108,IPI00404363

Peptide sequence: (R)TGNITSEGTYNSPFK(D)

Exclusive (unique to this protein): TRUE

SEQUEST XCorr score: 2.53 SEQUEST DCn score: 0.689

Fix modifications: None

Variable modifications: None

charge: 2 Observed m/z: 808.3798

Actual minus calculated peptide mass (AMU): -0.008789

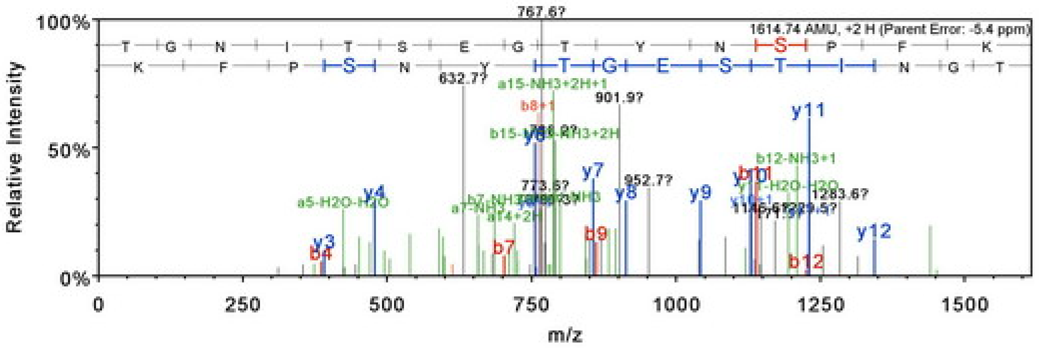

B	B Ions	B+2H	B-NH3	B-H2O	AA	Y Ions	Y+2H	Y-NH3	Y-H2O	Y
1	102.1			84.0	T	1615.8	808.4	1598.7	1597.8	15
2	159.1			141.1	G	1514.7	757.9	1497.7	1496.7	14
3	273.1		256.1	255.1	N	1457.7	729.4	1440.7	1439.7	13
4	386.2		369.2	368.2	I	1343.7	672.3	1326.6	1325.6	12
5	487.3		470.2	469.2	T	1230.6	615.8	1213.5	1212.5	11
6	574.3	287.7	557.3	556.3	S	1129.5	565.3	1112.5	1111.5	10
7	703.3	352.2	686.3	685.3	E	1042.5	521.8	1025.5	1024.5	9
8	760.4	380.7	743.3	742.3	G	913.4	457.2	896.4	895.4	8
9	861.4	431.2	844.4	843.4	T	856.4	428.7	839.4	838.4	7
10	1024.5	512.7	1007.4	1006.5	Y	755.4	378.2	738.4	737.4	6
11	1138.5	569.8	1121.5	1120.5	N	592.3		575.3	574.3	5
12	1225.5	613.3	1208.5	1207.5	S	478.3		461.2	460.3	4
13	1322.6	661.8	1305.6	1304.6	P	391.2		374.2		3
14	1469.7	735.3	1452.6	1451.6	F	294.2		277.2		2
15	1615.8	808.4	1598.7	1597.8	K	147.1		130.1		1

Gene symbol: Acta1

Protein name: Actin, alpha skeletal muscle

Protein accession numbers: IPI00110827,IPI00114593,IPI00480406,IPI00653007,IPI00654242

Peptide sequence: (K)YPIEHGIITNWDDMEK(I)

Exclusive (unique to this protein): TRUE

SEQUEST XCorr score: 2.01 SEQUEST DCn score: 0.533

Fix modifications: None

Variable modifications: M14: Oxidation (+16.00)

charge: 2 Observed m/z: 988.9535

Actual minus calculated peptide mass (AMU): -0.0074454

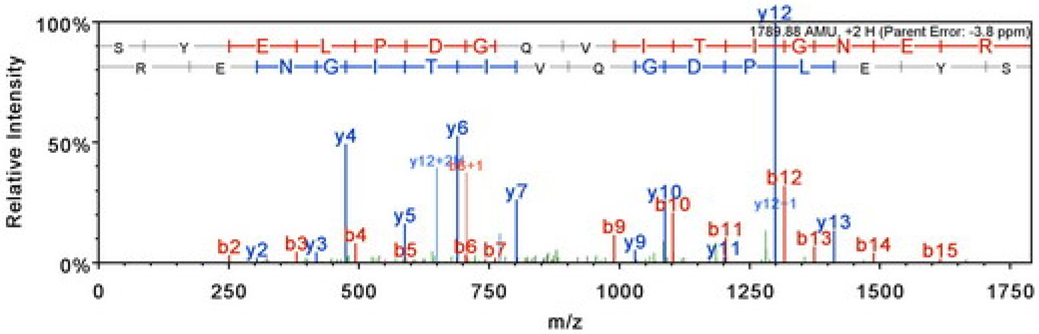

B	B Ions	B+2H	B-NH3	B-H2O	AA	Y Ions	Y+2H	Y-NH3	Y-H2O	Y
1	88.0			70.0	S	1790.9	896.0	1773.9	1772.9	16
2	251.1			233.1	Y	1703.9	852.4	1686.8	1685.9	15
3	380.2			362.1	E	1540.8	770.9	1523.8	1522.8	14
4	493.2			475.2	L	1411.8	706.4	1394.7	1393.7	13
5	590.3			572.3	P	1298.7	649.8	1281.6	1280.7	12
6	705.3	353.2		687.3	D	1201.6	601.3	1184.6	1183.6	11
7	762.3	381.7		744.3	G	1086.6	543.8	1069.6	1068.6	10
8	890.4	445.7	873.4	872.4	Q	1029.6	515.3	1012.5	1011.6	9
9	989.5	495.2	972.4	971.5	V	901.5	451.3	884.5	883.5	8
10	1102.5	551.8	1085.5	1084.5	I	802.4	401.7	785.4	784.4	7
11	1203.6	602.3	1186.6	1185.6	T	689.4	345.2	672.3	671.4	6
12	1316.7	658.8	1299.7	1298.7	I	588.3		571.3	570.3	5
13	1373.7	687.4	1356.7	1355.7	G	475.2		458.2	457.2	4
14	1487.7	744.4	1470.7	1469.7	N	418.2		401.2	400.2	3
15	1616.8	808.9	1599.8	1598.8	E	304.2		287.1	286.2	2
16	1790.9	896.0	1773.9	1772.9	R	175.1		158.1		1

Gene symbol: Actr2

Protein name: Actin-like protein 2

Protein accession numbers: IPI00177038,IPI00457901

Peptide sequence: (R)GYAFNHSADFETVR(M)

Exclusive (unique to this protein): TRUE

SEQUEST XCorr score: 2.11 SEQUEST DCn score: 0.44

Fix modifications: None

Variable modifications: None

charge: 2 Observed m/z: 807.3679

Actual minus calculated peptide mass (AMU): -0.0067152

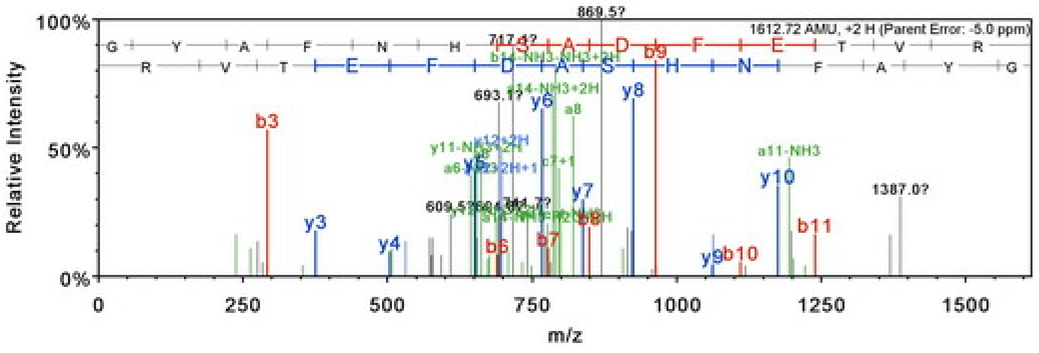

B	B Ions	B+2H	B-NH3	B-H2O	AA	Y Ions	Y+2H	Y-NH3	Y-H2O	Y
1	58.0				G	1613.7	807.4	1596.7	1595.7	14
2	221.1				Y	1556.7	778.9	1539.7	1538.7	13
3	292.1				A	1393.7	697.3	1376.6	1375.6	12
4	439.2				F	1322.6	661.8	1305.6	1304.6	11
5	553.2		536.2		N	1175.5	588.3	1158.5	1157.5	10
6	690.3	345.7	673.3		H	1061.5	531.3	1044.5	1043.5	9
7	777.3	389.2	760.3	759.3	S	924.4	462.7	907.4	906.4	8
8	848.4	424.7	831.3	830.4	A	837.4	419.2	820.4	819.4	7
9	963.4	482.2	946.4	945.4	D	766.4	383.7	749.4	748.4	6
10	1110.5	555.7	1093.4	1092.5	F	651.4		634.3	633.3	5
11	1239.5	620.3	1222.5	1221.5	E	504.3		487.3	486.3	4
12	1340.5	670.8	1323.5	1322.5	T	375.2		358.2	357.2	3
13	1439.6	720.3	1422.6	1421.6	V	274.2		257.2		2
14	1613.7	807.4	1596.7	1595.7	R	175.1		158.1		1

Gene symbol: Ada

Protein name: Adenosine deaminase

Protein accession numbers: IPI00261467

Peptide sequence: (R)VGHGYHTIEDEALYNR(L)

Exclusive (unique to this protein): TRUE

SEQUEST XCorr score: 2.25 SEQUEST DCn score: 0.583

Fix modifications: None

Variable modifications: None

charge: 2 Observed m/z: 937.4477

Actual minus calculated peptide mass (AMU): 0.004273

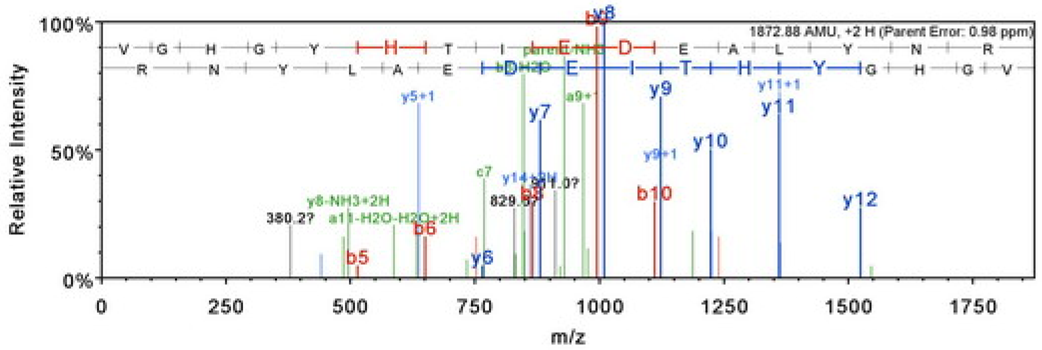

B	B Ions	B+2H	B-NH3	B-H2O	AA	Y Ions	Y+2H	Y-NH3	Y-H2O	Y
1	100.1				V	1873.9	937.5	1856.9	1855.9	16
2	157.1				G	1774.8	887.9	1757.8	1756.8	15
3	294.2	147.6			H	1717.8	859.4	1700.8	1699.8	14
4	351.2	176.1			G	1580.7	790.9	1563.7	1562.7	13
5	514.2	257.6			Y	1523.7	762.4	1506.7	1505.7	12
6	651.3	326.2			H	1360.7	680.8	1343.6	1342.6	11
7	752.4	376.7		734.3	T	1223.6	612.3	1206.6	1205.6	10
8	865.4	433.2		847.4	I	1122.5	561.8	1105.5	1104.5	9
9	994.5	497.7		976.5	E	1009.5	505.2	992.4	991.5	8
10	1109.5	555.3		1091.5	D	880.4	440.7	863.4	862.4	7
11	1238.5	619.8		1220.5	E	765.4	383.2	748.4	747.4	6
12	1309.6	655.3		1291.6	A	636.4		619.3		5
13	1422.7	711.8		1404.7	L	565.3		548.3		4
14	1585.7	793.4		1567.7	Y	452.2		435.2		3
15	1699.8	850.4	1682.8	1681.8	N	289.2		272.1		2
16	1873.9	937.5	1856.9	1855.9	R	175.1		158.1		1

Gene symbol: Adh5

Protein name: Alcohol dehydrogenase class 3

Protein accession numbers: IPI00555004

Peptide sequence: (K)AGDTVIPLYIPQCGECK(F)

Exclusive (unique to this protein): TRUE

SEQUEST XCorr score: 2.33   SEQUEST DCn score: 0.48

Fix modifications: C13: Carbamidomethyl (+57.02), C16: Carbamidomethyl (+57.02)Variable modifications: None

charge: 2   Observed m/z: 960.9618

Actual minus calculated peptide mass (AMU): -0.004395

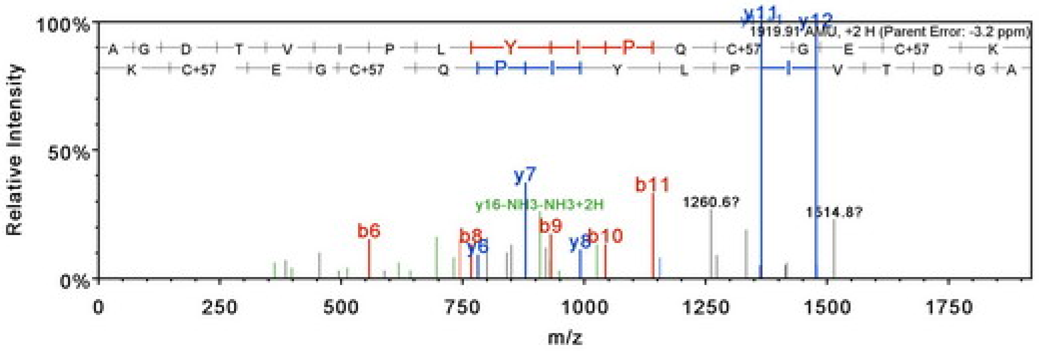

B	B Ions	B+2H	B-NH3	B-H2O	AA	Y Ions	Y+2H	Y-NH3	Y-H2O	Y
1	72.0				A	1920.9	961.0	1903.9	1902.9	17
2	129.1				G	1849.9	925.5	1832.9	1831.9	16
3	244.1			226.1	D	1792.9	896.9	1775.8	1774.9	15
4	345.1			327.1	T	1677.8	839.4	1660.8	1659.8	14
5	444.2			426.2	V	1576.8	788.9	1559.8	1558.8	13
6	557.3	279.2		539.3	I	1477.7	739.4	1460.7	1459.7	12
7	654.4	327.7		636.3	P	1364.6	682.8	1347.6	1346.6	11
8	767.4	384.2		749.4	L	1267.6	634.3	1250.6	1249.6	10
9	930.5	465.8		912.5	Y	1154.5	577.8	1137.5	1136.5	9
10	1043.6	522.3		1025.6	I	991.4	496.2	974.4	973.4	8
11	1140.6	570.8		1122.6	P	878.4	439.7	861.3	860.3	7
12	1268.7	634.9	1251.7	1250.7	Q	781.3	391.2	764.3	763.3	6
13	1428.7	714.9	1411.7	1410.7	C+57	653.2		636.2	635.2	5
14	1485.7	743.4	1468.7	1467.7	G	493.2		476.2	475.2	4
15	1614.8	807.9	1597.8	1596.8	E	436.2		419.2	418.2	3
16	1774.8	887.9	1757.8	1756.8	C+57	307.1		290.1		2
17	1920.9	961.0	1903.9	1902.9	K	147.1		130.1		1

Gene symbol: Adk

Protein name: Isoform Long of Adenosine kinase

Protein accession numbers: IPI00126940,IPI00138084

Peptide sequence: (R)SLVANLAAANCYK(K)

Exclusive (unique to this protein): TRUE

SEQUEST XCorr score: 3.11 SEQUEST DCn score: 0.725

Fix modifications: C11: Carbamidomethyl (+57.02)Variable modifications: None

charge: 2 Observed m/z: 697.8556

Actual minus calculated peptide mass (AMU): -0.006592

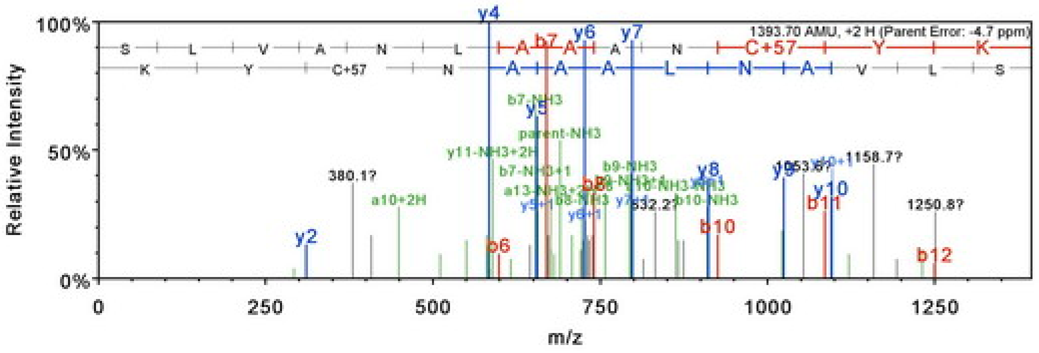

B	B Ions	B+2H	B-NH3	B-H2O	AA	Y Ions	Y+2H	Y-NH3	Y-H2O	Y
1	88.0			70.0	S	1394.7	697.9	1377.7	1376.7	13
2	201.1			183.1	L	1307.7	654.3	1290.7		12
3	300.2			282.2	V	1194.6	597.8	1177.6		11
4	371.2			353.2	A	1095.5	548.3	1078.5		10
5	485.3		468.3	467.3	N	1024.5	512.8	1007.5		9
6	598.4	299.7	581.3	580.4	L	910.5	455.7	893.4		8
7	669.4	335.2	652.4	651.4	A	797.4	399.2	780.3		7
8	740.4	370.7	723.4	722.4	A	726.3	363.7	709.3		6
9	811.5	406.2	794.4	793.5	A	655.3		638.3		5
10	925.5	463.3	908.5	907.5	N	584.3		567.2		4
11	1085.5	543.3	1068.5	1067.5	C+57	470.2		453.2		3
12	1248.6	624.8	1231.6	1230.6	Y	310.2		293.2		2
13	1394.7	697.9	1377.7	1376.7	K	147.1		130.1		1

Gene symbol: Adpgk

Protein name: Isoform 2 of ADP-dependent glucokinase

Protein accession numbers: IPI00556819

Peptide sequence: (R)LGPAPVPVGPLSPESR(L)

Exclusive (unique to this protein): TRUE

SEQUEST XCorr score: 2.09　　SEQUEST DCn score: 0.46

Fix modifications: None

Variable modifications: None

charge: 2　　Observed m/z: 786.938

Actual minus calculated peptide mass (AMU): -0.0070758

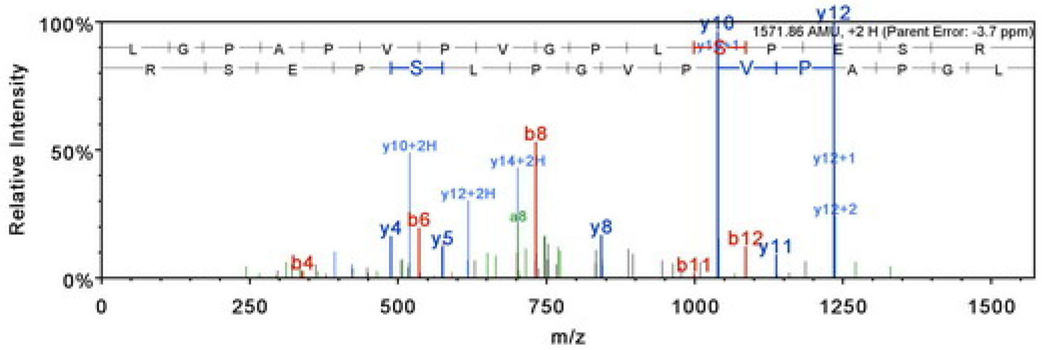

B	B Ions	B+2H	B-NH3	B-H2O	AA	Y Ions	Y+2H	Y-NH3	Y-H2O	Y
1	114.1				L	1572.9	786.9	1555.9	1554.9	16
2	171.1				G	1459.8	730.4	1442.8	1441.8	15
3	268.2				P	1402.8	701.9	1385.7	1384.8	14
4	339.2				A	1305.7	653.4	1288.7	1287.7	13
5	436.3				P	1234.7	617.8	1217.7	1216.7	12
6	535.3	268.2			V	1137.6	569.3	1120.6	1119.6	11
7	632.4	316.7			P	1038.6	519.8	1021.5	1020.6	10
8	731.5	366.2			V	941.5	471.3	924.5	923.5	9
9	788.5	394.7			G	842.4	421.7	825.4	824.4	8
10	885.5	443.3			P	785.4	393.2	768.4	767.4	7
11	998.6	499.8			L	688.4	344.7	671.3	670.4	6
12	1085.6	543.3		1067.6	S	575.3		558.3	557.3	5
13	1182.7	591.9		1164.7	P	488.3		471.2	470.2	4
14	1311.7	656.4		1293.7	E	391.2		374.2	373.2	3
15	1398.8	699.9		1380.8	S	262.1		245.1	244.1	2
16	1572.9	786.9	1555.9	1554.9	R	175.1		158.1		1

Gene symbol: Adsl

Protein name: Lung RCB-0558 LLC cDNA, RIKEN full-length enriched library, clone:G730029K06 product:adenylosuccinate lyase, full insert sequence

Protein accession numbers: IPI00265471,IPI00308217

Peptide sequence: (R)AFIITGQTYTR(K)

Exclusive (unique to this protein): TRUE

SEQUEST XCorr score: 2.85 SEQUEST DCn score: 0.637

Fix modifications: None

Variable modifications: None

charge: 2 Observed m/z: 635.8395

Actual minus calculated peptide mass (AMU): -0.0085453

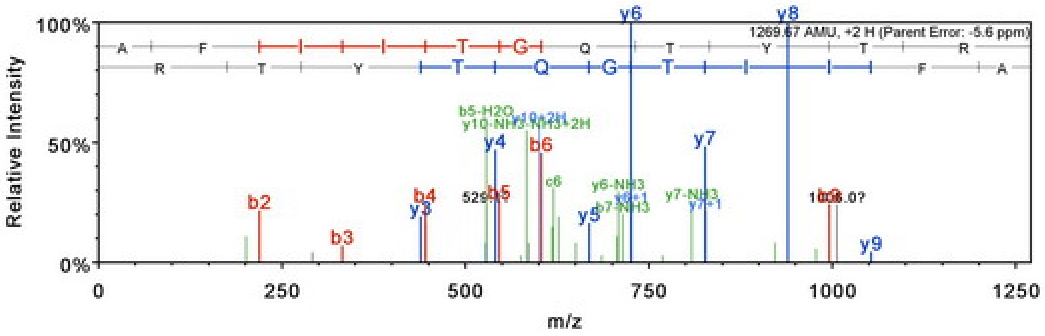

B	B Ions	B+2H	B-NH3	B-H2O	AA	Y Ions	Y+2H	Y-NH3	Y-H2O	Y
1	72.0				A	1270.7	635.8	1253.7	1252.7	11
2	219.1				F	1199.6	600.3	1182.6	1181.6	10
3	332.2				I	1052.6	526.8	1035.5	1034.6	9
4	445.3				I	939.5	470.3	922.5	921.5	8
5	546.3			528.3	T	826.4	413.7	809.4	808.4	7
6	603.4	302.2		585.3	G	725.4	363.2	708.3	707.4	6
7	731.4	366.2	714.4	713.4	Q	668.3		651.3	650.3	5
8	832.5	416.7	815.4	814.5	T	540.3		523.3	522.3	4
9	995.5	498.3	978.5	977.5	Y	439.2		422.2	421.2	3
10	1096.6	548.8	1079.5	1078.6	T	276.2		259.1	258.2	2
11	1270.7	635.8	1253.7	1252.7	R	175.1		158.1		1

Gene symbol: AI316787

Protein name: Uncharacterized protein C17orf32 homolog

Protein accession numbers: IPI00265298

Peptide sequence: (R)NVTCQDAQCGGTLSDLGK(Q)

Exclusive (unique to this protein): TRUE

SEQUEST XCorr score: 2.06 SEQUEST DCn score: 0.617

Fix modifications: C4: Carbamidomethyl (+57.02), C9: Carbamidomethyl (+57.02)Variable modifications: None

charge: 2 Observed m/z: 962.433

Actual minus calculated peptide mass (AMU): 0.003662

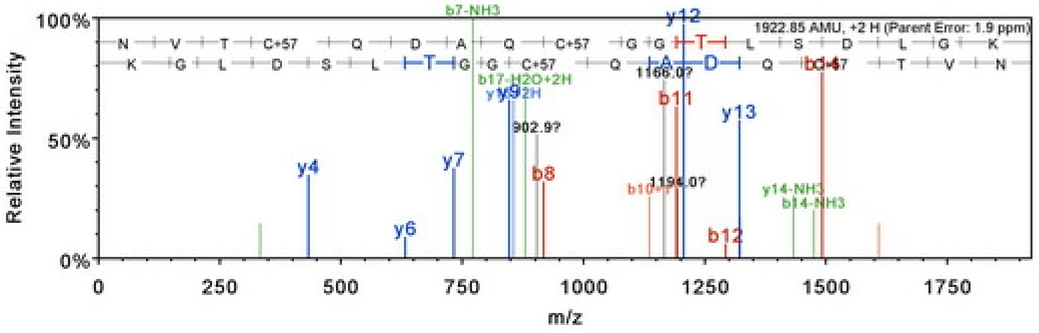

B	B Ions	B+2H	B-NH3	B-H2O	AA	Y Ions	Y+2H	Y-NH3	Y-H2O	Y
1	115.1		98.0		N	1923.9	962.4	1906.8	1905.8	18
2	214.1		197.1		V	1809.8	905.4	1792.8	1791.8	17
3	315.2		298.1	297.2	T	1710.7	855.9	1693.7	1692.7	16
4	475.2		458.2	457.2	C+57	1609.7	805.4	1592.7	1591.7	15
5	603.3		586.2	585.3	Q	1449.7	725.3	1432.6	1431.7	14
6	718.3	359.7	701.3	700.3	D	1321.6	661.3	1304.6	1303.6	13
7	789.3	395.2	772.3	771.3	A	1206.6	603.8	1189.5	1188.6	12
8	917.4	459.2	900.4	899.4	Q	1135.5	568.3	1118.5	1117.5	11
9	1077.4	539.2	1060.4	1059.4	C+57	1007.5	504.3	990.5	989.5	10
10	1134.4	567.7	1117.4	1116.4	G	847.5	424.2	830.4	829.4	9
11	1191.5	596.2	1174.4	1173.4	G	790.4	395.7	773.4	772.4	8
12	1292.5	646.8	1275.5	1274.5	T	733.4	367.2	716.4	715.4	7
13	1405.6	703.3	1388.6	1387.6	L	632.4	316.7	615.3	614.4	6
14	1492.6	746.8	1475.6	1474.6	S	519.3		502.3	501.3	5
15	1607.6	804.3	1590.6	1589.6	D	432.3		415.2	414.2	4
16	1720.7	860.9	1703.7	1702.7	L	317.2		300.2		3
17	1777.8	889.4	1760.7	1759.7	G	204.1		187.1		2
18	1923.9	962.4	1906.8	1905.8	K	147.1		130.1		1

Gene symbol: Amacr

Protein name: Alpha-methylacyl-CoA racemase

Protein accession numbers: IPI00114331,IPI00607025

Peptide sequence: (R)ASFITDGEQLPSPR(P)

Exclusive (unique to this protein): TRUE

SEQUEST XCorr score: 2.01 SEQUEST DCn score: 0.655

Fix modifications: None

Variable modifications: None

charge: 2 Observed m/z: 759.3793

Actual minus calculated peptide mass (AMU): -0.009522

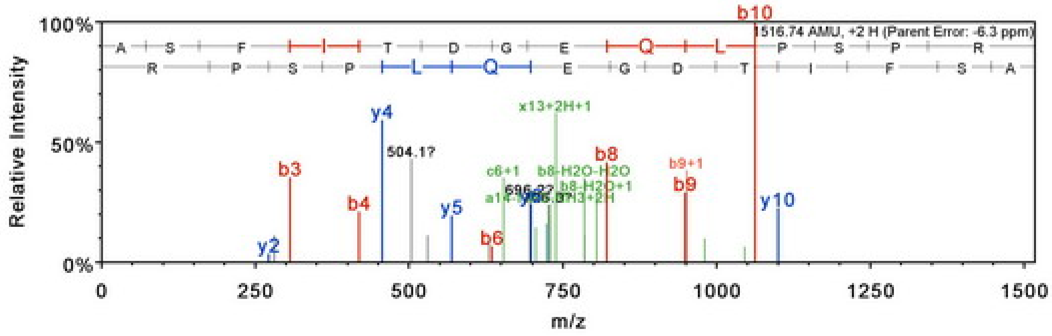

B	B Ions	B+2H	B-NH3	B-H2O	AA	Y Ions	Y+2H	Y-NH3	Y-H2O	Y
1	72.0				A	1517.8	759.4	1500.7	1499.8	14
2	159.1			141.1	S	1446.7	723.9	1429.7	1428.7	13
3	306.2			288.1	F	1359.7	680.4	1342.7	1341.7	12
4	419.2			401.2	I	1212.6	606.8	1195.6	1194.6	11
5	520.3			502.3	T	1099.5	550.3	1082.5	1081.5	10
6	635.3	318.2		617.3	D	998.5	499.8	981.5	980.5	9
7	692.3	346.7		674.3	G	883.5	442.2	866.4	865.5	8
8	821.4	411.2		803.4	E	826.4	413.7	809.4	808.4	7
9	949.4	475.2	932.4	931.4	Q	697.4	349.2	680.4	679.4	6
10	1062.5	531.8	1045.5	1044.5	L	569.3		552.3	551.3	5
11	1159.6	580.3	1142.5	1141.5	P	456.3		439.2	438.3	4
12	1246.6	623.8	1229.6	1228.6	S	359.2		342.2	341.2	3
13	1343.7	672.3	1326.6	1325.6	P	272.2		255.2		2
14	1517.8	759.4	1500.7	1499.8	R	175.1		158.1		1

Gene symbol: Ap2b1

Protein name: Isoform 1 of AP-2 complex subunit beta-1

Protein accession numbers: IPI00119689,IPI00378063

Peptide sequence: (R)LASQANIAQVLAELK(E)

Exclusive (unique to this protein): TRUE

SEQUEST XCorr score: 2.52 SEQUEST DCn score: 0.659

Fix modifications: None

Variable modifications: None

charge: 2 Observed m/z: 784.9516

Actual minus calculated peptide mass (AMU): -0.0059849

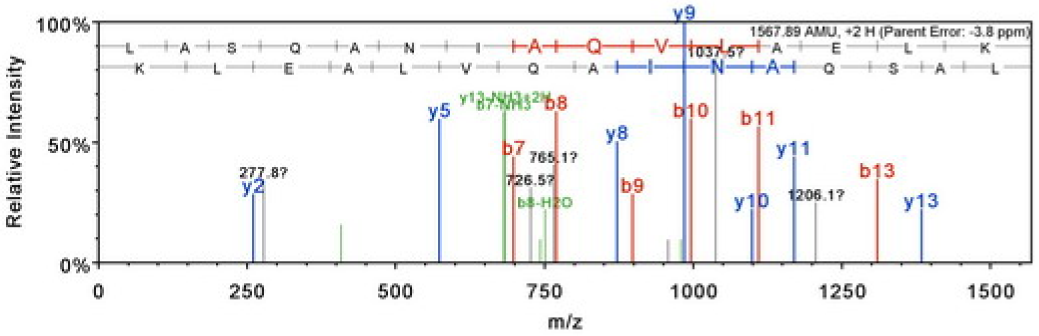

B	B Ions	B+2H	B-NH3	B-H2O	AA	Y Ions	Y+2H	Y-NH3	Y-H2O	Y
1	114.1				L	1568.9	785.0	1551.9	1550.9	15
2	185.1				A	1455.8	728.4	1438.8	1437.8	14
3	272.2			254.2	S	1384.8	692.9	1367.8	1366.8	13
4	400.2		383.2	382.2	Q	1297.8	649.4	1280.7	1279.7	12
5	471.3		454.2	453.3	A	1169.7	585.4	1152.7	1151.7	11
6	585.3	293.2	568.3	567.3	N	1098.7	549.8	1081.6	1080.6	10
7	698.4	349.7	681.4	680.4	I	984.6	492.8	967.6	966.6	9
8	769.4	385.2	752.4	751.4	A	871.5	436.3	854.5	853.5	8
9	897.5	449.2	880.5	879.5	Q	800.5	400.8	783.5	782.5	7
10	996.6	498.8	979.5	978.5	V	672.4	336.7	655.4	654.4	6
11	1109.6	555.3	1092.6	1091.6	L	573.4		556.3	555.4	5
12	1180.7	590.8	1163.6	1162.7	A	460.3		443.3	442.3	4
13	1309.7	655.4	1292.7	1291.7	E	389.2		372.2	371.2	3
14	1422.8	711.9	1405.8	1404.8	L	260.2		243.2		2
15	1568.9	785.0	1551.9	1550.9	K	147.1		130.1		1

Gene symbol: Apcs

Protein name: Serum amyloid P-component precursor

Protein accession numbers: IPI00309214

Peptide sequence: (K)APPSIVLGQEQDNYGGGFQR(S)

Exclusive (unique to this protein): TRUE

SEQUEST XCorr score: 2.11 SEQUEST DCn score: 0.636

Fix modifications: None

Variable modifications: None

charge: 2 Observed m/z: 1067.02

Actual minus calculated peptide mass (AMU): -0.00415

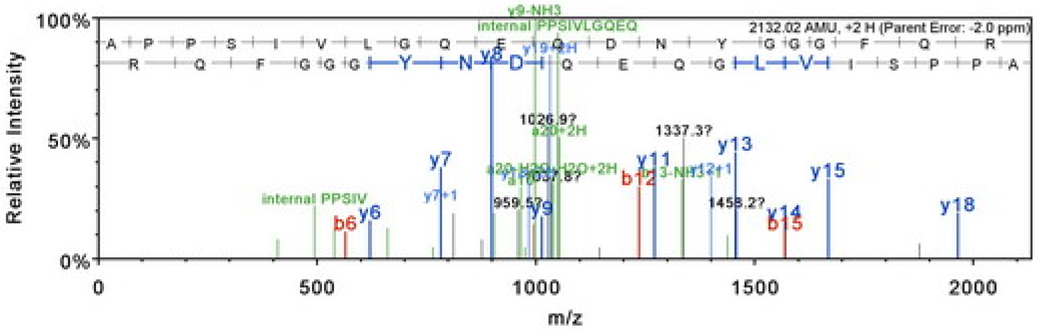

B	B Ions	B+2H	B-NH3	B-H2O	AA	Y Ions	Y+2H	Y-NH3	Y-H2O	Y
1	72.0				A	2133.0	1067.0	2116.0	2115.0	20
2	169.1				P	2062.0	1031.5	2045.0	2044.0	19
3	266.1				P	1965.0	983.0	1947.9	1946.9	18
4	353.2			335.2	S	1867.9	934.5	1850.9	1849.9	17
5	466.3			448.3	I	1780.9	890.9	1763.8	1762.9	16
6	565.3	283.2		547.3	V	1667.8	834.4	1650.8	1649.8	15
7	678.4	339.7		660.4	L	1568.7	784.9	1551.7	1550.7	14
8	735.4	368.2		717.4	G	1455.6	728.3	1438.6	1437.6	13
9	863.5	432.3	846.5	845.5	Q	1398.6	699.8	1381.6	1380.6	12
10	992.5	496.8	975.5	974.5	E	1270.5	635.8	1253.5	1252.5	11
11	1120.6	560.8	1103.6	1102.6	Q	1141.5	571.3	1124.5	1123.5	10
12	1235.6	618.3	1218.6	1217.6	D	1013.4	507.2	996.4	995.4	9
13	1349.7	675.3	1332.6	1331.7	N	898.4	449.7	881.4		8
14	1512.7	756.9	1495.7	1494.7	Y	784.4	392.7	767.4		7
15	1569.8	785.4	1552.7	1551.7	G	621.3	311.2	604.3		6
16	1626.8	813.9	1609.8	1608.8	G	564.3		547.3		5
17	1683.8	842.4	1666.8	1665.8	G	507.3		490.2		4
18	1830.9	915.9	1813.8	1812.9	F	450.3		433.2		3
19	1958.9	980.0	1941.9	1940.9	Q	303.2		286.2		2
20	2133.0	1067.0	2116.0	2115.0	R	175.1		158.1		1

Gene symbol: Apeh

Protein name: Isoform 2 of Acylamino-acid-releasing enzyme

Protein accession numbers: IPI00279218,IPI00387245

Peptide sequence: (R)QVLLSEPQEAAALYR(G)

Exclusive (unique to this protein): TRUE

SEQUEST XCorr score: 2.61 SEQUEST DCn score: 0.603

Fix modifications: None

Variable modifications: None

charge: 2 Observed m/z: 844.4522

Actual minus calculated peptide mass (AMU): -0.005493

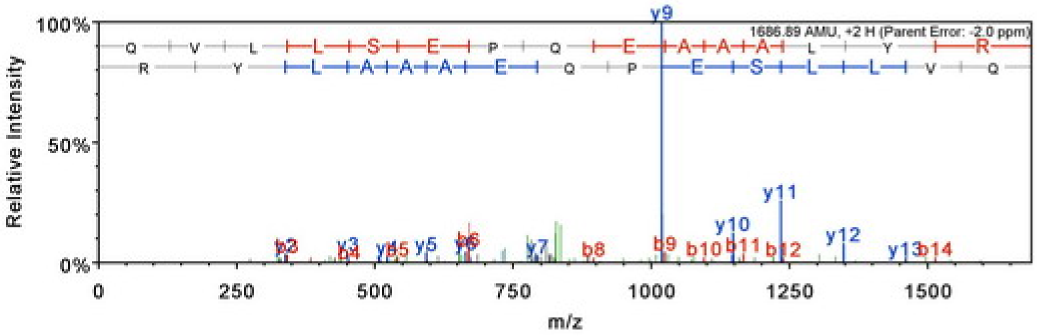

B	B Ions	B+2H	B-NH3	B-H2O	AA	Y Ions	Y+2H	Y-NH3	Y-H2O	Y
1	129.1		112.0		Q	1687.9	844.5	1670.9	1669.9	15
2	228.1		211.1		V	1559.8	780.4	1542.8	1541.8	14
3	341.2		324.2		L	1460.8	730.9	1443.8	1442.8	13
4	454.3		437.3		L	1347.7	674.4	1330.7	1329.7	12
5	541.3		524.3	523.3	S	1234.6	617.8	1217.6	1216.6	11
6	670.4	335.7	653.4	652.4	E	1147.6	574.3	1130.5	1129.6	10
7	767.4	384.2	750.4	749.4	P	1018.5	509.8	1001.5	1000.5	9
8	895.5	448.3	878.5	877.5	Q	921.5	461.2	904.5	903.5	8
9	1024.5	512.8	1007.5	1006.5	E	793.4	397.2	776.4	775.4	7
10	1095.6	548.3	1078.5	1077.6	A	664.4	332.7	647.4		6
11	1166.6	583.8	1149.6	1148.6	A	593.3		576.3		5
12	1237.6	619.3	1220.6	1219.6	A	522.3		505.3		4
13	1350.7	675.9	1333.7	1332.7	L	451.3		434.2		3
14	1513.8	757.4	1496.8	1495.8	Y	338.2		321.2		2
15	1687.9	844.5	1670.9	1669.9	R	175.1		158.1		1

Gene symbol: Apoa1bp

Protein name: Apolipoprotein A-I-binding protein precursor

Protein accession numbers: IPI00170307

Peptide sequence: (K)YQLNLPSYPDTECVYR(L)

Exclusive (unique to this protein): TRUE

SEQUEST XCorr score: 2.02 SEQUEST DCn score: 0.427

Fix modifications: C13: Carbamidomethyl (+57.02)Variable modifications: None

charge: 2 Observed m/z: 1009.4675

Actual minus calculated peptide mass (AMU): -0.005737

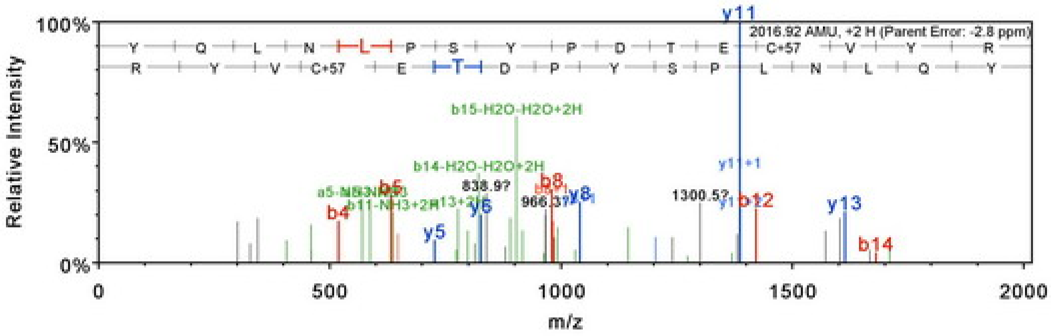

B	B Ions	B+2H	B-NH3	B-H2O	AA	Y Ions	Y+2H	Y-NH3	Y-H2O	Y
1	164.1				Y	2017.9	1009.5	2000.9	1999.9	16
2	292.1		275.1		Q	1854.9	927.9	1837.8	1836.9	15
3	405.2		388.2		L	1726.8	863.9	1709.8	1708.8	14
4	519.3		502.2		N	1613.7	807.4	1596.7	1595.7	13
5	632.3		615.3		L	1499.7	750.4	1482.7	1481.7	12
6	729.4	365.2	712.4		P	1386.6	693.8	1369.6	1368.6	11
7	816.4	408.7	799.4	798.4	S	1289.5	645.3	1272.5	1271.5	10
8	979.5	490.3	962.5	961.5	Y	1202.5	601.8	1185.5	1184.5	9
9	1076.5	538.8	1059.5	1058.5	P	1039.5	520.2	1022.4	1021.4	8
10	1191.6	596.3	1174.5	1173.6	D	942.4	471.7	925.4	924.4	7
11	1292.6	646.8	1275.6	1274.6	T	827.4	414.2	810.4	809.4	6
12	1421.7	711.3	1404.6	1403.7	E	726.3		709.3	708.3	5
13	1581.7	791.4	1564.7	1563.7	C+57	597.3		580.3		4
14	1680.8	840.9	1663.7	1662.8	V	437.3		420.2		3
15	1843.8	922.4	1826.8	1825.8	Y	338.2		321.2		2
16	2017.9	1009.5	2000.9	1999.9	R	175.1		158.1		1

Gene symbol: Arf5

Protein name: ADP-ribosylation factor 5

Protein accession numbers: IPI00221615

Peptide sequence: (K)NICFTVWDVGGQDK(I)

Exclusive (unique to this protein): TRUE

SEQUEST XCorr score: 2.02 SEQUEST DCn score: 0.625

Fix modifications: C3: Carbamidomethyl (+57.02)Variable modifications: None

charge: 2 Observed m/z: 819.884

Actual minus calculated peptide mass (AMU): 0.001343

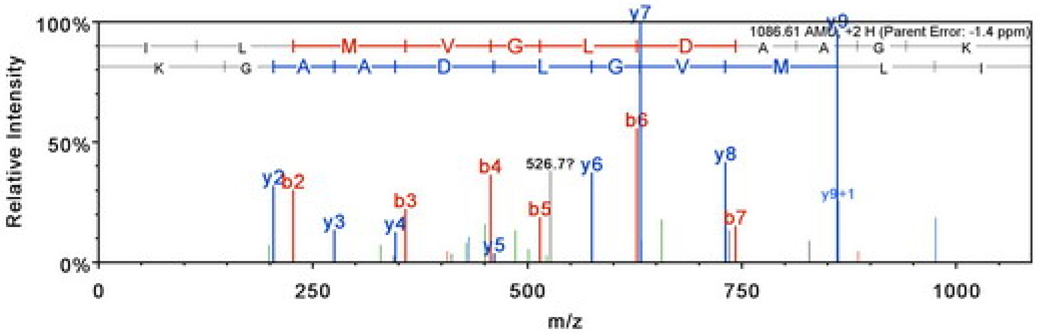

B	B Ions	B+2H	B-NH3	B-H2O	AA	Y Ions	Y+2H	Y-NH3	Y-H2O	Y
1	114.1				I	1087.6	544.3	1070.6	1069.6	11
2	227.2				L	974.5	487.8	957.5	956.5	10
3	358.2				M	861.5	431.2	844.4	843.4	9
4	457.3				V	730.4	365.7	713.4	712.4	8
5	514.3				G	631.3	316.2	614.3	613.3	7
6	627.4	314.2			L	574.3	287.7	557.3	556.3	6
7	742.4	371.7		724.4	D	461.2		444.2	443.2	5
8	813.5	407.2		795.4	A	346.2		329.2		4
9	884.5	442.8		866.5	A	275.2		258.1		3
10	941.5	471.3		923.5	G	204.1		187.1		2
11	1087.6	544.3	1070.6	1069.6	K	147.1		130.1		1

Gene symbol: Arl1
Protein name: Arl1 protein
Protein accession numbers: IPI00608124
Peptide sequence: (R)ILILGLDGAGK(T)
Exclusive (unique to this protein): TRUE
SEQUEST XCorr score: 2.12 SEQUEST DCn score: 0.477
Fix modifications: None
Variable modifications: None
charge: 2 Observed m/z: 535.3309
Actual minus calculated peptide mass (AMU): -0.0085453

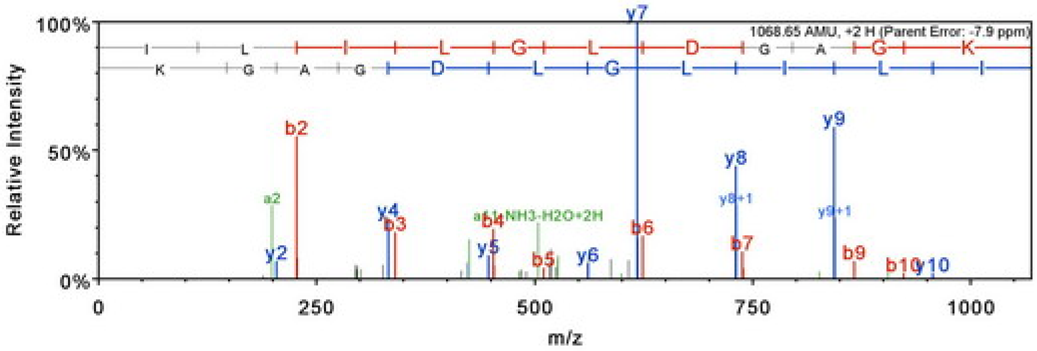

B	B Ions	B+2H	B-NH3	B-H2O	AA	Y Ions	Y+2H	Y-NH3	Y-H2O	Y
1	114.1				I	1069.7	535.3	1052.6	1051.7	11
2	227.2				L	956.6	478.8	939.6	938.6	10
3	340.3				I	843.5	422.3	826.5	825.5	9
4	453.3				L	730.4	365.7	713.4	712.4	8
5	510.4				G	617.3	309.2	600.3	599.3	7
6	623.5	312.2			L	560.3	280.7	543.3	542.3	6
7	738.5	369.7		720.5	D	447.2		430.2	429.2	5
8	795.5	398.3		777.5	G	332.2		315.2		4
9	866.5	433.8		848.5	A	275.2		258.1		3
10	923.6	462.3		905.6	G	204.1		187.1		2
11	1069.7	535.3	1052.6	1051.7	K	147.1		130.1		1

Gene symbol: Arl3
Protein name: ADP-ribosylation factor-like protein 3
Protein accession numbers: IPI00124787
Peptide sequence: (R)ILLLGLDNAGK(T)
Exclusive (unique to this protein): TRUE
SEQUEST XCorr score: 2.07 SEQUEST DCn score: 0.436
Fix modifications: None
Variable modifications: None
charge: 2 Observed m/z: 563.8414
Actual minus calculated peptide mass (AMU): -0.0089149

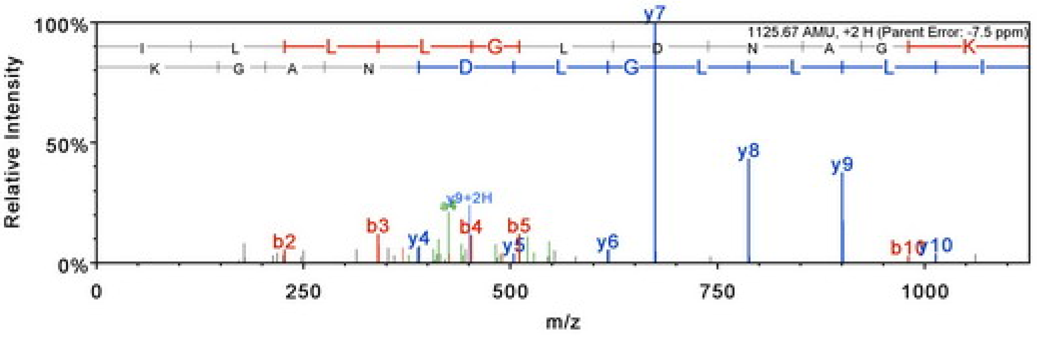

B	B Ions	B+2H	B-NH3	B-H2O	AA	Y Ions	Y+2H	Y-NH3	Y-H2O	Y
1	114.1				I	1126.7	563.9	1109.7	1108.7	11
2	227.2				L	1013.6	507.3	996.6	995.6	10
3	340.3				L	900.5	450.8	883.5	882.5	9
4	453.3				L	787.4	394.2	770.4	769.4	8
5	510.4				G	674.4	337.7	657.3	656.3	7
6	623.5	312.2			L	617.3	309.2	600.3	599.3	6
7	738.5	369.7		720.5	D	504.2		487.2	486.2	5
8	852.5	426.8	835.5	834.5	N	389.2		372.2		4
9	923.6	462.3	906.5	905.6	A	275.2		258.1		3
10	980.6	490.8	963.6	962.6	G	204.1		187.1		2
11	1126.7	563.9	1109.7	1108.7	K	147.1		130.1		1

Gene symbol: Armet

Protein name: ARMET protein precursor

Protein accession numbers: IPI00110350,IPI00404019,IPI00775791

Peptide sequence: (R)LCYYIGATDDAATK(I)

Exclusive (unique to this protein): TRUE

SEQUEST XCorr score: 2.4　　SEQUEST DCn score: 0.652

Fix modifications: C2: Carbamidomethyl (+57.02)Variable modifications: None

charge: 2　　Observed m/z: 781.3652

Actual minus calculated peptide mass (AMU): 0.0015855

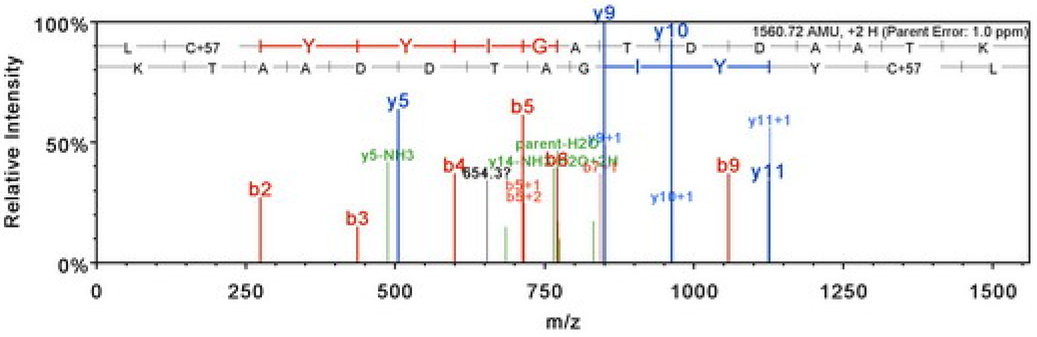

B	B Ions	B+2H	B-NH3	B-H2O	AA	Y Ions	Y+2H	Y-NH3	Y-H2O	Y
1	114.1				L	1561.7	781.4	1544.7	1543.7	14
2	274.1				C+57	1448.6	724.8	1431.6	1430.6	13
3	437.2				Y	1288.6	644.8	1271.6	1270.6	12
4	600.3				Y	1125.5	563.3	1108.5	1107.5	11
5	713.3				I	962.5	481.7	945.5	944.5	10
6	770.4	385.7			G	849.4	425.2	832.4	831.4	9
7	841.4	421.2			A	792.4	396.7	775.4	774.4	8
8	942.4	471.7		924.4	T	721.3	361.2	704.3	703.3	7
9	1057.5	529.2		1039.5	D	620.3	310.7	603.3	602.3	6
10	1172.5	586.8		1154.5	D	505.3		488.2	487.3	5
11	1243.5	622.3		1225.5	A	390.2		373.2	372.2	4
12	1314.6	657.8		1296.6	A	319.2		302.2	301.2	3
13	1415.6	708.3		1397.6	T	248.2		231.1	230.2	2
14	1561.7	781.4	1544.7	1543.7	K	147.1		130.1		1

Gene symbol: Asna1

Protein name: Arsenical pump-driving ATPase

Protein accession numbers: IPI00624501

Peptide sequence: (R)ESVLIISTDPAHNISDAFDQK(F)

Exclusive (unique to this protein): TRUE

SEQUEST XCorr score: 2.35 SEQUEST DCn score: 0.636

Fix modifications: None

Variable modifications: None

charge: 2 Observed m/z: 1150.5696

Actual minus calculated peptide mass (AMU): -0.009766

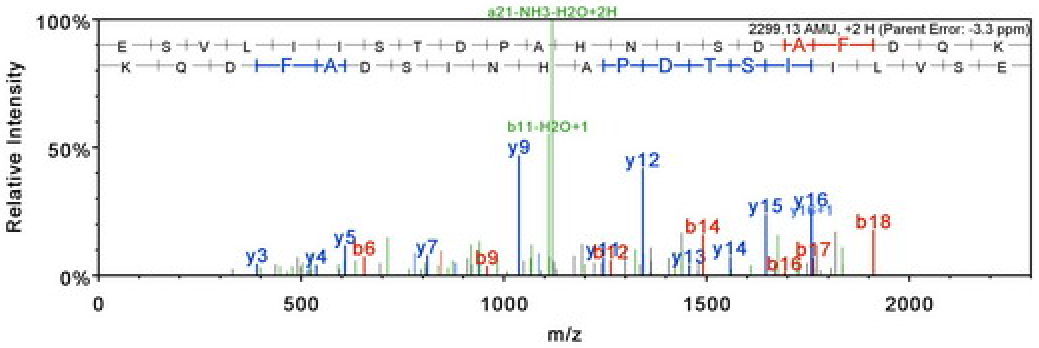

B	B Ions	B+2H	B-NH3	B-H2O	AA	Y Ions	Y+2H	Y-NH3	Y-H2O	Y
1	130.1			112.0	E	2300.1	1150.6	2283.1	2282.1	21
2	217.1			199.1	S	2171.1	1086.0	2154.1	2153.1	20
3	316.2			298.1	V	2084.1	1042.5	2067.0	2066.1	19
4	429.2			411.2	L	1985.0	993.0	1968.0	1967.0	18
5	542.3			524.3	I	1871.9	936.5	1854.9	1853.9	17
6	655.4	328.2		637.4	I	1758.8	879.9	1741.8	1740.8	16
7	742.4	371.7		724.4	S	1645.8	823.4	1628.7	1627.7	15
8	843.5	422.3		825.5	T	1558.7	779.9	1541.7	1540.7	14
9	958.5	479.8		940.5	D	1457.7	729.3	1440.6	1439.7	13
10	1055.6	528.3		1037.5	P	1342.6	671.8	1325.6	1324.6	12
11	1126.6	563.8		1108.6	A	1245.6	623.3	1228.6	1227.6	11
12	1263.7	632.3		1245.7	H	1174.5	587.8	1157.5	1156.5	10
13	1377.7	689.4	1360.7	1359.7	N	1037.5	519.3	1020.5	1019.5	9
14	1490.8	745.9	1473.8	1472.8	I	923.5	462.2	906.4	905.4	8
15	1577.8	789.4	1560.8	1559.8	S	810.4	405.7	793.3	792.4	7
16	1692.8	846.9	1675.8	1674.8	D	723.3	362.2	706.3	705.3	6
17	1763.9	882.4	1746.9	1745.9	A	608.3		591.3	590.3	5
18	1911.0	956.0	1893.9	1892.9	F	537.3		520.2	519.3	4
19	2026.0	1013.5	2009.0	2008.0	D	390.2		373.2	372.2	3
20	2154.0	1077.5	2137.0	2136.0	Q	275.2		258.1		2
21	2300.1	1150.6	2283.1	2282.1	K	147.1		130.1		1

Gene symbol: Atg5

Protein name: Autophagy protein 5

Protein accession numbers: IPI00114936

Peptide sequence: (R)IPTCFTLYQDEITER(E)

Exclusive (unique to this protein): TRUE

SEQUEST XCorr score: 2.5 SEQUEST DCn score: 0.648

Fix modifications: C4: Carbamidomethyl (+57.02)Variable modifications: None

charge: 2 Observed m/z: 943.4519

Actual minus calculated peptide mass (AMU): -0.004883

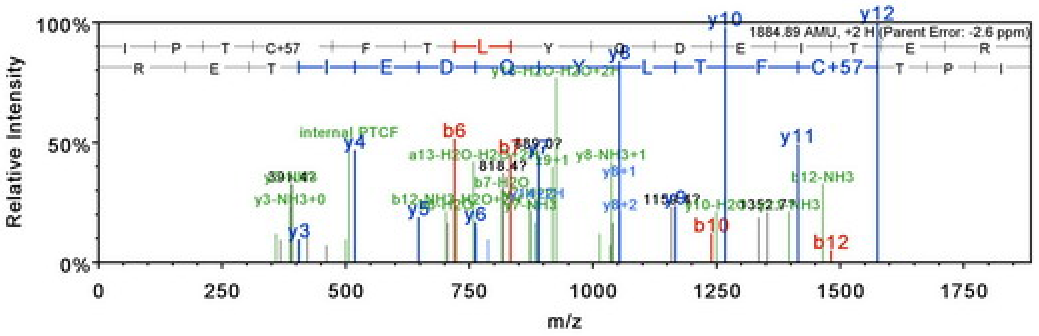

B	B Ions	B+2H	B-NH3	B-H2O	AA	Y Ions	Y+2H	Y-NH3	Y-H2O	Y
1	114.1				I	1885.9	943.5	1868.9	1867.9	15
2	211.1				P	1772.8	886.9	1755.8	1754.8	14
3	312.2			294.2	T	1675.8	838.4	1658.7	1657.8	13
4	472.2			454.2	C+57	1574.7	787.9	1557.7	1556.7	12
5	619.3			601.3	F	1414.7	707.9	1397.7	1396.7	11
6	720.3	360.7		702.3	T	1267.6	634.3	1250.6	1249.6	10
7	833.4	417.2		815.4	L	1166.6	583.8	1149.5	1148.6	9
8	996.5	498.8		978.5	Y	1053.5	527.3	1036.5	1035.5	8
9	1124.5	562.8	1107.5	1106.5	Q	890.4	445.7	873.4	872.4	7
10	1239.6	620.3	1222.5	1221.6	D	762.4	381.7	745.3	744.4	6
11	1368.6	684.8	1351.6	1350.6	E	647.3		630.3	629.3	5
12	1481.7	741.4	1464.7	1463.7	I	518.3		501.3	500.3	4
13	1582.8	791.9	1565.7	1564.7	T	405.2		388.2	387.2	3
14	1711.8	856.4	1694.8	1693.8	E	304.2		287.1	286.2	2
15	1885.9	943.5	1868.9	1867.9	R	175.1		158.1		1

Gene symbol: Atp1a1

Protein name: Sodium/potassium-transporting ATPase subunit alpha-1 precursor

Protein accession numbers: IPI00311682

Peptide sequence: (K)GVGIISEGNETVEDIAAR(L)

Exclusive (unique to this protein): TRUE

SEQUEST XCorr score: 2.65 SEQUEST DCn score: 0.64

Fix modifications: None

Variable modifications: None

charge: 2 Observed m/z: 915.4631

Actual minus calculated peptide mass (AMU): -0.006226

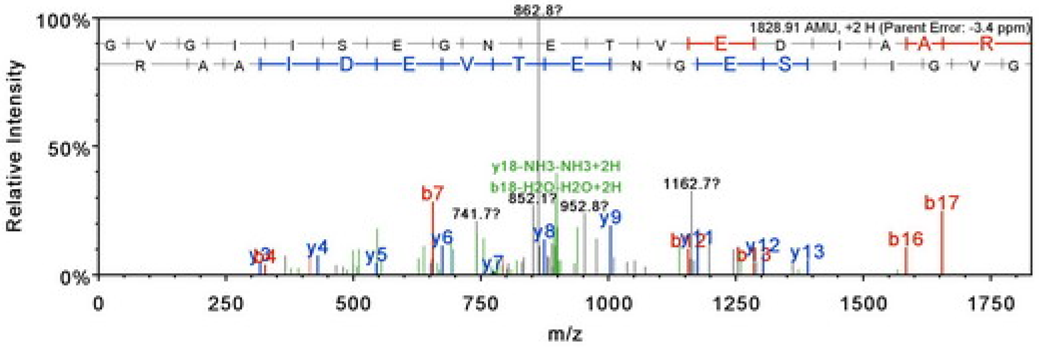

B	B Ions	B+2H	B-NH3	B-H2O	AA	Y Ions	Y+2H	Y-NH3	Y-H2O	Y
1	58.0				G	1829.9	915.5	1812.9	1811.9	18
2	157.1				V	1772.9	887.0	1755.9	1754.9	17
3	214.1				G	1673.8	837.4	1656.8	1655.8	16
4	327.2				I	1616.8	808.9	1599.8	1598.8	15
5	440.3				I	1503.7	752.4	1486.7	1485.7	14
6	527.3	264.2		509.3	S	1390.7	695.8	1373.6	1372.6	13
7	656.4	328.7		638.4	E	1303.6	652.3	1286.6	1285.6	12
8	713.4	357.2		695.4	G	1174.6	587.8	1157.5	1156.6	11
9	827.4	414.2	810.4	809.4	N	1117.5	559.3	1100.5	1099.5	10
10	956.5	478.7	939.4	938.5	E	1003.5	502.3	986.5	985.5	9
11	1057.5	529.3	1040.5	1039.5	T	874.5	437.7	857.4	856.5	8
12	1156.6	578.8	1139.6	1138.6	V	773.4	387.2	756.4	755.4	7
13	1285.6	643.3	1268.6	1267.6	E	674.4	337.7	657.3	656.3	6
14	1400.7	700.8	1383.6	1382.6	D	545.3		528.3	527.3	5
15	1513.7	757.4	1496.7	1495.7	I	430.3		413.3		4
16	1584.8	792.9	1567.8	1566.8	A	317.2		300.2		3
17	1655.8	828.4	1638.8	1637.8	A	246.2		229.1		2
18	1829.9	915.5	1812.9	1811.9	R	175.1		158.1		1

Gene symbol: Atp5f1

Protein name: ATP synthase B chain, mitochondrial precursor

Protein accession numbers: IPI00341282,IPI00758392

Peptide sequence: (R)HYLFDVQR(N)

Exclusive (unique to this protein): TRUE

SEQUEST XCorr score: 2.38 SEQUEST DCn score: 0.596

Fix modifications: None

Variable modifications: None

charge: 2 Observed m/z: 539.2771

Actual minus calculated peptide mass (AMU): -0.001831

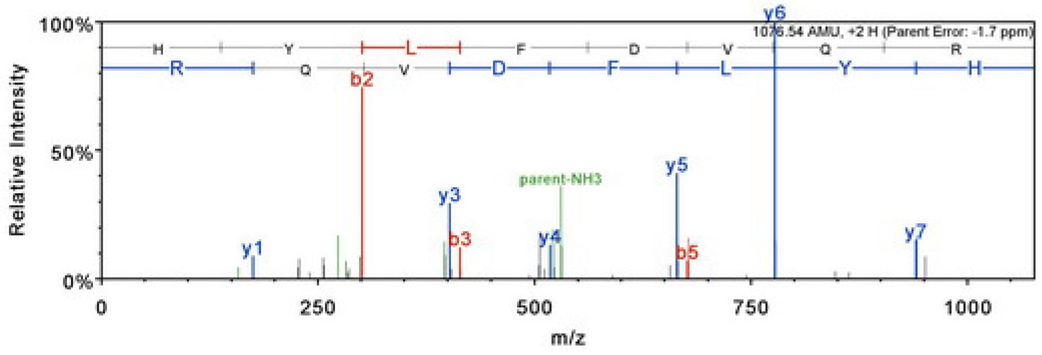

B	B Ions	B+2H	B-NH3	B-H2O	AA	Y Ions	Y+2H	Y-NH3	Y-H2O	Y
1	138.1	69.5			H	1077.5	539.3	1060.5	1059.5	8
2	301.1	151.1			Y	940.5	470.8	923.5	922.5	7
3	414.2	207.6			L	777.4	389.2	760.4	759.4	6
4	561.3	281.2			F	664.3		647.3	646.3	5
5	676.3	338.7		658.3	D	517.3		500.3	499.3	4
6	775.4	388.2		757.4	V	402.3		385.2		3
7	903.4	452.2	886.4	885.4	Q	303.2		286.2		2
8	1077.5	539.3	1060.5	1059.5	R	175.1		158.1		1

Gene symbol: Atp6v1c1
Protein name: Vacuolar ATP synthase subunit C 1
Protein accession numbers: IPI00130186
Peptide sequence: (R)SSNVLSEDQDSYLCNVTLFR(K)
Exclusive (unique to this protein): TRUE
SEQUEST XCorr score: 2.43   SEQUEST DCn score: 0.656
Fix modifications: C14: Carbamidomethyl (+57.02)Variable modifications: None
charge: 2   Observed m/z: 1174.044
Actual minus calculated peptide mass (AMU): -0.007324

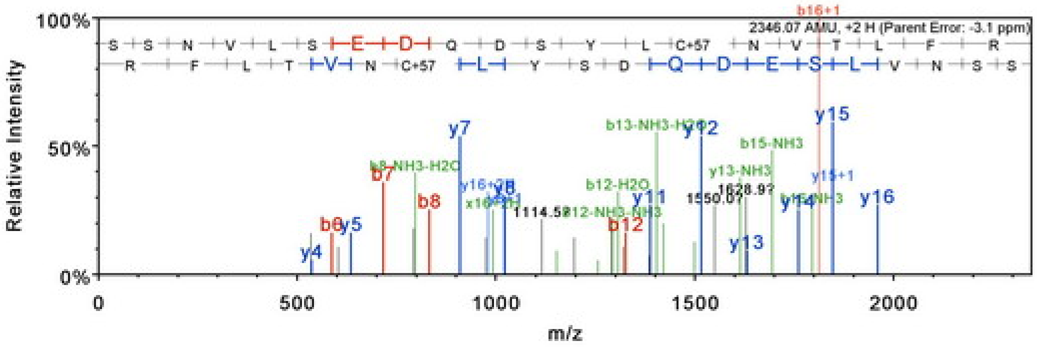

B	B Ions	B+2H	B-NH3	B-H2O	AA	Y Ions	Y+2H	Y-NH3	Y-H2O	Y
1	88.0			70.0	S	2347.1	1174.0	2330.1	2329.1	20
2	175.1			157.1	S	2260.1	1130.5	2243.0	2242.1	19
3	289.1		272.1	271.1	N	2173.0	1087.0	2156.0	2155.0	18
4	388.2		371.2	370.2	V	2059.0	1030.0	2042.0	2041.0	17
5	501.3		484.2	483.3	L	1959.9	980.5	1942.9	1941.9	16
6	588.3	294.7	571.3	570.3	S	1846.8	923.9	1829.8	1828.8	15
7	717.3	359.2	700.3	699.3	E	1759.8	880.4	1742.8	1741.8	14
8	832.4	416.7	815.3	814.4	D	1630.8	815.9	1613.7	1612.7	13
9	960.4	480.7	943.4	942.4	Q	1515.7	758.4	1498.7	1497.7	12
10	1075.5	538.2	1058.4	1057.4	D	1387.7	694.3	1370.6	1369.7	11
11	1162.5	581.8	1145.5	1144.5	S	1272.6	636.8	1255.6	1254.6	10
12	1325.5	663.3	1308.5	1307.5	Y	1185.6	593.3	1168.6	1167.6	9
13	1438.6	719.8	1421.6	1420.6	L	1022.6	511.8	1005.5	1004.5	8
14	1598.7	799.8	1581.6	1580.7	C+57	909.5	455.2	892.4	891.5	7
15	1712.7	856.9	1695.7	1694.7	N	749.4	375.2	732.4	731.4	6
16	1811.8	906.4	1794.8	1793.8	V	635.4		618.4	617.4	5
17	1912.8	956.9	1895.8	1894.8	T	536.3		519.3	518.3	4
18	2025.9	1013.5	2008.9	2007.9	L	435.3		418.3		3
19	2173.0	1087.0	2155.9	2155.0	F	322.2		305.2		2
20	2347.1	1174.0	2330.1	2329.1	R	175.1		158.1		1

Gene symbol: Atp6v1d
Protein name: Vacuolar ATP synthase subunit D
Protein accession numbers: IPI00118787
Peptide sequence: (K)FTAGDFSTTVIQNVNK(A)

Exclusive (unique to this protein): TRUE
SEQUEST XCorr score: 2.36 SEQUEST DCn score: 0.201
Fix modifications: None
Variable modifications: None
charge: 2 Observed m/z: 871.445
Actual minus calculated peptide mass (AMU): 0.006104

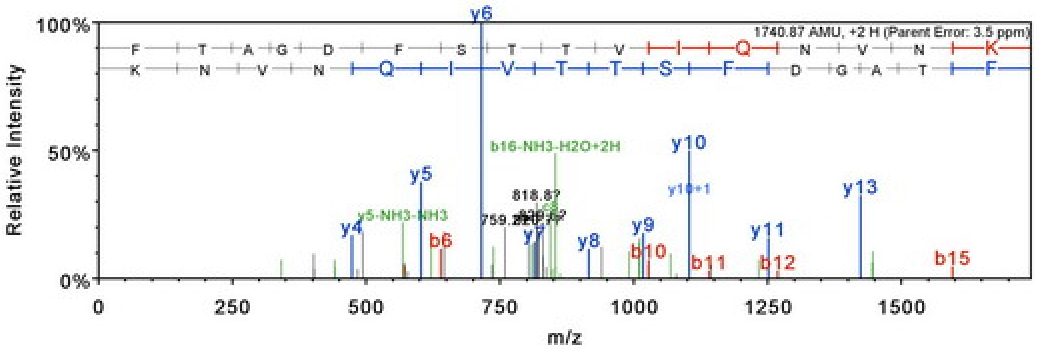

B	B Ions	B+2H	B-NH3	B-H2O	AA	Y Ions	Y+2H	Y-NH3	Y-H2O	Y
1	148.1				F	1741.9	871.4	1724.9	1723.9	16
2	249.1			231.1	T	1594.8	797.9	1577.8	1576.8	15
3	320.2			302.2	A	1493.8	747.4	1476.7	1475.8	14
4	377.2			359.2	G	1422.7	711.9	1405.7	1404.7	13
5	492.2			474.2	D	1365.7	683.4	1348.7	1347.7	12
6	639.3	320.1		621.3	F	1250.7	625.8	1233.7	1232.7	11
7	726.3	363.7		708.3	S	1103.6	552.3	1086.6	1085.6	10
8	827.4	414.2		809.4	T	1016.6	508.8	999.6	998.6	9
9	928.4	464.7		910.4	T	915.5	458.3	898.5	897.5	8
10	1027.5	514.2		1009.5	V	814.5	407.7	797.5		7
11	1140.6	570.8		1122.5	I	715.4	358.2	698.4		6
12	1268.6	634.8	1251.6	1250.6	Q	602.3		585.3		5
13	1382.7	691.8	1365.6	1364.7	N	474.3		457.2		4
14	1481.7	741.4	1464.7	1463.7	V	360.2		343.2		3
15	1595.8	798.4	1578.7	1577.8	N	261.2		244.1		2
16	1741.9	871.4	1724.9	1723.9	K	147.1		130.1		1

Gene symbol: Atp6v1e1
Protein name: Vacuolar ATP synthase subunit E 1
Protein accession numbers: IPI00119115
Peptide sequence: (R)ARDDLITDLLNEAK(Q)
Exclusive (unique to this protein): TRUE
SEQUEST XCorr score: 2.12 SEQUEST DCn score: 0.586
Fix modifications: None
Variable modifications: None
charge: 2 Observed m/z: 793.9243
Actual minus calculated peptide mass (AMU): 0.0015855

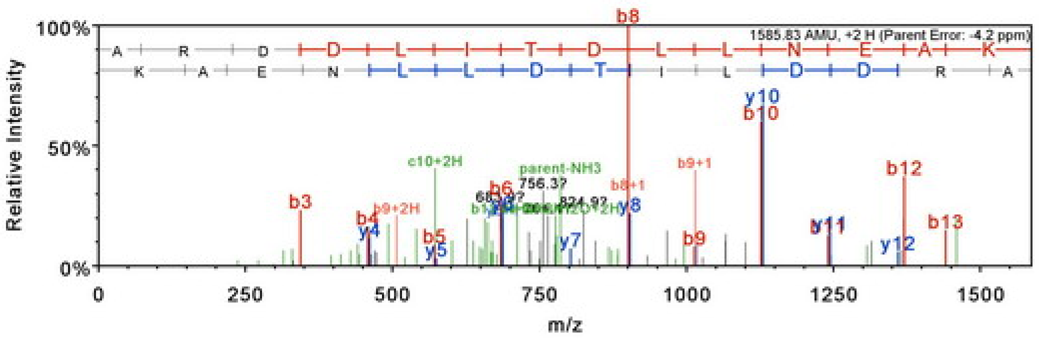

B	B Ions	B+2H	B-NH3	B-H2O	AA	Y Ions	Y+2H	Y-NH3	Y-H2O	Y
1	72.0				A	1586.8	793.9	1569.8	1568.8	14
2	228.2	114.6	211.1		R	1515.8	758.4	1498.8	1497.8	13
3	343.2	172.1	326.2	325.2	D	1359.7	680.4	1342.7	1341.7	12
4	458.2	229.6	441.2	440.2	D	1244.7	622.8	1227.7	1226.7	11
5	571.3	286.2	554.3	553.3	L	1129.7	565.3	1112.6	1111.6	10
6	684.4	342.7	667.3	666.4	I	1016.6	508.8	999.5	998.6	9
7	785.4	393.2	768.4	767.4	T	903.5	452.2	886.5	885.5	8
8	900.4	450.7	883.4	882.4	D	802.4	401.7	785.4	784.4	7
9	1013.5	507.3	996.5	995.5	L	687.4	344.2	670.4	669.4	6
10	1126.6	563.8	1109.6	1108.6	L	574.3		557.3	556.3	5
11	1240.7	620.8	1223.6	1222.6	N	461.2		444.2	443.2	4
12	1369.7	685.4	1352.7	1351.7	E	347.2		330.2	329.2	3
13	1440.7	720.9	1423.7	1422.7	A	218.2		201.1		2
14	1586.8	793.9	1569.8	1568.8	K	147.1		130.1		1

Gene symbol: AU042651

Protein name: Expressed sequence AU042651

Protein accession numbers: IPI00226116

Peptide sequence: (R)LQAGVGYANTFNCIR(M)

Exclusive (unique to this protein): TRUE

SEQUEST XCorr score: 2.11 SEQUEST DCn score: 0.496

Fix modifications: C13: Carbamidomethyl (+57.02)Variable modifications: None

charge: 2 Observed m/z: 842.4202

Actual minus calculated peptide mass (AMU): 0.004883

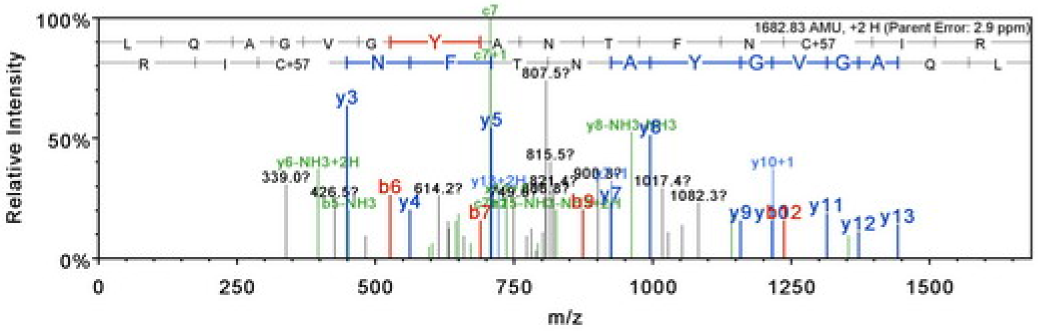

B	B Ions	B+2H	B-NH3	B-H2O	AA	Y Ions	Y+2H	Y-NH3	Y-H2O	Y
1	114.1				L	1683.8	842.4	1666.8	1665.8	15
2	242.2		225.1		Q	1570.7	785.9	1553.7	1552.7	14
3	313.2		296.2		A	1442.7	721.9	1425.7	1424.7	13
4	370.2		353.2		G	1371.7	686.3	1354.6	1353.6	12
5	469.3		452.3		V	1314.6	657.8	1297.6	1296.6	11
6	526.3	263.6	509.3		G	1215.6	608.3	1198.5	1197.5	10
7	689.4	345.2	672.3		Y	1158.5	579.8	1141.5	1140.5	9
8	760.4	380.7	743.4		A	995.5	498.2	978.5	977.5	8
9	874.4	437.7	857.4		N	924.4	462.7	907.4	906.4	7
10	975.5	488.3	958.5	957.5	T	810.4	405.7	793.4	792.4	6
11	1122.6	561.8	1105.5	1104.5	F	709.4		692.3		5
12	1236.6	618.8	1219.6	1218.6	N	562.3		545.3		4
13	1396.6	698.8	1379.6	1378.6	C+57	448.2		431.2		3
14	1509.7	755.4	1492.7	1491.7	I	288.2		271.2		2
15	1683.8	842.4	1666.8	1665.8	R	175.1		158.1		1

Gene symbol: BC023882

Protein name: CDNA sequence BC023882

Protein accession numbers: IPI00387484

Peptide sequence: (K)HWDTYRQLESLVQQLSQR(N)

Exclusive (unique to this protein): TRUE

SEQUEST XCorr score: 2.14 SEQUEST DCn score: 0.396

Fix modifications: None

Variable modifications: None

charge: 2 Observed m/z: 1144.0933

Actual minus calculated peptide mass (AMU): 0.020254

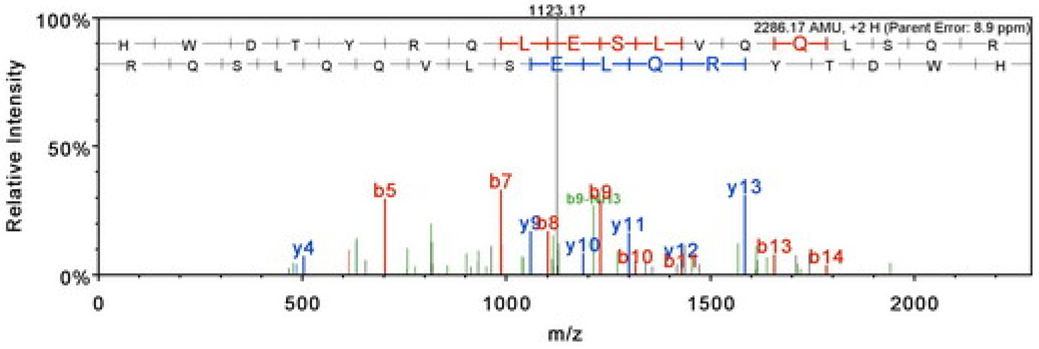

B	B Ions	B+2H	B-NH3	B-H2O	AA	Y Ions	Y+2H	Y-NH3	Y-H2O	Y
1	138.1	69.5			H	2287.2	1144.1	2270.1	2269.2	18
2	324.2	162.6			W	2150.1	1075.5	2133.1	2132.1	17
3	439.2	220.1		421.2	D	1964.0	982.5	1947.0	1946.0	16
4	540.2	270.6		522.2	T	1849.0	925.0	1832.0	1831.0	15
5	703.3	352.2		685.3	Y	1748.0	874.5	1730.9	1729.9	14
6	859.4	430.2	842.4	841.4	R	1584.9	793.0	1567.9	1566.9	13
7	987.4	494.2	970.4	969.4	Q	1428.8	714.9	1411.8	1410.8	12
8	1100.5	550.8	1083.5	1082.5	L	1300.7	650.9	1283.7	1282.7	11
9	1229.6	615.3	1212.5	1211.6	E	1187.6	594.3	1170.6	1169.6	10
10	1316.6	658.8	1299.6	1298.6	S	1058.6	529.8	1041.6	1040.6	9
11	1429.7	715.4	1412.7	1411.7	L	971.6	486.3	954.5	953.6	8
12	1528.8	764.9	1511.7	1510.7	V	858.5	429.7	841.5	840.5	7
13	1656.8	828.9	1639.8	1638.8	Q	759.4	380.2	742.4	741.4	6
14	1784.9	892.9	1767.9	1766.9	Q	631.4		614.3	613.3	5
15	1898.0	949.5	1880.9	1880.0	L	503.3		486.3	485.3	4
16	1985.0	993.0	1968.0	1967.0	S	390.2		373.2	372.2	3
17	2113.1	1057.0	2096.0	2095.0	Q	303.2		286.2		2
18	2287.2	1144.1	2270.1	2269.2	R	175.1		158.1		1

Gene symbol: Bcap31

Protein name: B-cell receptor-associated protein 31

Protein accession numbers: IPI00230422,IPI00828225

Peptide sequence: (R)LVTLISQQATLLASNEAFK(K)

Exclusive (unique to this protein): TRUE

SEQUEST XCorr score: 3.01 SEQUEST DCn score: 0.716

Fix modifications: None

Variable modifications: None

charge: 2 Observed m/z: 1024.0746

Actual minus calculated peptide mass (AMU): -0.002686

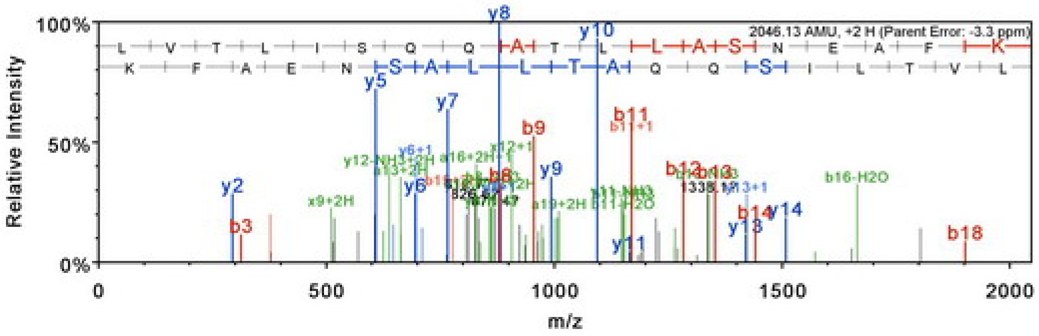

B	B Ions	B+2H	B-NH3	B-H2O	AA	Y Ions	Y+2H	Y-NH3	Y-H2O	Y
1	138.1	69.5			H	2287.2	1144.1	2270.1	2269.2	18
2	324.2	162.6			W	2150.1	1075.5	2133.1	2132.1	17
3	439.2	220.1		421.2	D	1964.0	982.5	1947.0	1946.0	16
4	540.2	270.6		522.2	T	1849.0	925.0	1832.0	1831.0	15
5	703.3	352.2		685.3	Y	1748.0	874.5	1730.9	1729.9	14
6	859.4	430.2	842.4	841.4	R	1584.9	793.0	1567.9	1566.9	13
7	987.4	494.2	970.4	969.4	Q	1428.8	714.9	1411.8	1410.8	12
8	1100.5	550.8	1083.5	1082.5	L	1300.7	650.9	1283.7	1282.7	11
9	1229.6	615.3	1212.5	1211.6	E	1187.6	594.3	1170.6	1169.6	10
10	1316.6	658.8	1299.6	1298.6	S	1058.6	529.8	1041.6	1040.6	9
11	1429.7	715.4	1412.7	1411.7	L	971.6	486.3	954.5	953.6	8
12	1528.8	764.9	1511.7	1510.7	V	858.5	429.7	841.5	840.5	7
13	1656.8	828.9	1639.8	1638.8	Q	759.4	380.2	742.4	741.4	6
14	1784.9	892.9	1767.9	1766.9	Q	631.4		614.3	613.3	5
15	1898.0	949.5	1880.9	1880.0	L	503.3		486.3	485.3	4
16	1985.0	993.0	1968.0	1967.0	S	390.2		373.2	372.2	3
17	2113.1	1057.0	2096.0	2095.0	Q	303.2		286.2		2
18	2287.2	1144.1	2270.1	2269.2	R	175.1		158.1		1

Gene symbol: C1qbp

Protein name: complement component 1, q subcomponent binding protein

Protein accession numbers: IPI00132799

Peptide sequence: (K)AEEQEPELTSTPNFVVEVTK(T)

Exclusive (unique to this protein): TRUE

SEQUEST XCorr score: 2.12 SEQUEST DCn score: 0.582

Fix modifications: None

Variable modifications: None

charge: 2 Observed m/z: 1124.0574

Actual minus calculated peptide mass (AMU): 0.0039054

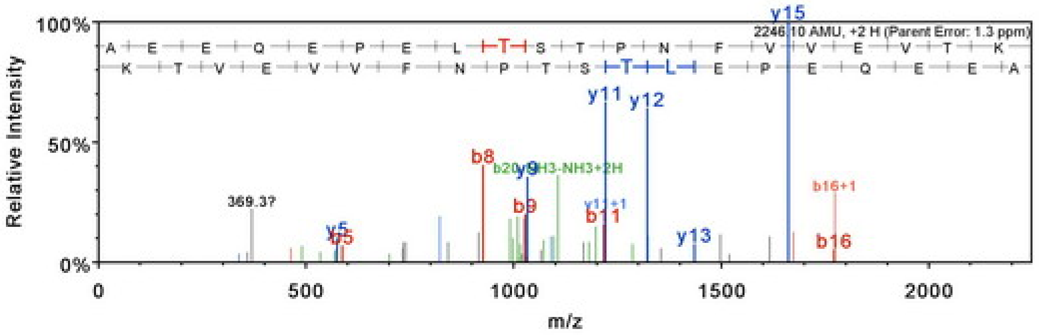

B	B Ions	B+2H	B-NH3	B-H2O	AA	Y Ions	Y+2H	Y-NH3	Y-H2O	Y
1	72.0				A	2247.1	1124.1	2230.1	2229.1	20
2	201.1			183.1	E	2176.1	1088.5	2159.0	2158.1	19
3	330.1			312.1	E	2047.0	1024.0	2030.0	2029.0	18
4	458.2		441.2	440.2	Q	1918.0	959.5	1901.0	1900.0	17
5	587.2		570.2	569.2	E	1789.9	895.5	1772.9	1771.9	16
6	684.3	342.7	667.3	666.3	P	1660.9	830.9	1643.9	1642.9	15
7	813.3	407.2	796.3	795.3	E	1563.8	782.4	1546.8	1545.8	14
8	926.4	463.7	909.4	908.4	L	1434.8	717.9	1417.8	1416.8	13
9	1027.5	514.2	1010.4	1009.5	T	1321.7	661.4	1304.7	1303.7	12
10	1114.5	557.8	1097.5	1096.5	S	1220.7	610.8	1203.6	1202.6	11
11	1215.5	608.3	1198.5	1197.5	T	1133.6	567.3	1116.6	1115.6	10
12	1312.6	656.8	1295.6	1294.6	P	1032.6	516.8	1015.6	1014.6	9
13	1426.6	713.8	1409.6	1408.6	N	935.5	468.3	918.5	917.5	8
14	1573.7	787.4	1556.7	1555.7	F	821.5	411.2	804.5	803.5	7
15	1672.8	836.9	1655.7	1654.8	V	674.4	337.7	657.4	656.4	6
16	1771.8	886.4	1754.8	1753.8	V	575.3		558.3	557.3	5
17	1900.9	950.9	1883.9	1882.9	E	476.3		459.3	458.3	4
18	2000.0	1000.5	1982.9	1981.9	V	347.2		330.2	329.2	3
19	2101.0	1051.0	2084.0	2083.0	T	248.2		231.1	230.2	2
20	2247.1	1124.1	2230.1	2229.1	K	147.1		130.1		1

Gene symbol: C730024G19Rik

Protein name: similar to F-box/LRR-repeat protein 20 (F-box and leucine-rich repeat protein 20) (F-box/LRR-repeat protein 2-like) isoform 1

Protein accession numbers: IPI00658665,IPI00674232,IPI00761387

Peptide sequence: (R)ITDSNISEVLHPEVQR(L)

Exclusive (unique to this protein): TRUE

SEQUEST XCorr score: 2.54 SEQUEST DCn score: 0.524

Fix modifications: None

Variable modifications: None

charge: 2 Observed m/z: 918.9774

Actual minus calculated peptide mass (AMU): 0.001343

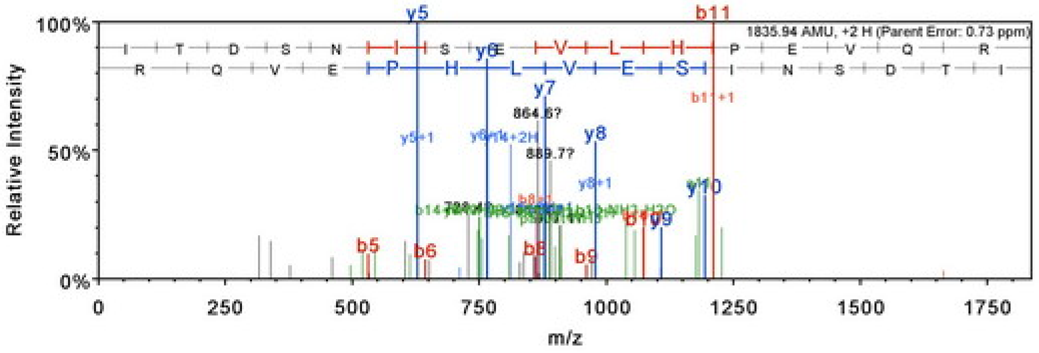

B	B Ions	B+2H	B-NH3	B-H2O	AA	Y Ions	Y+2H	Y-NH3	Y-H2O	Y
1	114.1				I	1837.0	919.0	1819.9	1818.9	16
2	215.1			197.1	T	1723.9	862.4	1706.8	1705.9	15
3	330.2			312.2	D	1622.8	811.9	1605.8	1604.8	14
4	417.2			399.2	S	1507.8	754.4	1490.8	1489.8	13
5	531.2		514.2	513.2	N	1420.8	710.9	1403.7	1402.7	12
6	644.3	322.7	627.3	626.3	I	1306.7	653.9	1289.7	1288.7	11
7	731.4	366.2	714.3	713.4	S	1193.6	597.3	1176.6	1175.6	10
8	860.4	430.7	843.4	842.4	E	1106.6	553.8	1089.6	1088.6	9
9	959.5	480.2	942.4	941.5	V	977.6	489.3	960.5	959.5	8
10	1072.5	536.8	1055.5	1054.5	L	878.5	439.8	861.5	860.5	7
11	1209.6	605.3	1192.6	1191.6	H	765.4	383.2	748.4	747.4	6
12	1306.7	653.8	1289.6	1288.7	P	628.3		611.3	610.3	5
13	1435.7	718.4	1418.7	1417.7	E	531.3		514.3	513.3	4
14	1534.8	767.9	1517.8	1516.8	V	402.3		385.2		3
15	1662.8	831.9	1645.8	1644.8	Q	303.2		286.2		2
16	1837.0	919.0	1819.9	1818.9	R	175.1		158.1		1

Gene symbol: Calm3;Calm1;Calm2

Protein name: 12 days pregnant adult female placenta cDNA, RIKEN full-length enriched library, clone:I530005B05 product:calmodulin 1, full insert sequence

Protein accession numbers: IPI00467841,IPI00761696

Peptide sequence: (R)VFDKDGNGYISAAELR(H)

Exclusive (unique to this protein): TRUE

SEQUEST XCorr score: 2.6 SEQUEST DCn score: 0.59

Fix modifications: None

Variable modifications: None

charge: 2 Observed m/z: 877.941

Actual minus calculated peptide mass (AMU): 0.002686

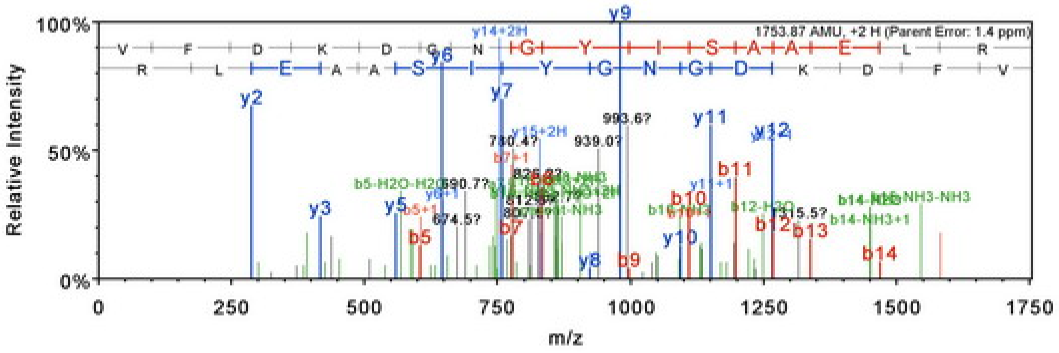

B	B Ions	B+2H	B-NH3	B-H2O	AA	Y Ions	Y+2H	Y-NH3	Y-H2O	Y
1	100.1				V	1754.9	877.9	1737.8	1736.9	16
2	247.1				F	1655.8	828.4	1638.8	1637.8	15
3	362.2			344.2	D	1508.7	754.9	1491.7	1490.7	14
4	490.3	245.6	473.2	472.3	K	1393.7	697.4	1376.7	1375.7	13
5	605.3	303.2	588.3	587.3	D	1265.6	633.3	1248.6	1247.6	12
6	662.3	331.7	645.3	644.3	G	1150.6	575.8	1133.6	1132.6	11
7	776.4	388.7	759.3	758.4	N	1093.6	547.3	1076.5	1075.5	10
8	833.4	417.2	816.4	815.4	G	979.5	490.3	962.5	961.5	9
9	996.4	498.7	979.4	978.4	Y	922.5	461.8	905.5	904.5	8
10	1109.5	555.3	1092.5	1091.5	I	759.4	380.2	742.4	741.4	7
11	1196.6	598.8	1179.5	1178.5	S	646.4	323.7	629.3	628.3	6
12	1267.6	634.3	1250.6	1249.6	A	559.3		542.3	541.3	5
13	1338.6	669.8	1321.6	1320.6	A	488.3		471.3	470.3	4
14	1467.7	734.3	1450.7	1449.7	E	417.3		400.2	399.2	3
15	1580.8	790.9	1563.7	1562.8	L	288.2		271.2		2
16	1754.9	877.9	1737.8	1736.9	R	175.1		158.1		1

Gene symbol: Calr

Protein name: Calreticulin precursor

Protein accession numbers: IPI00123639

Peptide sequence: (K)EQFLDGDAWTNR(W)

Exclusive (unique to this protein): TRUE

SEQUEST XCorr score: 2.15 SEQUEST DCn score: 0.604

Fix modifications: None

Variable modifications: None

charge: 2 Observed m/z: 726.3289

Actual minus calculated peptide mass (AMU): -0.005737

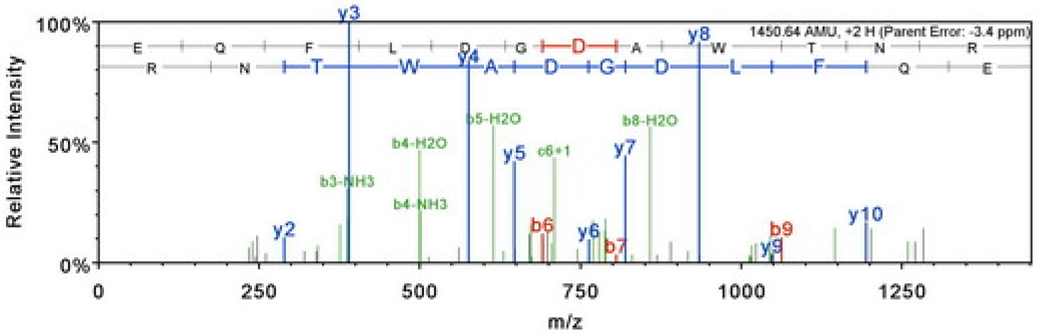

B	B Ions	B+2H	B-NH3	B-H2O	AA	Y Ions	Y+2H	Y-NH3	Y-H2O	Y
1	130.1			112.0	E	1451.7	726.3	1434.6	1433.7	12
2	258.1		241.1	240.1	Q	1322.6	661.8	1305.6	1304.6	11
3	405.2		388.2	387.2	F	1194.5	597.8	1177.5	1176.5	10
4	518.3		501.2	500.3	L	1047.5	524.3	1030.5	1029.5	9
5	633.3		616.3	615.3	D	934.4	467.7	917.4	916.4	8
6	690.3	345.7	673.3	672.3	G	819.4	410.2	802.4	801.4	7
7	805.3	403.2	788.3	787.3	D	762.4	381.7	745.3	744.3	6
8	876.4	438.7	859.4	858.4	A	647.3		630.3	629.3	5
9	1062.5	531.7	1045.4	1044.4	W	576.3		559.3	558.3	4
10	1163.5	582.3	1146.5	1145.5	T	390.2		373.2	372.2	3
11	1277.5	639.3	1260.5	1259.5	N	289.2		272.1		2
12	1451.7	726.3	1434.6	1433.7	R	175.1		158.1		1

Gene symbol: Cap1

Protein name: Adenylyl cyclase-associated protein 1

Protein accession numbers: IPI00137331

Peptide sequence: (R)VENQENVSNLVIDDTELK(Q)

Exclusive (unique to this protein): TRUE

SEQUEST XCorr score: 2.07 SEQUEST DCn score: 0.646

Fix modifications: None

Variable modifications: None

charge: 2 Observed m/z: 1030.0109

Actual minus calculated peptide mass (AMU): -0.0056153

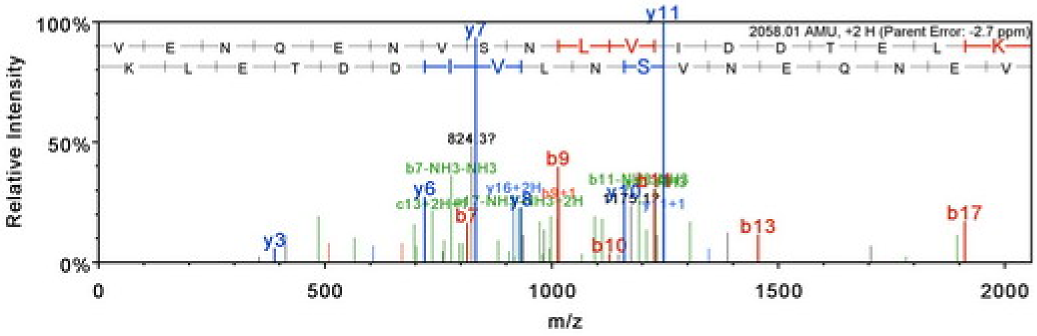

B	B Ions	B+2H	B-NH3	B-H2O	AA	Y Ions	Y+2H	Y-NH3	Y-H2O	Y
1	100.1				V	2059.0	1030.0	2042.0	2041.0	18
2	229.1			211.1	E	1960.0	980.5	1942.9	1941.9	17
3	343.2		326.1	325.2	N	1830.9	916.0	1813.9	1812.9	16
4	471.2		454.2	453.2	Q	1716.9	858.9	1699.8	1698.9	15
5	600.3		583.2	582.3	E	1588.8	794.9	1571.8	1570.8	14
6	714.3	357.7	697.3	696.3	N	1459.8	730.4	1442.7	1441.8	13
7	813.4	407.2	796.4	795.4	V	1345.7	673.4	1328.7	1327.7	12
8	900.4	450.7	883.4	882.4	S	1246.7	623.8	1229.6	1228.6	11
9	1014.5	507.7	997.4	996.4	N	1159.6	580.3	1142.6	1141.6	10
10	1127.5	564.3	1110.5	1109.5	L	1045.6	523.3	1028.5	1027.6	9
11	1226.6	613.8	1209.6	1208.6	V	932.5	466.8	915.5	914.5	8
12	1339.7	670.4	1322.7	1321.7	I	833.4	417.2	816.4	815.4	7
13	1454.7	727.9	1437.7	1436.7	D	720.3	360.7	703.3	702.3	6
14	1569.7	785.4	1552.7	1551.7	D	605.3		588.3	587.3	5
15	1670.8	835.9	1653.8	1652.8	T	490.3		473.3	472.3	4
16	1799.8	900.4	1782.8	1781.8	E	389.2		372.2	371.2	3
17	1912.9	957.0	1895.9	1894.9	L	260.2		243.2		2
18	2059.0	1030.0	2042.0	2041.0	K	147.1		130.1		1

Gene symbol: Capza1

Protein name: F-actin capping protein subunit alpha-1

Protein accession numbers: IPI00330063,IPI00653841,IPI00848950

Peptide sequence: (R)LLLNNDNLLR(E)

Exclusive (unique to this protein): TRUE

SEQUEST XCorr score: 2.19 SEQUEST DCn score: 0.555

Fix modifications: None

Variable modifications: None

charge: 2 Observed m/z: 599.3509

Actual minus calculated peptide mass (AMU): -0.001709

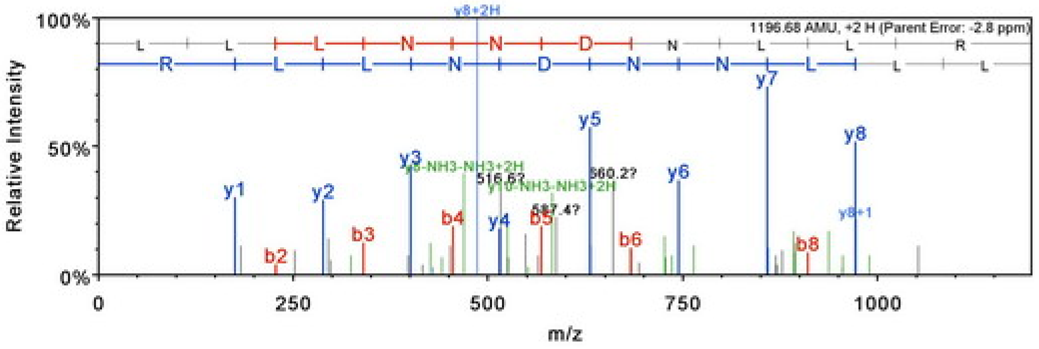

B	B Ions	B+2H	B-NH3	B-H2O	AA	Y Ions	Y+2H	Y-NH3	Y-H2O	Y
1	114.1				L	1197.7	599.4	1180.7	1179.7	10
2	227.2				L	1084.6	542.8	1067.6	1066.6	9
3	340.3				L	971.5	486.3	954.5	953.5	8
4	454.3		437.3		N	858.4	429.7	841.4	840.4	7
5	568.4		551.3		N	744.4	372.7	727.4	726.4	6
6	683.4	342.2	666.4	665.4	D	630.4		613.3	612.4	5
7	797.4	399.2	780.4	779.4	N	515.3		498.3		4
8	910.5	455.8	893.5	892.5	L	401.3		384.3		3
9	1023.6	512.3	1006.6	1005.6	L	288.2		271.2		2
10	1197.7	599.4	1180.7	1179.7	R	175.1		158.1		1

Gene symbol: Carhsp1

Protein name: Calcium-regulated heat stable protein 1

Protein accession numbers: IPI00133349,IPI00475159

Peptide sequence: (K)LQAVEVVITHLAPGTK(H)

Exclusive (unique to this protein): TRUE

SEQUEST XCorr score: 2.13 SEQUEST DCn score: 0.515

Fix modifications: None

Variable modifications: None

charge: 2 Observed m/z: 838.4866

Actual minus calculated peptide mass (AMU): -0.009644

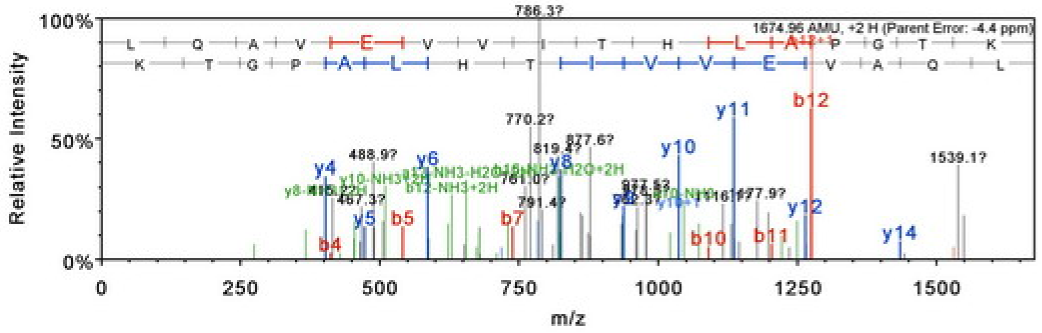

B	B Ions	B+2H	B-NH3	B-H2O	AA	Y Ions	Y+2H	Y-NH3	Y-H2O	Y
1	114.1				L	1676.0	838.5	1659.0	1658.0	16
2	242.2		225.1		Q	1562.9	782.0	1545.9	1544.9	15
3	313.2		296.2		A	1434.8	717.9	1417.8	1416.8	14
4	412.3		395.2		V	1363.8	682.4	1346.8	1345.8	13
5	541.3		524.3	523.3	E	1264.7	632.9	1247.7	1246.7	12
6	640.4	320.7	623.3	622.4	V	1135.7	568.4	1118.7	1117.7	11
7	739.4	370.2	722.4	721.4	V	1036.6	518.8	1019.6	1018.6	10
8	852.5	426.8	835.5	834.5	I	937.6	469.3	920.5	919.5	9
9	953.6	477.3	936.5	935.6	T	824.5	412.7	807.4	806.5	8
10	1090.6	545.8	1073.6	1072.6	H	723.4	362.2	706.4	705.4	7
11	1203.7	602.4	1186.7	1185.7	L	586.4	293.7	569.3	568.4	6
12	1274.8	637.9	1257.7	1256.7	A	473.3		456.3	455.3	5
13	1371.8	686.4	1354.8	1353.8	P	402.2		385.2	384.2	4
14	1428.8	714.9	1411.8	1410.8	G	305.2		288.2	287.2	3
15	1529.9	765.4	1512.8	1511.9	T	248.2		231.1	230.2	2
16	1676.0	838.5	1659.0	1658.0	K	147.1		130.1		1

Gene symbol: Ccdc117
Protein name: Coiled-coil domain-containing protein 117
Protein accession numbers: IPI00321929
Peptide sequence: (R)TEGLLEPQCADAPLYR(S)
Exclusive (unique to this protein): TRUE
SEQUEST XCorr score: 2.17 SEQUEST DCn score: 0.633
Fix modifications: C9: Carbamidomethyl (+57.02)Variable modifications: None
charge: 2 Observed m/z: 916.9447
Actual minus calculated peptide mass (AMU): -0.0040256

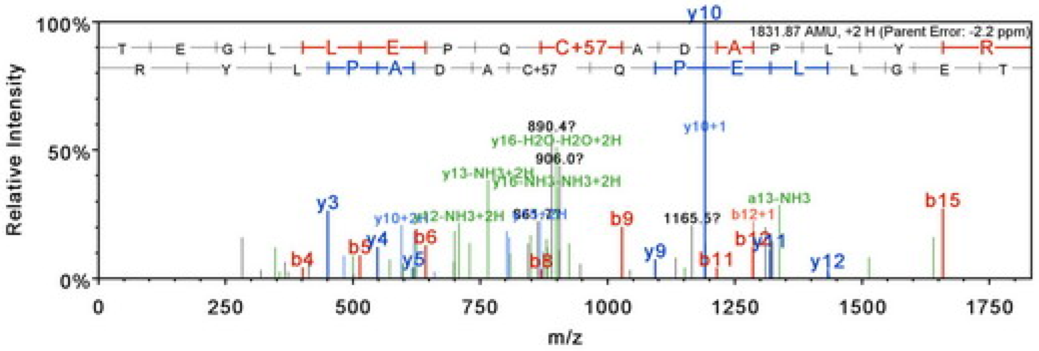

B	B Ions	B+2H	B-NH3	B-H2O	AA	Y Ions	Y+2H	Y-NH3	Y-H2O	Y
1	102.1			84.0	T	1832.9	917.0	1815.9	1814.9	16
2	231.1			213.1	E	1731.8	866.4	1714.8	1713.8	15
3	288.1			270.1	G	1602.8	801.9	1585.8	1584.8	14
4	401.2			383.2	L	1545.8	773.4	1528.8	1527.8	13
5	514.3			496.3	L	1432.7	716.9	1415.7	1414.7	12
6	643.3	322.2		625.3	E	1319.6	660.3	1302.6	1301.6	11
7	740.4	370.7		722.4	P	1190.6	595.8	1173.5	1172.5	10
8	868.4	434.7	851.4	850.4	Q	1093.5	547.3	1076.5	1075.5	9
9	1028.5	514.7	1011.5	1010.5	C+57	965.5	483.2	948.4	947.4	8
10	1099.5	550.3	1082.5	1081.5	A	805.4	403.2	788.4	787.4	7
11	1214.5	607.8	1197.5	1196.5	D	734.4	367.7	717.4	716.4	6
12	1285.6	643.3	1268.5	1267.6	A	619.4		602.3		5
13	1382.6	691.8	1365.6	1364.6	P	548.3		531.3		4
14	1495.7	748.4	1478.7	1477.7	L	451.3		434.2		3
15	1658.8	829.9	1641.8	1640.8	Y	338.2		321.2		2
16	1832.9	917.0	1815.9	1814.9	R	175.1		158.1		1

Gene symbol: Cct5
Protein name: T-complex protein 1 subunit epsilon
Protein accession numbers: IPI00116279
Peptide sequence: (K)VLVDINNPEPLIQTAK(T)
Exclusive (unique to this protein): TRUE
SEQUEST XCorr score: 2.18 SEQUEST DCn score: 0.615
Fix modifications: None

Variable modifications: None

charge: 2 Observed m/z: 882.4982

Actual minus calculated peptide mass (AMU): -0.002564

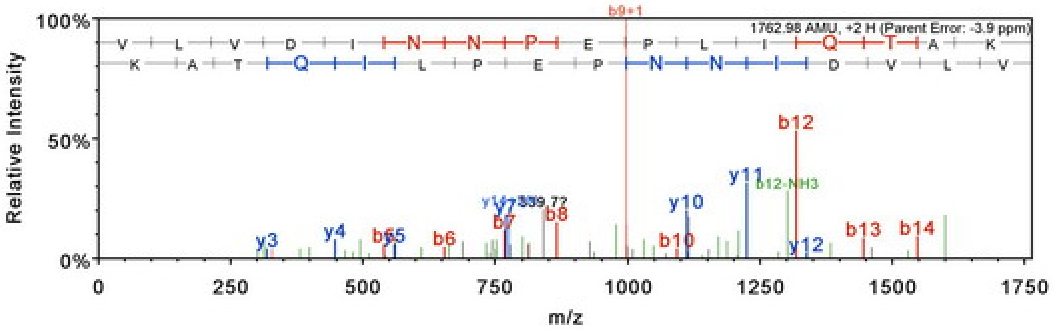

B	B Ions	B+2H	B-NH3	B-H2O	AA	Y Ions	Y+2H	Y-NH3	Y-H2O	Y
1	100.1				V	1764.0	882.5	1747.0	1746.0	16
2	213.2				L	1664.9	833.0	1647.9	1646.9	15
3	312.2				V	1551.8	776.4	1534.8	1533.8	14
4	427.3			409.3	D	1452.8	726.9	1435.7	1434.8	13
5	540.3			522.3	I	1337.7	669.4	1320.7	1319.7	12
6	654.4	327.7	637.4	636.4	N	1224.7	612.8	1207.6	1206.7	11
7	768.4	384.7	751.4	750.4	N	1110.6	555.8	1093.6	1092.6	10
8	865.5	433.2	848.5	847.5	P	996.6	498.8	979.6	978.6	9
9	994.5	497.8	977.5	976.5	E	899.5	450.3	882.5	881.5	8
10	1091.6	546.3	1074.5	1073.6	P	770.5	385.7	753.5	752.5	7
11	1204.7	602.8	1187.6	1186.7	L	673.4	337.2	656.4	655.4	6
12	1317.7	659.4	1300.7	1299.7	I	560.3		543.3	542.3	5
13	1445.8	723.4	1428.8	1427.8	Q	447.3		430.2	429.3	4
14	1546.9	773.9	1529.8	1528.8	T	319.2		302.2	301.2	3
15	1617.9	809.5	1600.9	1599.9	A	218.2		201.1		2
16	1764.0	882.5	1747.0	1746.0	K	147.1		130.1		1

Gene symbol: Cdo1

Protein name: Cysteine dioxygenase type 1

Protein accession numbers: IPI00134144

Peptide sequence: (R)ENQCAYINDSIGLHR(V)

Exclusive (unique to this protein): TRUE

SEQUEST XCorr score: 2.67 SEQUEST DCn score: 0.645

Fix modifications: C4: Carbamidomethyl (+57.02)Variable modifications: None

charge: 2 Observed m/z: 895.421

Actual minus calculated peptide mass (AMU): 0.005005

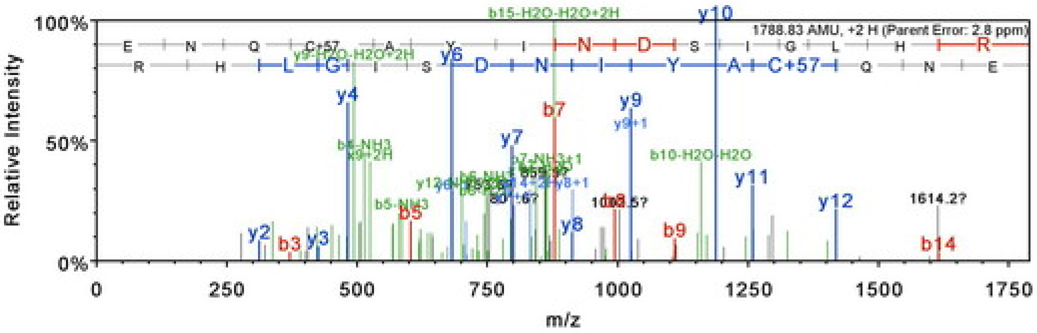

B	B Ions	B+2H	B-NH3	B-H2O	AA	Y Ions	Y+2H	Y-NH3	Y-H2O	Y
1	130.1			112.0	E	1789.8	895.4	1772.8	1771.8	15
2	244.1		227.1	226.1	N	1660.8	830.9	1643.8	1642.8	14
3	372.2		355.1	354.1	Q	1546.7	773.9	1529.7	1528.7	13
4	532.2		515.2	514.2	C+57	1418.7	709.9	1401.7	1400.7	12
5	603.2		586.2	585.2	A	1258.7	629.8	1241.6	1240.6	11
6	766.3	383.7	749.3	748.3	Y	1187.6	594.3	1170.6	1169.6	10
7	879.4	440.2	862.3	861.4	I	1024.5	512.8	1007.5	1006.5	9
8	993.4	497.2	976.4	975.4	N	911.5	456.2	894.4	893.5	8
9	1108.4	554.7	1091.4	1090.4	D	797.4	399.2	780.4	779.4	7
10	1195.5	598.2	1178.4	1177.5	S	682.4	341.7	665.4	664.4	6
11	1308.5	654.8	1291.5	1290.5	I	595.4	298.2	578.3		5
12	1365.6	683.3	1348.5	1347.6	G	482.3	241.7	465.3		4
13	1478.7	739.8	1461.6	1460.7	L	425.3	213.1	408.2		3
14	1615.7	808.4	1598.7	1597.7	H	312.2	156.6	295.2		2
15	1789.8	895.4	1772.8	1771.8	R	175.1		158.1		1

Gene symbol: Chchd2

Protein name: Coiled-coil-helix-coiled-coil-helix domain-containing protein 2

Protein accession numbers: IPI00284925,IPI00407413

Peptide sequence: (K)LCEGFNEVLR(Q)

Exclusive (unique to this protein): TRUE

SEQUEST XCorr score: 2.02 SEQUEST DCn score: 0.428

Fix modifications: C2: Carbamidomethyl (+57.02)Variable modifications: None

charge: 2 Observed m/z: 618.8061

Actual minus calculated peptide mass (AMU): -0.0004883

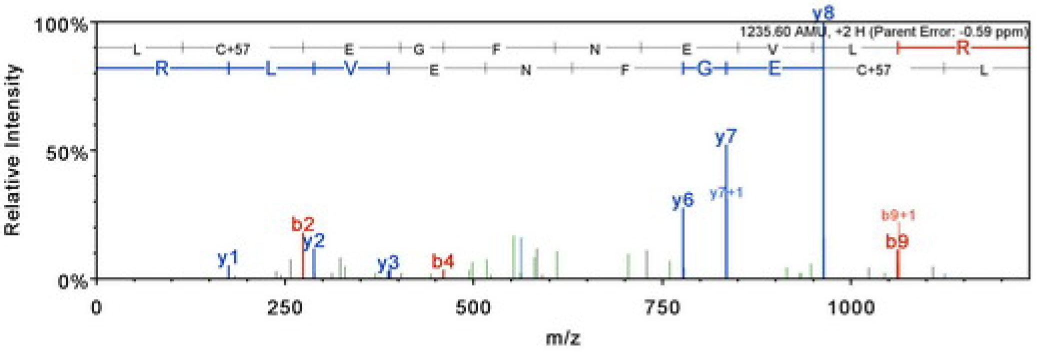

B	B Ions	B+2H	B-NH3	B-H2O	AA	Y Ions	Y+2H	Y-NH3	Y-H2O	Y
1	114.1				L	1236.6	618.8	1219.6	1218.6	10
2	274.1				C+57	1123.5	562.3	1106.5	1105.5	9
3	403.2			385.2	E	963.5	482.3	946.5	945.5	8
4	460.2			442.2	G	834.5	417.7	817.4	816.4	7
5	607.3			589.2	F	777.4	389.2	760.4	759.4	6
6	721.3	361.2	704.3	703.3	N	630.4		613.3	612.4	5
7	850.3	425.7	833.3	832.3	E	516.3		499.3	498.3	4
8	949.4	475.2	932.4	931.4	V	387.3		370.3		3
9	1062.5	531.8	1045.5	1044.5	L	288.2		271.2		2
10	1236.6	618.8	1219.6	1218.6	R	175.1		158.1		1

Gene symbol: Chchd6

Protein name: Coiled-coil-helix-coiled-coil-helix domain-containing protein 6

Protein accession numbers: IPI00313390

Peptide sequence: (R)RVEPVCSGLQAQILR(C)

Exclusive (unique to this protein): TRUE

SEQUEST XCorr score: 2.24 SEQUEST DCn score: 0.467

Fix modifications: C6: Carbamidomethyl (+57.02)Variable modifications: None

charge: 2 Observed m/z: 863.4779

Actual minus calculated peptide mass (AMU): 0.004395

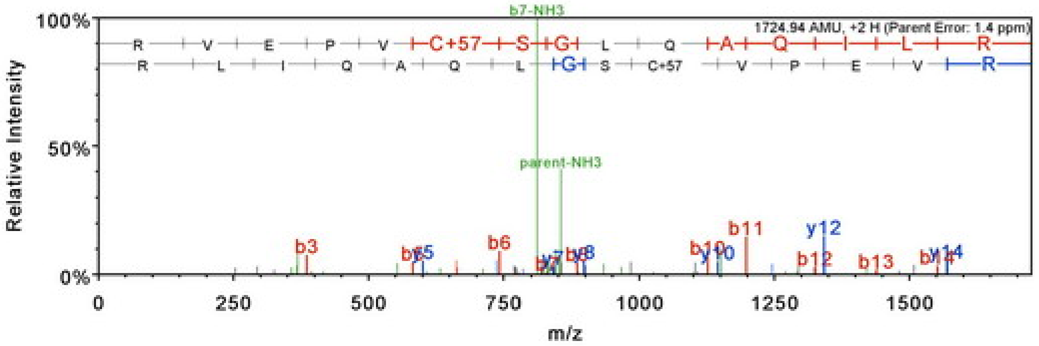

B	B Ions	B+2H	B-NH3	B-H2O	AA	Y Ions	Y+2H	Y-NH3	Y-H2O	Y
1	157.1	79.1	140.1		R	1725.9	863.5	1708.9	1707.9	15
2	256.2	128.6	239.2		V	1569.8	785.4	1552.8	1551.8	14
3	385.2	193.1	368.2	367.2	E	1470.8	735.9	1453.8	1452.8	13
4	482.3	241.6	465.3	464.3	P	1341.7	671.4	1324.7	1323.7	12
5	581.3	291.2	564.3	563.3	V	1244.7	622.8	1227.7	1226.7	11
6	741.4	371.2	724.4	723.4	C+57	1145.6	573.3	1128.6	1127.6	10
7	828.4	414.7	811.4	810.4	S	985.6	493.3	968.6	967.6	9
8	885.4	443.2	868.4	867.4	G	898.6	449.8	881.5		8
9	998.5	499.8	981.5	980.5	L	841.5	421.3	824.5		7
10	1126.6	563.8	1109.5	1108.6	Q	728.4	364.7	711.4		6
11	1197.6	599.3	1180.6	1179.6	A	600.4		583.4		5
12	1325.7	663.3	1308.6	1307.7	Q	529.4		512.3		4
13	1438.8	719.9	1421.7	1420.7	I	401.3		384.3		3
14	1551.8	776.4	1534.8	1533.8	L	288.2		271.2		2
15	1725.9	863.5	1708.9	1707.9	R	175.1		158.1		1

Gene symbol: Chmp5

Protein name: Charged multivesicular body protein 5

Protein accession numbers: IPI00110729

Peptide sequence: (K)APPPSLTDCIGTVDSR(A)

Exclusive (unique to this protein): TRUE

SEQUEST XCorr score: 2.11 SEQUEST DCn score: 0.604

Fix modifications: C9: Carbamidomethyl (+57.02)Variable modifications: None

charge: 2 Observed m/z: 843.4141

Actual minus calculated peptide mass (AMU): 0.003296

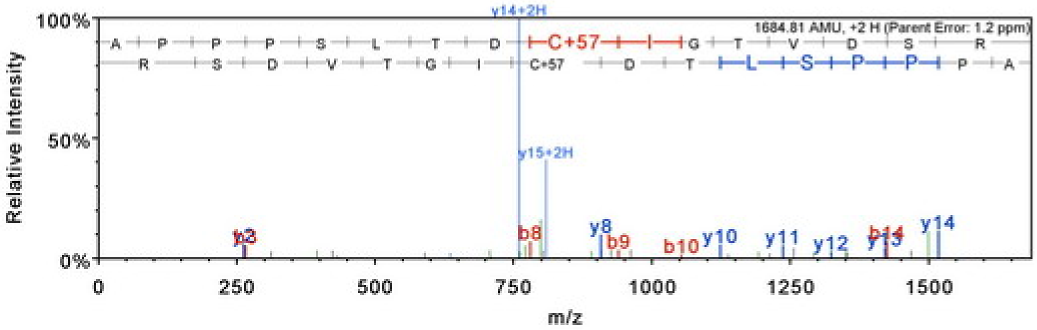

B	B Ions	B+2H	B-NH3	B-H2O	AA	Y Ions	Y+2H	Y-NH3	Y-H2O	Y
1	72.0				A	1685.8	843.4	1668.8	1667.8	16
2	169.1				P	1614.8	807.9	1597.8	1596.8	15
3	266.1				P	1517.7	759.4	1500.7	1499.7	14
4	363.2				P	1420.7	710.8	1403.7	1402.7	13
5	450.2			432.2	S	1323.6	662.3	1306.6	1305.6	12
6	563.3	282.2		545.3	L	1236.6	618.8	1219.6	1218.6	11
7	664.4	332.7		646.4	T	1123.5	562.3	1106.5	1105.5	10
8	779.4	390.2		761.4	D	1022.5	511.7	1005.4	1004.5	9
9	939.4	470.2		921.4	C+57	907.4	454.2	890.4	889.4	8
10	1052.5	526.8		1034.5	I	747.4	374.2	730.4	729.4	7
11	1109.5	555.3		1091.5	G	634.3	317.7	617.3	616.3	6
12	1210.6	605.8		1192.6	T	577.3		560.3	559.3	5
13	1309.7	655.3		1291.6	V	476.3		459.2	458.2	4
14	1424.7	712.8		1406.7	D	377.2		360.2	359.2	3
15	1511.7	756.4		1493.7	S	262.1		245.1	244.1	2
16	1685.8	843.4	1668.8	1667.8	R	175.1		158.1		1

Gene symbol: Chordc1

Protein name: 18-day embryo whole body cDNA, RIKEN full-length enriched library, clone:1110001O09 product:cysteine and histidine-rich domain

Protein accession numbers: IPI00134017

Peptide sequence: (K)TSDFNTFLAQEGCTR(G)

Exclusive (unique to this protein): TRUE

SEQUEST XCorr score: 2.18 SEQUEST DCn score: 0.675

Fix modifications: C13: Carbamidomethyl (+57.02)Variable modifications: None

charge: 2 Observed m/z: 873.8902

Actual minus calculated peptide mass (AMU): -0.003296

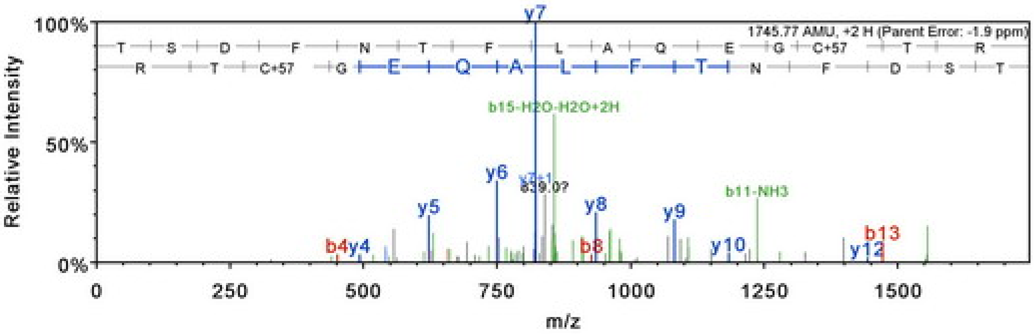

B	B Ions	B+2H	B-NH3	B-H2O	AA	Y Ions	Y+2H	Y-NH3	Y-H2O	Y
1	102.1			84.0	T	1746.8	873.9	1729.8	1728.8	15
2	189.1			171.1	S	1645.7	823.4	1628.7	1627.7	14
3	304.1			286.1	D	1558.7	779.9	1541.7	1540.7	13
4	451.2			433.2	F	1443.7	722.3	1426.6	1425.7	12
5	565.2		548.2	547.2	N	1296.6	648.8	1279.6	1278.6	11
6	666.3	333.6	649.3	648.3	T	1182.6	591.8	1165.5	1164.5	10
7	813.3	407.2	796.3	795.3	F	1081.5	541.3	1064.5	1063.5	9
8	926.4	463.7	909.4	908.4	L	934.4	467.7	917.4	916.4	8
9	997.5	499.2	980.4	979.5	A	821.4	411.2	804.3	803.4	7
10	1125.5	563.3	1108.5	1107.5	Q	750.3	375.7	733.3	732.3	6
11	1254.6	627.8	1237.5	1236.5	E	622.3		605.2	604.3	5
12	1311.6	656.3	1294.6	1293.6	G	493.2		476.2	475.2	4
13	1471.6	736.3	1454.6	1453.6	C+57	436.2		419.2	418.2	3
14	1572.7	786.8	1555.6	1554.7	T	276.2		259.1	258.2	2
15	1746.8	873.9	1729.8	1728.8	R	175.1		158.1		1

Gene symbol: Ckap5

Protein name: Cytoskeleton associated protein 5

Protein accession numbers: IPI00317134,IPI00337930

Peptide sequence: (R)DAAFEALGTALK(V)

Exclusive (unique to this protein): TRUE

SEQUEST XCorr score: 2.21 SEQUEST DCn score: 0.622

Fix modifications: None

Variable modifications: None

charge: 2 Observed m/z: 603.8196

Actual minus calculated peptide mass (AMU): -0.005859

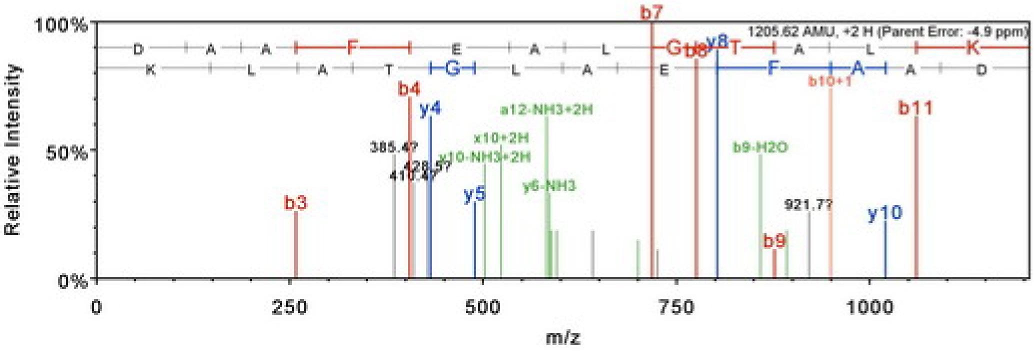

B	B Ions	B+2H	B-NH3	B-H2O	AA	Y Ions	Y+2H	Y-NH3	Y-H2O	Y
1	116.0			98.0	D	1206.6	603.8	1189.6	1188.6	12
2	187.1			169.1	A	1091.6	546.3	1074.6	1073.6	11
3	258.1			240.1	A	1020.6	510.8	1003.6	1002.6	10
4	405.2			387.2	F	949.5	475.3	932.5	931.5	9
5	534.2			516.2	E	802.5	401.7	785.4	784.5	8
6	605.3	303.1		587.3	A	673.4	337.2	656.4	655.4	7
7	718.3	359.7		700.3	L	602.4	301.7	585.4	584.4	6
8	775.4	388.2		757.4	G	489.3		472.3	471.3	5
9	876.4	438.7		858.4	T	432.3		415.3	414.3	4
10	947.5	474.2		929.4	A	331.2		314.2		3
11	1060.5	530.8		1042.5	L	260.2		243.2		2
12	1206.6	603.8	1189.6	1188.6	K	147.1		130.1		1

Gene symbol: Clta

Protein name: clathrin, light polypeptide (Lca) isoform d

Protein accession numbers: IPI00648658,IPI00828932

Peptide sequence: (R)AAEEAFVNDIDESSPGTEWER(V)

Exclusive (unique to this protein): TRUE

SEQUEST XCorr score: 3.64 SEQUEST DCn score: 0.663

Fix modifications: None

Variable modifications: None

charge: 2 Observed m/z: 1176.5206

Actual minus calculated peptide mass (AMU): 0.006592

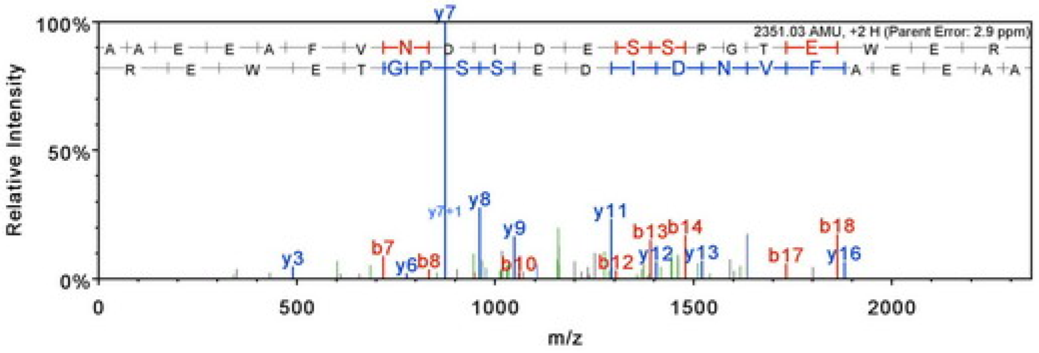

B	B Ions	B+2H	B-NH3	B-H2O	AA	Y Ions	Y+2H	Y-NH3	Y-H2O	Y
1	72.0				A	2352.0	1176.5	2335.0	2334.0	21
2	143.1				A	2281.0	1141.0	2264.0	2263.0	20
3	272.1			254.1	E	2210.0	1105.5	2192.9	2191.9	19
4	401.2			383.2	E	2080.9	1041.0	2063.9	2062.9	18
5	472.2			454.2	A	1951.9	976.4	1934.8	1933.9	17
6	619.3	310.1		601.3	F	1880.8	940.9	1863.8	1862.8	16
7	718.3	359.7		700.3	V	1733.8	867.4	1716.7	1715.8	15
8	832.4	416.7	815.4	814.4	N	1634.7	817.9	1617.7	1616.7	14
9	947.4	474.2	930.4	929.4	D	1520.7	760.8	1503.6	1502.6	13
10	1060.5	530.8	1043.5	1042.5	I	1405.6	703.3	1388.6	1387.6	12
11	1175.5	588.3	1158.5	1157.5	D	1292.5	646.8	1275.5	1274.5	11
12	1304.6	652.8	1287.5	1286.5	E	1177.5	589.3	1160.5	1159.5	10
13	1391.6	696.3	1374.6	1373.6	S	1048.5	524.7	1031.4	1030.5	9
14	1478.6	739.8	1461.6	1460.6	S	961.4	481.2	944.4	943.4	8
15	1575.7	788.3	1558.7	1557.7	P	874.4	437.7	857.4	856.4	7
16	1632.7	816.9	1615.7	1614.7	G	777.4	389.2	760.3	759.3	6
17	1733.8	867.4	1716.7	1715.7	T	720.3		703.3	702.3	5
18	1862.8	931.9	1845.8	1844.8	E	619.3		602.3	601.3	4
19	2048.9	1024.9	2031.9	2030.9	W	490.2		473.2	472.2	3
20	2177.9	1089.5	2160.9	2159.9	E	304.2		287.1	286.2	2
21	2352.0	1176.5	2335.0	2334.0	R	175.1		158.1		1

Gene symbol: Cnbp

Protein name: Isoform 2 of Cellular nucleic acid-binding protein

Protein accession numbers: IPI00137229,IPI00330767,IPI00454151

Peptide sequence: (R)GFQFVSSSLPDICYR(C)

Exclusive (unique to this protein): TRUE

SEQUEST XCorr score: 2.01 SEQUEST DCn score: 0.584

Fix modifications: C13: Carbamidomethyl (+57.02)Variable modifications: None

charge: 2 Observed m/z: 888.4268

Actual minus calculated peptide mass (AMU): 0.003052

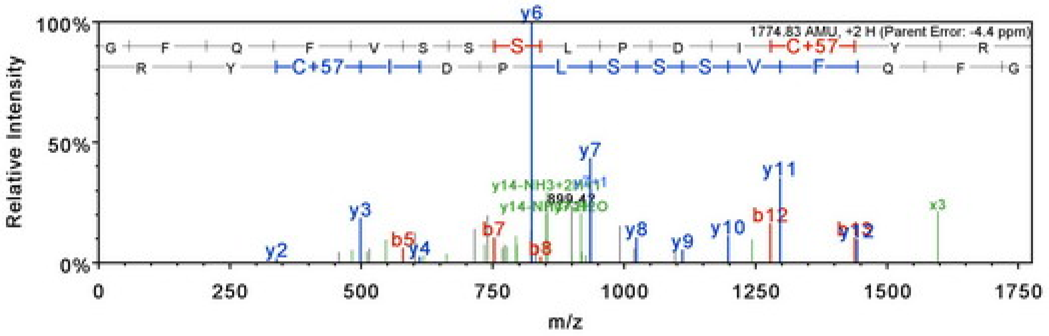

B	B Ions	B+2H	B-NH3	B-H2O	AA	Y Ions	Y+2H	Y-NH3	Y-H2O	Y
1	58.0				G	1775.8	888.4	1758.8	1757.8	15
2	205.1				F	1718.8	859.9	1701.8	1700.8	14
3	333.2		316.1		Q	1571.8	786.4	1554.7	1553.7	13
4	480.2		463.2		F	1443.7	722.4	1426.7	1425.7	12
5	579.3		562.3		V	1296.6	648.8	1279.6	1278.6	11
6	666.3	333.7	649.3	648.3	S	1197.6	599.3	1180.5	1179.5	10
7	753.4	377.2	736.3	735.4	S	1110.5	555.8	1093.5	1092.5	9
8	840.4	420.7	823.4	822.4	S	1023.5	512.3	1006.5	1005.5	8
9	953.5	477.2	936.5	935.5	L	936.5	468.7	919.4	918.5	7
10	1050.5	525.8	1033.5	1032.5	P	823.4	412.2	806.4	805.4	6
11	1165.5	583.3	1148.5	1147.5	D	726.3		709.3	708.3	5
12	1278.6	639.8	1261.6	1260.6	I	611.3		594.3		4
13	1438.7	719.8	1421.6	1420.7	C+57	498.2		481.2		3
14	1601.7	801.4	1584.7	1583.7	Y	338.2		321.2		2
15	1775.8	888.4	1758.8	1757.8	R	175.1		158.1		1

Gene symbol: Cops2

Protein name: Isoform 1 of COP9 signalosome complex subunit 2

Protein accession numbers: IPI00120513,IPI00480307,IPI00756831

Peptide sequence: (R)IDQVNQLLELDHQK(R)

Exclusive (unique to this protein): TRUE

SEQUEST XCorr score: 2.06 SEQUEST DCn score: 0.465

Fix modifications: None

Variable modifications: None

charge: 2 Observed m/z: 846.9481

Actual minus calculated peptide mass (AMU): -0.0039054

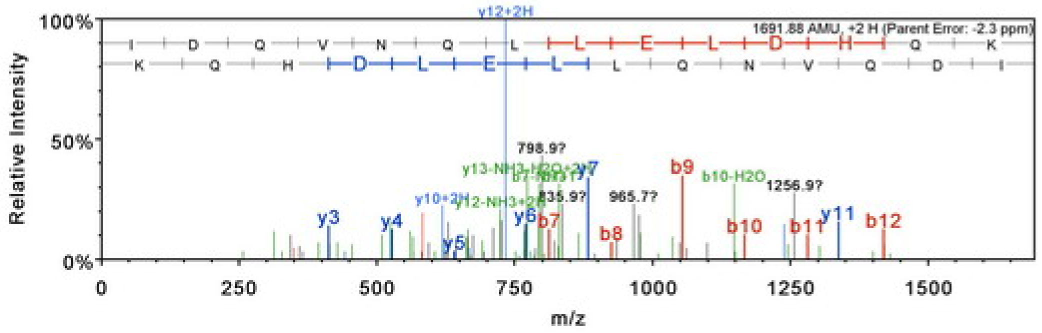

B	B Ions	B+2H	B-NH3	B-H2O	AA	Y Ions	Y+2H	Y-NH3	Y-H2O	Y
1	114.1				I	1692.9	847.0	1675.9	1674.9	14
2	229.1			211.1	D	1579.8	790.4	1562.8	1561.8	13
3	357.2		340.2	339.2	Q	1464.8	732.9	1447.8	1446.8	12
4	456.3		439.2	438.2	V	1336.7	668.9	1319.7	1318.7	11
5	570.3		553.3	552.3	N	1237.7	619.3	1220.6	1219.6	10
6	698.4	349.7	681.3	680.3	Q	1123.6	562.3	1106.6	1105.6	9
7	811.4	406.2	794.4	793.4	L	995.6	498.3	978.5	977.5	8
8	924.5	462.8	907.5	906.5	L	882.5	441.7	865.4	864.5	7
9	1053.6	527.3	1036.5	1035.5	E	769.4	385.2	752.4	751.4	6
10	1166.6	583.8	1149.6	1148.6	L	640.3	320.7	623.3	622.3	5
11	1281.7	641.3	1264.6	1263.7	D	527.3	264.1	510.2	509.3	4
12	1418.7	709.9	1401.7	1400.7	H	412.2	206.6	395.2		3
13	1546.8	773.9	1529.8	1528.8	Q	275.2		258.1		2
14	1692.9	847.0	1675.9	1674.9	K	147.1		130.1		1

Gene symbol: Cops3

Protein name: COP9 signalosome complex subunit 3

Protein accession numbers: IPI00131870,IPI00650021

Peptide sequence: (R)YATDTFAGLCHQLTNALVER(K)

Exclusive (unique to this protein): TRUE

SEQUEST XCorr score: 2.03 SEQUEST DCn score: 0.648

Fix modifications: C10: Carbamidomethyl (+57.02)Variable modifications: None

charge: 2 Observed m/z: 1140.556

Actual minus calculated peptide mass (AMU): -0.00415

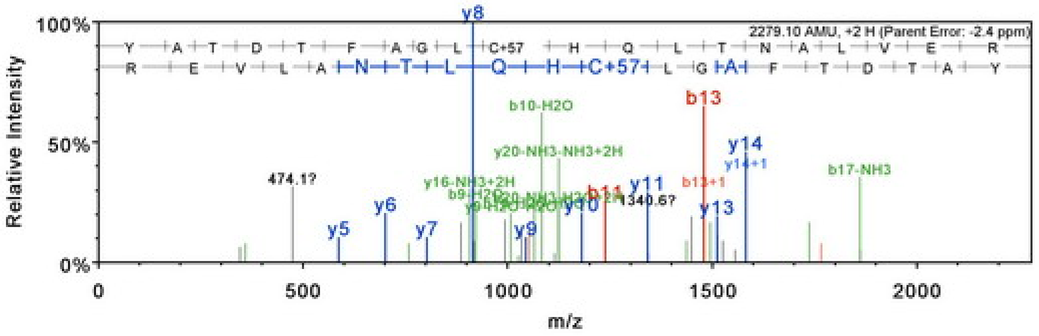

B	B Ions	B+2H	B-NH3	B-H2O	AA	Y Ions	Y+2H	Y-NH3	Y-H2O	Y
1	164.1				Y	2280.1	1140.6	2263.1	2262.1	20
2	235.1				A	2117.0	1059.0	2100.0	2099.0	19
3	336.2			318.2	T	2046.0	1023.5	2029.0	2028.0	18
4	451.2			433.2	D	1945.0	973.0	1927.9	1927.0	17
5	552.2			534.2	T	1829.9	915.5	1812.9	1811.9	16
6	699.3	350.2		681.3	F	1728.9	865.0	1711.9	1710.9	15
7	770.3	385.7		752.3	A	1581.8	791.4	1564.8	1563.8	14
8	827.4	414.2		809.4	G	1510.8	755.9	1493.8	1492.8	13
9	940.4	470.7		922.4	L	1453.8	727.4	1436.7	1435.8	12
10	1100.5	550.7		1082.5	C+57	1340.7	670.8	1323.7	1322.7	11
11	1237.5	619.3		1219.5	H	1180.6	590.8	1163.6	1162.6	10
12	1365.6	683.3	1348.6	1347.6	Q	1043.6	522.3	1026.6	1025.6	9
13	1478.7	739.8	1461.7	1460.7	L	915.5	458.3	898.5	897.5	8
14	1579.7	790.4	1562.7	1561.7	T	802.4	401.7	785.4	784.4	7
15	1693.8	847.4	1676.7	1675.8	N	701.4	351.2	684.4	683.4	6
16	1764.8	882.9	1747.8	1746.8	A	587.4		570.3	569.3	5
17	1877.9	939.5	1860.9	1859.9	L	516.3		499.3	498.3	4
18	1977.0	989.0	1959.9	1958.9	V	403.2		386.2	385.2	3
19	2106.0	1053.5	2089.0	2088.0	E	304.2		287.1	286.2	2
20	2280.1	1140.6	2263.1	2262.1	R	175.1		158.1		1

Gene symbol: Cops5

Protein name: COP9 signalosome complex subunit 5

Protein accession numbers: IPI00135087

Peptide sequence: (K)GYKPPDEGPSEYQTIPLNK(I)

Exclusive (unique to this protein): TRUE

SEQUEST XCorr score: 2.01 SEQUEST DCn score: 0.601

Fix modifications: None

Variable modifications: None

charge: 2 Observed m/z: 1067.0266

Actual minus calculated peptide mass (AMU): -0.005371

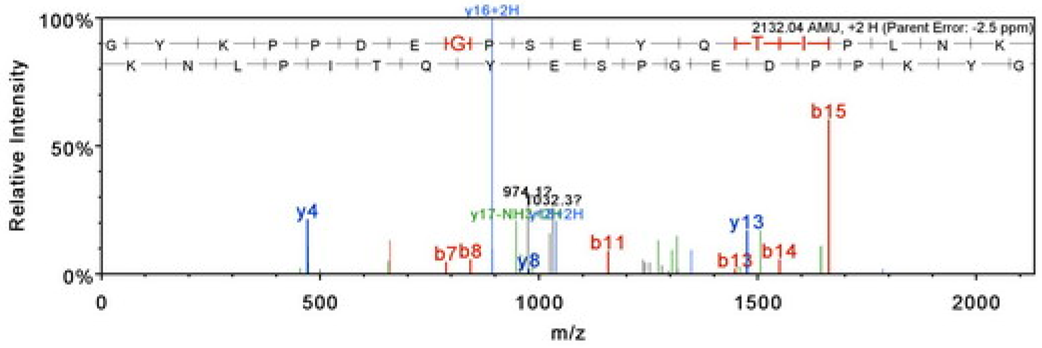

B	B Ions	B+2H	B-NH3	B-H2O	AA	Y Ions	Y+2H	Y-NH3	Y-H2O	Y
1	58.0				G	2133.1	1067.0	2116.0	2115.0	19
2	221.1				Y	2076.0	1038.5	2059.0	2058.0	18
3	349.2	175.1	332.2		K	1913.0	957.0	1895.9	1895.0	17
4	446.2	223.6	429.2		P	1784.9	892.9	1767.8	1766.9	16
5	543.3	272.1	526.3		P	1687.8	844.4	1670.8	1669.8	15
6	658.3	329.7	641.3	640.3	D	1590.8	795.9	1573.7	1572.8	14
7	787.4	394.2	770.3	769.4	E	1475.7	738.4	1458.7	1457.7	13
8	844.4	422.7	827.4	826.4	G	1346.7	673.9	1329.7	1328.7	12
9	941.4	471.2	924.4	923.4	P	1289.7	645.3	1272.7	1271.7	11
10	1028.5	514.7	1011.4	1010.5	S	1192.6	596.8	1175.6	1174.6	10
11	1157.5	579.3	1140.5	1139.5	E	1105.6	553.3	1088.6	1087.6	9
12	1320.6	660.8	1303.5	1302.6	Y	976.6	488.8	959.5	958.5	8
13	1448.6	724.8	1431.6	1430.6	Q	813.5	407.3	796.5	795.5	7
14	1549.7	775.3	1532.7	1531.7	T	685.4	343.2	668.4	667.4	6
15	1662.8	831.9	1645.7	1644.8	I	584.4		567.4		5
16	1759.8	880.4	1742.8	1741.8	P	471.3		454.3		4
17	1872.9	937.0	1855.9	1854.9	L	374.2		357.2		3
18	1987.0	994.0	1969.9	1968.9	N	261.2		244.1		2
19	2133.1	1067.0	2116.0	2115.0	K	147.1		130.1		1

Gene symbol: Cops6

Protein name: COP9 signalosome complex subunit 6

Protein accession numbers: IPI00131873,IPI00830393,IPI00830412

Peptide sequence: (K)ASEAGEVPFNHEILR(E)

Exclusive (unique to this protein): TRUE

SEQUEST XCorr score: 2.13 SEQUEST DCn score: 0.604

Fix modifications: None

Variable modifications: None

charge: 2 Observed m/z: 834.9174

Actual minus calculated peptide mass (AMU): -0.007813

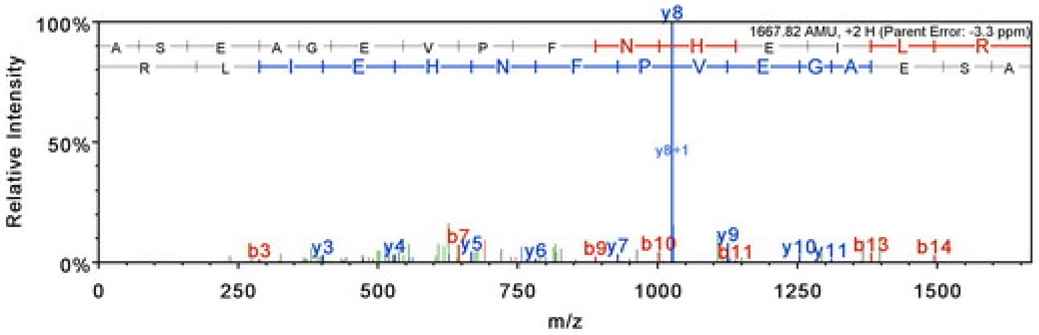

B	B Ions	B+2H	B-NH3	B-H2O	AA	Y Ions	Y+2H	Y-NH3	Y-H2O	Y
1	72.0				A	1668.8	834.9	1651.8	1650.8	15
2	159.1			141.1	S	1597.8	799.4	1580.8	1579.8	14
3	288.1			270.1	E	1510.8	755.9	1493.7	1492.8	13
4	359.2			341.2	A	1381.7	691.4	1364.7	1363.7	12
5	416.2			398.2	G	1310.7	655.9	1293.7	1292.7	11
6	545.2	273.1		527.2	E	1253.7	627.3	1236.6	1235.7	10
7	644.3	322.7		626.3	V	1124.6	562.8	1107.6	1106.6	9
8	741.3	371.2		723.3	P	1025.5	513.3	1008.5	1007.5	8
9	888.4	444.7		870.4	F	928.5	464.8	911.5	910.5	7
10	1002.5	501.7	985.4	984.4	N	781.4	391.2	764.4	763.4	6
11	1139.5	570.3	1122.5	1121.5	H	667.4	334.2	650.4	649.4	5
12	1268.5	634.8	1251.5	1250.5	E	530.3		513.3	512.3	4
13	1381.6	691.3	1364.6	1363.6	I	401.3		384.3		3
14	1494.7	747.9	1477.7	1476.7	L	288.2		271.2		2
15	1668.8	834.9	1651.8	1650.8	R	175.1		158.1		1

Gene symbol: Copz1

Protein name: Coatomer subunit zeta-1

Protein accession numbers: IPI00139637,IPI00761970

Peptide sequence: (R)GEDVPLTEQTVSQVLQSAK(E)

Exclusive (unique to this protein): TRUE

SEQUEST XCorr score: 2.36 SEQUEST DCn score: 0.594

Fix modifications: None

Variable modifications: None

charge: 2 Observed m/z: 1015.0287

Actual minus calculated peptide mass (AMU): 0.00415

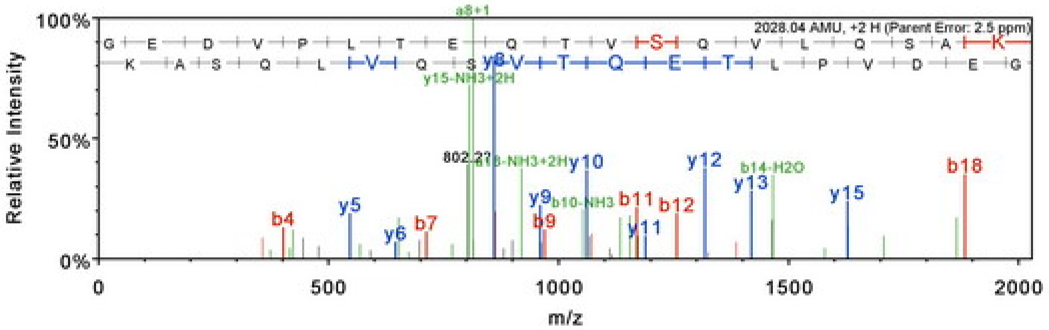

B	B Ions	B+2H	B-NH3	B-H2O	AA	Y Ions	Y+2H	Y-NH3	Y-H2O	Y
1	58.0				G	2029.1	1015.0	2012.0	2011.0	19
2	187.1			169.1	E	1972.0	986.5	1955.0	1954.0	18
3	302.1			284.1	D	1843.0	922.0	1826.0	1825.0	17
4	401.2			383.2	V	1728.0	864.5	1710.9	1709.9	16
5	498.2			480.2	P	1628.9	815.0	1611.9	1610.9	15
6	611.3	306.2		593.3	L	1531.8	766.4	1514.8	1513.8	14
7	712.4	356.7		694.3	T	1418.8	709.9	1401.7	1400.7	13
8	841.4	421.2		823.4	E	1317.7	659.4	1300.7	1299.7	12
9	969.5	485.2	952.4	951.4	Q	1188.7	594.8	1171.6	1170.7	11
10	1070.5	535.8	1053.5	1052.5	T	1060.6	530.8	1043.6	1042.6	10
11	1169.6	585.3	1152.5	1151.6	V	959.6	480.3	942.5	941.5	9
12	1256.6	628.8	1239.6	1238.6	S	860.5	430.8	843.5	842.5	8
13	1384.7	692.8	1367.6	1366.7	Q	773.5	387.2	756.4	755.4	7
14	1483.7	742.4	1466.7	1465.7	V	645.4	323.2	628.4	627.4	6
15	1596.8	798.9	1579.8	1578.8	L	546.3		529.3	528.3	5
16	1724.9	862.9	1707.8	1706.9	Q	433.2		416.2	415.2	4
17	1811.9	906.5	1794.9	1793.9	S	305.2		288.2	287.2	3
18	1882.9	942.0	1865.9	1864.9	A	218.2		201.1		2
19	2029.1	1015.0	2012.0	2011.0	K	147.1		130.1		1

Gene symbol: Cpne8

Protein name: copine VIII isoform 2

Protein accession numbers: IPI00118969,IPI00120173,IPI00461579

Peptide sequence: (R)DTFSKSDPICVLYTQAVGNK(E)

Exclusive (unique to this protein): TRUE

SEQUEST XCorr score: 2.04 SEQUEST DCn score: 0.409

Fix modifications: C10: Carbamidomethyl (+57.02)Variable modifications: None

charge: 2 Observed m/z: 1122.0526

Actual minus calculated peptide mass (AMU): -0.004395

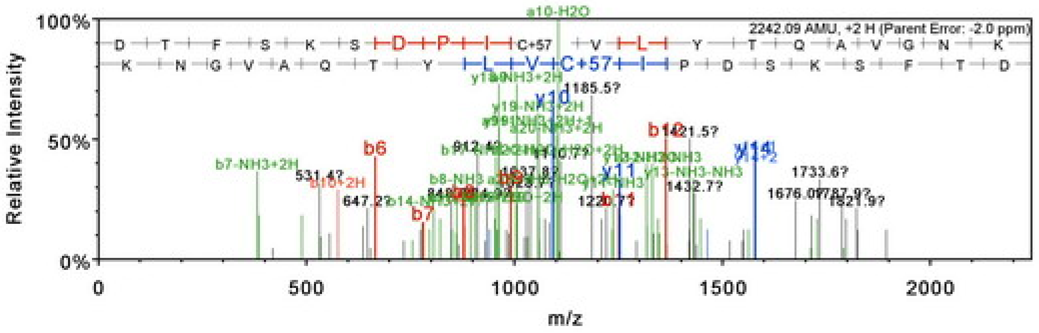

B	B Ions	B+2H	B-NH3	B-H2O	AA	Y Ions	Y+2H	Y-NH3	Y-H2O	Y
1	116.0			98.0	D	2243.1	1122.0	2226.1	2225.1	20
2	217.1			199.1	T	2128.1	1064.5	2111.1	2110.1	19
3	364.2			346.1	F	2027.0	1014.0	2010.0	2009.0	18
4	451.2			433.2	S	1880.0	940.5	1862.9	1862.0	17
5	579.3	290.1	562.3	561.3	K	1792.9	897.0	1775.9	1774.9	16
6	666.3	333.7	649.3	648.3	S	1664.8	832.9	1647.8	1646.8	15
7	781.3	391.2	764.3	763.3	D	1577.8	789.4	1560.8	1559.8	14
8	878.4	439.7	861.4	860.4	P	1462.8	731.9	1445.8	1444.8	13
9	991.5	496.2	974.5	973.5	I	1365.7	683.4	1348.7	1347.7	12
10	1151.5	576.3	1134.5	1133.5	C+57	1252.6	626.8	1235.6	1234.6	11
11	1250.6	625.8	1233.5	1232.6	V	1092.6	546.8	1075.6	1074.6	10
12	1363.7	682.3	1346.6	1345.7	L	993.5	497.3	976.5	975.5	9
13	1526.7	763.9	1509.7	1508.7	Y	880.5	440.7	863.4	862.4	8
14	1627.8	814.4	1610.7	1609.8	T	717.4	359.2	700.4	699.4	7
15	1755.8	878.4	1738.8	1737.8	Q	616.3	308.7	599.3		6
16	1826.9	913.9	1809.8	1808.9	A	488.3		471.3		5
17	1925.9	963.5	1908.9	1907.9	V	417.3		400.2		4
18	1983.0	992.0	1965.9	1964.9	G	318.2		301.2		3
19	2097.0	1049.0	2080.0	2079.0	N	261.2		244.1		2
20	2243.1	1122.0	2226.1	2225.1	K	147.1		130.1		1

Gene symbol: Crym

Protein name: Mu-crystallin homolog

Protein accession numbers: IPI00120030

Peptide sequence: (R)APAFLSAEEVQDHLR(S)

Exclusive (unique to this protein): TRUE

SEQUEST XCorr score: 3.01 SEQUEST DCn score: 0.625

Fix modifications: None

Variable modifications: None

charge: 2 Observed m/z: 841.926

Actual minus calculated peptide mass (AMU): -0.006104

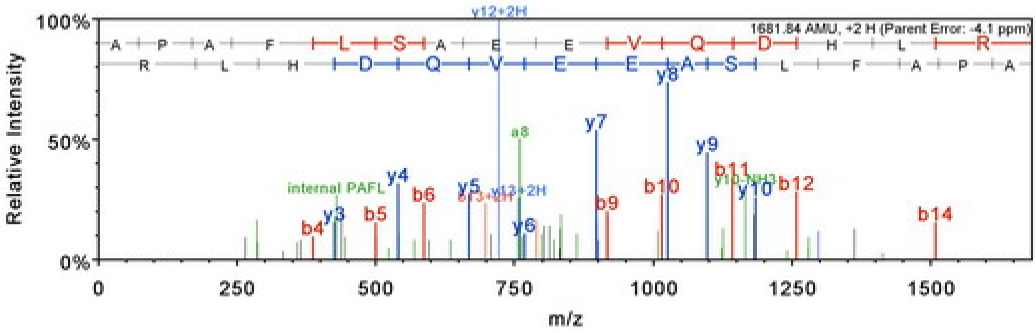

B	B Ions	B+2H	B-NH3	B-H2O	AA	Y Ions	Y+2H	Y-NH3	Y-H2O	Y
1	72.0				A	1682.9	841.9	1665.8	1664.8	15
2	169.1				P	1611.8	806.4	1594.8	1593.8	14
3	240.1				A	1514.8	757.9	1497.7	1496.8	13
4	387.2				F	1443.7	722.4	1426.7	1425.7	12
5	500.3				L	1296.7	648.8	1279.6	1278.6	11
6	587.3	294.2		569.3	S	1183.6	592.3	1166.5	1165.6	10
7	658.4	329.7		640.4	A	1096.5	548.8	1079.5	1078.5	9
8	787.4	394.2		769.4	E	1025.5	513.3	1008.5	1007.5	8
9	916.4	458.7		898.4	E	896.5	448.7	879.4	878.5	7
10	1015.5	508.3		997.5	V	767.4	384.2	750.4	749.4	6
11	1143.6	572.3	1126.5	1125.6	Q	668.4	334.7	651.3	650.3	5
12	1258.6	629.8	1241.6	1240.6	D	540.3	270.6	523.3	522.3	4
13	1395.7	698.3	1378.6	1377.6	H	425.3	213.1	408.2		3
14	1508.7	754.9	1491.7	1490.7	L	288.2		271.2		2
15	1682.9	841.9	1665.8	1664.8	R	175.1		158.1		1

Gene symbol: Cse1l

Protein name: Exportin-2

Protein accession numbers: IPI00112414

Peptide sequence: (R)AADEEAFEDNSEEYIR(R)

Exclusive (unique to this protein): TRUE

SEQUEST XCorr score: 2.93 SEQUEST DCn score: 0.832

Fix modifications: None

Variable modifications: None

charge: 2 Observed m/z: 944.3967

Actual minus calculated peptide mass (AMU): -0.003052

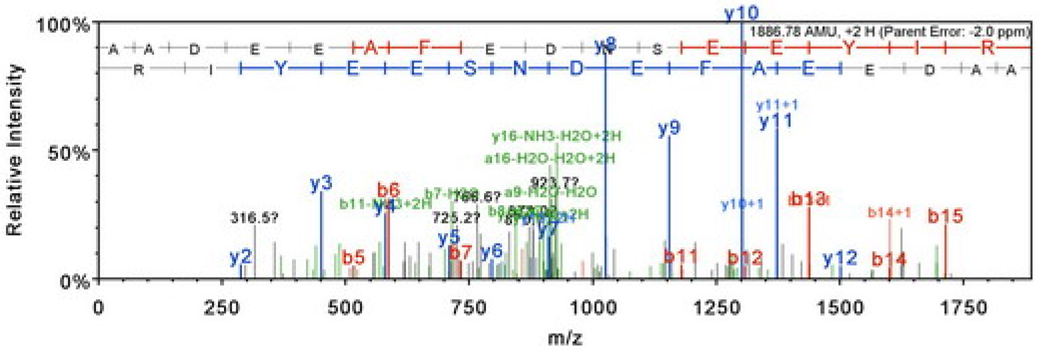

B	B Ions	B+2H	B-NH3	B-H2O	AA	Y Ions	Y+2H	Y-NH3	Y-H2O	Y
1	72.0				A	1887.8	944.4	1870.8	1869.8	16
2	143.1				A	1816.8	908.9	1799.7	1798.7	15
3	258.1			240.1	D	1745.7	873.4	1728.7	1727.7	14
4	387.2			369.1	E	1630.7	815.9	1613.7	1612.7	13
5	516.2			498.2	E	1501.6	751.3	1484.6	1483.6	12
6	587.2	294.1		569.2	A	1372.6	686.8	1355.6	1354.6	11
7	734.3	367.7		716.3	F	1301.6	651.3	1284.5	1283.5	10
8	863.3	432.2		845.3	E	1154.5	577.8	1137.5	1136.5	9
9	978.4	489.7		960.4	D	1025.5	513.2	1008.4	1007.4	8
10	1092.4	546.7	1075.4	1074.4	N	910.4	455.7	893.4	892.4	7
11	1179.4	590.2	1162.4	1161.4	S	796.4	398.7	779.4	778.4	6
12	1308.5	654.8	1291.5	1290.5	E	709.4		692.3	691.3	5
13	1437.5	719.3	1420.5	1419.5	E	580.3		563.3	562.3	4
14	1600.6	800.8	1583.6	1582.6	Y	451.3		434.2		3
15	1713.7	857.3	1696.7	1695.7	I	288.2		271.2		2
16	1887.8	944.4	1870.8	1869.8	R	175.1		158.1		1

Gene symbol: Cul1

Protein name: Cullin-1

Protein accession numbers: IPI00124047

Peptide sequence: (K)HQQLLGEVLTQLSSR(F)

Exclusive (unique to this protein): TRUE

SEQUEST XCorr score: 2.16 SEQUEST DCn score: 0.704

Fix modifications: None

Variable modifications: None

charge: 2 Observed m/z: 854.9686

Actual minus calculated peptide mass (AMU): -0.005371

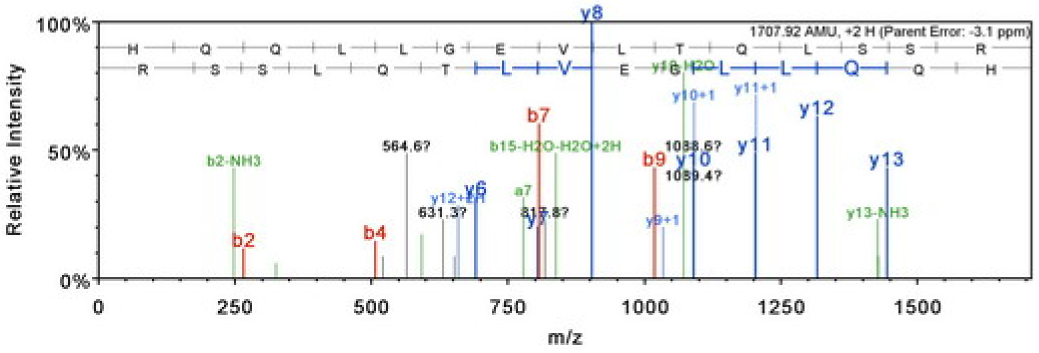

B	B Ions	B+2H	B-NH3	B-H2O	AA	Y Ions	Y+2H	Y-NH3	Y-H2O	Y
1	138.1	69.5			H	1708.9	855.0	1691.9	1690.9	15
2	266.1	133.6	249.1		Q	1571.9	786.4	1554.9	1553.9	14
3	394.2	197.6	377.2		Q	1443.8	722.4	1426.8	1425.8	13
4	507.3	254.1	490.2		L	1315.8	658.4	1298.7	1297.8	12
5	620.4	310.7	603.3		L	1202.7	601.8	1185.7	1184.7	11
6	677.4	339.2	660.4		G	1089.6	545.3	1072.6	1071.6	10
7	806.4	403.7	789.4	788.4	E	1032.6	516.8	1015.5	1014.6	9
8	905.5	453.3	888.5	887.5	V	903.5	452.3	886.5	885.5	8
9	1018.6	509.8	1001.5	1000.6	L	804.5	402.7	787.4	786.5	7
10	1119.6	560.3	1102.6	1101.6	T	691.4	346.2	674.4	673.4	6
11	1247.7	624.3	1230.7	1229.7	Q	590.3		573.3	572.3	5
12	1360.8	680.9	1343.7	1342.8	L	462.3		445.2	444.3	4
13	1447.8	724.4	1430.8	1429.8	S	349.2		332.2	331.2	3
14	1534.8	767.9	1517.8	1516.8	S	262.1		245.1	244.1	2
15	1708.9	855.0	1691.9	1690.9	R	175.1		158.1		1

Gene symbol: Cycs;LOC672195

Protein name: Cytochrome c, somatic

Protein accession numbers: IPI00222419,IPI00265239

Peptide sequence: (K)TGPNLHGLFGR(K)

Exclusive (unique to this protein): TRUE

SEQUEST XCorr score: 2.34 SEQUEST DCn score: 0.7

Fix modifications: None

Variable modifications: None

charge: 2 Observed m/z: 584.814

Actual minus calculated peptide mass (AMU): -0.002686

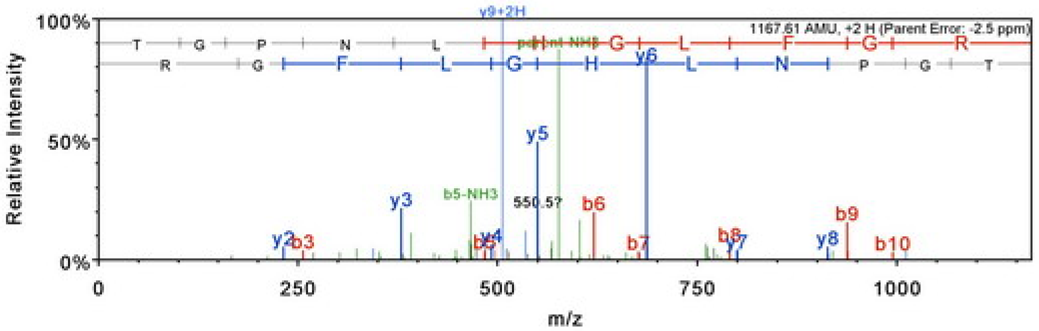

B	B Ions	B+2H	B-NH3	B-H2O	AA	Y Ions	Y+2H	Y-NH3	Y-H2O	Y
1	102.1			84.0	T	1168.6	584.8	1151.6	1150.6	11
2	159.1			141.1	G	1067.6	534.3	1050.5		10
3	256.1			238.1	P	1010.6	505.8	993.5		9
4	370.2		353.2	352.2	N	913.5	457.3	896.5		8
5	483.3		466.2	465.3	L	799.5	400.2	782.4		7
6	620.3	310.7	603.3	602.3	H	686.4	343.7	669.4		6
7	677.3	339.2	660.3	659.3	G	549.3		532.3		5
8	790.4	395.7	773.4	772.4	L	492.3		475.3		4
9	937.5	469.3	920.5	919.5	F	379.2		362.2		3
10	994.5	497.8	977.5	976.5	G	232.1		215.1		2
11	1168.6	584.8	1151.6	1150.6	R	175.1		158.1		1

Gene symbol: D10Jhu81e
Protein name: ES1 protein homolog, mitochondrial precursor
Protein accession numbers: IPI00133284
Peptide sequence: (K)ITSLAQLNAANHDAAIFPGGFGAAK(N)
Exclusive (unique to this protein): TRUE
SEQUEST XCorr score: 2.03 SEQUEST DCn score: 0.662
Fix modifications: None
Variable modifications: None
charge: 2 Observed m/z: 1228.1467
Actual minus calculated peptide mass (AMU): 0.011954

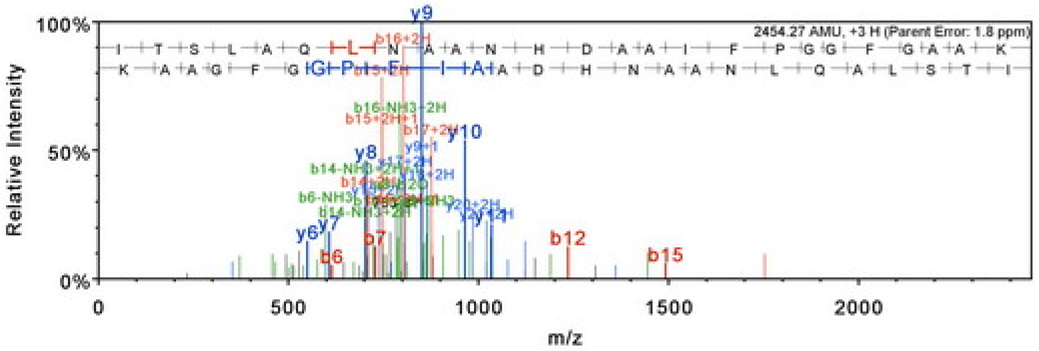

B	B Ions	B+2H	B-NH3	B-H2O	AA	Y Ions	Y+2H	Y-NH3	Y-H2O	Y
1	114.1				I	2455.3	1228.1	2438.3	2437.3	25
2	215.1			197.1	T	2342.2	1171.6	2325.2	2324.2	24
3	302.2			284.2	S	2241.1	1121.1	2224.1	2223.1	23
4	415.3			397.3	L	2154.1	1077.6	2137.1	2136.1	22
5	486.3			468.3	A	2041.0	1021.0	2024.0	2023.0	21
6	614.4	307.7	597.3	596.3	Q	1970.0	985.5	1953.0	1952.0	20
7	727.4	364.2	710.4	709.4	L	1841.9	921.5	1824.9	1823.9	19
8	841.5	421.2	824.5	823.5	N	1728.9	864.9	1711.8	1710.8	18
9	912.5	456.8	895.5	894.5	A	1614.8	807.9	1597.8	1596.8	17
10	983.6	492.3	966.5	965.5	A	1543.8	772.4	1526.7	1525.8	16
11	1097.6	549.3	1080.6	1079.6	N	1472.7	736.9	1455.7	1454.7	15
12	1234.7	617.8	1217.6	1216.6	H	1358.7	679.9	1341.7	1340.7	14
13	1349.7	675.3	1332.7	1331.7	D	1221.6	611.3	1204.6	1203.6	13
14	1420.7	710.9	1403.7	1402.7	A	1106.6	553.8	1089.6		12
15	1491.8	746.4	1474.7	1473.8	A	1035.6	518.3	1018.5		11
16	1604.8	802.9	1587.8	1586.8	I	964.5	482.8	947.5		10
17	1751.9	876.5	1734.9	1733.9	F	851.4	426.2	834.4		9
18	1849.0	925.0	1831.9	1831.0	P	704.4	352.7	687.4		8
19	1906.0	953.5	1889.0	1888.0	G	607.3	304.2	590.3		7
20	1963.0	982.0	1946.0	1945.0	G	550.3	275.7	533.3		6
21	2110.1	1055.5	2093.1	2092.1	F	493.3		476.3		5
22	2167.1	1084.0	2150.1	2149.1	G	346.2		329.2		4
23	2238.1	1119.6	2221.1	2220.1	A	289.2		272.2		3
24	2309.2	1155.1	2292.1	2291.2	A	218.2		201.1		2
25	2455.3	1228.1	2438.3	2437.3	K	147.1		130.1		1

Gene symbol: Dctn2

Protein name: Dynactin subunit 2

Protein accession numbers: IPI00116112

Peptide sequence: (R)ENLATVEGNFASIDAR(M)

Exclusive (unique to this protein): TRUE

SEQUEST XCorr score: 2.67 SEQUEST DCn score: 0.687

Fix modifications: None

Variable modifications: None

charge: 2 Observed m/z: 853.9185

Actual minus calculated peptide mass (AMU): -0.005859

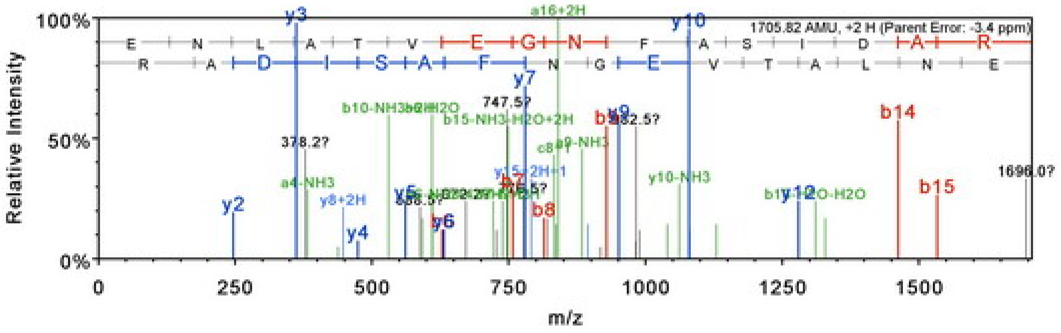

B	B Ions	B+2H	B-NH3	B-H2O	AA	Y Ions	Y+2H	Y-NH3	Y-H2O	Y
1	130.1			112.0	E	1706.8	853.9	1689.8	1688.8	16
2	244.1		227.1	226.1	N	1577.8	789.4	1560.8	1559.8	15
3	357.2		340.2	339.2	L	1463.8	732.4	1446.7	1445.7	14
4	428.2		411.2	410.2	A	1350.7	675.8	1333.6	1332.7	13
5	529.3		512.2	511.3	T	1279.6	640.3	1262.6	1261.6	12
6	628.3	314.7	611.3	610.3	V	1178.6	589.8	1161.5	1160.6	11
7	757.4	379.2	740.4	739.4	E	1079.5	540.3	1062.5	1061.5	10
8	814.4	407.7	797.4	796.4	G	950.5	475.7	933.4	932.5	9
9	928.4	464.7	911.4	910.4	N	893.5	447.2	876.4	875.4	8
10	1075.5	538.3	1058.5	1057.5	F	779.4	390.2	762.4	761.4	7
11	1146.5	573.8	1129.5	1128.5	A	632.3	316.7	615.3	614.3	6
12	1233.6	617.3	1216.5	1215.6	S	561.3		544.3	543.3	5
13	1346.7	673.8	1329.6	1328.7	I	474.3		457.2	456.3	4
14	1461.7	731.4	1444.7	1443.7	D	361.2		344.2	343.2	3
15	1532.7	766.9	1515.7	1514.7	A	246.2		229.1		2
16	1706.8	853.9	1689.8	1688.8	R	175.1		158.1		1

Gene symbol: Dcun1d1

Protein name: testis derived transcript 3

Protein accession numbers: IPI00316951,IPI00752092,IPI00785543,IPI00849604

Peptide sequence: (K)LDVATDNFFQNPELYIR(E)

Exclusive (unique to this protein): TRUE

SEQUEST XCorr score: 2.31 SEQUEST DCn score: 0.729

Fix modifications: None

Variable modifications: None

charge: 2 Observed m/z: 1028.0104

Actual minus calculated peptide mass (AMU): -0.006104

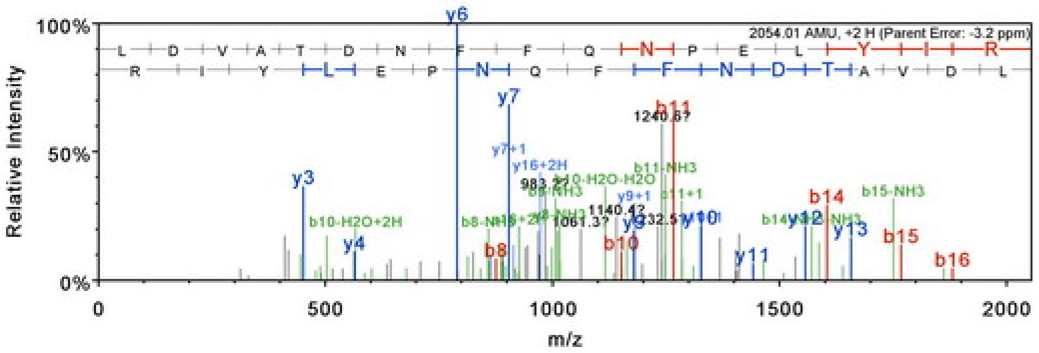

B	B Ions	B+2H	B-NH3	B-H2O	AA	Y Ions	Y+2H	Y-NH3	Y-H2O	Y
1	114.1				L	2055.0	1028.0	2038.0	2037.0	17
2	229.1			211.1	D	1941.9	971.5	1924.9	1923.9	16
3	328.2			310.2	V	1826.9	914.0	1809.9	1808.9	15
4	399.2			381.2	A	1727.8	864.4	1710.8	1709.8	14
5	500.3			482.3	T	1656.8	828.9	1639.8	1638.8	13
6	615.3	308.2		597.3	D	1555.8	778.4	1538.7	1537.7	12
7	729.3	365.2	712.3	711.3	N	1440.7	720.9	1423.7	1422.7	11
8	876.4	438.7	859.4	858.4	F	1326.7	663.9	1309.7	1308.7	10
9	1023.5	512.2	1006.5	1005.5	F	1179.6	590.3	1162.6	1161.6	9
10	1151.5	576.3	1134.5	1133.5	Q	1032.5	516.8	1015.5	1014.5	8
11	1265.6	633.3	1248.5	1247.6	N	904.5	452.8	887.5	886.5	7
12	1362.6	681.8	1345.6	1344.6	P	790.5	395.7	773.4	772.4	6
13	1491.7	746.3	1474.7	1473.7	E	693.4		676.4	675.4	5
14	1604.8	802.9	1587.7	1586.8	L	564.4		547.3		4
15	1767.8	884.4	1750.8	1749.8	Y	451.3		434.2		3
16	1880.9	941.0	1863.9	1862.9	I	288.2		271.2		2
17	2055.0	1028.0	2038.0	2037.0	R	175.1		158.1		1

Gene symbol: Dcxr

Protein name: L-xylulose reductase

Protein accession numbers: IPI00395140,IPI00649563

Peptide sequence: (R)GVPGAIVNVSSQASQR(A)

Exclusive (unique to this protein): TRUE

SEQUEST XCorr score: 2.17 SEQUEST DCn score: 0.63

Fix modifications: None

Variable modifications: None

charge: 2 Observed m/z: 785.4227

Actual minus calculated peptide mass (AMU): 0.002686

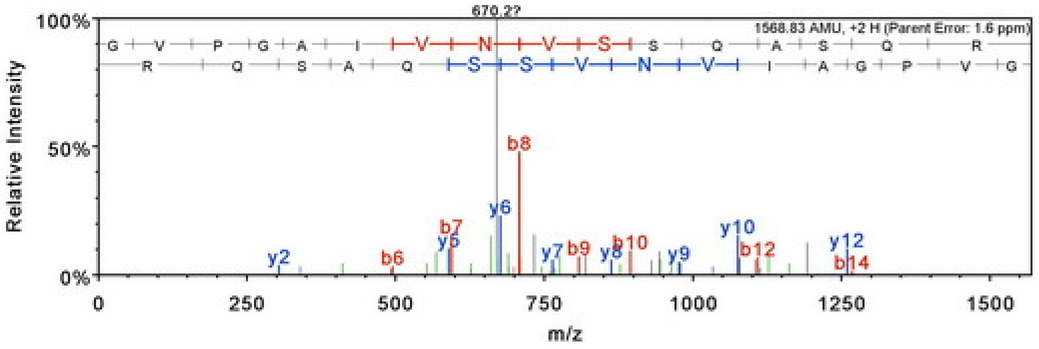

B	B Ions	B+2H	B-NH3	B-H2O	AA	Y Ions	Y+2H	Y-NH3	Y-H2O	Y
1	58.0				G	1569.8	785.4	1552.8	1551.8	16
2	157.1				V	1512.8	756.9	1495.8	1494.8	15
3	254.2				P	1413.7	707.4	1396.7	1395.7	14
4	311.2				G	1316.7	658.9	1299.7	1298.7	13
5	382.2				A	1259.7	630.3	1242.6	1241.7	12
6	495.3	248.2			I	1188.6	594.8	1171.6	1170.6	11
7	594.4	297.7			V	1075.5	538.3	1058.5	1057.5	10
8	708.4	354.7	691.4		N	976.5	488.7	959.5	958.5	9
9	807.5	404.2	790.5		V	862.4	431.7	845.4	844.4	8
10	894.5	447.8	877.5	876.5	S	763.4	382.2	746.3	745.4	7
11	981.5	491.3	964.5	963.5	S	676.3	338.7	659.3	658.3	6
12	1109.6	555.3	1092.6	1091.6	Q	589.3		572.3	571.3	5
13	1180.6	590.8	1163.6	1162.6	A	461.3		444.2	443.2	4
14	1267.7	634.3	1250.6	1249.7	S	390.2		373.2	372.2	3
15	1395.7	698.4	1378.7	1377.7	Q	303.2		286.2		2
16	1569.8	785.4	1552.8	1551.8	R	175.1		158.1		1

Gene symbol: Ddx19a

Protein name: ATP-dependent RNA helicase DDX19A

Protein accession numbers: IPI00123651

Peptide sequence: (R)SNLVDNTNQVEVLQR(D)

Exclusive (unique to this protein): TRUE

SEQUEST XCorr score: 2.17 SEQUEST DCn score: 0.479

Fix modifications: None

Variable modifications: None

charge: 2 Observed m/z: 864.9445

Actual minus calculated peptide mass (AMU): -0.006958

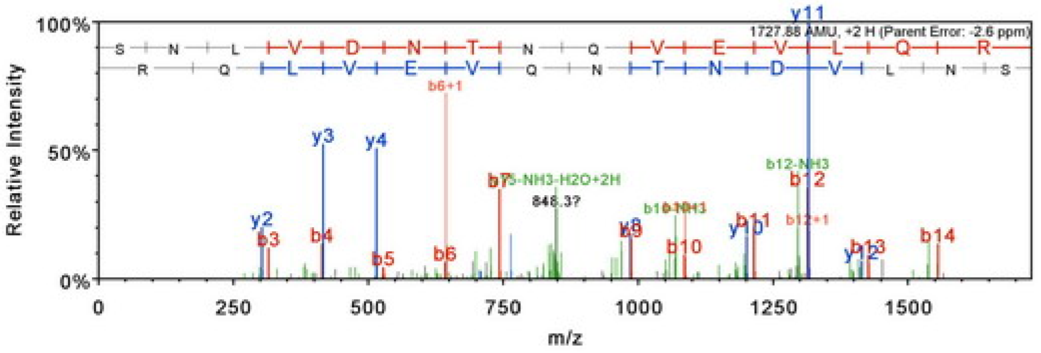

B	B Ions	B+2H	B-NH3	B-H2O	AA	Y Ions	Y+2H	Y-NH3	Y-H2O	Y
1	88.0			70.0	S	1728.9	865.0	1711.9	1710.9	15
2	202.1		185.1	184.1	N	1641.9	821.4	1624.8	1623.9	14
3	315.2		298.1	297.2	L	1527.8	764.4	1510.8	1509.8	13
4	414.2		397.2	396.2	V	1414.7	707.9	1397.7	1396.7	12
5	529.3		512.2	511.3	D	1315.7	658.3	1298.6	1297.7	11
6	643.3	322.2	626.3	625.3	N	1200.6	600.8	1183.6	1182.6	10
7	744.4	372.7	727.3	726.3	T	1086.6	543.8	1069.6	1068.6	9
8	858.4	429.7	841.4	840.4	N	985.5	493.3	968.5	967.5	8
9	986.5	493.7	969.4	968.4	Q	871.5	436.3	854.5	853.5	7
10	1085.5	543.3	1068.5	1067.5	V	743.4	372.2	726.4	725.4	6
11	1214.6	607.8	1197.5	1196.5	E	644.4		627.4	626.4	5
12	1313.6	657.3	1296.6	1295.6	V	515.3		498.3		4
13	1426.7	713.9	1409.7	1408.7	L	416.3		399.2		3
14	1554.8	777.9	1537.8	1536.8	Q	303.2		286.2		2
15	1728.9	865.0	1711.9	1710.9	R	175.1		158.1		1

Gene symbol: Dera

Protein name: Putative deoxyribose-phosphate aldolase

Protein accession numbers: IPI00130333

Peptide sequence: (R)IGASSLLSDIER(Q)

Exclusive (unique to this protein): TRUE

SEQUEST XCorr score: 2.38 SEQUEST DCn score: 0.398

Fix modifications: None

Variable modifications: None

charge: 2 Observed m/z: 630.8433

Actual minus calculated peptide mass (AMU): -0.001343

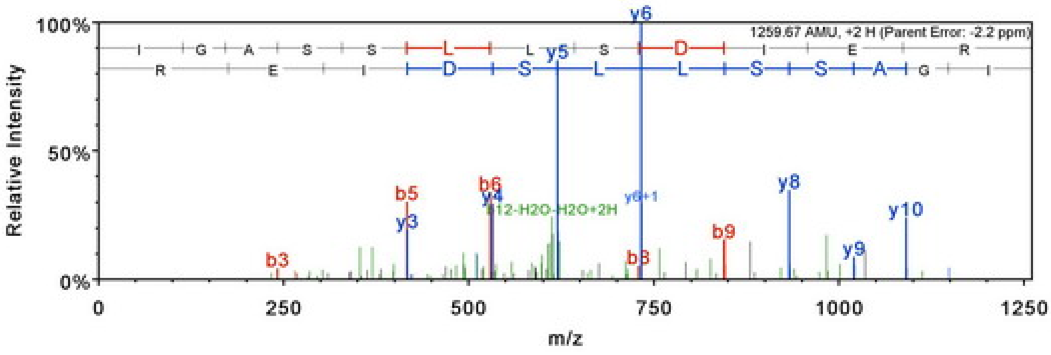

B	B Ions	B+2H	B-NH3	B-H2O	AA	Y Ions	Y+2H	Y-NH3	Y-H2O	Y
1	114.1				I	1260.7	630.8	1243.7	1242.7	12
2	171.1				G	1147.6	574.3	1130.6	1129.6	11
3	242.2				A	1090.6	545.8	1073.5	1072.6	10
4	329.2			311.2	S	1019.5	510.3	1002.5	1001.5	9
5	416.2			398.2	S	932.5	466.8	915.5	914.5	8
6	529.3	265.1		511.3	L	845.5	423.2	828.5	827.5	7
7	642.4	321.7		624.4	L	732.4	366.7	715.4	714.4	6
8	729.4	365.2		711.4	S	619.3		602.3	601.3	5
9	844.4	422.7		826.4	D	532.3		515.3	514.3	4
10	957.5	479.3		939.5	I	417.3		400.2	399.2	3
11	1086.6	543.8		1068.6	E	304.2		287.1	286.2	2
12	1260.7	630.8	1243.7	1242.7	R	175.1		158.1		1

Gene symbol: Dhrs7b

Protein name: Dehydrogenase/reductase

Protein accession numbers: IPI00114878

Peptide sequence: (R)NAVVVVTGATSGLGR(E)

Exclusive (unique to this protein): TRUE

SEQUEST XCorr score: 2.93 SEQUEST DCn score: 0.672

Fix modifications: None

Variable modifications: None

charge: 2 Observed m/z: 700.8969

Actual minus calculated peptide mass (AMU): -0.0001221

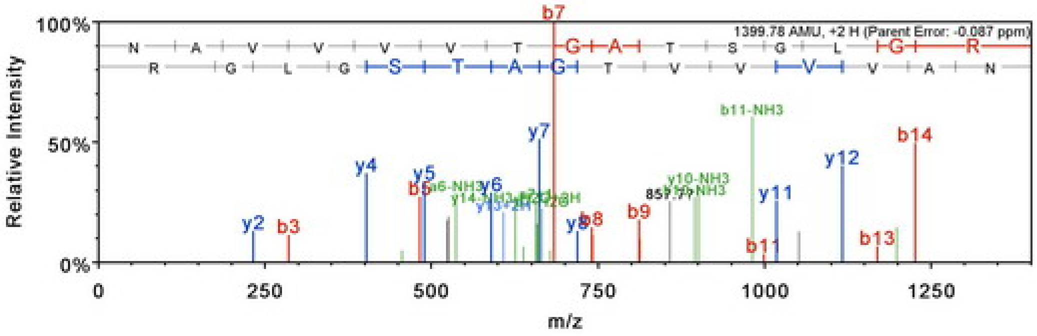

B	B Ions	B+2H	B-NH3	B-H2O	AA	Y Ions	Y+2H	Y-NH3	Y-H2O	Y
1	115.1		98.0		N	1400.8	700.9	1383.8	1382.8	15
2	186.1		169.1		A	1286.7	643.9	1269.7	1268.7	14
3	285.2		268.1		V	1215.7	608.4	1198.7	1197.7	13
4	384.2		367.2		V	1116.6	558.8	1099.6	1098.6	12
5	483.3		466.3		V	1017.6	509.3	1000.5	999.6	11
6	582.4	291.7	565.3		V	918.5	459.8	901.5	900.5	10
7	683.4	342.2	666.4	665.4	T	819.4	410.2	802.4	801.4	9
8	740.4	370.7	723.4	722.4	G	718.4	359.7	701.4	700.4	8
9	811.5	406.2	794.4	793.5	A	661.4	331.2	644.3	643.4	7
10	912.5	456.8	895.5	894.5	T	590.3	295.7	573.3	572.3	6
11	999.6	500.3	982.5	981.5	S	489.3		472.3	471.3	5
12	1056.6	528.8	1039.5	1038.6	G	402.3		385.2		4
13	1169.7	585.3	1152.6	1151.6	L	345.2		328.2		3
14	1226.7	613.8	1209.7	1208.7	G	232.1		215.1		2
15	1400.8	700.9	1383.8	1382.8	R	175.1		158.1		1

Gene symbol: Dld

Protein name: TIB-55 BB88 cDNA, RIKEN full-length enriched library, clone:I730035F24 product:dihydrolipoamide dehydrogenase, full insert sequence

Protein accession numbers: IPI00331564

Peptide sequence: (K)NETLGGTCLNVGCIPSK(A)

Exclusive (unique to this protein): TRUE

SEQUEST XCorr score: 2 SEQUEST DCn score: 0.606

Fix modifications: C8: Carbamidomethyl (+57.02), C13: Carbamidomethyl (+57.02)Variable modifications: None

charge: 2 Observed m/z: 910.4363

Actual minus calculated peptide mass (AMU): -0.003784

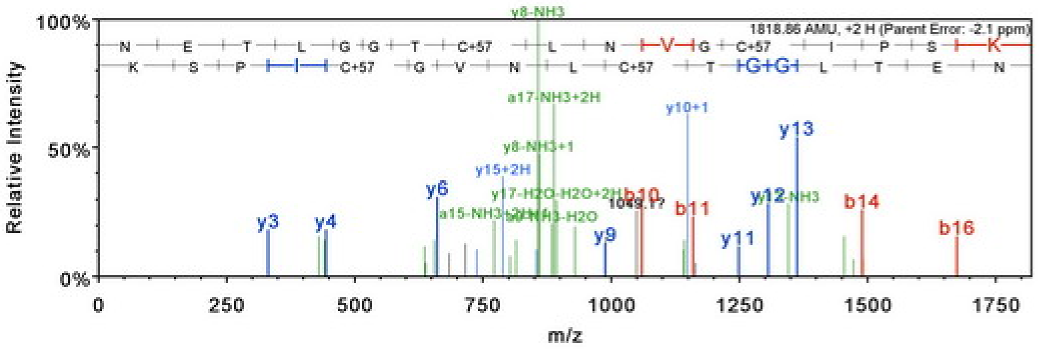

B	B Ions	B+2H	B-NH3	B-H2O	AA	Y Ions	Y+2H	Y-NH3	Y-H2O	Y
1	115.1		98.0		N	1819.9	910.4	1802.8	1801.9	17
2	244.1		227.1	226.1	E	1705.8	853.4	1688.8	1687.8	16
3	345.1		328.1	327.1	T	1576.8	788.9	1559.8	1558.8	15
4	458.2		441.2	440.2	L	1475.7	738.4	1458.7	1457.7	14
5	515.3		498.2	497.2	G	1362.7	681.8	1345.6	1344.6	13
6	572.3	286.6	555.2	554.3	G	1305.6	653.3	1288.6	1287.6	12
7	673.3	337.2	656.3	655.3	T	1248.6	624.8	1231.6	1230.6	11
8	833.4	417.2	816.3	815.3	C+57	1147.6	574.3	1130.5	1129.5	10
9	946.4	473.7	929.4	928.4	L	987.5	494.3	970.5	969.5	9
10	1060.5	530.7	1043.5	1042.5	N	874.5	437.7	857.4	856.4	8
11	1159.5	580.3	1142.5	1141.5	V	760.4	380.7	743.4	742.4	7
12	1216.6	608.8	1199.5	1198.5	G	661.3	331.2	644.3	643.3	6
13	1376.6	688.8	1359.6	1358.6	C+57	604.3		587.3	586.3	5
14	1489.7	745.3	1472.7	1471.7	I	444.3		427.3	426.3	4
15	1586.7	793.9	1569.7	1568.7	P	331.2		314.2	313.2	3
16	1673.8	837.4	1656.7	1655.8	S	234.2		217.1	216.1	2
17	1819.9	910.4	1802.8	1801.9	K	147.1		130.1		1

Gene symbol: Dmn

Protein name: Synemin isoform H

Protein accession numbers: IPI00469184

Peptide sequence: (K)PGPVVLSVGADISGSGRMPGSER(S)

Exclusive (unique to this protein): TRUE

SEQUEST XCorr score: 2.59 SEQUEST DCn score: 0.24

Fix modifications: None

Variable modifications: None

charge: 3 Observed m/z: 742.3797

Actual minus calculated peptide mass (AMU): -0.011456

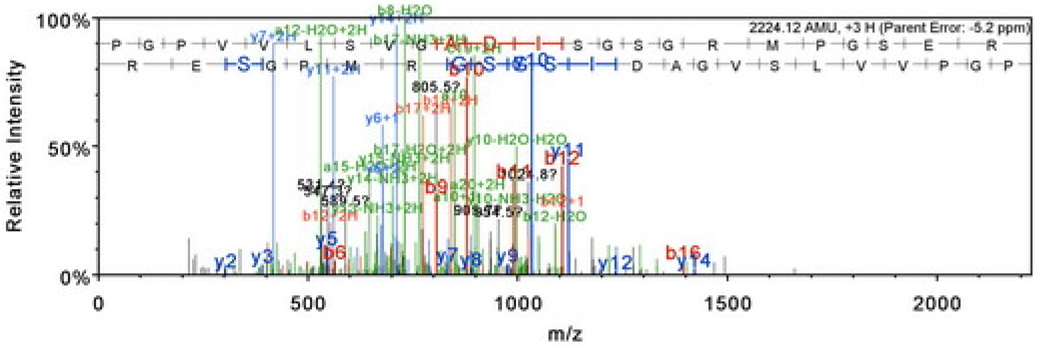

B	B Ions	B+2H	B-NH3	B-H2O	AA	Y Ions	Y+2H	Y-NH3	Y-H2O	Y
1	98.1				P	2225.1	1113.1	2208.1	2207.1	23
2	155.1				G	2128.1	1064.5	2111.1	2110.1	22
3	252.1				P	2071.1	1036.0	2054.0	2053.1	21
4	351.2				V	1974.0	987.5	1957.0	1956.0	20
5	450.3				V	1874.9	938.0	1857.9	1856.9	19
6	563.4	282.2			L	1775.9	888.4	1758.8	1757.9	18
7	650.4	325.7		632.4	S	1662.8	831.9	1645.8	1644.8	17
8	749.5	375.2		731.5	V	1575.8	788.4	1558.7	1557.7	16
9	806.5	403.7		788.5	G	1476.7	738.9	1459.7	1458.7	15
10	877.5	439.3		859.5	A	1419.7	710.3	1402.6	1401.7	14
11	992.5	496.8		974.5	D	1348.6	674.8	1331.6	1330.6	13
12	1105.6	553.3		1087.6	I	1233.6	617.3	1216.6	1215.6	12
13	1192.7	596.8		1174.7	S	1120.5	560.8	1103.5	1102.5	11
14	1249.7	625.3		1231.7	G	1033.5	517.3	1016.5	1015.5	10
15	1336.7	668.9		1318.7	S	976.5	488.7	959.4	958.5	9
16	1393.7	697.4		1375.7	G	889.4	445.2	872.4	871.4	8
17	1549.8	775.4	1532.8	1531.8	R	832.4	416.7	815.4	814.4	7
18	1680.9	840.9	1663.9	1662.9	M	676.3	338.7	659.3	658.3	6
19	1777.9	889.5	1760.9	1759.9	P	545.3		528.2	527.3	5
20	1835.0	918.0	1817.9	1816.9	G	448.2		431.2	430.2	4
21	1922.0	961.5	1905.0	1904.0	S	391.2		374.2	373.2	3
22	2051.0	1026.0	2034.0	2033.0	E	304.2		287.1	286.2	2
23	2225.1	1113.1	2208.1	2207.1	R	175.1		158.1		1

Gene symbol: Dnaja2

Protein name: DnaJ homolog subfamily A member 2

Protein accession numbers: IPI00136251

Peptide sequence: (K)NVLCSACSGQGGKSGAVQK(C)

Exclusive (unique to this protein): TRUE

SEQUEST XCorr score: 2.01 SEQUEST DCn score: 0.175

Fix modifications: C4: Carbamidomethyl (+57.02), C7: Carbamidomethyl (+57.02)Variable modifications: None

charge: 2 Observed m/z: 954.4629

Actual minus calculated peptide mass (AMU): 0.011149

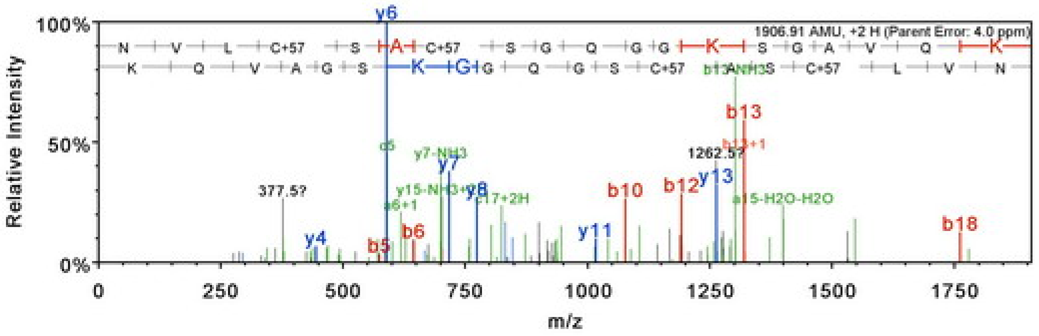

B	B Ions	B+2H	B-NH3	B-H2O	AA	Y Ions	Y+2H	Y-NH3	Y-H2O	Y
1	115.1		98.0		N	1907.9	954.5	1890.9	1889.9	19
2	214.1		197.1		V	1793.9	897.4	1776.8	1775.9	18
3	327.2		310.2		L	1694.8	847.9	1677.8	1676.8	17
4	487.2		470.2		C+57	1581.7	791.4	1564.7	1563.7	16
5	574.3		557.2	556.3	S	1421.7	711.3	1404.7	1403.7	15
6	645.3	323.2	628.3	627.3	A	1334.7	667.8	1317.6	1316.6	14
7	805.3	403.2	788.3	787.3	C+57	1263.6	632.3	1246.6	1245.6	13
8	892.4	446.7	875.3	874.4	S	1103.6	552.3	1086.5	1085.6	12
9	949.4	475.2	932.4	931.4	G	1016.6	508.8	999.5	998.5	11
10	1077.5	539.2	1060.4	1059.4	Q	959.5	480.3	942.5	941.5	10
11	1134.5	567.7	1117.4	1116.5	G	831.5	416.2	814.4	813.5	9
12	1191.5	596.3	1174.5	1173.5	G	774.5	387.7	757.4	756.4	8
13	1319.6	660.3	1302.6	1301.6	K	717.4	359.2	700.4	699.4	7
14	1406.6	703.8	1389.6	1388.6	S	589.3	295.2	572.3	571.3	6
15	1463.6	732.3	1446.6	1445.6	G	502.3		485.3		5
16	1534.7	767.8	1517.7	1516.7	A	445.3		428.3		4
17	1633.7	817.4	1616.7	1615.7	V	374.2		357.2		3
18	1761.8	881.4	1744.8	1743.8	Q	275.2		258.1		2
19	1907.9	954.5	1890.9	1889.9	K	147.1		130.1		1

Gene symbol: Dnajc13

Protein name: DnaJ (Hsp40) homolog, subfamily C, member 13 isoform 1

Protein accession numbers: IPI00378580,IPI00458074

Peptide sequence: (R)SLQYGEQVSEILSR(S)

Exclusive (unique to this protein): TRUE

SEQUEST XCorr score: 2.46 SEQUEST DCn score: 0.487

Fix modifications: None

Variable modifications: None

charge: 2 Observed m/z: 804.9125

Actual minus calculated peptide mass (AMU): -0.006226

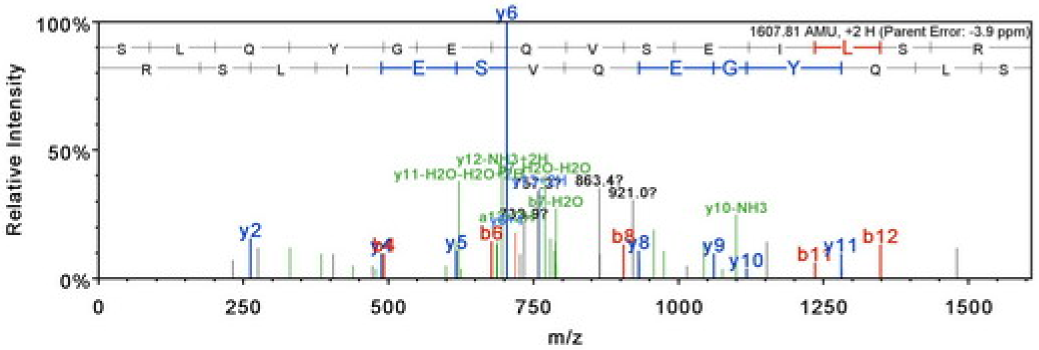

B	B Ions	B+2H	B-NH3	B-H2O	AA	Y Ions	Y+2H	Y-NH3	Y-H2O	Y
1	88.0			70.0	S	1608.8	804.9	1591.8	1590.8	14
2	201.1			183.1	L	1521.8	761.4	1504.8	1503.8	13
3	329.2		312.2	311.2	Q	1408.7	704.9	1391.7	1390.7	12
4	492.3		475.2	474.2	Y	1280.7	640.8	1263.6	1262.6	11
5	549.3		532.2	531.3	G	1117.6	559.3	1100.6	1099.6	10
6	678.3	339.7	661.3	660.3	E	1060.6	530.8	1043.5	1042.5	9
7	806.4	403.7	789.3	788.4	Q	931.5	466.3	914.5	913.5	8
8	905.4	453.2	888.4	887.4	V	803.5	402.2	786.4	785.5	7
9	992.5	496.7	975.4	974.5	S	704.4	352.7	687.4	686.4	6
10	1121.5	561.3	1104.5	1103.5	E	617.4		600.3	599.4	5
11	1234.6	617.8	1217.6	1216.6	I	488.3		471.3	470.3	4
12	1347.7	674.3	1330.7	1329.7	L	375.2		358.2	357.2	3
13	1434.7	717.9	1417.7	1416.7	S	262.1		245.1	244.1	2
14	1608.8	804.9	1591.8	1590.8	R	175.1		158.1		1

Gene symbol: Dnm1l

Protein name: Isoform 2 of Dynamin-1-like protein

Protein accession numbers: IPI00172221,IPI00556723,IPI00556781

Peptide sequence: (R)TLESVDPLGGLNTIDILTAIR(N)

Exclusive (unique to this protein): TRUE

SEQUEST XCorr score: 2.75 SEQUEST DCn score: 0.765

Fix modifications: None

Variable modifications: None

charge: 2 Observed m/z: 1106.1146

Actual minus calculated peptide mass (AMU): -0.002686

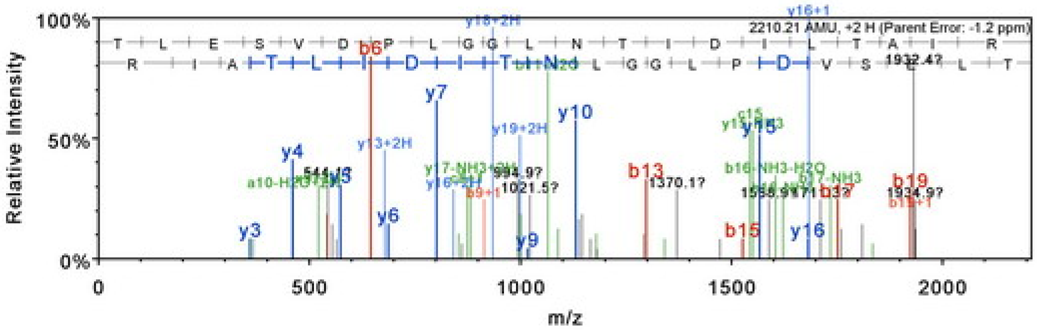

B	B Ions	B+2H	B-NH3	B-H2O	AA	Y Ions	Y+2H	Y-NH3	Y-H2O	Y
1	102.1			84.0	T	2211.2	1106.1	2194.2	2193.2	21
2	215.1			197.1	L	2110.2	1055.6	2093.2	2092.2	20
3	344.2			326.2	E	1997.1	999.1	1980.1	1979.1	19
4	431.2			413.2	S	1868.1	934.5	1851.0	1850.0	18
5	530.3			512.3	V	1781.0	891.0	1764.0	1763.0	17
6	645.3	323.2		627.3	D	1682.0	841.5	1664.9	1663.9	16
7	742.4	371.7		724.4	P	1566.9	784.0	1549.9	1548.9	15
8	855.5	428.2		837.4	L	1469.9	735.4	1452.8	1451.9	14
9	912.5	456.7		894.5	G	1356.8	678.9	1339.8	1338.8	13
10	969.5	485.3		951.5	G	1299.8	650.4	1282.7	1281.8	12
11	1082.6	541.8		1064.6	L	1242.7	621.9	1225.7	1224.7	11
12	1196.6	598.8	1179.6	1178.6	N	1129.7	565.3	1112.6	1111.7	10
13	1297.7	649.3	1280.6	1279.7	T	1015.6	508.3	998.6	997.6	9
14	1410.8	705.9	1393.7	1392.7	I	914.6	457.8	897.5	896.6	8
15	1525.8	763.4	1508.8	1507.8	D	801.5	401.3	784.5	783.5	7
16	1638.9	819.9	1621.8	1620.9	I	686.5	343.7	669.4	668.5	6
17	1751.9	876.5	1734.9	1733.9	L	573.4		556.4	555.4	5
18	1853.0	927.0	1836.0	1835.0	T	460.3		443.3	442.3	4
19	1924.0	962.5	1907.0	1906.0	A	359.2		342.2		3
20	2037.1	1019.1	2020.1	2019.1	I	288.2		271.2		2
21	2211.2	1106.1	2194.2	2193.2	R	175.1		158.1		1

Gene symbol: Dock7

Protein name: Isoform 2 of Dedicator of cytokinesis protein 7

Protein accession numbers: IPI00339428,IPI00816914,IPI00828891

Peptide sequence: (R)IGDMRIMENNLESELK(S)

Exclusive (unique to this protein): TRUE

SEQUEST XCorr score: 2.02 SEQUEST DCn score: 0.315

Fix modifications: None

Variable modifications: M4: Oxidation (+16.00), M7: Oxidation (+16.00)

charge: 2 Observed m/z: 962.4594

Actual minus calculated peptide mass (AMU): -0.004761

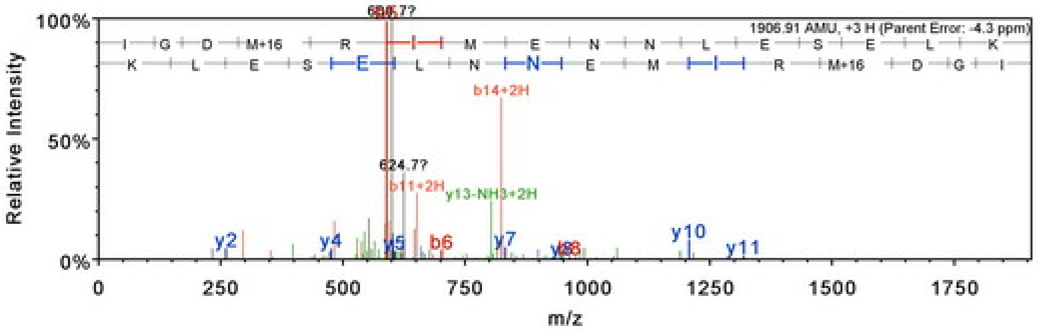

B	B Ions	B+2H	B-NH3	B-H2O	AA	Y Ions	Y+2H	Y-NH3	Y-H2O	Y
1	114.1				I	1907.9	954.5	1890.9	1889.9	16
2	171.1				G	1794.8	897.9	1777.8	1776.8	15
3	286.1			268.1	D	1737.8	869.4	1720.8	1719.8	14
4	433.2			415.2	M+16	1622.8	811.9	1605.8	1604.8	13
5	589.3	295.1	572.3	571.3	R	1475.8	738.4	1458.7	1457.7	12
6	702.4	351.7	685.3	684.4	I	1319.7	660.3	1302.6	1301.6	11
7	833.4	417.2	816.4	815.4	M	1206.6	603.8	1189.5	1188.6	10
8	962.4	481.7	945.4	944.4	E	1075.5	538.3	1058.5	1057.5	9
9	1076.5	538.8	1059.5	1058.5	N	946.5	473.8	929.5	928.5	8
10	1190.5	595.8	1173.5	1172.5	N	832.4	416.7	815.4	814.4	7
11	1303.6	652.3	1286.6	1285.6	L	718.4	359.7	701.4	700.4	6
12	1432.7	716.8	1415.6	1414.7	E	605.3		588.3	587.3	5
13	1519.7	760.4	1502.7	1501.7	S	476.3		459.3	458.3	4
14	1648.7	824.9	1631.7	1630.7	E	389.2		372.2	371.2	3
15	1761.8	881.4	1744.8	1743.8	L	260.2		243.2		2
16	1907.9	954.5	1890.9	1889.9	K	147.1		130.1		1

Gene symbol: Dpm1

Protein name: Dolichol-phosphate mannosyltransferase

Protein accession numbers: IPI00115668,IPI00751087

Peptide sequence: (R)EVAEQLAEIYGPDR(I)

Exclusive (unique to this protein): TRUE

SEQUEST XCorr score: 2.2 SEQUEST DCn score: 0.608

Fix modifications: None

Variable modifications: None

charge: 2 Observed m/z: 795.3901

Actual minus calculated peptide mass (AMU): -0.0089149

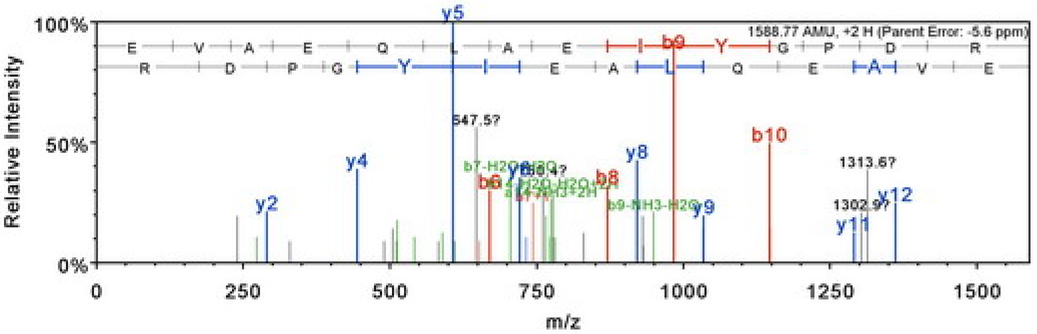

B	B Ions	B+2H	B-NH3	B-H2O	AA	Y Ions	Y+2H	Y-NH3	Y-H2O	Y
1	130.1			112.0	E	1589.8	795.4	1572.8	1571.8	14
2	229.1			211.1	V	1460.7	730.9	1443.7	1442.7	13
3	300.2			282.2	A	1361.7	681.3	1344.6	1343.7	12
4	429.2			411.2	E	1290.6	645.8	1273.6	1272.6	11
5	557.3		540.2	539.3	Q	1161.6	581.3	1144.6	1143.6	10
6	670.3	335.7	653.3	652.3	L	1033.5	517.3	1016.5	1015.5	9
7	741.4	371.2	724.4	723.4	A	920.5	460.7	903.4	902.4	8
8	870.4	435.7	853.4	852.4	E	849.4	425.2	832.4	831.4	7
9	983.5	492.3	966.5	965.5	I	720.4	360.7	703.3	702.4	6
10	1146.6	573.8	1129.5	1128.6	Y	607.3		590.3	589.3	5
11	1203.6	602.3	1186.6	1185.6	G	444.2		427.2	426.2	4
12	1300.6	650.8	1283.6	1282.6	P	387.2		370.2	369.2	3
13	1415.7	708.3	1398.6	1397.7	D	290.2		273.1	272.1	2
14	1589.8	795.4	1572.8	1571.8	R	175.1		158.1		1

Gene symbol: Dstn

Protein name: Destrin

Protein accession numbers: IPI00127942

Peptide sequence: (R)YALYDASFETK(E)

Exclusive (unique to this protein): TRUE

SEQUEST XCorr score: 2.54 SEQUEST DCn score: 0.613

Fix modifications: None

Variable modifications: None

charge: 2 Observed m/z: 654.3118

Actual minus calculated peptide mass (AMU): -0.0002441

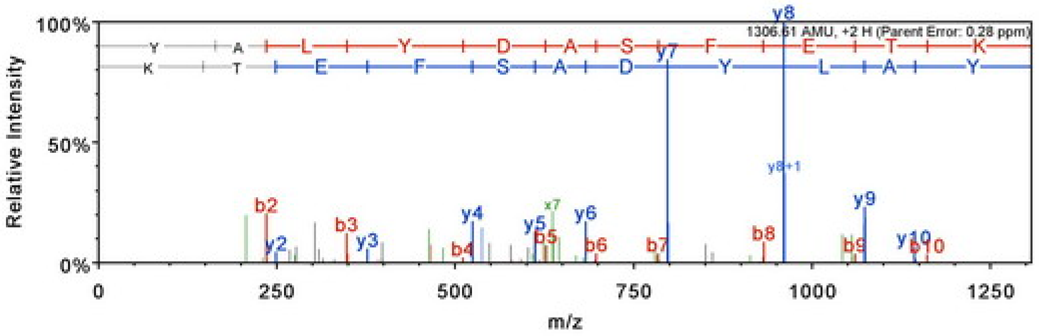

B	B Ions	B+2H	B-NH3	B-H2O	AA	Y Ions	Y+2H	Y-NH3	Y-H2O	Y
1	164.1				Y	1307.6	654.3	1290.6	1289.6	11
2	235.1				A	1144.5	572.8	1127.5	1126.5	10
3	348.2				L	1073.5	537.3	1056.5	1055.5	9
4	511.3				Y	960.4	480.7	943.4	942.4	8
5	626.3			608.3	D	797.4	399.2	780.3	779.4	7
6	697.3	349.2		679.3	A	682.3	341.7	665.3	664.3	6
7	784.4	392.7		766.3	S	611.3		594.3	593.3	5
8	931.4	466.2		913.4	F	524.3		507.3	506.3	4
9	1060.5	530.7		1042.5	E	377.2		360.2	359.2	3
10	1161.5	581.3		1143.5	T	248.2		231.1	230.2	2
11	1307.6	654.3	1290.6	1289.6	K	147.1		130.1		1

Gene symbol: Dtymk

Protein name: Isoform Long of Thymidylate kinase

Protein accession numbers: IPI00130997,IPI00230524,IPI00831272

Peptide sequence: (R)GALIVLEGVDR(A)

Exclusive (unique to this protein): TRUE

SEQUEST XCorr score: 2.16 SEQUEST DCn score: 0.534

Fix modifications: None

Variable modifications: None

charge: 2 Observed m/z: 571.3302

Actual minus calculated peptide mass (AMU): -0.005737

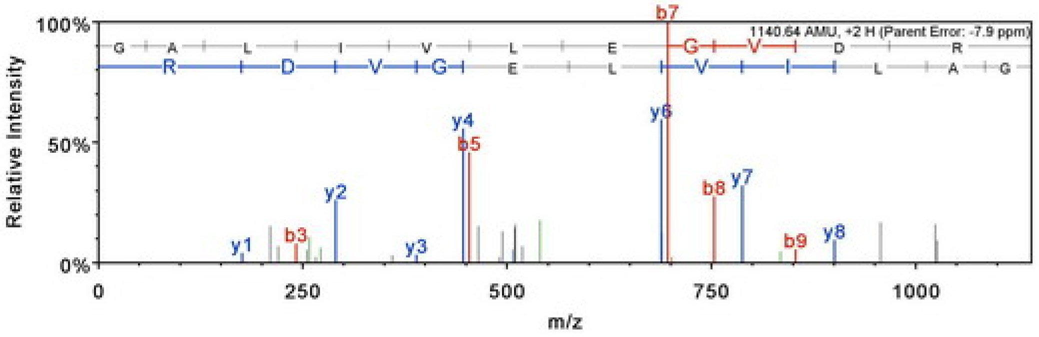

B	B Ions	B+2H	B-NH3	B-H2O	AA	Y Ions	Y+2H	Y-NH3	Y-H2O	Y
1	58.0				G	1141.7	571.3	1124.6	1123.7	11
2	129.1				A	1084.6	542.8	1067.6	1066.6	10
3	242.2				L	1013.6	507.3	996.6	995.6	9
4	355.2				I	900.5	450.8	883.5	882.5	8
5	454.3				V	787.4	394.2	770.4	769.4	7
6	567.4	284.2			L	688.4	344.7	671.3	670.4	6
7	696.4	348.7		678.4	E	575.3		558.3	557.3	5
8	753.5	377.2		735.4	G	446.2		429.2	428.2	4
9	852.5	426.8		834.5	V	389.2		372.2	371.2	3
10	967.6	484.3		949.5	D	290.2		273.1	272.1	2
11	1141.7	571.3	1124.6	1123.7	R	175.1		158.1		1

Gene symbol: Dus3l

Protein name: Isoform 1 of tRNA-dihydrouridine synthase 3-like

Protein accession numbers: IPI00129184

#NAME?

Exclusive (unique to this protein): TRUE

SEQUEST XCorr score: 2.01 SEQUEST DCn score: 0.702

Fix modifications: C19: Carbamidomethyl (+57.02)Variable modifications: M1: Oxidation (+16.00)

charge: 2 Observed m/z: 992.9106

Actual minus calculated peptide mass (AMU): 0.01575

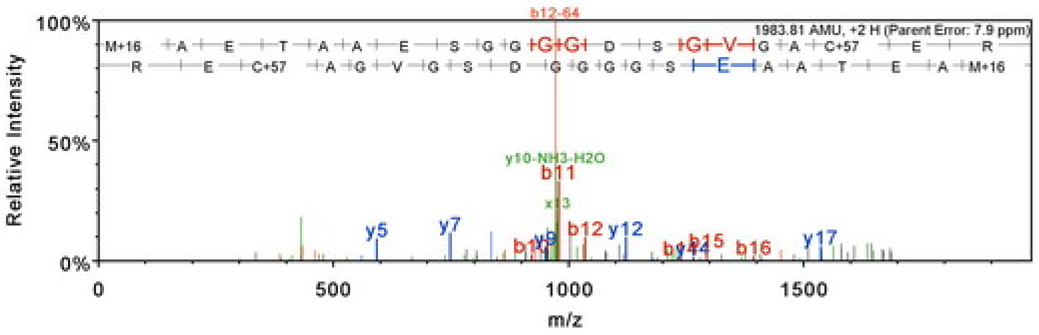

B	B Ions	B+2H	B-NH3	B-H2O	AA	Y Ions	Y+2H	Y-NH3	Y-H2O	Y
1	148.0				M+16	1984.8	992.9	1967.8	1966.8	21
2	219.1				A	1837.8	919.4	1820.7	1819.8	20
3	348.1			330.1	E	1766.7	883.9	1749.7	1748.7	19
4	449.2			431.2	T	1637.7	819.4	1620.7	1619.7	18
5	520.2			502.2	A	1536.6	768.8	1519.6	1518.6	17
6	591.2	296.1		573.2	A	1465.6	733.3	1448.6	1447.6	16
7	720.3	360.7		702.3	E	1394.6	697.8	1377.5	1376.5	15
8	807.3	404.2		789.3	S	1265.5	633.3	1248.5	1247.5	14
9	864.3	432.7		846.3	G	1178.5	589.8	1161.5	1160.5	13
10	921.4	461.2		903.4	G	1121.5	561.2	1104.4	1103.5	12
11	978.4	489.7		960.4	G	1064.4	532.7	1047.4	1046.4	11
12	1035.4	518.2		1017.4	G	1007.4	504.2	990.4	989.4	10
13	1150.4	575.7		1132.4	D	950.4	475.7	933.4	932.4	9
14	1237.5	619.2		1219.5	S	835.4	418.2	818.4	817.4	8
15	1294.5	647.8		1276.5	G	748.3	374.7	731.3	730.3	7
16	1393.5	697.3		1375.5	V	691.3	346.2	674.3	673.3	6
17	1450.6	725.8		1432.6	G	592.3		575.2	574.2	5
18	1521.6	761.3		1503.6	A	535.2		518.2	517.2	4
19	1681.6	841.3		1663.6	C+57	464.2		447.2	446.2	3
20	1810.7	905.9		1792.7	E	304.2		287.1	286.2	2
21	1984.8	992.9	1967.8	1966.8	R	175.1		158.1		1

Gene symbol: Dynll1

Protein name: Dynein light chain 1, cytoplasmic

Protein accession numbers: IPI00121623,IPI00381660

Peptide sequence: (K)YNPTWHCIVGR(N)

Exclusive (unique to this protein): TRUE

SEQUEST XCorr score: 2.31 SEQUEST DCn score: 0.634

Fix modifications: C7: Carbamidomethyl (+57.02)Variable modifications: None

charge: 2 Observed m/z: 701.8393

Actual minus calculated peptide mass (AMU): 0.001709

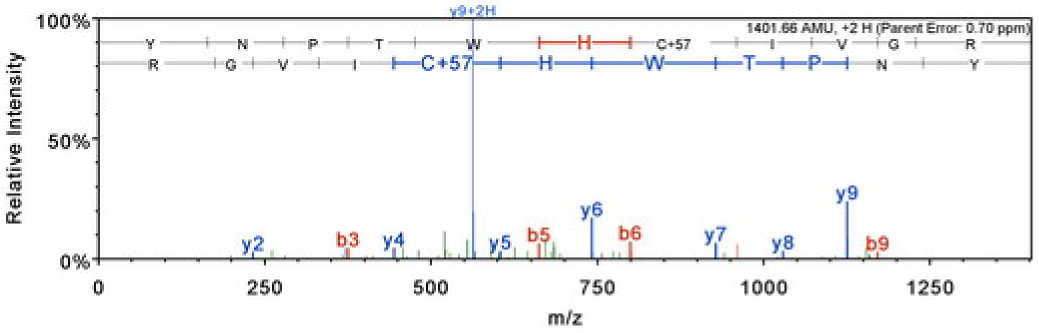

B	B Ions	B+2H	B-NH3	B-H2O	AA	Y Ions	Y+2H	Y-NH3	Y-H2O	Y
1	164.1				Y	1402.7	701.8	1385.6	1384.7	11
2	278.1		261.1		N	1239.6	620.3	1222.6	1221.6	10
3	375.2		358.1		P	1125.6	563.3	1108.5	1107.5	9
4	476.2		459.2	458.2	T	1028.5	514.8	1011.5	1010.5	8
5	662.3		645.3	644.3	W	927.5	464.2	910.4		7
6	799.4	400.2	782.3	781.3	H	741.4	371.2	724.4		6
7	959.4	480.2	942.4	941.4	C+57	604.3		587.3		5
8	1072.5	536.7	1055.4	1054.5	I	444.3		427.3		4
9	1171.5	586.3	1154.5	1153.5	V	331.2		314.2		3
10	1228.6	614.8	1211.5	1210.5	G	232.1		215.1		2
11	1402.7	701.8	1385.6	1384.7	R	175.1		158.1		1

Gene symbol: E330021D16Rik

Protein name: In vitro fertilized eggs cDNA, RIKEN full-length enriched library, clone:7420462M15 product:similar to NICE-5 protein, full insert sequence

Protein accession numbers: IPI00226065

Peptide sequence: (R)FGVDNHYTEQVAR(R)

Exclusive (unique to this protein): TRUE

SEQUEST XCorr score: 2.05　　SEQUEST DCn score: 0.471

Fix modifications: None

Variable modifications: None

charge: 2　　Observed m/z: 768.3669

Actual minus calculated peptide mass (AMU): 0.001831

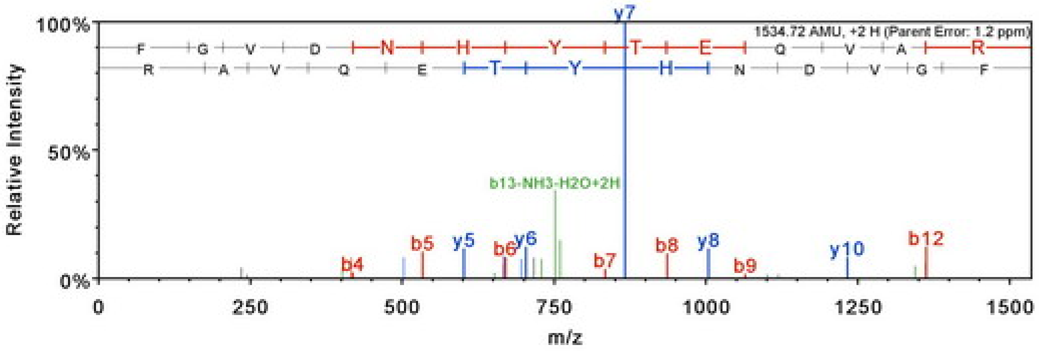

B	B Ions	B+2H	B-NH3	B-H2O	AA	Y Ions	Y+2H	Y-NH3	Y-H2O	Y
1	148.1				F	1535.7	768.4	1518.7	1517.7	13
2	205.1				G	1388.7	694.8	1371.6	1370.7	12
3	304.2				V	1331.6	666.3	1314.6	1313.6	11
4	419.2			401.2	D	1232.6	616.8	1215.5	1214.6	10
5	533.2		516.2	515.2	N	1117.5	559.3	1100.5	1099.5	9
6	670.3	335.7	653.3	652.3	H	1003.5	502.3	986.5	985.5	8
7	833.4	417.2	816.3	815.4	Y	866.4	433.7	849.4	848.4	7
8	934.4	467.7	917.4	916.4	T	703.4	352.2	686.4	685.4	6
9	1063.5	532.2	1046.4	1045.4	E	602.3		585.3	584.3	5
10	1191.5	596.3	1174.5	1173.5	Q	473.3		456.3		4
11	1290.6	645.8	1273.5	1272.6	V	345.2		328.2		3
12	1361.6	681.3	1344.6	1343.6	A	246.2		229.1		2
13	1535.7	768.4	1518.7	1517.7	R	175.1		158.1		1

Gene symbol: Echdc3

Protein name: Enoyl Coenzyme A hydratase domain containing 3

Protein accession numbers: IPI00318283

Peptide sequence: (K)SSFATPGVNVGLFCSTPAVALGR(A)

Exclusive (unique to this protein): TRUE

SEQUEST XCorr score: 2.46 SEQUEST DCn score: 0.646

Fix modifications: C14: Carbamidomethyl (+57.02)Variable modifications: None

charge: 2 Observed m/z: 1154.5885

Actual minus calculated peptide mass (AMU): -0.006836

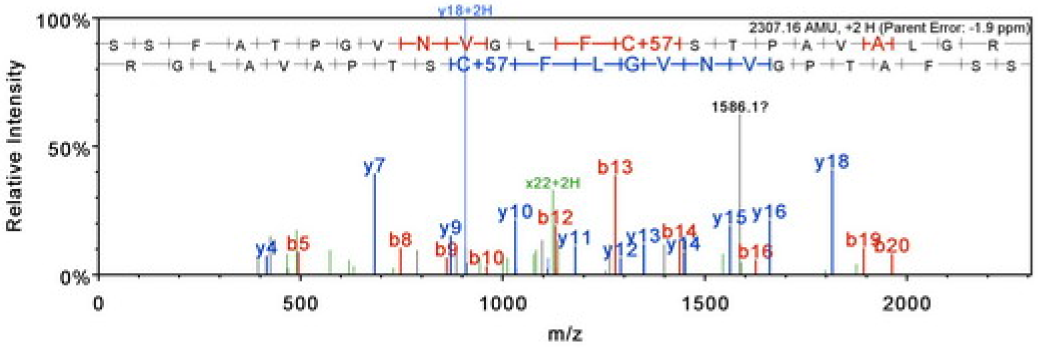

B	B Ions	B+2H	B-NH3	B-H2O	AA	Y Ions	Y+2H	Y-NH3	Y-H2O	Y
1	88.0			70.0	S	2308.2	1154.6	2291.2	2290.2	23
2	175.1			157.1	S	2221.1	1111.1	2204.1	2203.1	22
3	322.1			304.1	F	2134.1	1067.6	2117.1	2116.1	21
4	393.2			375.2	A	1987.0	994.0	1970.0	1969.0	20
5	494.2			476.2	T	1916.0	958.5	1899.0	1898.0	19
6	591.3	296.1		573.3	P	1815.0	908.0	1797.9	1797.0	18
7	648.3	324.7		630.3	G	1717.9	859.5	1700.9	1699.9	17
8	747.4	374.2		729.4	V	1660.9	831.0	1643.9	1642.9	16
9	861.4	431.2	844.4	843.4	N	1561.8	781.4	1544.8	1543.8	15
10	960.5	480.7	943.5	942.5	V	1447.8	724.4	1430.8	1429.8	14
11	1017.5	509.3	1000.5	999.5	G	1348.7	674.9	1331.7	1330.7	13
12	1130.6	565.8	1113.6	1112.6	L	1291.7	646.4	1274.7	1273.7	12
13	1277.7	639.3	1260.6	1259.6	F	1178.6	589.8	1161.6	1160.6	11
14	1437.7	719.4	1420.7	1419.7	C+57	1031.5	516.3	1014.5	1013.5	10
15	1524.7	762.9	1507.7	1506.7	S	871.5	436.3	854.5	853.5	9
16	1625.8	813.4	1608.7	1607.8	T	784.5	392.7	767.4	766.5	8
17	1722.8	861.9	1705.8	1704.8	P	683.4	342.2	666.4		7
18	1793.9	897.4	1776.8	1775.8	A	586.4	293.7	569.3		6
19	1892.9	947.0	1875.9	1874.9	V	515.3		498.3		5
20	1964.0	982.5	1946.9	1946.0	A	416.3		399.2		4
21	2077.0	1039.0	2060.0	2059.0	L	345.2		328.2		3
22	2134.1	1067.5	2117.0	2116.1	G	232.1		215.1		2
23	2308.2	1154.6	2291.2	2290.2	R	175.1		158.1		1

Gene symbol: Echs1

Protein name: Enoyl-CoA hydratase, mitochondrial precursor

Protein accession numbers: IPI00454049

Peptide sequence: (K)AQFGQPEILLGTIPGAGGTQR(L)

Exclusive (unique to this protein): TRUE

SEQUEST XCorr score: 2.02 SEQUEST DCn score: 0.521

Fix modifications: None

Variable modifications: None

charge: 2 Observed m/z: 1056.0692

Actual minus calculated peptide mass (AMU): 0.005127

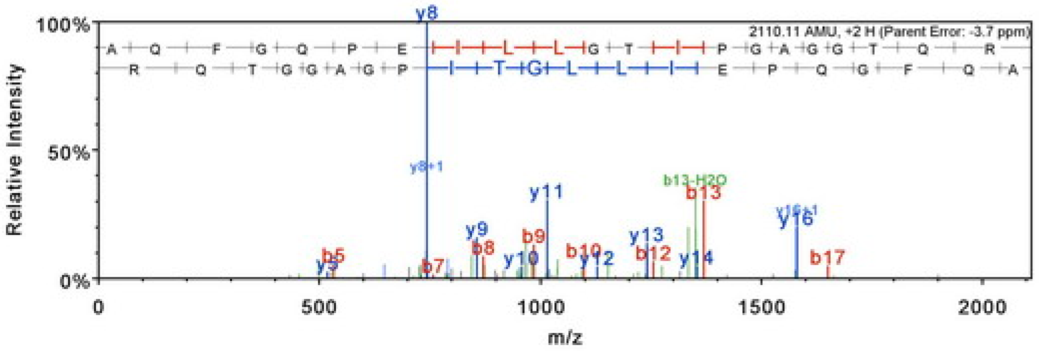

B	B Ions	B+2H	B-NH3	B-H2O	AA	Y Ions	Y+2H	Y-NH3	Y-H2O	Y
1	72.0				A	2111.1	1056.1	2094.1	2093.1	21
2	200.1		183.1		Q	2040.1	1020.6	2023.1	2022.1	20
3	347.2		330.2		F	1912.0	956.5	1895.0	1894.0	19
4	404.2		387.2		G	1765.0	883.0	1747.9	1747.0	18
5	532.3		515.2		Q	1707.9	854.5	1690.9	1689.9	17
6	629.3	315.2	612.3		P	1579.9	790.4	1562.9	1561.9	16
7	758.4	379.7	741.3	740.3	E	1482.8	741.9	1465.8	1464.8	15
8	871.4	436.2	854.4	853.4	I	1353.8	677.4	1336.8	1335.8	14
9	984.5	492.8	967.5	966.5	L	1240.7	620.9	1223.7	1222.7	13
10	1097.6	549.3	1080.6	1079.6	L	1127.6	564.3	1110.6	1109.6	12
11	1154.6	577.8	1137.6	1136.6	G	1014.5	507.8	997.5	996.5	11
12	1255.7	628.3	1238.6	1237.7	T	957.5	479.3	940.5	939.5	10
13	1368.8	684.9	1351.7	1350.7	I	856.5	428.7	839.4	838.5	9
14	1465.8	733.4	1448.8	1447.8	P	743.4	372.2	726.4	725.4	8
15	1522.8	761.9	1505.8	1504.8	G	646.3	323.7	629.3	628.3	7
16	1593.9	797.4	1576.8	1575.9	A	589.3	295.2	572.3	571.3	6
17	1650.9	826.0	1633.9	1632.9	G	518.3		501.2	500.3	5
18	1707.9	854.5	1690.9	1689.9	G	461.3		444.2	443.2	4
19	1809.0	905.0	1791.9	1790.9	T	404.2		387.2	386.2	3
20	1937.0	969.0	1920.0	1919.0	Q	303.2		286.2		2
21	2111.1	1056.1	2094.1	2093.1	R	175.1		158.1		1

Gene symbol: Edc4

Protein name: Isoform 1 of Enhancer of mRNA-decapping protein 4

Protein accession numbers: IPI00330066,IPI00831141

Peptide sequence: (R)QGPELGSQLGLDGGPGDGDR(H)

Exclusive (unique to this protein): TRUE

SEQUEST XCorr score: 2 SEQUEST DCn score: 0.642

Fix modifications: None

Variable modifications: None

charge: 2 Observed m/z: 962.9507

Actual minus calculated peptide mass (AMU): -0.006958

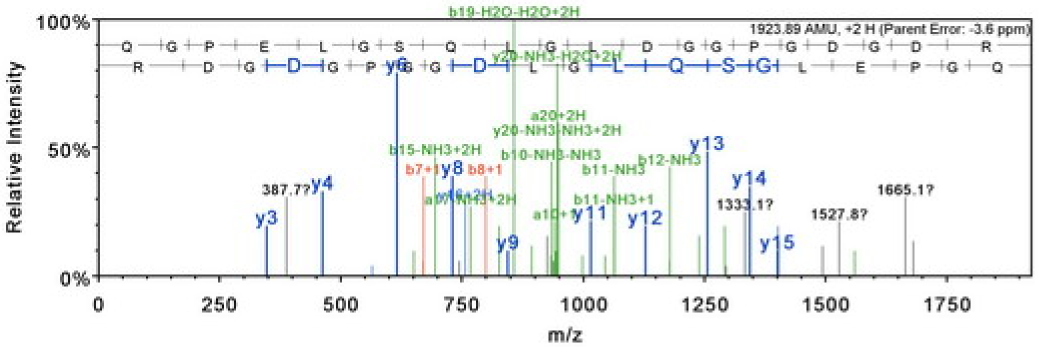

B	B Ions	B+2H	B-NH3	B-H2O	AA	Y Ions	Y+2H	Y-NH3	Y-H2O	Y
1	129.1		112.0		Q	1924.9	963.0	1907.9	1906.9	20
2	186.1		169.1		G	1796.8	898.9	1779.8	1778.8	19
3	283.1		266.1		P	1739.8	870.4	1722.8	1721.8	18
4	412.2		395.2	394.2	E	1642.8	821.9	1625.7	1624.8	17
5	525.3		508.2	507.3	L	1513.7	757.4	1496.7	1495.7	16
6	582.3	291.7	565.3	564.3	G	1400.6	700.8	1383.6	1382.6	15
7	669.3	335.2	652.3	651.3	S	1343.6	672.3	1326.6	1325.6	14
8	797.4	399.2	780.4	779.4	Q	1256.6	628.8	1239.6	1238.6	13
9	910.5	455.7	893.4	892.5	L	1128.5	564.8	1111.5	1110.5	12
10	967.5	484.3	950.5	949.5	G	1015.4	508.2	998.4	997.4	11
11	1080.6	540.8	1063.5	1062.6	L	958.4	479.7	941.4	940.4	10
12	1195.6	598.3	1178.6	1177.6	D	845.3	423.2	828.3	827.3	9
13	1252.6	626.8	1235.6	1234.6	G	730.3	365.7	713.3	712.3	8
14	1309.6	655.3	1292.6	1291.6	G	673.3	337.2	656.3	655.3	7
15	1406.7	703.9	1389.7	1388.7	P	616.3	308.6	599.2	598.3	6
16	1463.7	732.4	1446.7	1445.7	G	519.2		502.2	501.2	5
17	1578.7	789.9	1561.7	1560.7	D	462.2		445.2	444.2	4
18	1635.8	818.4	1618.7	1617.8	G	347.2		330.1	329.2	3
19	1750.8	875.9	1733.8	1732.8	D	290.2		273.1	272.1	2
20	1924.9	963.0	1907.9	1906.9	R	175.1		158.1		1

Gene symbol: EG216818

Protein name: similar to ubiquitin A-52 residue ribosomal protein fusion product 1

Protein accession numbers: IPI00345192,IPI00849015

Peptide sequence: (K)TLTDNTITLEVKPSDTIENVK(D)

Exclusive (unique to this protein): TRUE

SEQUEST XCorr score: 2.83 SEQUEST DCn score: 0.52

Fix modifications: None

Variable modifications: None

charge: 2 Observed m/z: 1166.115

Actual minus calculated peptide mass (AMU): -0.007568

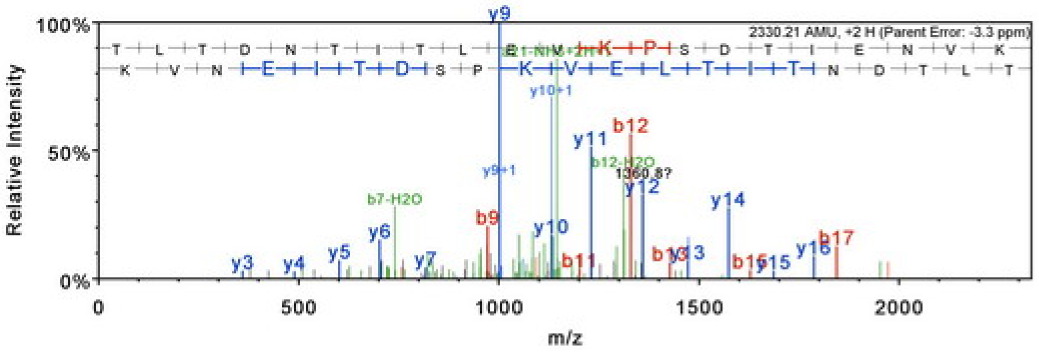

B	B Ions	B+2H	B-NH3	B-H2O	AA	Y Ions	Y+2H	Y-NH3	Y-H2O	Y
1	102.1			84.0	T	2331.2	1166.1	2314.2	2313.2	21
2	215.1			197.1	L	2230.2	1115.6	2213.2	2212.2	20
3	316.2			298.2	T	2117.1	1059.0	2100.1	2099.1	19
4	431.2			413.2	D	2016.1	1008.5	1999.0	1998.0	18
5	545.3		528.2	527.3	N	1901.0	951.0	1884.0	1883.0	17
6	646.3	323.7	629.3	628.3	T	1787.0	894.0	1770.0	1769.0	16
7	759.4	380.2	742.4	741.4	I	1685.9	843.5	1668.9	1667.9	15
8	860.4	430.7	843.4	842.4	T	1572.9	786.9	1555.8	1554.8	14
9	973.5	487.3	956.5	955.5	L	1471.8	736.4	1454.8	1453.8	13
10	1102.6	551.8	1085.5	1084.5	E	1358.7	679.9	1341.7	1340.7	12
11	1201.6	601.3	1184.6	1183.6	V	1229.7	615.3	1212.7	1211.7	11
12	1329.7	665.4	1312.7	1311.7	K	1130.6	565.8	1113.6	1112.6	10
13	1426.8	713.9	1409.8	1408.8	P	1002.5	501.8	985.5	984.5	9
14	1513.8	757.4	1496.8	1495.8	S	905.5	453.2	888.4	887.5	8
15	1628.8	814.9	1611.8	1610.8	D	818.4	409.7	801.4	800.4	7
16	1729.9	865.5	1712.9	1711.9	T	703.4	352.2	686.4	685.4	6
17	1843.0	922.0	1825.9	1825.0	I	602.4		585.3	584.3	5
18	1972.0	986.5	1955.0	1954.0	E	489.3		472.2	471.3	4
19	2086.1	1043.5	2069.0	2068.1	N	360.2		343.2		3
20	2185.1	1093.1	2168.1	2167.1	V	246.2		229.2		2
21	2331.2	1166.1	2314.2	2313.2	K	147.1		130.1		1

Gene symbol: EG233991

Protein name: similar to 60S ribosomal protein L22

Protein accession numbers: IPI00350162,IPI00755995

Peptide sequence: (K)ITVTSEVPFSK(R)

Exclusive (unique to this protein): TRUE

SEQUEST XCorr score: 2.15 SEQUEST DCn score: 0.614

Fix modifications: None

Variable modifications: None

charge: 2 Observed m/z: 604.3331

Actual minus calculated peptide mass (AMU): 0.0008545

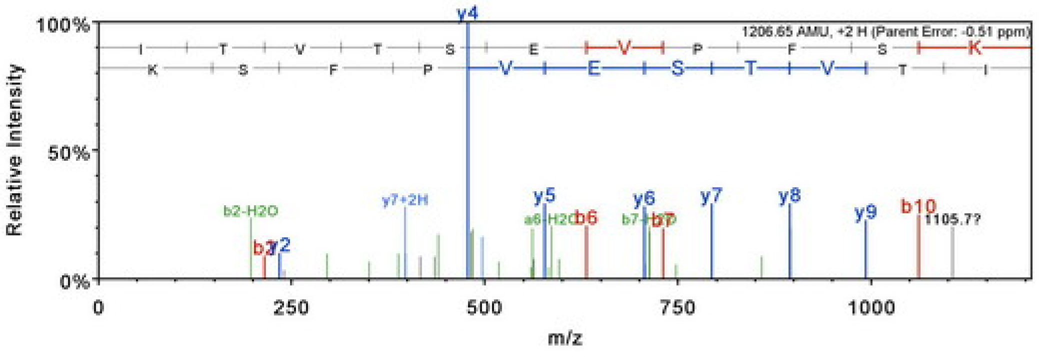

B	B Ions	B+2H	B-NH3	B-H2O	AA	Y Ions	Y+2H	Y-NH3	Y-H2O	Y
1	114.1				I	1207.7	604.3	1190.6	1189.7	11
2	215.1			197.1	T	1094.6	547.8	1077.5	1076.6	10
3	314.2			296.2	V	993.5	497.3	976.5	975.5	9
4	415.3			397.3	T	894.5	447.7	877.4	876.5	8
5	502.3			484.3	S	793.4	397.2	776.4	775.4	7
6	631.3	316.2		613.3	E	706.4	353.7	689.4	688.4	6
7	730.4	365.7		712.4	V	577.3		560.3	559.3	5
8	827.5	414.2		809.4	P	478.3		461.2	460.3	4
9	974.5	487.8		956.5	F	381.2		364.2	363.2	3
10	1061.5	531.3		1043.5	S	234.2		217.1	216.1	2
11	1207.7	604.3	1190.6	1189.7	K	147.1		130.1		1

Gene symbol: EG243302

Protein name: similar to 40S ribosomal protein S25

Protein accession numbers: IPI00115992,IPI00137735,IPI00461419,IPI00605858,IPI00624402,IPI00625320,IPI00677917,IPI00848593,IPI00850188

Peptide sequence: (R)DKLNNLVLFDK(A)

Exclusive (unique to this protein): TRUE

SEQUEST XCorr score: 2.25 SEQUEST DCn score: 0.488

Fix modifications: None

Variable modifications: None

charge: 2 Observed m/z: 659.8684

Actual minus calculated peptide mass (AMU): -0.008301

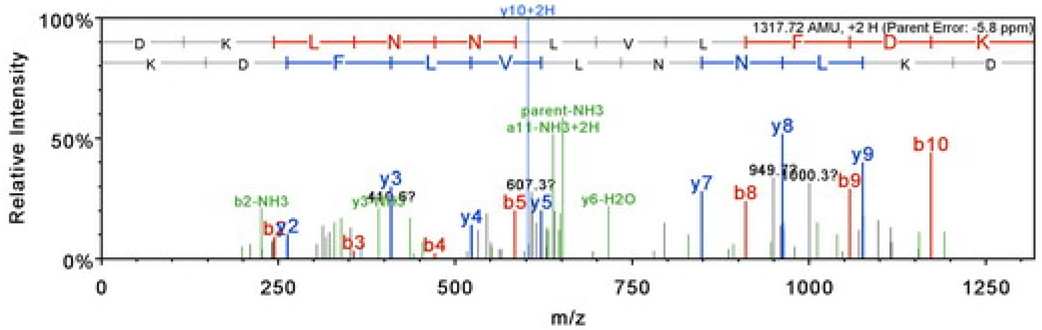

B	B Ions	B+2H	B-NH3	B-H2O	AA	Y Ions	Y+2H	Y-NH3	Y-H2O	Y
1	116.0			98.0	D	1318.7	659.9	1301.7	1300.7	11
2	244.1	122.6	227.1	226.1	K	1203.7	602.4	1186.7	1185.7	10
3	357.2	179.1	340.2	339.2	L	1075.6	538.3	1058.6	1057.6	9
4	471.3	236.1	454.2	453.3	N	962.5	481.8	945.5	944.5	8
5	585.3	293.2	568.3	567.3	N	848.5	424.8	831.5	830.5	7
6	698.4	349.7	681.4	680.4	L	734.5	367.7	717.4	716.4	6
7	797.5	399.2	780.4	779.4	V	621.4		604.3	603.4	5
8	910.5	455.8	893.5	892.5	L	522.3		505.3	504.3	4
9	1057.6	529.3	1040.6	1039.6	F	409.2		392.2	391.2	3
10	1172.6	586.8	1155.6	1154.6	D	262.1		245.1	244.1	2
11	1318.7	659.9	1301.7	1300.7	K	147.1		130.1		1

Gene symbol: EG432865
Protein name: similar to ribosomal protein S12
Protein accession numbers: IPI00116908,IPI00225634,IPI00405659,IPI00463049,IPI00463052,IPI00473415,IPI00757455
Peptide sequence: (K)LVEALCAEHQINLIK(A)
Exclusive (unique to this protein): TRUE
SEQUEST XCorr score: 2.06 SEQUEST DCn score: 0.452
Fix modifications: C6: Carbamidomethyl (+57.02)Variable modifications: None
charge: 2 Observed m/z: 875.9774
Actual minus calculated peptide mass (AMU): -0.005859

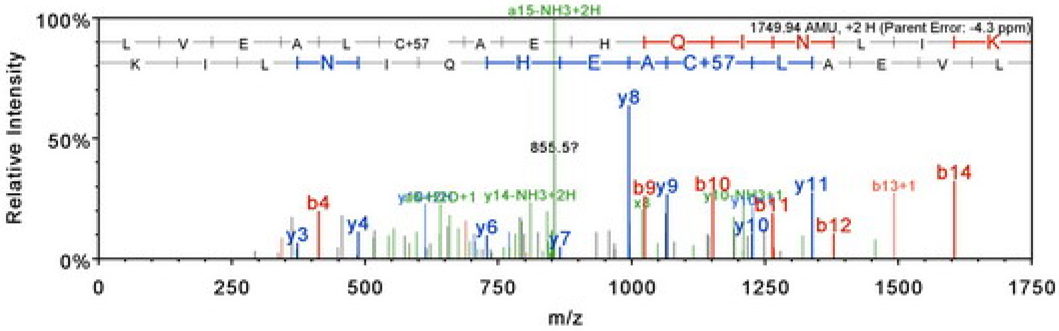

B	B Ions	B+2H	B-NH3	B-H2O	AA	Y Ions	Y+2H	Y-NH3	Y-H2O	Y
1	114.1				L	1751.0	876.0	1733.9	1732.9	15
2	213.2				V	1637.9	819.4	1620.8	1619.9	14
3	342.2			324.2	E	1538.8	769.9	1521.8	1520.8	13
4	413.2			395.2	A	1409.8	705.4	1392.7	1391.8	12
5	526.3			508.3	L	1338.7	669.9	1321.7	1320.7	11
6	686.4	343.7		668.3	C+57	1225.6	613.3	1208.6	1207.6	10
7	757.4	379.2		739.4	A	1065.6	533.3	1048.6	1047.6	9
8	886.4	443.7		868.4	E	994.6	497.8	977.5	976.6	8
9	1023.5	512.3		1005.5	H	865.5	433.3	848.5		7
10	1151.5	576.3	1134.5	1133.5	Q	728.5	364.7	711.4		6
11	1264.6	632.8	1247.6	1246.6	I	600.4		583.4		5
12	1378.7	689.8	1361.7	1360.7	N	487.3		470.3		4
13	1491.8	746.4	1474.7	1473.8	L	373.3		356.3		3
14	1604.9	802.9	1587.8	1586.8	I	260.2		243.2		2
15	1751.0	876.0	1733.9	1732.9	K	147.1		130.1		1

Gene symbol: EG432987
Protein name: Predicted
Protein accession numbers: IPI00330480
Peptide sequence: (R)SLDLDSIIAEVK(A)
Exclusive (unique to this protein): TRUE
SEQUEST XCorr score: 2.04 SEQUEST DCn score: 0.385
Fix modifications: None
Variable modifications: None
charge: 2 Observed m/z: 651.8583

Actual minus calculated peptide mass (AMU): -0.0070758

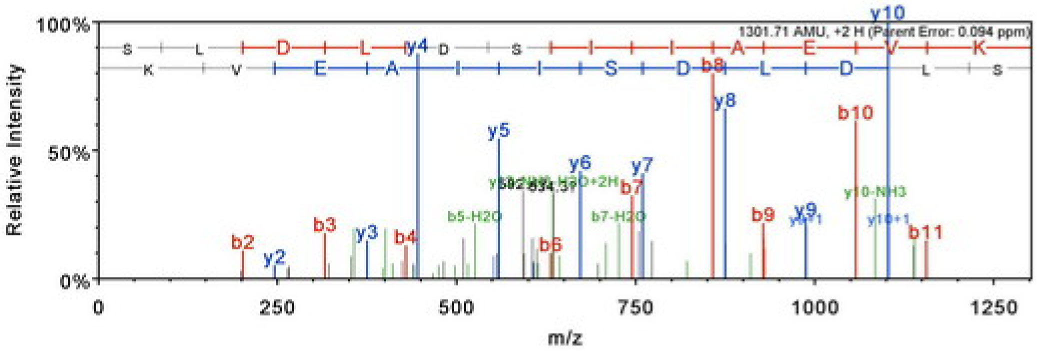

B	B Ions	B+2H	B-NH3	B-H2O	AA	Y Ions	Y+2H	Y-NH3	Y-H2O	Y
1	88.0			70.0	S	1302.7	651.9	1285.7	1284.7	12
2	201.1			183.1	L	1215.7	608.4	1198.7	1197.7	11
3	316.2			298.1	D	1102.6	551.8	1085.6	1084.6	10
4	429.2			411.2	L	987.6	494.3	970.6	969.6	9
5	544.3			526.3	D	874.5	437.8	857.5	856.5	8
6	631.3	316.2		613.3	S	759.5	380.2	742.4	741.5	7
7	744.4	372.7		726.4	I	672.4	336.7	655.4	654.4	6
8	857.5	429.2		839.5	I	559.4		542.3	541.3	5
9	928.5	464.8		910.5	A	446.3		429.2	428.3	4
10	1057.5	529.3		1039.5	E	375.2		358.2	357.2	3
11	1156.6	578.8		1138.6	V	246.2		229.2		2
12	1302.7	651.9	1285.7	1284.7	K	147.1		130.1		1

Gene symbol: EG545121;LOC672818

Protein name: similar to ribosomal protein S14

Protein accession numbers: IPI00112407,IPI00322562,IPI00663582

Peptide sequence: (R)IEDVTPIPSDSTR(R)

Exclusive (unique to this protein): TRUE

SEQUEST XCorr score: 2.16　　SEQUEST DCn score: 0.642

Fix modifications: None

Variable modifications: None

charge: 2　　Observed m/z: 715.3625

Actual minus calculated peptide mass (AMU): -0.0003662

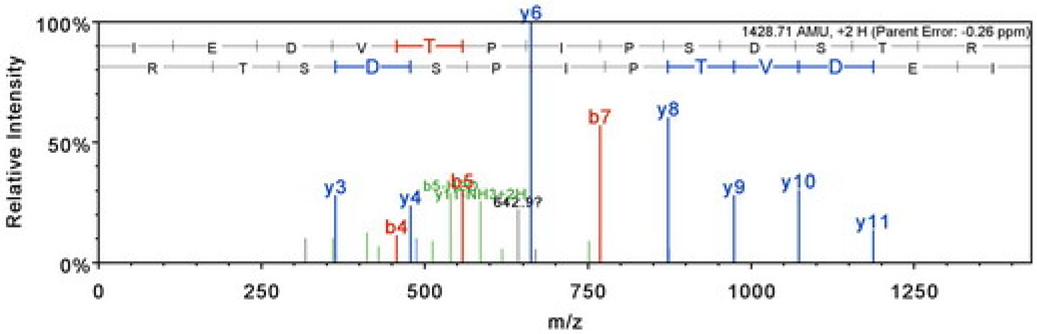

B	B Ions	B+2H	B-NH3	B-H2O	AA	Y Ions	Y+2H	Y-NH3	Y-H2O	Y
1	114.1				I	1429.7	715.4	1412.7	1411.7	13
2	243.1			225.1	E	1316.6	658.8	1299.6	1298.6	12
3	358.2			340.2	D	1187.6	594.3	1170.6	1169.6	11
4	457.2			439.2	V	1072.6	536.8	1055.5	1054.5	10
5	558.3			540.3	T	973.5	487.3	956.5	955.5	9
6	655.3	328.2		637.3	P	872.5	436.7	855.4	854.4	8
7	768.4	384.7		750.4	I	775.4	388.2	758.4	757.4	7
8	865.5	433.2		847.5	P	662.3	331.7	645.3	644.3	6
9	952.5	476.8		934.5	S	565.3		548.2	547.3	5
10	1067.5	534.3		1049.5	D	478.2		461.2	460.2	4
11	1154.6	577.8		1136.5	S	363.2		346.2	345.2	3
12	1255.6	628.3		1237.6	T	276.2		259.1	258.2	2
13	1429.7	715.4	1412.7	1411.7	R	175.1		158.1		1

Gene symbol: EG626175

Protein name: similar to 40S ribosomal protein S26

Protein accession numbers: IPI00261455,IPI00377441,IPI00658829,IPI00674601,IPI00754798,IPI00762861

Peptide sequence: (R)DISEASVFDAYVLPK(L)

Exclusive (unique to this protein): TRUE

SEQUEST XCorr score: 2.4 SEQUEST DCn score: 0.615

Fix modifications: None

Variable modifications: None

charge: 2 Observed m/z: 827.4192

Actual minus calculated peptide mass (AMU): -0.0070758

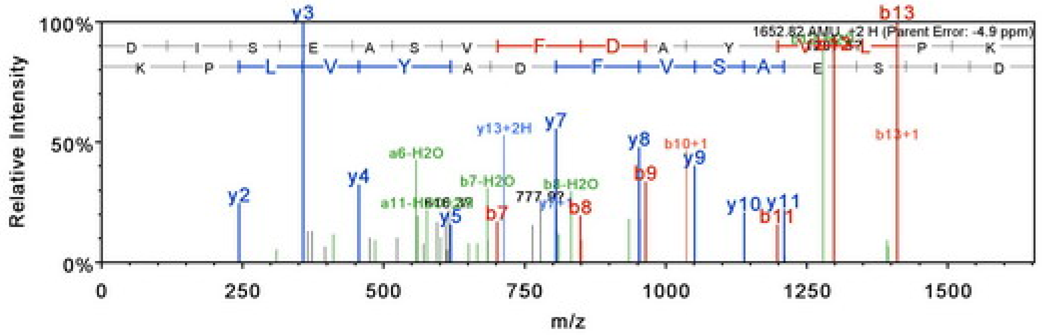

B	B Ions	B+2H	B-NH3	B-H2O	AA	Y Ions	Y+2H	Y-NH3	Y-H2O	Y
1	116.0			98.0	D	1653.8	827.4	1636.8	1635.8	15
2	229.1			211.1	I	1538.8	769.9	1521.8	1520.8	14
3	316.2			298.1	S	1425.7	713.4	1408.7	1407.7	13
4	445.2			427.2	E	1338.7	669.9	1321.7	1320.7	12
5	516.2			498.2	A	1209.7	605.3	1192.6	1191.6	11
6	603.3	302.1		585.3	S	1138.6	569.8	1121.6	1120.6	10
7	702.3	351.7		684.3	V	1051.6	526.3	1034.6	1033.6	9
8	849.4	425.2		831.4	F	952.5	476.8	935.5	934.5	8
9	964.4	482.7		946.4	D	805.5	403.2	788.4	787.4	7
10	1035.5	518.2		1017.5	A	690.4	345.7	673.4		6
11	1198.5	599.8		1180.5	Y	619.4		602.4		5
12	1297.6	649.3		1279.6	V	456.3		439.3		4
13	1410.7	705.8		1392.7	L	357.3		340.2		3
14	1507.7	754.4		1489.7	P	244.2		227.1		2
15	1653.8	827.4	1636.8	1635.8	K	147.1		130.1		1
