# Supplementary material for: Proteomic-based identification of maternal proteins in mature mouse oocytes
Source: BMC Genomics. 2009 Aug 3;10:348. doi: 10.1186/1471-2164-10-348 (PMC2730056; doi:10.1186/1471-2164-10-348)
Supplement: Additional file 3 — Data from single peptide-based identification of oocyte proteins. Contains MS/MS spectra and fragment tables of single peptide-based identifications. [file 1471-2164-10-348-S3.pdf]

Gene symbol: EG665646
Protein name: similar to Heterogeneous nuclear ribonucleoprotein A1 (Helix-destabilizing protein) (Single-strand binding protein) (hnRNP core protein A1) (HDP-1) (Topoisomerase-inhibitor suppressed) isoform 2
Protein accession numbers: IPI00224251,IPI00553777,IPI00753579,IPI00753723,IPI00817004
Peptide sequence: (R)GFAFVTFDDHDSVDK(I)
Exclusive (unique to this protein): TRUE
SEQUEST XCorr score: 2.2 SEQUEST DCn score: 0.51
Fix modifications: None
Variable modifications: None
charge: 2 Observed m/z: 850.386
Actual minus calculated peptide mass (AMU): 0.003662

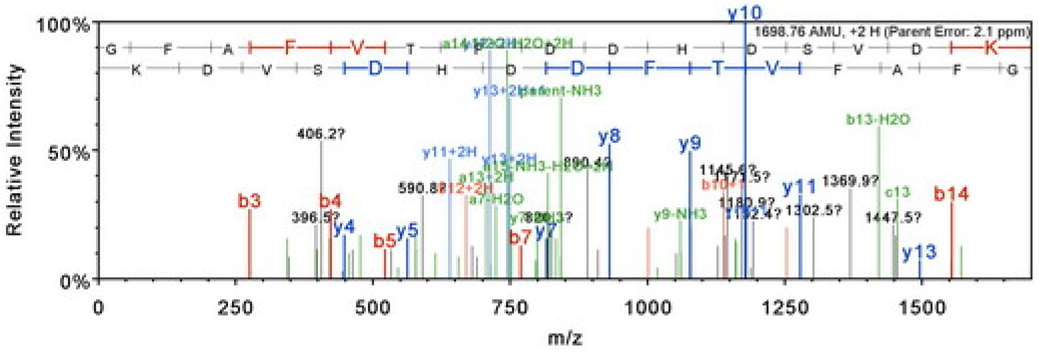

B	B Ions	B+2H	B-NH3	B-H2O	AA	Y Ions	Y+2H	Y-NH3	Y-H2O	Y
1	58.0				G	1699.8	850.4	1682.7	1681.8	15
2	205.1				F	1642.7	821.9	1625.7	1624.7	14
3	276.1				A	1495.7	748.3	1478.6	1477.7	13
4	423.2				F	1424.6	712.8	1407.6	1406.6	12
5	522.3				V	1277.6	639.3	1260.5	1259.5	11
6	623.3	312.2		605.3	T	1178.5	589.8	1161.5	1160.5	10
7	770.4	385.7		752.4	F	1077.5	539.2	1060.4	1059.4	9
8	885.4	443.2		867.4	D	930.4	465.7	913.4	912.4	8
9	1000.4	500.7		982.4	D	815.4	408.2	798.3	797.3	7
10	1137.5	569.3		1119.5	H	700.3	350.7	683.3	682.3	6
11	1252.5	626.8		1234.5	D	563.3		546.2	545.3	5
12	1339.6	670.3		1321.5	S	448.2		431.2	430.2	4
13	1438.6	719.8		1420.6	V	361.2		344.2	343.2	3
14	1553.7	777.3		1535.6	D	262.1		245.1	244.1	2
15	1699.8	850.4	1682.7	1681.8	K	147.1		130.1		1

Gene symbol: EG667682;Rpl31;LOC676592
Protein name: 60S ribosomal protein L31
Protein accession numbers: IPI00123007,IPI00126910,IPI00676466,IPI00677102,IPI00753412
Peptide sequence: (K)LYTLVTYVPVTTFK(N)
Exclusive (unique to this protein): TRUE
SEQUEST XCorr score: 2.13 SEQUEST DCn score: 0.642
Fix modifications: None

Variable modifications: None

charge: 2   Observed m/z: 822.9641

Actual minus calculated peptide mass (AMU): -0.005005

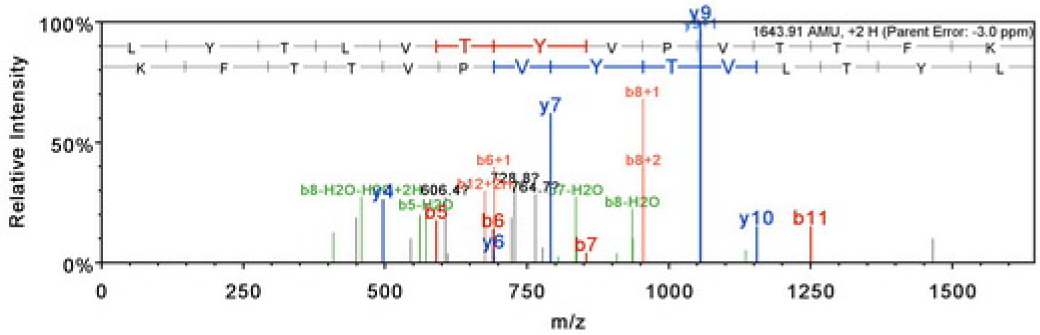

B	B Ions	B+2H	B-NH3	B-H2O	AA	Y Ions	Y+2H	Y-NH3	Y-H2O	Y
1	114.1				L	1644.9	823.0	1627.9	1626.9	14
2	277.2				Y	1531.8	766.4	1514.8	1513.8	13
3	378.2			360.2	T	1368.8	684.9	1351.8	1350.8	12
4	491.3			473.3	L	1267.7	634.4	1250.7	1249.7	11
5	590.4			572.3	V	1154.7	577.8	1137.6	1136.6	10
6	691.4	346.2		673.4	T	1055.6	528.3	1038.5	1037.6	9
7	854.5	427.7		836.5	Y	954.5	477.8	937.5	936.5	8
8	953.5	477.3		935.5	V	791.5	396.2	774.4	773.5	7
9	1050.6	525.8		1032.6	P	692.4	346.7	675.4	674.4	6
10	1149.7	575.3		1131.7	V	595.4		578.3	577.3	5
11	1250.7	625.9		1232.7	T	496.3		479.3	478.3	4
12	1351.8	676.4		1333.7	T	395.2		378.2	377.2	3
13	1498.8	749.9		1480.8	F	294.2		277.2		2
14	1644.9	823.0	1627.9	1626.9	K	147.1		130.1		1

Gene symbol: EG667847;LOC676775

Protein name: similar to 40S ribosomal protein S2 isoform 2

Protein accession numbers: IPI00752723,IPI00753940

Peptide sequence: (K)TYSYLTPDLWK(E)

Exclusive (unique to this protein): TRUE

SEQUEST XCorr score: 2.38   SEQUEST DCn score: 0.702

Fix modifications: None

Variable modifications: None

charge: 2   Observed m/z: 693.8522

Actual minus calculated peptide mass (AMU): 0.0019551

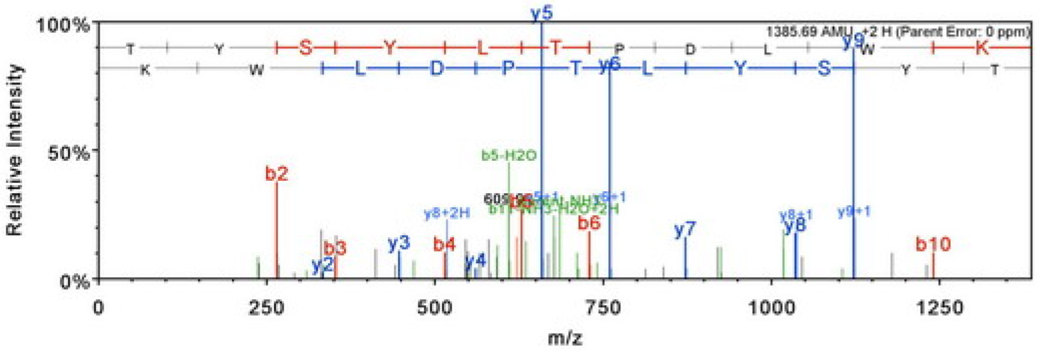

B	B Ions	B+2H	B-NH3	B-H2O	AA	Y Ions	Y+2H	Y-NH3	Y-H2O	Y
1	102.1			84.0	T	1386.7	693.9	1369.7	1368.7	11
2	265.1			247.1	Y	1285.7	643.3	1268.6	1267.6	10
3	352.2			334.1	S	1122.6	561.8	1105.6	1104.6	9
4	515.2			497.2	Y	1035.5	518.3	1018.5	1017.5	8
5	628.3			610.3	L	872.5	436.8	855.5	854.5	7
6	729.4	365.2		711.3	T	759.4	380.2	742.4	741.4	6
7	826.4	413.7		808.4	P	658.4		641.3	640.4	5
8	941.4	471.2		923.4	D	561.3		544.3	543.3	4
9	1054.5	527.8		1036.5	L	446.3		429.3		3
10	1240.6	620.8		1222.6	W	333.2		316.2		2
11	1386.7	693.9	1369.7	1368.7	K	147.1		130.1		1

Gene symbol: EG668182;LOC675192;LOC672281
Protein name: similar to 60S ribosomal protein L13
Protein accession numbers: IPI00134097,IPI00224505,IPI00621028,IPI00625838,IPI00653474,IPI00761637
Peptide sequence: (R)VDTWFNQPAR(K)
Exclusive (unique to this protein): TRUE
SEQUEST XCorr score: 2.05 SEQUEST DCn score: 0.501
Fix modifications: None
Variable modifications: None
charge: 2 Observed m/z: 617.3032
Actual minus calculated peptide mass (AMU): -0.0031752

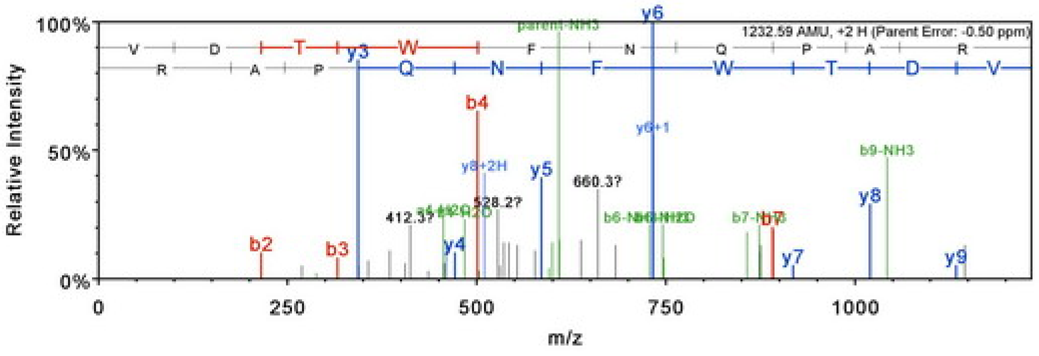

B	B Ions	B+2H	B-NH3	B-H2O	AA	Y Ions	Y+2H	Y-NH3	Y-H2O	Y
1	100.1				V	1233.6	617.3	1216.6	1215.6	10
2	215.1			197.1	D	1134.5	567.8	1117.5	1116.5	9
3	316.2			298.1	T	1019.5	510.3	1002.5	1001.5	8
4	502.2			484.2	W	918.5	459.7	901.4		7
5	649.3			631.3	F	732.4	366.7	715.4		6
6	763.3	382.2	746.3	745.3	N	585.3		568.3		5
7	891.4	446.2	874.4	873.4	Q	471.3		454.2		4
8	988.5	494.7	971.4	970.4	P	343.2		326.2		3
9	1059.5	530.3	1042.5	1041.5	A	246.2		229.1		2
10	1233.6	617.3	1216.6	1215.6	R	175.1		158.1		1

Gene symbol: Eif2s2

Protein name: Eukaryotic translation initiation factor 2 subunit 2

Protein accession numbers: IPI00116302,IPI00480398

Peptide sequence: (R)DYTYEELLNR(V)

Exclusive (unique to this protein): TRUE

SEQUEST XCorr score: 2.2 SEQUEST DCn score: 0.558

Fix modifications: None

Variable modifications: None

charge: 2 Observed m/z: 658.3093

Actual minus calculated peptide mass (AMU): -0.006226

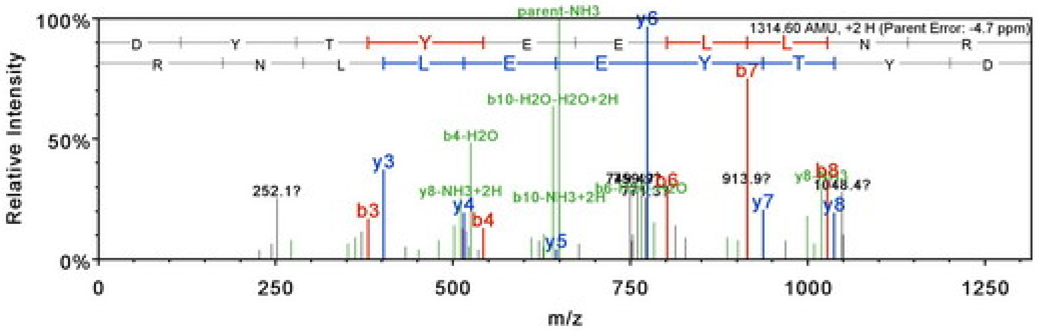

B	B Ions	B+2H	B-NH3	B-H2O	AA	Y Ions	Y+2H	Y-NH3	Y-H2O	Y
1	116.0			98.0	D	1315.6	658.3	1298.6	1297.6	10
2	279.1			261.1	Y	1200.6	600.8	1183.6	1182.6	9
3	380.2			362.1	T	1037.5	519.3	1020.5	1019.5	8
4	543.2			525.2	Y	936.5	468.7	919.5	918.5	7
5	672.3			654.2	E	773.4	387.2	756.4	755.4	6
6	801.3	401.2		783.3	E	644.4		627.4	626.4	5
7	914.4	457.7		896.4	L	515.3		498.3		4
8	1027.5	514.2		1009.5	L	402.3		385.2		3
9	1141.5	571.3	1124.5	1123.5	N	289.2		272.1		2
10	1315.6	658.3	1298.6	1297.6	R	175.1		158.1		1

Gene symbol: Eif4e1b

Protein name: 2 cells egg cDNA, RIKEN full-length enriched library, clone:B020005H16 product:weakly similar to Eukaryotic translation initiation factor eIF4E-1

Protein accession numbers: IPI00341534

Peptide sequence: (R)AWQDNLQLVTK(F)

Exclusive (unique to this protein): TRUE

SEQUEST XCorr score: 3.56 SEQUEST DCn score: 0.647

Fix modifications: None

Variable modifications: None

charge: 2 Observed m/z: 658.3536

Actual minus calculated peptide mass (AMU): -0.001831

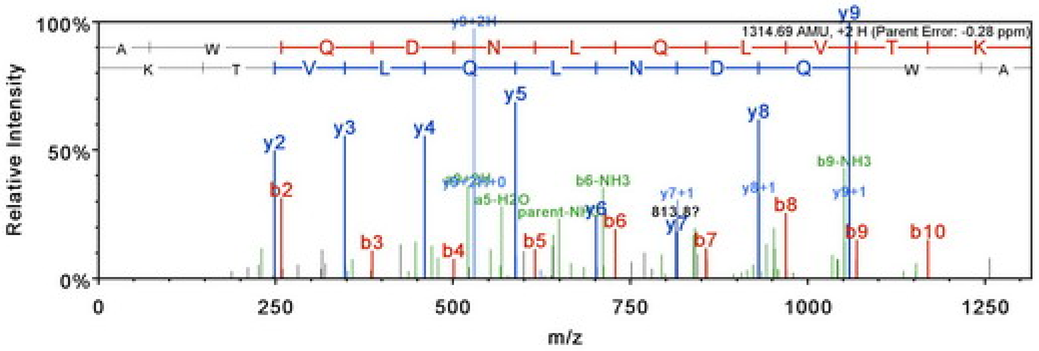

B	B Ions	B+2H	B-NH3	B-H2O	AA	Y Ions	Y+2H	Y-NH3	Y-H2O	Y
1	72.0				A	1315.7	658.4	1298.7	1297.7	11
2	258.1				W	1244.7	622.8	1227.6	1226.7	10
3	386.2		369.2		Q	1058.6	529.8	1041.6	1040.6	9
4	501.2		484.2	483.2	D	930.5	465.8	913.5	912.5	8
5	615.3		598.2	597.2	N	815.5	408.3	798.5	797.5	7
6	728.3	364.7	711.3	710.3	L	701.5	351.2	684.4	683.5	6
7	856.4	428.7	839.4	838.4	Q	588.4		571.4	570.4	5
8	969.5	485.2	952.5	951.5	L	460.3		443.3	442.3	4
9	1068.5	534.8	1051.5	1050.5	V	347.2		330.2	329.2	3
10	1169.6	585.3	1152.6	1151.6	T	248.2		231.1	230.2	2
11	1315.7	658.4	1298.7	1297.7	K	147.1		130.1		1

Gene symbol: Eif4e3

Protein name: Eukaryotic translation initiation factor 4E type 3

Protein accession numbers: IPI00319027

Peptide sequence: (R)SLPGATAAECASNLK(K)

Exclusive (unique to this protein): TRUE

SEQUEST XCorr score: 2.28 SEQUEST DCn score: 0.583

Fix modifications: C10: Carbamidomethyl (+57.02)Variable modifications: None

charge: 2 Observed m/z: 745.371

Actual minus calculated peptide mass (AMU): 0.002075

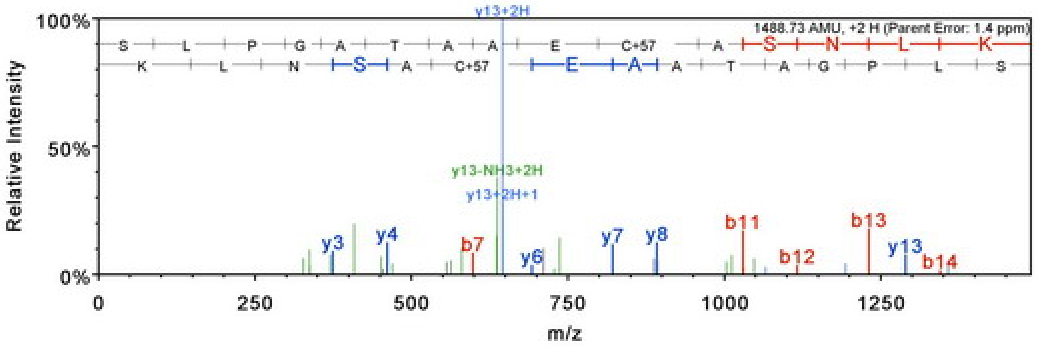

B	B Ions	B+2H	B-NH3	B-H2O	AA	Y Ions	Y+2H	Y-NH3	Y-H2O	Y
1	88.0			70.0	S	1489.7	745.4	1472.7	1471.7	15
2	201.1			183.1	L	1402.7	701.9	1385.7	1384.7	14
3	298.2			280.2	P	1289.6	645.3	1272.6	1271.6	13
4	355.2			337.2	G	1192.6	596.8	1175.5	1174.5	12
5	426.2			408.2	A	1135.5	568.3	1118.5	1117.5	11
6	527.3	264.1		509.3	T	1064.5	532.8	1047.5	1046.5	10
7	598.3	299.7		580.3	A	963.5	482.2	946.4	945.5	9
8	669.4	335.2		651.4	A	892.4	446.7	875.4	874.4	8
9	798.4	399.7		780.4	E	821.4	411.2	804.4	803.4	7
10	958.4	479.7		940.4	C+57	692.3	346.7	675.3	674.3	6
11	1029.5	515.2		1011.5	A	532.3		515.3	514.3	5
12	1116.5	558.8		1098.5	S	461.3		444.3	443.3	4
13	1230.5	615.8	1213.5	1212.5	N	374.2		357.2		3
14	1343.6	672.3	1326.6	1325.6	L	260.2		243.2		2
15	1489.7	745.4	1472.7	1471.7	K	147.1		130.1		1

Gene symbol: Eif4h

Protein name: Isoform Long of Eukaryotic translation initiation factor 4H

Protein accession numbers: IPI00124742,IPI00222560

Peptide sequence: (R)TVATPLNQVANPNSAIFGGAR(P)

Exclusive (unique to this protein): TRUE

SEQUEST XCorr score: 2.62 SEQUEST DCn score: 0.644

Fix modifications: None

Variable modifications: None

charge: 2 Observed m/z: 1049.5596

Actual minus calculated peptide mass (AMU): 0.006836

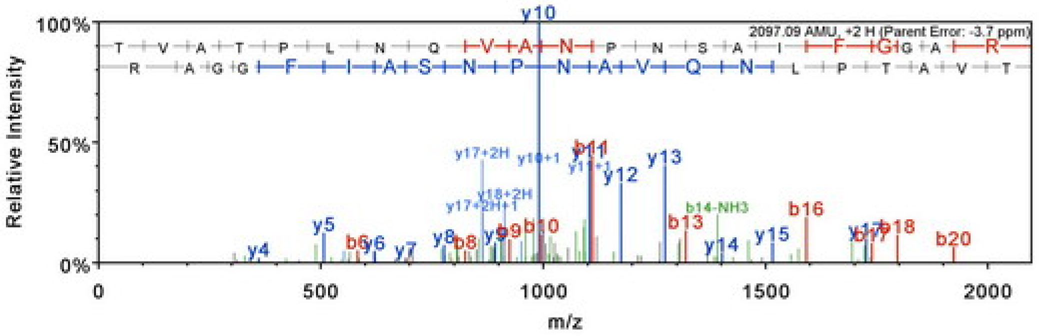

B	B Ions	B+2H	B-NH3	B-H2O	AA	Y Ions	Y+2H	Y-NH3	Y-H2O	Y
1	102.1			84.0	T	2098.1	1049.6	2081.1	2080.1	21
2	201.1			183.1	V	1997.1	999.0	1980.0	1979.1	20
3	272.2			254.2	A	1898.0	949.5	1881.0	1880.0	19
4	373.2			355.2	T	1827.0	914.0	1809.9	1808.9	18
5	470.3			452.3	P	1725.9	863.5	1708.9	1707.9	17
6	583.4	292.2		565.3	L	1628.9	814.9	1611.8	1610.8	16
7	697.4	349.2	680.4	679.4	N	1515.8	758.4	1498.7	1497.8	15
8	825.5	413.2	808.4	807.4	Q	1401.7	701.4	1384.7	1383.7	14
9	924.5	462.8	907.5	906.5	V	1273.7	637.3	1256.6	1255.7	13
10	995.6	498.3	978.5	977.5	A	1174.6	587.8	1157.6	1156.6	12
11	1109.6	555.3	1092.6	1091.6	N	1103.6	552.3	1086.5	1085.5	11
12	1206.7	603.8	1189.6	1188.6	P	989.5	495.3	972.5	971.5	10
13	1320.7	660.9	1303.7	1302.7	N	892.5	446.7	875.4	874.5	9
14	1407.7	704.4	1390.7	1389.7	S	778.4	389.7	761.4	760.4	8
15	1478.8	739.9	1461.7	1460.8	A	691.4	346.2	674.4		7
16	1591.8	796.4	1574.8	1573.8	I	620.4	310.7	603.3		6
17	1738.9	870.0	1721.9	1720.9	F	507.3		490.2		5
18	1795.9	898.5	1778.9	1777.9	G	360.2		343.2		4
19	1853.0	927.0	1835.9	1835.0	G	303.2		286.2		3
20	1924.0	962.5	1907.0	1906.0	A	246.2		229.1		2
21	2098.1	1049.6	2081.1	2080.1	R	175.1		158.1		1

Gene symbol: Eif5a

Protein name: Eukaryotic translation initiation factor 5A-1

Protein accession numbers: IPI00108125,IPI00648714

Peptide sequence: (K)VHLVGIDIFTGK(K)

Exclusive (unique to this protein): TRUE

SEQUEST XCorr score: 2.01 SEQUEST DCn score: 0.546

Fix modifications: None

Variable modifications: None

charge: 2 Observed m/z: 649.8741

Actual minus calculated peptide mass (AMU): -0.0070758

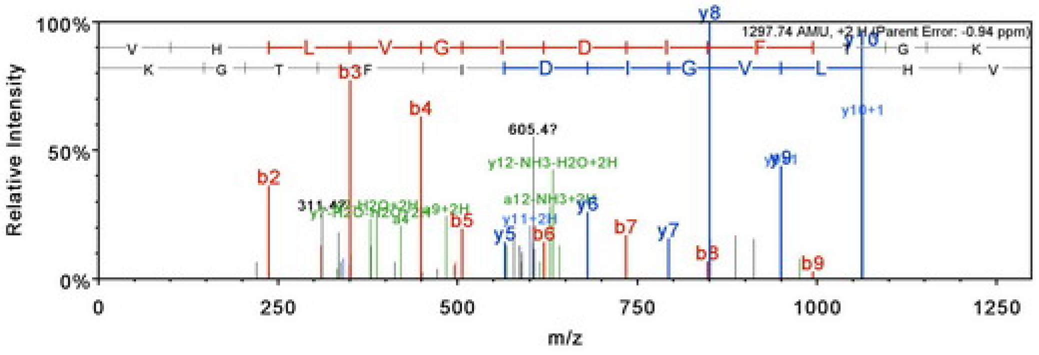

B	B Ions	B+2H	B-NH3	B-H2O	AA	Y Ions	Y+2H	Y-NH3	Y-H2O	Y
1	100.1				V	1298.8	649.9	1281.7	1280.7	12
2	237.1	119.1			H	1199.7	600.3	1182.7	1181.7	11
3	350.2	175.6			L	1062.6	531.8	1045.6	1044.6	10
4	449.3	225.2			V	949.5	475.3	932.5	931.5	9
5	506.3	253.7			G	850.5	425.7	833.4	832.5	8
6	619.4	310.2			I	793.5	397.2	776.4	775.4	7
7	734.4	367.7		716.4	D	680.4	340.7	663.3	662.4	6
8	847.5	424.3		829.5	I	565.3		548.3	547.3	5
9	994.6	497.8		976.6	F	452.3		435.2	434.2	4
10	1095.6	548.3		1077.6	T	305.2		288.2	287.2	3
11	1152.6	576.8		1134.6	G	204.1		187.1		2
12	1298.8	649.9	1281.7	1280.7	K	147.1		130.1		1

Gene symbol: Elmod2
Protein name: ELMO domain-containing protein 2
Protein accession numbers: IPI00467315
Peptide sequence: (K)QWADIGFQGDDPK(T)
Exclusive (unique to this protein): TRUE
SEQUEST XCorr score: 2.12 SEQUEST DCn score: 0.473
Fix modifications: None
Variable modifications: None
charge: 2 Observed m/z: 738.8408
Actual minus calculated peptide mass (AMU): -0.002441

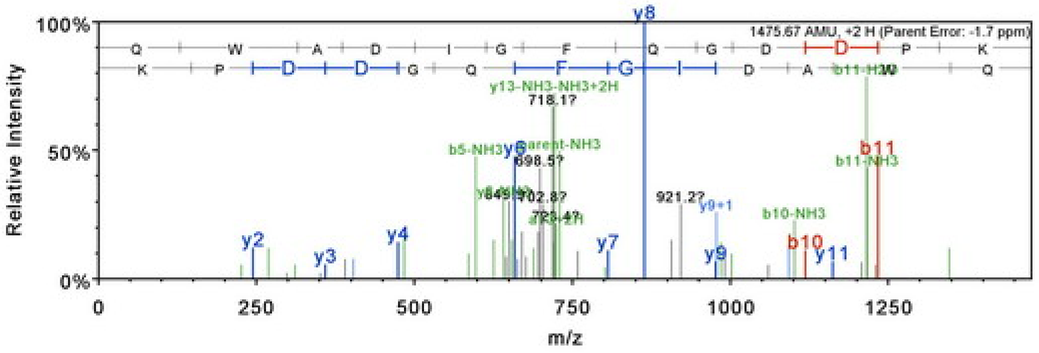

B	B Ions	B+2H	B-NH3	B-H2O	AA	Y Ions	Y+2H	Y-NH3	Y-H2O	Y
1	129.1		112.0		Q	1476.7	738.8	1459.7	1458.7	13
2	315.2		298.1		W	1348.6	674.8	1331.6	1330.6	12
3	386.2		369.2		A	1162.5	581.8	1145.5	1144.5	11
4	501.2		484.2	483.2	D	1091.5	546.3	1074.5	1073.5	10
5	614.3		597.3	596.3	I	976.5	488.7	959.5	958.5	9
6	671.3	336.2	654.3	653.3	G	863.4	432.2	846.4	845.4	8
7	818.4	409.7	801.4	800.4	F	806.4	403.7	789.3	788.4	7
8	946.4	473.7	929.4	928.4	Q	659.3	330.2	642.3	641.3	6
9	1003.5	502.2	986.4	985.5	G	531.2		514.2	513.2	5
10	1118.5	559.8	1101.5	1100.5	D	474.2		457.2	456.2	4
11	1233.5	617.3	1216.5	1215.5	D	359.2		342.2	341.2	3
12	1330.6	665.8	1313.5	1312.6	P	244.2		227.1		2
13	1476.7	738.8	1459.7	1458.7	K	147.1		130.1		1

Gene symbol: Eprs
Protein name: Bifunctional aminoacyl-tRNA synthetase
Protein accession numbers: IPI00339916
Peptide sequence: (R)VSETVAFTDVNSILR(Y)
Exclusive (unique to this protein): TRUE
SEQUEST XCorr score: 2.25 SEQUEST DCn score: 0.621
Fix modifications: None
Variable modifications: None
charge: 2 Observed m/z: 825.9382
Actual minus calculated peptide mass (AMU): -0.001709

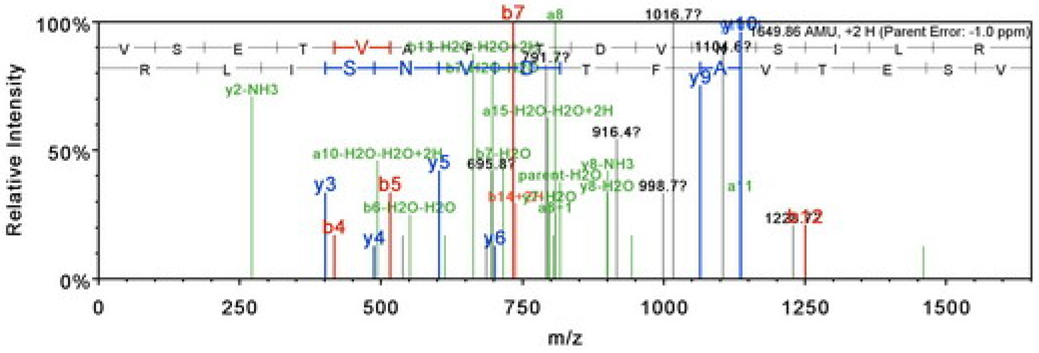

B	B Ions	B+2H	B-NH3	B-H2O	AA	Y Ions	Y+2H	Y-NH3	Y-H2O	Y
1	100.1				V	1650.9	825.9	1633.8	1632.9	15
2	187.1			169.1	S	1551.8	776.4	1534.8	1533.8	14
3	316.2			298.1	E	1464.8	732.9	1447.7	1446.8	13
4	417.2			399.2	T	1335.7	668.4	1318.7	1317.7	12
5	516.3			498.3	V	1234.7	617.8	1217.7	1216.7	11
6	587.3	294.2		569.3	A	1135.6	568.3	1118.6	1117.6	10
7	734.4	367.7		716.4	F	1064.6	532.8	1047.5	1046.6	9
8	835.4	418.2		817.4	T	917.5	459.3	900.5	899.5	8
9	950.5	475.7		932.4	D	816.5	408.7	799.4	798.5	7
10	1049.5	525.3		1031.5	V	701.4	351.2	684.4	683.4	6
11	1163.6	582.3	1146.5	1145.5	N	602.4		585.3	584.4	5
12	1250.6	625.8	1233.6	1232.6	S	488.3		471.3	470.3	4
13	1363.7	682.3	1346.7	1345.7	I	401.3		384.3		3
14	1476.8	738.9	1459.7	1458.8	L	288.2		271.2		2
15	1650.9	825.9	1633.8	1632.9	R	175.1		158.1		1

Gene symbol: Etfb
Protein name: Electron transfer flavoprotein subunit beta
Protein accession numbers: IPI00121440
Peptide sequence: (R)GIHVEIPGAQAESLGPLQVAR(V)
Exclusive (unique to this protein): TRUE
SEQUEST XCorr score: 3.28 SEQUEST DCn score: 0.685
Fix modifications: None

Variable modifications: None

charge: 2 Observed m/z: 1071.5848

Actual minus calculated peptide mass (AMU): -0.005371

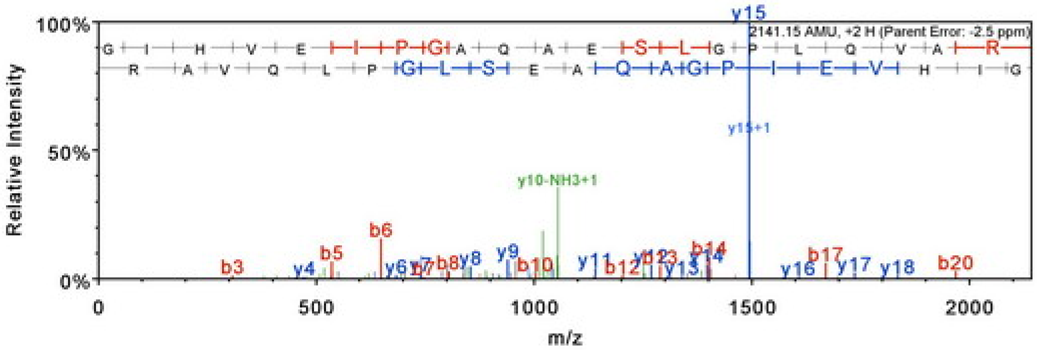

B	B Ions	B+2H	B-NH3	B-H2O	AA	Y Ions	Y+2H	Y-NH3	Y-H2O	Y
1	58.0				G	2142.2	1071.6	2125.1	2124.2	21
2	171.1				I	2085.2	1043.1	2068.1	2067.1	20
3	308.2	154.6			H	1972.1	986.5	1955.0	1954.1	19
4	407.2	204.1			V	1835.0	918.0	1818.0	1817.0	18
5	536.3	268.6		518.3	E	1735.9	868.5	1718.9	1717.9	17
6	649.4	325.2		631.4	I	1606.9	804.0	1589.9	1588.9	16
7	746.4	373.7		728.4	P	1493.8	747.4	1476.8	1475.8	15
8	803.4	402.2		785.4	G	1396.8	698.9	1379.7	1378.7	14
9	874.5	437.7		856.5	A	1339.7	670.4	1322.7	1321.7	13
10	1002.5	501.8	985.5	984.5	Q	1268.7	634.9	1251.7	1250.7	12
11	1073.6	537.3	1056.5	1055.6	A	1140.6	570.8	1123.6	1122.6	11
12	1202.6	601.8	1185.6	1184.6	E	1069.6	535.3	1052.6	1051.6	10
13	1289.7	645.3	1272.6	1271.6	S	940.6	470.8	923.5	922.6	9
14	1402.7	701.9	1385.7	1384.7	L	853.5	427.3	836.5		8
15	1459.8	730.4	1442.7	1441.7	G	740.4	370.7	723.4		7
16	1556.8	778.9	1539.8	1538.8	P	683.4	342.2	666.4		6
17	1669.9	835.5	1652.9	1651.9	L	586.4		569.3		5
18	1798.0	899.5	1780.9	1779.9	Q	473.3		456.3		4
19	1897.0	949.0	1880.0	1879.0	V	345.2		328.2		3
20	1968.1	984.5	1951.0	1950.1	A	246.2		229.1		2
21	2142.2	1071.6	2125.1	2124.2	R	175.1		158.1		1

Gene symbol: Fbxo38

Protein name: F-box only protein 38

Protein accession numbers: IPI00341601

Peptide sequence: (R)EVLPVDADEEQAGPSGLQR(V)

Exclusive (unique to this protein): TRUE

SEQUEST XCorr score: 2.1 SEQUEST DCn score: 0.548

Fix modifications: None

Variable modifications: None

charge: 2 Observed m/z: 1005.4883

Actual minus calculated peptide mass (AMU): -0.009399

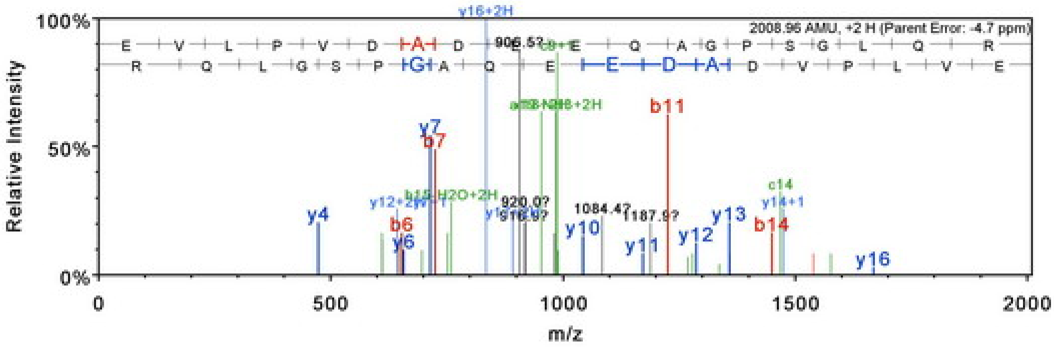

B	B Ions	B+2H	B-NH3	B-H2O	AA	Y Ions	Y+2H	Y-NH3	Y-H2O	Y
1	130.1			112.0	E	2010.0	1005.5	1993.0	1992.0	19
2	229.1			211.1	V	1880.9	941.0	1863.9	1862.9	18
3	342.2			324.2	L	1781.9	891.4	1764.8	1763.9	17
4	439.3			421.3	P	1668.8	834.9	1651.8	1650.8	16
5	538.3			520.3	V	1571.7	786.4	1554.7	1553.7	15
6	653.4	327.2		635.3	D	1472.7	736.8	1455.6	1454.7	14
7	724.4	362.7		706.4	A	1357.6	679.3	1340.6	1339.6	13
8	839.4	420.2		821.4	D	1286.6	643.8	1269.6	1268.6	12
9	968.5	484.7		950.5	E	1171.6	586.3	1154.5	1153.6	11
10	1097.5	549.3		1079.5	E	1042.5	521.8	1025.5	1024.5	10
11	1225.6	613.3	1208.5	1207.5	Q	913.5	457.3	896.5	895.5	9
12	1296.6	648.8	1279.6	1278.6	A	785.4	393.2	768.4	767.4	8
13	1353.6	677.3	1336.6	1335.6	G	714.4	357.7	697.4	696.4	7
14	1450.7	725.8	1433.6	1432.7	P	657.4	329.2	640.3	639.4	6
15	1537.7	769.4	1520.7	1519.7	S	560.3		543.3	542.3	5
16	1594.7	797.9	1577.7	1576.7	G	473.3		456.3		4
17	1707.8	854.4	1690.8	1689.8	L	416.3		399.2		3
18	1835.9	918.4	1818.8	1817.9	Q	303.2		286.2		2
19	2010.0	1005.5	1993.0	1992.0	R	175.1		158.1		1

Gene symbol: Fdft1

Protein name: Squalene synthetase

Protein accession numbers: IPI00134836,IPI00338068

Peptide sequence: (R)TQNLPNCQLISR(S)

Exclusive (unique to this protein): TRUE

SEQUEST XCorr score: 2.24 SEQUEST DCn score: 0.548

Fix modifications: C7: Carbamidomethyl (+57.02)Variable modifications: None

charge: 2 Observed m/z: 722.3715

Actual minus calculated peptide mass (AMU): -0.0028056

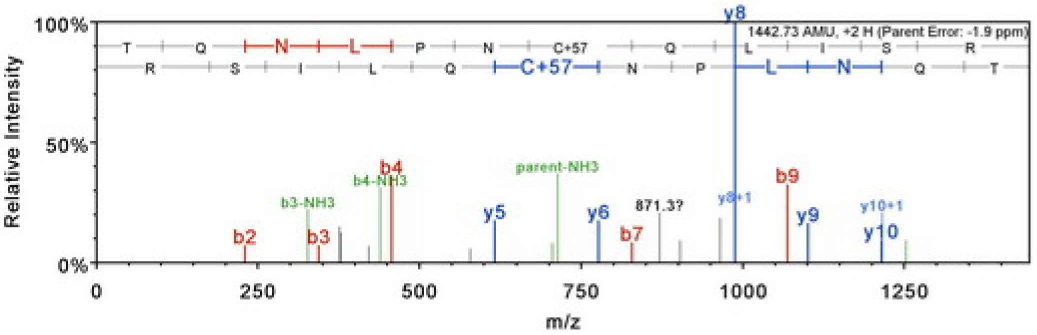

B	B Ions	B+2H	B-NH3	B-H2O	AA	Y Ions	Y+2H	Y-NH3	Y-H2O	Y
1	102.1			84.0	T	1443.7	722.4	1426.7	1425.7	12
2	230.1		213.1	212.1	Q	1342.7	671.9	1325.7	1324.7	11
3	344.2		327.1	326.2	N	1214.6	607.8	1197.6	1196.6	10
4	457.2		440.2	439.2	L	1100.6	550.8	1083.6	1082.6	9
5	554.3		537.3	536.3	P	987.5	494.3	970.5	969.5	8
6	668.3	334.7	651.3	650.3	N	890.5	445.7	873.4	872.4	7
7	828.4	414.7	811.3	810.4	C+57	776.4	388.7	759.4	758.4	6
8	956.4	478.7	939.4	938.4	Q	616.4		599.4	598.4	5
9	1069.5	535.3	1052.5	1051.5	L	488.3		471.3	470.3	4
10	1182.6	591.8	1165.6	1164.6	I	375.2		358.2	357.2	3
11	1269.6	635.3	1252.6	1251.6	S	262.1		245.1	244.1	2
12	1443.7	722.4	1426.7	1425.7	R	175.1		158.1		1

Gene symbol: Fh1

Protein name: Isoform Mitochondrial of Fumarate hydratase, mitochondrial precursor

Protein accession numbers: IPI00129928,IPI00759940

Peptide sequence: (R)IYELAAGGTAVGTGLNTR(I)

Exclusive (unique to this protein): TRUE

SEQUEST XCorr score: 2.62 SEQUEST DCn score: 0.488

Fix modifications: None

Variable modifications: None

charge: 2 Observed m/z: 882.4662

Actual minus calculated peptide mass (AMU): -0.004761

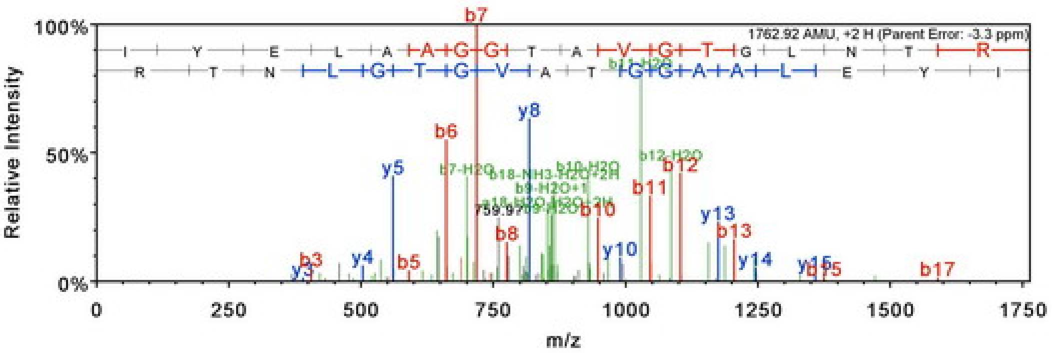

B	B Ions	B+2H	B-NH3	B-H2O	AA	Y Ions	Y+2H	Y-NH3	Y-H2O	Y
1	114.1				I	1763.9	882.5	1746.9	1745.9	18
2	277.2				Y	1650.9	825.9	1633.8	1632.8	17
3	406.2			388.2	E	1487.8	744.4	1470.8	1469.8	16
4	519.3			501.3	L	1358.7	679.9	1341.7	1340.7	15
5	590.3			572.3	A	1245.7	623.3	1228.6	1227.6	14
6	661.4	331.2		643.4	A	1174.6	587.8	1157.6	1156.6	13
7	718.4	359.7		700.4	G	1103.6	552.3	1086.5	1085.6	12
8	775.4	388.2		757.4	G	1046.6	523.8	1029.5	1028.5	11
9	876.5	438.7		858.4	T	989.5	495.3	972.5	971.5	10
10	947.5	474.3		929.5	A	888.5	444.8	871.5	870.5	9
11	1046.5	523.8		1028.5	V	817.5	409.2	800.4	799.4	8
12	1103.6	552.3		1085.6	G	718.4	359.7	701.4	700.4	7
13	1204.6	602.8		1186.6	T	661.4	331.2	644.3	643.4	6
14	1261.6	631.3		1243.6	G	560.3		543.3	542.3	5
15	1374.7	687.9		1356.7	L	503.3		486.3	485.3	4
16	1488.8	744.9	1471.7	1470.8	N	390.2		373.2	372.2	3
17	1589.8	795.4	1572.8	1571.8	T	276.2		259.1	258.2	2
18	1763.9	882.5	1746.9	1745.9	R	175.1		158.1		1

Gene symbol: Fis1

Protein name: Mitochondrial fission 1 protein

Protein accession numbers: IPI00132217

Peptide sequence: (R)GIVLLEELLPK(G)

Exclusive (unique to this protein): TRUE

SEQUEST XCorr score: 2.13 SEQUEST DCn score: 0.493

Fix modifications: None

Variable modifications: None

charge: 2 Observed m/z: 612.3849

Actual minus calculated peptide mass (AMU): 0.0001221

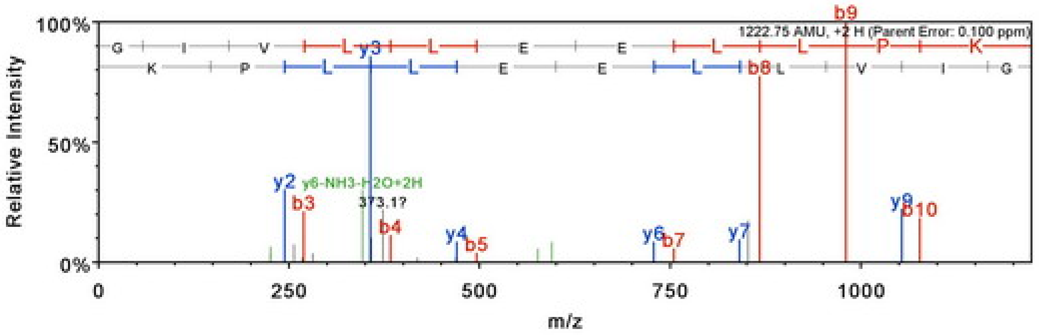

B	B Ions	B+2H	B-NH3	B-H2O	AA	Y Ions	Y+2H	Y-NH3	Y-H2O	Y
1	58.0				G	1223.8	612.4	1206.7	1205.8	11
2	171.1				I	1166.7	583.9	1149.7	1148.7	10
3	270.2				V	1053.7	527.3	1036.6	1035.7	9
4	383.3				L	954.6	477.8	937.6	936.6	8
5	496.4				L	841.5	421.3	824.5	823.5	7
6	625.4	313.2		607.4	E	728.4	364.7	711.4	710.4	6
7	754.4	377.7		736.4	E	599.4		582.4	581.4	5
8	867.5	434.3		849.5	L	470.3		453.3		4
9	980.6	490.8		962.6	L	357.3		340.2		3
10	1077.7	539.3		1059.7	P	244.2		227.1		2
11	1223.8	612.4	1206.7	1205.8	K	147.1		130.1		1

Gene symbol: Fkbp1a

Protein name: 14 days pregnant adult female placenta cDNA, RIKEN full-length enriched library, clone:I530022I01 product:FK506 binding protein 1a, full insert sequence

Protein accession numbers: IPI00653179

Peptide sequence: (R)GWEEGVAQMSVGQR(A)

Exclusive (unique to this protein): TRUE

SEQUEST XCorr score: 2.05 SEQUEST DCn score: 0.679

Fix modifications: None

Variable modifications: M9: Oxidation (+16.00)

charge: 2 Observed m/z: 775.3591

Actual minus calculated peptide mass (AMU): 0.0035358

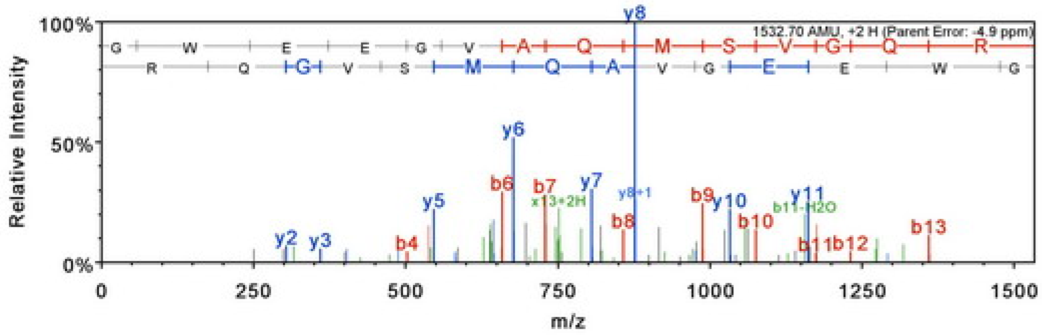

B	B Ions	B+2H	B-NH3	B-H2O	AA	Y Ions	Y+2H	Y-NH3	Y-H2O	Y
1	58.0				G	1533.7	767.4	1516.7	1515.7	14
2	244.1				W	1476.7	738.9	1459.7	1458.7	13
3	373.2			355.1	E	1290.6	645.8	1273.6	1272.6	12
4	502.2			484.2	E	1161.6	581.3	1144.5	1143.6	11
5	559.2			541.2	G	1032.5	516.8	1015.5	1014.5	10
6	658.3	329.7		640.3	V	975.5	488.3	958.5	957.5	9
7	729.3	365.2		711.3	A	876.4	438.7	859.4	858.4	8
8	857.4	429.2	840.4	839.4	Q	805.4	403.2	788.4	787.4	7
9	988.4	494.7	971.4	970.4	M	677.3	339.2	660.3	659.3	6
10	1075.5	538.2	1058.4	1057.4	S	546.3		529.3	528.3	5
11	1174.5	587.8	1157.5	1156.5	V	459.3		442.2		4
12	1231.5	616.3	1214.5	1213.5	G	360.2		343.2		3
13	1359.6	680.3	1342.6	1341.6	Q	303.2		286.2		2
14	1533.7	767.4	1516.7	1515.7	R	175.1		158.1		1

Gene symbol: Ganab
Protein name: Isoform 2 of Neutral alpha-glucosidase AB precursor
Protein accession numbers: IPI00115679
Peptide sequence: (R)LSFQHDPETSVLILR(K)
Exclusive (unique to this protein): TRUE
SEQUEST XCorr score: 2.71 SEQUEST DCn score: 0.583
Fix modifications: None
Variable modifications: None
charge: 2 Observed m/z: 877.9727
Actual minus calculated peptide mass (AMU): -0.0067152

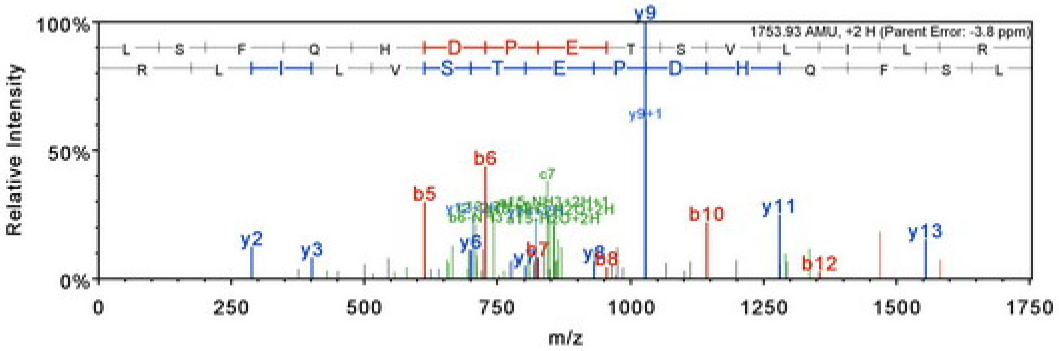

B	B Ions	B+2H	B-NH3	B-H2O	AA	Y Ions	Y+2H	Y-NH3	Y-H2O	Y
1	114.1				L	1754.9	878.0	1737.9	1736.9	15
2	201.1			183.1	S	1641.9	821.4	1624.8	1623.9	14
3	348.2			330.2	F	1554.8	777.9	1537.8	1536.8	13
4	476.3		459.2	458.2	Q	1407.8	704.4	1390.7	1389.8	12
5	613.3	307.2	596.3	595.3	H	1279.7	640.4	1262.7	1261.7	11
6	728.3	364.7	711.3	710.3	D	1142.6	571.8	1125.6	1124.6	10
7	825.4	413.2	808.4	807.4	P	1027.6	514.3	1010.6	1009.6	9
8	954.4	477.7	937.4	936.4	E	930.6	465.8	913.5	912.6	8
9	1055.5	528.2	1038.5	1037.5	T	801.5	401.3	784.5	783.5	7
10	1142.5	571.8	1125.5	1124.5	S	700.5	350.7	683.5	682.5	6
11	1241.6	621.3	1224.5	1223.6	V	613.4		596.4		5
12	1354.7	677.8	1337.6	1336.7	L	514.4		497.4		4
13	1467.8	734.4	1450.7	1449.7	I	401.3		384.3		3
14	1580.8	790.9	1563.8	1562.8	L	288.2		271.2		2
15	1754.9	878.0	1737.9	1736.9	R	175.1		158.1		1

Gene symbol: Gclm
Protein name: Glutamate--cysteine ligase regulatory subunit
Protein accession numbers: IPI00114329
Peptide sequence: (K)TLNEWSSQISPDLVR(E)
Exclusive (unique to this protein): TRUE
SEQUEST XCorr score: 2.03 SEQUEST DCn score: 0.484
Fix modifications: None

Variable modifications: None

charge: 2 Observed m/z: 872.949

Actual minus calculated peptide mass (AMU): 0.003052

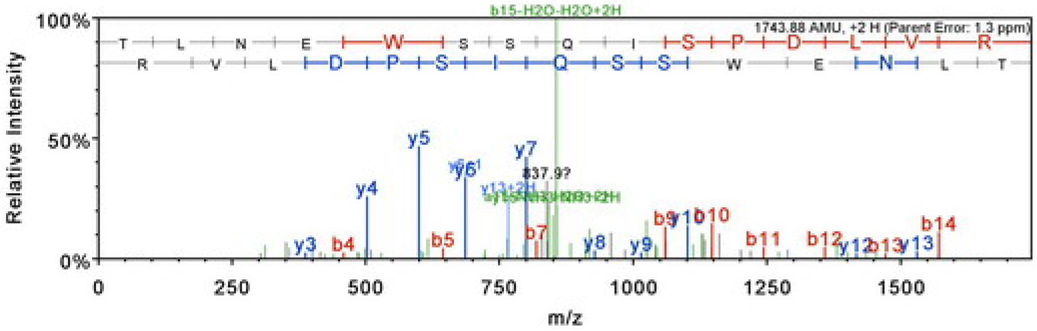

B	B Ions	B+2H	B-NH3	B-H2O	AA	Y Ions	Y+2H	Y-NH3	Y-H2O	Y
1	102.1			84.0	T	1744.9	873.0	1727.9	1726.9	15
2	215.1			197.1	L	1643.8	822.4	1626.8	1625.8	14
3	329.2		312.2	311.2	N	1530.8	765.9	1513.7	1512.7	13
4	458.2		441.2	440.2	E	1416.7	708.9	1399.7	1398.7	12
5	644.3		627.3	626.3	W	1287.7	644.3	1270.6	1269.7	11
6	731.3	366.2	714.3	713.3	S	1101.6	551.3	1084.6	1083.6	10
7	818.4	409.7	801.3	800.4	S	1014.6	507.8	997.5	996.6	9
8	946.4	473.7	929.4	928.4	Q	927.5	464.3	910.5	909.5	8
9	1059.5	530.3	1042.5	1041.5	I	799.5	400.2	782.4	781.5	7
10	1146.5	573.8	1129.5	1128.5	S	686.4	343.7	669.4	668.4	6
11	1243.6	622.3	1226.6	1225.6	P	599.4		582.3	581.3	5
12	1358.6	679.8	1341.6	1340.6	D	502.3		485.3	484.3	4
13	1471.7	736.4	1454.7	1453.7	L	387.3		370.3		3
14	1570.8	785.9	1553.8	1552.8	V	274.2		257.2		2
15	1744.9	873.0	1727.9	1726.9	R	175.1		158.1		1

Gene symbol: Gda

Protein name: Guanine deaminase

Protein accession numbers: IPI00469987

Peptide sequence: (R)TPPLALVFR(G)

Exclusive (unique to this protein): TRUE

SEQUEST XCorr score: 2.02 SEQUEST DCn score: 0.428

Fix modifications: None

Variable modifications: None

charge: 2 Observed m/z: 507.3086

Actual minus calculated peptide mass (AMU): -0.005554

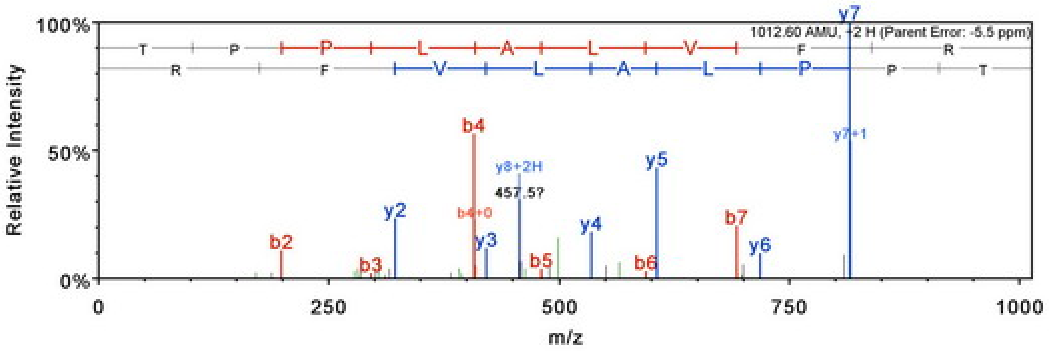

B	B Ions	B+2H	B-NH3	B-H2O	AA	Y Ions	Y+2H	Y-NH3	Y-H2O	Y
1	102.1			84.0	T	1013.6	507.3	996.6	995.6	9
2	199.1			181.1	P	912.6	456.8	895.5		8
3	296.2			278.2	P	815.5	408.3	798.5		7
4	409.3			391.2	L	718.5	359.7	701.4		6
5	480.3			462.3	A	605.4		588.4		5
6	593.4	297.2		575.4	L	534.3		517.3		4
7	692.4	346.7		674.4	V	421.3		404.2		3
8	839.5	420.3		821.5	F	322.2		305.2		2
9	1013.6	507.3	996.6	995.6	R	175.1		158.1		1

Gene symbol: Gmppb

Protein name: 2 days neonate thymus thymic cells cDNA, RIKEN full-length enriched library, clone:E430010H19 product:GDP-MANNOSE PYROPHOSPHORYLASE B homolog

Protein accession numbers: IPI00113992

Peptide sequence: (R)LYSGPGIVGNVLVDPSAR(I)

Exclusive (unique to this protein): TRUE

SEQUEST XCorr score: 2.56 SEQUEST DCn score: 0.583

Fix modifications: None

Variable modifications: None

charge: 2 Observed m/z: 907.492

Actual minus calculated peptide mass (AMU): -0.005127

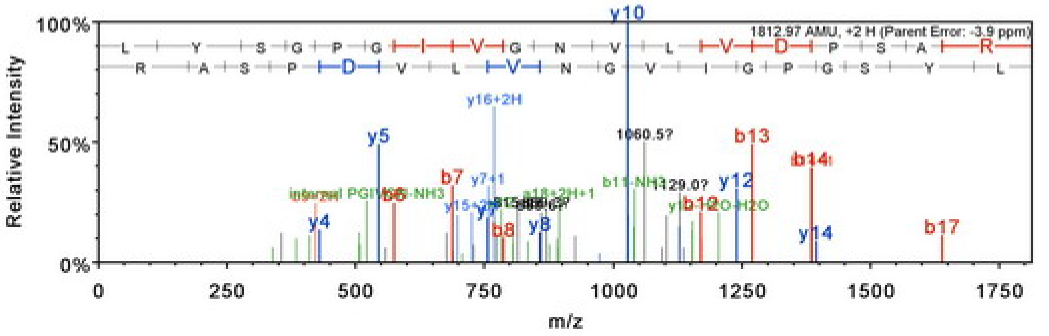

B	B Ions	B+2H	B-NH3	B-H2O	AA	Y Ions	Y+2H	Y-NH3	Y-H2O	Y
1	114.1				L	1814.0	907.5	1797.0	1796.0	18
2	277.2				Y	1700.9	851.0	1683.9	1682.9	17
3	364.2			346.2	S	1537.8	769.4	1520.8	1519.8	16
4	421.2			403.2	G	1450.8	725.9	1433.8	1432.8	15
5	518.3			500.3	P	1393.8	697.4	1376.8	1375.8	14
6	575.3	288.2		557.3	G	1296.7	648.9	1279.7	1278.7	13
7	688.4	344.7		670.4	I	1239.7	620.4	1222.7	1221.7	12
8	787.4	394.2		769.4	V	1126.6	563.8	1109.6	1108.6	11
9	844.5	422.7		826.5	G	1027.5	514.3	1010.5	1009.5	10
10	958.5	479.8	941.5	940.5	N	970.5	485.8	953.5	952.5	9
11	1057.6	529.3	1040.5	1039.6	V	856.5	428.8	839.5	838.5	8
12	1170.7	585.8	1153.6	1152.6	L	757.4	379.2	740.4	739.4	7
13	1269.7	635.4	1252.7	1251.7	V	644.3	322.7	627.3	626.3	6
14	1384.8	692.9	1367.7	1366.7	D	545.3		528.2	527.3	5
15	1481.8	741.4	1464.8	1463.8	P	430.2		413.2	412.2	4
16	1568.8	784.9	1551.8	1550.8	S	333.2		316.2	315.2	3
17	1639.9	820.4	1622.8	1621.9	A	246.2		229.1		2
18	1814.0	907.5	1797.0	1796.0	R	175.1		158.1		1

Gene symbol: Gnb1

Protein name: Guanine nucleotide-binding protein G(I)/G(S)/G(T) subunit beta 1

Protein accession numbers: IPI00120716

Peptide sequence: (K)ACADATLSQITNNIDPVGR(I)

Exclusive (unique to this protein): TRUE

SEQUEST XCorr score: 3.18　　SEQUEST DCn score: 0.6

Fix modifications: C2: Carbamidomethyl (+57.02)Variable modifications: None

charge: 2　　Observed m/z: 1008.4969

Actual minus calculated peptide mass (AMU): 0.0039054

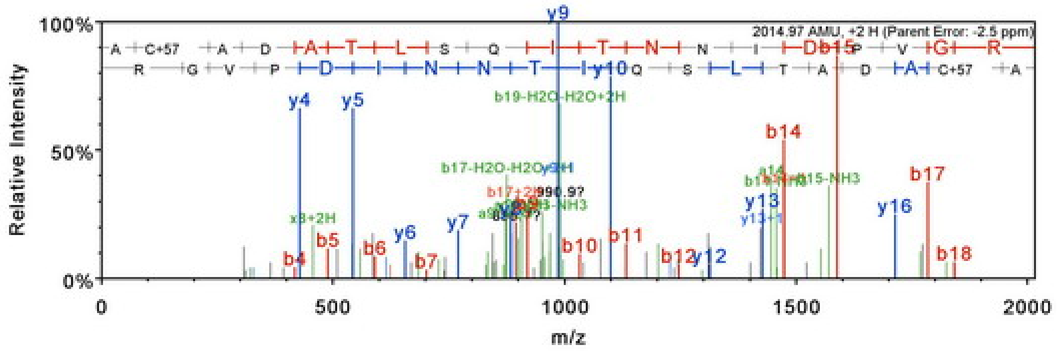

B	B Ions	B+2H	B-NH3	B-H2O	AA	Y Ions	Y+2H	Y-NH3	Y-H2O	Y
1	72.0				A	2016.0	1008.5	1999.0	1998.0	19
2	232.1				C+57	1945.0	973.0	1927.9	1926.9	18
3	303.1				A	1784.9	893.0	1767.9	1766.9	17
4	418.1			400.1	D	1713.9	857.4	1696.9	1695.9	16
5	489.2			471.2	A	1598.9	799.9	1581.8	1580.8	15
6	590.2	295.6		572.2	T	1527.8	764.4	1510.8	1509.8	14
7	703.3	352.2		685.3	L	1426.8	713.9	1409.7	1408.8	13
8	790.3	395.7		772.3	S	1313.7	657.3	1296.7	1295.7	12
9	918.4	459.7	901.4	900.4	Q	1226.7	613.8	1209.6	1208.6	11
10	1031.5	516.3	1014.5	1013.5	I	1098.6	549.8	1081.6	1080.6	10
11	1132.5	566.8	1115.5	1114.5	T	985.5	493.3	968.5	967.5	9
12	1246.6	623.8	1229.5	1228.6	N	884.5	442.7	867.4	866.5	8
13	1360.6	680.8	1343.6	1342.6	N	770.4	385.7	753.4	752.4	7
14	1473.7	737.4	1456.7	1455.7	I	656.4	328.7	639.4	638.4	6
15	1588.7	794.9	1571.7	1570.7	D	543.3		526.3	525.3	5
16	1685.8	843.4	1668.8	1667.8	P	428.3		411.2		4
17	1784.9	892.9	1767.8	1766.8	V	331.2		314.2		3
18	1841.9	921.4	1824.8	1823.9	G	232.1		215.1		2
19	2016.0	1008.5	1999.0	1998.0	R	175.1		158.1		1

Gene symbol: Grhpr

Protein name: Glyoxylate reductase/hydroxypyruvate reductase

Protein accession numbers: IPI00130530

Peptide sequence: (K)VFVTGPLPAEGR(A)

Exclusive (unique to this protein): TRUE

SEQUEST XCorr score: 2.2 SEQUEST DCn score: 0.543

Fix modifications: None

Variable modifications: None

charge: 2 Observed m/z: 621.8422

Actual minus calculated peptide mass (AMU): -0.008301

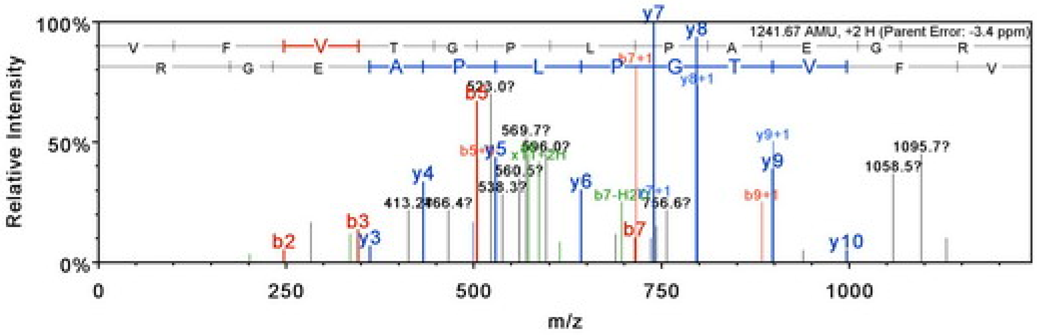

B	B Ions	B+2H	B-NH3	B-H2O	AA	Y Ions	Y+2H	Y-NH3	Y-H2O	Y
1	100.1				V	1242.7	621.9	1225.7	1224.7	12
2	247.1				F	1143.6	572.3	1126.6	1125.6	11
3	346.2				V	996.6	498.8	979.5	978.5	10
4	447.3			429.3	T	897.5	449.2	880.5	879.5	9
5	504.3			486.3	G	796.4	398.7	779.4	778.4	8
6	601.3	301.2		583.3	P	739.4	370.2	722.4	721.4	7
7	714.4	357.7		696.4	L	642.4	321.7	625.3	624.4	6
8	811.5	406.2		793.5	P	529.3		512.3	511.3	5
9	882.5	441.8		864.5	A	432.2		415.2	414.2	4
10	1011.6	506.3		993.5	E	361.2		344.2	343.2	3
11	1068.6	534.8		1050.6	G	232.1		215.1		2
12	1242.7	621.9	1225.7	1224.7	R	175.1		158.1		1

Gene symbol: Gsta4

Protein name: Glutathione S-transferase A4

Protein accession numbers: IPI00323911

Peptide sequence: (K)EKEESYDLILSR(A)

Exclusive (unique to this protein): TRUE

SEQUEST XCorr score: 2.88 SEQUEST DCn score: 0.663

Fix modifications: None

Variable modifications: None

charge: 2 Observed m/z: 741.3804

Actual minus calculated peptide mass (AMU): 0.004273

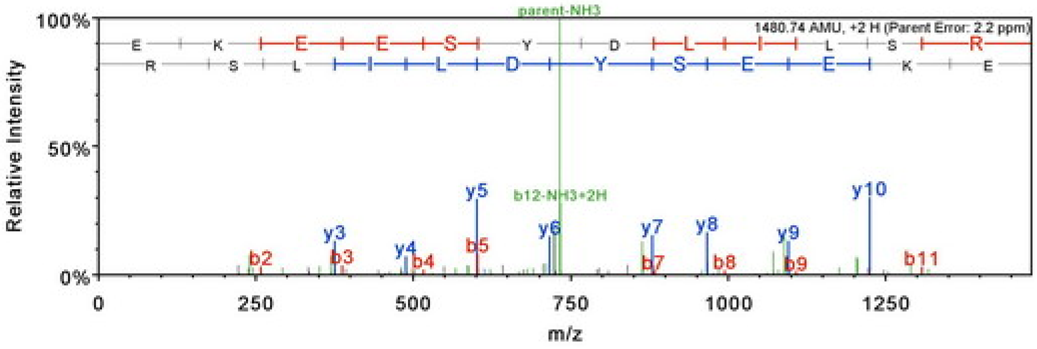

B	B Ions	B+2H	B-NH3	B-H2O	AA	Y Ions	Y+2H	Y-NH3	Y-H2O	Y
1	130.1			112.0	E	1481.8	741.4	1464.7	1463.7	12
2	258.1	129.6	241.1	240.1	K	1352.7	676.9	1335.7	1334.7	11
3	387.2	194.1	370.2	369.2	E	1224.6	612.8	1207.6	1206.6	10
4	516.2	258.6	499.2	498.2	E	1095.6	548.3	1078.5	1077.6	9
5	603.3	302.1	586.2	585.3	S	966.5	483.8	949.5	948.5	8
6	766.3	383.7	749.3	748.3	Y	879.5	440.3	862.5	861.5	7
7	881.4	441.2	864.3	863.3	D	716.4	358.7	699.4	698.4	6
8	994.4	497.7	977.4	976.4	L	601.4		584.4	583.4	5
9	1107.5	554.3	1090.5	1089.5	I	488.3		471.3	470.3	4
10	1220.6	610.8	1203.6	1202.6	L	375.2		358.2	357.2	3
11	1307.6	654.3	1290.6	1289.6	S	262.1		245.1	244.1	2
12	1481.8	741.4	1464.7	1463.7	R	175.1		158.1		1

Gene symbol: Guk1

Protein name: 18-day embryo whole body cDNA, RIKEN full-length enriched library, clone:1110048P09 product:guanylate kinase 1, full insert sequence

Protein accession numbers: IPI00228828

Peptide sequence: (R)ICVLDVDLQGVR(S)

Exclusive (unique to this protein): TRUE

SEQUEST XCorr score: 2.1 SEQUEST DCn score: 0.581

Fix modifications: C2: Carbamidomethyl (+57.02)Variable modifications: None

charge: 2 Observed m/z: 693.8704

Actual minus calculated peptide mass (AMU): -0.008789

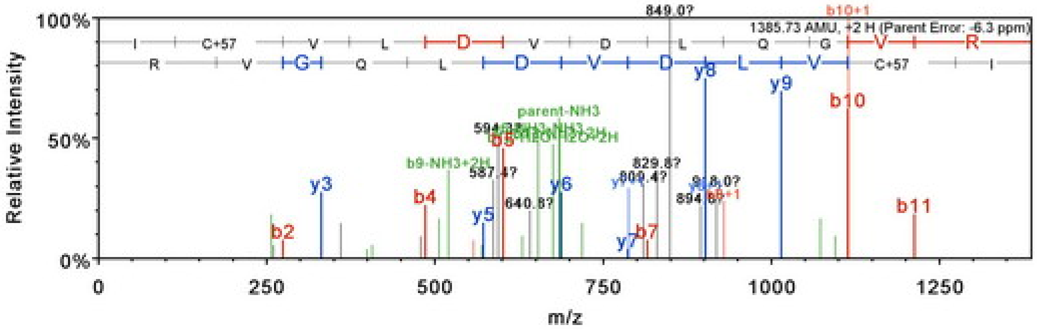

B	B Ions	B+2H	B-NH3	B-H2O	AA	Y Ions	Y+2H	Y-NH3	Y-H2O	Y
1	114.1				I	1386.7	693.9	1369.7	1368.7	12
2	274.1				C+57	1273.7	637.3	1256.6	1255.7	11
3	373.2				V	1113.6	557.3	1096.6	1095.6	10
4	486.3				L	1014.6	507.8	997.5	996.6	9
5	601.3			583.3	D	901.5	451.2	884.5	883.5	8
6	700.4	350.7		682.4	V	786.5	393.7	769.4	768.4	7
7	815.4	408.2		797.4	D	687.4	344.2	670.4	669.4	6
8	928.5	464.7		910.5	L	572.4		555.3		5
9	1056.5	528.8	1039.5	1038.5	Q	459.3		442.2		4
10	1113.6	557.3	1096.5	1095.5	G	331.2		314.2		3
11	1212.6	606.8	1195.6	1194.6	V	274.2		257.2		2
12	1386.7	693.9	1369.7	1368.7	R	175.1		158.1		1

Gene symbol: Gyg

Protein name: Glycogenin-1

Protein accession numbers: IPI00264062

Peptide sequence: (R)MVVLTSPQVSDSMR(K)

Exclusive (unique to this protein): TRUE

SEQUEST XCorr score: 2 SEQUEST DCn score: 0.525

Fix modifications: None

Variable modifications: None

charge: 2 Observed m/z: 775.3872

Actual minus calculated peptide mass (AMU): -0.005249

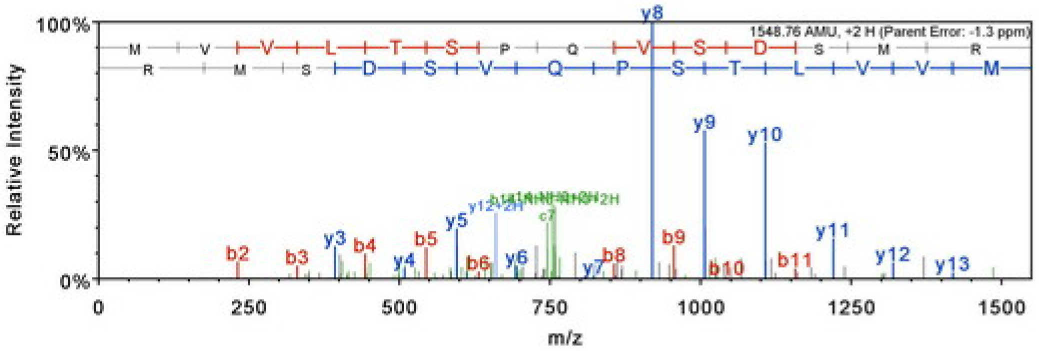

B	B Ions	B+2H	B-NH3	B-H2O	AA	Y Ions	Y+2H	Y-NH3	Y-H2O	Y
1	132.1				M	1549.8	775.4	1532.8	1531.8	14
2	231.1				V	1418.7	709.9	1401.7	1400.7	13
3	330.2				V	1319.7	660.3	1302.6	1301.7	12
4	443.3				L	1220.6	610.8	1203.6	1202.6	11
5	544.3			526.3	T	1107.5	554.3	1090.5	1089.5	10
6	631.4	316.2		613.3	S	1006.5	503.7	989.4	988.5	9
7	728.4	364.7		710.4	P	919.4	460.2	902.4	901.4	8
8	856.5	428.7	839.4	838.5	Q	822.4	411.7	805.4	804.4	7
9	955.5	478.3	938.5	937.5	V	694.3	347.7	677.3	676.3	6
10	1042.6	521.8	1025.5	1024.5	S	595.3		578.2	577.2	5
11	1157.6	579.3	1140.6	1139.6	D	508.2		491.2	490.2	4
12	1244.6	622.8	1227.6	1226.6	S	393.2		376.2	375.2	3
13	1375.7	688.3	1358.6	1357.7	M	306.2		289.1		2
14	1549.8	775.4	1532.8	1531.8	R	175.1		158.1		1

Gene symbol: H2-Ke6

Protein name: Isoform Short of Estradiol 17-beta-dehydrogenase 8

Protein accession numbers: IPI00115598,IPI00230550

Peptide sequence: (R)LAAEGAAVAACDLDGAAAQDTVR(L)

Exclusive (unique to this protein): TRUE

SEQUEST XCorr score: 3.16 SEQUEST DCn score: 0.59

Fix modifications: C11: Carbamidomethyl (+57.02)Variable modifications: None

charge: 2 Observed m/z: 1108.5385

Actual minus calculated peptide mass (AMU): 0.0070758

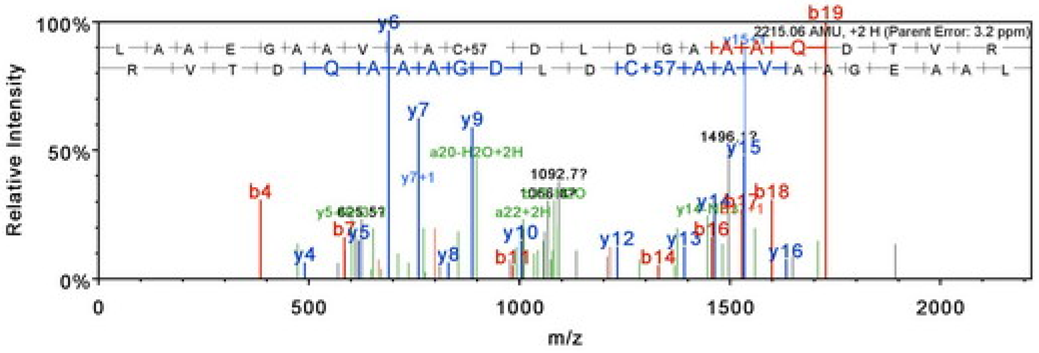

B	B Ions	B+2H	B-NH3	B-H2O	AA	Y Ions	Y+2H	Y-NH3	Y-H2O	Y
1	114.1				L	2216.1	1108.5	2199.0	2198.1	23
2	185.1				A	2103.0	1052.0	2085.9	2085.0	22
3	256.2				A	2031.9	1016.5	2014.9	2013.9	21
4	385.2			367.2	E	1960.9	981.0	1943.9	1942.9	20
5	442.2			424.2	G	1831.9	916.4	1814.8	1813.9	19
6	513.3	257.1		495.3	A	1774.8	887.9	1757.8	1756.8	18
7	584.3	292.7		566.3	A	1703.8	852.4	1686.8	1685.8	17
8	683.4	342.2		665.4	V	1632.8	816.9	1615.7	1614.8	16
9	754.4	377.7		736.4	A	1533.7	767.4	1516.7	1515.7	15
10	825.5	413.2		807.4	A	1462.7	731.8	1445.6	1444.7	14
11	985.5	493.2		967.5	C+57	1391.6	696.3	1374.6	1373.6	13
12	1100.5	550.8		1082.5	D	1231.6	616.3	1214.6	1213.6	12
13	1213.6	607.3		1195.6	L	1116.6	558.8	1099.5	1098.5	11
14	1328.6	664.8		1310.6	D	1003.5	502.2	986.5	985.5	10
15	1385.6	693.3		1367.6	G	888.5	444.7	871.4	870.4	9
16	1456.7	728.8		1438.7	A	831.4	416.2	814.4	813.4	8
17	1527.7	764.4		1509.7	A	760.4	380.7	743.4	742.4	7
18	1598.8	799.9		1580.7	A	689.4	345.2	672.3	671.4	6
19	1726.8	863.9	1709.8	1708.8	Q	618.3		601.3	600.3	5
20	1841.8	921.4	1824.8	1823.8	D	490.3		473.2	472.3	4
21	1942.9	971.9	1925.9	1924.9	T	375.2		358.2	357.2	3
22	2042.0	1021.5	2024.9	2023.9	V	274.2		257.2		2
23	2216.1	1108.5	2199.0	2198.1	R	175.1		158.1		1

Gene symbol: Hadh

Protein name: Hydroxyacyl-coenzyme A dehydrogenase, mitochondrial precursor

Protein accession numbers: IPI00121105

Peptide sequence: (R)LLVPYLIEAVR(L)

Exclusive (unique to this protein): TRUE

SEQUEST XCorr score: 2.05 SEQUEST DCn score: 0.632

Fix modifications: None

Variable modifications: None

charge: 2 Observed m/z: 643.3992

Actual minus calculated peptide mass (AMU): 0.0021955

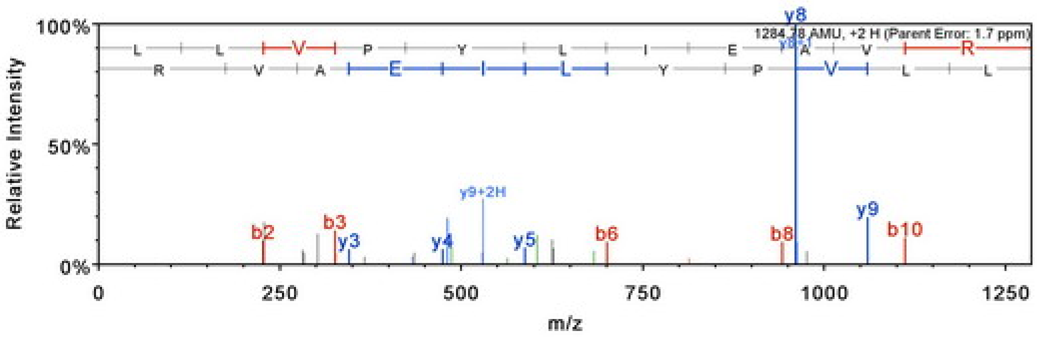

B	B Ions	B+2H	B-NH3	B-H2O	AA	Y Ions	Y+2H	Y-NH3	Y-H2O	Y
1	114.1				L	1285.8	643.4	1268.8	1267.8	11
2	227.2				L	1172.7	586.9	1155.7	1154.7	10
3	326.2				V	1059.6	530.3	1042.6	1041.6	9
4	423.3				P	960.6	480.8	943.5	942.5	8
5	586.4				Y	863.5	432.3	846.5	845.5	7
6	699.4	350.2			L	700.4	350.7	683.4	682.4	6
7	812.5	406.8			I	587.4		570.3	569.3	5
8	941.6	471.3		923.6	E	474.3		457.2	456.3	4
9	1012.6	506.8		994.6	A	345.2		328.2		3
10	1111.7	556.3		1093.7	V	274.2		257.2		2
11	1285.8	643.4	1268.8	1267.8	R	175.1		158.1		1

Gene symbol: Hibadh

Protein name: 3-hydroxyisobutyrate dehydrogenase, mitochondrial precursor

Protein accession numbers: IPI00116222

Peptide sequence: (K)TPILLGSLAHQIYR(M)

Exclusive (unique to this protein): TRUE

SEQUEST XCorr score: 2.15 SEQUEST DCn score: 0.514

Fix modifications: None

Variable modifications: None

charge: 2 Observed m/z: 791.4611

Actual minus calculated peptide mass (AMU): 0.002319

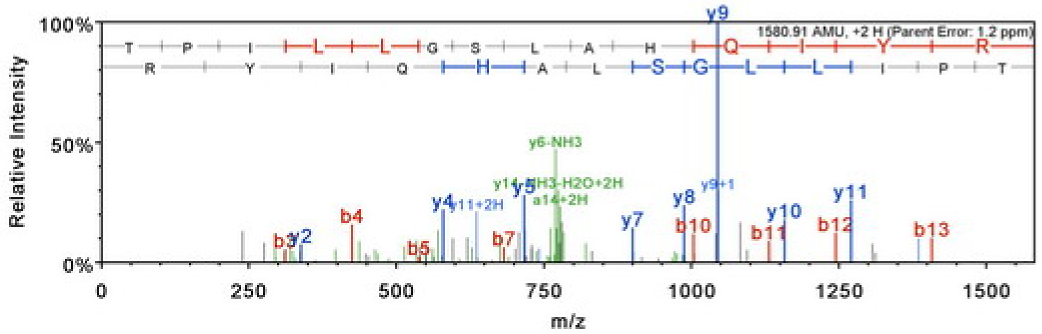

B	B Ions	B+2H	B-NH3	B-H2O	AA	Y Ions	Y+2H	Y-NH3	Y-H2O	Y
1	102.1			84.0	T	1581.9	791.5	1564.9	1563.9	14
2	199.1			181.1	P	1480.9	740.9	1463.8	1462.9	13
3	312.2			294.2	I	1383.8	692.4	1366.8	1365.8	12
4	425.3			407.3	L	1270.7	635.9	1253.7	1252.7	11
5	538.4			520.4	L	1157.6	579.3	1140.6	1139.6	10
6	595.4	298.2		577.4	G	1044.6	522.8	1027.5	1026.5	9
7	682.4	341.7		664.4	S	987.5	494.3	970.5	969.5	8
8	795.5	398.3		777.5	L	900.5	450.8	883.5		7
9	866.5	433.8		848.5	A	787.4	394.2	770.4		6
10	1003.6	502.3		985.6	H	716.4	358.7	699.4		5
11	1131.7	566.3	1114.6	1113.6	Q	579.3		562.3		4
12	1244.7	622.9	1227.7	1226.7	I	451.3		434.2		3
13	1407.8	704.4	1390.8	1389.8	Y	338.2		321.2		2
14	1581.9	791.5	1564.9	1563.9	R	175.1		158.1		1

Gene symbol: Hint1

Protein name: Histidine triad nucleotide-binding protein 1

Protein accession numbers: IPI00108189

Peptide sequence: (K)AQVAQPGGDTIFGK(I)

Exclusive (unique to this protein): TRUE

SEQUEST XCorr score: 2.32 SEQUEST DCn score: 0.626

Fix modifications: None

Variable modifications: None

charge: 2 Observed m/z: 694.8636

Actual minus calculated peptide mass (AMU): 0.001831

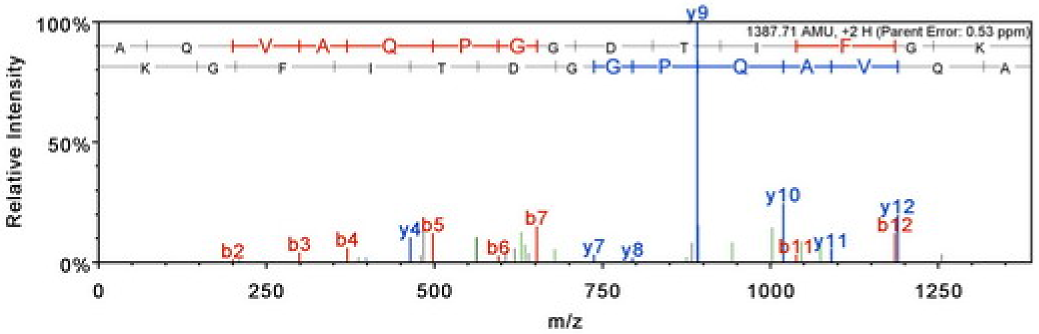

B	B Ions	B+2H	B-NH3	B-H2O	AA	Y Ions	Y+2H	Y-NH3	Y-H2O	Y
1	72.0				A	1388.7	694.9	1371.7	1370.7	14
2	200.1		183.1		Q	1317.7	659.3	1300.7	1299.7	13
3	299.2		282.2		V	1189.6	595.3	1172.6	1171.6	12
4	370.2		353.2		A	1090.5	545.8	1073.5	1072.5	11
5	498.3		481.2		Q	1019.5	510.3	1002.5	1001.5	10
6	595.3	298.2	578.3		P	891.5	446.2	874.4	873.5	9
7	652.3	326.7	635.3		G	794.4	397.7	777.4	776.4	8
8	709.4	355.2	692.3		G	737.4	369.2	720.4	719.4	7
9	824.4	412.7	807.4	806.4	D	680.4	340.7	663.3	662.4	6
10	925.4	463.2	908.4	907.4	T	565.3		548.3	547.3	5
11	1038.5	519.8	1021.5	1020.5	I	464.3		447.3		4
12	1185.6	593.3	1168.6	1167.6	F	351.2		334.2		3
13	1242.6	621.8	1225.6	1224.6	G	204.1		187.1		2
14	1388.7	694.9	1371.7	1370.7	K	147.1		130.1		1

Gene symbol: Hist1h2bl;Hist1h2bf;Hist1h2bj;Hist1h2bn;RP23-38E20.1;Hist1h2be;OTTMUSG00000013203;Hist1h2bc;Hist1h2bg;Hist1h2bb

Protein name: similar to Histone H2B F

Protein accession numbers: IPI00461514,IPI00752556

Peptide sequence: (K)AMGIMNSFVNDIFER(I)

Exclusive (unique to this protein): TRUE

SEQUEST XCorr score: 2.39 SEQUEST DCn score: 0.752

Fix modifications: None

Variable modifications: None

charge: 2 Observed m/z: 872.4142

Actual minus calculated peptide mass (AMU): 0.0008545

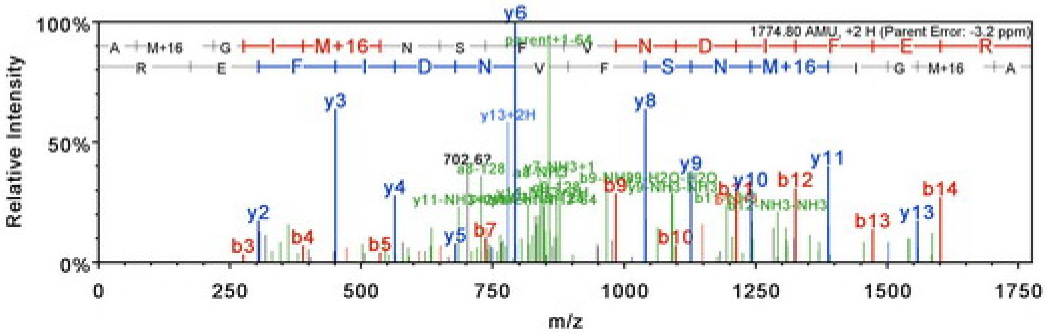

B	B Ions	B+2H	B-NH3	B-H2O	AA	Y Ions	Y+2H	Y-NH3	Y-H2O	Y
1	72.0				A	1775.8	888.4	1758.8	1757.8	15
2	219.1				M+16	1704.8	852.9	1687.8	1686.8	14
3	276.1				G	1557.7	779.4	1540.7	1539.7	13
4	389.2				I	1500.7	750.9	1483.7	1482.7	12
5	536.2				M+16	1387.6	694.3	1370.6	1369.6	11
6	650.3	325.6	633.2		N	1240.6	620.8	1223.6	1222.6	10
7	737.3	369.2	720.3	719.3	S	1126.5	563.8	1109.5	1108.5	9
8	884.4	442.7	867.3	866.4	F	1039.5	520.3	1022.5	1021.5	8
9	983.4	492.2	966.4	965.4	V	892.5	446.7	875.4	874.4	7
10	1097.5	549.2	1080.5	1079.5	N	793.4	397.2	776.4	775.4	6
11	1212.5	606.8	1195.5	1194.5	D	679.3		662.3	661.3	5
12	1325.6	663.3	1308.6	1307.6	I	564.3		547.3	546.3	4
13	1472.7	736.8	1455.6	1454.6	F	451.2		434.2	433.2	3
14	1601.7	801.4	1584.7	1583.7	E	304.2		287.1	286.2	2
15	1775.8	888.4	1758.8	1757.8	R	175.1		158.1		1

Gene symbol: Hmox2

Protein name: Heme oxygenase 2

Protein accession numbers: IPI00309322

Peptide sequence: (K)GTLGGSNCPFQTTVAVLR(K)

Exclusive (unique to this protein): TRUE

SEQUEST XCorr score: 2.24 SEQUEST DCn score: 0.479

Fix modifications: C8: Carbamidomethyl (+57.02)Variable modifications: None

charge: 2 Observed m/z: 939.4814

Actual minus calculated peptide mass (AMU): 0.0004883

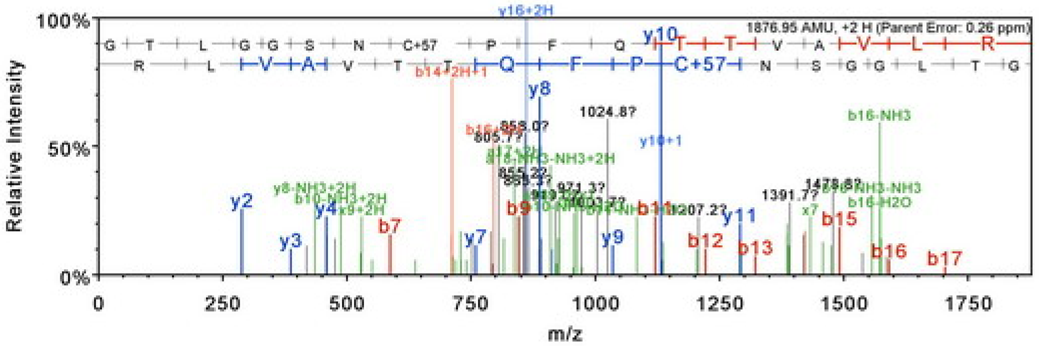

B	B Ions	B+2H	B-NH3	B-H2O	AA	Y Ions	Y+2H	Y-NH3	Y-H2O	Y
1	58.0				G	1878.0	939.5	1860.9	1859.9	18
2	159.1			141.1	T	1820.9	911.0	1803.9	1802.9	17
3	272.2			254.2	L	1719.9	860.5	1702.9	1701.9	16
4	329.2			311.2	G	1606.8	803.9	1589.8	1588.8	15
5	386.2			368.2	G	1549.8	775.4	1532.8	1531.8	14
6	473.2	237.1		455.2	S	1492.8	746.9	1475.7	1474.8	13
7	587.3	294.1	570.3	569.3	N	1405.7	703.4	1388.7	1387.7	12
8	747.3	374.2	730.3	729.3	C+57	1291.7	646.4	1274.7	1273.7	11
9	844.4	422.7	827.3	826.4	P	1131.7	566.3	1114.6	1113.6	10
10	991.4	496.2	974.4	973.4	F	1034.6	517.8	1017.6	1016.6	9
11	1119.5	560.3	1102.5	1101.5	Q	887.5	444.3	870.5	869.5	8
12	1220.5	610.8	1203.5	1202.5	T	759.5	380.2	742.5	741.5	7
13	1321.6	661.3	1304.6	1303.6	T	658.4	329.7	641.4	640.4	6
14	1420.7	710.8	1403.6	1402.6	V	557.4		540.4		5
15	1491.7	746.4	1474.7	1473.7	A	458.3		441.3		4
16	1590.8	795.9	1573.7	1572.8	V	387.3		370.3		3
17	1703.8	852.4	1686.8	1685.8	L	288.2		271.2		2
18	1878.0	939.5	1860.9	1859.9	R	175.1		158.1		1

Gene symbol: Hpca

Protein name: Neuron-specific calcium-binding protein hippocalcin

Protein accession numbers: IPI00230310,IPI00230449

Peptide sequence: (R)TFDTNSDGTIDFR(E)

Exclusive (unique to this protein): TRUE

SEQUEST XCorr score: 2.09 SEQUEST DCn score: 0.809

Fix modifications: None

Variable modifications: None

charge: 2 Observed m/z: 744.8345

Actual minus calculated peptide mass (AMU): 0.0003662

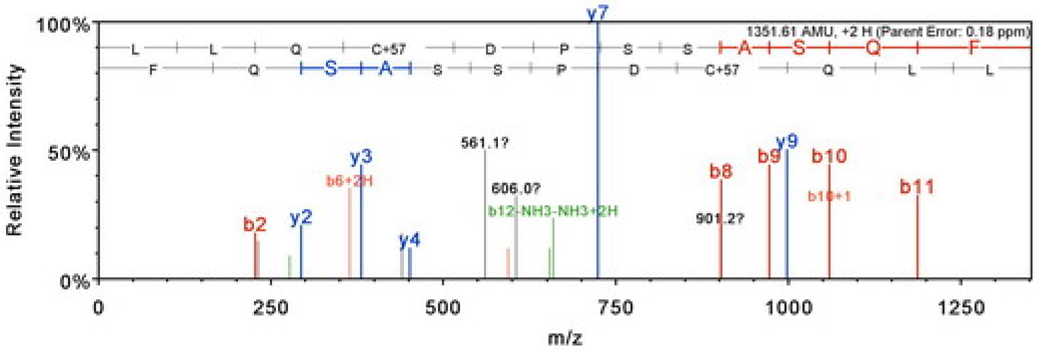

B	B Ions	B+2H	B-NH3	B-H2O	AA	Y Ions	Y+2H	Y-NH3	Y-H2O	Y
1	114.1				L	1352.6		1335.6	1334.6	12
2	227.2				L	1239.5		1222.5	1221.5	11
3	355.2		338.2		Q	1126.5		1109.4	1108.4	10
4	515.3		498.2		C+57	998.4		981.4	980.4	9
5	630.3		613.3	612.3	D	838.4		821.3	820.4	8
6	727.4	364.2	710.3	709.3	P	723.3		706.3	705.3	7
7	814.4	407.7	797.4	796.4	S	626.3		609.3	608.3	6
8	901.4	451.2	884.4	883.4	S	539.3		522.2	521.2	5
9	972.5	486.7	955.4	954.4	A	452.2		435.2	434.2	4
10	1059.5	530.2	1042.5	1041.5	S	381.2		364.2	363.2	3
11	1187.5	594.3	1170.5	1169.5	Q	294.2		277.1		2
12	1352.6	676.8	1335.6	1334.6	F	166.1				1

Gene symbol: Hsd17b12

Protein name: Isoform 1 of Estradiol 17-beta-dehydrogenase 12

Protein accession numbers: IPI00119219,IPI00458208

Peptide sequence: (R)AFQVWCVGNEALVGPR(L)

Exclusive (unique to this protein): TRUE

SEQUEST XCorr score: 2.01 SEQUEST DCn score: 0.623

Fix modifications: C6: Carbamidomethyl (+57.02)Variable modifications: None

charge: 2 Observed m/z: 901.9552

Actual minus calculated peptide mass (AMU): 0.001343

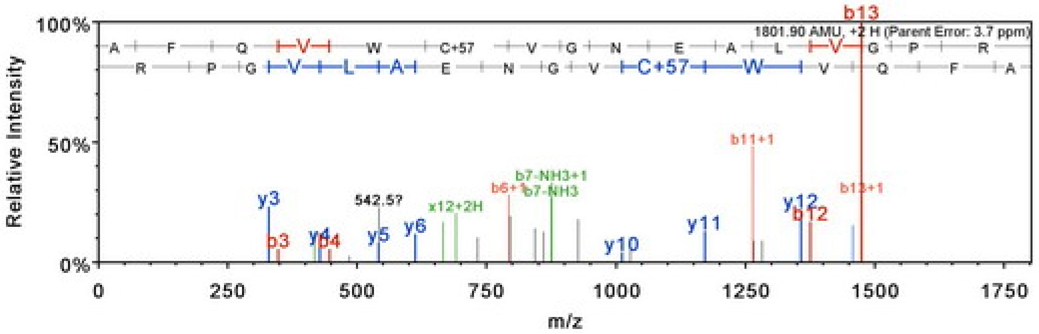

B	B Ions	B+2H	B-NH3	B-H2O	AA	Y Ions	Y+2H	Y-NH3	Y-H2O	Y
1	72.0				A	1802.9	902.0	1785.9	1784.9	16
2	219.1				F	1731.9	866.4	1714.8	1713.9	15
3	347.2		330.2		Q	1584.8	792.9	1567.8	1566.8	14
4	446.2		429.2		V	1456.7	728.9	1439.7	1438.7	13
5	632.3		615.3		W	1357.7	679.3	1340.6	1339.7	12
6	792.4	396.7	775.3		C+57	1171.6	586.3	1154.6	1153.6	11
7	891.4	446.2	874.4		V	1011.6	506.3	994.5	993.6	10
8	948.4	474.7	931.4		G	912.5	456.8	895.5	894.5	9
9	1062.5	531.8	1045.5		N	855.5	428.2	838.4	837.5	8
10	1191.5	596.3	1174.5	1173.5	E	741.4	371.2	724.4	723.4	7
11	1262.6	631.8	1245.5	1244.5	A	612.4	306.7	595.4		6
12	1375.7	688.3	1358.6	1357.6	L	541.4		524.3		5
13	1474.7	737.9	1457.7	1456.7	V	428.3		411.2		4
14	1531.7	766.4	1514.7	1513.7	G	329.2		312.2		3
15	1628.8	814.9	1611.8	1610.8	P	272.2		255.2		2
16	1802.9	902.0	1785.9	1784.9	R	175.1		158.1		1

Gene symbol: Hspa9

Protein name: Stress-70 protein, mitochondrial precursor

Protein accession numbers: IPI00133903

Peptide sequence: (R)QATKDAGQISGLNVLR(V)

Exclusive (unique to this protein): TRUE

SEQUEST XCorr score: 2.28 SEQUEST DCn score: 0.395

Fix modifications: None

Variable modifications: None

charge: 2 Observed m/z: 835.9622

Actual minus calculated peptide mass (AMU): -0.002441

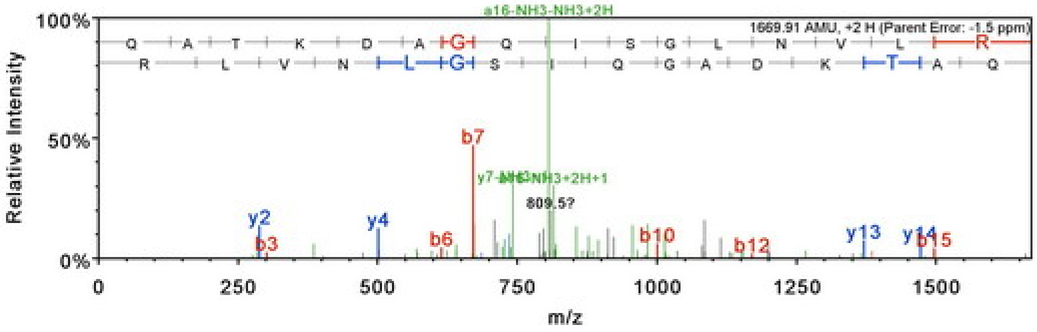

B	B Ions	B+2H	B-NH3	B-H2O	AA	Y Ions	Y+2H	Y-NH3	Y-H2O	Y
1	129.1		112.0		Q	1670.9	836.0	1653.9	1652.9	16
2	200.1		183.1		A	1542.9	771.9	1525.8	1524.9	15
3	301.2		284.1	283.1	T	1471.8	736.4	1454.8	1453.8	14
4	429.3	215.1	412.2	411.2	K	1370.8	685.9	1353.8	1352.8	13
5	544.3	272.6	527.3	526.3	D	1242.7	621.8	1225.7	1224.7	12
6	615.3	308.2	598.3	597.3	A	1127.7	564.3	1110.6	1109.6	11
7	672.3	336.7	655.3	654.3	G	1056.6	528.8	1039.6	1038.6	10
8	800.4	400.7	783.4	782.4	Q	999.6	500.3	982.6	981.6	9
9	913.5	457.2	896.5	895.5	I	871.5	436.3	854.5	853.5	8
10	1000.5	500.8	983.5	982.5	S	758.5	379.7	741.4	740.4	7
11	1057.5	529.3	1040.5	1039.5	G	671.4	336.2	654.4		6
12	1170.6	585.8	1153.6	1152.6	L	614.4		597.4		5
13	1284.7	642.8	1267.6	1266.6	N	501.3		484.3		4
14	1383.7	692.4	1366.7	1365.7	V	387.3		370.3		3
15	1496.8	748.9	1479.8	1478.8	L	288.2		271.2		2
16	1670.9	836.0	1653.9	1652.9	R	175.1		158.1		1

Gene symbol: Htra2

Protein name: Serine protease HTRA2, mitochondrial precursor

Protein accession numbers: IPI00275992,IPI00830249,IPI00830364,IPI00831347

Peptide sequence: (K)LAQNAEDVYEAVR(T)

Exclusive (unique to this protein): TRUE

SEQUEST XCorr score: 2.34 SEQUEST DCn score: 0.678

Fix modifications: None

Variable modifications: None

charge: 2 Observed m/z: 739.3662

Actual minus calculated peptide mass (AMU): -0.0040256

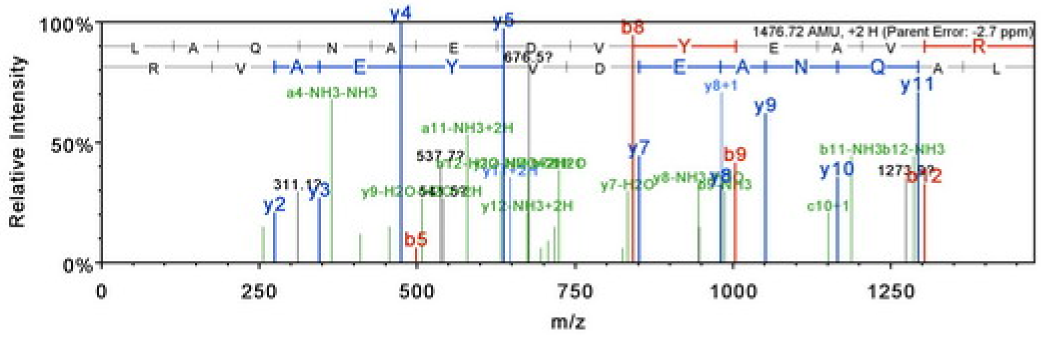

B	B Ions	B+2H	B-NH3	B-H2O	AA	Y Ions	Y+2H	Y-NH3	Y-H2O	Y
1	114.1				L	1477.7	739.4	1460.7	1459.7	13
2	185.1				A	1364.6	682.8	1347.6	1346.6	12
3	313.2		296.2		Q	1293.6	647.3	1276.6	1275.6	11
4	427.2		410.2		N	1165.5	583.3	1148.5	1147.5	10
5	498.3		481.2		A	1051.5	526.3	1034.5	1033.5	9
6	627.3	314.2	610.3	609.3	E	980.5	490.7	963.4	962.5	8
7	742.3	371.7	725.3	724.3	D	851.4	426.2	834.4	833.4	7
8	841.4	421.2	824.4	823.4	V	736.4	368.7	719.4	718.4	6
9	1004.5	502.7	987.4	986.5	Y	637.3		620.3	619.3	5
10	1133.5	567.3	1116.5	1115.5	E	474.3		457.2	456.3	4
11	1204.5	602.8	1187.5	1186.5	A	345.2		328.2		3
12	1303.6	652.3	1286.6	1285.6	V	274.2		257.2		2
13	1477.7	739.4	1460.7	1459.7	R	175.1		158.1		1

Gene symbol: Iigp1

Protein name: Interferon-inducible GTPase 1

Protein accession numbers: IPI00323496,IPI00849310

Peptide sequence: (R)LARDWEIEVDQVEAMIK(S)

Exclusive (unique to this protein): TRUE

SEQUEST XCorr score: 2 SEQUEST DCn score: 0.133

Fix modifications: None

Variable modifications: None

charge: 2 Observed m/z: 1023.0154

Actual minus calculated peptide mass (AMU): -0.01505

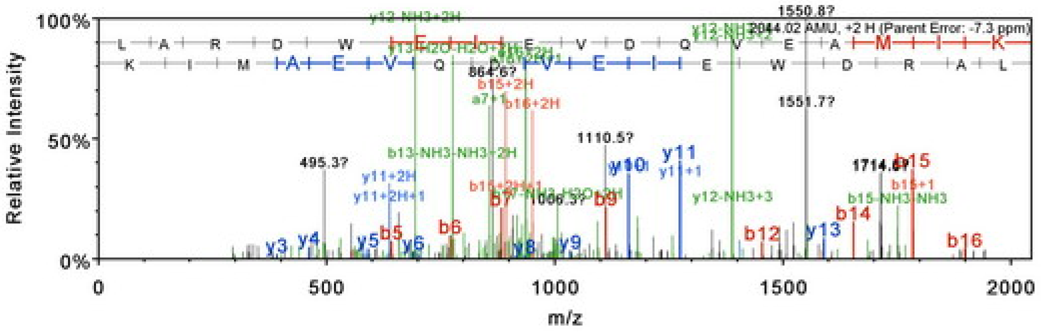

B	B Ions	B+2H	B-NH3	B-H2O	AA	Y Ions	Y+2H	Y-NH3	Y-H2O	Y
1	114.1				L	2045.0	1023.0	2028.0	2027.0	17
2	185.1				A	1932.0	966.5	1914.9	1913.9	16
3	341.2	171.1	324.2		R	1860.9	931.0	1843.9	1842.9	15
4	456.3	228.6	439.2	438.3	D	1704.8	852.9	1687.8	1686.8	14
5	642.3	321.7	625.3	624.3	W	1589.8	795.4	1572.8	1571.8	13
6	771.4	386.2	754.4	753.4	E	1403.7	702.4	1386.7	1385.7	12
7	884.5	442.7	867.4	866.5	I	1274.7	637.8	1257.6	1256.7	11
8	1013.5	507.3	996.5	995.5	E	1161.6	581.3	1144.6	1143.6	10
9	1112.6	556.8	1095.5	1094.6	V	1032.5	516.8	1015.5	1014.5	9
10	1227.6	614.3	1210.6	1209.6	D	933.5	467.2	916.5	915.5	8
11	1355.7	678.3	1338.6	1337.7	Q	818.4	409.7	801.4	800.4	7
12	1454.7	727.9	1437.7	1436.7	V	690.4	345.7	673.4	672.4	6
13	1583.8	792.4	1566.7	1565.8	E	591.3		574.3	573.3	5
14	1654.8	827.9	1637.8	1636.8	A	462.3		445.3		4
15	1785.9	893.4	1768.8	1767.8	M	391.2		374.2		3
16	1898.9	950.0	1881.9	1880.9	I	260.2		243.2		2
17	2045.0	1023.0	2028.0	2027.0	K	147.1		130.1		1

Gene symbol: Immt

Protein name: Isoform 1 of Mitochondrial inner membrane protein

Protein accession numbers: IPI00228150,IPI00381412,IPI00381413,IPI00554845

Peptide sequence: (R)GIEQAVQSHAVAEEEAR(K)

Exclusive (unique to this protein): TRUE

SEQUEST XCorr score: 3.42 SEQUEST DCn score: 0.601

Fix modifications: None

Variable modifications: None

charge: 2 Observed m/z: 912.4451

Actual minus calculated peptide mass (AMU): -0.0063456

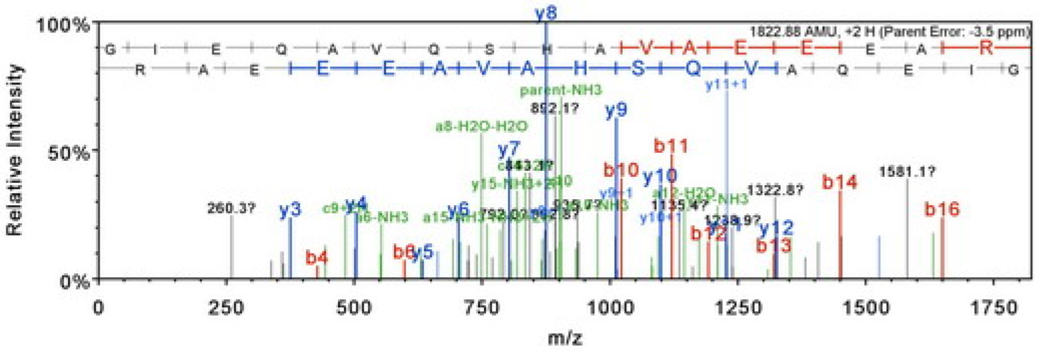

B	B Ions	B+2H	B-NH3	B-H2O	AA	Y Ions	Y+2H	Y-NH3	Y-H2O	Y
1	58.0				G	1823.9	912.5	1806.9	1805.9	17
2	171.1				I	1766.9	883.9	1749.8	1748.9	16
3	300.2			282.2	E	1653.8	827.4	1636.8	1635.8	15
4	428.2		411.2	410.2	Q	1524.7	762.9	1507.7	1506.7	14
5	499.3		482.2	481.2	A	1396.7	698.8	1379.7	1378.7	13
6	598.3	299.7	581.3	580.3	V	1325.6	663.3	1308.6	1307.6	12
7	726.4	363.7	709.4	708.4	Q	1226.6	613.8	1209.5	1208.6	11
8	813.4	407.2	796.4	795.4	S	1098.5	549.8	1081.5	1080.5	10
9	950.5	475.7	933.4	932.5	H	1011.5	506.3	994.5	993.5	9
10	1021.5	511.3	1004.5	1003.5	A	874.4	437.7	857.4	856.4	8
11	1120.6	560.8	1103.5	1102.6	V	803.4	402.2	786.4	785.4	7
12	1191.6	596.3	1174.6	1173.6	A	704.3	352.7	687.3	686.3	6
13	1320.7	660.8	1303.6	1302.6	E	633.3		616.3	615.3	5
14	1449.7	725.4	1432.7	1431.7	E	504.2		487.2	486.2	4
15	1578.7	789.9	1561.7	1560.7	E	375.2		358.2	357.2	3
16	1649.8	825.4	1632.8	1631.8	A	246.2		229.1		2
17	1823.9	912.5	1806.9	1805.9	R	175.1		158.1		1

Gene symbol: Ints6

Protein name: Isoform 1 of Integrator complex subunit 6

Protein accession numbers: IPI00461472,IPI00798463

Peptide sequence: (R)GDRYMLVTFEEPPYAIK(A)

Exclusive (unique to this protein): TRUE

SEQUEST XCorr score: 2.01 SEQUEST DCn score: 0.197

Fix modifications: None

Variable modifications: M5: Oxidation (+16.00)

charge: 2 Observed m/z: 1023.0156

Actual minus calculated peptide mass (AMU): 0.017658

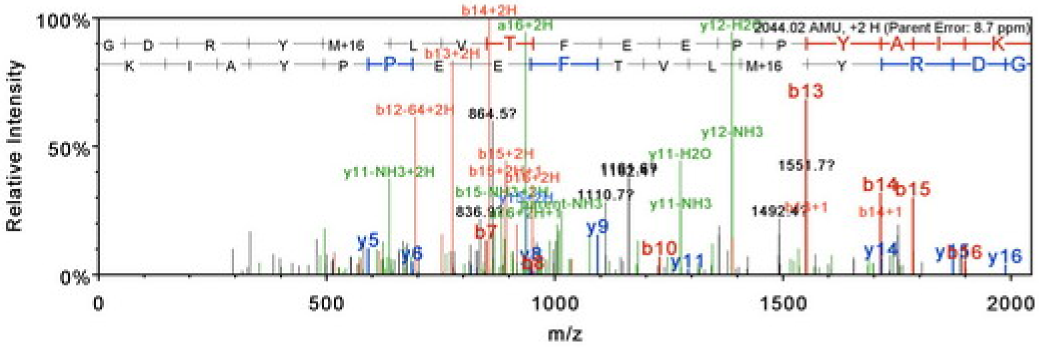

B	B Ions	B+2H	B-NH3	B-H2O	AA	Y Ions	Y+2H	Y-NH3	Y-H2O	Y
1	58.0				G	2045.0	1023.0	2028.0	2027.0	17
2	173.1			155.1	D	1988.0	994.5	1971.0	1970.0	16
3	329.2	165.1	312.1	311.2	R	1873.0	937.0	1855.9	1855.0	15
4	492.2	246.6	475.2	474.2	Y	1716.9	858.9	1699.8	1698.9	14
5	639.3	320.1	622.2	621.3	M+16	1553.8	777.4	1536.8	1535.8	13
6	752.3	376.7	735.3	734.3	L	1406.8	703.9	1389.7	1388.8	12
7	851.4	426.2	834.4	833.4	V	1293.7	647.3	1276.7	1275.7	11
8	952.5	476.7	935.4	934.5	T	1194.6	597.8	1177.6	1176.6	10
9	1099.5	550.3	1082.5	1081.5	F	1093.6	547.3	1076.5	1075.5	9
10	1228.6	614.8	1211.5	1210.6	E	946.5	473.8	929.5	928.5	8
11	1357.6	679.3	1340.6	1339.6	E	817.5	409.2	800.4	799.4	7
12	1454.7	727.8	1437.6	1436.7	P	688.4	344.7	671.4		6
13	1551.7	776.4	1534.7	1533.7	P	591.4		574.3		5
14	1714.8	857.9	1697.8	1696.8	Y	494.3		477.3		4
15	1785.8	893.4	1768.8	1767.8	A	331.2		314.2		3
16	1898.9	950.0	1881.9	1880.9	I	260.2		243.2		2
17	2045.0	1023.0	2028.0	2027.0	K	147.1		130.1		1

Gene symbol: Itgb4bp

Protein name: Eukaryotic translation initiation factor 6

Protein accession numbers: IPI00115862

Peptide sequence: (R)ASFENNCEVGCFAK(L)

Exclusive (unique to this protein): TRUE

SEQUEST XCorr score: 2.45 SEQUEST DCn score: 0.709

Fix modifications: C7: Carbamidomethyl (+57.02), C11: Carbamidomethyl (+57.02)Variable modifications: None

charge: 2 Observed m/z: 816.8444

Actual minus calculated peptide mass (AMU): 0.002319

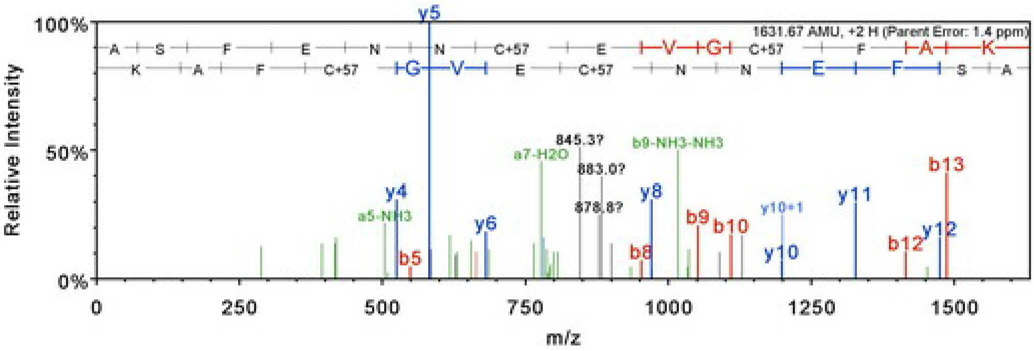

B	B Ions	B+2H	B-NH3	B-H2O	AA	Y Ions	Y+2H	Y-NH3	Y-H2O	Y
1	72.0				A	1632.7	816.8	1615.7	1614.7	14
2	159.1			141.1	S	1561.6	781.3	1544.6	1543.6	13
3	306.2			288.1	F	1474.6	737.8	1457.6	1456.6	12
4	435.2			417.2	E	1327.5	664.3	1310.5	1309.5	11
5	549.2		532.2	531.2	N	1198.5	599.8	1181.5	1180.5	10
6	663.3	332.1	646.3	645.3	N	1084.5	542.7	1067.4	1066.5	9
7	823.3	412.2	806.3	805.3	C+57	970.4	485.7	953.4	952.4	8
8	952.4	476.7	935.3	934.3	E	810.4	405.7	793.4	792.4	7
9	1051.4	526.2	1034.4	1033.4	V	681.3	341.2	664.3		6
10	1108.4	554.7	1091.4	1090.4	G	582.3		565.2		5
11	1268.5	634.7	1251.4	1250.5	C+57	525.3		508.2		4
12	1415.5	708.3	1398.5	1397.5	F	365.2		348.2		3
13	1486.6	743.8	1469.5	1468.6	A	218.2		201.1		2
14	1632.7	816.8	1615.7	1614.7	K	147.1		130.1		1

Gene symbol: Klk7

Protein name: Kallikrein-7 precursor

Protein accession numbers: IPI00126800

Peptide sequence: (K)THVNDIMLVRLDEPVK(M)

Exclusive (unique to this protein): TRUE

SEQUEST XCorr score: 2.16 SEQUEST DCn score: 0.52

Fix modifications: None

Variable modifications: M7: Oxidation (+16.00)

charge: 2 Observed m/z: 948.0143

Actual minus calculated peptide mass (AMU): 0.01453

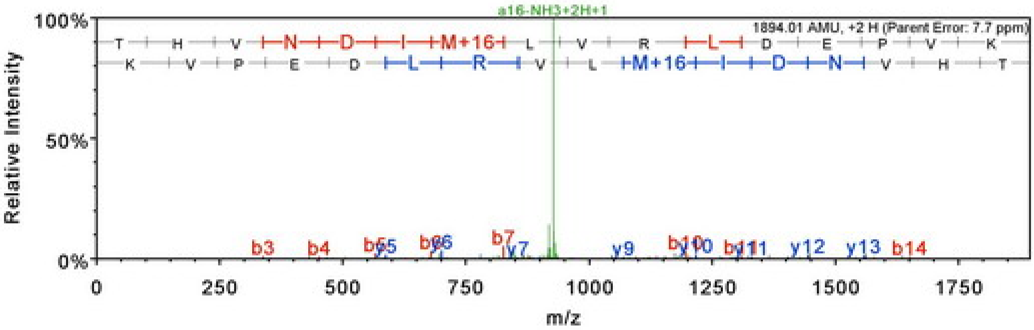

B	B Ions	B+2H	B-NH3	B-H2O	AA	Y Ions	Y+2H	Y-NH3	Y-H2O	Y
1	102.1			84.0	T	1895.0	948.0	1878.0	1877.0	16
2	239.1	120.1		221.1	H	1794.0	897.5	1776.9	1776.0	15
3	338.2	169.6		320.2	V	1656.9	829.0	1639.9	1638.9	14
4	452.2	226.6	435.2	434.2	N	1557.8	779.4	1540.8	1539.8	13
5	567.3	284.1	550.2	549.2	D	1443.8	722.4	1426.8	1425.8	12
6	680.3	340.7	663.3	662.3	I	1328.8	664.9	1311.7	1310.8	11
7	827.4	414.2	810.4	809.4	M+16	1215.7	608.3	1198.7	1197.7	10
8	940.5	470.7	923.4	922.5	L	1068.6	534.8	1051.6	1050.6	9
9	1039.5	520.3	1022.5	1021.5	V	955.6	478.3	938.5	937.6	8
10	1195.6	598.3	1178.6	1177.6	R	856.5	428.8	839.5	838.5	7
11	1308.7	654.9	1291.7	1290.7	L	700.4	350.7	683.4	682.4	6
12	1423.7	712.4	1406.7	1405.7	D	587.3		570.3	569.3	5
13	1552.8	776.9	1535.8	1534.8	E	472.3		455.3	454.3	4
14	1649.8	825.4	1632.8	1631.8	P	343.2		326.2		3
15	1748.9	875.0	1731.9	1730.9	V	246.2		229.2		2
16	1895.0	948.0	1878.0	1877.0	K	147.1		130.1		1

Gene symbol: Krt1

Protein name: Keratin, type II cytoskeletal 1

Protein accession numbers: IPI00625729,IPI00828242

Peptide sequence: (R)FLEQQNQVLQTK(W)

Exclusive (unique to this protein): TRUE

SEQUEST XCorr score: 2 SEQUEST DCn score: 0.382

Fix modifications: None

Variable modifications: None

charge: 2 Observed m/z: 738.3942

Actual minus calculated peptide mass (AMU): -0.005371

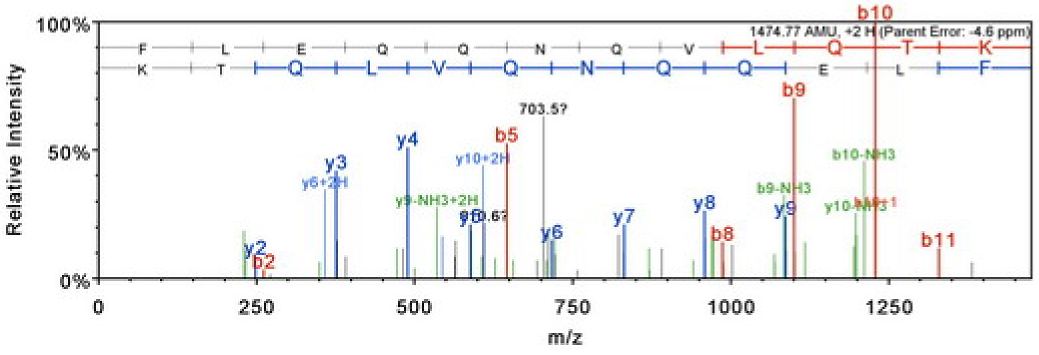

B	B Ions	B+2H	B-NH3	B-H2O	AA	Y Ions	Y+2H	Y-NH3	Y-H2O	Y
1	148.1				F	1475.8	738.4	1458.8	1457.8	12
2	261.2				L	1328.7	664.9	1311.7	1310.7	11
3	390.2			372.2	E	1215.6	608.3	1198.6	1197.6	10
4	518.3		501.2	500.3	Q	1086.6	543.8	1069.6	1068.6	9
5	646.3		629.3	628.3	Q	958.5	479.8	941.5	940.5	8
6	760.4	380.7	743.3	742.4	N	830.5	415.7	813.5	812.5	7
7	888.4	444.7	871.4	870.4	Q	716.4	358.7	699.4	698.4	6
8	987.5	494.3	970.5	969.5	V	588.4		571.4	570.4	5
9	1100.6	550.8	1083.5	1082.6	L	489.3		472.3	471.3	4
10	1228.6	614.8	1211.6	1210.6	Q	376.2		359.2	358.2	3
11	1329.7	665.3	1312.7	1311.7	T	248.2		231.1	230.2	2
12	1475.8	738.4	1458.8	1457.8	K	147.1		130.1		1

Gene symbol: Krt14

Protein name: Type I epidermal keratin (Fragment)

Protein accession numbers: IPI00828528

Peptide sequence: (R)LLEGEDAHLSSSQFSSGSQSSR(D)

Exclusive (unique to this protein): TRUE

SEQUEST XCorr score: 2.06 SEQUEST DCn score: 0.518

Fix modifications: None

Variable modifications: None

charge: 2 Observed m/z: 1155.0376

Actual minus calculated peptide mass (AMU): 0.00293

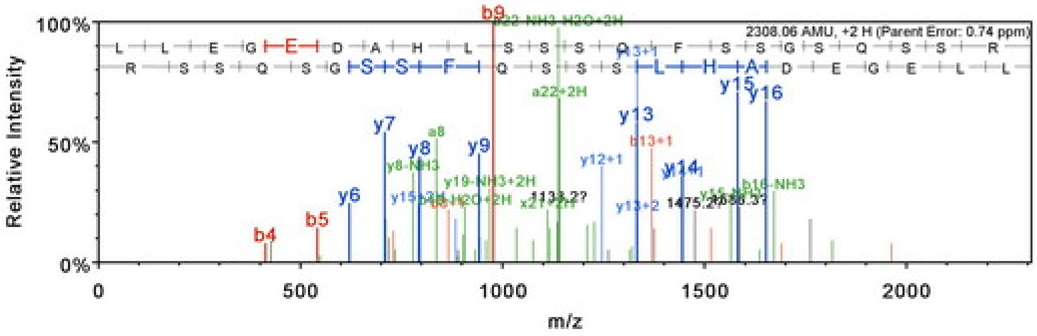

B	B Ions	B+2H	B-NH3	B-H2O	AA	Y Ions	Y+2H	Y-NH3	Y-H2O	Y
1	114.1				L	2309.1	1155.0	2292.0	2291.1	22
2	227.2				L	2196.0	1098.5	2178.9	2178.0	21
3	356.2			338.2	E	2082.9	1042.0	2065.9	2064.9	20
4	413.2			395.2	G	1953.9	977.4	1936.8	1935.8	19
5	542.3			524.3	E	1896.8	948.9	1879.8	1878.8	18
6	657.3	329.2		639.3	D	1767.8	884.4	1750.8	1749.8	17
7	728.4	364.7		710.3	A	1652.8	826.9	1635.7	1634.8	16
8	865.4	433.2		847.4	H	1581.7	791.4	1564.7	1563.7	15
9	978.5	489.8		960.5	L	1444.7	722.8	1427.6	1426.7	14
10	1065.5	533.3		1047.5	S	1331.6	666.3	1314.6	1313.6	13
11	1152.5	576.8		1134.5	S	1244.5	622.8	1227.5	1226.5	12
12	1239.6	620.3		1221.6	S	1157.5	579.3	1140.5	1139.5	11
13	1367.6	684.3	1350.6	1349.6	Q	1070.5	535.8	1053.5	1052.5	10
14	1514.7	757.9	1497.7	1496.7	F	942.4	471.7	925.4	924.4	9
15	1601.7	801.4	1584.7	1583.7	S	795.4	398.2	778.3	777.4	8
16	1688.8	844.9	1671.8	1670.8	S	708.3	354.7	691.3	690.3	7
17	1745.8	873.4	1728.8	1727.8	G	621.3	311.2	604.3	603.3	6
18	1832.8	916.9	1815.8	1814.8	S	564.3		547.3	546.3	5
19	1960.9	981.0	1943.9	1942.9	Q	477.2		460.2	459.2	4
20	2047.9	1024.5	2030.9	2029.9	S	349.2		332.2	331.2	3
21	2134.9	1068.0	2117.9	2116.9	S	262.1		245.1	244.1	2
22	2309.1	1155.0	2292.0	2291.1	R	175.1		158.1		1

Gene symbol: Krt78

Protein name: similar to Keratin, type I cytoskeletal 10

Protein accession numbers: IPI00750213,IPI00755181,IPI00798492,IPI00828744

Peptide sequence: (K)QSLEASLAETEGR(Y)

Exclusive (unique to this protein): TRUE

SEQUEST XCorr score: 2.44 SEQUEST DCn score: 0.721

Fix modifications: None

Variable modifications: None

charge: 2 Observed m/z: 695.8404

Actual minus calculated peptide mass (AMU): -0.008423

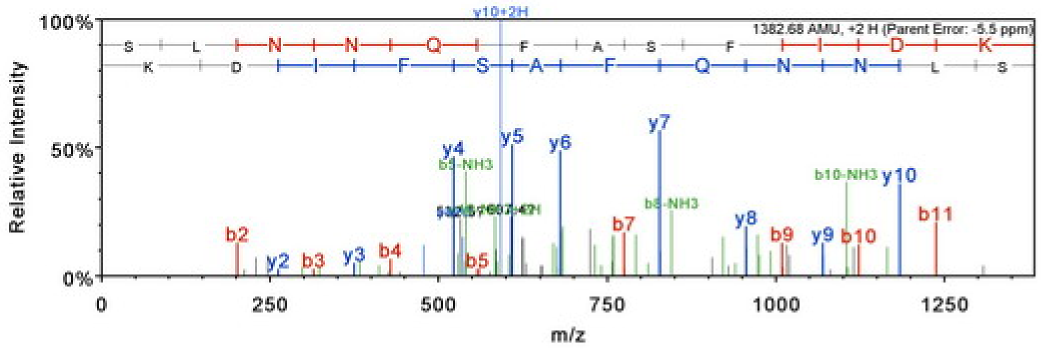

B	B Ions	B+2H	B-NH3	B-H2O	AA	Y Ions	Y+2H	Y-NH3	Y-H2O	Y
1	88.0			70.0	S	1383.7	692.4	1366.7	1365.7	12
2	201.1			183.1	L	1296.7	648.8	1279.6	1278.7	11
3	315.2		298.1	297.2	N	1183.6	592.3	1166.5	1165.6	10
4	429.2		412.2	411.2	N	1069.5	535.3	1052.5	1051.5	9
5	557.3		540.2	539.3	Q	955.5	478.3	938.5	937.5	8
6	704.3	352.7	687.3	686.3	F	827.4	414.2	810.4	809.4	7
7	775.4	388.2	758.4	757.4	A	680.4	340.7	663.3	662.4	6
8	862.4	431.7	845.4	844.4	S	609.3		592.3	591.3	5
9	1009.5	505.2	992.5	991.5	F	522.3		505.3	504.3	4
10	1122.6	561.8	1105.5	1104.5	I	375.2		358.2	357.2	3
11	1237.6	619.3	1220.6	1219.6	D	262.1		245.1	244.1	2
12	1383.7	692.4	1366.7	1365.7	K	147.1		130.1		1

Gene symbol: Krt79

Protein name: Keratin 79

Protein accession numbers: IPI00124499

Peptide sequence: (R)NLDLDSIIAEVK(A)

Exclusive (unique to this protein): TRUE

SEQUEST XCorr score: 2.01 SEQUEST DCn score: 0.561

Fix modifications: None

Variable modifications: None

charge: 2 Observed m/z: 665.3663

Actual minus calculated peptide mass (AMU): -0.0019551

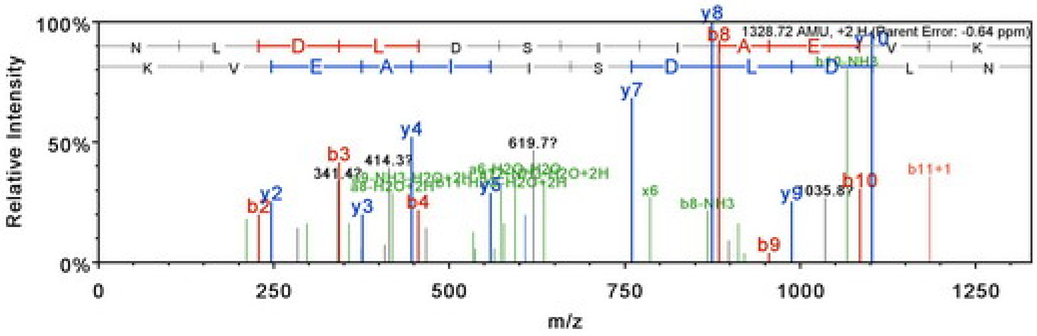

B	B Ions	B+2H	B-NH3	B-H2O	AA	Y Ions	Y+2H	Y-NH3	Y-H2O	Y
1	115.1		98.0		N	1329.7	665.4	1312.7	1311.7	12
2	228.1		211.1		L	1215.7	608.4	1198.7	1197.7	11
3	343.2		326.1	325.2	D	1102.6	551.8	1085.6	1084.6	10
4	456.3		439.2	438.2	L	987.6	494.3	970.6	969.6	9
5	571.3		554.3	553.3	D	874.5	437.8	857.5	856.5	8
6	658.3	329.7	641.3	640.3	S	759.5	380.2	742.4	741.5	7
7	771.4	386.2	754.4	753.4	I	672.4	336.7	655.4	654.4	6
8	884.5	442.7	867.5	866.5	I	559.4		542.3	541.3	5
9	955.5	478.3	938.5	937.5	A	446.3		429.2	428.3	4
10	1084.5	542.8	1067.5	1066.5	E	375.2		358.2	357.2	3
11	1183.6	592.3	1166.6	1165.6	V	246.2		229.2		2
12	1329.7	665.4	1312.7	1311.7	K	147.1		130.1		1

Gene symbol: Lamb1-1

Protein name: laminin B1 subunit 1

Protein accession numbers: IPI00338785

Peptide sequence: (R)YSDIEPSTEGEVIFR(A)

Exclusive (unique to this protein): TRUE

SEQUEST XCorr score: 2.1 SEQUEST DCn score: 0.383

Fix modifications: None

Variable modifications: None

charge: 2 Observed m/z: 871.4152

Actual minus calculated peptide mass (AMU): -0.005859

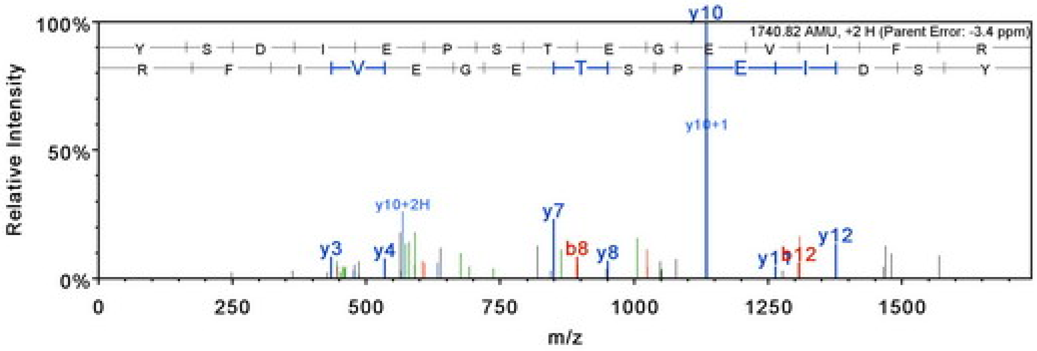

B	B Ions	B+2H	B-NH3	B-H2O	AA	Y Ions	Y+2H	Y-NH3	Y-H2O	Y
1	164.1				Y	1741.8	871.4	1724.8	1723.8	15
2	251.1			233.1	S	1578.8	789.9	1561.7	1560.8	14
3	366.1			348.1	D	1491.7	746.4	1474.7	1473.7	13
4	479.2			461.2	I	1376.7	688.9	1359.7	1358.7	12
5	608.3			590.3	E	1263.6	632.3	1246.6	1245.6	11
6	705.3	353.2		687.3	P	1134.6	567.8	1117.5	1116.6	10
7	792.3	396.7		774.3	S	1037.5	519.3	1020.5	1019.5	9
8	893.4	447.2		875.4	T	950.5	475.8	933.5	932.5	8
9	1022.4	511.7		1004.4	E	849.5	425.2	832.4	831.4	7
10	1079.5	540.2		1061.4	G	720.4	360.7	703.4	702.4	6
11	1208.5	604.8		1190.5	E	663.4		646.4	645.4	5
12	1307.6	654.3		1289.5	V	534.3		517.3		4
13	1420.7	710.8		1402.6	I	435.3		418.3		3
14	1567.7	784.4		1549.7	F	322.2		305.2		2
15	1741.8	871.4	1724.8	1723.8	R	175.1		158.1		1

Gene symbol: Lcp1

Protein name: Plastin-2

Protein accession numbers: IPI00118892

Peptide sequence: (K)FSLVGIAGQDLNEGNR(T)

Exclusive (unique to this protein): TRUE

SEQUEST XCorr score: 2.21 SEQUEST DCn score: 0.603

Fix modifications: None

Variable modifications: None

charge: 2 Observed m/z: 845.4285

Actual minus calculated peptide mass (AMU): -0.006958

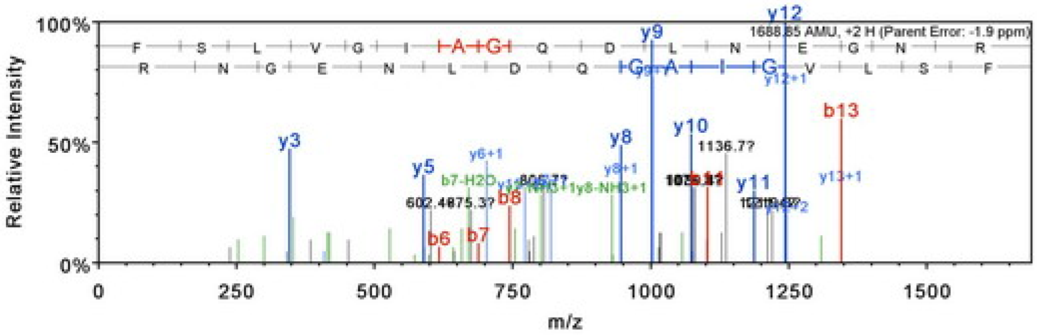

B	B Ions	B+2H	B-NH3	B-H2O	AA	Y Ions	Y+2H	Y-NH3	Y-H2O	Y
1	148.1				F	1689.9	845.4	1672.8	1671.9	16
2	235.1			217.1	S	1542.8	771.9	1525.8	1524.8	15
3	348.2			330.2	L	1455.8	728.4	1438.7	1437.8	14
4	447.3			429.3	V	1342.7	671.8	1325.7	1324.7	13
5	504.3			486.3	G	1243.6	622.3	1226.6	1225.6	12
6	617.4	309.2		599.4	I	1186.6	593.8	1169.6	1168.6	11
7	688.4	344.7		670.4	A	1073.5	537.3	1056.5	1055.5	10
8	745.4	373.2		727.4	G	1002.5	501.7	985.4	984.5	9
9	873.5	437.3	856.5	855.5	Q	945.4	473.2	928.4	927.4	8
10	988.5	494.8	971.5	970.5	D	817.4	409.2	800.4	799.4	7
11	1101.6	551.3	1084.6	1083.6	L	702.4	351.7	685.3	684.3	6
12	1215.6	608.3	1198.6	1197.6	N	589.3		572.2	571.3	5
13	1344.7	672.8	1327.7	1326.7	E	475.2		458.2	457.2	4
14	1401.7	701.4	1384.7	1383.7	G	346.2		329.2		3
15	1515.7	758.4	1498.7	1497.7	N	289.2		272.1		2
16	1689.9	845.4	1672.8	1671.9	R	175.1		158.1		1

Gene symbol: Lman2

Protein name: 9 days embryo whole body cDNA, RIKEN full-length enriched library, clone:D030015M14 product:VESICULAR INTEGRAL-MEMBRANE PROTEIN VIP36 (VIP36) homolog

Protein accession numbers: IPI00308609,IPI00321634

Peptide sequence: (R)WSELAGCTADFR(N)

Exclusive (unique to this protein): TRUE

SEQUEST XCorr score: 3.08 SEQUEST DCn score: 0.718

Fix modifications: C7: Carbamidomethyl (+57.02)Variable modifications: None

charge: 2 Observed m/z: 706.8142

Actual minus calculated peptide mass (AMU): -0.0063456

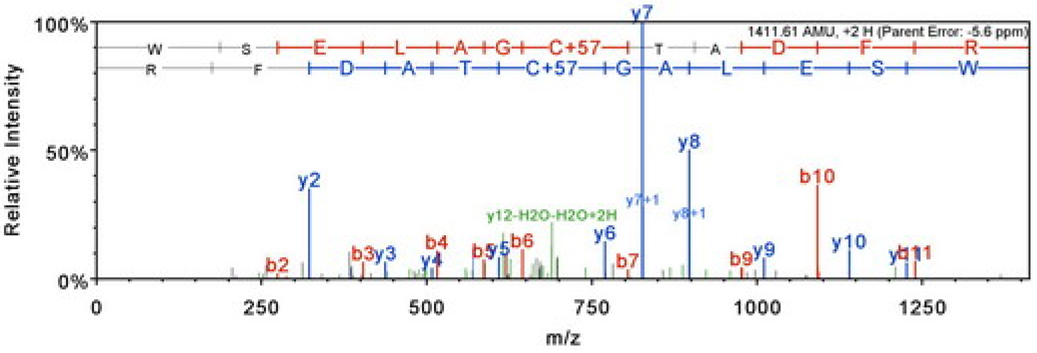

B	B Ions	B+2H	B-NH3	B-H2O	AA	Y Ions	Y+2H	Y-NH3	Y-H2O	Y
1	187.1				W	1412.6	706.8	1395.6	1394.6	12
2	274.1			256.1	S	1226.5	613.8	1209.5	1208.5	11
3	403.2			385.2	E	1139.5	570.3	1122.5	1121.5	10
4	516.3			498.2	L	1010.5	505.7	993.5	992.5	9
5	587.3			569.3	A	897.4	449.2	880.4	879.4	8
6	644.3	322.7		626.3	G	826.4	413.7	809.3	808.3	7
7	804.3	402.7		786.3	C+57	769.3	385.2	752.3	751.3	6
8	905.4	453.2		887.4	T	609.3		592.3	591.3	5
9	976.4	488.7		958.4	A	508.3		491.2	490.2	4
10	1091.5	546.2		1073.4	D	437.2		420.2	419.2	3
11	1238.5	619.8		1220.5	F	322.2		305.2		2
12	1412.6	706.8	1395.6	1394.6	R	175.1		158.1		1

Gene symbol: LOC382492

Protein name: similar to 40S ribosomal protein S17

Protein accession numbers: IPI00381546,IPI00465880,IPI00755495,IPI00756135,IPI00848900

Peptide sequence: (R)DNYVPEVSALDQEIIEVDPDTK(E)

Exclusive (unique to this protein): TRUE

SEQUEST XCorr score: 2.23 SEQUEST DCn score: 0.4

Fix modifications: None

Variable modifications: None

charge: 2 Observed m/z: 1245.0983

Actual minus calculated peptide mass (AMU): -0.004883

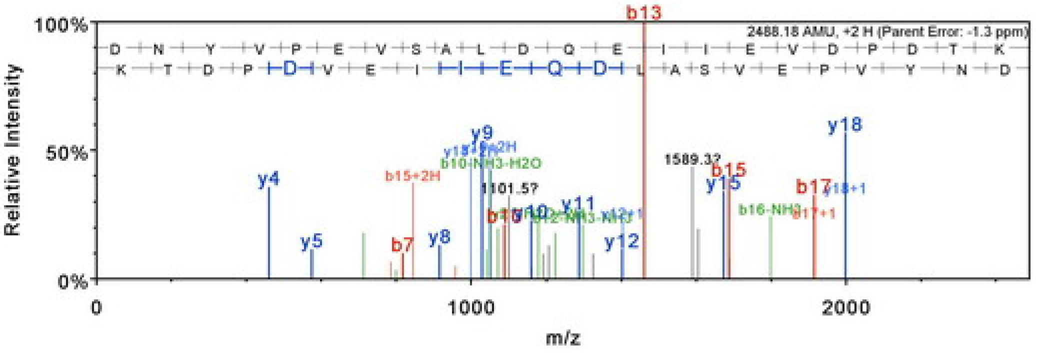

B	B Ions	B+2H	B-NH3	B-H2O	AA	Y Ions	Y+2H	Y-NH3	Y-H2O	Y
1	116.0			98.0	D	2489.2	1245.1	2472.2	2471.2	22
2	230.1		213.1	212.1	N	2374.2	1187.6	2357.1	2356.2	21
3	393.1		376.1	375.1	Y	2260.1	1130.6	2243.1	2242.1	20
4	492.2		475.2	474.2	V	2097.1	1049.0	2080.0	2079.1	19
5	589.3		572.2	571.3	P	1998.0	999.5	1981.0	1980.0	18
6	718.3	359.7	701.3	700.3	E	1900.9	951.0	1883.9	1882.9	17
7	817.4	409.2	800.4	799.4	V	1771.9	886.5	1754.9	1753.9	16
8	904.4	452.7	887.4	886.4	S	1672.8	836.9	1655.8	1654.8	15
9	975.4	488.2	958.4	957.4	A	1585.8	793.4	1568.8	1567.8	14
10	1088.5	544.8	1071.5	1070.5	L	1514.8	757.9	1497.7	1496.8	13
11	1203.5	602.3	1186.5	1185.5	D	1401.7	701.3	1384.7	1383.7	12
12	1331.6	666.3	1314.6	1313.6	Q	1286.7	643.8	1269.6	1268.6	11
13	1460.7	730.8	1443.6	1442.6	E	1158.6	579.8	1141.6	1140.6	10
14	1573.7	787.4	1556.7	1555.7	I	1029.5	515.3	1012.5	1011.5	9
15	1686.8	843.9	1669.8	1668.8	I	916.5	458.7	899.4	898.5	8
16	1815.9	908.4	1798.8	1797.9	E	803.4	402.2	786.4	785.4	7
17	1914.9	958.0	1897.9	1896.9	V	674.3	337.7	657.3	656.3	6
18	2030.0	1015.5	2012.9	2012.0	D	575.3		558.2	557.3	5
19	2127.0	1064.0	2110.0	2109.0	P	460.2		443.2	442.2	4
20	2242.0	1121.5	2225.0	2224.0	D	363.2		346.2	345.2	3
21	2343.1	1172.0	2326.1	2325.1	T	248.2		231.1	230.2	2
22	2489.2	1245.1	2472.2	2471.2	K	147.1		130.1		1

Gene symbol: LOC627816

Protein name: similar to protein phosphatase 1, catalytic subunit, gamma isoform isoform 9

Protein accession numbers: IPI00752131

Peptide sequence: (K)TFTDCFNCLPIAAIVDEK(I)

Exclusive (unique to this protein): TRUE

SEQUEST XCorr score: 2.21 SEQUEST DCn score: 0.555

Fix modifications: C5: Carbamidomethyl (+57.02), C8: Carbamidomethyl (+57.02)Variable modifications: None

charge: 2 Observed m/z: 1057.4959

Actual minus calculated peptide mass (AMU): -0.010253

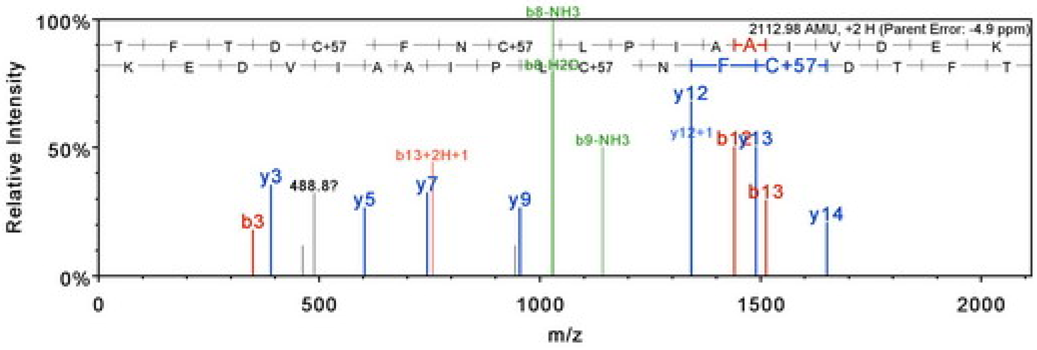

B	B Ions	B+2H	B-NH3	B-H2O	AA	Y Ions	Y+2H	Y-NH3	Y-H2O	Y
1	102.1			84.0	T	2114.0	1057.5	2097.0	2096.0	18
2	249.1			231.1	F	2013.0	1007.0	1995.9	1994.9	17
3	350.2			332.2	T	1865.9	933.4	1848.9	1847.9	16
4	465.2			447.2	D	1764.8	882.9	1747.8	1746.8	15
5	625.2			607.2	C+57	1649.8	825.4	1632.8	1631.8	14
6	772.3	386.7		754.3	F	1489.8	745.4	1472.8	1471.8	13
7	886.3	443.7	869.3	868.3	N	1342.7	671.9	1325.7	1324.7	12
8	1046.4	523.7	1029.3	1028.4	C+57	1228.7	614.8	1211.6	1210.7	11
9	1159.5	580.2	1142.4	1141.4	L	1068.6	534.8	1051.6	1050.6	10
10	1256.5	628.8	1239.5	1238.5	P	955.6	478.3	938.5	937.5	9
11	1369.6	685.3	1352.6	1351.6	I	858.5	429.8	841.5	840.5	8
12	1440.6	720.8	1423.6	1422.6	A	745.4	373.2	728.4	727.4	7
13	1511.7	756.3	1494.6	1493.7	A	674.4	337.7	657.4	656.4	6
14	1624.8	812.9	1607.7	1606.7	I	603.3		586.3	585.3	5
15	1723.8	862.4	1706.8	1705.8	V	490.3		473.2	472.2	4
16	1838.9	919.9	1821.8	1820.8	D	391.2		374.2	373.2	3
17	1967.9	984.5	1950.9	1949.9	E	276.2		259.1	258.1	2
18	2114.0	1057.5	2097.0	2096.0	K	147.1		130.1		1

Gene symbol: LOC630393

Protein name: similar to PRAME family member DJ1198H6.2 isoform 6

Protein accession numbers: IPI00623662,IPI00667531,IPI00848712

Peptide sequence: (R)LDESTTYLLQWAQQR(K)

Exclusive (unique to this protein): TRUE

SEQUEST XCorr score: 2.23 SEQUEST DCn score: 0.478

Fix modifications: None

Variable modifications: None

charge: 2 Observed m/z: 926.4629

Actual minus calculated peptide mass (AMU): -0.0063456

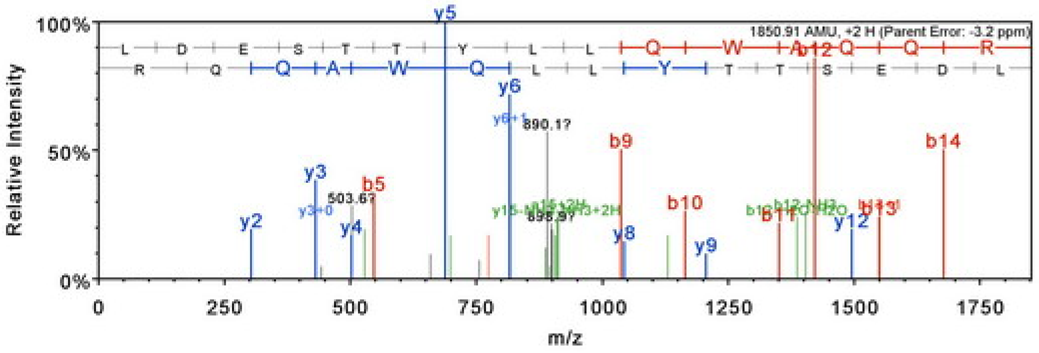

B	B Ions	B+2H	B-NH3	B-H2O	AA	Y Ions	Y+2H	Y-NH3	Y-H2O	Y
1	114.1				L	1851.9	926.5	1834.9	1833.9	15
2	229.1			211.1	D	1738.8	869.9	1721.8	1720.8	14
3	358.2			340.2	E	1623.8	812.4	1606.8	1605.8	13
4	445.2			427.2	S	1494.8	747.9	1477.7	1476.8	12
5	546.2			528.2	T	1407.7	704.4	1390.7	1389.7	11
6	647.3	324.2		629.3	T	1306.7	653.9	1289.7	1288.7	10
7	810.4	405.7		792.3	Y	1205.6	603.3	1188.6		9
8	923.4	462.2		905.4	L	1042.6	521.8	1025.5		8
9	1036.5	518.8		1018.5	L	929.5	465.3	912.5		7
10	1164.6	582.8	1147.5	1146.6	Q	816.4	408.7	799.4		6
11	1350.7	675.8	1333.6	1332.7	W	688.4		671.3		5
12	1421.7	711.4	1404.7	1403.7	A	502.3		485.3		4
13	1549.8	775.4	1532.7	1531.7	Q	431.2		414.2		3
14	1677.8	839.4	1660.8	1659.8	Q	303.2		286.2		2
15	1851.9	926.5	1834.9	1833.9	R	175.1		158.1		1

Gene symbol: LOC668706;LOC672854

Protein name: similar to 60S ribosomal protein L12

Protein accession numbers: IPI00338838

Peptide sequence: (R)QAQIEVVPSASALIIK(A)

Exclusive (unique to this protein): TRUE

SEQUEST XCorr score: 2.07   SEQUEST DCn score: 0.634

Fix modifications: None

Variable modifications: None

charge: 2   Observed m/z: 833.9921

Actual minus calculated peptide mass (AMU): 0.001831

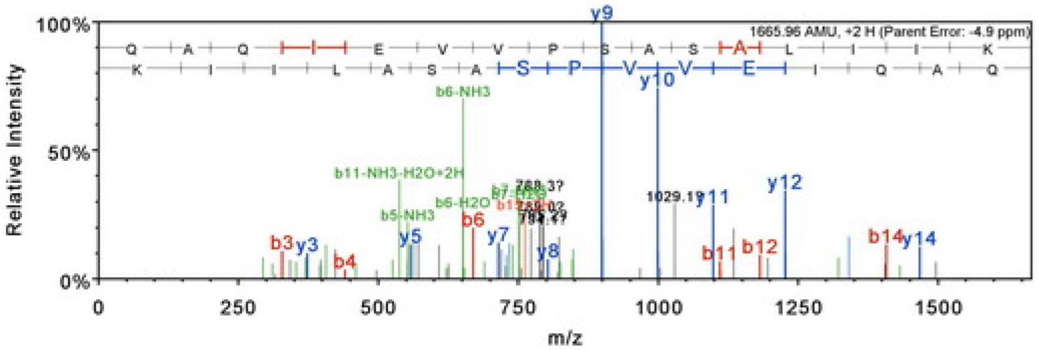

B	B Ions	B+2H	B-NH3	B-H2O	AA	Y Ions	Y+2H	Y-NH3	Y-H2O	Y
1	129.1		112.0		Q	1667.0	834.0	1650.0	1649.0	16
2	200.1		183.1		A	1538.9	770.0	1521.9	1520.9	15
3	328.2		311.1		Q	1467.9	734.4	1450.9	1449.9	14
4	441.3		424.2		I	1339.8	670.4	1322.8	1321.8	13
5	570.3		553.3	552.3	E	1226.7	613.9	1209.7	1208.7	12
6	669.4	335.2	652.3	651.4	V	1097.7	549.4	1080.7	1079.7	11
7	768.4	384.7	751.4	750.4	V	998.6	499.8	981.6	980.6	10
8	865.5	433.2	848.5	847.5	P	899.6	450.3	882.5	881.6	9
9	952.5	476.8	935.5	934.5	S	802.5	401.8	785.5	784.5	8
10	1023.6	512.3	1006.5	1005.5	A	715.5	358.2	698.5	697.5	7
11	1110.6	555.8	1093.5	1092.6	S	644.4	322.7	627.4	626.4	6
12	1181.6	591.3	1164.6	1163.6	A	557.4		540.4		5
13	1294.7	647.9	1277.7	1276.7	L	486.4		469.3		4
14	1407.8	704.4	1390.8	1389.8	I	373.3		356.3		3
15	1520.9	760.9	1503.8	1502.9	I	260.2		243.2		2
16	1667.0	834.0	1650.0	1649.0	K	147.1		130.1		1

Gene symbol: LOC671726;EG666974

Protein name: similar to Proteasome subunit alpha type 5 (Proteasome zeta chain) (Macropain zeta chain) (Multicatalytic endopeptidase complex zeta chain) isoform 1

Protein accession numbers: IPI00122562,IPI00131407,IPI00757768

Peptide sequence: (R)AIGSASEGAQSSLQEVYHK(S)

Exclusive (unique to this protein): TRUE

SEQUEST XCorr score: 2.02   SEQUEST DCn score: 0.615

Fix modifications: None

Variable modifications: None

charge: 2   Observed m/z: 981.4853

Actual minus calculated peptide mass (AMU): 0.006226

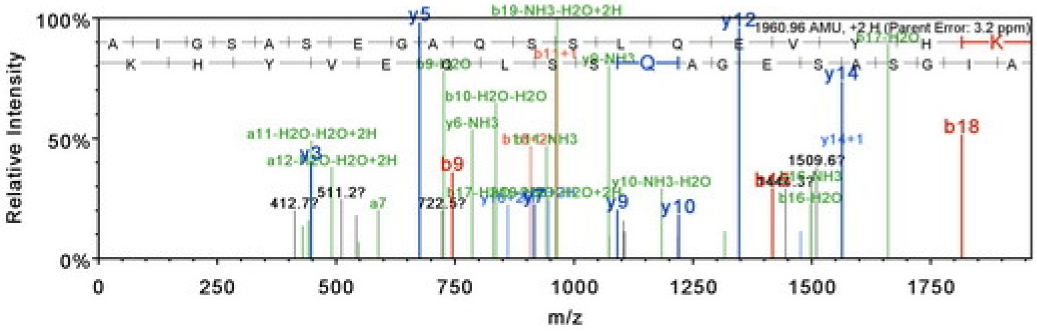

B	B Ions	B+2H	B-NH3	B-H2O	AA	Y Ions	Y+2H	Y-NH3	Y-H2O	Y
1	72.0				A	1962.0	981.5	1944.9	1944.0	19
2	185.1				I	1890.9	946.0	1873.9	1872.9	18
3	242.2				G	1777.8	889.4	1760.8	1759.8	17
4	329.2			311.2	S	1720.8	860.9	1703.8	1702.8	16
5	400.2			382.2	A	1633.8	817.4	1616.8	1615.8	15
6	487.3	244.1		469.2	S	1562.8	781.9	1545.7	1544.7	14
7	616.3	308.7		598.3	E	1475.7	738.4	1458.7	1457.7	13
8	673.3	337.2		655.3	G	1346.7	673.8	1329.6	1328.7	12
9	744.4	372.7		726.3	A	1289.7	645.3	1272.6	1271.6	11
10	872.4	436.7	855.4	854.4	Q	1218.6	609.8	1201.6	1200.6	10
11	959.4	480.2	942.4	941.4	S	1090.5	545.8	1073.5	1072.5	9
12	1046.5	523.7	1029.5	1028.5	S	1003.5	502.3	986.5	985.5	8
13	1159.6	580.3	1142.5	1141.5	L	916.5	458.8	899.5	898.5	7
14	1287.6	644.3	1270.6	1269.6	Q	803.4	402.2	786.4	785.4	6
15	1416.7	708.8	1399.6	1398.7	E	675.4	338.2	658.3	657.3	5
16	1515.7	758.4	1498.7	1497.7	V	546.3	273.7	529.3		4
17	1678.8	839.9	1661.8	1660.8	Y	447.2	224.1	430.2		3
18	1815.9	908.4	1798.8	1797.8	H	284.2	142.6	267.1		2
19	1962.0	981.5	1944.9	1944.0	K	147.1		130.1		1

Gene symbol: LOC671953

Protein name: similar to Myosin light polypeptide 6

Protein accession numbers: IPI00264053,IPI00354819,IPI00409817,IPI00474757,IPI00753099,IPI00848866

Peptide sequence: (K)DQGTYEDYVEGLR(V)

Exclusive (unique to this protein): TRUE

SEQUEST XCorr score: 2.38 SEQUEST DCn score: 0.545

Fix modifications: None

Variable modifications: None

charge: 2 Observed m/z: 772.8438

Actual minus calculated peptide mass (AMU): -0.007324

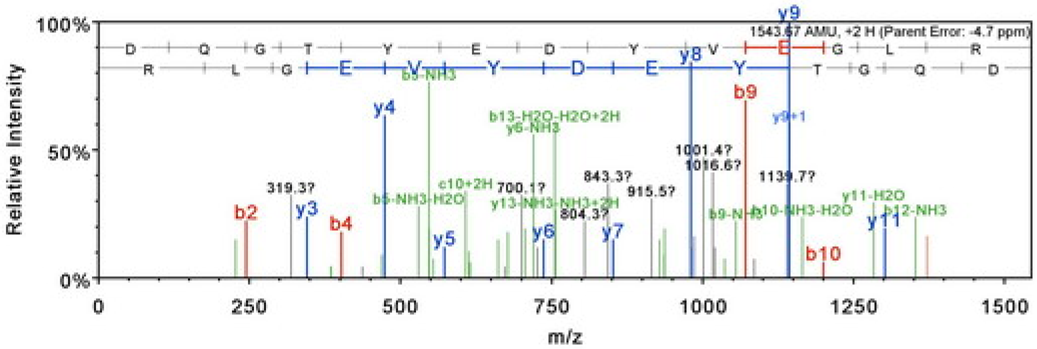

B	B Ions	B+2H	B-NH3	B-H2O	AA	Y Ions	Y+2H	Y-NH3	Y-H2O	Y
1	116.0			98.0	D	1544.7	772.9	1527.7	1526.7	13
2	244.1		227.1	226.1	Q	1429.7	715.3	1412.6	1411.7	12
3	301.1		284.1	283.1	G	1301.6	651.3	1284.6	1283.6	11
4	402.2		385.1	384.2	T	1244.6	622.8	1227.5	1226.6	10
5	565.2		548.2	547.2	Y	1143.5	572.3	1126.5	1125.5	9
6	694.3	347.6	677.2	676.3	E	980.5	490.7	963.4	962.5	8
7	809.3	405.2	792.3	791.3	D	851.4	426.2	834.4	833.4	7
8	972.4	486.7	955.3	954.4	Y	736.4	368.7	719.4	718.4	6
9	1071.4	536.2	1054.4	1053.4	V	573.3		556.3	555.3	5
10	1200.5	600.7	1183.4	1182.5	E	474.3		457.2	456.3	4
11	1257.5	629.3	1240.5	1239.5	G	345.2		328.2		3
12	1370.6	685.8	1353.5	1352.6	L	288.2		271.2		2
13	1544.7	772.9	1527.7	1526.7	R	175.1		158.1		1

Gene symbol: LOC673608

Protein name: similar to 60S ribosomal protein L27a

Protein accession numbers: IPI00754044

Peptide sequence: (K)TGVAPIIDVVR(S)

Exclusive (unique to this protein): TRUE

SEQUEST XCorr score: 2.14 SEQUEST DCn score: 0.465

Fix modifications: None

Variable modifications: None

charge: 2 Observed m/z: 570.3395

Actual minus calculated peptide mass (AMU): -0.007935

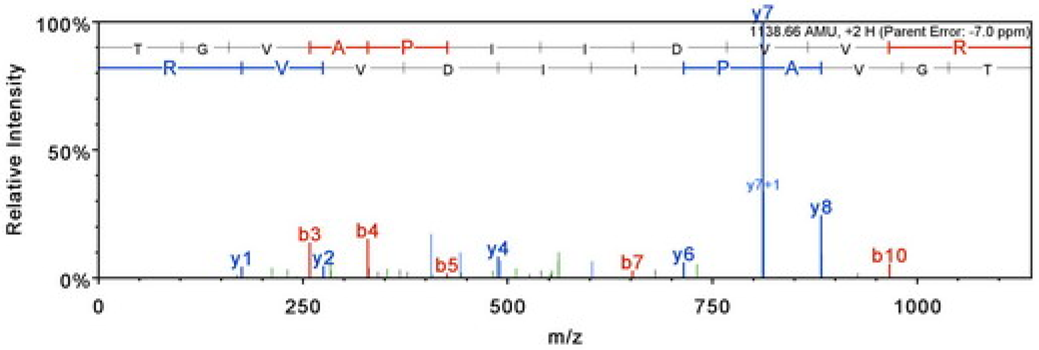

B	B Ions	B+2H	B-NH3	B-H2O	AA	Y Ions	Y+2H	Y-NH3	Y-H2O	Y
1	102.1			84.0	T	1139.7	570.3	1122.7	1121.7	11
2	159.1			141.1	G	1038.6	519.8	1021.6	1020.6	10
3	258.1			240.1	V	981.6	491.3	964.6	963.6	9
4	329.2			311.2	A	882.5	441.8	865.5	864.5	8
5	426.2			408.2	P	811.5	406.3	794.5	793.5	7
6	539.3	270.2		521.3	I	714.5	357.7	697.4	696.4	6
7	652.4	326.7		634.4	I	601.4		584.3	583.4	5
8	767.4	384.2		749.4	D	488.3		471.3	470.3	4
9	866.5	433.8		848.5	V	373.3		356.2		3
10	965.6	483.3		947.6	V	274.2		257.2		2
11	1139.7	570.3	1122.7	1121.7	R	175.1		158.1		1

Gene symbol: LOC675088;EG621100;Rpl27;LOC669376
Protein name: 60S ribosomal protein L27
Protein accession numbers: IPI00122421,IPI00626531,IPI00674087
Peptide sequence: (K)NIDDGTSDRPYSHALVAGIDR(Y)
Exclusive (unique to this protein): TRUE
SEQUEST XCorr score: 3.32 SEQUEST DCn score: 0.614
Fix modifications: None
Variable modifications: None
charge: 3 Observed m/z: 758.039
Actual minus calculated peptide mass (AMU): 0.005371

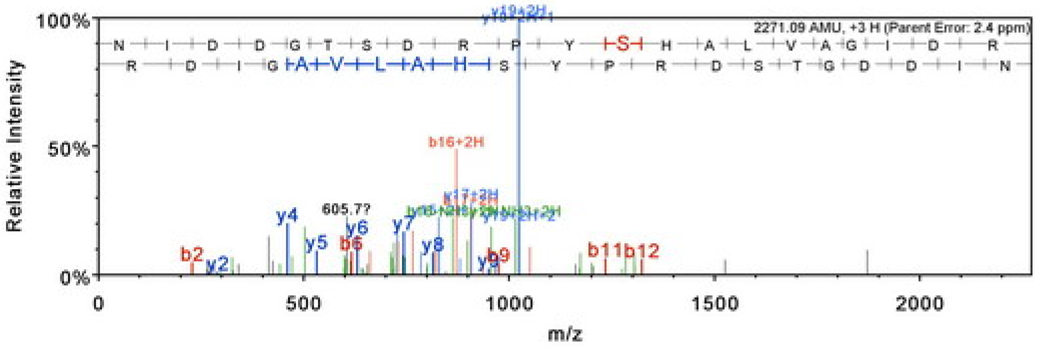

B	B Ions	B+2H	B-NH3	B-H2O	AA	Y Ions	Y+2H	Y-NH3	Y-H2O	Y
1	115.1		98.0		N	2272.1	1136.5	2255.1	2254.1	21
2	228.1		211.1		I	2158.1	1079.5	2141.0	2140.0	20
3	343.2		326.1	325.2	D	2045.0	1023.0	2027.9	2027.0	19
4	458.2		441.2	440.2	D	1929.9	965.5	1912.9	1911.9	18
5	515.2		498.2	497.2	G	1814.9	908.0	1797.9	1796.9	17
6	616.3	308.6	599.2	598.3	T	1757.9	879.5	1740.9	1739.9	16
7	703.3	352.2	686.3	685.3	S	1656.9	828.9	1639.8	1638.8	15
8	818.3	409.7	801.3	800.3	D	1569.8	785.4	1552.8	1551.8	14
9	974.4	487.7	957.4	956.4	R	1454.8	727.9	1437.8	1436.8	13
10	1071.5	536.2	1054.4	1053.5	P	1298.7	649.9	1281.7	1280.7	12
11	1234.5	617.8	1217.5	1216.5	Y	1201.6	601.3	1184.6	1183.6	11
12	1321.6	661.3	1304.5	1303.6	S	1038.6	519.8	1021.5	1020.6	10
13	1458.6	729.8	1441.6	1440.6	H	951.5	476.3	934.5	933.5	9
14	1529.7	765.3	1512.6	1511.7	A	814.5	407.7	797.5	796.5	8
15	1642.8	821.9	1625.7	1624.7	L	743.4	372.2	726.4	725.4	7
16	1741.8	871.4	1724.8	1723.8	V	630.4	315.7	613.3	612.4	6
17	1812.9	906.9	1795.8	1794.8	A	531.3		514.3	513.3	5
18	1869.9	935.4	1852.9	1851.9	G	460.3		443.2	442.2	4
19	1983.0	992.0	1965.9	1965.0	I	403.2		386.2	385.2	3
20	2098.0	1049.5	2081.0	2080.0	D	290.2		273.1	272.1	2
21	2272.1	1136.5	2255.1	2254.1	R	175.1		158.1		1

Gene symbol: LOC675151;Ogdhl
Protein name: oxoglutarate dehydrogenase-like

Protein accession numbers: IPI00342603,IPI00749952,IPI00755352,IPI00756987
Peptide sequence: (R)LPTTTFIGGPENTLSLR(E)
Exclusive (unique to this protein): TRUE
SEQUEST XCorr score: 2.11 SEQUEST DCn score: 0.591
Fix modifications: None
Variable modifications: None
charge: 2 Observed m/z: 908.9913
Actual minus calculated peptide mass (AMU): -0.0063456

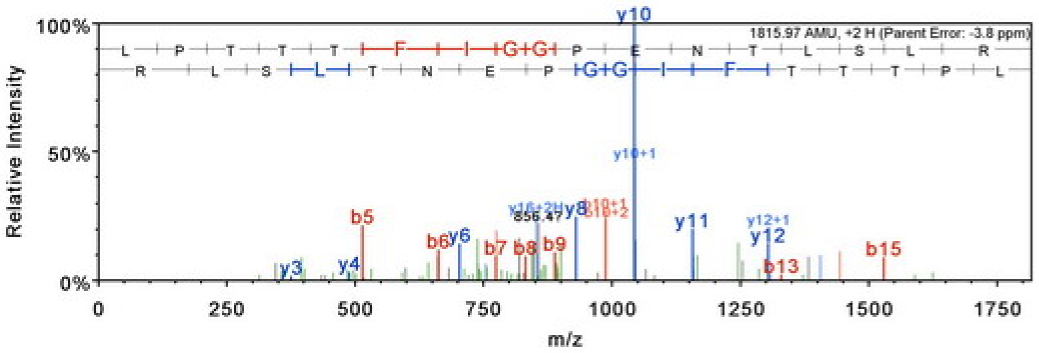

B	B Ions	B+2H	B-NH3	B-H2O	AA	Y Ions	Y+2H	Y-NH3	Y-H2O	Y
1	114.1				L	1817.0	909.0	1800.0	1799.0	17
2	211.1				P	1703.9	852.5	1686.9	1685.9	16
3	312.2			294.2	T	1606.8	803.9	1589.8	1588.8	15
4	413.2			395.2	T	1505.8	753.4	1488.8	1487.8	14
5	514.3			496.3	T	1404.8	702.9	1387.7	1386.7	13
6	661.4	331.2		643.4	F	1303.7	652.4	1286.7	1285.7	12
7	774.4	387.7		756.4	I	1156.6	578.8	1139.6	1138.6	11
8	831.5	416.2		813.5	G	1043.5	522.3	1026.5	1025.5	10
9	888.5	444.8		870.5	G	986.5	493.8	969.5	968.5	9
10	985.5	493.3		967.5	P	929.5	465.3	912.5	911.5	8
11	1114.6	557.8		1096.6	E	832.5	416.7	815.4	814.4	7
12	1228.6	614.8	1211.6	1210.6	N	703.4	352.2	686.4	685.4	6
13	1329.7	665.3	1312.6	1311.7	T	589.4		572.3	571.4	5
14	1442.8	721.9	1425.7	1424.7	L	488.3		471.3	470.3	4
15	1529.8	765.4	1512.8	1511.8	S	375.2		358.2	357.2	3
16	1642.9	821.9	1625.8	1624.9	L	288.2		271.2		2
17	1817.0	909.0	1800.0	1799.0	R	175.1		158.1		1

Gene symbol: LOC675789;Rpl24;EG668829
Protein name: similar to ribosomal protein L24
Protein accession numbers: IPI00134202,IPI00323806,IPI00762051
Peptide sequence: (R)AITGASLADIMAK(R)
Exclusive (unique to this protein): TRUE
SEQUEST XCorr score: 2.04 SEQUEST DCn score: 0.625
Fix modifications: None
Variable modifications: None
charge: 2 Observed m/z: 631.3457

Actual minus calculated peptide mass (AMU): 0.0008545

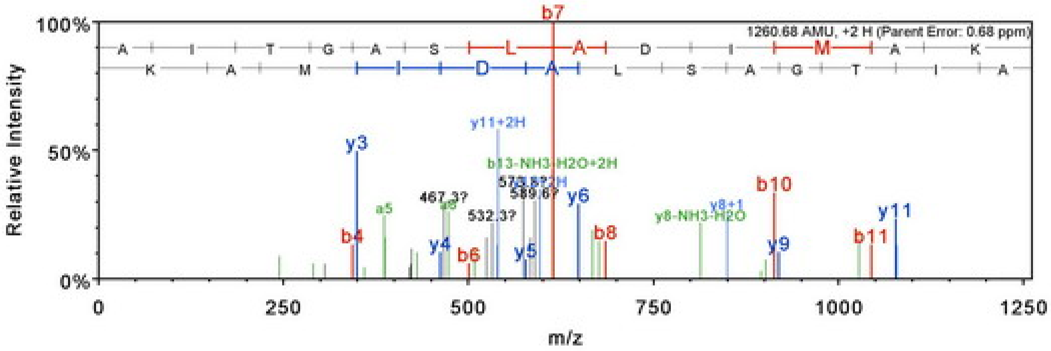

B	B Ions	B+2H	B-NH3	B-H2O	AA	Y Ions	Y+2H	Y-NH3	Y-H2O	Y
1	72.0				A	1261.7	631.4	1244.7	1243.7	13
2	185.1				I	1190.7	595.8	1173.6	1172.6	12
3	286.2			268.2	T	1077.6	539.3	1060.5	1059.5	11
4	343.2			325.2	G	976.5	488.8	959.5	958.5	10
5	414.2			396.2	A	919.5	460.3	902.5	901.5	9
6	501.3	251.1		483.3	S	848.5	424.7	831.4	830.4	8
7	614.4	307.7		596.3	L	761.4	381.2	744.4	743.4	7
8	685.4	343.2		667.4	A	648.3	324.7	631.3	630.3	6
9	800.4	400.7		782.4	D	577.3		560.3	559.3	5
10	913.5	457.3		895.5	I	462.3		445.3		4
11	1044.5	522.8		1026.5	M	349.2		332.2		3
12	1115.6	558.3		1097.6	A	218.2		201.1		2
13	1261.7	631.4	1244.7	1243.7	K	147.1		130.1		1

Gene symbol: LOC676847;EG639162

Protein name: similar to ribosomal protein S15a

Protein accession numbers: IPI00113394,IPI00230660,IPI00749889,IPI00849692

Peptide sequence: (K)HGYIGEFEIIDDHR(A)

Exclusive (unique to this protein): TRUE

SEQUEST XCorr score: 2.72 SEQUEST DCn score: 0.589

Fix modifications: None

Variable modifications: None

charge: 3 Observed m/z: 567.6024

Actual minus calculated peptide mass (AMU): -0.011954

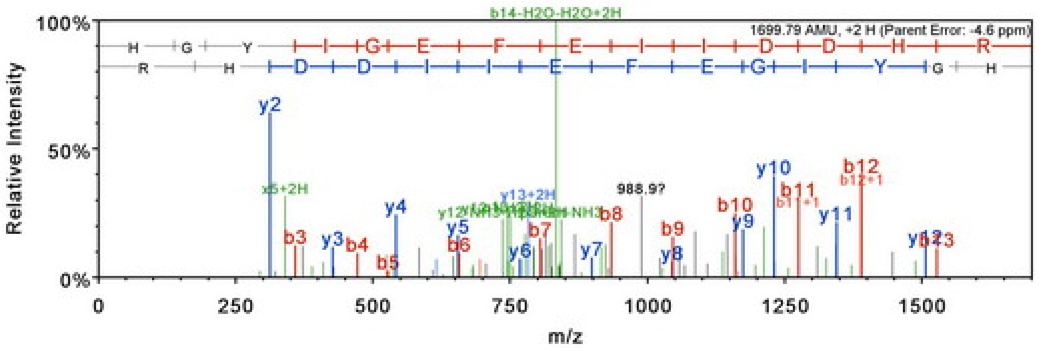

B	B Ions	B+2H	B-NH3	B-H2O	AA	Y Ions	Y+2H	Y-NH3	Y-H2O	Y
1	138.1	69.5			H	1700.8	850.9	1683.8	1682.8	14
2	195.1	98.0			G	1563.7	782.4	1546.7	1545.7	13
3	358.2	179.6			Y	1506.7	753.9	1489.7	1488.7	12
4	471.2	236.1			I	1343.7	672.3	1326.6	1325.7	11
5	528.3	264.6			G	1230.6	615.8	1213.5	1212.6	10
6	657.3	329.2		639.3	E	1173.5	587.3	1156.5	1155.5	9
7	804.4	402.7		786.4	F	1044.5	522.8	1027.5	1026.5	8
8	933.4	467.2		915.4	E	897.4	449.2	880.4	879.4	7
9	1046.5	523.8		1028.5	I	768.4	384.7	751.4	750.4	6
10	1159.6	580.3		1141.6	I	655.3	328.2	638.3	637.3	5
11	1274.6	637.8		1256.6	D	542.2	271.6	525.2	524.2	4
12	1389.6	695.3		1371.6	D	427.2	214.1	410.2	409.2	3
13	1526.7	763.9		1508.7	H	312.2	156.6	295.2		2
14	1700.8	850.9	1683.8	1682.8	R	175.1		158.1		1

Gene symbol: LOC677429;9630033F20Rik

Protein name: Uncharacterized protein C12orf5 homolog

Protein accession numbers: IPI00227451

Peptide sequence: (K)IIQGQGVDAPLSETGFR(Q)

Exclusive (unique to this protein): TRUE

SEQUEST XCorr score: 2.18 SEQUEST DCn score: 0.488

Fix modifications: None

Variable modifications: None

charge: 2 Observed m/z: 894.4644

Actual minus calculated peptide mass (AMU): -0.008423

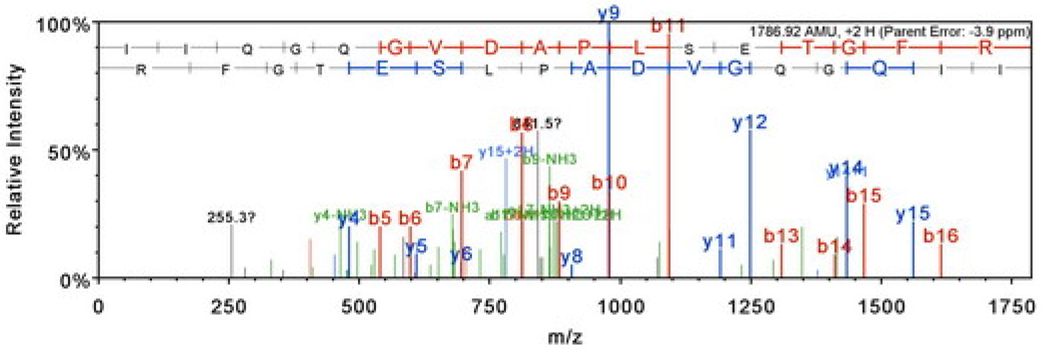

B	B Ions	B+2H	B-NH3	B-H2O	AA	Y Ions	Y+2H	Y-NH3	Y-H2O	Y
1	114.1				I	1787.9	894.5	1770.9	1769.9	17
2	227.2				I	1674.9	837.9	1657.8	1656.8	16
3	355.2		338.2		Q	1561.8	781.4	1544.7	1543.8	15
4	412.3		395.2		G	1433.7	717.4	1416.7	1415.7	14
5	540.3		523.3		Q	1376.7	688.8	1359.7	1358.7	13
6	597.3	299.2	580.3		G	1248.6	624.8	1231.6	1230.6	12
7	696.4	348.7	679.4		V	1191.6	596.3	1174.6	1173.6	11
8	811.4	406.2	794.4	793.4	D	1092.5	546.8	1075.5	1074.5	10
9	882.5	441.7	865.4	864.5	A	977.5	489.3	960.5	959.5	9
10	979.5	490.3	962.5	961.5	P	906.5	453.7	889.4	888.5	8
11	1092.6	546.8	1075.6	1074.6	L	809.4	405.2	792.4	791.4	7
12	1179.6	590.3	1162.6	1161.6	S	696.3	348.7	679.3	678.3	6
13	1308.7	654.8	1291.7	1290.7	E	609.3		592.3	591.3	5
14	1409.7	705.4	1392.7	1391.7	T	480.3		463.2	462.3	4
15	1466.8	733.9	1449.7	1448.7	G	379.2		362.2		3
16	1613.8	807.4	1596.8	1595.8	F	322.2		305.2		2
17	1787.9	894.5	1770.9	1769.9	R	175.1		158.1		1

Gene symbol: Lrpap1

Protein name: Alpha-2-macroglobulin receptor-associated protein precursor

Protein accession numbers: IPI00469307

Peptide sequence: (K)IQEYNVLLDTLSR(A)

Exclusive (unique to this protein): TRUE

SEQUEST XCorr score: 2.2 SEQUEST DCn score: 0.354

Fix modifications: None

Variable modifications: None

charge: 2 Observed m/z: 782.4194

Actual minus calculated peptide mass (AMU): -0.007568

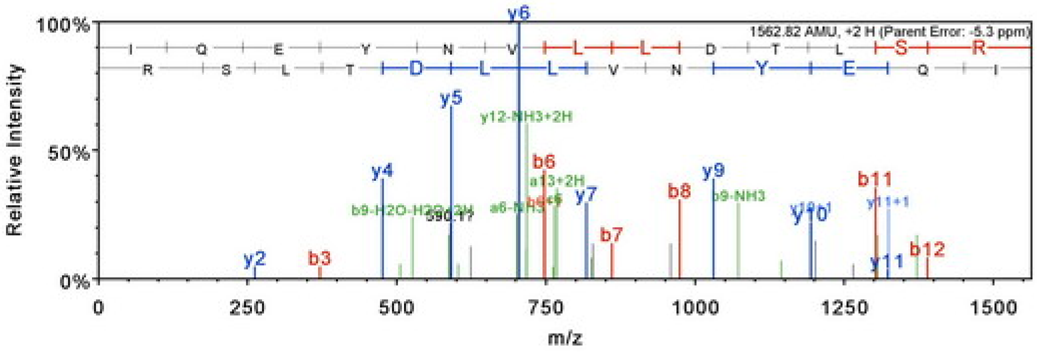

B	B Ions	B+2H	B-NH3	B-H2O	AA	Y Ions	Y+2H	Y-NH3	Y-H2O	Y
1	114.1				I	1563.8	782.4	1546.8	1545.8	13
2	242.2		225.1		Q	1450.8	725.9	1433.7	1432.7	12
3	371.2		354.2	353.2	E	1322.7	661.9	1305.7	1304.7	11
4	534.3		517.2	516.3	Y	1193.7	597.3	1176.6	1175.6	10
5	648.3		631.3	630.3	N	1030.6	515.8	1013.6	1012.6	9
6	747.4	374.2	730.3	729.4	V	916.6	458.8	899.5	898.5	8
7	860.5	430.7	843.4	842.4	L	817.5	409.2	800.5	799.5	7
8	973.5	487.3	956.5	955.5	L	704.4	352.7	687.4	686.4	6
9	1088.6	544.8	1071.5	1070.5	D	591.3		574.3	573.3	5
10	1189.6	595.3	1172.6	1171.6	T	476.3		459.3	458.3	4
11	1302.7	651.9	1285.7	1284.7	L	375.2		358.2	357.2	3
12	1389.7	695.4	1372.7	1371.7	S	262.1		245.1	244.1	2
13	1563.8	782.4	1546.8	1545.8	R	175.1		158.1		1

Gene symbol: Lrrc59

Protein name: Leucine-rich repeat-containing protein 59

Protein accession numbers: IPI00123281,IPI00653744

Peptide sequence: (R)LVTLPVSFAQLK(N)

Exclusive (unique to this protein): TRUE

SEQUEST XCorr score: 2.5 SEQUEST DCn score: 0.433

Fix modifications: None

Variable modifications: None

charge: 2 Observed m/z: 658.4033

Actual minus calculated peptide mass (AMU): -0.0003662

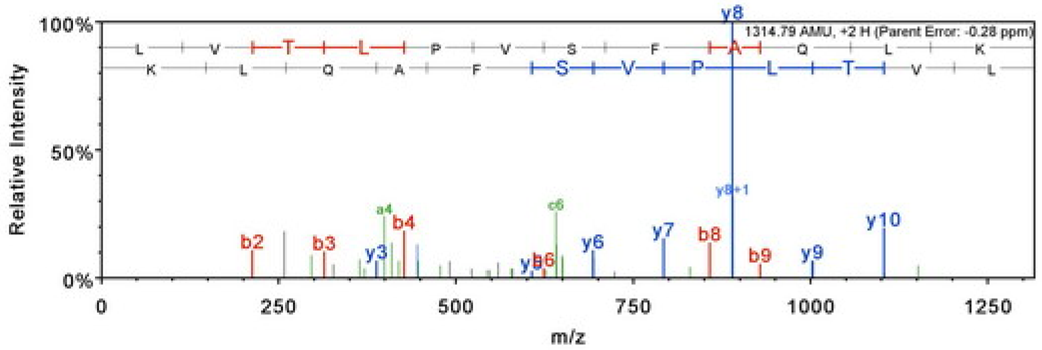

B	B Ions	B+2H	B-NH3	B-H2O	AA	Y Ions	Y+2H	Y-NH3	Y-H2O	Y
1	114.1				L	1315.8	658.4	1298.8	1297.8	12
2	213.2				V	1202.7	601.9	1185.7	1184.7	11
3	314.2			296.2	T	1103.7	552.3	1086.6	1085.6	10
4	427.3			409.3	L	1002.6	501.8	985.6	984.6	9
5	524.3			506.3	P	889.5	445.3	872.5	871.5	8
6	623.4	312.2		605.4	V	792.5	396.7	775.4	774.5	7
7	710.5	355.7		692.4	S	693.4	347.2	676.4	675.4	6
8	857.5	429.3		839.5	F	606.4		589.3		5
9	928.6	464.8		910.5	A	459.3		442.3		4
10	1056.6	528.8	1039.6	1038.6	Q	388.3		371.2		3
11	1169.7	585.4	1152.7	1151.7	L	260.2		243.2		2
12	1315.8	658.4	1298.8	1297.8	K	147.1		130.1		1

Gene symbol: Lsm1
Protein name: U6 snRNA-associated Sm-like protein LSm1
Protein accession numbers: IPI00121579
Peptide sequence: (R)SIDQFANLVLHQTVER(I)
Exclusive (unique to this protein): TRUE
SEQUEST XCorr score: 2.41 SEQUEST DCn score: 0.625
Fix modifications: None
Variable modifications: None
charge: 2 Observed m/z: 935.4913
Actual minus calculated peptide mass (AMU): -0.007568

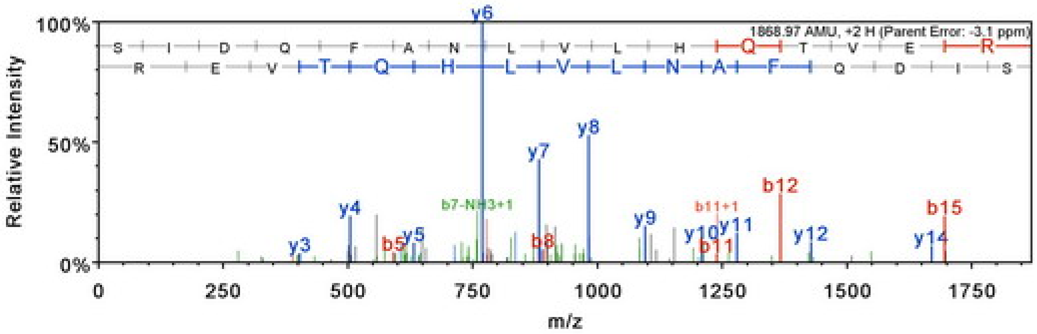

B	B Ions	B+2H	B-NH3	B-H2O	AA	Y Ions	Y+2H	Y-NH3	Y-H2O	Y
1	88.0			70.0	S	1870.0	935.5	1853.0	1852.0	16
2	201.1			183.1	I	1783.0	892.0	1765.9	1764.9	15
3	316.2			298.1	D	1669.9	835.4	1652.8	1651.9	14
4	444.2		427.2	426.2	Q	1554.8	777.9	1537.8	1536.8	13
5	591.3		574.3	573.3	F	1426.8	713.9	1409.8	1408.8	12
6	662.3	331.7	645.3	644.3	A	1279.7	640.4	1262.7	1261.7	11
7	776.4	388.7	759.3	758.4	N	1208.7	604.8	1191.7	1190.7	10
8	889.4	445.2	872.4	871.4	L	1094.6	547.8	1077.6	1076.6	9
9	988.5	494.8	971.5	970.5	V	981.6	491.3	964.5	963.5	8
10	1101.6	551.3	1084.6	1083.6	L	882.5	441.7	865.5	864.5	7
11	1238.7	619.8	1221.6	1220.6	H	769.4	385.2	752.4	751.4	6
12	1366.7	683.9	1349.7	1348.7	Q	632.3		615.3	614.3	5
13	1467.8	734.4	1450.7	1449.8	T	504.3		487.3	486.3	4
14	1566.8	783.9	1549.8	1548.8	V	403.2		386.2	385.2	3
15	1695.9	848.4	1678.8	1677.9	E	304.2		287.1	286.2	2
16	1870.0	935.5	1853.0	1852.0	R	175.1		158.1		1

Gene symbol: Lsm14b
Protein name: LSM14 protein homolog B
Protein accession numbers: IPI00229470
Peptide sequence: (R)YEGILYTIDTDNSTVALAK(V)
Exclusive (unique to this protein): TRUE
SEQUEST XCorr score: 2 SEQUEST DCn score: 0.585
Fix modifications: None

Variable modifications: None

charge: 2 Observed m/z: 1044.0276

Actual minus calculated peptide mass (AMU): -0.007568

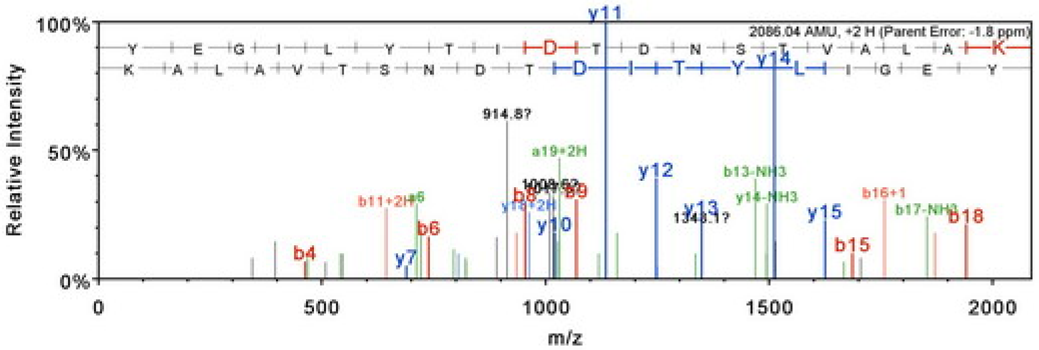

B	B Ions	B+2H	B-NH3	B-H2O	AA	Y Ions	Y+2H	Y-NH3	Y-H2O	Y
1	164.1				Y	2087.1	1044.0	2070.0	2069.0	19
2	293.1			275.1	E	1924.0	962.5	1907.0	1906.0	18
3	350.1			332.1	G	1795.0	898.0	1777.9	1776.9	17
4	463.2			445.2	I	1737.9	869.5	1720.9	1719.9	16
5	576.3			558.3	L	1624.8	812.9	1607.8	1606.8	15
6	739.4	370.2		721.4	Y	1511.8	756.4	1494.7	1493.8	14
7	840.4	420.7		822.4	T	1348.7	674.9	1331.7	1330.7	13
8	953.5	477.3		935.5	I	1247.7	624.3	1230.6	1229.6	12
9	1068.5	534.8		1050.5	D	1134.6	567.8	1117.5	1116.5	11
10	1169.6	585.3		1151.6	T	1019.5	510.3	1002.5	1001.5	10
11	1284.6	642.8		1266.6	D	918.5	459.8	901.5	900.5	9
12	1398.6	699.8	1381.6	1380.6	N	803.5	402.2	786.4	785.5	8
13	1485.7	743.3	1468.7	1467.7	S	689.4	345.2	672.4	671.4	7
14	1586.7	793.9	1569.7	1568.7	T	602.4	301.7	585.4	584.4	6
15	1685.8	843.4	1668.8	1667.8	V	501.3		484.3		5
16	1756.8	878.9	1739.8	1738.8	A	402.3		385.3		4
17	1869.9	935.5	1852.9	1851.9	L	331.2		314.2		3
18	1941.0	971.0	1923.9	1922.9	A	218.2		201.1		2
19	2087.1	1044.0	2070.0	2069.0	K	147.1		130.1		1

Gene symbol: Lsm2

Protein name: SnRNP core Sm protein homolog Sm-X5

Protein accession numbers: IPI00136054,IPI00461314

Peptide sequence: (R)YVQLPADEVDTQLLQDAAR(K)

Exclusive (unique to this protein): TRUE

SEQUEST XCorr score: 3.57 SEQUEST DCn score: 0.681

Fix modifications: None

Variable modifications: None

charge: 2 Observed m/z: 1073.0425

Actual minus calculated peptide mass (AMU): -0.005859

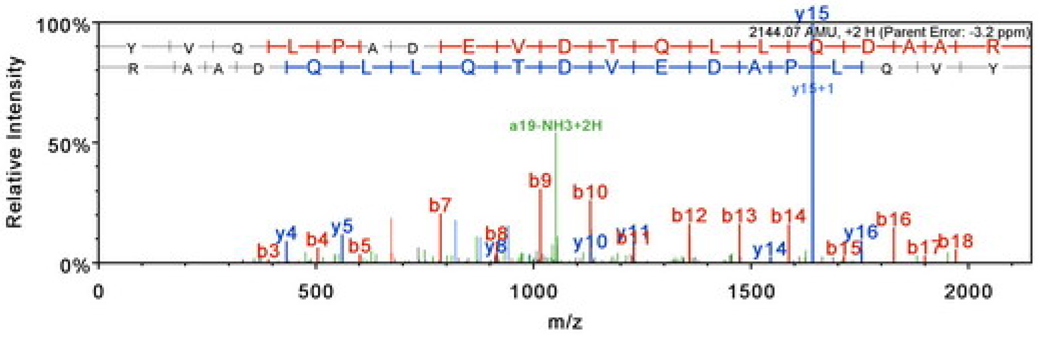

B	B Ions	B+2H	B-NH3	B-H2O	AA	Y Ions	Y+2H	Y-NH3	Y-H2O	Y
1	164.1				Y	2145.1	1073.0	2128.1	2127.1	19
2	263.1				V	1982.0	991.5	1965.0	1964.0	18
3	391.2		374.2		Q	1883.0	942.0	1865.9	1864.9	17
4	504.3		487.3		L	1754.9	878.0	1737.9	1736.9	16
5	601.3		584.3		P	1641.8	821.4	1624.8	1623.8	15
6	672.4	336.7	655.4		A	1544.8	772.9	1527.7	1526.8	14
7	787.4	394.2	770.4	769.4	D	1473.7	737.4	1456.7	1455.7	13
8	916.4	458.7	899.4	898.4	E	1358.7	679.9	1341.7	1340.7	12
9	1015.5	508.3	998.5	997.5	V	1229.7	615.3	1212.6	1211.6	11
10	1130.5	565.8	1113.5	1112.5	D	1130.6	565.8	1113.5	1112.6	10
11	1231.6	616.3	1214.6	1213.6	T	1015.6	508.3	998.5	997.5	9
12	1359.6	680.3	1342.6	1341.6	Q	914.5	457.8	897.5	896.5	8
13	1472.7	736.9	1455.7	1454.7	L	786.5	393.7	769.4	768.4	7
14	1585.8	793.4	1568.8	1567.8	L	673.4	337.2	656.3	655.4	6
15	1713.9	857.4	1696.8	1695.9	Q	560.3		543.3	542.3	5
16	1828.9	915.0	1811.9	1810.9	D	432.2		415.2	414.2	4
17	1899.9	950.5	1882.9	1881.9	A	317.2		300.2		3
18	1971.0	986.0	1953.9	1953.0	A	246.2		229.1		2
19	2145.1	1073.0	2128.1	2127.1	R	175.1		158.1		1

Gene symbol: Lsm3

Protein name: U6 snRNA-associated Sm-like protein LSm3

Protein accession numbers: IPI00230388

Peptide sequence: (R)GDGVVLVAPPLR(V)

Exclusive (unique to this protein): TRUE

SEQUEST XCorr score: 2.62 SEQUEST DCn score: 0.589

Fix modifications: None

Variable modifications: None

charge: 2 Observed m/z: 596.8527

Actual minus calculated peptide mass (AMU): -0.008057

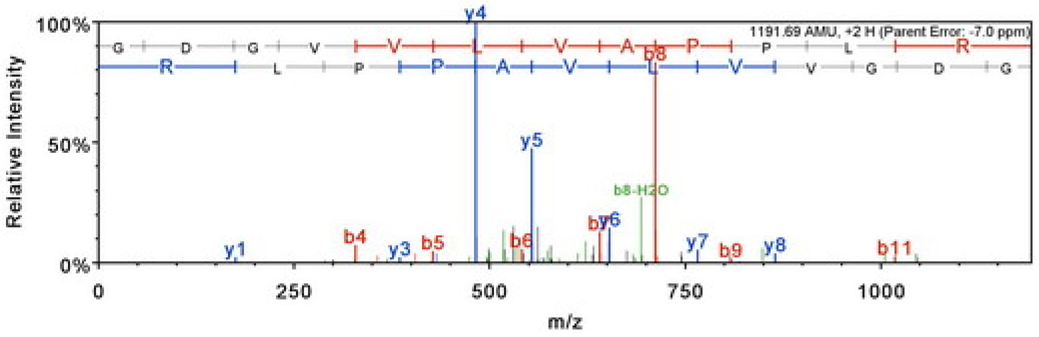

B	B Ions	B+2H	B-NH3	B-H2O	AA	Y Ions	Y+2H	Y-NH3	Y-H2O	Y
1	58.0				G	1192.7	596.9	1175.7	1174.7	12
2	173.1			155.1	D	1135.7	568.4	1118.7	1117.7	11
3	230.1			212.1	G	1020.7	510.8	1003.6		10
4	329.2			311.1	V	963.6	482.3	946.6		9
5	428.2			410.2	V	864.6	432.8	847.5		8
6	541.3	271.1		523.3	L	765.5	383.3	748.5		7
7	640.4	320.7		622.4	V	652.4	326.7	635.4		6
8	711.4	356.2		693.4	A	553.4		536.3		5
9	808.5	404.7		790.5	P	482.3		465.3		4
10	905.5	453.3		887.5	P	385.3		368.2		3
11	1018.6	509.8		1000.6	L	288.2		271.2		2
12	1192.7	596.9	1175.7	1174.7	R	175.1		158.1		1

Gene symbol: Lsm4

Protein name: 13 days embryo liver cDNA, RIKEN full-length enriched library, clone:2510005B08 product:U6 snRNA-associated SM-like protein 4, full insert sequence

Protein accession numbers: IPI00134784

Peptide sequence: (R)IPDEIIDMVR(E)

Exclusive (unique to this protein): TRUE

SEQUEST XCorr score: 2.1 SEQUEST DCn score: 0.467

Fix modifications: None

Variable modifications: None

charge: 2 Observed m/z: 600.8154

Actual minus calculated peptide mass (AMU): -0.0070758

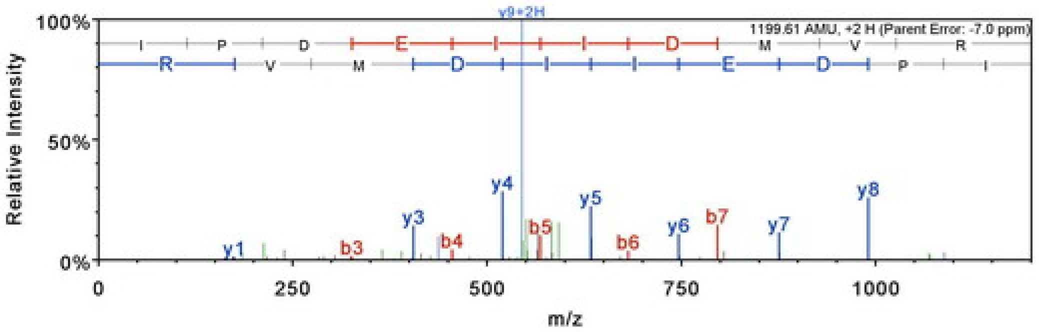

B	B Ions	B+2H	B-NH3	B-H2O	AA	Y Ions	Y+2H	Y-NH3	Y-H2O	Y
1	114.1				I	1200.6	600.8	1183.6	1182.6	10
2	211.1				P	1087.5	544.3	1070.5	1069.5	9
3	326.2			308.2	D	990.5	495.8	973.5	972.5	8
4	455.2			437.2	E	875.5	438.2	858.4	857.5	7
5	568.3			550.3	I	746.4	373.7	729.4	728.4	6
6	681.4	341.2		663.4	I	633.3		616.3	615.3	5
7	796.4	398.7		778.4	D	520.3		503.2	502.2	4
8	927.5	464.2		909.4	M	405.2		388.2		3
9	1026.5	513.8		1008.5	V	274.2		257.2		2
10	1200.6	600.8	1183.6	1182.6	R	175.1		158.1		1

Gene symbol: Ltb4dh

Protein name: NADP-dependent leukotriene B4 12-hydroxydehydrogenase

Protein accession numbers: IPI00131887

Peptide sequence: (R)TGPCPQGPAPEVVIYQQLR(M)

Exclusive (unique to this protein): TRUE

SEQUEST XCorr score: 2.06 SEQUEST DCn score: 0.639

Fix modifications: C4: Carbamidomethyl (+57.02)Variable modifications: None

charge: 2 Observed m/z: 1055.5388

Actual minus calculated peptide mass (AMU): -0.005859

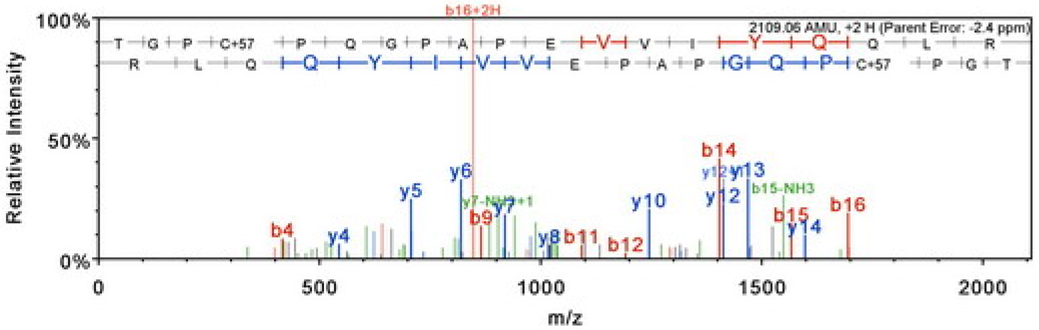

B	B Ions	B+2H	B-NH3	B-H2O	AA	Y Ions	Y+2H	Y-NH3	Y-H2O	Y
1	102.1			84.0	T	2110.1	1055.5	2093.1	2092.1	19
2	159.1			141.1	G	2009.0	1005.0	1992.0	1991.0	18
3	256.1			238.1	P	1952.0	976.5	1935.0	1934.0	17
4	416.2			398.2	C+57	1855.0	928.0	1837.9	1836.9	16
5	513.2			495.2	P	1694.9	848.0	1677.9	1676.9	15
6	641.3	321.1	624.3	623.3	Q	1597.9	799.4	1580.8	1579.9	14
7	698.3	349.7	681.3	680.3	G	1469.8	735.4	1452.8	1451.8	13
8	795.4	398.2	778.3	777.3	P	1412.8	706.9	1395.8	1394.8	12
9	866.4	433.7	849.4	848.4	A	1315.7	658.4	1298.7	1297.7	11
10	963.4	482.2	946.4	945.4	P	1244.7	622.9	1227.7	1226.7	10
11	1092.5	546.7	1075.5	1074.5	E	1147.7	574.3	1130.6	1129.6	9
12	1191.5	596.3	1174.5	1173.5	V	1018.6	509.8	1001.6		8
13	1290.6	645.8	1273.6	1272.6	V	919.5	460.3	902.5		7
14	1403.7	702.4	1386.7	1385.7	I	820.5	410.7	803.4		6
15	1566.8	783.9	1549.7	1548.8	Y	707.4		690.4		5
16	1694.8	847.9	1677.8	1676.8	Q	544.3		527.3		4
17	1822.9	911.9	1805.9	1804.9	Q	416.3		399.2		3
18	1936.0	968.5	1918.9	1918.0	L	288.2		271.2		2
19	2110.1	1055.5	2093.1	2092.1	R	175.1		158.1		1

Gene symbol: Lyrm4

Protein name: LYR motif-containing protein 4

Protein accession numbers: IPI00169804

Peptide sequence: (K)NVKDPVEIQALVNK(A)

Exclusive (unique to this protein): TRUE

SEQUEST XCorr score: 2.01 SEQUEST DCn score: 0.592

Fix modifications: None

Variable modifications: None

charge: 2 Observed m/z: 783.9481

Actual minus calculated peptide mass (AMU): 0.0028056

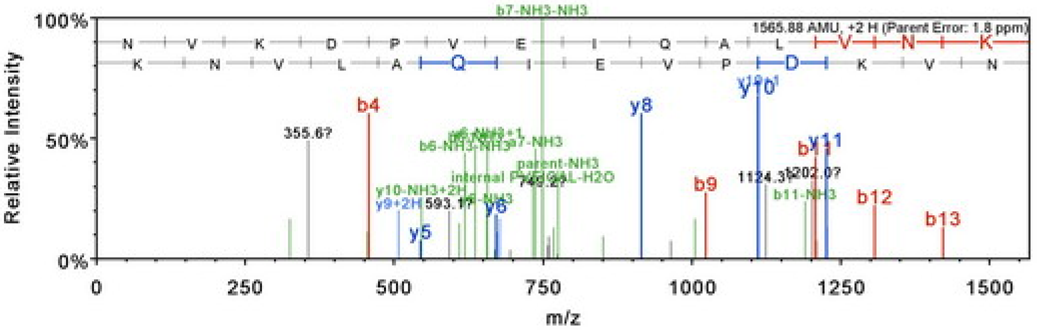

B	B Ions	B+2H	B-NH3	B-H2O	AA	Y Ions	Y+2H	Y-NH3	Y-H2O	Y
1	115.1		98.0		N	1566.9	784.0	1549.9	1548.9	14
2	214.1		197.1		V	1452.8	726.9	1435.8	1434.8	13
3	342.2	171.6	325.2		K	1353.8	677.4	1336.8	1335.8	12
4	457.2	229.1	440.2	439.2	D	1225.7	613.3	1208.7	1207.7	11
5	554.3	277.7	537.3	536.3	P	1110.7	555.8	1093.6	1092.6	10
6	653.4	327.2	636.3	635.4	V	1013.6	507.3	996.6	995.6	9
7	782.4	391.7	765.4	764.4	E	914.5	457.8	897.5	896.5	8
8	895.5	448.3	878.5	877.5	I	785.5	393.3	768.5		7
9	1023.6	512.3	1006.5	1005.5	Q	672.4	336.7	655.4		6
10	1094.6	547.8	1077.6	1076.6	A	544.4		527.3		5
11	1207.7	604.3	1190.6	1189.7	L	473.3		456.3		4
12	1306.7	653.9	1289.7	1288.7	V	360.2		343.2		3
13	1420.8	710.9	1403.8	1402.8	N	261.2		244.1		2
14	1566.9	784.0	1549.9	1548.9	K	147.1		130.1		1

Gene symbol: Lyz

Protein name: Lysozyme C type P precursor

Protein accession numbers: IPI00113427

Peptide sequence: (R)STDYGIFQINSR(Y)

Exclusive (unique to this protein): TRUE

SEQUEST XCorr score: 3.06 SEQUEST DCn score: 0.549

Fix modifications: None

Variable modifications: None

charge: 2 Observed m/z: 700.8396

Actual minus calculated peptide mass (AMU): -0.009644

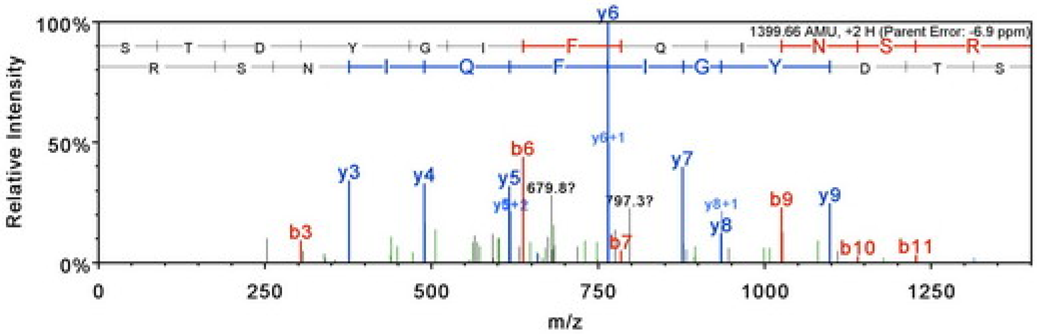

B	B Ions	B+2H	B-NH3	B-H2O	AA	Y Ions	Y+2H	Y-NH3	Y-H2O	Y
1	88.0			70.0	S	1400.7	700.8	1383.7	1382.7	12
2	189.1			171.1	T	1313.7	657.3	1296.6	1295.6	11
3	304.1			286.1	D	1212.6	606.8	1195.6	1194.6	10
4	467.2			449.2	Y	1097.6	549.3	1080.5	1079.6	9
5	524.2			506.2	G	934.5	467.8	917.5	916.5	8
6	637.3	319.2		619.3	I	877.5	439.3	860.5	859.5	7
7	784.4	392.7		766.3	F	764.4	382.7	747.4	746.4	6
8	912.4	456.7	895.4	894.4	Q	617.3		600.3	599.3	5
9	1025.5	513.3	1008.5	1007.5	I	489.3		472.3	471.3	4
10	1139.5	570.3	1122.5	1121.5	N	376.2		359.2	358.2	3
11	1226.6	613.8	1209.5	1208.6	S	262.1		245.1	244.1	2
12	1400.7	700.8	1383.7	1382.7	R	175.1		158.1		1

Gene symbol: Mad2l1
Protein name: Mitotic spindle assembly checkpoint protein MAD2A
Protein accession numbers: IPI00323422
Peptide sequence: (K)WEESGPQFITNCEEVR(L)
Exclusive (unique to this protein): TRUE
SEQUEST XCorr score: 2.02 SEQUEST DCn score: 0.579
Fix modifications: C12: Carbamidomethyl (+57.02)Variable modifications: None
charge: 2 Observed m/z: 990.9389
Actual minus calculated peptide mass (AMU): -0.006104

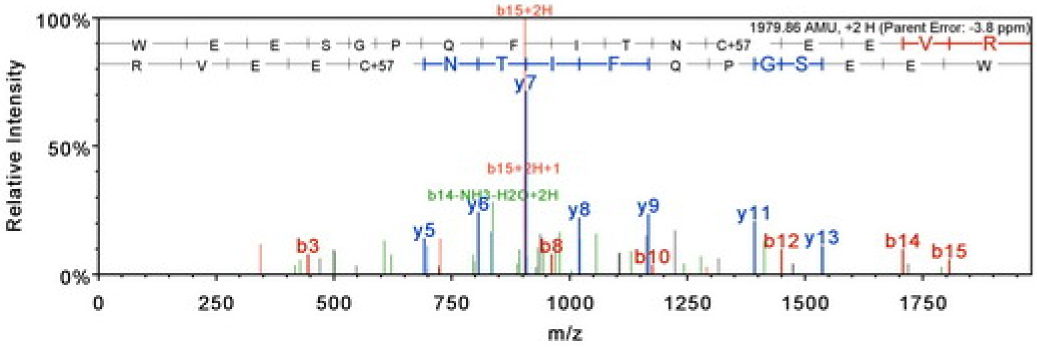

B	B Ions	B+2H	B-NH3	B-H2O	AA	Y Ions	Y+2H	Y-NH3	Y-H2O	Y
1	187.1				W	1980.9	990.9	1963.9	1962.9	16
2	316.1			298.1	E	1794.8	897.9	1777.8	1776.8	15
3	445.2			427.2	E	1665.8	833.4	1648.7	1647.7	14
4	532.2			514.2	S	1536.7	768.9	1519.7	1518.7	13
5	589.2			571.2	G	1449.7	725.3	1432.7	1431.7	12
6	686.3	343.6		668.3	P	1392.7	696.8	1375.6	1374.7	11
7	814.3	407.7	797.3	796.3	Q	1295.6	648.3	1278.6	1277.6	10
8	961.4	481.2	944.4	943.4	F	1167.5	584.3	1150.5	1149.5	9
9	1074.5	537.8	1057.5	1056.5	I	1020.5	510.7	1003.5	1002.5	8
10	1175.5	588.3	1158.5	1157.5	T	907.4	454.2	890.4	889.4	7
11	1289.6	645.3	1272.5	1271.6	N	806.4	403.7	789.3	788.3	6
12	1449.6	725.3	1432.6	1431.6	C+57	692.3		675.3	674.3	5
13	1578.7	789.8	1561.6	1560.6	E	532.3		515.3	514.3	4
14	1707.7	854.4	1690.7	1689.7	E	403.2		386.2	385.2	3
15	1806.8	903.9	1789.7	1788.8	V	274.2		257.2		2
16	1980.9	990.9	1963.9	1962.9	R	175.1		158.1		1

Gene symbol: Magmas
Protein name: Mitochondrial import inner membrane translocase subunit TIM16
Protein accession numbers: IPI00133167,IPI00762013
Peptide sequence: (R)AGHQSAAASNLSGLSLQEAQQILNVSK(L)
Exclusive (unique to this protein): TRUE
SEQUEST XCorr score: 2.68 SEQUEST DCn score: 0.558
Fix modifications: None

Variable modifications: None

charge: 3 Observed m/z: 908.1392

Actual minus calculated peptide mass (AMU): -0.01001

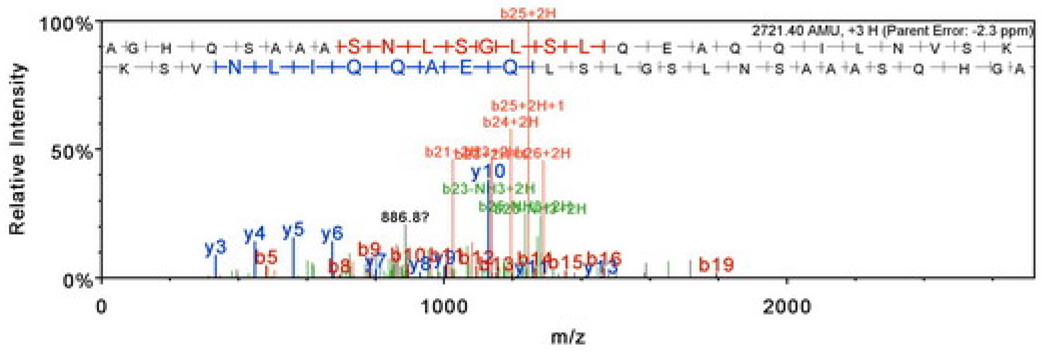

B	B Ions	B+2H	B-NH3	B-H2O	AA	Y Ions	Y+2H	Y-NH3	Y-H2O	Y
1	72.0				A	2722.4	1361.7	2705.4	2704.4	27
2	129.1				G	2651.4	1326.2	2634.3	2633.4	26
3	266.1	133.6			H	2594.3	1297.7	2577.3	2576.3	25
4	394.2	197.6	377.2		Q	2457.3	1229.2	2440.3	2439.3	24
5	481.2	241.1	464.2	463.2	S	2329.2	1165.1	2312.2	2311.2	23
6	552.3	276.6	535.2	534.2	A	2242.2	1121.6	2225.2	2224.2	22
7	623.3	312.2	606.3	605.3	A	2171.2	1086.1	2154.1	2153.2	21
8	694.3	347.7	677.3	676.3	A	2100.1	1050.6	2083.1	2082.1	20
9	781.4	391.2	764.3	763.4	S	2029.1	1015.1	2012.1	2011.1	19
10	895.4	448.2	878.4	877.4	N	1942.1	971.5	1925.0	1924.1	18
11	1008.5	504.8	991.5	990.5	L	1828.0	914.5	1811.0	1810.0	17
12	1095.5	548.3	1078.5	1077.5	S	1714.9	858.0	1697.9	1696.9	16
13	1152.5	576.8	1135.5	1134.5	G	1627.9	814.5	1610.9	1609.9	15
14	1265.6	633.3	1248.6	1247.6	L	1570.9	785.9	1553.9	1552.9	14
15	1352.7	676.8	1335.6	1334.7	S	1457.8	729.4	1440.8	1439.8	13
16	1465.7	733.4	1448.7	1447.7	L	1370.8	685.9	1353.7	1352.8	12
17	1593.8	797.4	1576.8	1575.8	Q	1257.7	629.3	1240.7	1239.7	11
18	1722.8	861.9	1705.8	1704.8	E	1129.6	565.3	1112.6	1111.6	10
19	1793.9	897.4	1776.9	1775.9	A	1000.6	500.8	983.6	982.6	9
20	1921.9	961.5	1904.9	1903.9	Q	929.5	465.3	912.5	911.5	8
21	2050.0	1025.5	2033.0	2032.0	Q	801.5	401.3	784.5	783.5	7
22	2163.1	1082.0	2146.1	2145.1	I	673.4	337.2	656.4	655.4	6
23	2276.2	1138.6	2259.1	2258.2	L	560.3		543.3	542.3	5
24	2390.2	1195.6	2373.2	2372.2	N	447.3		430.2	429.3	4
25	2489.3	1245.1	2472.3	2471.3	V	333.2		316.2	315.2	3
26	2576.3	1288.7	2559.3	2558.3	S	234.2		217.1	216.1	2
27	2722.4	1361.7	2705.4	2704.4	K	147.1		130.1		1

Gene symbol: Mapre1

Protein name: Microtubule-associated protein RP/EB family member 1

Protein accession numbers: IPI00117896

Peptide sequence: (R)NIELICQENEGENDPVLQR(I)

Exclusive (unique to this protein): TRUE

SEQUEST XCorr score: 2.94 SEQUEST DCn score: 0.71

Fix modifications: C6: Carbamidomethyl (+57.02)Variable modifications: None

charge: 2 Observed m/z: 1135.5449

Actual minus calculated peptide mass (AMU): 0.009522

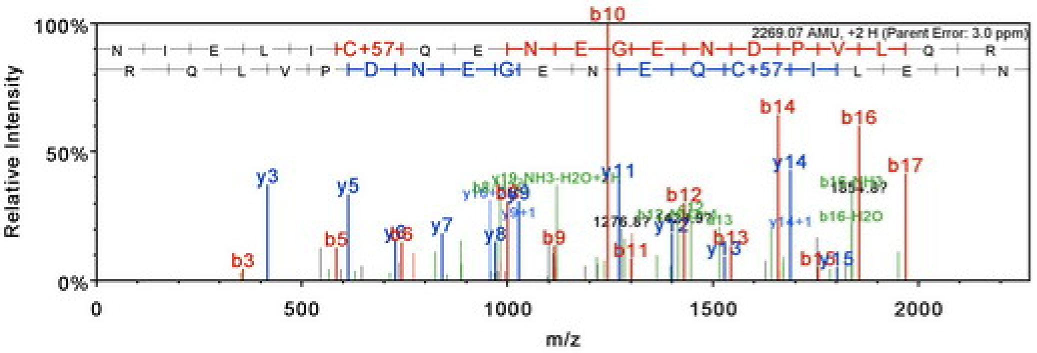

B	B Ions	B+2H	B-NH3	B-H2O	AA	Y Ions	Y+2H	Y-NH3	Y-H2O	Y
1	115.1		98.0		N	2270.1	1135.5	2253.1	2252.1	19
2	228.1		211.1		I	2156.0	1078.5	2139.0	2138.0	18
3	357.2		340.2	339.2	E	2043.0	1022.0	2025.9	2024.9	17
4	470.3		453.2	452.3	L	1913.9	957.5	1896.9	1895.9	16
5	583.4		566.3	565.3	I	1800.8	900.9	1783.8	1782.8	15
6	743.4	372.2	726.4	725.4	C+57	1687.7	844.4	1670.7	1669.7	14
7	871.4	436.2	854.4	853.4	Q	1527.7	764.4	1510.7	1509.7	13
8	1000.5	500.7	983.5	982.5	E	1399.7	700.3	1382.6	1381.6	12
9	1114.5	557.8	1097.5	1096.5	N	1270.6	635.8	1253.6	1252.6	11
10	1243.6	622.3	1226.5	1225.5	E	1156.6	578.8	1139.5	1138.5	10
11	1300.6	650.8	1283.6	1282.6	G	1027.5	514.3	1010.5	1009.5	9
12	1429.6	715.3	1412.6	1411.6	E	970.5	485.8	953.5	952.5	8
13	1543.7	772.3	1526.6	1525.7	N	841.5	421.2	824.4	823.4	7
14	1658.7	829.9	1641.7	1640.7	D	727.4	364.2	710.4	709.4	6
15	1755.8	878.4	1738.7	1737.7	P	612.4		595.4		5
16	1854.8	927.9	1837.8	1836.8	V	515.3		498.3		4
17	1967.9	984.5	1950.9	1949.9	L	416.3		399.2		3
18	2096.0	1048.5	2078.9	2077.9	Q	303.2		286.2		2
19	2270.1	1135.5	2253.1	2252.1	R	175.1		158.1		1

Gene symbol: Mapre2

Protein name: Isoform 1 of Microtubule-associated protein RP/EB family member 2

Protein accession numbers: IPI00403682,IPI00474073

Peptide sequence: (R)EIELLCQEHGQENDDLVQR(L)

Exclusive (unique to this protein): TRUE

SEQUEST XCorr score: 2.27 SEQUEST DCn score: 0.632

Fix modifications: C6: Carbamidomethyl (+57.02)Variable modifications: None

charge: 2 Observed m/z: 1163.0397

Actual minus calculated peptide mass (AMU): -0.006836

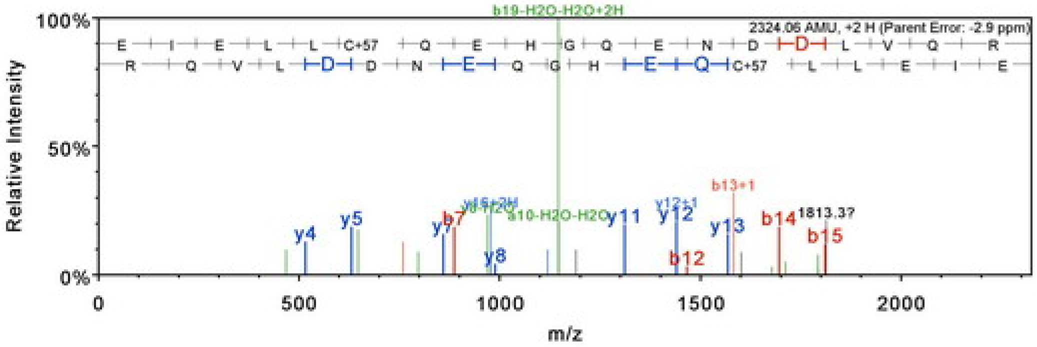

B	B Ions	B+2H	B-NH3	B-H2O	AA	Y Ions	Y+2H	Y-NH3	Y-H2O	Y
1	130.1			112.0	E	2325.1	1163.0	2308.1	2307.1	19
2	243.1			225.1	I	2196.0	1098.5	2179.0	2178.0	18
3	372.2			354.2	E	2082.9	1042.0	2065.9	2064.9	17
4	485.3			467.3	L	1953.9	977.5	1936.9	1935.9	16
5	598.4			580.3	L	1840.8	920.9	1823.8	1822.8	15
6	758.4	379.7		740.4	C+57	1727.7	864.4	1710.7	1709.7	14
7	886.4	443.7	869.4	868.4	Q	1567.7	784.4	1550.7	1549.7	13
8	1015.5	508.2	998.5	997.5	E	1439.7	720.3	1422.6	1421.6	12
9	1152.5	576.8	1135.5	1134.5	H	1310.6	655.8	1293.6	1292.6	11
10	1209.6	605.3	1192.5	1191.5	G	1173.5	587.3	1156.5	1155.5	10
11	1337.6	669.3	1320.6	1319.6	Q	1116.5	558.8	1099.5	1098.5	9
12	1466.7	733.8	1449.6	1448.7	E	988.5	494.7	971.4	970.5	8
13	1580.7	790.9	1563.7	1562.7	N	859.4	430.2	842.4	841.4	7
14	1695.7	848.4	1678.7	1677.7	D	745.4	373.2	728.4	727.4	6
15	1810.8	905.9	1793.7	1792.7	D	630.4		613.3	612.4	5
16	1923.8	962.4	1906.8	1905.8	L	515.3		498.3		4
17	2022.9	1012.0	2005.9	2004.9	V	402.3		385.2		3
18	2151.0	1076.0	2133.9	2133.0	Q	303.2		286.2		2
19	2325.1	1163.0	2308.1	2307.1	R	175.1		158.1		1

Gene symbol: Mif

Protein name: Macrophage migration inhibitory factor

Protein accession numbers: IPI00230427

Peptide sequence: (R)ASVPEGFLSELTQQLAQATGK(P)

Exclusive (unique to this protein): TRUE

SEQUEST XCorr score: 3.17 SEQUEST DCn score: 0.556

Fix modifications: None

Variable modifications: None

charge: 2 Observed m/z: 1088.0715

Actual minus calculated peptide mass (AMU): 0.005371

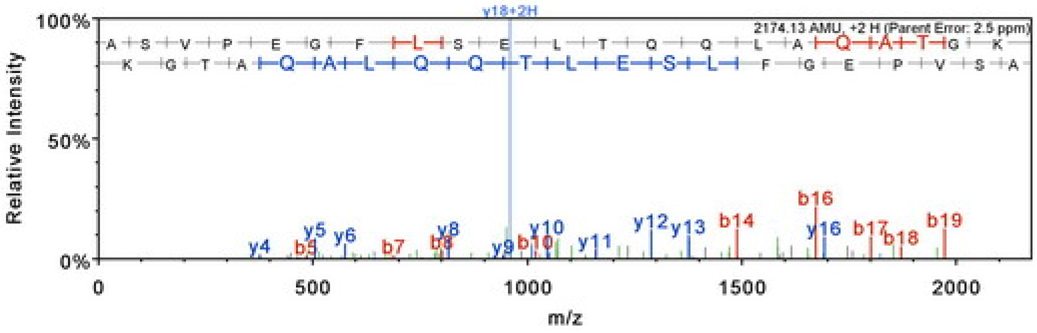

B	B Ions	B+2H	B-NH3	B-H2O	AA	Y Ions	Y+2H	Y-NH3	Y-H2O	Y
1	72.0				A	2175.1	1088.1	2158.1	2157.1	21
2	159.1			141.1	S	2104.1	1052.5	2087.1	2086.1	20
3	258.1			240.1	V	2017.1	1009.0	2000.0	1999.1	19
4	355.2			337.2	P	1918.0	959.5	1901.0	1900.0	18
5	484.2			466.2	E	1820.9	911.0	1803.9	1802.9	17
6	541.3	271.1		523.3	G	1691.9	846.5	1674.9	1673.9	16
7	688.3	344.7		670.3	F	1634.9	817.9	1617.9	1616.9	15
8	801.4	401.2		783.4	L	1487.8	744.4	1470.8	1469.8	14
9	888.5	444.7		870.4	S	1374.7	687.9	1357.7	1356.7	13
10	1017.5	509.3		999.5	E	1287.7	644.4	1270.7	1269.7	12
11	1130.6	565.8		1112.6	L	1158.7	579.8	1141.6	1140.6	11
12	1231.6	616.3		1213.6	T	1045.6	523.3	1028.5	1027.5	10
13	1359.7	680.3	1342.7	1341.7	Q	944.5	472.8	927.5	926.5	9
14	1487.7	744.4	1470.7	1469.7	Q	816.5	408.7	799.4	798.5	8
15	1600.8	800.9	1583.8	1582.8	L	688.4	344.7	671.4	670.4	7
16	1671.9	836.4	1654.8	1653.9	A	575.3	288.2	558.3	557.3	6
17	1799.9	900.5	1782.9	1781.9	Q	504.3		487.3	486.3	5
18	1871.0	936.0	1853.9	1852.9	A	376.2		359.2	358.2	4
19	1972.0	986.5	1955.0	1954.0	T	305.2		288.2	287.2	3
20	2029.0	1015.0	2012.0	2011.0	G	204.1		187.1		2
21	2175.1	1088.1	2158.1	2157.1	K	147.1		130.1		1

Gene symbol: Mllt4

Protein name: myeloid/lymphoid or mixed lineage-leukemia translocation to 4 homolog isoform 1

Protein accession numbers: IPI00137836,IPI00660193,IPI00753111,IPI00845596

Peptide sequence: (R)TQDATGPELILPASIEFR(E)

Exclusive (unique to this protein): TRUE

SEQUEST XCorr score: 2.21 SEQUEST DCn score: 0.301

Fix modifications: None

Variable modifications: None

charge: 2 Observed m/z: 979.5111

Actual minus calculated peptide mass (AMU): -0.009522

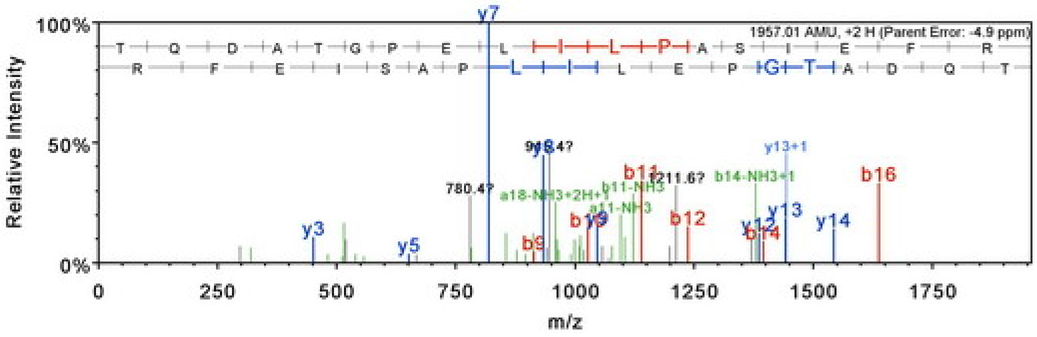

B	B Ions	B+2H	B-NH3	B-H2O	AA	Y Ions	Y+2H	Y-NH3	Y-H2O	Y
1	102.1			84.0	T	1958.0	979.5	1941.0	1940.0	18
2	230.1		213.1	212.1	Q	1857.0	929.0	1840.0	1839.0	17
3	345.1		328.1	327.1	D	1728.9	865.0	1711.9	1710.9	16
4	416.2		399.2	398.2	A	1613.9	807.5	1596.9	1595.9	15
5	517.2		500.2	499.2	T	1542.9	771.9	1525.8	1524.8	14
6	574.3	287.6	557.2	556.2	G	1441.8	721.4	1424.8	1423.8	13
7	671.3	336.2	654.3	653.3	P	1384.8	692.9	1367.8	1366.8	12
8	800.3	400.7	783.3	782.3	E	1287.7	644.4	1270.7	1269.7	11
9	913.4	457.2	896.4	895.4	L	1158.7	579.9	1141.7	1140.7	10
10	1026.5	513.8	1009.5	1008.5	I	1045.6	523.3	1028.6	1027.6	9
11	1139.6	570.3	1122.6	1121.6	L	932.5	466.8	915.5	914.5	8
12	1236.7	618.8	1219.6	1218.6	P	819.4	410.2	802.4	801.4	7
13	1307.7	654.4	1290.7	1289.7	A	722.4	361.7	705.4	704.4	6
14	1394.7	697.9	1377.7	1376.7	S	651.4		634.3	633.3	5
15	1507.8	754.4	1490.8	1489.8	I	564.3		547.3	546.3	4
16	1636.8	818.9	1619.8	1618.8	E	451.2		434.2	433.2	3
17	1783.9	892.5	1766.9	1765.9	F	322.2		305.2		2
18	1958.0	979.5	1941.0	1940.0	R	175.1		158.1		1

Gene symbol: Mtap

Protein name: S-methyl-5-thioadenosine phosphorylase

Protein accession numbers: IPI00132096

Peptide sequence: (K)IGIIGGTGLDDPEILEGR(T)

Exclusive (unique to this protein): TRUE

SEQUEST XCorr score: 2.45 SEQUEST DCn score: 0.662

Fix modifications: None

Variable modifications: None

charge: 2 Observed m/z: 912.9919

Actual minus calculated peptide mass (AMU): 0.004761

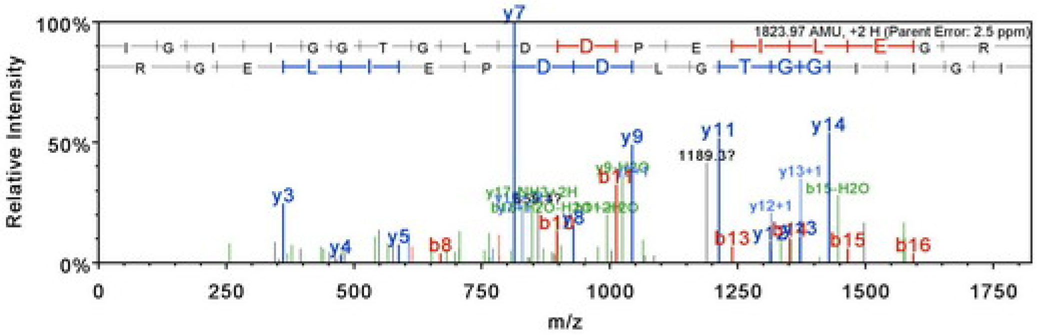

B	B Ions	B+2H	B-NH3	B-H2O	AA	Y Ions	Y+2H	Y-NH3	Y-H2O	Y
1	114.1				I	1825.0	913.0	1807.9	1807.0	18
2	171.1				G	1711.9	856.5	1694.9	1693.9	17
3	284.2				I	1654.9	827.9	1637.8	1636.9	16
4	397.3				I	1541.8	771.4	1524.8	1523.8	15
5	454.3				G	1428.7	714.9	1411.7	1410.7	14
6	511.3	256.2			G	1371.7	686.3	1354.7	1353.7	13
7	612.4	306.7		594.4	T	1314.7	657.8	1297.6	1296.6	12
8	669.4	335.2		651.4	G	1213.6	607.3	1196.6	1195.6	11
9	782.5	391.7		764.5	L	1156.6	578.8	1139.6	1138.6	10
10	897.5	449.3		879.5	D	1043.5	522.3	1026.5	1025.5	9
11	1012.5	506.8		994.5	D	928.5	464.7	911.5	910.5	8
12	1109.6	555.3		1091.6	P	813.5	407.2	796.4	795.4	7
13	1238.6	619.8		1220.6	E	716.4	358.7	699.4	698.4	6
14	1351.7	676.4		1333.7	I	587.4		570.3	569.3	5
15	1464.8	732.9		1446.8	L	474.3		457.2	456.3	4
16	1593.8	797.4		1575.8	E	361.2		344.2	343.2	3
17	1650.9	825.9		1632.9	G	232.1		215.1		2
18	1825.0	913.0	1807.9	1807.0	R	175.1		158.1		1

Gene symbol: Mtmr14

Protein name: Myotubularin-related protein 14 precursor

Protein accession numbers: IPI00396726

Peptide sequence: (R)FVCPVILFK(G)

Exclusive (unique to this protein): TRUE

SEQUEST XCorr score: 2 SEQUEST DCn score: 0.472

Fix modifications: C3: Carbamidomethyl (+57.02)Variable modifications: None

charge: 2 Observed m/z: 561.8202

Actual minus calculated peptide mass (AMU): -0.006226

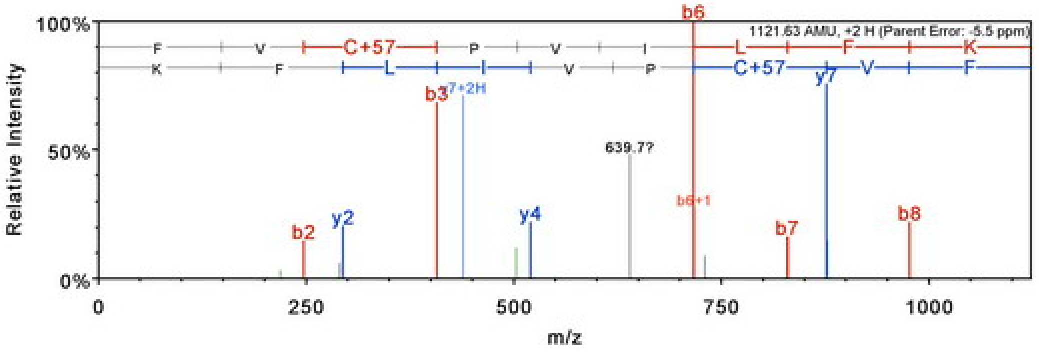

B	B Ions	B+2H	B-NH3	B-H2O	AA	Y Ions	Y+2H	Y-NH3	Y-H2O	Y
1	148.1				F	1122.6	561.8	1105.6		9
2	247.1				V	975.6	488.3	958.5		8
3	407.2				C+57	876.5	438.8	859.5		7
4	504.2				P	716.5	358.7	699.4		6
5	603.3				V	619.4		602.4		5
6	716.4	358.7			I	520.4		503.3		4
7	829.5	415.2			L	407.3		390.2		3
8	976.5	488.8			F	294.2		277.2		2
9	1122.6	561.8	1105.6		K	147.1		130.1		1

Gene symbol: Nans

Protein name: N-acetylneuraminic acid synthase

Protein accession numbers: IPI00114925

Peptide sequence: (K)GSDHSASLEPGELAELVR(S)

Exclusive (unique to this protein): TRUE

SEQUEST XCorr score: 2.44 SEQUEST DCn score: 0.627

Fix modifications: None

Variable modifications: None

charge: 2 Observed m/z: 933.9626

Actual minus calculated peptide mass (AMU): -0.002564

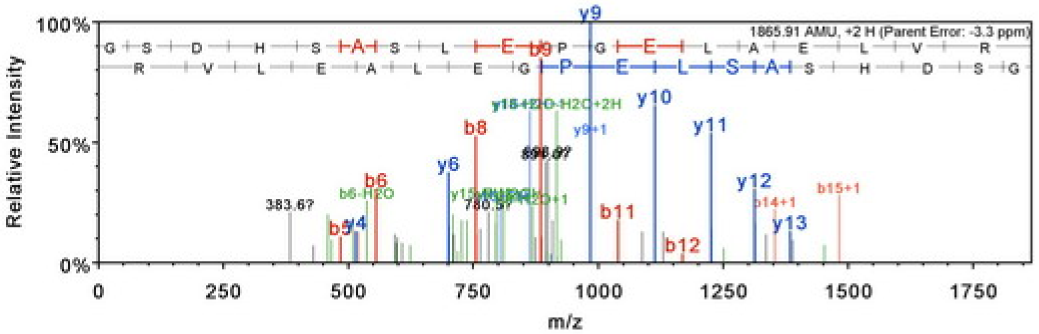

B	B Ions	B+2H	B-NH3	B-H2O	AA	Y Ions	Y+2H	Y-NH3	Y-H2O	Y
1	58.0				G	1866.9	934.0	1849.9	1848.9	18
2	145.1			127.1	S	1809.9	905.5	1792.9	1791.9	17
3	260.1			242.1	D	1722.9	861.9	1705.8	1704.9	16
4	397.2	199.1		379.1	H	1607.8	804.4	1590.8	1589.8	15
5	484.2	242.6		466.2	S	1470.8	735.9	1453.8	1452.8	14
6	555.2	278.1		537.2	A	1383.8	692.4	1366.7	1365.7	13
7	642.3	321.6		624.2	S	1312.7	656.9	1295.7	1294.7	12
8	755.3	378.2		737.3	L	1225.7	613.3	1208.7	1207.7	11
9	884.4	442.7		866.4	E	1112.6	556.8	1095.6	1094.6	10
10	981.4	491.2		963.4	P	983.6	492.3	966.5	965.5	9
11	1038.5	519.7		1020.4	G	886.5	443.8	869.5	868.5	8
12	1167.5	584.3		1149.5	E	829.5	415.2	812.5	811.5	7
13	1280.6	640.8		1262.6	L	700.4	350.7	683.4	682.4	6
14	1351.6	676.3		1333.6	A	587.4		570.3	569.3	5
15	1480.7	740.8		1462.7	E	516.3		499.3	498.3	4
16	1593.7	797.4		1575.7	L	387.3		370.3		3
17	1692.8	846.9		1674.8	V	274.2		257.2		2
18	1866.9	934.0	1849.9	1848.9	R	175.1		158.1		1

Gene symbol: Nat14

Protein name: Adult male olfactory brain cDNA, RIKEN full-length enriched library, clone:6430557O09 product:LEUCINE-ZIPPER-LIKE PROTEIN 1 homolog

Protein accession numbers: IPI00225865

Peptide sequence: (R)GSGDVCGVLALAPGANVGDGAR(V)

Exclusive (unique to this protein): TRUE

SEQUEST XCorr score: 2.6 SEQUEST DCn score: 0.672

Fix modifications: C6: Carbamidomethyl (+57.02)Variable modifications: None

charge: 2 Observed m/z: 1006.9979

Actual minus calculated peptide mass (AMU): 0.005371

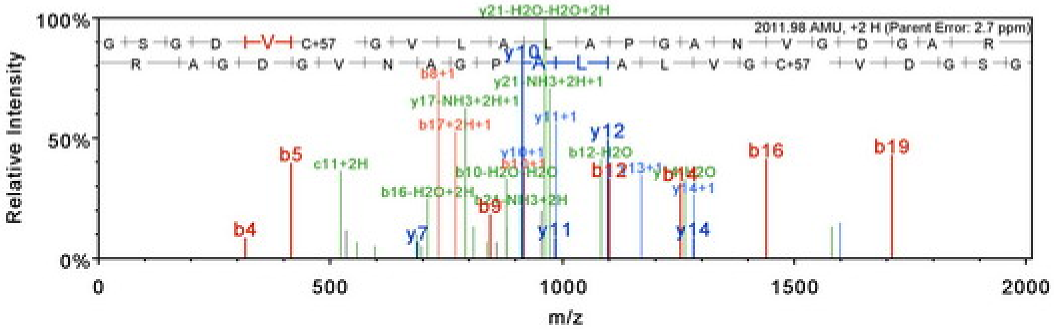

B	B Ions	B+2H	B-NH3	B-H2O	AA	Y Ions	Y+2H	Y-NH3	Y-H2O	Y
1	58.0				G	2013.0	1007.0	1996.0	1995.0	22
2	145.1			127.1	S	1956.0	978.5	1938.9	1938.0	21
3	202.1			184.1	G	1868.9	935.0	1851.9	1850.9	20
4	317.1			299.1	D	1811.9	906.5	1794.9	1793.9	19
5	416.2			398.2	V	1696.9	848.9	1679.9	1678.9	18
6	576.2	288.6		558.2	C+57	1597.8	799.4	1580.8	1579.8	17
7	633.2	317.1		615.2	G	1437.8	719.4	1420.8	1419.8	16
8	732.3	366.7		714.3	V	1380.8	690.9	1363.7	1362.8	15
9	845.4	423.2		827.4	L	1281.7	641.4	1264.7	1263.7	14
10	916.4	458.7		898.4	A	1168.6	584.8	1151.6	1150.6	13
11	1029.5	515.3		1011.5	L	1097.6	549.3	1080.5	1079.6	12
12	1100.5	550.8		1082.5	A	984.5	492.8	967.5	966.5	11
13	1197.6	599.3		1179.6	P	913.5	457.2	896.4	895.4	10
14	1254.6	627.8		1236.6	G	816.4	408.7	799.4	798.4	9
15	1325.7	663.3		1307.6	A	759.4	380.2	742.4	741.4	8
16	1439.7	720.4	1422.7	1421.7	N	688.3	344.7	671.3	670.3	7
17	1538.8	769.9	1521.7	1520.8	V	574.3	287.7	557.3	556.3	6
18	1595.8	798.4	1578.8	1577.8	G	475.2		458.2	457.2	5
19	1710.8	855.9	1693.8	1692.8	D	418.2		401.2	400.2	4
20	1767.8	884.4	1750.8	1749.8	G	303.2		286.2		3
21	1838.9	919.9	1821.8	1820.9	A	246.2		229.1		2
22	2013.0	1007.0	1996.0	1995.0	R	175.1		158.1		1

Gene symbol: Nbeal2

Protein name: mKIAA0540 protein

Protein accession numbers: IPI00421222,IPI00675407

Peptide sequence: (K)SSEDRMERNAVAAFLLMLR(N)
Exclusive (unique to this protein): TRUE
SEQUEST XCorr score: 2.5 SEQUEST DCn score: 0.328
Fix modifications: None
Variable modifications: M17: Oxidation (+16.00)
charge: 3 Observed m/z: 742.3746
Actual minus calculated peptide mass (AMU): -0.008789

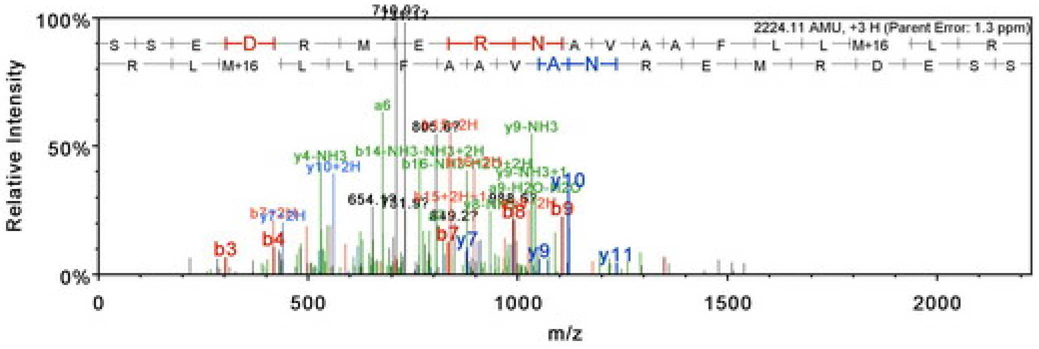

B	B Ions	B+2H	B-NH3	B-H2O	AA	Y Ions	Y+2H	Y-NH3	Y-H2O	Y
1	88.0			70.0	S	2225.1	1113.1	2208.1	2207.1	19
2	175.1			157.1	S	2138.1	1069.5	2121.1	2120.1	18
3	304.1			286.1	E	2051.1	1026.0	2034.0	2033.0	17
4	419.1			401.1	D	1922.0	961.5	1905.0	1904.0	16
5	575.2	288.1	558.2	557.2	R	1807.0	904.0	1790.0	1789.0	15
6	706.3	353.7	689.3	688.3	M	1650.9	826.0	1633.9	1632.9	14
7	835.3	418.2	818.3	817.3	E	1519.8	760.4	1502.8	1501.8	13
8	991.4	496.2	974.4	973.4	R	1390.8	695.9	1373.8		12
9	1105.5	553.2	1088.4	1087.5	N	1234.7	617.9	1217.7		11
10	1176.5	588.8	1159.5	1158.5	A	1120.7	560.8	1103.6		10
11	1275.6	638.3	1258.5	1257.6	V	1049.6	525.3	1032.6		9
12	1346.6	673.8	1329.6	1328.6	A	950.6	475.8	933.5		8
13	1417.7	709.3	1400.6	1399.6	A	879.5	440.3	862.5		7
14	1564.7	782.9	1547.7	1546.7	F	808.5	404.7	791.5		6
15	1677.8	839.4	1660.8	1659.8	L	661.4		644.4		5
16	1790.9	896.0	1773.9	1772.9	L	548.3		531.3		4
17	1937.9	969.5	1920.9	1919.9	M+16	435.2		418.2		3
18	2051.0	1026.0	2034.0	2033.0	L	288.2		271.2		2
19	2225.1	1113.1	2208.1	2207.1	R	175.1		158.1		1

Gene symbol: Ndufc2
Protein name: Adult male small intestine cDNA, RIKEN full-length enriched library, clone:2010300P09 product:1810004I06RIK PROTEIN (SIMILAR TO NADH DEHYDROGENASE (UBIQUINONE) 1, SUBCOMPLEX UNKNOWN, 2) (14.5KD, B14.5B) homolog
Protein accession numbers: IPI00110825
Peptide sequence: (K)TYAEILEPFHPVR
Exclusive (unique to this protein): TRUE
SEQUEST XCorr score: 2.08 SEQUEST DCn score: 0.573

Fix modifications: None

Variable modifications: None

charge: 2 Observed m/z: 786.411

Actual minus calculated peptide mass (AMU): -0.008057

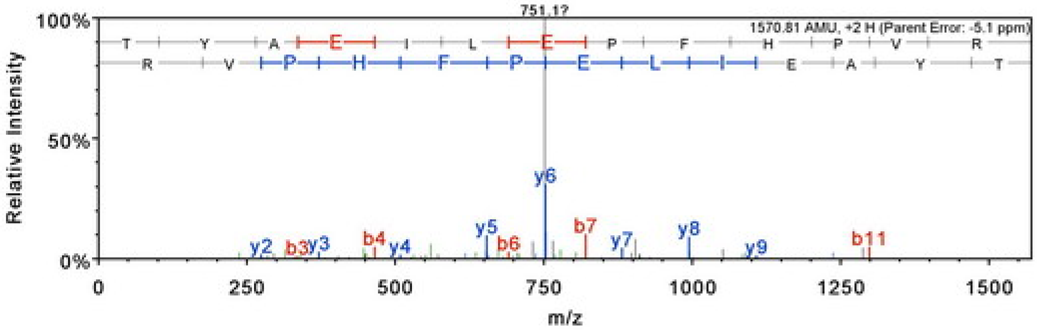

B	B Ions	B+2H	B-NH3	B-H2O	AA	Y Ions	Y+2H	Y-NH3	Y-H2O	Y
1	102.1			84.0	T	1571.8	786.4	1554.8	1553.8	13
2	265.1			247.1	Y	1470.8	735.9	1453.8	1452.8	12
3	336.2			318.2	A	1307.7	654.4	1290.7	1289.7	11
4	465.2			447.2	E	1236.7	618.8	1219.7	1218.7	10
5	578.3			560.3	I	1107.6	554.3	1090.6	1089.6	9
6	691.4	346.2		673.4	L	994.6	497.8	977.5	976.5	8
7	820.4	410.7		802.4	E	881.5	441.2	864.4	863.5	7
8	917.5	459.2		899.5	P	752.4	376.7	735.4		6
9	1064.5	532.8		1046.5	F	655.4	328.2	638.3		5
10	1201.6	601.3		1183.6	H	508.3	254.7	491.3		4
11	1298.6	649.8		1280.6	P	371.2		354.2		3
12	1397.7	699.4		1379.7	V	274.2		257.2		2
13	1571.8	786.4	1554.8	1553.8	R	175.1		158.1		1

Gene symbol: Ndufs5;LOC631712

Protein name: NADH dehydrogenase [ubiquinone] iron-sulfur protein 5

Protein accession numbers: IPI00117300,IPI00649019

Peptide sequence: (K)IEFDDFEECLLR(Y)

Exclusive (unique to this protein): TRUE

SEQUEST XCorr score: 2.26 SEQUEST DCn score: 0.753

Fix modifications: C9: Carbamidomethyl (+57.02)Variable modifications: None

charge: 2 Observed m/z: 793.3607

Actual minus calculated peptide mass (AMU): -0.007568

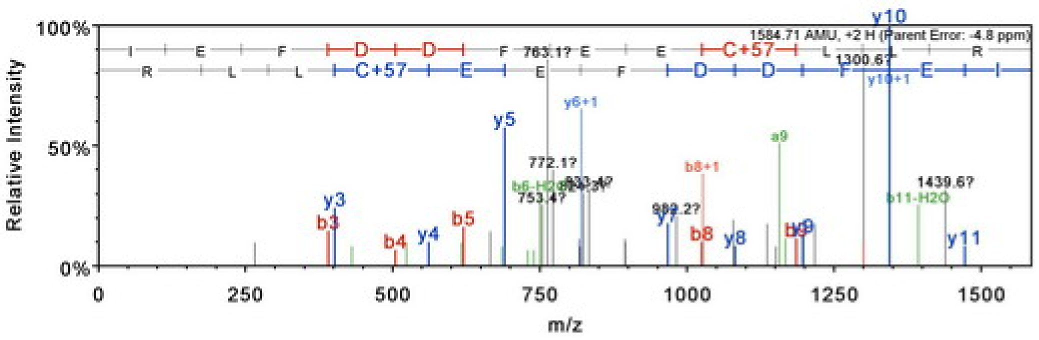

B	B Ions	B+2H	B-NH3	B-H2O	AA	Y Ions	Y+2H	Y-NH3	Y-H2O	Y
1	114.1				I	1585.7	793.4	1568.7	1567.7	12
2	243.1			225.1	E	1472.6	736.8	1455.6	1454.6	11
3	390.2			372.2	F	1343.6	672.3	1326.6	1325.6	10
4	505.2			487.2	D	1196.5	598.8	1179.5	1178.5	9
5	620.3			602.3	D	1081.5	541.3	1064.5	1063.5	8
6	767.3	384.2		749.3	F	966.5	483.7	949.5	948.5	7
7	896.4	448.7		878.4	E	819.4	410.2	802.4	801.4	6
8	1025.4	513.2		1007.4	E	690.4		673.3	672.4	5
9	1185.4	593.2		1167.4	C+57	561.3		544.3		4
10	1298.5	649.8		1280.5	L	401.3		384.3		3
11	1411.6	706.3		1393.6	L	288.2		271.2		2
12	1585.7	793.4	1568.7	1567.7	R	175.1		158.1		1

Gene symbol: Nit2

Protein name: Nit protein 2

Protein accession numbers: IPI00119945

Peptide sequence: (R)FAELAQIYAQR(G)

Exclusive (unique to this protein): TRUE

SEQUEST XCorr score: 2.32 SEQUEST DCn score: 0.473

Fix modifications: None

Variable modifications: None

charge: 2 Observed m/z: 655.3482

Actual minus calculated peptide mass (AMU): -0.0019551

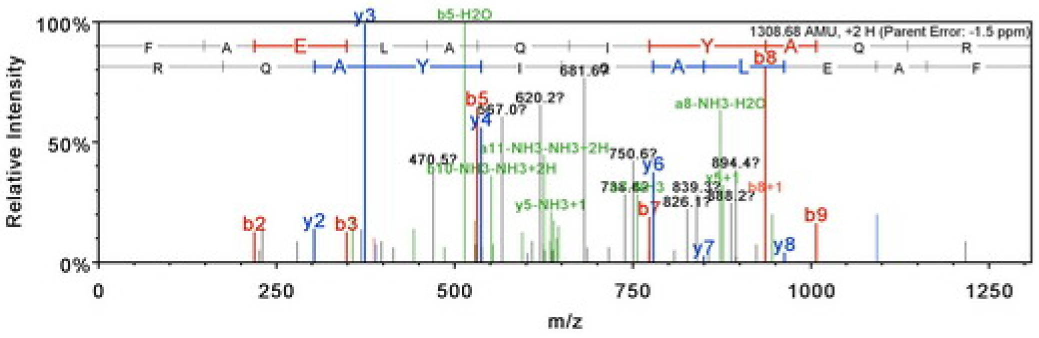

B	B Ions	B+2H	B-NH3	B-H2O	AA	Y Ions	Y+2H	Y-NH3	Y-H2O	Y
1	148.1				F	1309.7	655.4	1292.7	1291.7	11
2	219.1				A	1162.6	581.8	1145.6	1144.6	10
3	348.2			330.2	E	1091.6	546.3	1074.6	1073.6	9
4	461.2			443.2	L	962.5	481.8	945.5		8
5	532.3			514.3	A	849.5	425.2	832.4		7
6	660.3	330.7	643.3	642.3	Q	778.4	389.7	761.4		6
7	773.4	387.2	756.4	755.4	I	650.4		633.3		5
8	936.5	468.8	919.5	918.5	Y	537.3		520.3		4
9	1007.5	504.3	990.5	989.5	A	374.2		357.2		3
10	1135.6	568.3	1118.5	1117.6	Q	303.2		286.2		2
11	1309.7	655.4	1292.7	1291.7	R	175.1		158.1		1

Gene symbol: Nlrp4b

Protein name: NACHT, LRR and PYD domains-containing protein 4B

Protein accession numbers: IPI00226298

Peptide sequence: (R)NIAQEAFSLIR(E)

Exclusive (unique to this protein): TRUE

SEQUEST XCorr score: 2.1 SEQUEST DCn score: 0.663

Fix modifications: None

Variable modifications: None

charge: 2 Observed m/z: 631.3452

Actual minus calculated peptide mass (AMU): -0.007935

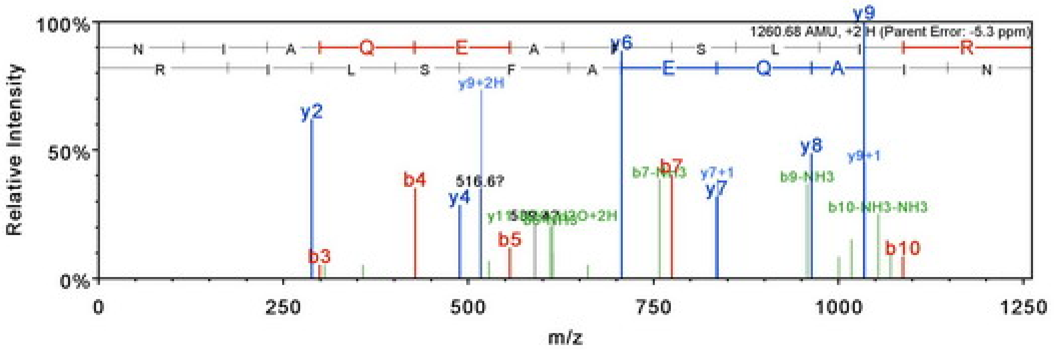

B	B Ions	B+2H	B-NH3	B-H2O	AA	Y Ions	Y+2H	Y-NH3	Y-H2O	Y
1	115.1		98.0		N	1261.7	631.4	1244.7	1243.7	11
2	228.1		211.1		I	1147.7	574.3	1130.6	1129.6	10
3	299.2		282.2		A	1034.6	517.8	1017.5	1016.6	9
4	427.2		410.2		Q	963.5	482.3	946.5	945.5	8
5	556.3		539.3	538.3	E	835.5	418.2	818.4	817.5	7
6	627.3	314.2	610.3	609.3	A	706.4	353.7	689.4	688.4	6
7	774.4	387.7	757.4	756.4	F	635.4		618.4	617.4	5
8	861.4	431.2	844.4	843.4	S	488.3		471.3	470.3	4
9	974.5	487.8	957.5	956.5	L	401.3		384.3		3
10	1087.6	544.3	1070.5	1069.6	I	288.2		271.2		2
11	1261.7	631.4	1244.7	1243.7	R	175.1		158.1		1

Gene symbol: Nlrp4f

Protein name: ES cells cDNA, RIKEN full-length enriched library, clone:C330026N02 product:hypothetical Leucine rich repeat, ribonuclease inhibitor type containing protein, full insert sequence

Protein accession numbers: IPI00226572

Peptide sequence: (K)TLSHLDISSNDLKDEGLK(I)

Exclusive (unique to this protein): TRUE

SEQUEST XCorr score: 2.03 SEQUEST DCn score: 0.603

Fix modifications: None

Variable modifications: None

charge: 2 Observed m/z: 993.0112

Actual minus calculated peptide mass (AMU): -0.004883

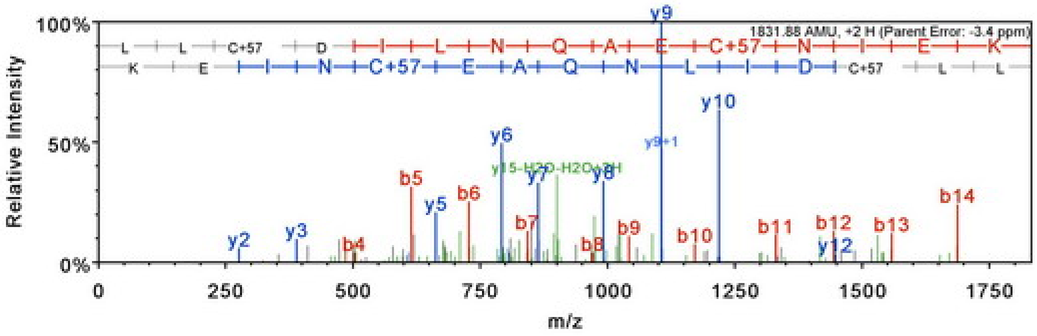

B	B Ions	B+2H	B-NH3	B-H2O	AA	Y Ions	Y+2H	Y-NH3	Y-H2O	Y
1	114.1				L	1832.9	917.0	1815.9	1814.9	15
2	227.2				L	1719.8	860.4	1702.8	1701.8	14
3	387.2				C+57	1606.7	803.9	1589.7	1588.7	13
4	502.2			484.2	D	1446.7	723.9	1429.7	1428.7	12
5	615.3			597.3	I	1331.7	666.3	1314.6	1313.7	11
6	728.4	364.7		710.4	L	1218.6	609.8	1201.5	1200.6	10
7	842.4	421.7	825.4	824.4	N	1105.5	553.3	1088.5	1087.5	9
8	970.5	485.8	953.5	952.5	Q	991.5	496.2	974.4	973.4	8
9	1041.5	521.3	1024.5	1023.5	A	863.4	432.2	846.4	845.4	7
10	1170.6	585.8	1153.6	1152.6	E	792.4	396.7	775.3	774.4	6
11	1330.6	665.8	1313.6	1312.6	C+57	663.3		646.3	645.3	5
12	1444.7	722.8	1427.6	1426.7	N	503.3		486.3	485.3	4
13	1557.7	779.4	1540.7	1539.7	I	389.2		372.2	371.2	3
14	1686.8	843.9	1669.8	1668.8	E	276.2		259.1	258.1	2
15	1832.9	917.0	1815.9	1814.9	K	147.1		130.1		1

Peptide sequence: (K)ETFIQNDYDAFENLLISK(G)

Exclusive (unique to this protein): TRUE

SEQUEST XCorr score: 2.56 SEQUEST DCn score: 0.665

Fix modifications: None

Variable modifications: None

charge: 2 Observed m/z: 1080.5265

Actual minus calculated peptide mass (AMU): -0.004883

Peptide sequence: (K)IFGSILMSSK(S)

Exclusive (unique to this protein): FALSE
SEQUEST XCorr score: 2.37 SEQUEST DCn score: 0.554
Fix modifications: None
Variable modifications: None
charge: 2 Observed m/z: 541.7973
Actual minus calculated peptide mass (AMU): -0.005249

Peptide sequence: (R)GCQDLAEVLK(N)
Exclusive (unique to this protein): FALSE
SEQUEST XCorr score: 2.43 SEQUEST DCn score: 0.469
Fix modifications: C2: Carbamidomethyl (+57.02)Variable modifications: None
charge: 2 Observed m/z: 566.7841
Actual minus calculated peptide mass (AMU): -0.0070758

Peptide sequence: (K)NYSDFIVAAYCLK(H)
Exclusive (unique to this protein): FALSE
SEQUEST XCorr score: 2.44 SEQUEST DCn score: 0.653
Fix modifications: C11: Carbamidomethyl (+57.02)Variable modifications: None
charge: 2 Observed m/z: 782.3776
Actual minus calculated peptide mass (AMU): -0.004517

Peptide sequence: (K)VLNLASNNLNQGISSLCK(A)
Exclusive (unique to this protein): FALSE
SEQUEST XCorr score: 2.41 SEQUEST DCn score: 0.659
Fix modifications: C17: Carbamidomethyl (+57.02)Variable modifications: None
charge: 2 Observed m/z: 973.0115
Actual minus calculated peptide mass (AMU): -0.002441

Peptide sequence: (K)LLCDILNQAECNIEK(L)
Exclusive (unique to this protein): FALSE
SEQUEST XCorr score: 2.58 SEQUEST DCn score: 0.454
Fix modifications: C3: Carbamidomethyl (+57.02), C11: Carbamidomethyl (+57.02)Variable modifications: None
charge: 2 Observed m/z: 916.9451
Actual minus calculated peptide mass (AMU): -0.00647

Peptide sequence: (K)ASREDLANLLLK(C)
Exclusive (unique to this protein): FALSE
SEQUEST XCorr score: 2.07 SEQUEST DCn score: 0.177

Fix modifications: None
Variable modifications: None
charge: 2 Observed m/z: 671.8851
Actual minus calculated peptide mass (AMU): -0.007202

Peptide sequence: (K)MLPESSLLLSTTPETFEK(M)
Exclusive (unique to this protein): FALSE
SEQUEST XCorr score: 2.22 SEQUEST DCn score: 0.6
Fix modifications: None
Variable modifications: None
charge: 2 Observed m/z: 1012.0167
Actual minus calculated peptide mass (AMU): -0.005737

Peptide sequence: (K)QPVSTLLSSLLR(R)
Exclusive (unique to this protein): FALSE
SEQUEST XCorr score: 2 SEQUEST DCn score: 0.57
Fix modifications: None
Variable modifications: None
charge: 2 Observed m/z: 657.3895
Actual minus calculated peptide mass (AMU): -0.0081757

Peptide sequence: (K)LGVFFGHR(L)
Exclusive (unique to this protein): FALSE
SEQUEST XCorr score: 2.24 SEQUEST DCn score: 0.253
Fix modifications: None
Variable modifications: None
charge: 2 Observed m/z: 466.7558
Actual minus calculated peptide mass (AMU): -0.006836

Peptide sequence: (R)EWPSPSAPIEEILSQPEK(L)
Exclusive (unique to this protein): FALSE
SEQUEST XCorr score: 2.3 SEQUEST DCn score: 0.564
Fix modifications: None
Variable modifications: None
charge: 2 Observed m/z: 1019.0104
Actual minus calculated peptide mass (AMU): -0.005249

Peptide sequence: (R)NGIFDSDIPTLLDIGMLGK(I)
Exclusive (unique to this protein): FALSE

SEQUEST XCorr score: 2.72 SEQUEST DCn score: 0.55
Fix modifications: None
Variable modifications: M16: Oxidation (+16.00)
charge: 2 Observed m/z: 1018.0223
Actual minus calculated peptide mass (AMU): -0.005859

Peptide sequence: (K)TASLAELISR(E)
Exclusive (unique to this protein): FALSE
SEQUEST XCorr score: 2.87 SEQUEST DCn score: 0.534
Fix modifications: None
Variable modifications: None
charge: 2 Observed m/z: 530.8018
Actual minus calculated peptide mass (AMU): -0.004639

Peptide sequence: (R)AQEAFSLVR(E)
Exclusive (unique to this protein): FALSE
SEQUEST XCorr score: 2.13 SEQUEST DCn score: 0.375
Fix modifications: None
Variable modifications: None
charge: 2 Observed m/z: 510.775
Actual minus calculated peptide mass (AMU): -0.005554

Gene symbol: Ogdh
Protein name: Isoform 4 of 2-oxoglutarate dehydrogenase E1 component, mitochondrial precursor
Protein accession numbers: IPI00420882,IPI00626237,IPI00719841,IPI00845652
Peptide sequence: (R)NTNAGAPPGTAYQSPLSLSR(S)
Exclusive (unique to this protein): TRUE
SEQUEST XCorr score: 2.14 SEQUEST DCn score: 0.505
Fix modifications: None
Variable modifications: None
charge: 2 Observed m/z: 1001.5018
Actual minus calculated peptide mass (AMU): -0.003662

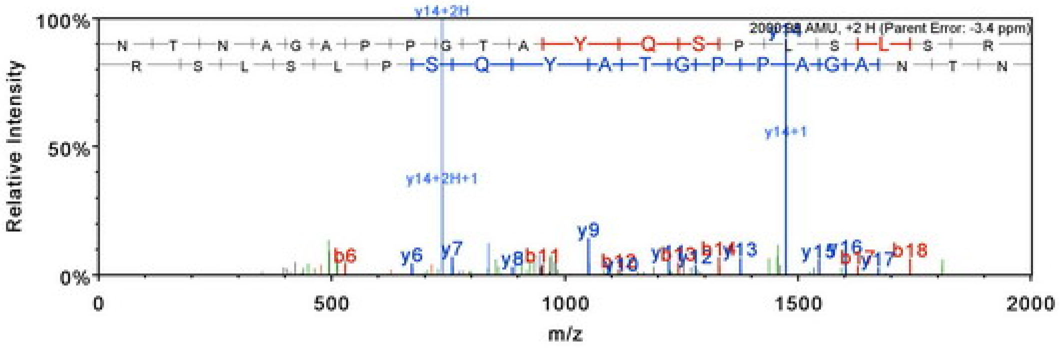

B	B Ions	B+2H	B-NH3	B-H2O	AA	Y Ions	Y+2H	Y-NH3	Y-H2O	Y
1	115.1		98.0		N	2002.0	1001.5	1985.0	1984.0	20
2	216.1		199.1	198.1	T	1888.0	944.5	1870.9	1870.0	19
3	330.1		313.1	312.1	N	1786.9	894.0	1769.9	1768.9	18
4	401.2		384.2	383.2	A	1672.9	836.9	1655.8	1654.9	17
5	458.2		441.2	440.2	G	1601.8	801.4	1584.8	1583.8	16
6	529.2	265.1	512.2	511.2	A	1544.8	772.9	1527.8	1526.8	15
7	626.3	313.7	609.3	608.3	P	1473.8	737.4	1456.7	1455.8	14
8	723.3	362.2	706.3	705.3	P	1376.7	688.9	1359.7	1358.7	13
9	780.4	390.7	763.3	762.4	G	1279.7	640.3	1262.6	1261.7	12
10	881.4	441.2	864.4	863.4	T	1222.6	611.8	1205.6	1204.6	11
11	952.5	476.7	935.4	934.4	A	1121.6	561.3	1104.6	1103.6	10
12	1115.5	558.3	1098.5	1097.5	Y	1050.6	525.8	1033.5	1032.5	9
13	1243.6	622.3	1226.5	1225.6	Q	887.5	444.3	870.5	869.5	8
14	1330.6	665.8	1313.6	1312.6	S	759.4	380.2	742.4	741.4	7
15	1427.7	714.3	1410.6	1409.7	P	672.4	336.7	655.4	654.4	6
16	1540.7	770.9	1523.7	1522.7	L	575.4		558.3	557.3	5
17	1627.8	814.4	1610.8	1609.8	S	462.3		445.2	444.3	4
18	1740.9	870.9	1723.8	1722.9	L	375.2		358.2	357.2	3
19	1827.9	914.5	1810.9	1809.9	S	262.1		245.1	244.1	2
20	2002.0	1001.5	1985.0	1984.0	R	175.1		158.1		1

Gene symbol: Omt2b

Protein name: oocyte maturation, beta

Protein accession numbers: IPI00127073,IPI00624133,IPI00753313

Peptide sequence: (R)GLQSPVWWNR(R)

Exclusive (unique to this protein): TRUE

SEQUEST XCorr score: 2.34 SEQUEST DCn score: 0.675

Fix modifications: None

Variable modifications: None

charge: 2 Observed m/z: 621.819

Actual minus calculated peptide mass (AMU): -0.008057

Gene symbol: ORF34

Protein name: similar to Protein CXorf17 homolog isoform 2
Protein accession numbers: IPI00750786
Peptide sequence: (K)NLTTNQFLMQEVAKQLGLK(R)
Exclusive (unique to this protein): TRUE
SEQUEST XCorr score: 2.02 SEQUEST DCn score: 0.335
Fix modifications: None
Variable modifications: None
charge: 2 Observed m/z: 1088.5839
Actual minus calculated peptide mass (AMU): -0.020254

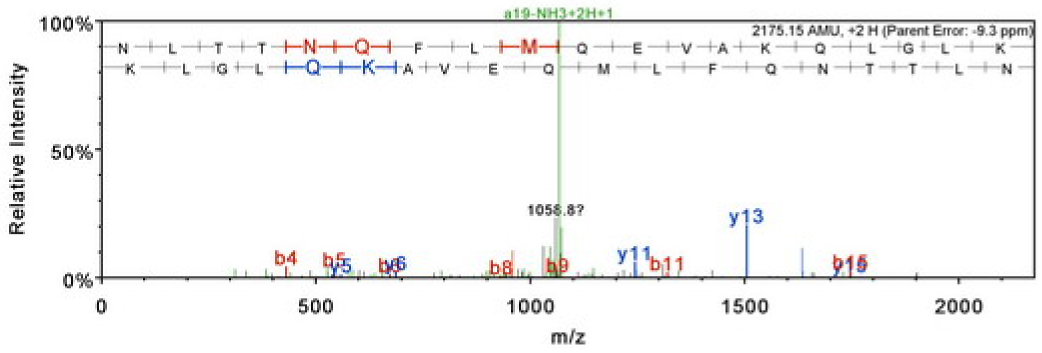

B	B Ions	B+2H	B-NH3	B-H2O	AA	Y Ions	Y+2H	Y-NH3	Y-H2O	Y
1	115.1		98.0		N	2176.2	1088.6	2159.2	2158.2	19
2	228.1		211.1		L	2062.1	1031.6	2045.1	2044.1	18
3	329.2		312.2	311.2	T	1949.1	975.0	1932.0	1931.0	17
4	430.2		413.2	412.2	T	1848.0	924.5	1831.0	1830.0	16
5	544.3		527.3	526.3	N	1747.0	874.0	1729.9	1729.0	15
6	672.3	336.7	655.3	654.3	Q	1632.9	817.0	1615.9	1614.9	14
7	819.4	410.2	802.4	801.4	F	1504.9	752.9	1487.8	1486.9	13
8	932.5	466.8	915.5	914.5	L	1357.8	679.4	1340.8	1339.8	12
9	1063.5	532.3	1046.5	1045.5	M	1244.7	622.9	1227.7	1226.7	11
10	1191.6	596.3	1174.6	1173.6	Q	1113.7	557.3	1096.6	1095.7	10
11	1320.6	660.8	1303.6	1302.6	E	985.6	493.3	968.6	967.6	9
12	1419.7	710.4	1402.7	1401.7	V	856.6	428.8	839.5		8
13	1490.7	745.9	1473.7	1472.7	A	757.5	379.3	740.5		7
14	1618.8	809.9	1601.8	1600.8	K	686.5	343.7	669.4		6
15	1746.9	874.0	1729.9	1728.9	Q	558.4		541.3		5
16	1860.0	930.5	1842.9	1842.0	L	430.3		413.3		4
17	1917.0	959.0	1900.0	1899.0	G	317.2		300.2		3
18	2030.1	1015.5	2013.1	2012.1	L	260.2		243.2		2
19	2176.2	1088.6	2159.2	2158.2	K	147.1		130.1		1

Gene symbol: Osbp
Protein name: oxysterol binding protein
Protein accession numbers: IPI00755161
Peptide sequence: (R)GVVGPGPAAIAAPGGGGAGPPAVGGGGGR(G)
Exclusive (unique to this protein): TRUE
SEQUEST XCorr score: 3.01 SEQUEST DCn score: 0.726
Fix modifications: None

Variable modifications: None

charge: 2 Observed m/z: 1119.5885

Actual minus calculated peptide mass (AMU): -0.0056153

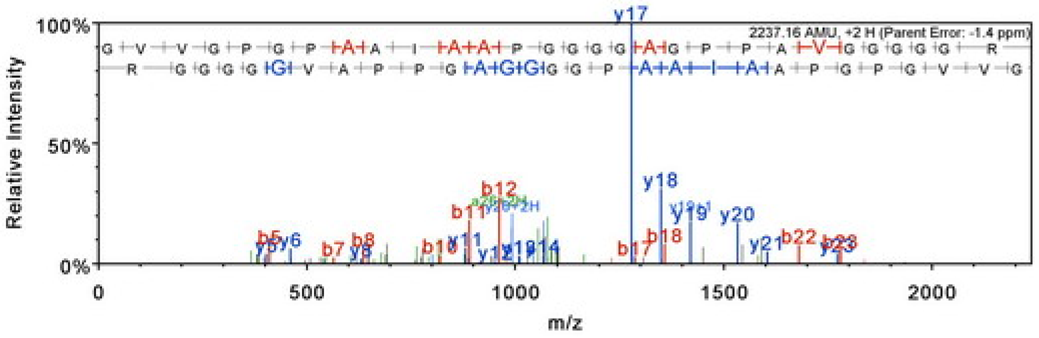

B	B Ions	B+2H	B-NH3	B-H2O	AA	Y Ions	Y+2H	Y-NH3	Y-H2O	Y
1	58.0				G	2238.2	1119.6	2221.2		29
2	157.1				V	2181.2	1091.1	2164.1		28
3	256.2				V	2082.1	1041.5	2065.1		27
4	313.2				G	1983.0	992.0	1966.0		26
5	410.2				P	1926.0	963.5	1909.0		25
6	467.3	234.1			G	1828.9	915.0	1811.9		24
7	564.3	282.7			P	1771.9	886.5	1754.9		23
8	635.4	318.2			A	1674.9	837.9	1657.8		22
9	706.4	353.7			A	1603.8	802.4	1586.8		21
10	819.5	410.2			I	1532.8	766.9	1515.8		20
11	890.5	445.8			A	1419.7	710.4	1402.7		19
12	961.6	481.3			A	1348.7	674.8	1331.7		18
13	1058.6	529.8			P	1277.6	639.3	1260.6		17
14	1115.6	558.3			G	1180.6	590.8	1163.6		16
15	1172.6	586.8			G	1123.6	562.3	1106.5		15
16	1229.7	615.3			G	1066.5	533.8	1049.5		14
17	1286.7	643.9			G	1009.5	505.3	992.5		13
18	1357.7	679.4			A	952.5	476.8	935.5		12
19	1414.7	707.9			G	881.5	441.2	864.4		11
20	1511.8	756.4			P	824.4	412.7	807.4		10
21	1608.9	804.9			P	727.4	364.2	710.4		9
22	1679.9	840.5			A	630.3	315.7	613.3		8
23	1779.0	890.0			V	559.3	280.2	542.3		7
24	1836.0	918.5			G	460.2	230.6	443.2		6
25	1893.0	947.0			G	403.2		386.2		5
26	1950.0	975.5			G	346.2		329.2		4
27	2007.0	1004.0			G	289.2		272.1		3
28	2064.1	1032.5			G	232.1		215.1		2
29	2238.2	1119.6	2221.2		R	175.1		158.1		1

Gene symbol: OTTMUSG00000001305

Protein name: CDNA sequence BC099439

Protein accession numbers: IPI00346337

Peptide sequence: (R)ECGFTSQVARPEAPATVGLAFR(T)

Exclusive (unique to this protein): TRUE

SEQUEST XCorr score: 2.49 SEQUEST DCn score: 0.507

Fix modifications: C2: Carbamidomethyl (+57.02)Variable modifications: None

charge: 2 Observed m/z: 1182.5895

Actual minus calculated peptide mass (AMU): -0.006104

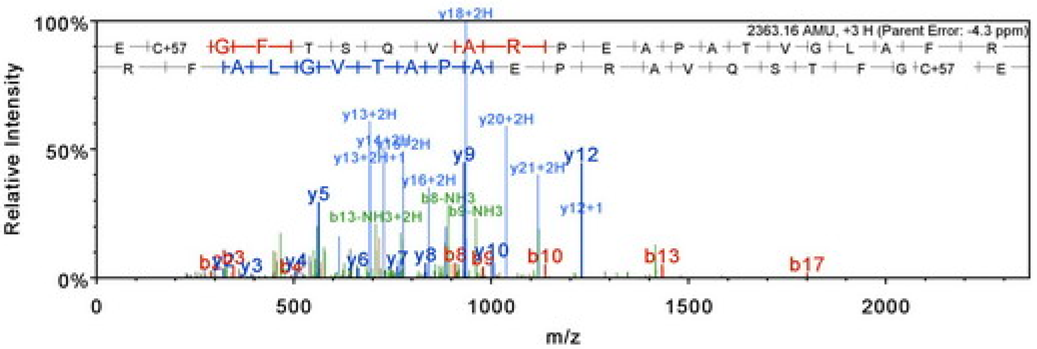

B	B Ions	B+2H	B-NH3	B-H2O	AA	Y Ions	Y+2H	Y-NH3	Y-H2O	Y
1	130.1			112.0	E	2364.2	1182.6	2347.2	2346.2	22
2	290.1			272.1	C+57	2235.1	1118.1	2218.1	2217.1	21
3	347.1			329.1	G	2075.1	1038.1	2058.1	2057.1	20
4	494.2			476.2	F	2018.1	1009.6	2001.1	2000.1	19
5	595.2			577.2	T	1871.0	936.0	1854.0	1853.0	18
6	682.3	341.6		664.2	S	1770.0	885.5	1752.9	1752.0	17
7	810.3	405.7	793.3	792.3	Q	1682.9	842.0	1665.9	1664.9	16
8	909.4	455.2	892.4	891.4	V	1554.9	777.9	1537.9	1536.9	15
9	980.4	490.7	963.4	962.4	A	1455.8	728.4	1438.8	1437.8	14
10	1136.5	568.8	1119.5	1118.5	R	1384.8	692.9	1367.7	1366.8	13
11	1233.6	617.3	1216.5	1215.6	P	1228.7	614.8	1211.6	1210.7	12
12	1362.6	681.8	1345.6	1344.6	E	1131.6	566.3	1114.6	1113.6	11
13	1433.7	717.3	1416.6	1415.6	A	1002.6	501.8	985.6	984.6	10
14	1530.7	765.9	1513.7	1512.7	P	931.5	466.3	914.5	913.5	9
15	1601.7	801.4	1584.7	1583.7	A	834.5	417.8	817.5	816.5	8
16	1702.8	851.9	1685.8	1684.8	T	763.5	382.2	746.4	745.4	7
17	1801.9	901.4	1784.8	1783.8	V	662.4	331.7	645.4		6
18	1858.9	929.9	1841.9	1840.9	G	563.3		546.3		5
19	1972.0	986.5	1954.9	1954.0	L	506.3		489.3		4
20	2043.0	1022.0	2026.0	2025.0	A	393.2		376.2		3
21	2190.1	1095.5	2173.0	2172.1	F	322.2		305.2		2
22	2364.2	1182.6	2347.2	2346.2	R	175.1		158.1		1

Gene symbol: P4hb

Protein name: 17 days embryo kidney cDNA, RIKEN full-length enriched library, clone:I920160L24 product:prolyl 4-hydroxylase, beta polypeptide, full insert sequence

Protein accession numbers: IPI00122815,IPI00133522

Peptide sequence: (K)VDATEESDLAQQYGVR(G)

Exclusive (unique to this protein): TRUE

SEQUEST XCorr score: 2.78 SEQUEST DCn score: 0.649

Fix modifications: None

Variable modifications: None

charge: 2 Observed m/z: 890.92

Actual minus calculated peptide mass (AMU): -0.0031752

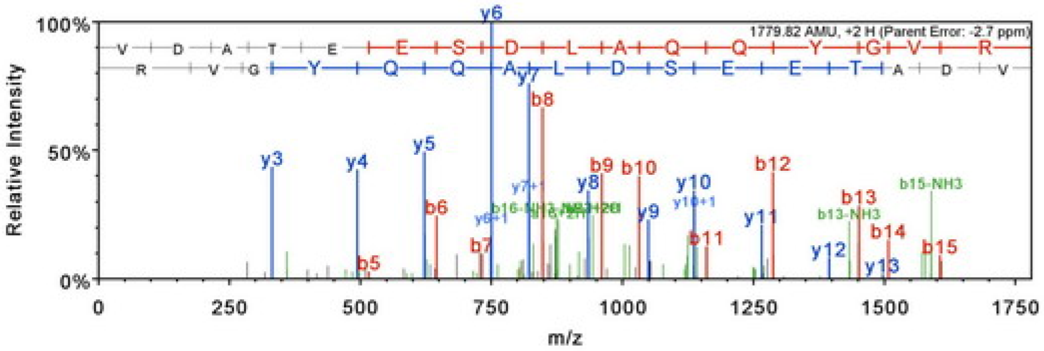

B	B Ions	B+2H	B-NH3	B-H2O	AA	Y Ions	Y+2H	Y-NH3	Y-H2O	Y
1	100.1				V	1780.8	890.9	1763.8	1762.8	16
2	215.1			197.1	D	1681.8	841.4	1664.7	1663.8	15
3	286.1			268.1	A	1566.7	783.9	1549.7	1548.7	14
4	387.2			369.2	T	1495.7	748.4	1478.7	1477.7	13
5	516.2			498.2	E	1394.7	697.8	1377.6	1376.6	12
6	645.3	323.1		627.3	E	1265.6	633.3	1248.6	1247.6	11
7	732.3	366.7		714.3	S	1136.6	568.8	1119.5	1118.6	10
8	847.3	424.2		829.3	D	1049.5	525.3	1032.5	1031.5	9
9	960.4	480.7		942.4	L	934.5	467.8	917.5		8
10	1031.5	516.2		1013.4	A	821.4	411.2	804.4		7
11	1159.5	580.3	1142.5	1141.5	Q	750.4	375.7	733.4		6
12	1287.6	644.3	1270.5	1269.6	Q	622.3		605.3		5
13	1450.6	725.8	1433.6	1432.6	Y	494.3		477.3		4
14	1507.7	754.3	1490.6	1489.6	G	331.2		314.2		3
15	1606.7	803.9	1589.7	1588.7	V	274.2		257.2		2
16	1780.8	890.9	1763.8	1762.8	R	175.1		158.1		1

Gene symbol: Pafah1b3

Protein name: Platelet-activating factor acetylhydrolase IB subunit gamma

Protein accession numbers: IPI00118819

Peptide sequence: (R)VVVLGLLPR(G)

Exclusive (unique to this protein): TRUE

SEQUEST XCorr score: 2.62 SEQUEST DCn score: 0.768

Fix modifications: None

Variable modifications: None

charge: 2 Observed m/z: 483.3263

Actual minus calculated peptide mass (AMU): -0.0065349

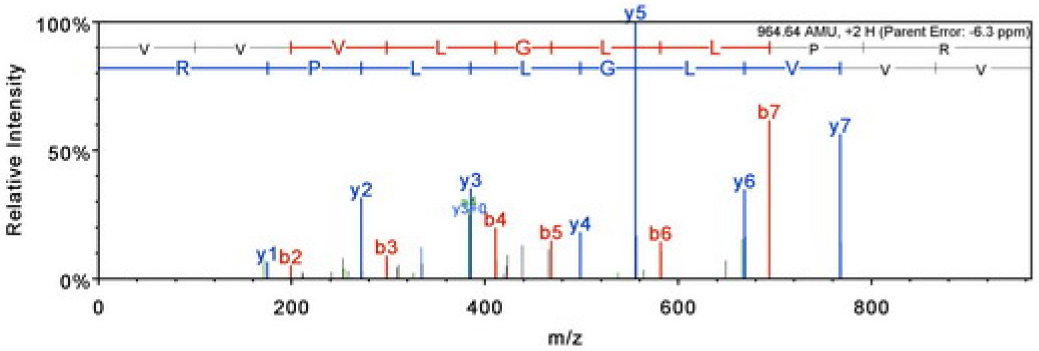

B	B Ions	B+2H	B-NH3	B-H2O	AA	Y Ions	Y+2H	Y-NH3	Y-H2O	Y
1	100.1				V	965.7	483.3	948.6		9
2	199.1				V	866.6	433.8	849.6		8
3	298.2				V	767.5	384.3	750.5		7
4	411.3				L	668.5	334.7	651.4		6
5	468.3				G	555.4		538.3		5
6	581.4	291.2			L	498.3		481.3		4
7	694.5	347.8			L	385.3		368.2		3
8	791.5	396.3			P	272.2		255.2		2
9	965.7	483.3	948.6		R	175.1		158.1		1

Gene symbol: Pcbp1

Protein name: Poly(rC)-binding protein 1

Protein accession numbers: IPI00128904

Peptide sequence: (R)IITLTGPTNAIFK(A)

Exclusive (unique to this protein): TRUE

SEQUEST XCorr score: 2.26 SEQUEST DCn score: 0.669

Fix modifications: None

Variable modifications: None

charge: 2 Observed m/z: 694.9084

Actual minus calculated peptide mass (AMU): -0.00647

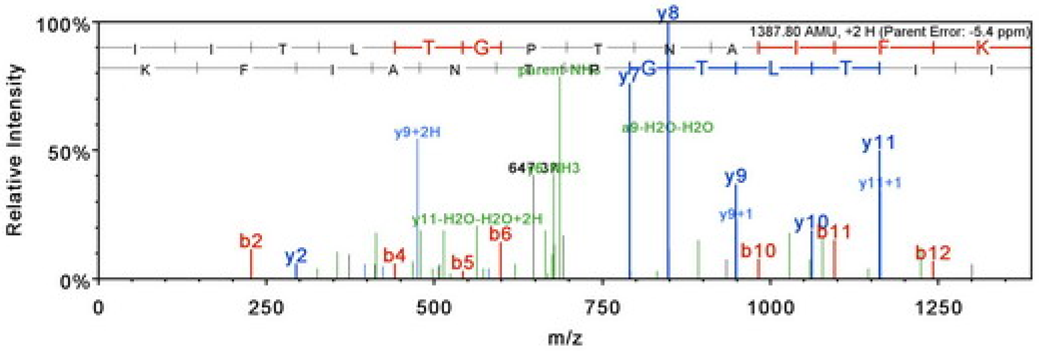

B	B Ions	B+2H	B-NH3	B-H2O	AA	Y Ions	Y+2H	Y-NH3	Y-H2O	Y
1	114.1				I	1388.8	694.9	1371.8	1370.8	13
2	227.2				I	1275.7	638.4	1258.7	1257.7	12
3	328.2			310.2	T	1162.7	581.8	1145.6	1144.6	11
4	441.3			423.3	L	1061.6	531.3	1044.6	1043.6	10
5	542.4			524.3	T	948.5	474.8	931.5	930.5	9
6	599.4	300.2		581.4	G	847.5	424.2	830.4	829.5	8
7	696.4	348.7		678.4	P	790.5	395.7	773.4	772.4	7
8	797.5	399.2		779.5	T	693.4	347.2	676.4	675.4	6
9	911.5	456.3	894.5	893.5	N	592.4		575.3		5
10	982.6	491.8	965.5	964.6	A	478.3		461.3		4
11	1095.6	548.3	1078.6	1077.6	I	407.3		390.2		3
12	1242.7	621.9	1225.7	1224.7	F	294.2		277.2		2
13	1388.8	694.9	1371.8	1370.8	K	147.1		130.1		1

Gene symbol: Pcdhga2

Protein name: Protocadherin gamma A2

Protein accession numbers: IPI00229636

Peptide sequence: (K)YKLNSNDHFSLDVRTGADGNK(Y)

Exclusive (unique to this protein): TRUE

SEQUEST XCorr score: 2.55 SEQUEST DCn score: 0.366

Fix modifications: None

Variable modifications: None

charge: 3 Observed m/z: 784.3781

Actual minus calculated peptide mass (AMU): -0.019257

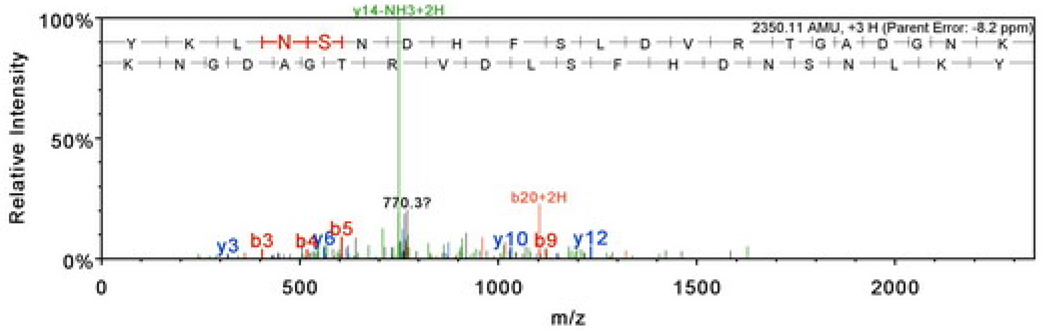

B	B Ions	B+2H	B-NH3	B-H2O	AA	Y Ions	Y+2H	Y-NH3	Y-H2O	Y
1	164.1				Y	2351.1	1176.1	2334.1	2333.1	21
2	292.2	146.6	275.1		K	2188.1	1094.5	2171.1	2170.1	20
3	405.3	203.1	388.2		L	2060.0	1030.5	2043.0	2042.0	19
4	519.3	260.1	502.3		N	1946.9	974.0	1929.9	1928.9	18
5	606.3	303.7	589.3	588.3	S	1832.9	916.9	1815.8	1814.8	17
6	720.4	360.7	703.3	702.4	N	1745.8	873.4	1728.8	1727.8	16
7	835.4	418.2	818.4	817.4	D	1631.8	816.4	1614.8	1613.8	15
8	972.5	486.7	955.4	954.4	H	1516.8	758.9	1499.7	1498.7	14
9	1119.5	560.3	1102.5	1101.5	F	1379.7	690.4	1362.7	1361.7	13
10	1206.5	603.8	1189.5	1188.5	S	1232.6	616.8	1215.6	1214.6	12
11	1319.6	660.3	1302.6	1301.6	L	1145.6	573.3	1128.6	1127.6	11
12	1434.7	717.8	1417.6	1416.7	D	1032.5	516.8	1015.5	1014.5	10
13	1533.7	767.4	1516.7	1515.7	V	917.5	459.2	900.5	899.5	9
14	1689.8	845.4	1672.8	1671.8	R	818.4	409.7	801.4	800.4	8
15	1790.9	896.0	1773.9	1772.9	T	662.3	331.7	645.3	644.3	7
16	1847.9	924.5	1830.9	1829.9	G	561.3	281.1	544.2	543.3	6
17	1918.9	960.0	1901.9	1900.9	A	504.2		487.2	486.2	5
18	2034.0	1017.5	2016.9	2016.0	D	433.2		416.2	415.2	4
19	2091.0	1046.0	2074.0	2073.0	G	318.2		301.2		3
20	2205.0	1103.0	2188.0	2187.0	N	261.2		244.1		2
21	2351.1	1176.1	2334.1	2333.1	K	147.1		130.1		1

Gene symbol: Pcmt1

Protein name: Isoform 1 of Protein-L-isoaspartate(D-aspartate) O-methyltransferase

Protein accession numbers: IPI00329913,IPI00480558,IPI00757109

Peptide sequence: (K)ALDVGSGSGILTACFAR(M)

Exclusive (unique to this protein): TRUE

SEQUEST XCorr score: 2.94 SEQUEST DCn score: 0.722

Fix modifications: C14: Carbamidomethyl (+57.02)Variable modifications: None

charge: 2 Observed m/z: 847.927

Actual minus calculated peptide mass (AMU): -0.0074454

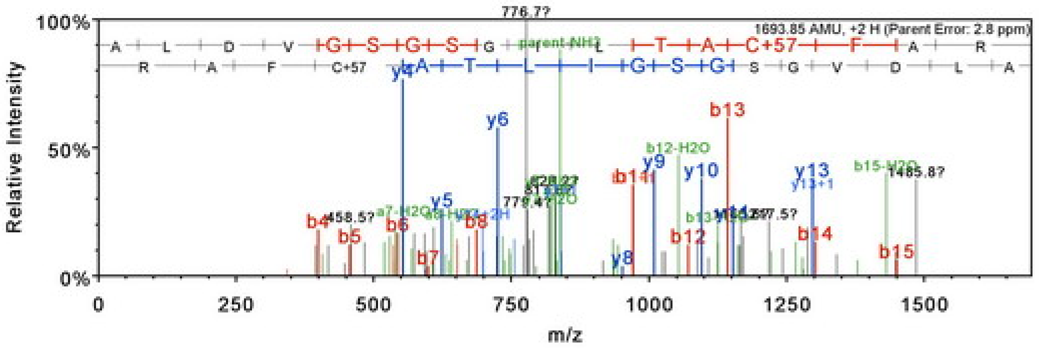

B	B Ions	B+2H	B-NH3	B-H2O	AA	Y Ions	Y+2H	Y-NH3	Y-H2O	Y
1	72.0				A	1694.9	847.9	1677.8	1676.8	17
2	185.1				L	1623.8	812.4	1606.8	1605.8	16
3	300.2			282.2	D	1510.7	755.9	1493.7	1492.7	15
4	399.2			381.2	V	1395.7	698.4	1378.7	1377.7	14
5	456.3			438.2	G	1296.6	648.8	1279.6	1278.6	13
6	543.3	272.1		525.3	S	1239.6	620.3	1222.6	1221.6	12
7	600.3	300.7		582.3	G	1152.6	576.8	1135.6	1134.6	11
8	687.3	344.2		669.3	S	1095.6	548.3	1078.5	1077.5	10
9	744.4	372.7		726.3	G	1008.5	504.8	991.5	990.5	9
10	857.4	429.2		839.4	I	951.5	476.3	934.5	933.5	8
11	970.5	485.8		952.5	L	838.4	419.7	821.4	820.4	7
12	1071.6	536.3		1053.6	T	725.3	363.2	708.3	707.3	6
13	1142.6	571.8		1124.6	A	624.3		607.3		5
14	1302.6	651.8		1284.6	C+57	553.3		536.2		4
15	1449.7	725.4		1431.7	F	393.2		376.2		3
16	1520.7	760.9		1502.7	A	246.2		229.1		2
17	1694.9	847.9	1677.8	1676.8	R	175.1		158.1		1

Gene symbol: Pcx

Protein name: Activated spleen cDNA, RIKEN full-length enriched library, clone:F830201B12 product:pyruvate carboxylase, full insert sequence

Protein accession numbers: IPI00114710

Peptide sequence: (R)AEAEAQAEELSFPR(S)

Exclusive (unique to this protein): TRUE

SEQUEST XCorr score: 2.64 SEQUEST DCn score: 0.701

Fix modifications: None

Variable modifications: None

charge: 2 Observed m/z: 774.3681

Actual minus calculated peptide mass (AMU): -0.005859

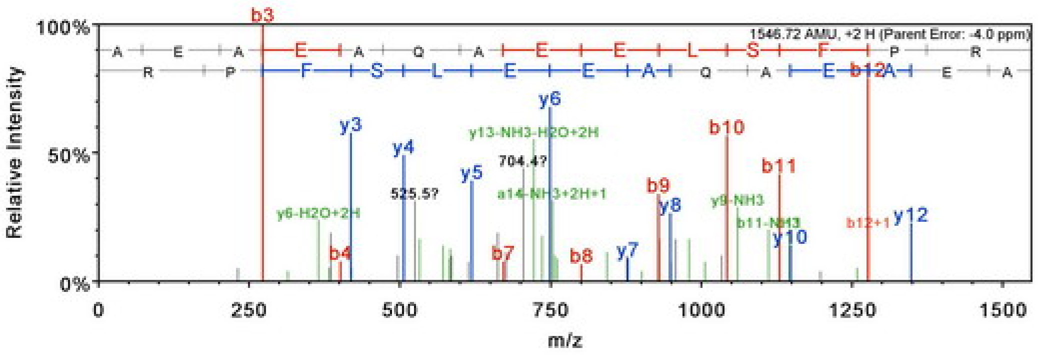

B	B Ions	B+2H	B-NH3	B-H2O	AA	Y Ions	Y+2H	Y-NH3	Y-H2O	Y
1	72.0				A	1547.7	774.4	1530.7	1529.7	14
2	201.1			183.1	E	1476.7	738.9	1459.7	1458.7	13
3	272.1			254.1	A	1347.7	674.3	1330.6	1329.6	12
4	401.2			383.2	E	1276.6	638.8	1259.6	1258.6	11
5	472.2			454.2	A	1147.6	574.3	1130.5	1129.6	10
6	600.3	300.6	583.2	582.3	Q	1076.5	538.8	1059.5	1058.5	9
7	671.3	336.2	654.3	653.3	A	948.5	474.7	931.5	930.5	8
8	800.3	400.7	783.3	782.3	E	877.4	439.2	860.4	859.4	7
9	929.4	465.2	912.4	911.4	E	748.4	374.7	731.4	730.4	6
10	1042.5	521.7	1025.4	1024.5	L	619.4		602.3	601.4	5
11	1129.5	565.3	1112.5	1111.5	S	506.3		489.3	488.3	4
12	1276.6	638.8	1259.5	1258.6	F	419.2		402.2		3
13	1373.6	687.3	1356.6	1355.6	P	272.2		255.2		2
14	1547.7	774.4	1530.7	1529.7	R	175.1		158.1		1

Gene symbol: Pdcd5

Protein name: Programmed cell death protein 5

Protein accession numbers: IPI00116120

Peptide sequence: (R)NSILAQVLDQSAR(A)

Exclusive (unique to this protein): TRUE

SEQUEST XCorr score: 2.51 SEQUEST DCn score: 0.371

Fix modifications: None

Variable modifications: None

charge: 2 Observed m/z: 707.8834

Actual minus calculated peptide mass (AMU): -0.006592

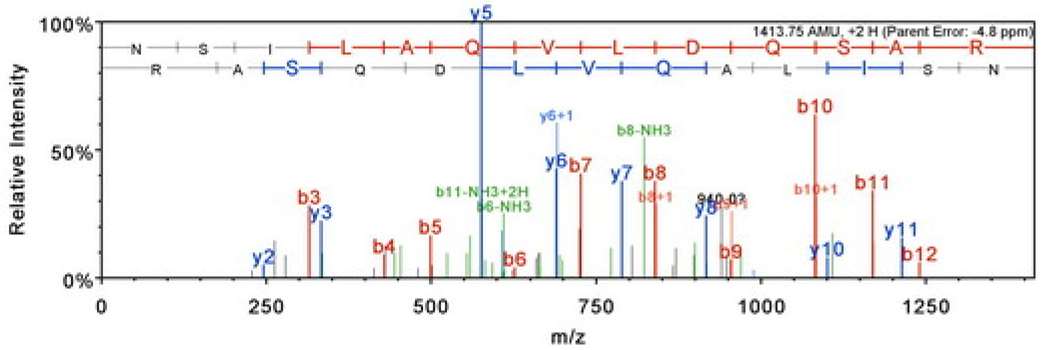

B	B Ions	B+2H	B-NH3	B-H2O	AA	Y Ions	Y+2H	Y-NH3	Y-H2O	Y
1	115.1		98.0		N	1414.8	707.9	1397.7	1396.8	13
2	202.1		185.1	184.1	S	1300.7	650.9	1283.7	1282.7	12
3	315.2		298.1	297.2	I	1213.7	607.4	1196.7	1195.7	11
4	428.3		411.2	410.2	L	1100.6	550.8	1083.6	1082.6	10
5	499.3		482.3	481.3	A	987.5	494.3	970.5	969.5	9
6	627.4	314.2	610.3	609.3	Q	916.5	458.8	899.5	898.5	8
7	726.4	363.7	709.4	708.4	V	788.4	394.7	771.4	770.4	7
8	839.5	420.3	822.5	821.5	L	689.4	345.2	672.3	671.4	6
9	954.5	477.8	937.5	936.5	D	576.3		559.3	558.3	5
10	1082.6	541.8	1065.6	1064.6	Q	461.3		444.2	443.2	4
11	1169.6	585.3	1152.6	1151.6	S	333.2		316.2	315.2	3
12	1240.7	620.8	1223.6	1222.6	A	246.2		229.1		2
13	1414.8	707.9	1397.7	1396.8	R	175.1		158.1		1

Gene symbol: Pdha1

Protein name: Pyruvate dehydrogenase E1 component alpha subunit, somatic form, mitochondrial precursor

Protein accession numbers: IPI00337893

Peptide sequence: (R)LEEGPPVTTVLTR(E)

Exclusive (unique to this protein): TRUE

SEQUEST XCorr score: 2.22 SEQUEST DCn score: 0.607

Fix modifications: None

Variable modifications: None

charge: 2 Observed m/z: 706.3906

Actual minus calculated peptide mass (AMU): -0.006592

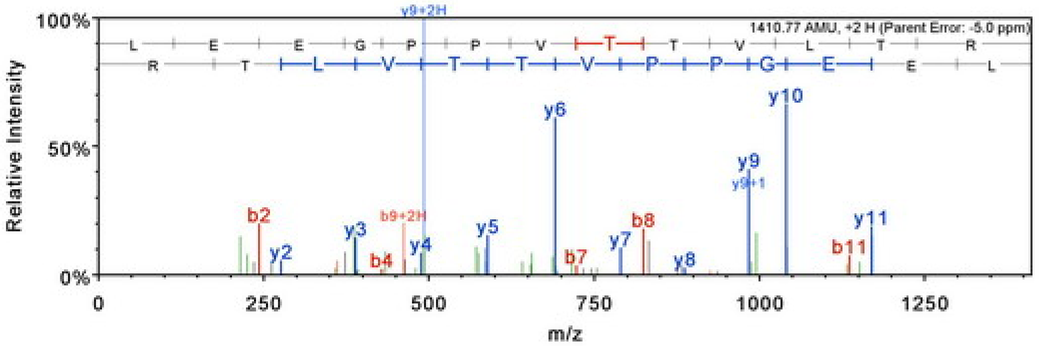

B	B Ions	B+2H	B-NH3	B-H2O	AA	Y Ions	Y+2H	Y-NH3	Y-H2O	Y
1	114.1				L	1411.8	706.4	1394.8	1393.8	13
2	243.1			225.1	E	1298.7	649.9	1281.7	1280.7	12
3	372.2			354.2	E	1169.7	585.3	1152.6	1151.6	11
4	429.2			411.2	G	1040.6	520.8	1023.6	1022.6	10
5	526.3			508.2	P	983.6	492.3	966.6	965.6	9
6	623.3	312.2		605.3	P	886.5	443.8	869.5	868.5	8
7	722.4	361.7		704.4	V	789.5	395.3	772.5	771.5	7
8	823.4	412.2		805.4	T	690.4	345.7	673.4	672.4	6
9	924.5	462.7		906.5	T	589.4		572.3	571.4	5
10	1023.5	512.3		1005.5	V	488.3		471.3	470.3	4
11	1136.6	568.8		1118.6	L	389.3		372.2	371.2	3
12	1237.7	619.3		1219.7	T	276.2		259.1	258.2	2
13	1411.8	706.4	1394.8	1393.8	R	175.1		158.1		1

Gene symbol: Pfdn2

Protein name: Prefoldin subunit 2

Protein accession numbers: IPI00119545

Peptide sequence: (K)GAVSAEQVIAGFNR(L)

Exclusive (unique to this protein): TRUE

SEQUEST XCorr score: 2.07 SEQUEST DCn score: 0.295

Fix modifications: None

Variable modifications: None

charge: 2 Observed m/z: 709.8745

Actual minus calculated peptide mass (AMU): 0.002075

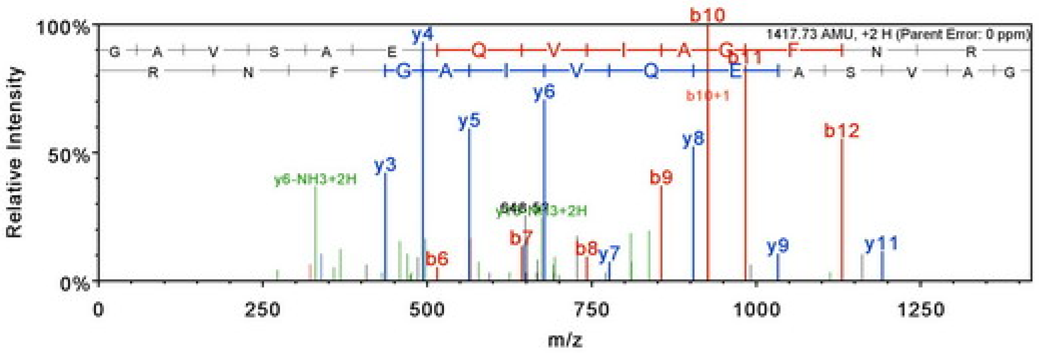

B	B Ions	B+2H	B-NH3	B-H2O	AA	Y Ions	Y+2H	Y-NH3	Y-H2O	Y
1	58.0				G	1418.7	709.9	1401.7	1400.7	14
2	129.1				A	1361.7	681.4	1344.7	1343.7	13
3	228.1				V	1290.7	645.8	1273.7	1272.7	12
4	315.2			297.2	S	1191.6	596.3	1174.6	1173.6	11
5	386.2			368.2	A	1104.6	552.8	1087.5	1086.6	10
6	515.3	258.1		497.2	E	1033.5	517.3	1016.5	1015.5	9
7	643.3	322.2	626.3	625.3	Q	904.5	452.8	887.5		8
8	742.4	371.7	725.4	724.4	V	776.4	388.7	759.4		7
9	855.5	428.2	838.4	837.5	I	677.4	339.2	660.4		6
10	926.5	463.8	909.5	908.5	A	564.3		547.3		5
11	983.5	492.3	966.5	965.5	G	493.3		476.2		4
12	1130.6	565.8	1113.6	1112.6	F	436.2		419.2		3
13	1244.6	622.8	1227.6	1226.6	N	289.2		272.1		2
14	1418.7	709.9	1401.7	1400.7	R	175.1		158.1		1
