# Supplementary material for: Proteomic-based identification of maternal proteins in mature mouse oocytes
Source: BMC Genomics. 2009 Aug 3;10:348. doi: 10.1186/1471-2164-10-348 (PMC2730056; doi:10.1186/1471-2164-10-348)
Supplement: Additional file 4 — Data from single peptide-based identification of oocyte proteins. Contains MS/MS spectra and fragment tables of single peptide-based identifications. [file 1471-2164-10-348-S4.pdf]

Gene symbol: Pfn2
Protein name: Isoform 2 of Profilin-2
Protein accession numbers: IPI00227805,IPI00228645,IPI00845675
Peptide sequence: (R)EGFFTNGLTLGAK(K)
Exclusive (unique to this protein): TRUE
SEQUEST XCorr score: 2.16   SEQUEST DCn score: 0.626
Fix modifications: None
Variable modifications: None
charge: 2   Observed m/z: 677.8497
Actual minus calculated peptide mass (AMU): -0.0092755

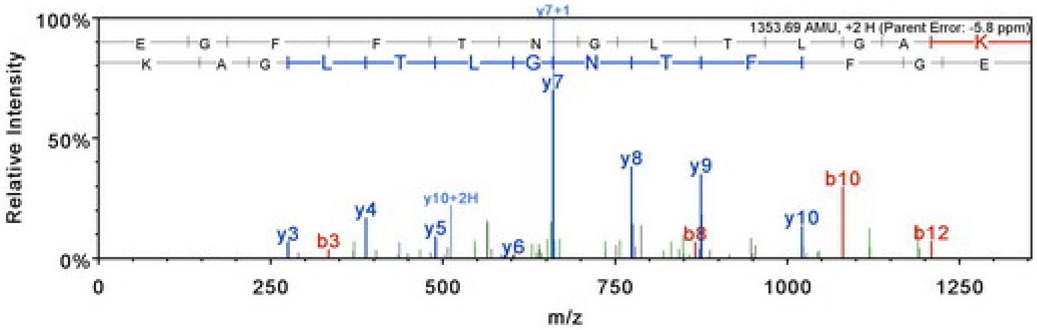

B	B Ions	B+2H	B-NH3	B-H2O	AA	Y Ions	Y+2H	Y-NH3	Y-H2O	Y
1	130.1			112.0	E	1354.7	677.9	1337.7	1336.7	13
2	187.1			169.1	G	1225.7	613.3	1208.6	1207.7	12
3	334.1			316.1	F	1168.6	584.8	1151.6	1150.6	11
4	481.2			463.2	F	1021.6	511.3	1004.5	1003.6	10
5	582.3			564.3	T	874.5	437.8	857.5	856.5	9
6	696.3	348.7	679.3	678.3	N	773.5	387.2	756.4	755.4	8
7	753.3	377.2	736.3	735.3	G	659.4	330.2	642.4	641.4	7
8	866.4	433.7	849.4	848.4	L	602.4	301.7	585.4	584.4	6
9	967.5	484.2	950.4	949.4	T	489.3		472.3	471.3	5
10	1080.5	540.8	1063.5	1062.5	L	388.3		371.2		4
11	1137.6	569.3	1120.5	1119.5	G	275.2		258.1		3
12	1208.6	604.8	1191.6	1190.6	A	218.2		201.1		2
13	1354.7	677.9	1337.7	1336.7	K	147.1		130.1		1

Gene symbol: Pgam5
Protein name: Isoform 2 of Phosphoglycerate mutase family member 5 precursor
Protein accession numbers: IPI00226387,IPI00461730,IPI00848970
Peptide sequence: (R)QEEDSYEIFICHANVIR(Y)
Exclusive (unique to this protein): TRUE
SEQUEST XCorr score: 2.24   SEQUEST DCn score: 0.644
Fix modifications: C11: Carbamidomethyl (+57.02)Variable modifications: None
charge: 2   Observed m/z: 1061.9933
Actual minus calculated peptide mass (AMU): -0.007813

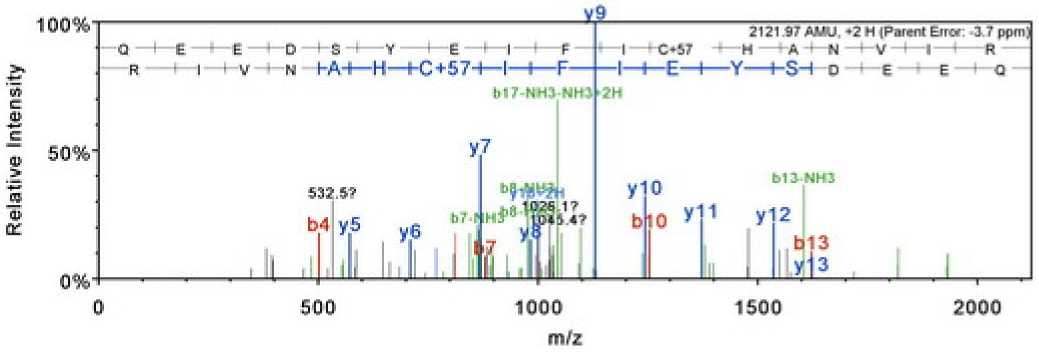

B	B Ions	B+2H	B-NH3	B-H2O	AA	Y Ions	Y+2H	Y-NH3	Y-H2O	Y
1	129.1		112.0		Q	2123.0	1062.0	2106.0	2105.0	17
2	258.1		241.1	240.1	E	1994.9	998.0	1977.9	1976.9	16
3	387.2		370.1	369.1	E	1865.9	933.5	1848.9	1847.9	15
4	502.2		485.2	484.2	D	1736.8	868.9	1719.8	1718.8	14
5	589.2		572.2	571.2	S	1621.8	811.4	1604.8	1603.8	13
6	752.3	376.6	735.3	734.3	Y	1534.8	767.9	1517.8	1516.8	12
7	881.3	441.2	864.3	863.3	E	1371.7	686.4	1354.7	1353.7	11
8	994.4	497.7	977.4	976.4	I	1242.7	621.8	1225.7		10
9	1141.5	571.2	1124.4	1123.5	F	1129.6	565.3	1112.6		9
10	1254.5	627.8	1237.5	1236.5	I	982.5	491.8	965.5		8
11	1414.6	707.8	1397.6	1396.6	C+57	869.4	435.2	852.4		7
12	1551.6	776.3	1534.6	1533.6	H	709.4	355.2	692.4		6
13	1622.7	811.8	1605.7	1604.7	A	572.4		555.3		5
14	1736.7	868.9	1719.7	1718.7	N	501.3		484.3		4
15	1835.8	918.4	1818.8	1817.8	V	387.3		370.3		3
16	1948.9	974.9	1931.9	1930.9	I	288.2		271.2		2
17	2123.0	1062.0	2106.0	2105.0	R	175.1		158.1		1

Gene symbol: Pgk1;EG433594;EG668435

Protein name: Phosphoglycerate kinase 1

Protein accession numbers: IPI00555069

Peptide sequence: (K)LGDVYVNDAFGTAHR(A)

Exclusive (unique to this protein): TRUE

SEQUEST XCorr score: 2.4 SEQUEST DCn score: 0.741

Fix modifications: None

Variable modifications: None

charge: 2 Observed m/z: 817.8967

Actual minus calculated peptide mass (AMU): -0.007202

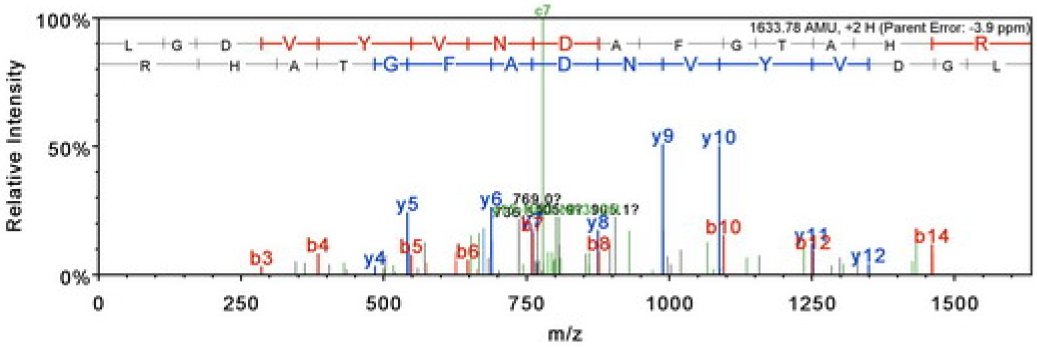

B	B Ions	B+2H	B-NH3	B-H2O	AA	Y Ions	Y+2H	Y-NH3	Y-H2O	Y
1	114.1				L	1634.8	817.9	1617.8	1616.8	15
2	171.1				G	1521.7	761.4	1504.7	1503.7	14
3	286.1			268.1	D	1464.7	732.9	1447.7	1446.7	13
4	385.2			367.2	V	1349.7	675.3	1332.6	1331.7	12
5	548.3			530.3	Y	1250.6	625.8	1233.6	1232.6	11
6	647.3	324.2		629.3	V	1087.5	544.3	1070.5	1069.5	10
7	761.4	381.2	744.4	743.4	N	988.5	494.7	971.4	970.5	9
8	876.4	438.7	859.4	858.4	D	874.4	437.7	857.4	856.4	8
9	947.5	474.2	930.4	929.4	A	759.4	380.2	742.4	741.4	7
10	1094.5	547.8	1077.5	1076.5	F	688.4	344.7	671.3	670.3	6
11	1151.5	576.3	1134.5	1133.5	G	541.3	271.1	524.3	523.3	5
12	1252.6	626.8	1235.6	1234.6	T	484.3	242.6	467.2	466.3	4
13	1323.6	662.3	1306.6	1305.6	A	383.2	192.1	366.2		3
14	1460.7	730.8	1443.7	1442.7	H	312.2	156.6	295.2		2
15	1634.8	817.9	1617.8	1616.8	R	175.1		158.1		1

Gene symbol: Pgrmc2

Protein name: Membrane-associated progesterone receptor component 2

Protein accession numbers: IPI00351206

Peptide sequence: (R)GLCSGPGAGEESPAATLPR(M)

Exclusive (unique to this protein): TRUE

SEQUEST XCorr score: 2.41 SEQUEST DCn score: 0.743

Fix modifications: C3: Carbamidomethyl (+57.02)Variable modifications: None

charge: 2 Observed m/z: 913.9413

Actual minus calculated peptide mass (AMU): 0.003784

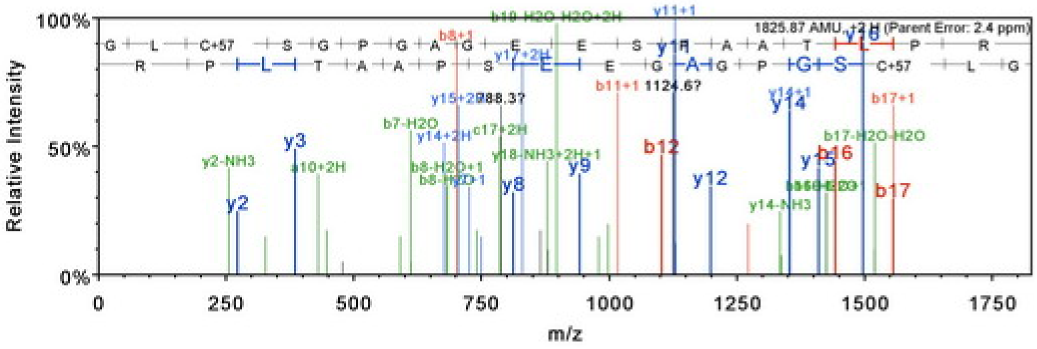

B	B Ions	B+2H	B-NH3	B-H2O	AA	Y Ions	Y+2H	Y-NH3	Y-H2O	Y
1	58.0				G	1826.9	913.9	1809.8	1808.9	19
2	171.1				L	1769.9	885.4	1752.8	1751.8	18
3	331.1				C+57	1656.8	828.9	1639.7	1638.8	17
4	418.2			400.2	S	1496.7	748.9	1479.7	1478.7	16
5	475.2			457.2	G	1409.7	705.4	1392.7	1391.7	15
6	572.3	286.6		554.2	P	1352.7	676.8	1335.7	1334.7	14
7	629.3	315.1		611.3	G	1255.6	628.3	1238.6	1237.6	13
8	700.3	350.7		682.3	A	1198.6	599.8	1181.6	1180.6	12
9	757.3	379.2		739.3	G	1127.6	564.3	1110.5	1109.6	11
10	886.4	443.7		868.4	E	1070.5	535.8	1053.5	1052.5	10
11	1015.4	508.2		997.4	E	941.5	471.3	924.5	923.5	9
12	1102.5	551.7		1084.4	S	812.5	406.7	795.4	794.5	8
13	1199.5	600.3		1181.5	P	725.4	363.2	708.4	707.4	7
14	1270.5	635.8		1252.5	A	628.4	314.7	611.4	610.4	6
15	1341.6	671.3		1323.6	A	557.3		540.3	539.3	5
16	1442.6	721.8		1424.6	T	486.3		469.3	468.3	4
17	1555.7	778.4		1537.7	L	385.3		368.2		3
18	1652.8	826.9		1634.8	P	272.2		255.2		2
19	1826.9	913.9	1809.8	1808.9	R	175.1		158.1		1

Gene symbol: Pitpna

Protein name: Phosphatidylinositol transfer protein alpha isoform

Protein accession numbers: IPI00230003

Peptide sequence: (R)VILPVSVDEYQVGQLYSVAEASK(N)

Exclusive (unique to this protein): TRUE

SEQUEST XCorr score: 2.4 SEQUEST DCn score: 0.638

Fix modifications: None

Variable modifications: None

charge: 2 Observed m/z: 1247.6605

Actual minus calculated peptide mass (AMU): 0.005371

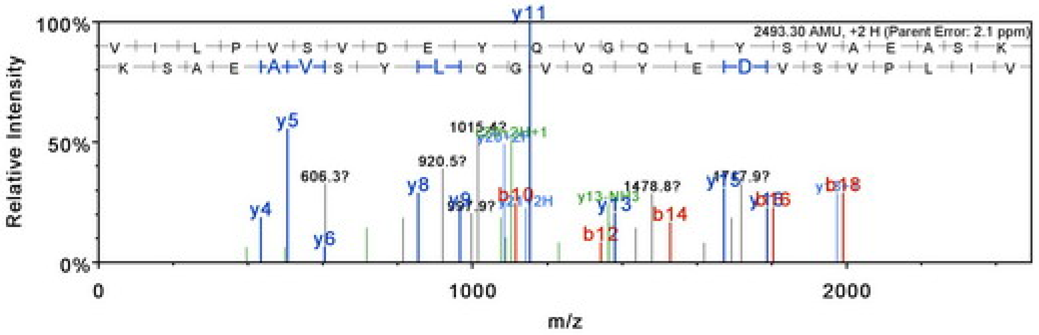

B	B Ions	B+2H	B-NH3	B-H2O	AA	Y Ions	Y+2H	Y-NH3	Y-H2O	Y
1	100.1				V	2494.3	1247.7	2477.3	2476.3	23
2	213.2				I	2395.2	1198.1	2378.2	2377.2	22
3	326.2				L	2282.2	1141.6	2265.1	2264.1	21
4	423.3				P	2169.1	1085.0	2152.0	2151.1	20
5	522.4				V	2072.0	1036.5	2055.0	2054.0	19
6	609.4	305.2		591.4	S	1973.0	987.0	1955.9	1954.9	18
7	708.5	354.7		690.5	V	1885.9	943.5	1868.9	1867.9	17
8	823.5	412.3		805.5	D	1786.9	893.9	1769.8	1768.8	16
9	952.5	476.8		934.5	E	1671.8	836.4	1654.8	1653.8	15
10	1115.6	558.3		1097.6	Y	1542.8	771.9	1525.8	1524.8	14
11	1243.7	622.3	1226.6	1225.7	Q	1379.7	690.4	1362.7	1361.7	13
12	1342.7	671.9	1325.7	1324.7	V	1251.7	626.3	1234.6	1233.7	12
13	1399.8	700.4	1382.7	1381.7	G	1152.6	576.8	1135.6	1134.6	11
14	1527.8	764.4	1510.8	1509.8	Q	1095.6	548.3	1078.5	1077.6	10
15	1640.9	821.0	1623.9	1622.9	L	967.5	484.3	950.5	949.5	9
16	1804.0	902.5	1786.9	1785.9	Y	854.4	427.7	837.4	836.4	8
17	1891.0	946.0	1874.0	1873.0	S	691.4	346.2	674.3	673.4	7
18	1990.1	995.5	1973.0	1972.0	V	604.3	302.7	587.3	586.3	6
19	2061.1	1031.0	2044.1	2043.1	A	505.3		488.2	487.3	5
20	2190.1	1095.6	2173.1	2172.1	E	434.2		417.2	416.2	4
21	2261.2	1131.1	2244.1	2243.2	A	305.2		288.2	287.2	3
22	2348.2	1174.6	2331.2	2330.2	S	234.2		217.1	216.1	2
23	2494.3	1247.7	2477.3	2476.3	K	147.1		130.1		1

Gene symbol: Plaa

Protein name: Phospholipase A-2-activating protein

Protein accession numbers: IPI00226234,IPI00828952

Peptide sequence: (R)YVPGTSGPSNTVQTADPFTGAGR(Y)

Exclusive (unique to this protein): TRUE

SEQUEST XCorr score: 2.03 SEQUEST DCn score: 0.489

Fix modifications: None

Variable modifications: None

charge: 2 Observed m/z: 1140.5455

Actual minus calculated peptide mass (AMU): -0.006592

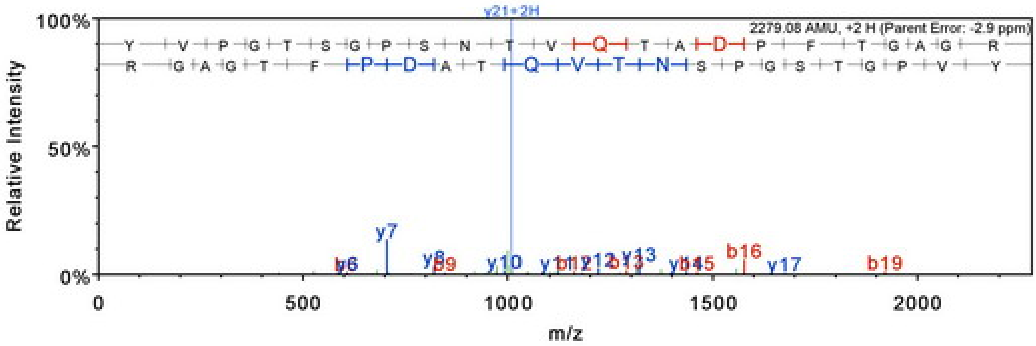

B	B Ions	B+2H	B-NH3	B-H2O	AA	Y Ions	Y+2H	Y-NH3	Y-H2O	Y
1	164.1				Y	2280.1	1140.5	2263.1	2262.1	23
2	263.1				V	2117.0	1059.0	2100.0	2099.0	22
3	360.2				P	2018.0	1009.5	2000.9	2000.0	21
4	417.2				G	1920.9	961.0	1903.9	1902.9	20
5	518.3			500.3	T	1863.9	932.5	1846.9	1845.9	19
6	605.3	303.2		587.3	S	1762.8	881.9	1745.8	1744.8	18
7	662.3	331.7		644.3	G	1675.8	838.4	1658.8	1657.8	17
8	759.4	380.2		741.4	P	1618.8	809.9	1601.8	1600.8	16
9	846.4	423.7		828.4	S	1521.7	761.4	1504.7	1503.7	15
10	960.4	480.7	943.4	942.4	N	1434.7	717.9	1417.7	1416.7	14
11	1061.5	531.3	1044.5	1043.5	T	1320.7	660.8	1303.6	1302.6	13
12	1160.6	580.8	1143.5	1142.5	V	1219.6	610.3	1202.6	1201.6	12
13	1288.6	644.8	1271.6	1270.6	Q	1120.5	560.8	1103.5	1102.5	11
14	1389.7	695.3	1372.6	1371.7	T	992.5	496.7	975.5	974.5	10
15	1460.7	730.9	1443.7	1442.7	A	891.4	446.2	874.4	873.4	9
16	1575.7	788.4	1558.7	1557.7	D	820.4	410.7	803.4	802.4	8
17	1672.8	836.9	1655.8	1654.8	P	705.4	353.2	688.3	687.4	7
18	1819.9	910.4	1802.8	1801.8	F	608.3	304.7	591.3	590.3	6
19	1920.9	961.0	1903.9	1902.9	T	461.3		444.2	443.2	5
20	1977.9	989.5	1960.9	1959.9	G	360.2		343.2		4
21	2049.0	1025.0	2031.9	2031.0	A	303.2		286.2		3
22	2106.0	1053.5	2088.9	2088.0	G	232.1		215.1		2
23	2280.1	1140.5	2263.1	2262.1	R	175.1		158.1		1

Gene symbol: Polr2d

Protein name: DNA-directed RNA polymerase II subunit RPB4

Protein accession numbers: IPI00268215

Peptide sequence: (R)AGDVEEDASQLIFPK(E)

Exclusive (unique to this protein): TRUE

SEQUEST XCorr score: 2.28 SEQUEST DCn score: 0.395

Fix modifications: None

Variable modifications: None

charge: 2 Observed m/z: 809.9033

Actual minus calculated peptide mass (AMU): 0.0021955

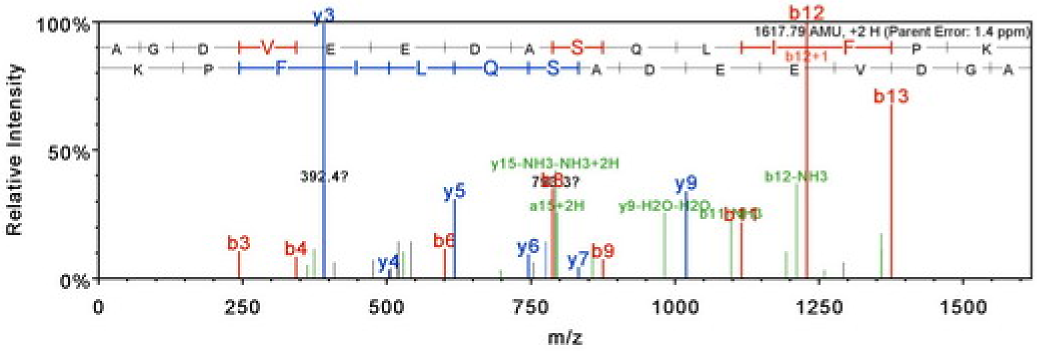

B	B Ions	B+2H	B-NH3	B-H2O	AA	Y Ions	Y+2H	Y-NH3	Y-H2O	Y
1	72.0				A	1618.8	809.9	1601.8	1600.8	15
2	129.1				G	1547.8	774.4	1530.7	1529.8	14
3	244.1			226.1	D	1490.7	745.9	1473.7	1472.7	13
4	343.2			325.2	V	1375.7	688.4	1358.7	1357.7	12
5	472.2			454.2	E	1276.6	638.8	1259.6	1258.6	11
6	601.3	301.1		583.2	E	1147.6	574.3	1130.6	1129.6	10
7	716.3	358.6		698.3	D	1018.6	509.8	1001.5	1000.6	9
8	787.3	394.2		769.3	A	903.5	452.3	886.5	885.5	8
9	874.3	437.7		856.3	S	832.5	416.8	815.5	814.5	7
10	1002.4	501.7	985.4	984.4	Q	745.5	373.2	728.4		6
11	1115.5	558.3	1098.5	1097.5	L	617.4		600.4		5
12	1228.6	614.8	1211.5	1210.6	I	504.3		487.3		4
13	1375.6	688.3	1358.6	1357.6	F	391.2		374.2		3
14	1472.7	736.9	1455.7	1454.7	P	244.2		227.1		2
15	1618.8	809.9	1601.8	1600.8	K	147.1		130.1		1

Gene symbol: Ppp2cb

Protein name: Serine/threonine-protein phosphatase 2A catalytic subunit beta isoform

Protein accession numbers: IPI00111556,IPI00120374,IPI00473972,IPI00751938

Peptide sequence: (R)NVVTIFSAPNYCYR(C)

Exclusive (unique to this protein): TRUE

SEQUEST XCorr score: 2.58 SEQUEST DCn score: 0.508

Fix modifications: C12: Carbamidomethyl (+57.02)Variable modifications: None

charge: 2 Observed m/z: 852.413

Actual minus calculated peptide mass (AMU): -0.003296

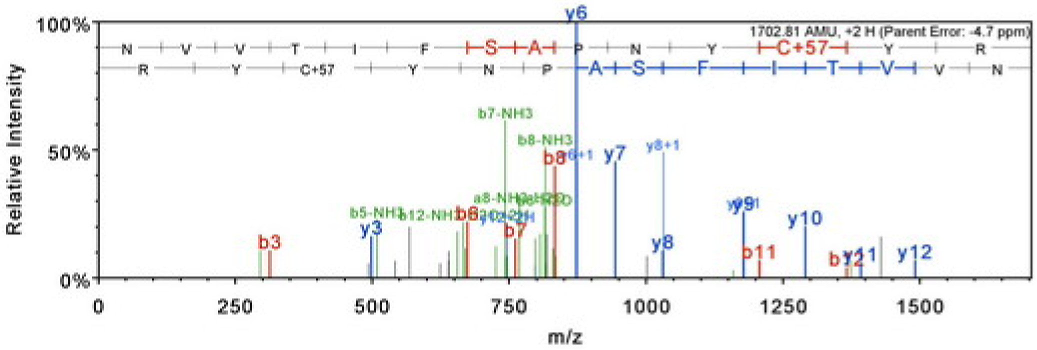

B	B Ions	B+2H	B-NH3	B-H2O	AA	Y Ions	Y+2H	Y-NH3	Y-H2O	Y
1	115.1		98.0		N	1703.8	852.4	1686.8	1685.8	14
2	214.1		197.1		V	1589.8	795.4	1572.8	1571.8	13
3	313.2		296.2		V	1490.7	745.9	1473.7	1472.7	12
4	414.2		397.2	396.2	T	1391.6	696.3	1374.6	1373.6	11
5	527.3		510.3	509.3	I	1290.6	645.8	1273.6	1272.6	10
6	674.4	337.7	657.4	656.4	F	1177.5	589.3	1160.5	1159.5	9
7	761.4	381.2	744.4	743.4	S	1030.4	515.7	1013.4	1012.4	8
8	832.5	416.7	815.4	814.5	A	943.4	472.2	926.4		7
9	929.5	465.3	912.5	911.5	P	872.4	436.7	855.4		6
10	1043.5	522.3	1026.5	1025.5	N	775.3		758.3		5
11	1206.6	603.8	1189.6	1188.6	Y	661.3		644.3		4
12	1366.7	683.8	1349.6	1348.6	C+57	498.2		481.2		3
13	1529.7	765.4	1512.7	1511.7	Y	338.2		321.2		2
14	1703.8	852.4	1686.8	1685.8	R	175.1		158.1		1

Gene symbol: Ppp2r1b

Protein name: Adult male testis cDNA, RIKEN full-length enriched library, clone:4933415I16 product:SERINE/THREONINE PROTEIN PHOSPHATASE 2A, 65 kDa REGULATORY SUBUNIT A, BETA ISOFORM (PP2A, SUBUNIT A, PR65-BETA ISOFORM) (PP2A, SUBUNIT A, R1-BETA ISOFORM) homolog

Protein accession numbers: IPI00222306,IPI00655135

Peptide sequence: (R)QLSQSLLPAIVELAEDAK(W)

Exclusive (unique to this protein): TRUE

SEQUEST XCorr score: 2.84 SEQUEST DCn score: 0.578

Fix modifications: None

Variable modifications: None

charge: 2 Observed m/z: 963.0317

Actual minus calculated peptide mass (AMU): -0.0040256

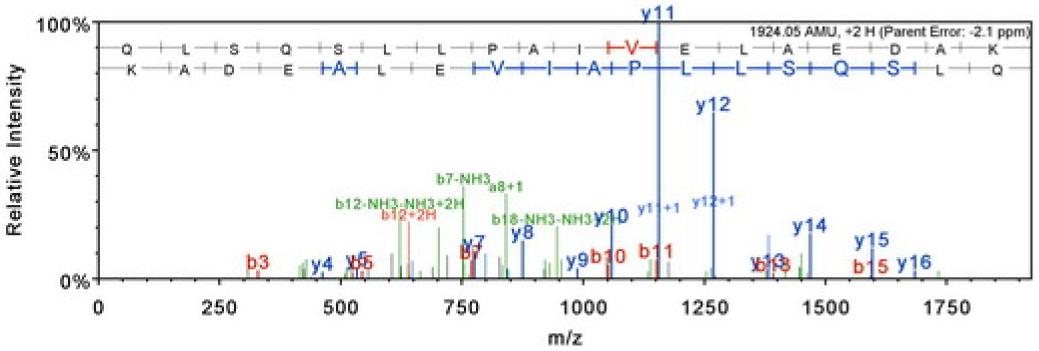

B	B Ions	B+2H	B-NH3	B-H2O	AA	Y Ions	Y+2H	Y-NH3	Y-H2O	Y
1	129.1		112.0		Q	1925.1	963.0	1908.0	1907.1	18
2	242.2		225.1		L	1797.0	899.0	1780.0	1779.0	17
3	329.2		312.2	311.2	S	1683.9	842.5	1666.9	1665.9	16
4	457.2		440.2	439.2	Q	1596.9	799.0	1579.9	1578.9	15
5	544.3		527.3	526.3	S	1468.8	734.9	1451.8	1450.8	14
6	657.4	329.2	640.3	639.4	L	1381.8	691.4	1364.8	1363.8	13
7	770.4	385.7	753.4	752.4	L	1268.7	634.9	1251.7	1250.7	12
8	867.5	434.3	850.5	849.5	P	1155.6	578.3	1138.6	1137.6	11
9	938.5	469.8	921.5	920.5	A	1058.6	529.8	1041.5	1040.6	10
10	1051.6	526.3	1034.6	1033.6	I	987.5	494.3	970.5	969.5	9
11	1150.7	575.9	1133.7	1132.7	V	874.5	437.7	857.4	856.4	8
12	1279.7	640.4	1262.7	1261.7	E	775.4	388.2	758.4	757.4	7
13	1392.8	696.9	1375.8	1374.8	L	646.3	323.7	629.3	628.3	6
14	1463.9	732.4	1446.8	1445.8	A	533.3		516.2	515.3	5
15	1592.9	797.0	1575.9	1574.9	E	462.2		445.2	444.2	4
16	1707.9	854.5	1690.9	1689.9	D	333.2		316.2	315.2	3
17	1779.0	890.0	1761.9	1760.9	A	218.2		201.1		2
18	1925.1	963.0	1908.0	1907.1	K	147.1		130.1		1

Gene symbol: Ppp2r4

Protein name: Serine/threonine-protein phosphatase 2A regulatory subunit B'

Protein accession numbers: IPI00118723

Peptide sequence: (R)WIDETPPVDQPSR(F)

Exclusive (unique to this protein): TRUE

SEQUEST XCorr score: 2.14 SEQUEST DCn score: 0.638

Fix modifications: None

Variable modifications: None

charge: 2 Observed m/z: 770.3733

Actual minus calculated peptide mass (AMU): -0.0059849

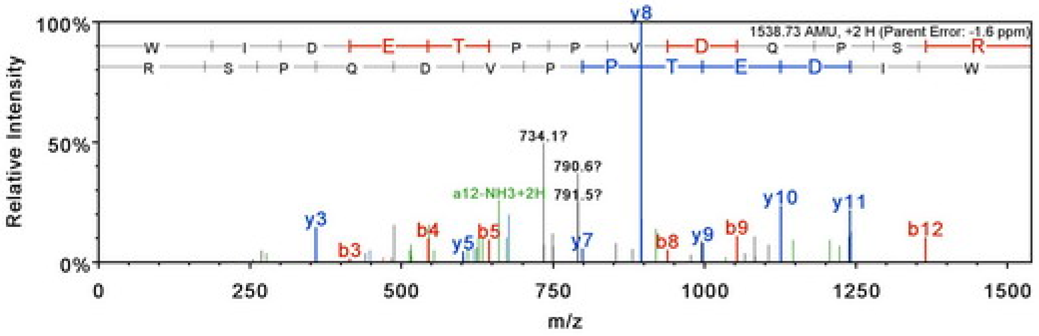

B	B Ions	B+2H	B-NH3	B-H2O	AA	Y Ions	Y+2H	Y-NH3	Y-H2O	Y
1	187.1				W	1539.7	770.4	1522.7	1521.7	13
2	300.2				I	1353.7	677.3	1336.6	1335.7	12
3	415.2			397.2	D	1240.6	620.8	1223.5	1222.6	11
4	544.2			526.2	E	1125.5	563.3	1108.5	1107.5	10
5	645.3			627.3	T	996.5	498.8	979.5	978.5	9
6	742.3	371.7		724.3	P	895.5	448.2	878.4	877.5	8
7	839.4	420.2		821.4	P	798.4	399.7	781.4	780.4	7
8	938.5	469.7		920.5	V	701.4	351.2	684.3	683.4	6
9	1053.5	527.3		1035.5	D	602.3		585.3	584.3	5
10	1181.5	591.3	1164.5	1163.5	Q	487.3		470.2	469.3	4
11	1278.6	639.8	1261.6	1260.6	P	359.2		342.2	341.2	3
12	1365.6	683.3	1348.6	1347.6	S	262.1		245.1	244.1	2
13	1539.7	770.4	1522.7	1521.7	R	175.1		158.1		1

Gene symbol: Prei3

Protein name: Preimplantation protein 3

Protein accession numbers: IPI00420832

Peptide sequence: (R)QIFDEYENETFLCHR(F)

Exclusive (unique to this protein): TRUE

SEQUEST XCorr score: 2.06 SEQUEST DCn score: 0.593

Fix modifications: C13: Carbamidomethyl (+57.02)Variable modifications: None

charge: 2 Observed m/z: 1000.9418

Actual minus calculated peptide mass (AMU): -0.005737

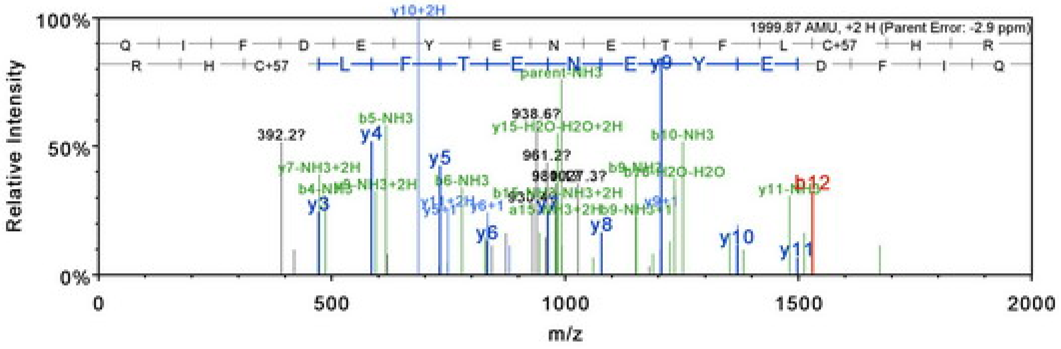

B	B Ions	B+2H	B-NH3	B-H2O	AA	Y Ions	Y+2H	Y-NH3	Y-H2O	Y
1	129.1		112.0		Q	2000.9	1000.9	1983.9	1982.9	15
2	242.2		225.1		I	1872.8	936.9	1855.8	1854.8	14
3	389.2		372.2		F	1759.7	880.4	1742.7	1741.7	13
4	504.3		487.2	486.2	D	1612.7	806.8	1595.6	1594.7	12
5	633.3		616.3	615.3	E	1497.6	749.3	1480.6	1479.6	11
6	796.4	398.7	779.3	778.3	Y	1368.6	684.8	1351.6	1350.6	10
7	925.4	463.2	908.4	907.4	E	1205.5	603.3	1188.5	1187.5	9
8	1039.4	520.2	1022.4	1021.4	N	1076.5	538.8	1059.5	1058.5	8
9	1168.5	584.7	1151.5	1150.5	E	962.5	481.7	945.4	944.4	7
10	1269.5	635.3	1252.5	1251.5	T	833.4	417.2	816.4	815.4	6
11	1416.6	708.8	1399.6	1398.6	F	732.4	366.7	715.3		5
12	1529.7	765.3	1512.7	1511.7	L	585.3	293.2	568.3		4
13	1689.7	845.4	1672.7	1671.7	C+57	472.2	236.6	455.2		3
14	1826.8	913.9	1809.7	1808.8	H	312.2	156.6	295.2		2
15	2000.9	1000.9	1983.9	1982.9	R	175.1		158.1		1

Gene symbol: Prss1

Protein name: Trypsinogen 16

Protein accession numbers: IPI00130391

Peptide sequence: (R)LGEHNINVLEGNEQFIDAAK(I)

Exclusive (unique to this protein): TRUE

SEQUEST XCorr score: 2.03 SEQUEST DCn score: 0.356

Fix modifications: None

Variable modifications: None

charge: 2 Observed m/z: 1106.0525

Actual minus calculated peptide mass (AMU): -0.007324

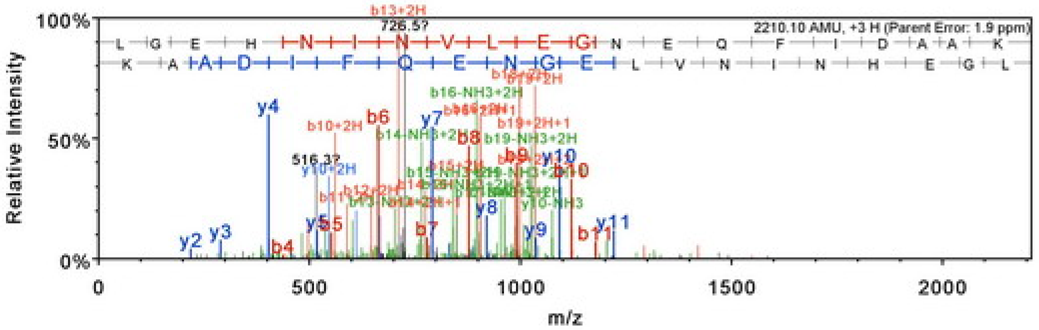

B	B Ions	B+2H	B-NH3	B-H2O	AA	Y Ions	Y+2H	Y-NH3	Y-H2O	Y
1	114.1				L	2211.1	1106.1	2194.1	2193.1	20
2	171.1				G	2098.0	1049.5	2081.0	2080.0	19
3	300.2			282.2	E	2041.0	1021.0	2024.0	2023.0	18
4	437.2	219.1		419.2	H	1912.0	956.5	1894.9	1894.0	17
5	551.3	276.1	534.2	533.3	N	1774.9	888.0	1757.9	1756.9	16
6	664.3	332.7	647.3	646.3	I	1660.9	830.9	1643.8	1642.8	15
7	778.4	389.7	761.4	760.4	N	1547.8	774.4	1530.7	1529.8	14
8	877.5	439.2	860.4	859.4	V	1433.7	717.4	1416.7	1415.7	13
9	990.5	495.8	973.5	972.5	L	1334.7	667.8	1317.6	1316.7	12
10	1119.6	560.3	1102.5	1101.6	E	1221.6	611.3	1204.5	1203.6	11
11	1176.6	588.8	1159.6	1158.6	G	1092.5	546.8	1075.5	1074.5	10
12	1290.6	645.8	1273.6	1272.6	N	1035.5	518.3	1018.5	1017.5	9
13	1419.7	710.4	1402.7	1401.7	E	921.5	461.2	904.4	903.5	8
14	1547.8	774.4	1530.7	1529.7	Q	792.4	396.7	775.4	774.4	7
15	1694.8	847.9	1677.8	1676.8	F	664.4	332.7	647.3	646.4	6
16	1807.9	904.5	1790.9	1789.9	I	517.3		500.3	499.3	5
17	1922.9	962.0	1905.9	1904.9	D	404.2		387.2	386.2	4
18	1994.0	997.5	1976.9	1976.0	A	289.2		272.2		3
19	2065.0	1033.0	2048.0	2047.0	A	218.2		201.1		2
20	2211.1	1106.1	2194.1	2193.1	K	147.1		130.1		1

Gene symbol: Psen1

Protein name: Isoform 1 of Presenilin-1

Protein accession numbers: IPI00117124

Peptide sequence: (R)AAVQELSGSILTSEDPEER(G)

Exclusive (unique to this protein): TRUE

SEQUEST XCorr score: 2.49 SEQUEST DCn score: 0.689

Fix modifications: None

Variable modifications: None

charge: 2 Observed m/z: 1016.0005

Actual minus calculated peptide mass (AMU): 0.004883

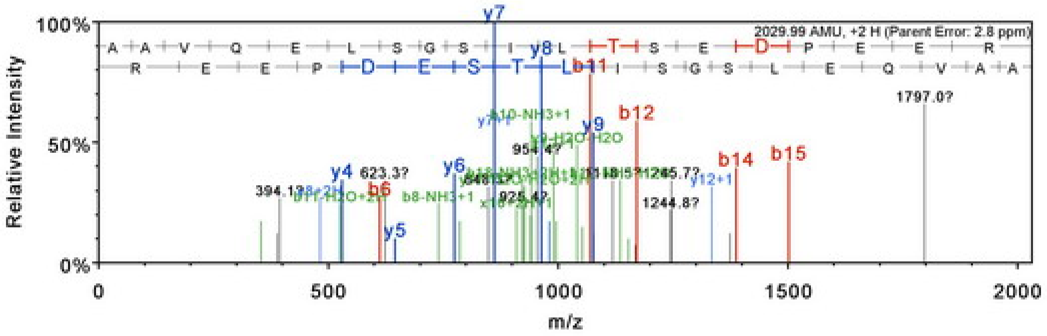

B	B Ions	B+2H	B-NH3	B-H2O	AA	Y Ions	Y+2H	Y-NH3	Y-H2O	Y
1	72.0				A	2031.0	1016.0	2014.0	2013.0	19
2	143.1				A	1960.0	980.5	1942.9	1941.9	18
3	242.2				V	1888.9	945.0	1871.9	1870.9	17
4	370.2		353.2		Q	1789.9	895.4	1772.8	1771.8	16
5	499.3		482.2	481.2	E	1661.8	831.4	1644.8	1643.8	15
6	612.3	306.7	595.3	594.3	L	1532.7	766.9	1515.7	1514.7	14
7	699.4	350.2	682.3	681.4	S	1419.7	710.3	1402.6	1401.7	13
8	756.4	378.7	739.4	738.4	G	1332.6	666.8	1315.6	1314.6	12
9	843.4	422.2	826.4	825.4	S	1275.6	638.3	1258.6	1257.6	11
10	956.5	478.8	939.5	938.5	I	1188.6	594.8	1171.5	1170.6	10
11	1069.6	535.3	1052.6	1051.6	L	1075.5	538.3	1058.5	1057.5	9
12	1170.6	585.8	1153.6	1152.6	T	962.4	481.7	945.4	944.4	8
13	1257.7	629.3	1240.6	1239.7	S	861.4	431.2	844.3	843.4	7
14	1386.7	693.9	1369.7	1368.7	E	774.3	387.7	757.3	756.3	6
15	1501.7	751.4	1484.7	1483.7	D	645.3		628.3	627.3	5
16	1598.8	799.9	1581.8	1580.8	P	530.3		513.2	512.3	4
17	1727.8	864.4	1710.8	1709.8	E	433.2		416.2	415.2	3
18	1856.9	928.9	1839.9	1838.9	E	304.2		287.1	286.2	2
19	2031.0	1016.0	2014.0	2013.0	R	175.1		158.1		1

Gene symbol: Psma1

Protein name: Proteasome subunit alpha type 1

Protein accession numbers: IPI00283862

Peptide sequence: (R)NQYDNDVTVWSPQGR(I)

Exclusive (unique to this protein): TRUE

SEQUEST XCorr score: 2.23 SEQUEST DCn score: 0.646

Fix modifications: None

Variable modifications: None

charge: 2 Observed m/z: 889.9113

Actual minus calculated peptide mass (AMU): 0.004883

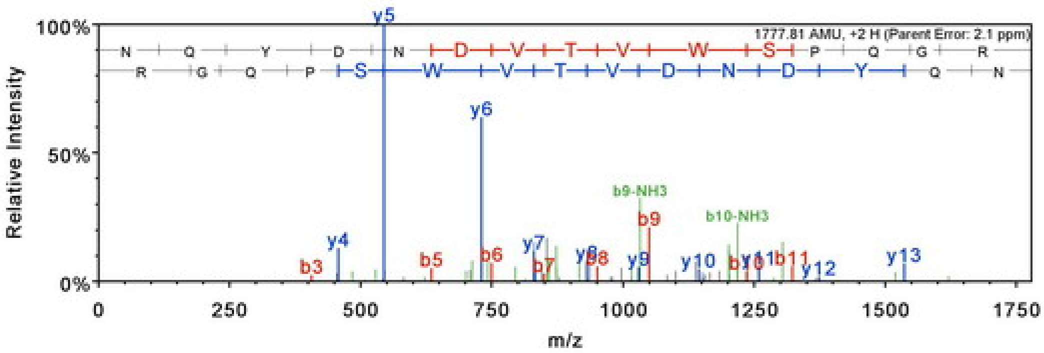

B	B Ions	B+2H	B-NH3	B-H2O	AA	Y Ions	Y+2H	Y-NH3	Y-H2O	Y
1	115.1		98.0		N	1778.8	889.9	1761.8	1760.8	15
2	243.1		226.1		Q	1664.8	832.9	1647.7	1646.8	14
3	406.2		389.2		Y	1536.7	768.9	1519.7	1518.7	13
4	521.2		504.2	503.2	D	1373.6	687.3	1356.6	1355.6	12
5	635.2		618.2	617.2	N	1258.6	629.8	1241.6	1240.6	11
6	750.3	375.6	733.2	732.3	D	1144.6	572.8	1127.5	1126.6	10
7	849.3	425.2	832.3	831.3	V	1029.5	515.3	1012.5	1011.5	9
8	950.4	475.7	933.4	932.4	T	930.5	465.7	913.5	912.5	8
9	1049.5	525.2	1032.4	1031.4	V	829.4	415.2	812.4	811.4	7
10	1235.5	618.3	1218.5	1217.5	W	730.4	365.7	713.3	712.4	6
11	1322.6	661.8	1305.5	1304.5	S	544.3		527.3	526.3	5
12	1419.6	710.3	1402.6	1401.6	P	457.3		440.2		4
13	1547.7	774.3	1530.7	1529.7	Q	360.2		343.2		3
14	1604.7	802.9	1587.7	1586.7	G	232.1		215.1		2
15	1778.8	889.9	1761.8	1760.8	R	175.1		158.1		1

Gene symbol: Psma2

Protein name: In vitro fertilized eggs cDNA, RIKEN full-length enriched library, clone:7420458H06 product:proteasome (prosome, macropain) subunit, alpha type 2, full insert sequence

Protein accession numbers: IPI00420745

Peptide sequence: (K)LAQQYYLVYQEPIPTAQLVQR(V)

Exclusive (unique to this protein): TRUE

SEQUEST XCorr score: 2.59 SEQUEST DCn score: 0.773

Fix modifications: None

Variable modifications: None

charge: 2 Observed m/z: 1261.1738

Actual minus calculated peptide mass (AMU): -0.005859

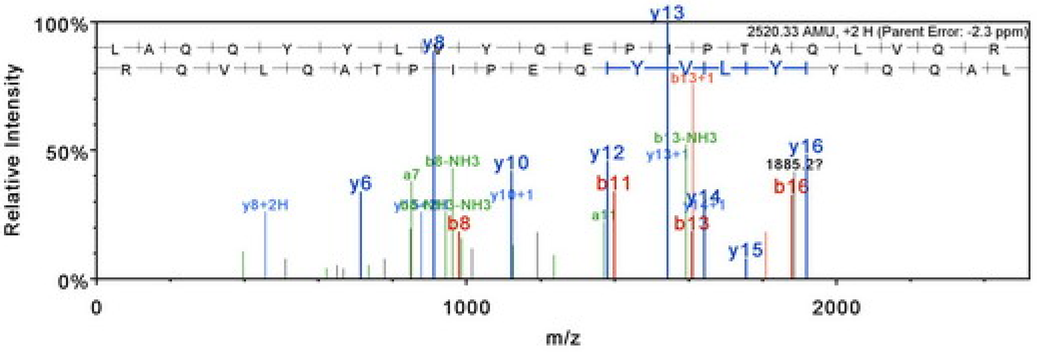

B	B Ions	B+2H	B-NH3	B-H2O	AA	Y Ions	Y+2H	Y-NH3	Y-H2O	Y
1	114.1				L	2521.3	1261.2	2504.3	2503.3	21
2	185.1				A	2408.3	1204.6	2391.2	2390.3	20
3	313.2		296.2		Q	2337.2	1169.1	2320.2	2319.2	19
4	441.3		424.2		Q	2209.2	1105.1	2192.1	2191.2	18
5	604.3		587.3		Y	2081.1	1041.1	2064.1	2063.1	17
6	767.4	384.2	750.4		Y	1918.0	959.5	1901.0	1900.0	16
7	880.5	440.7	863.4		L	1755.0	878.0	1738.0	1737.0	15
8	979.5	490.3	962.5		V	1641.9	821.5	1624.9	1623.9	14
9	1142.6	571.8	1125.6		Y	1542.8	771.9	1525.8	1524.8	13
10	1270.7	635.8	1253.6		Q	1379.8	690.4	1362.7	1361.8	12
11	1399.7	700.4	1382.7	1381.7	E	1251.7	626.4	1234.7	1233.7	11
12	1496.7	748.9	1479.7	1478.7	P	1122.7	561.8	1105.6	1104.7	10
13	1609.8	805.4	1592.8	1591.8	I	1025.6	513.3	1008.6	1007.6	9
14	1706.9	853.9	1689.9	1688.9	P	912.5	456.8	895.5	894.5	8
15	1807.9	904.5	1790.9	1789.9	T	815.5	408.2	798.5	797.5	7
16	1879.0	940.0	1861.9	1861.0	A	714.4	357.7	697.4		6
17	2007.0	1004.0	1990.0	1989.0	Q	643.4		626.4		5
18	2120.1	1060.6	2103.1	2102.1	L	515.3		498.3		4
19	2219.2	1110.1	2202.2	2201.2	V	402.3		385.2		3
20	2347.2	1174.1	2330.2	2329.2	Q	303.2		286.2		2
21	2521.3	1261.2	2504.3	2503.3	R	175.1		158.1		1

Gene symbol: Psma4

Protein name: Proteasome subunit alpha type 4

Protein accession numbers: IPI00277001

Peptide sequence: (K)LLDEVFFSEK(I)

Exclusive (unique to this protein): TRUE

SEQUEST XCorr score: 2.08 SEQUEST DCn score: 0.543

Fix modifications: None

Variable modifications: None

charge: 2 Observed m/z: 613.8148

Actual minus calculated peptide mass (AMU): -0.0092755

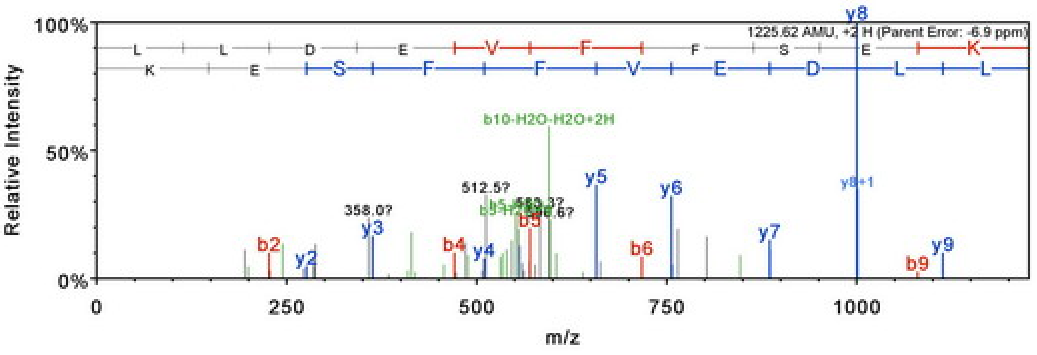

B	B Ions	B+2H	B-NH3	B-H2O	AA	Y Ions	Y+2H	Y-NH3	Y-H2O	Y
1	114.1				L	1226.6	613.8	1209.6	1208.6	10
2	227.2				L	1113.5	557.3	1096.5	1095.5	9
3	342.2			324.2	D	1000.5	500.7	983.4	982.5	8
4	471.3			453.2	E	885.4	443.2	868.4	867.4	7
5	570.3			552.3	V	756.4	378.7	739.4	738.4	6
6	717.4	359.2		699.4	F	657.3		640.3	639.3	5
7	864.5	432.7		846.4	F	510.3		493.2	492.3	4
8	951.5	476.3		933.5	S	363.2		346.2	345.2	3
9	1080.5	540.8		1062.5	E	276.2		259.1	258.1	2
10	1226.6	613.8	1209.6	1208.6	K	147.1		130.1		1

Gene symbol: Psmb1

Protein name: Proteasome subunit beta type 1 precursor

Protein accession numbers: IPI00113845

Peptide sequence: (K)GAVYSFDPVGSYQR(D)

Exclusive (unique to this protein): TRUE

SEQUEST XCorr score: 2.73 SEQUEST DCn score: 0.563

Fix modifications: None

Variable modifications: None

charge: 2 Observed m/z: 773.3669

Actual minus calculated peptide mass (AMU): -0.00769

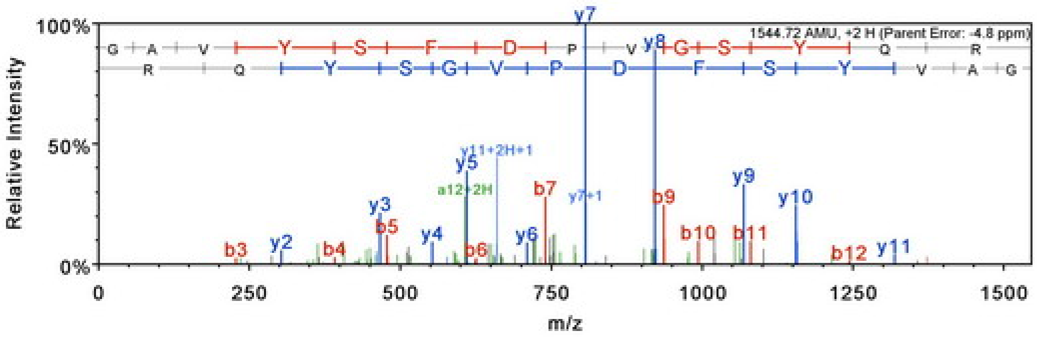

B	B Ions	B+2H	B-NH3	B-H2O	AA	Y Ions	Y+2H	Y-NH3	Y-H2O	Y
1	58.0				G	1545.7	773.4	1528.7	1527.7	14
2	129.1				A	1488.7	744.9	1471.7	1470.7	13
3	228.1				V	1417.7	709.3	1400.7	1399.7	12
4	391.2				Y	1318.6	659.8	1301.6	1300.6	11
5	478.2			460.2	S	1155.5	578.3	1138.5	1137.5	10
6	625.3	313.2		607.3	F	1068.5	534.8	1051.5	1050.5	9
7	740.3	370.7		722.3	D	921.4	461.2	904.4	903.4	8
8	837.4	419.2		819.4	P	806.4	403.7	789.4	788.4	7
9	936.5	468.7		918.4	V	709.4	355.2	692.3	691.4	6
10	993.5	497.2		975.5	G	610.3		593.3	592.3	5
11	1080.5	540.8		1062.5	S	553.3		536.3	535.3	4
12	1243.6	622.3		1225.5	Y	466.2		449.2		3
13	1371.6	686.3	1354.6	1353.6	Q	303.2		286.2		2
14	1545.7	773.4	1528.7	1527.7	R	175.1		158.1		1

Gene symbol: Psmc6

Protein name: 26S protease regulatory subunit S10B

Protein accession numbers: IPI00125971

Peptide sequence: (K)ALQSVGQIVGEVLK(Q)

Exclusive (unique to this protein): TRUE

SEQUEST XCorr score: 2.37 SEQUEST DCn score: 0.518

Fix modifications: None

Variable modifications: None

charge: 2 Observed m/z: 720.9257

Actual minus calculated peptide mass (AMU): 0.0008545

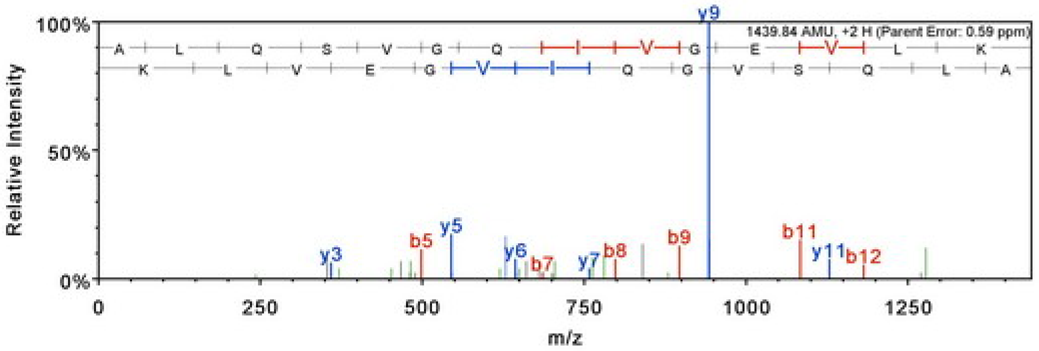

B	B Ions	B+2H	B-NH3	B-H2O	AA	Y Ions	Y+2H	Y-NH3	Y-H2O	Y
1	72.0				A	1440.8	720.9	1423.8	1422.8	14
2	185.1				L	1369.8	685.4	1352.8	1351.8	13
3	313.2		296.2		Q	1256.7	628.9	1239.7	1238.7	12
4	400.2		383.2	382.2	S	1128.7	564.8	1111.6	1110.7	11
5	499.3		482.3	481.3	V	1041.6	521.3	1024.6	1023.6	10
6	556.3	278.7	539.3	538.3	G	942.6	471.8	925.5	924.6	9
7	684.4	342.7	667.3	666.4	Q	885.5	443.3	868.5	867.5	8
8	797.5	399.2	780.4	779.4	I	757.5	379.3	740.5	739.5	7
9	896.5	448.8	879.5	878.5	V	644.4	322.7	627.4	626.4	6
10	953.5	477.3	936.5	935.5	G	545.3		528.3	527.3	5
11	1082.6	541.8	1065.6	1064.6	E	488.3		471.3	470.3	4
12	1181.7	591.3	1164.6	1163.6	V	359.3		342.2		3
13	1294.7	647.9	1277.7	1276.7	L	260.2		243.2		2
14	1440.8	720.9	1423.8	1422.8	K	147.1		130.1		1

Gene symbol: Psme3

Protein name: Proteasome activator complex subunit 3

Protein accession numbers: IPI00113660,IPI00649191

Peptide sequence: (R)TVESEAASYLDQISR(Y)

Exclusive (unique to this protein): TRUE

SEQUEST XCorr score: 2.05 SEQUEST DCn score: 0.331

Fix modifications: None

Variable modifications: None

charge: 2 Observed m/z: 834.9106

Actual minus calculated peptide mass (AMU): 0.005493

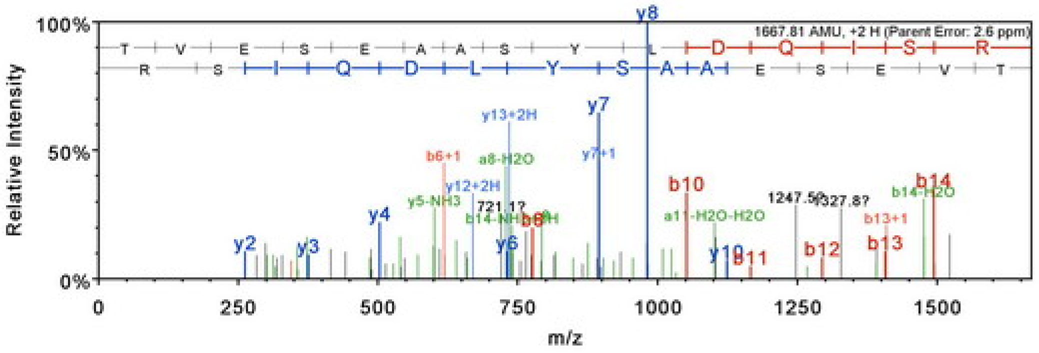

B	B Ions	B+2H	B-NH3	B-H2O	AA	Y Ions	Y+2H	Y-NH3	Y-H2O	Y
1	102.1			84.0	T	1668.8	834.9	1651.8	1650.8	15
2	201.1			183.1	V	1567.8	784.4	1550.7	1549.8	14
3	330.2			312.2	E	1468.7	734.9	1451.7	1450.7	13
4	417.2			399.2	S	1339.7	670.3	1322.6	1321.6	12
5	546.2			528.2	E	1252.6	626.8	1235.6	1234.6	11
6	617.3	309.1		599.3	A	1123.6	562.3	1106.5	1105.6	10
7	688.3	344.7		670.3	A	1052.5	526.8	1035.5	1034.5	9
8	775.4	388.2		757.3	S	981.5	491.3	964.5	963.5	8
9	938.4	469.7		920.4	Y	894.5	447.7	877.4	876.5	7
10	1051.5	526.3		1033.5	L	731.4	366.2	714.4	713.4	6
11	1166.5	583.8		1148.5	D	618.3		601.3	600.3	5
12	1294.6	647.8	1277.5	1276.6	Q	503.3		486.3	485.3	4
13	1407.7	704.3	1390.6	1389.7	I	375.2		358.2	357.2	3
14	1494.7	747.9	1477.7	1476.7	S	262.1		245.1	244.1	2
15	1668.8	834.9	1651.8	1650.8	R	175.1		158.1		1

Gene symbol: Pycrl

Protein name: pyrroline-5-carboxylate reductase-like

Protein accession numbers: IPI00153234

Peptide sequence: (R)TDVLTPAGTTIHGLHALER(G)

Exclusive (unique to this protein): TRUE

SEQUEST XCorr score: 2.24 SEQUEST DCn score: 0.479

Fix modifications: None

Variable modifications: None

charge: 2 Observed m/z: 1001.5414

Actual minus calculated peptide mass (AMU): 0.002319

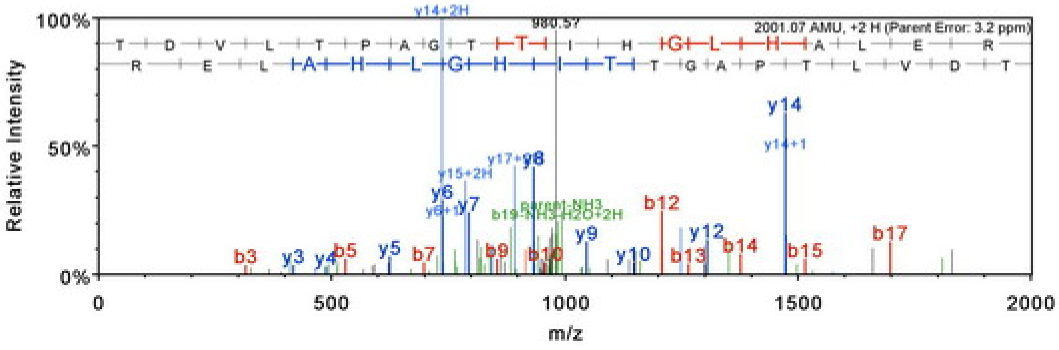

B	B Ions	B+2H	B-NH3	B-H2O	AA	Y Ions	Y+2H	Y-NH3	Y-H2O	Y
1	102.1			84.0	T	2002.1	1001.5	1985.1	1984.1	19
2	217.1			199.1	D	1901.0	951.0	1884.0	1883.0	18
3	316.2			298.1	V	1786.0	893.5	1769.0	1768.0	17
4	429.2			411.2	L	1686.9	844.0	1669.9	1668.9	16
5	530.3			512.3	T	1573.9	787.4	1556.8	1555.8	15
6	627.3	314.2		609.3	P	1472.8	736.9	1455.8	1454.8	14
7	698.4	349.7		680.4	A	1375.7	688.4	1358.7	1357.7	13
8	755.4	378.2		737.4	G	1304.7	652.9	1287.7	1286.7	12
9	856.4	428.7		838.4	T	1247.7	624.4	1230.7	1229.7	11
10	957.5	479.3		939.5	T	1146.6	573.8	1129.6	1128.6	10
11	1070.6	535.8		1052.6	I	1045.6	523.3	1028.6	1027.6	9
12	1207.6	604.3		1189.6	H	932.5	466.8	915.5	914.5	8
13	1264.7	632.8		1246.6	G	795.5	398.2	778.4	777.4	7
14	1377.7	689.4		1359.7	L	738.4	369.7	721.4	720.4	6
15	1514.8	757.9		1496.8	H	625.3	313.2	608.3	607.3	5
16	1585.8	793.4		1567.8	A	488.3		471.3	470.3	4
17	1698.9	850.0		1680.9	L	417.3		400.2	399.2	3
18	1828.0	914.5		1810.0	E	304.2		287.1	286.2	2
19	2002.1	1001.5	1985.1	1984.1	R	175.1		158.1		1

Gene symbol: Qdpr

Protein name: Dihydropteridine reductase

Protein accession numbers: IPI00459279

Peptide sequence: (K)MTDSFTEQADQVTADVGK(L)

Exclusive (unique to this protein): TRUE

SEQUEST XCorr score: 2.29 SEQUEST DCn score: 0.619

Fix modifications: None

Variable modifications: None

charge: 2 Observed m/z: 971.9413

Actual minus calculated peptide mass (AMU): 0.00415

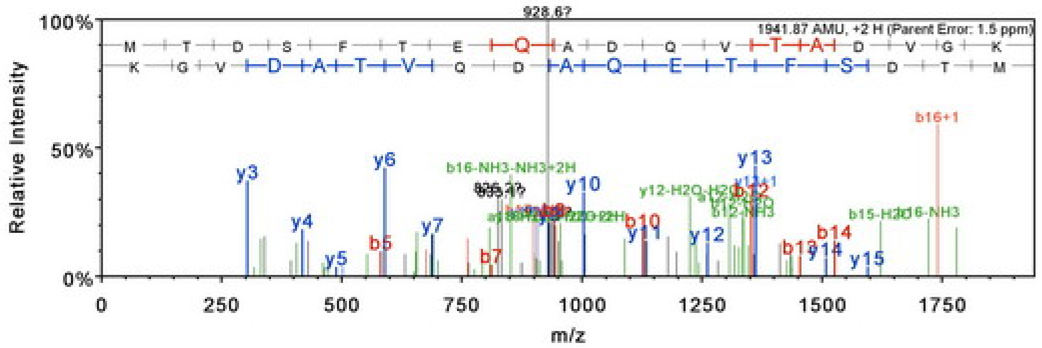

B	B Ions	B+2H	B-NH3	B-H2O	AA	Y Ions	Y+2H	Y-NH3	Y-H2O	Y
1	132.1				M	1942.9	971.9	1925.8	1924.9	18
2	233.1			215.1	T	1811.8	906.4	1794.8	1793.8	17
3	348.1			330.1	D	1710.8	855.9	1693.8	1692.8	16
4	435.2			417.1	S	1595.8	798.4	1578.7	1577.7	15
5	582.2			564.2	F	1508.7	754.9	1491.7	1490.7	14
6	683.3	342.1		665.3	T	1361.7	681.3	1344.6	1343.6	13
7	812.3	406.7		794.3	E	1260.6	630.8	1243.6	1242.6	12
8	940.4	470.7	923.4	922.4	Q	1131.6	566.3	1114.5	1113.5	11
9	1011.4	506.2	994.4	993.4	A	1003.5	502.3	986.5	985.5	10
10	1126.4	563.7	1109.4	1108.4	D	932.5	466.7	915.4	914.5	9
11	1254.5	627.8	1237.5	1236.5	Q	817.4	409.2	800.4	799.4	8
12	1353.6	677.3	1336.5	1335.5	V	689.4	345.2	672.4	671.4	7
13	1454.6	727.8	1437.6	1436.6	T	590.3	295.7	573.3	572.3	6
14	1525.7	763.3	1508.6	1507.6	A	489.3		472.2	471.3	5
15	1640.7	820.8	1623.7	1622.7	D	418.2		401.2	400.2	4
16	1739.7	870.4	1722.7	1721.7	V	303.2		286.2		3
17	1796.8	898.9	1779.7	1778.8	G	204.1		187.1		2
18	1942.9	971.9	1925.8	1924.9	K	147.1		130.1		1

Gene symbol: Rab1

Protein name: Ras-related protein Rab-1A

Protein accession numbers: IPI00114560

Peptide sequence: (K)EFADSLGIPFLETSAK(N)

Exclusive (unique to this protein): TRUE

SEQUEST XCorr score: 2.51 SEQUEST DCn score: 0.412

Fix modifications: None

Variable modifications: None

charge: 2 Observed m/z: 862.9375

Actual minus calculated peptide mass (AMU): -0.007813

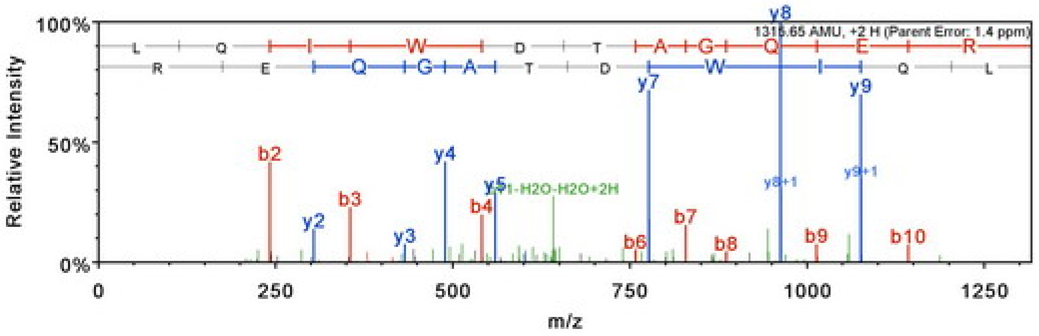

B	B Ions	B+2H	B-NH3	B-H2O	AA	Y Ions	Y+2H	Y-NH3	Y-H2O	Y
1	114.1				L	1316.7	658.8	1299.6	1298.7	11
2	242.2		225.1		Q	1203.6	602.3	1186.5	1185.6	10
3	355.2		338.2		I	1075.5	538.3	1058.5	1057.5	9
4	541.3		524.3		W	962.4	481.7	945.4	944.4	8
5	656.3		639.3	638.3	D	776.4	388.7	759.3	758.3	7
6	757.4	379.2	740.4	739.4	T	661.3	331.2	644.3	643.3	6
7	828.4	414.7	811.4	810.4	A	560.3		543.3	542.3	5
8	885.5	443.2	868.4	867.4	G	489.2		472.2	471.2	4
9	1013.5	507.3	996.5	995.5	Q	432.2		415.2	414.2	3
10	1142.5	571.8	1125.5	1124.5	E	304.2		287.1	286.2	2
11	1316.7	658.8	1299.6	1298.7	R	175.1		158.1		1

Gene symbol: Rab11b

Protein name: Ras-related protein Rab-11B

Protein accession numbers: IPI00135869,IPI00651918

Peptide sequence: (R)GAVGALLVYDIAK(H)

Exclusive (unique to this protein): TRUE

SEQUEST XCorr score: 2.17 SEQUEST DCn score: 0.584

Fix modifications: None

Variable modifications: None

charge: 2 Observed m/z: 645.3774

Actual minus calculated peptide mass (AMU): -0.0001221

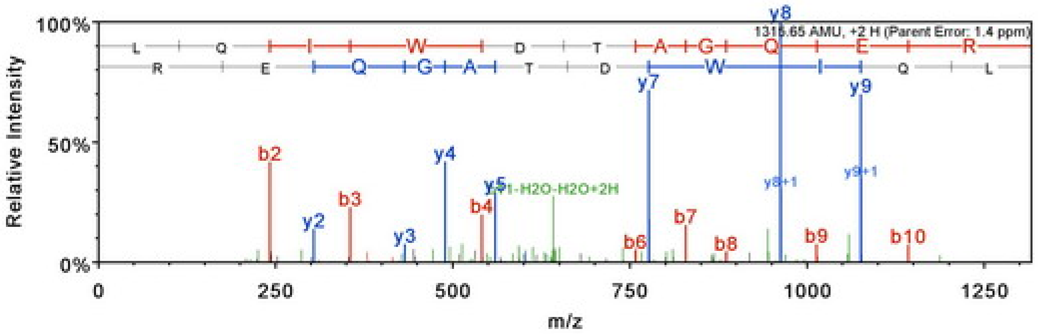

B	B Ions	B+2H	B-NH3	B-H2O	AA	Y Ions	Y+2H	Y-NH3	Y-H2O	Y
1	114.1				L	1316.7	658.8	1299.6	1298.7	11
2	242.2		225.1		Q	1203.6	602.3	1186.5	1185.6	10
3	355.2		338.2		I	1075.5	538.3	1058.5	1057.5	9
4	541.3		524.3		W	962.4	481.7	945.4	944.4	8
5	656.3		639.3	638.3	D	776.4	388.7	759.3	758.3	7
6	757.4	379.2	740.4	739.4	T	661.3	331.2	644.3	643.3	6
7	828.4	414.7	811.4	810.4	A	560.3		543.3	542.3	5
8	885.5	443.2	868.4	867.4	G	489.2		472.2	471.2	4
9	1013.5	507.3	996.5	995.5	Q	432.2		415.2	414.2	3
10	1142.5	571.8	1125.5	1124.5	E	304.2		287.1	286.2	2
11	1316.7	658.8	1299.6	1298.7	R	175.1		158.1		1

Gene symbol: Rab22a

Protein name: Ras-related protein Rab-22A
Protein accession numbers: IPI00116729,IPI00751807
Peptide sequence: (K)FLIWDTAGQER(F)
Exclusive (unique to this protein): TRUE
SEQUEST XCorr score: 2.02 SEQUEST DCn score: 0.41
Fix modifications: None
Variable modifications: None
charge: 2 Observed m/z: 668.3349
Actual minus calculated peptide mass (AMU): -0.007935

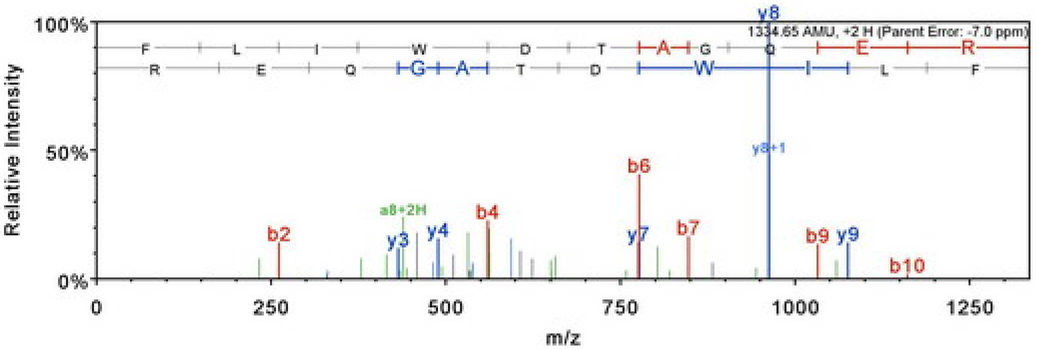

B	B Ions	B+2H	B-NH3	B-H2O	AA	Y Ions	Y+2H	Y-NH3	Y-H2O	Y
1	148.1				F	1335.7	668.3	1318.6	1317.7	11
2	261.2				L	1188.6	594.8	1171.6	1170.6	10
3	374.2				I	1075.5	538.3	1058.5	1057.5	9
4	560.3				W	962.4	481.7	945.4	944.4	8
5	675.4			657.3	D	776.4	388.7	759.3	758.3	7
6	776.4	388.7		758.4	T	661.3	331.2	644.3	643.3	6
7	847.4	424.2		829.4	A	560.3		543.3	542.3	5
8	904.5	452.7		886.5	G	489.2		472.2	471.2	4
9	1032.5	516.8	1015.5	1014.5	Q	432.2		415.2	414.2	3
10	1161.6	581.3	1144.5	1143.5	E	304.2		287.1	286.2	2
11	1335.7	668.3	1318.6	1317.7	R	175.1		158.1		1

Gene symbol: Rab27a
Protein name: Ras-related protein Rab-27A
Protein accession numbers: IPI00112374
Peptide sequence: (R)IHLQLWDTAGQER(F)
Exclusive (unique to this protein): TRUE
SEQUEST XCorr score: 2.05 SEQUEST DCn score: 0.579
Fix modifications: None
Variable modifications: None
charge: 2 Observed m/z: 783.9012
Actual minus calculated peptide mass (AMU): -0.008423

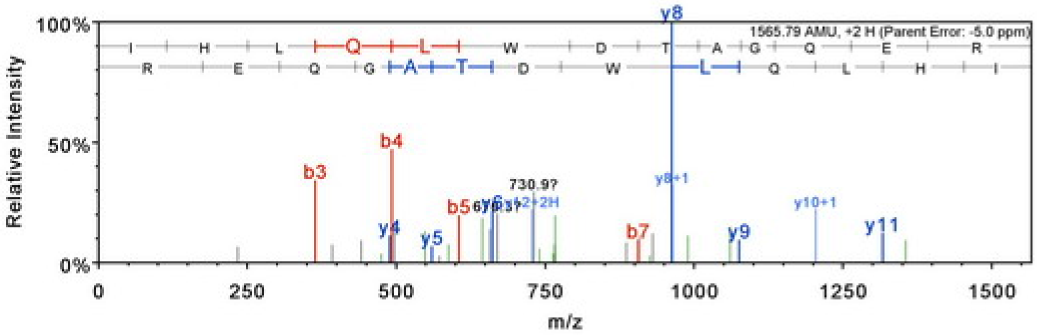

B	B Ions	B+2H	B-NH3	B-H2O	AA	Y Ions	Y+2H	Y-NH3	Y-H2O	Y
1	114.1				I	1566.8	783.9	1549.8	1548.8	13
2	251.2	126.1			H	1453.7	727.4	1436.7	1435.7	12
3	364.2	182.6			L	1316.7	658.8	1299.6	1298.7	11
4	492.3	246.7	475.3		Q	1203.6	602.3	1186.5	1185.6	10
5	605.4	303.2	588.4		L	1075.5	538.3	1058.5	1057.5	9
6	791.5	396.2	774.4		W	962.4	481.7	945.4	944.4	8
7	906.5	453.8	889.5	888.5	D	776.4	388.7	759.3	758.3	7
8	1007.5	504.3	990.5	989.5	T	661.3	331.2	644.3	643.3	6
9	1078.6	539.8	1061.5	1060.6	A	560.3		543.3	542.3	5
10	1135.6	568.3	1118.6	1117.6	G	489.2		472.2	471.2	4
11	1263.7	632.3	1246.6	1245.6	Q	432.2		415.2	414.2	3
12	1392.7	696.9	1375.7	1374.7	E	304.2		287.1	286.2	2
13	1566.8	783.9	1549.8	1548.8	R	175.1		158.1		1

Gene symbol: Rab38

Protein name: Ras-related protein Rab-38

Protein accession numbers: IPI00154087

Peptide sequence: (R)LQLWDIAGQER(F)

Exclusive (unique to this protein): TRUE

SEQUEST XCorr score: 2.45 SEQUEST DCn score: 0.636

Fix modifications: None

Variable modifications: None

charge: 2 Observed m/z: 664.8478

Actual minus calculated peptide mass (AMU): -0.008789

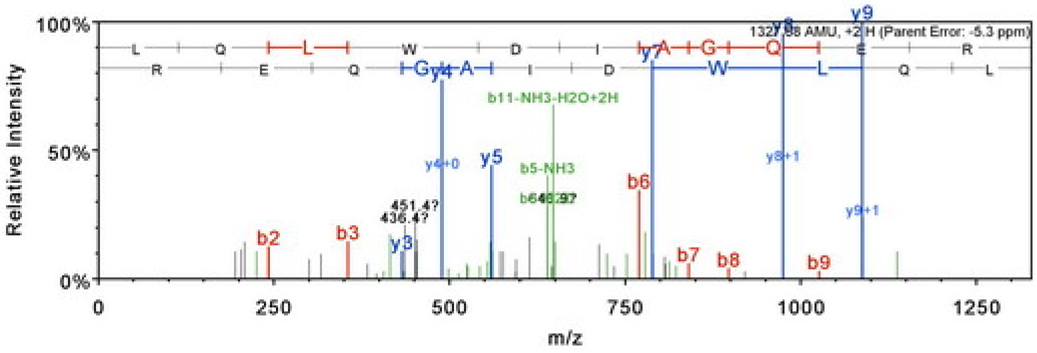

B	B Ions	B+2H	B-NH3	B-H2O	AA	Y Ions	Y+2H	Y-NH3	Y-H2O	Y
1	114.1				L	1328.7	664.9	1311.7	1310.7	11
2	242.2		225.1		Q	1215.6	608.3	1198.6	1197.6	10
3	355.2		338.2		L	1087.5	544.3	1070.5	1069.5	9
4	541.3		524.3		W	974.5	487.7	957.4	956.5	8
5	656.3		639.3	638.3	D	788.4	394.7	771.4	770.4	7
6	769.4	385.2	752.4	751.4	I	673.4	337.2	656.3	655.4	6
7	840.5	420.7	823.4	822.5	A	560.3		543.3	542.3	5
8	897.5	449.3	880.5	879.5	G	489.2		472.2	471.2	4
9	1025.5	513.3	1008.5	1007.5	Q	432.2		415.2	414.2	3
10	1154.6	577.8	1137.6	1136.6	E	304.2		287.1	286.2	2
11	1328.7	664.9	1311.7	1310.7	R	175.1		158.1		1

Gene symbol: Rab5a

Protein name: Ras-related protein Rab-5A

Protein accession numbers: IPI00132410

Peptide sequence: (K)FEIWDTAGQER(Y)

Exclusive (unique to this protein): TRUE

SEQUEST XCorr score: 2.1 SEQUEST DCn score: 0.437

Fix modifications: None

Variable modifications: None

charge: 2 Observed m/z: 676.3168

Actual minus calculated peptide mass (AMU): -0.002564

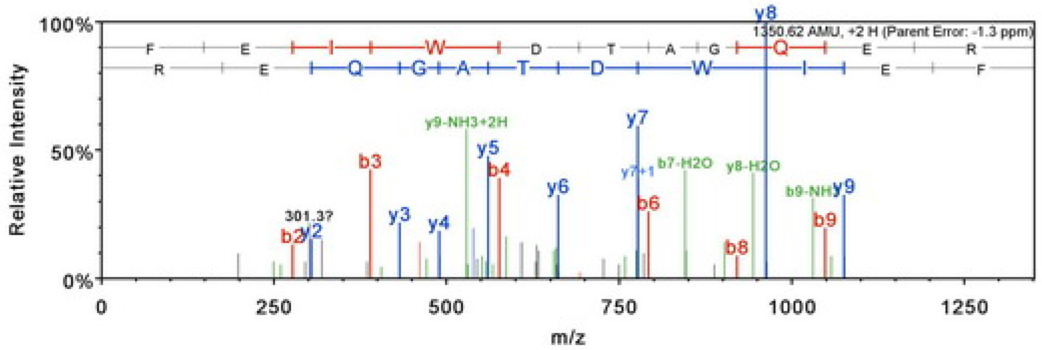

B	B Ions	B+2H	B-NH3	B-H2O	AA	Y Ions	Y+2H	Y-NH3	Y-H2O	Y
1	148.1				F	1351.6	676.3	1334.6	1333.6	11
2	277.1			259.1	E	1204.6	602.8	1187.5	1186.5	10
3	390.2			372.2	I	1075.5	538.3	1058.5	1057.5	9
4	576.3			558.3	W	962.4	481.7	945.4	944.4	8
5	691.3			673.3	D	776.4	388.7	759.3	758.3	7
6	792.4	396.7		774.4	T	661.3	331.2	644.3	643.3	6
7	863.4	432.2		845.4	A	560.3		543.3	542.3	5
8	920.4	460.7		902.4	G	489.2		472.2	471.2	4
9	1048.5	524.7	1031.5	1030.5	Q	432.2		415.2	414.2	3
10	1177.5	589.3	1160.5	1159.5	E	304.2		287.1	286.2	2
11	1351.6	676.3	1334.6	1333.6	R	175.1		158.1		1

Gene symbol: Rab5b

Protein name: Rab5B

Protein accession numbers: IPI00116563,IPI00458976,IPI00849548

Peptide sequence: (R)GAQAAIVVYDITNQETFAR(A)

Exclusive (unique to this protein): TRUE

SEQUEST XCorr score: 2.5 SEQUEST DCn score: 0.638

Fix modifications: None

Variable modifications: None

charge: 2 Observed m/z: 1034.0258

Actual minus calculated peptide mass (AMU): -0.007324

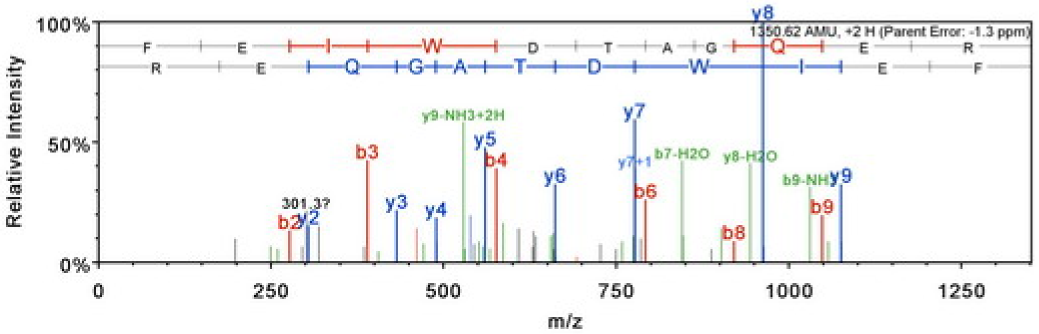

B	B Ions	B+2H	B-NH3	B-H2O	AA	Y Ions	Y+2H	Y-NH3	Y-H2O	Y
1	148.1				F	1351.6	676.3	1334.6	1333.6	11
2	277.1			259.1	E	1204.6	602.8	1187.5	1186.5	10
3	390.2			372.2	I	1075.5	538.3	1058.5	1057.5	9
4	576.3			558.3	W	962.4	481.7	945.4	944.4	8
5	691.3			673.3	D	776.4	388.7	759.3	758.3	7
6	792.4	396.7		774.4	T	661.3	331.2	644.3	643.3	6
7	863.4	432.2		845.4	A	560.3		543.3	542.3	5
8	920.4	460.7		902.4	G	489.2		472.2	471.2	4
9	1048.5	524.7	1031.5	1030.5	Q	432.2		415.2	414.2	3
10	1177.5	589.3	1160.5	1159.5	E	304.2		287.1	286.2	2
11	1351.6	676.3	1334.6	1333.6	R	175.1		158.1		1

Gene symbol: Rac1

Protein name: Mammary gland RCB-0527 Jyg-MC(B) cDNA, RIKEN full-length enriched library, clone:G930005L08 product:RAS-related C3 botulinum substrate 1, full insert sequence

Protein accession numbers: IPI00127408,IPI00761613

Peptide sequence: (K)PVNLGLWDTAGQEDYDR(L)

Exclusive (unique to this protein): TRUE

SEQUEST XCorr score: 2.68 SEQUEST DCn score: 0.796

Fix modifications: None

Variable modifications: None

charge: 2 Observed m/z: 974.9591

Actual minus calculated peptide mass (AMU): 0.006104

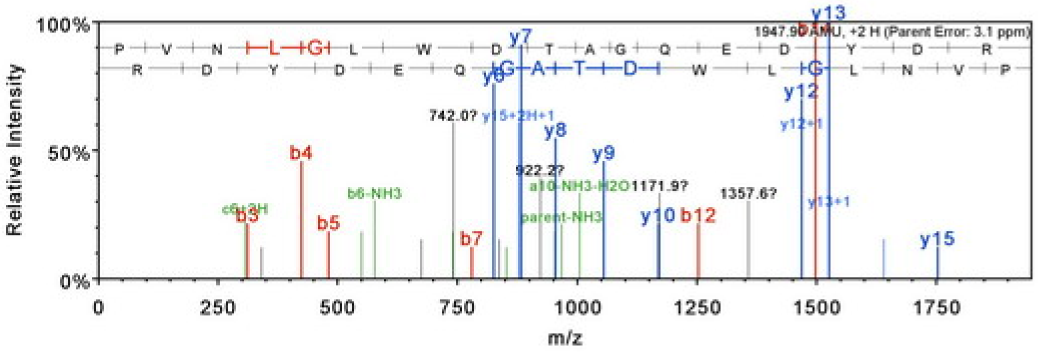

B	B Ions	B+2H	B-NH3	B-H2O	AA	Y Ions	Y+2H	Y-NH3	Y-H2O	Y
1	98.1				P	1948.9	975.0	1931.9	1930.9	17
2	197.1				V	1851.9	926.4	1834.8	1833.8	16
3	311.2		294.2		N	1752.8	876.9	1735.8	1734.8	15
4	424.3		407.2		L	1638.7	819.9	1621.7	1620.7	14
5	481.3		464.3		G	1525.7	763.3	1508.6	1507.7	13
6	594.4	297.7	577.3		L	1468.6	734.8	1451.6	1450.6	12
7	780.4	390.7	763.4		W	1355.5	678.3	1338.5	1337.5	11
8	895.5	448.2	878.4	877.5	D	1169.5	585.2	1152.4	1151.5	10
9	996.5	498.8	979.5	978.5	T	1054.4	527.7	1037.4	1036.4	9
10	1067.5	534.3	1050.5	1049.5	A	953.4	477.2	936.4	935.4	8
11	1124.6	562.8	1107.5	1106.6	G	882.4	441.7	865.3	864.4	7
12	1252.6	626.8	1235.6	1234.6	Q	825.3	413.2	808.3	807.3	6
13	1381.7	691.3	1364.7	1363.7	E	697.3		680.3	679.3	5
14	1496.7	748.9	1479.7	1478.7	D	568.2		551.2	550.2	4
15	1659.8	830.4	1642.7	1641.8	Y	453.2		436.2	435.2	3
16	1774.8	887.9	1757.8	1756.8	D	290.2		273.1	272.1	2
17	1948.9	975.0	1931.9	1930.9	R	175.1		158.1		1

Gene symbol: Rad51

Protein name: DNA repair protein RAD51 homolog 1

Protein accession numbers: IPI00118524,IPI00750989,IPI00756623

Peptide sequence: (R)YGLSGSDVLDNVAYAR(G)

Exclusive (unique to this protein): TRUE

SEQUEST XCorr score: 2.28 SEQUEST DCn score: 0.634

Fix modifications: None

Variable modifications: None

charge: 2 Observed m/z: 850.4151

Actual minus calculated peptide mass (AMU): -0.006592

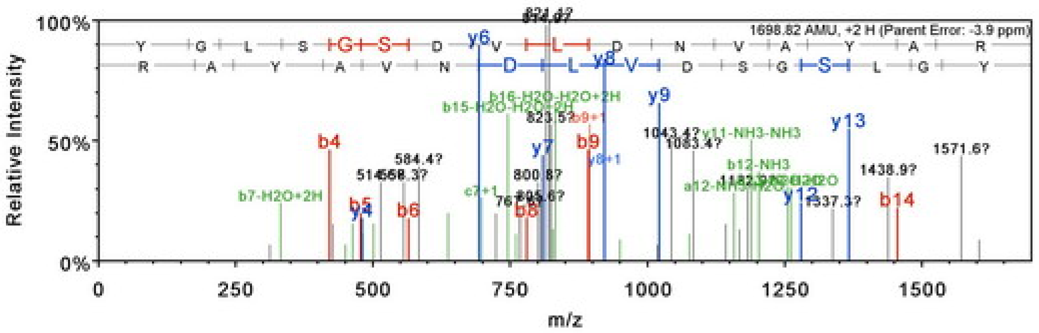

B	B Ions	B+2H	B-NH3	B-H2O	AA	Y Ions	Y+2H	Y-NH3	Y-H2O	Y
1	164.1				Y	1699.8	850.4	1682.8	1681.8	16
2	221.1				G	1536.8	768.9	1519.7	1518.8	15
3	334.2				L	1479.7	740.4	1462.7	1461.7	14
4	421.2			403.2	S	1366.7	683.8	1349.6	1348.7	13
5	478.2			460.2	G	1279.6	640.3	1262.6	1261.6	12
6	565.3	283.1		547.3	S	1222.6	611.8	1205.6	1204.6	11
7	680.3	340.7		662.3	D	1135.6	568.3	1118.5	1117.6	10
8	779.4	390.2		761.4	V	1020.6	510.8	1003.5	1002.5	9
9	892.4	446.7		874.4	L	921.5	461.2	904.5	903.5	8
10	1007.5	504.2		989.5	D	808.4	404.7	791.4	790.4	7
11	1121.5	561.3	1104.5	1103.5	N	693.4	347.2	676.3		6
12	1220.6	610.8	1203.5	1202.6	V	579.3		562.3		5
13	1291.6	646.3	1274.6	1273.6	A	480.3		463.2		4
14	1454.7	727.8	1437.7	1436.7	Y	409.2		392.2		3
15	1525.7	763.4	1508.7	1507.7	A	246.2		229.1		2
16	1699.8	850.4	1682.8	1681.8	R	175.1		158.1		1

Gene symbol: Ranbp5

Protein name: Isoform 2 of Importin beta-3

Protein accession numbers: IPI00409936,IPI00626994

Peptide sequence: (R)ATAAFILANEHNVALFK(H)

Exclusive (unique to this protein): TRUE

SEQUEST XCorr score: 2.29 SEQUEST DCn score: 0.526

Fix modifications: None

Variable modifications: None

charge: 2 Observed m/z: 915.4974

Actual minus calculated peptide mass (AMU): -0.004395

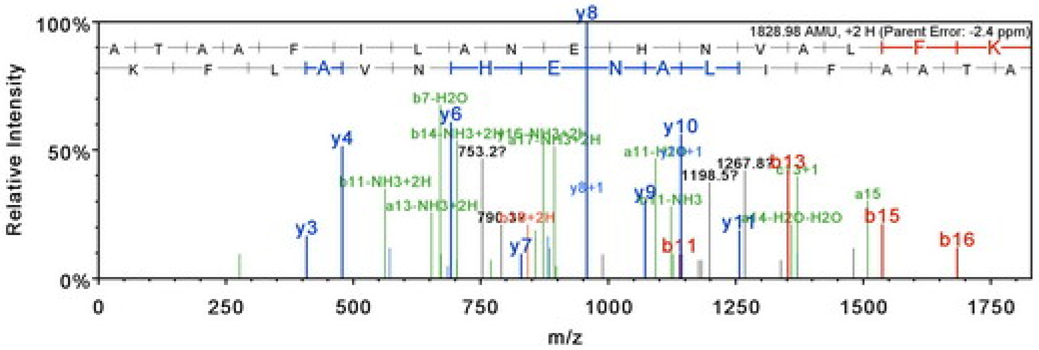

B	B Ions	B+2H	B-NH3	B-H2O	AA	Y Ions	Y+2H	Y-NH3	Y-H2O	Y
1	72.0				A	1830.0	915.5	1813.0	1812.0	17
2	173.1			155.1	T	1759.0	880.0	1741.9	1740.9	16
3	244.1			226.1	A	1657.9	829.5	1640.9	1639.9	15
4	315.2			297.2	A	1586.9	793.9	1569.8	1568.9	14
5	462.2			444.2	F	1515.8	758.4	1498.8	1497.8	13
6	575.3	288.2		557.3	I	1368.8	684.9	1351.7	1350.8	12
7	688.4	344.7		670.4	L	1255.7	628.3	1238.7	1237.7	11
8	759.4	380.2		741.4	A	1142.6	571.8	1125.6	1124.6	10
9	873.5	437.3	856.5	855.5	N	1071.6	536.3	1054.5	1053.5	9
10	1002.5	501.8	985.5	984.5	E	957.5	479.3	940.5	939.5	8
11	1139.6	570.3	1122.6	1121.6	H	828.5	414.7	811.5		7
12	1253.6	627.3	1236.6	1235.6	N	691.4	346.2	674.4		6
13	1352.7	676.9	1335.7	1334.7	V	577.4		560.3		5
14	1423.7	712.4	1406.7	1405.7	A	478.3		461.3		4
15	1536.8	768.9	1519.8	1518.8	L	407.3		390.2		3
16	1683.9	842.5	1666.9	1665.9	F	294.2		277.2		2
17	1830.0	915.5	1813.0	1812.0	K	147.1		130.1		1

Gene symbol: Rbbp5

Protein name: NOD-derived CD11c +ve dendritic cells cDNA, RIKEN full-length enriched library, clone:F630106K17 product:retinoblastoma binding protein 5, full insert sequence

Protein accession numbers: IPI00226384

Peptide sequence: (K)GSCFLINTADRIIRVYDGR(E)

Exclusive (unique to this protein): TRUE

SEQUEST XCorr score: 2.55   SEQUEST DCn score: 0.364

Fix modifications: C3: Carbamidomethyl (+57.02)Variable modifications: None

charge: 2   Observed m/z: 1113.5671

Actual minus calculated peptide mass (AMU): -0.019052

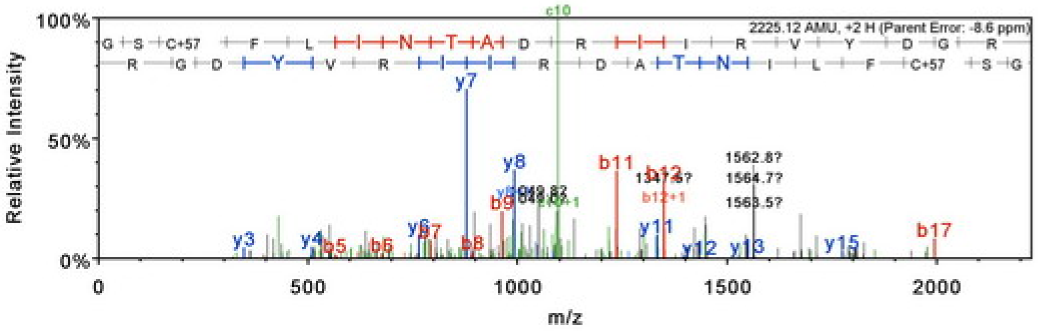

B	B Ions	B+2H	B-NH3	B-H2O	AA	Y Ions	Y+2H	Y-NH3	Y-H2O	Y
1	58.0				G	2226.2	1113.6	2209.1	2208.1	19
2	145.1			127.1	S	2169.1	1085.1	2152.1	2151.1	18
3	305.1			287.1	C+57	2082.1	1041.5	2065.1	2064.1	17
4	452.2			434.2	F	1922.1	961.5	1905.0	1904.1	16
5	565.2			547.2	L	1775.0	888.0	1758.0	1757.0	15
6	678.3	339.7		660.3	I	1661.9	831.5	1644.9	1643.9	14
7	792.4	396.7	775.3	774.4	N	1548.8	774.9	1531.8	1530.8	13
8	893.4	447.2	876.4	875.4	T	1434.8	717.9	1417.8	1416.8	12
9	964.5	482.7	947.4	946.5	A	1333.7	667.4	1316.7	1315.7	11
10	1079.5	540.3	1062.5	1061.5	D	1262.7	631.9	1245.7	1244.7	10
11	1235.6	618.3	1218.6	1217.6	R	1147.7	574.3	1130.6	1129.7	9
12	1348.7	674.8	1331.6	1330.7	I	991.6	496.3	974.5	973.6	8
13	1461.8	731.4	1444.7	1443.7	I	878.5	439.8	861.5	860.5	7
14	1617.9	809.4	1600.8	1599.8	R	765.4	383.2	748.4	747.4	6
15	1716.9	859.0	1699.9	1698.9	V	609.3		592.3	591.3	5
16	1880.0	940.5	1863.0	1862.0	Y	510.2		493.2	492.2	4
17	1995.0	998.0	1978.0	1977.0	D	347.2		330.1	329.2	3
18	2052.0	1026.5	2035.0	2034.0	G	232.1		215.1		2
19	2226.2	1113.6	2209.1	2208.1	R	175.1		158.1		1

Gene symbol: Rbpms2

Protein name: RNA-binding protein with multiple splicing 2

Protein accession numbers: IPI00121434,IPI00461350

Peptide sequence: (R)TLFVSGLPVDIKPR(E)

Exclusive (unique to this protein): TRUE

SEQUEST XCorr score: 2.2 SEQUEST DCn score: 0.735

Fix modifications: None

Variable modifications: None

charge: 2 Observed m/z: 771.4526

Actual minus calculated peptide mass (AMU): -0.0085453

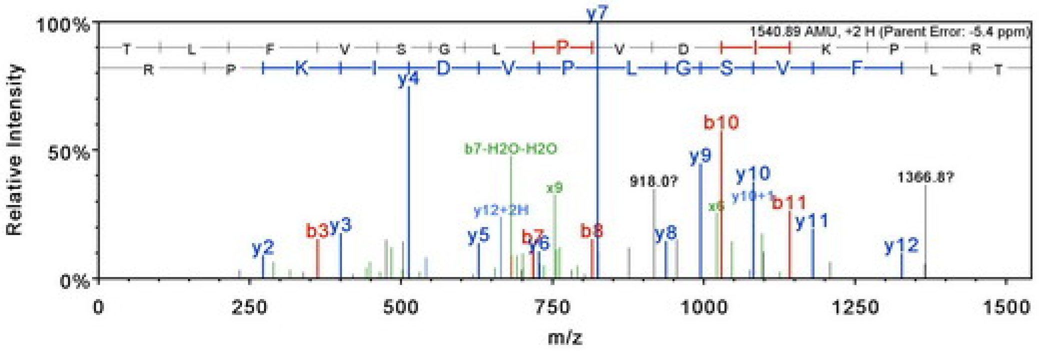

B	B Ions	B+2H	B-NH3	B-H2O	AA	Y Ions	Y+2H	Y-NH3	Y-H2O	Y
1	102.1			84.0	T	1541.9	771.5	1524.9	1523.9	14
2	215.1			197.1	L	1440.9	720.9	1423.8	1422.9	13
3	362.2			344.2	F	1327.8	664.4	1310.8	1309.8	12
4	461.3			443.3	V	1180.7	590.9	1163.7	1162.7	11
5	548.3			530.3	S	1081.6	541.3	1064.6	1063.6	10
6	605.3	303.2		587.3	G	994.6	497.8	977.6	976.6	9
7	718.4	359.7		700.4	L	937.6	469.3	920.6	919.6	8
8	815.5	408.2		797.5	P	824.5	412.8	807.5	806.5	7
9	914.5	457.8		896.5	V	727.5	364.2	710.4	709.4	6
10	1029.6	515.3		1011.6	D	628.4	314.7	611.4	610.4	5
11	1142.7	571.8		1124.6	I	513.4	257.2	496.3		4
12	1270.7	635.9	1253.7	1252.7	K	400.3	200.6	383.2		3
13	1367.8	684.4	1350.8	1349.8	P	272.2		255.2		2
14	1541.9	771.5	1524.9	1523.9	R	175.1		158.1		1

Gene symbol: Rbx1

Protein name: RING-box protein 1

Protein accession numbers: IPI00124752,IPI00849056,IPI00850857

Peptide sequence: (R)QVCPLDNREWEFQK(Y)

Exclusive (unique to this protein): TRUE

SEQUEST XCorr score: 2.01 SEQUEST DCn score: 0.335

Fix modifications: C3: Carbamidomethyl (+57.02)Variable modifications: None

charge: 2 Observed m/z: 924.9404

Actual minus calculated peptide mass (AMU): 0.002564

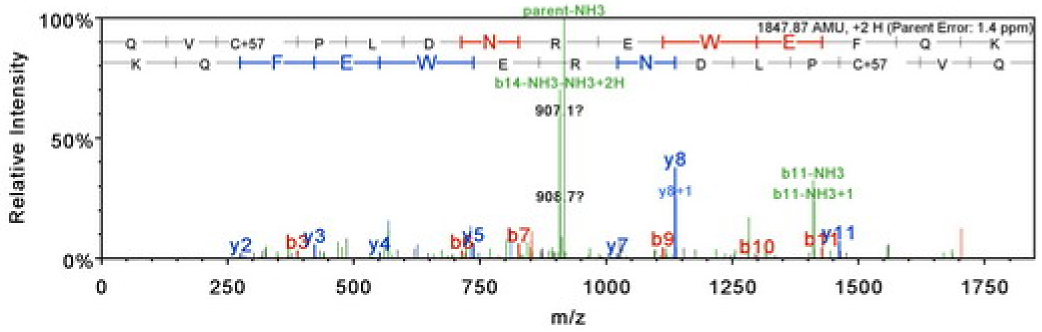

B	B Ions	B+2H	B-NH3	B-H2O	AA	Y Ions	Y+2H	Y-NH3	Y-H2O	Y
1	129.1		112.0		Q	1848.9	924.9	1831.8	1830.9	14
2	228.1		211.1		V	1720.8	860.9	1703.8	1702.8	13
3	388.2		371.1		C+57	1621.7	811.4	1604.7	1603.7	12
4	485.2		468.2		P	1461.7	731.4	1444.7	1443.7	11
5	598.3		581.3		L	1364.7	682.8	1347.6	1346.7	10
6	713.3	357.2	696.3	695.3	D	1251.6	626.3	1234.5	1233.6	9
7	827.4	414.2	810.4	809.4	N	1136.5	568.8	1119.5	1118.5	8
8	983.5	492.2	966.5	965.5	R	1022.5	511.8	1005.5	1004.5	7
9	1112.5	556.8	1095.5	1094.5	E	866.4	433.7	849.4	848.4	6
10	1298.6	649.8	1281.6	1280.6	W	737.4		720.3	719.4	5
11	1427.6	714.3	1410.6	1409.6	E	551.3		534.3	533.3	4
12	1574.7	787.9	1557.7	1556.7	F	422.2		405.2		3
13	1702.8	851.9	1685.7	1684.8	Q	275.2		258.1		2
14	1848.9	924.9	1831.8	1830.9	K	147.1		130.1		1

Gene symbol: Rdh11
Protein name: Retinol dehydrogenase 11
Protein accession numbers: IPI00136098
Peptide sequence: (R)EIQAVTGNSQVFVR(K)
Exclusive (unique to this protein): TRUE
SEQUEST XCorr score: 2.71   SEQUEST DCn score: 0.69
Fix modifications: None
Variable modifications: None
charge: 2   Observed m/z: 774.4144
Actual minus calculated peptide mass (AMU): 0.00293

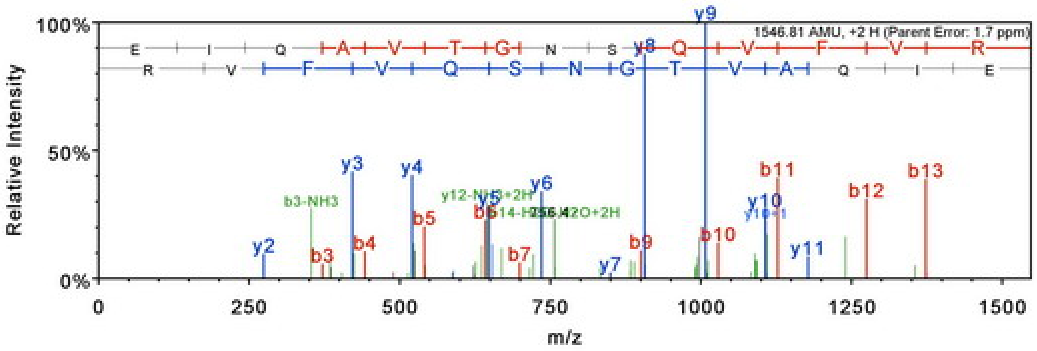

B	B Ions	B+2H	B-NH3	B-H2O	AA	Y Ions	Y+2H	Y-NH3	Y-H2O	Y
1	130.1			112.0	E	1547.8	774.4	1530.8	1529.8	14
2	243.1			225.1	I	1418.8	709.9	1401.8	1400.8	13
3	371.2		354.2	353.2	Q	1305.7	653.4	1288.7	1287.7	12
4	442.2		425.2	424.2	A	1177.6	589.3	1160.6	1159.6	11
5	541.3		524.3	523.3	V	1106.6	553.8	1089.6	1088.6	10
6	642.4	321.7	625.3	624.3	T	1007.5	504.3	990.5	989.5	9
7	699.4	350.2	682.3	681.4	G	906.5	453.7	889.5	888.5	8
8	813.4	407.2	796.4	795.4	N	849.5	425.2	832.4	831.5	7
9	900.4	450.7	883.4	882.4	S	735.4	368.2	718.4	717.4	6
10	1028.5	514.8	1011.5	1010.5	Q	648.4		631.4		5
11	1127.6	564.3	1110.5	1109.6	V	520.3		503.3		4
12	1274.6	637.8	1257.6	1256.6	F	421.3		404.2		3
13	1373.7	687.4	1356.7	1355.7	V	274.2		257.2		2
14	1547.8	774.4	1530.8	1529.8	R	175.1		158.1		1

Gene symbol: Rnf141
Protein name: NOD-derived CD11c +ve dendritic cells cDNA, RIKEN full-length enriched library, clone:F630030D05 product:ring finger protein 141, full insert sequence
Protein accession numbers: IPI00454038
Peptide sequence: (R)ESGSLTYEEFLGR(V)
Exclusive (unique to this protein): TRUE
SEQUEST XCorr score: 2.14   SEQUEST DCn score: 0.582
Fix modifications: None

Variable modifications: None

charge: 2 Observed m/z: 744.3551

Actual minus calculated peptide mass (AMU): 0.0004883

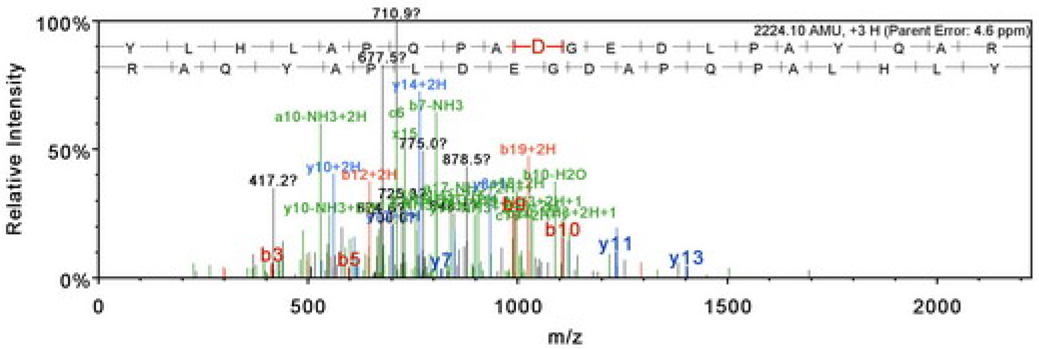

B	B Ions	B+2H	B-NH3	B-H2O	AA	Y Ions	Y+2H	Y-NH3	Y-H2O	Y
1	164.1				Y	2225.1	1113.0	2208.1	2207.1	20
2	277.2				L	2062.0	1031.5	2045.0	2044.0	19
3	414.2	207.6			H	1949.0	975.0	1931.9	1930.9	18
4	527.3	264.1			L	1811.9	906.5	1794.9	1793.9	17
5	598.3	299.7			A	1698.8	849.9	1681.8	1680.8	16
6	695.4	348.2			P	1627.8	814.4	1610.8	1609.8	15
7	823.5	412.2	806.4		Q	1530.7	765.9	1513.7	1512.7	14
8	920.5	460.8	903.5		P	1402.7	701.8	1385.6	1384.7	13
9	991.5	496.3	974.5		A	1305.6	653.3	1288.6	1287.6	12
10	1106.6	553.8	1089.5	1088.5	D	1234.6	617.8	1217.5	1216.6	11
11	1163.6	582.3	1146.6	1145.6	G	1119.5	560.3	1102.5	1101.5	10
12	1292.6	646.8	1275.6	1274.6	E	1062.5	531.8	1045.5	1044.5	9
13	1407.7	704.3	1390.6	1389.6	D	933.5	467.2	916.5	915.5	8
14	1520.7	760.9	1503.7	1502.7	L	818.5	409.7	801.4		7
15	1617.8	809.4	1600.8	1599.8	P	705.4	353.2	688.3		6
16	1688.8	844.9	1671.8	1670.8	A	608.3		591.3		5
17	1851.9	926.5	1834.9	1833.9	Y	537.3		520.3		4
18	1980.0	990.5	1962.9	1961.9	Q	374.2		357.2		3
19	2051.0	1026.0	2034.0	2033.0	A	246.2		229.1		2
20	2225.1	1113.0	2208.1	2207.1	R	175.1		158.1		1

Gene symbol: Rnf31

Protein name: Muscle protein 534 (Fragment)

Protein accession numbers: IPI00461570,IPI00468517,IPI00474602,IPI00762546,IPI00762888

Peptide sequence: (R)YLHLAPQPADGEDLPAYQAR(L)

Exclusive (unique to this protein): TRUE

SEQUEST XCorr score: 2.57 SEQUEST DCn score: 0.22

Fix modifications: None

Variable modifications: None

charge: 3 Observed m/z: 742.3751

Actual minus calculated peptide mass (AMU): 0.010253

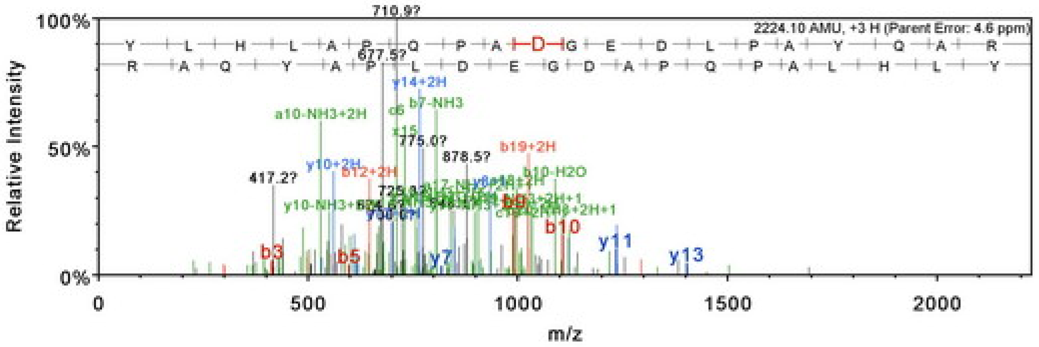

B	B Ions	B+2H	B-NH3	B-H2O	AA	Y Ions	Y+2H	Y-NH3	Y-H2O	Y
1	164.1				Y	2225.1	1113.0	2208.1	2207.1	20
2	277.2				L	2062.0	1031.5	2045.0	2044.0	19
3	414.2	207.6			H	1949.0	975.0	1931.9	1930.9	18
4	527.3	264.1			L	1811.9	906.5	1794.9	1793.9	17
5	598.3	299.7			A	1698.8	849.9	1681.8	1680.8	16
6	695.4	348.2			P	1627.8	814.4	1610.8	1609.8	15
7	823.5	412.2	806.4		Q	1530.7	765.9	1513.7	1512.7	14
8	920.5	460.8	903.5		P	1402.7	701.8	1385.6	1384.7	13
9	991.5	496.3	974.5		A	1305.6	653.3	1288.6	1287.6	12
10	1106.6	553.8	1089.5	1088.5	D	1234.6	617.8	1217.5	1216.6	11
11	1163.6	582.3	1146.6	1145.6	G	1119.5	560.3	1102.5	1101.5	10
12	1292.6	646.8	1275.6	1274.6	E	1062.5	531.8	1045.5	1044.5	9
13	1407.7	704.3	1390.6	1389.6	D	933.5	467.2	916.5	915.5	8
14	1520.7	760.9	1503.7	1502.7	L	818.5	409.7	801.4		7
15	1617.8	809.4	1600.8	1599.8	P	705.4	353.2	688.3		6
16	1688.8	844.9	1671.8	1670.8	A	608.3		591.3		5
17	1851.9	926.5	1834.9	1833.9	Y	537.3		520.3		4
18	1980.0	990.5	1962.9	1961.9	Q	374.2		357.2		3
19	2051.0	1026.0	2034.0	2033.0	A	246.2		229.1		2
20	2225.1	1113.0	2208.1	2207.1	R	175.1		158.1		1

Gene symbol: Rpa3

Protein name: Replication protein A 14 kDa subunit

Protein accession numbers: IPI00132128

Peptide sequence: (K)IINELPQFFPVGLPQHE

Exclusive (unique to this protein): TRUE

SEQUEST XCorr score: 2.11 SEQUEST DCn score: 0.655

Fix modifications: None

Variable modifications: None

charge: 2 Observed m/z: 989.5241

Actual minus calculated peptide mass (AMU): -0.003784

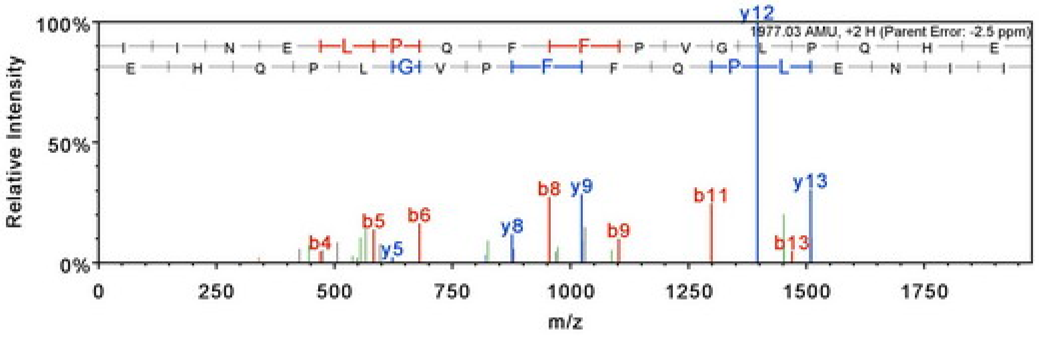

B	B Ions	B+2H	B-NH3	B-H2O	AA	Y Ions	Y+2H	Y-NH3	Y-H2O	Y
1	114.1				I	1978.0	989.5	1961.0	1960.0	17
2	227.2				I	1865.0	933.0	1847.9	1847.0	16
3	341.2		324.2		N	1751.9	876.4	1734.9	1733.9	15
4	470.3		453.2	452.3	E	1637.8	819.4	1620.8	1619.8	14
5	583.4		566.3	565.3	L	1508.8	754.9	1491.8	1490.8	13
6	680.4	340.7	663.4	662.4	P	1395.7	698.4	1378.7	1377.7	12
7	808.5	404.7	791.4	790.5	Q	1298.7	649.8	1281.6	1280.6	11
8	955.5	478.3	938.5	937.5	F	1170.6	585.8	1153.6	1152.6	10
9	1102.6	551.8	1085.6	1084.6	F	1023.5	512.3	1006.5	1005.5	9
10	1199.7	600.3	1182.6	1181.6	P	876.5	438.7	859.4	858.5	8
11	1298.7	649.9	1281.7	1280.7	V	779.4	390.2	762.4	761.4	7
12	1355.7	678.4	1338.7	1337.7	G	680.3	340.7	663.3	662.3	6
13	1468.8	734.9	1451.8	1450.8	L	623.3		606.3	605.3	5
14	1565.9	783.4	1548.9	1547.9	P	510.2		493.2	492.2	4
15	1693.9	847.5	1676.9	1675.9	Q	413.2		396.2	395.2	3
16	1831.0	916.0	1814.0	1813.0	H	285.1			267.1	2
17	1978.0	989.5	1961.0	1960.0	E	148.1			130.1	1

Gene symbol: Rpe

Protein name: Unknown protein (Fragment)

Protein accession numbers: IPI00459757

Peptide sequence: (K)IGPSILNSDLANLGAECLR(M)

Exclusive (unique to this protein): TRUE

SEQUEST XCorr score: 2.42 SEQUEST DCn score: 0.605

Fix modifications: C17: Carbamidomethyl (+57.02)Variable modifications: None

charge: 2 Observed m/z: 1007.0226

Actual minus calculated peptide mass (AMU): -0.0067152

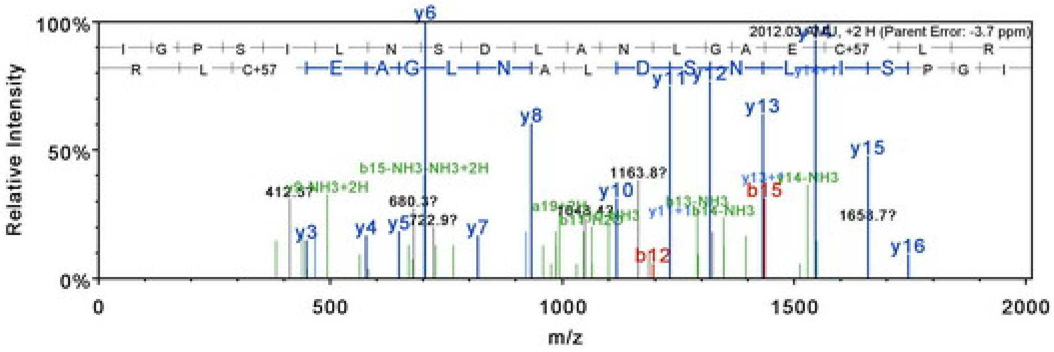

B	B Ions	B+2H	B-NH3	B-H2O	AA	Y Ions	Y+2H	Y-NH3	Y-H2O	Y
1	114.1				I	2013.0	1007.0	1996.0	1995.0	19
2	171.1				G	1900.0	950.5	1882.9	1882.0	18
3	268.2				P	1842.9	922.0	1825.9	1824.9	17
4	355.2			337.2	S	1745.9	873.5	1728.9	1727.9	16
5	468.3			450.3	I	1658.9	829.9	1641.8	1640.8	15
6	581.4	291.2		563.4	L	1545.8	773.4	1528.7	1527.8	14
7	695.4	348.2	678.4	677.4	N	1432.7	716.9	1415.7	1414.7	13
8	782.4	391.7	765.4	764.4	S	1318.6	659.8	1301.6	1300.6	12
9	897.5	449.2	880.4	879.5	D	1231.6	616.3	1214.6	1213.6	11
10	1010.6	505.8	993.5	992.5	L	1116.6	558.8	1099.6	1098.6	10
11	1081.6	541.3	1064.6	1063.6	A	1003.5	502.3	986.5	985.5	9
12	1195.6	598.3	1178.6	1177.6	N	932.5	466.7	915.4	914.5	8
13	1308.7	654.9	1291.7	1290.7	L	818.4	409.7	801.4	800.4	7
14	1365.7	683.4	1348.7	1347.7	G	705.3	353.2	688.3	687.3	6
15	1436.8	718.9	1419.8	1418.8	A	648.3		631.3	630.3	5
16	1565.8	783.4	1548.8	1547.8	E	577.3		560.3	559.3	4
17	1725.9	863.4	1708.8	1707.8	C+57	448.2		431.2		3
18	1838.9	920.0	1821.9	1820.9	L	288.2		271.2		2
19	2013.0	1007.0	1996.0	1995.0	R	175.1		158.1		1

Gene symbol: Rpl11

Protein name: 60S ribosomal protein L11

Protein accession numbers: IPI00331461,IPI00340036,IPI00474856,IPI00624735,IPI00626239

Peptide sequence: (K)VLEQLTGQTPVFSK(A)

Exclusive (unique to this protein): TRUE

SEQUEST XCorr score: 2.17 SEQUEST DCn score: 0.658

Fix modifications: None

Variable modifications: None

charge: 2 Observed m/z: 773.9287

Actual minus calculated peptide mass (AMU): 0.001343

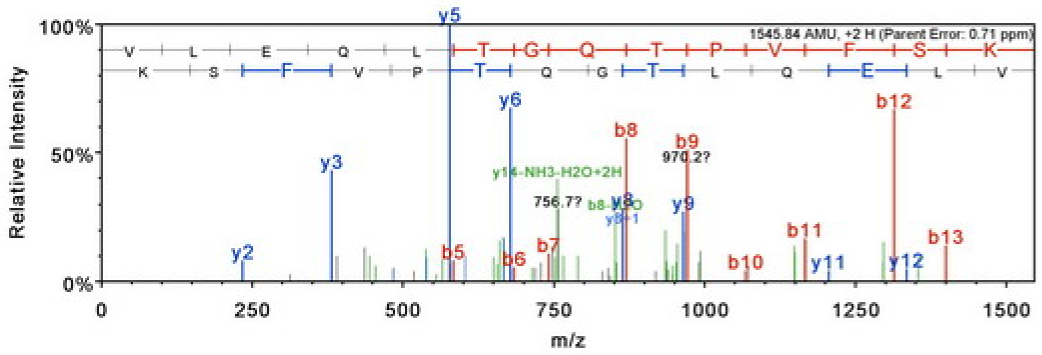

B	B Ions	B+2H	B-NH3	B-H2O	AA	Y Ions	Y+2H	Y-NH3	Y-H2O	Y
1	100.1				V	1546.9	773.9	1529.8	1528.8	14
2	213.2				L	1447.8	724.4	1430.8	1429.8	13
3	342.2			324.2	E	1334.7	667.9	1317.7	1316.7	12
4	470.3		453.2	452.3	Q	1205.7	603.3	1188.6	1187.6	11
5	583.4		566.3	565.3	L	1077.6	539.3	1060.6	1059.6	10
6	684.4	342.7	667.4	666.4	T	964.5	482.8	947.5	946.5	9
7	741.4	371.2	724.4	723.4	G	863.5	432.2	846.4	845.5	8
8	869.5	435.2	852.5	851.5	Q	806.4	403.7	789.4	788.4	7
9	970.5	485.8	953.5	952.5	T	678.4	339.7	661.4	660.4	6
10	1067.6	534.3	1050.5	1049.6	P	577.3		560.3	559.3	5
11	1166.6	583.8	1149.6	1148.6	V	480.3		463.3	462.3	4
12	1313.7	657.4	1296.7	1295.7	F	381.2		364.2	363.2	3
13	1400.7	700.9	1383.7	1382.7	S	234.2		217.1	216.1	2
14	1546.9	773.9	1529.8	1528.8	K	147.1		130.1		1

Gene symbol: Rpl13a

Protein name: 60S ribosomal protein L13a

Protein accession numbers: IPI00223217,IPI00654400

Peptide sequence: (R)CEGINISGNFYR(N)

Exclusive (unique to this protein): TRUE

SEQUEST XCorr score: 2.24 SEQUEST DCn score: 0.704

Fix modifications: C1: Carbamidomethyl (+57.02)Variable modifications: None

charge: 2 Observed m/z: 715.3269

Actual minus calculated peptide mass (AMU): -0.0074454

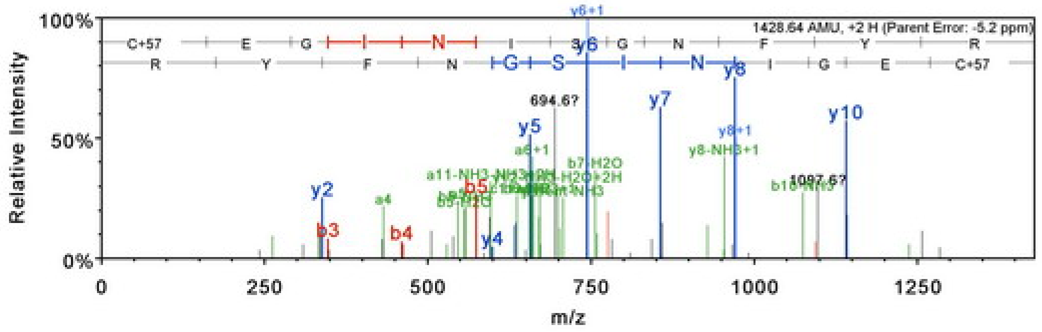

B	B Ions	B+2H	B-NH3	B-H2O	AA	Y Ions	Y+2H	Y-NH3	Y-H2O	Y
1	161.0				C+57	1429.7	715.3	1412.6	1411.6	12
2	290.1			272.1	E	1269.6	635.3	1252.6	1251.6	11
3	347.1			329.1	G	1140.6	570.8	1123.5	1122.6	10
4	460.2			442.2	I	1083.6	542.3	1066.5	1065.5	9
5	574.2		557.2	556.2	N	970.5	485.7	953.5	952.5	8
6	687.3	344.2	670.3	669.3	I	856.4	428.7	839.4	838.4	7
7	774.4	387.7	757.3	756.3	S	743.4	372.2	726.3	725.3	6
8	831.4	416.2	814.3	813.4	G	656.3		639.3		5
9	945.4	473.2	928.4	927.4	N	599.3		582.3		4
10	1092.5	546.7	1075.5	1074.5	F	485.3		468.2		3
11	1255.5	628.3	1238.5	1237.5	Y	338.2		321.2		2
12	1429.7	715.3	1412.6	1411.6	R	175.1		158.1		1

Gene symbol: Rpl14

Protein name: 60S ribosomal protein L14

Protein accession numbers: IPI00133185,IPI00473728,IPI00848835

Peptide sequence: (K)LVAIVDVIDQNR(A)

Exclusive (unique to this protein): TRUE

SEQUEST XCorr score: 2.2　　SEQUEST DCn score: 0.484

Fix modifications: None

Variable modifications: None

charge: 2　　Observed m/z: 677.8852

Actual minus calculated peptide mass (AMU): -0.006958

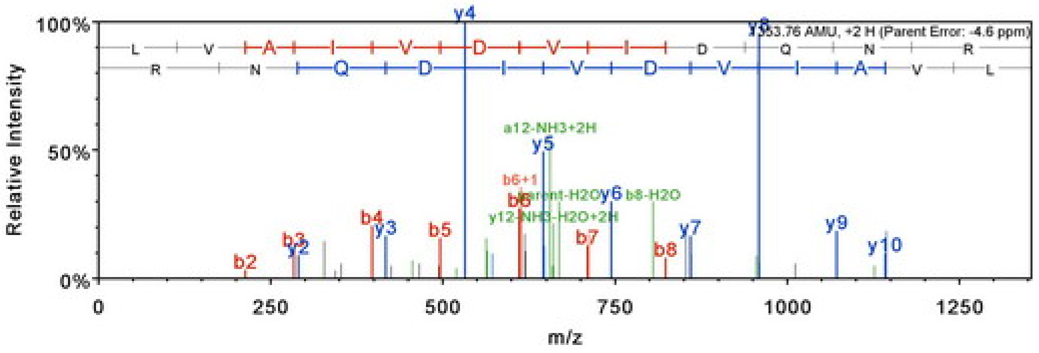

B	B Ions	B+2H	B-NH3	B-H2O	AA	Y Ions	Y+2H	Y-NH3	Y-H2O	Y
1	114.1				L	1354.8	677.9	1337.7	1336.8	12
2	213.2				V	1241.7	621.4	1224.7	1223.7	11
3	284.2				A	1142.6	571.8	1125.6	1124.6	10
4	397.3				I	1071.6	536.3	1054.5	1053.6	9
5	496.4				V	958.5	479.8	941.5	940.5	8
6	611.4	306.2		593.4	D	859.4	430.2	842.4	841.4	7
7	710.5	355.7		692.4	V	744.4	372.7	727.4	726.4	6
8	823.5	412.3		805.5	I	645.3		628.3	627.3	5
9	938.6	469.8		920.6	D	532.3		515.2	514.2	4
10	1066.6	533.8	1049.6	1048.6	Q	417.2		400.2		3
11	1180.7	590.8	1163.6	1162.7	N	289.2		272.1		2
12	1354.8	677.9	1337.7	1336.8	R	175.1		158.1		1

Gene symbol: Rpl19

Protein name: 60S ribosomal protein L19

Protein accession numbers: IPI00122426,IPI00264398,IPI00354889,IPI00466604,IPI00648315,IPI00850221

Peptide sequence: (K)VWLDPNETNEIANANSR(Q)

Exclusive (unique to this protein): TRUE

SEQUEST XCorr score: 2.43　　SEQUEST DCn score: 0.663

Fix modifications: None

Variable modifications: None

charge: 2　　Observed m/z: 971.9629

Actual minus calculated peptide mass (AMU): -0.007813

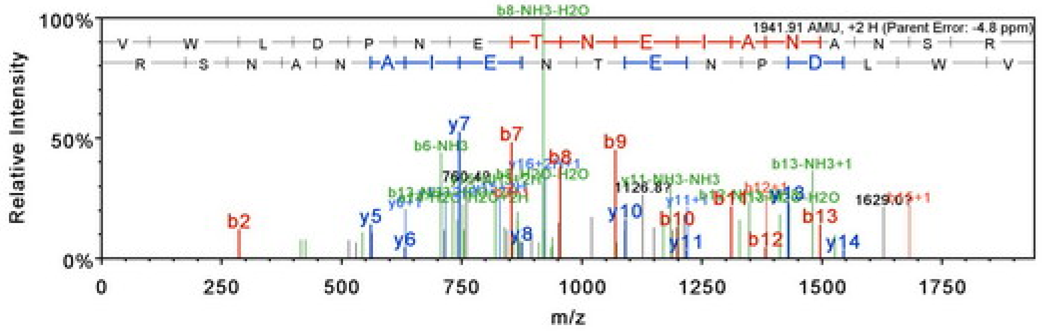

B	B Ions	B+2H	B-NH3	B-H2O	AA	Y Ions	Y+2H	Y-NH3	Y-H2O	Y
1	100.1				V	1942.9	972.0	1925.9	1924.9	17
2	286.2				W	1843.9	922.4	1826.8	1825.9	16
3	399.2				L	1657.8	829.4	1640.8	1639.8	15
4	514.3			496.3	D	1544.7	772.9	1527.7	1526.7	14
5	611.3			593.3	P	1429.7	715.3	1412.6	1411.7	13
6	725.4	363.2	708.3	707.4	N	1332.6	666.8	1315.6	1314.6	12
7	854.4	427.7	837.4	836.4	E	1218.6	609.8	1201.5	1200.6	11
8	955.5	478.2	938.4	937.4	T	1089.5	545.3	1072.5	1071.5	10
9	1069.5	535.3	1052.5	1051.5	N	988.5	494.7	971.5	970.5	9
10	1198.5	599.8	1181.5	1180.5	E	874.4	437.7	857.4	856.4	8
11	1311.6	656.3	1294.6	1293.6	I	745.4	373.2	728.4	727.4	7
12	1382.7	691.8	1365.6	1364.7	A	632.3	316.7	615.3	614.3	6
13	1496.7	748.9	1479.7	1478.7	N	561.3		544.3	543.3	5
14	1567.7	784.4	1550.7	1549.7	A	447.2		430.2	429.2	4
15	1681.8	841.4	1664.8	1663.8	N	376.2		359.2	358.2	3
16	1768.8	884.9	1751.8	1750.8	S	262.1		245.1	244.1	2
17	1942.9	972.0	1925.9	1924.9	R	175.1		158.1		1

Gene symbol: Rpl23

Protein name: 60S ribosomal protein L23

Protein accession numbers: IPI00139780,IPI00849782

Peptide sequence: (R)ISLGLPVGAVINCADNTGAK(N)

Exclusive (unique to this protein): TRUE

SEQUEST XCorr score: 3.3 SEQUEST DCn score: 0.641

Fix modifications: C13: Carbamidomethyl (+57.02)Variable modifications: None

charge: 2 Observed m/z: 985.5203

Actual minus calculated peptide mass (AMU): -0.005493

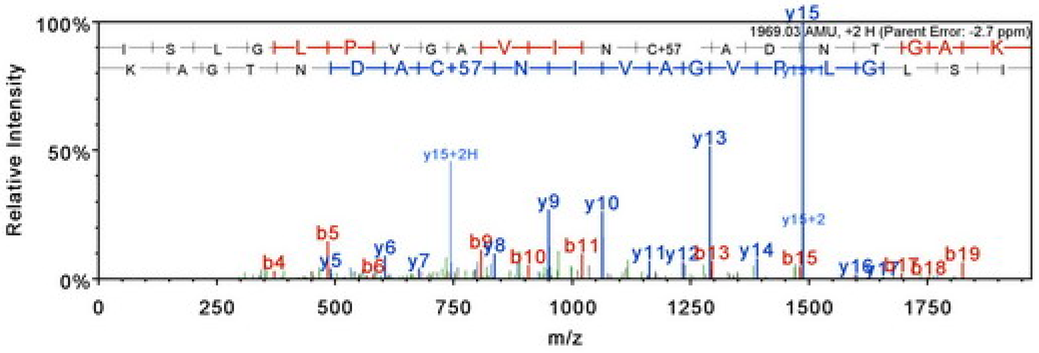

B	B Ions	B+2H	B-NH3	B-H2O	AA	Y Ions	Y+2H	Y-NH3	Y-H2O	Y
1	114.1				I	1970.0	985.5	1953.0	1952.0	20
2	201.1			183.1	S	1857.0	929.0	1839.9	1838.9	19
3	314.2			296.2	L	1769.9	885.5	1752.9	1751.9	18
4	371.2			353.2	G	1656.8	828.9	1639.8	1638.8	17
5	484.3			466.3	L	1599.8	800.4	1582.8	1581.8	16
6	581.4	291.2		563.4	P	1486.7	743.9	1469.7	1468.7	15
7	680.4	340.7		662.4	V	1389.7	695.3	1372.7	1371.7	14
8	737.5	369.2		719.5	G	1290.6	645.8	1273.6	1272.6	13
9	808.5	404.8		790.5	A	1233.6	617.3	1216.6	1215.6	12
10	907.6	454.3		889.6	V	1162.5	581.8	1145.5	1144.5	11
11	1020.7	510.8		1002.6	I	1063.5	532.3	1046.5	1045.5	10
12	1134.7	567.9	1117.7	1116.7	N	950.4	475.7	933.4	932.4	9
13	1294.7	647.9	1277.7	1276.7	C+57	836.4	418.7	819.3	818.4	8
14	1365.8	683.4	1348.7	1347.8	A	676.3	338.7	659.3	658.3	7
15	1480.8	740.9	1463.8	1462.8	D	605.3	303.2	588.3	587.3	6
16	1594.8	797.9	1577.8	1576.8	N	490.3		473.2	472.3	5
17	1695.9	848.4	1678.9	1677.9	T	376.2		359.2	358.2	4
18	1752.9	877.0	1735.9	1734.9	G	275.2		258.1		3
19	1823.9	912.5	1806.9	1805.9	A	218.2		201.1		2
20	1970.0	985.5	1953.0	1952.0	K	147.1		130.1		1

Gene symbol: Rpl6

Protein name: 60S ribosomal protein L6

Protein accession numbers: IPI00313222,IPI00626312

Peptide sequence: (K)QLDSGLLLVTGPLVINR(V)

Exclusive (unique to this protein): TRUE

SEQUEST XCorr score: 2.37 SEQUEST DCn score: 0.76

Fix modifications: None

Variable modifications: None

charge: 2 Observed m/z: 904.537

Actual minus calculated peptide mass (AMU): 0.001343

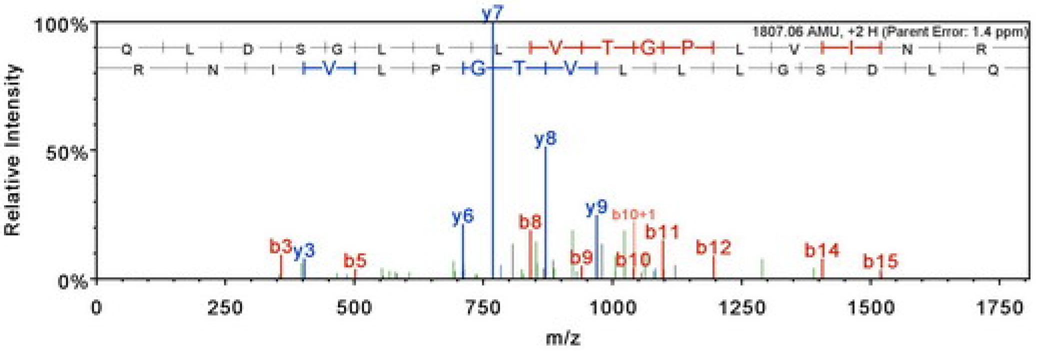

B	B Ions	B+2H	B-NH3	B-H2O	AA	Y Ions	Y+2H	Y-NH3	Y-H2O	Y
1	129.1		112.0		Q	1808.1	904.5	1791.0	1790.1	17
2	242.2		225.1		L	1680.0	840.5	1663.0	1662.0	16
3	357.2		340.2	339.2	D	1566.9	784.0	1549.9	1548.9	15
4	444.2		427.2	426.2	S	1451.9	726.5	1434.9	1433.9	14
5	501.2		484.2	483.2	G	1364.9	682.9	1347.8	1346.9	13
6	614.3	307.7	597.3	596.3	L	1307.8	654.4	1290.8	1289.8	12
7	727.4	364.2	710.4	709.4	L	1194.8	597.9	1177.7	1176.8	11
8	840.5	420.8	823.5	822.5	L	1081.7	541.3	1064.7	1063.7	10
9	939.6	470.3	922.5	921.5	V	968.6	484.8	951.6	950.6	9
10	1040.6	520.8	1023.6	1022.6	T	869.5	435.3	852.5	851.5	8
11	1097.6	549.3	1080.6	1079.6	G	768.5	384.7	751.5		7
12	1194.7	597.8	1177.7	1176.7	P	711.5	356.2	694.4		6
13	1307.8	654.4	1290.7	1289.8	L	614.4		597.4		5
14	1406.8	703.9	1389.8	1388.8	V	501.3		484.3		4
15	1519.9	760.5	1502.9	1501.9	I	402.3		385.2		3
16	1634.0	817.5	1616.9	1615.9	N	289.2		272.1		2
17	1808.1	904.5	1791.0	1790.1	R	175.1		158.1		1

Gene symbol: Rpl9

Protein name: 60S ribosomal protein L9

Protein accession numbers: IPI00122413,IPI00407917,IPI00473762,IPI00850681

Peptide sequence: (K)TILSNQTVDIPENVEITLK(G)

Exclusive (unique to this protein): TRUE

SEQUEST XCorr score: 2.14 SEQUEST DCn score: 0.46

Fix modifications: None

Variable modifications: None

charge: 2 Observed m/z: 1064.0779

Actual minus calculated peptide mass (AMU): -0.0070758

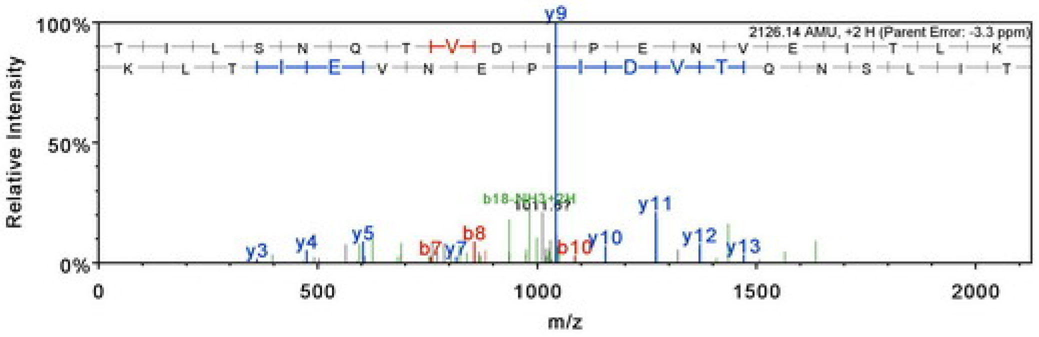

B	B Ions	B+2H	B-NH3	B-H2O	AA	Y Ions	Y+2H	Y-NH3	Y-H2O	Y
1	102.1			84.0	T	2127.2	1064.1	2110.1	2109.1	19
2	215.1			197.1	I	2026.1	1013.6	2009.1	2008.1	18
3	328.2			310.2	L	1913.0	957.0	1896.0	1895.0	17
4	415.3			397.3	S	1799.9	900.5	1782.9	1781.9	16
5	529.3		512.3	511.3	N	1712.9	857.0	1695.9	1694.9	15
6	657.4	329.2	640.3	639.4	Q	1598.9	799.9	1581.8	1580.9	14
7	758.4	379.7	741.4	740.4	T	1470.8	735.9	1453.8	1452.8	13
8	857.5	429.2	840.5	839.5	V	1369.8	685.4	1352.7	1351.8	12
9	972.5	486.8	955.5	954.5	D	1270.7	635.9	1253.7	1252.7	11
10	1085.6	543.3	1068.6	1067.6	I	1155.7	578.3	1138.6	1137.7	10
11	1182.6	591.8	1165.6	1164.6	P	1042.6	521.8	1025.5	1024.6	9
12	1311.7	656.3	1294.7	1293.7	E	945.5	473.3	928.5	927.5	8
13	1425.7	713.4	1408.7	1407.7	N	816.5	408.8	799.5	798.5	7
14	1524.8	762.9	1507.8	1506.8	V	702.4	351.7	685.4	684.4	6
15	1653.8	827.4	1636.8	1635.8	E	603.4		586.4	585.4	5
16	1766.9	884.0	1749.9	1748.9	I	474.3		457.3	456.3	4
17	1868.0	934.5	1850.9	1850.0	T	361.3		344.2	343.2	3
18	1981.1	991.0	1964.0	1963.0	L	260.2		243.2		2
19	2127.2	1064.1	2110.1	2109.1	K	147.1		130.1		1

Gene symbol: Rps11

Protein name: Adult male cerebellum cDNA, RIKEN full-length enriched library, clone:1500004H08 product:ribosomal protein S11, full insert sequence

Protein accession numbers: IPI00117569

Peptide sequence: (R)DVQIGDIVTVGECRPLSK(T)

Exclusive (unique to this protein): TRUE

SEQUEST XCorr score: 2.13 SEQUEST DCn score: 0.67

Fix modifications: C13: Carbamidomethyl (+57.02)Variable modifications: None

charge: 2 Observed m/z: 993.5176

Actual minus calculated peptide mass (AMU): -0.005859

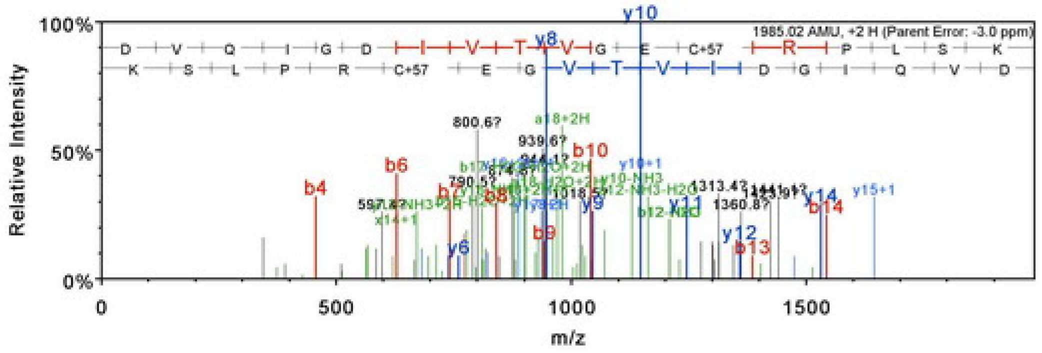

B	B Ions	B+2H	B-NH3	B-H2O	AA	Y Ions	Y+2H	Y-NH3	Y-H2O	Y
1	116.0			98.0	D	1986.0	993.5	1969.0	1968.0	18
2	215.1			197.1	V	1871.0	936.0	1854.0	1853.0	17
3	343.2		326.1	325.2	Q	1771.9	886.5	1754.9	1753.9	16
4	456.3		439.2	438.2	I	1643.9	822.4	1626.9	1625.9	15
5	513.3		496.2	495.3	G	1530.8	765.9	1513.8	1512.8	14
6	628.3	314.7	611.3	610.3	D	1473.8	737.4	1456.8	1455.8	13
7	741.4	371.2	724.4	723.4	I	1358.8	679.9	1341.7	1340.7	12
8	840.5	420.7	823.4	822.4	V	1245.7	623.3	1228.6	1227.7	11
9	941.5	471.3	924.5	923.5	T	1146.6	573.8	1129.6	1128.6	10
10	1040.6	520.8	1023.5	1022.6	V	1045.5	523.3	1028.5	1027.5	9
11	1097.6	549.3	1080.6	1079.6	G	946.5	473.7	929.5	928.5	8
12	1226.6	613.8	1209.6	1208.6	E	889.5	445.2	872.4	871.5	7
13	1386.7	693.8	1369.6	1368.7	C+57	760.4	380.7	743.4	742.4	6
14	1542.8	771.9	1525.7	1524.8	R	600.4	300.7	583.4	582.4	5
15	1639.8	820.4	1622.8	1621.8	P	444.3		427.3	426.3	4
16	1752.9	877.0	1735.9	1734.9	L	347.2		330.2	329.2	3
17	1839.9	920.5	1822.9	1821.9	S	234.2		217.1	216.1	2
18	1986.0	993.5	1969.0	1968.0	K	147.1		130.1		1

Gene symbol: Rps13;EG625298

Protein name: 40S ribosomal protein S13

Protein accession numbers: IPI00125901,IPI00673997,IPI00751092

Peptide sequence: (K)GLTPSQIGVILR(D)

Exclusive (unique to this protein): TRUE

SEQUEST XCorr score: 2.56 SEQUEST DCn score: 0.48

Fix modifications: None

Variable modifications: None

charge: 2 Observed m/z: 627.3795

Actual minus calculated peptide mass (AMU): -0.007202

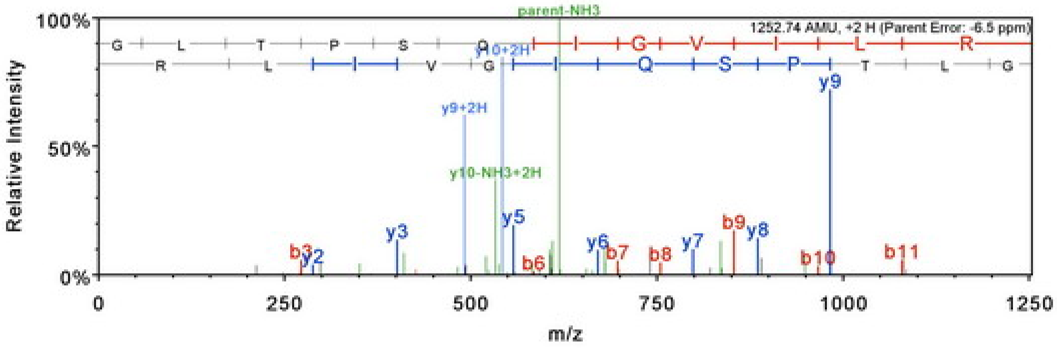

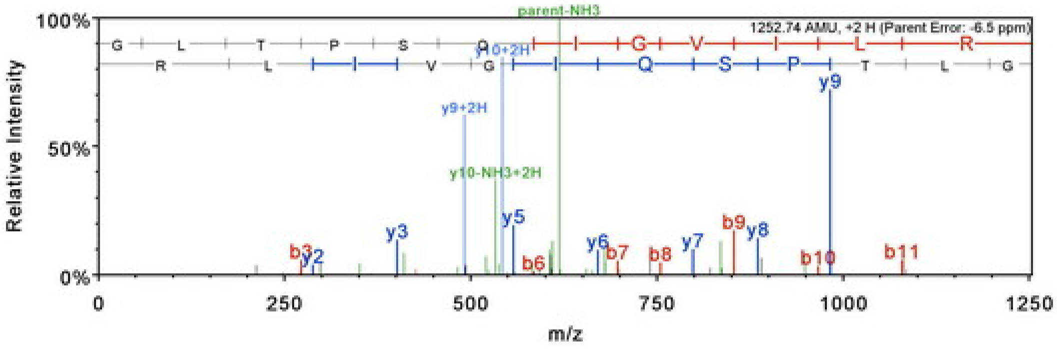

Gene symbol: Rps19

Protein name: 40S ribosomal protein S19

Protein accession numbers: IPI00113241,IPI00125787,IPI00665513

Peptide sequence: (K)ELAPYDENWFYTR(A)

Exclusive (unique to this protein): TRUE

SEQUEST XCorr score: 2.87 SEQUEST DCn score: 0.662

Fix modifications: None

Variable modifications: None

charge: 2 Observed m/z: 852.3865

Actual minus calculated peptide mass (AMU): -0.005249

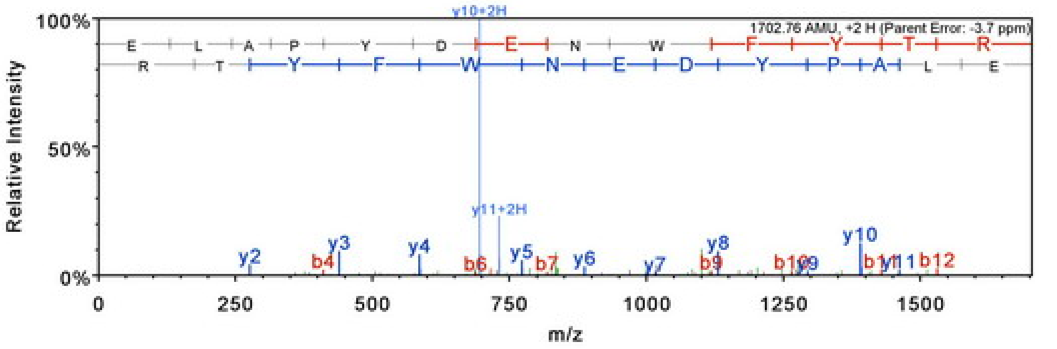

B	B Ions	B+2H	B-NH3	B-H2O	AA	Y Ions	Y+2H	Y-NH3	Y-H2O	Y
1	130.1			112.0	E	1703.8	852.4	1686.7	1685.8	13
2	243.1			225.1	L	1574.7	787.9	1557.7	1556.7	12
3	314.2			296.2	A	1461.6	731.3	1444.6	1443.6	11
4	411.2			393.2	P	1390.6	695.8	1373.6	1372.6	10
5	574.3			556.3	Y	1293.5	647.3	1276.5	1275.5	9
6	689.3	345.2		671.3	D	1130.5	565.8	1113.5	1112.5	8
7	818.4	409.7		800.4	E	1015.5	508.2	998.4	997.5	7
8	932.4	466.7	915.4	914.4	N	886.4	443.7	869.4	868.4	6
9	1118.5	559.7	1101.5	1100.5	W	772.4		755.4	754.4	5
10	1265.5	633.3	1248.5	1247.5	F	586.3		569.3	568.3	4
11	1428.6	714.8	1411.6	1410.6	Y	439.2		422.2	421.2	3
12	1529.7	765.3	1512.6	1511.7	T	276.2		259.1	258.2	2
13	1703.8	852.4	1686.7	1685.8	R	175.1		158.1		1

Gene symbol: Rps20

Protein name: 40S ribosomal protein S20

Protein accession numbers: IPI00323819

Peptide sequence: (R)LIDLHSPSEIVK(Q)

Exclusive (unique to this protein): TRUE

SEQUEST XCorr score: 2.24 SEQUEST DCn score: 0.503

Fix modifications: None

Variable modifications: None

charge: 2 Observed m/z: 675.8857

Actual minus calculated peptide mass (AMU): 0.0001221

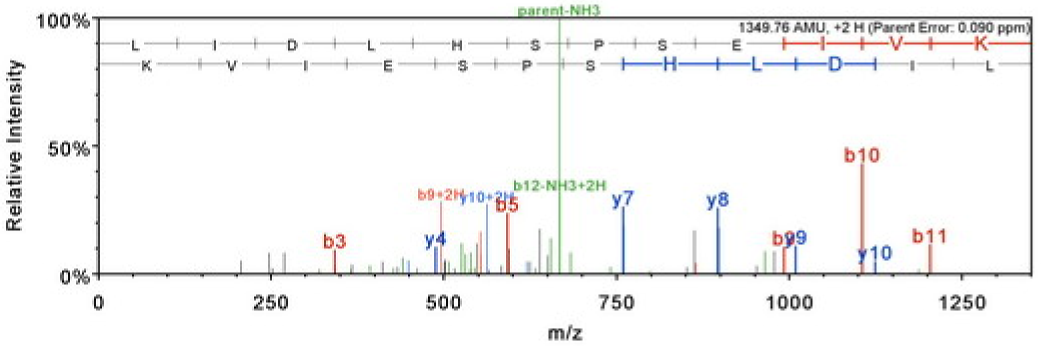

B	B Ions	B+2H	B-NH3	B-H2O	AA	Y Ions	Y+2H	Y-NH3	Y-H2O	Y
1	114.1				L	1350.8	675.9	1333.7	1332.8	12
2	227.2				I	1237.7	619.3	1220.7	1219.7	11
3	342.2			324.2	D	1124.6	562.8	1107.6	1106.6	10
4	455.3			437.3	L	1009.6	505.3	992.5	991.6	9
5	592.4	296.7		574.3	H	896.5	448.8	879.5	878.5	8
6	679.4	340.2		661.4	S	759.4	380.2	742.4	741.4	7
7	776.4	388.7		758.4	P	672.4	336.7	655.4	654.4	6
8	863.5	432.2		845.5	S	575.3		558.3	557.3	5
9	992.5	496.8		974.5	E	488.3		471.3	470.3	4
10	1105.6	553.3		1087.6	I	359.3		342.2		3
11	1204.7	602.8		1186.7	V	246.2		229.2		2
12	1350.8	675.9	1333.7	1332.8	K	147.1		130.1		1

Gene symbol: Rps23

Protein name: 11 days embryo whole body cDNA, RIKEN full-length enriched library, clone:2700086E01 product:ribosomal protein S23, full insert sequence

Protein accession numbers: IPI00131357,IPI00605025,IPI00750216,IPI00761494,IPI00762201

Peptide sequence: (K)VANVSLLALYK(G)

Exclusive (unique to this protein): TRUE

SEQUEST XCorr score: 2.47 SEQUEST DCn score: 0.629

Fix modifications: None

Variable modifications: None

charge: 2 Observed m/z: 595.8579

Actual minus calculated peptide mass (AMU): -0.006958

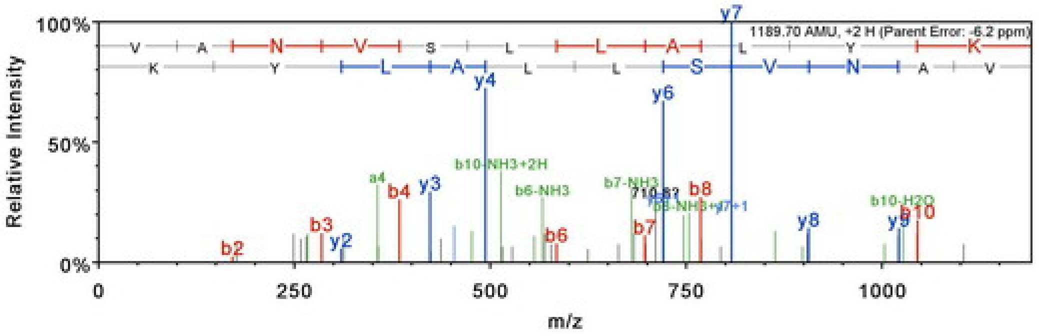

B	B Ions	B+2H	B-NH3	B-H2O	AA	Y Ions	Y+2H	Y-NH3	Y-H2O	Y
1	100.1				V	1190.7	595.9	1173.7	1172.7	11
2	171.1				A	1091.7	546.3	1074.6	1073.6	10
3	285.2		268.1		N	1020.6	510.8	1003.6	1002.6	9
4	384.2		367.2		V	906.6	453.8	889.5	888.6	8
5	471.3		454.2	453.3	S	807.5	404.3	790.5	789.5	7
6	584.3	292.7	567.3	566.3	L	720.5	360.7	703.4		6
7	697.4	349.2	680.4	679.4	L	607.4		590.4		5
8	768.5	384.7	751.4	750.5	A	494.3		477.3		4
9	881.6	441.3	864.5	863.5	L	423.3		406.2		3
10	1044.6	522.8	1027.6	1026.6	Y	310.2		293.2		2
11	1190.7	595.9	1173.7	1172.7	K	147.1		130.1		1

Gene symbol: Rps4x

Protein name: 40S ribosomal protein S4, X isoform

Protein accession numbers: IPI00331092,IPI00662162,IPI00752798

Peptide sequence: (R)LSNIFVIGK(G)

Exclusive (unique to this protein): TRUE

SEQUEST XCorr score: 2.01 SEQUEST DCn score: 0.45

Fix modifications: None

Variable modifications: None

charge: 2 Observed m/z: 495.8017

Actual minus calculated peptide mass (AMU): -0.003296

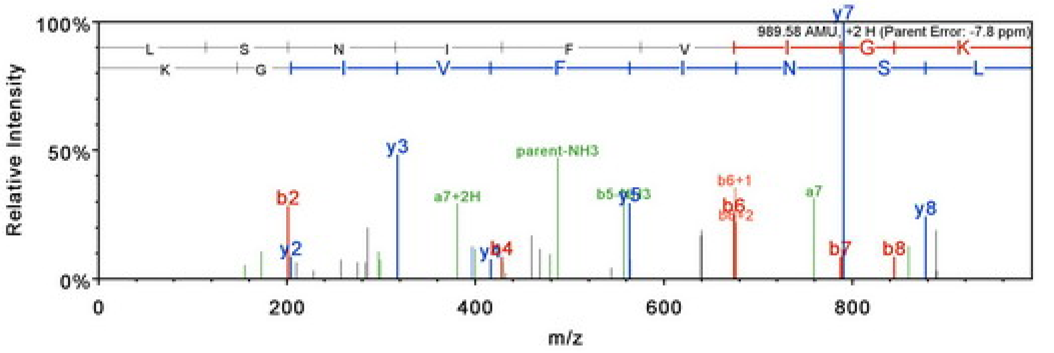

B	B Ions	B+2H	B-NH3	B-H2O	AA	Y Ions	Y+2H	Y-NH3	Y-H2O	Y
1	114.1				L	990.6	495.8	973.6	972.6	9
2	201.1			183.1	S	877.5	439.3	860.5	859.5	8
3	315.2		298.1	297.2	N	790.5	395.8	773.5		7
4	428.3		411.2	410.2	I	676.4	338.7	659.4		6
5	575.3		558.3	557.3	F	563.4		546.3		5
6	674.4	337.7	657.4	656.4	V	416.3		399.3		4
7	787.5	394.2	770.5	769.5	I	317.2		300.2		3
8	844.5	422.8	827.5	826.5	G	204.1		187.1		2
9	990.6	495.8	973.6	972.6	K	147.1		130.1		1

Gene symbol: Rps5

Protein name: 40S ribosomal protein S5

Protein accession numbers: IPI00125521

Peptide sequence: (R)VNQAIWLLCTGAR(E)

Exclusive (unique to this protein): TRUE

SEQUEST XCorr score: 2.04 SEQUEST DCn score: 0.606

Fix modifications: C9: Carbamidomethyl (+57.02)Variable modifications: None

charge: 2 Observed m/z: 751.4026

Actual minus calculated peptide mass (AMU): 0.002441

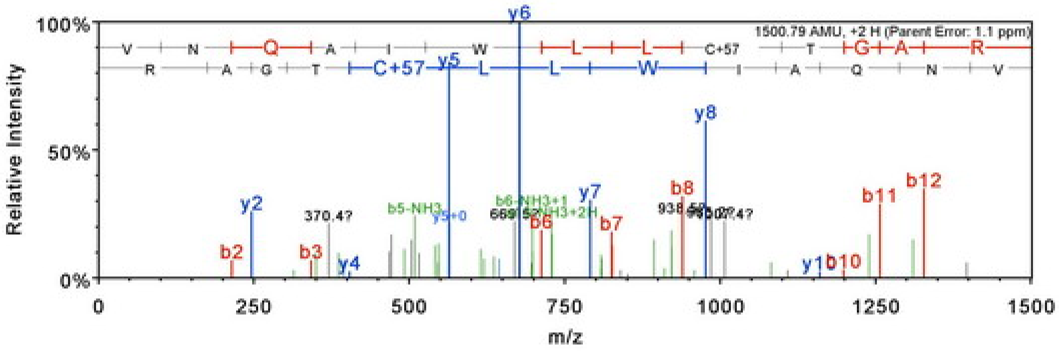

B	B Ions	B+2H	B-NH3	B-H2O	AA	Y Ions	Y+2H	Y-NH3	Y-H2O	Y
1	100.1				V	1501.8	751.4	1484.8	1483.8	13
2	214.1		197.1		N	1402.7	701.9	1385.7	1384.7	12
3	342.2		325.2		Q	1288.7	644.9	1271.7	1270.7	11
4	413.2		396.2		A	1160.6	580.8	1143.6	1142.6	10
5	526.3		509.3		I	1089.6	545.3	1072.6	1071.6	9
6	712.4	356.7	695.4		W	976.5	488.8	959.5	958.5	8
7	825.5	413.2	808.4		L	790.4	395.7	773.4	772.4	7
8	938.6	469.8	921.5		L	677.3	339.2	660.3	659.3	6
9	1098.6	549.8	1081.5		C+57	564.3		547.2	546.3	5
10	1199.6	600.3	1182.6	1181.6	T	404.2		387.2	386.2	4
11	1256.7	628.8	1239.6	1238.6	G	303.2		286.2		3
12	1327.7	664.4	1310.7	1309.7	A	246.2		229.1		2
13	1501.8	751.4	1484.8	1483.8	R	175.1		158.1		1

Gene symbol: Rps9

Protein name: 40S ribosomal protein S9

Protein accession numbers: IPI00420726

Peptide sequence: (R)LFEGNALLR(R)

Exclusive (unique to this protein): TRUE

SEQUEST XCorr score: 2.12 SEQUEST DCn score: 0.445

Fix modifications: None

Variable modifications: None

charge: 2 Observed m/z: 516.7955

Actual minus calculated peptide mass (AMU): -0.0012249

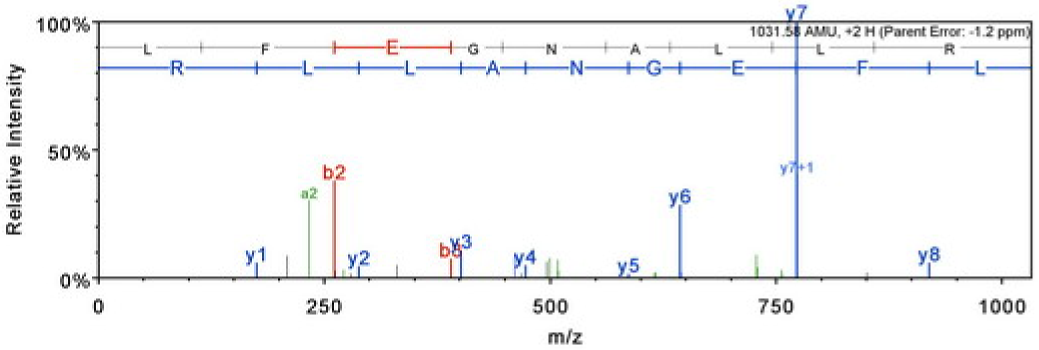

B	B Ions	B+2H	B-NH3	B-H2O	AA	Y Ions	Y+2H	Y-NH3	Y-H2O	Y
1	114.1				L	1032.6	516.8	1015.6	1014.6	9
2	261.2				F	919.5	460.3	902.5	901.5	8
3	390.2			372.2	E	772.4	386.7	755.4	754.4	7
4	447.2			429.2	G	643.4	322.2	626.4		6
5	561.3		544.2	543.3	N	586.4		569.3		5
6	632.3	316.7	615.3	614.3	A	472.3		455.3		4
7	745.4	373.2	728.4	727.4	L	401.3		384.3		3
8	858.5	429.7	841.5	840.5	L	288.2		271.2		2
9	1032.6	516.8	1015.6	1014.6	R	175.1		158.1		1

Gene symbol: Sart3
Protein name: Isoform 1 of Squamous cell carcinoma antigen recognized by T-cells 3
Protein accession numbers: IPI00458854
Peptide sequence: (R)ALEYLQQEVEER(F)
Exclusive (unique to this protein): TRUE
SEQUEST XCorr score: 2.91 SEQUEST DCn score: 0.541
Fix modifications: None
Variable modifications: None
charge: 2 Observed m/z: 753.872
Actual minus calculated peptide mass (AMU): -0.007935

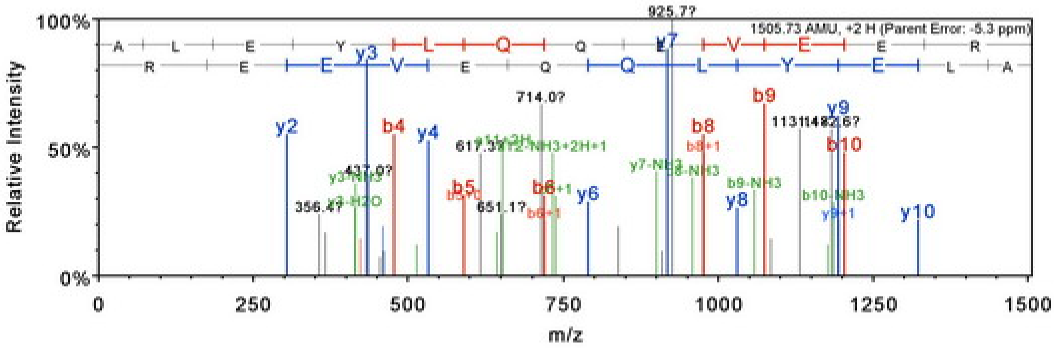

B	B Ions	B+2H	B-NH3	B-H2O	AA	Y Ions	Y+2H	Y-NH3	Y-H2O	Y
1	72.0				A	1506.7	753.9	1489.7	1488.7	12
2	185.1				L	1435.7	718.4	1418.7	1417.7	11
3	314.2			296.2	E	1322.6	661.8	1305.6	1304.6	10
4	477.2			459.2	Y	1193.6	597.3	1176.5	1175.6	9
5	590.3			572.3	L	1030.5	515.8	1013.5	1012.5	8
6	718.4	359.7	701.4	700.4	Q	917.4	459.2	900.4	899.4	7
7	846.4	423.7	829.4	828.4	Q	789.4	395.2	772.4	771.4	6
8	975.5	488.2	958.5	957.5	E	661.3		644.3	643.3	5
9	1074.5	537.8	1057.5	1056.5	V	532.3		515.3	514.3	4
10	1203.6	602.3	1186.6	1185.6	E	433.2		416.2	415.2	3
11	1332.6	666.8	1315.6	1314.6	E	304.2		287.1	286.2	2
12	1506.7	753.9	1489.7	1488.7	R	175.1		158.1		1

Gene symbol: Sdf2
Protein name: Stromal cell-derived factor 2 precursor
Protein accession numbers: IPI00123852
Peptide sequence: (R)YGSGSGQQSVTGVTSVDDSNSYWR(I)
Exclusive (unique to this protein): TRUE
SEQUEST XCorr score: 2.56 SEQUEST DCn score: 0.811
Fix modifications: None
Variable modifications: None
charge: 2 Observed m/z: 1269.0599
Actual minus calculated peptide mass (AMU): -0.0056153

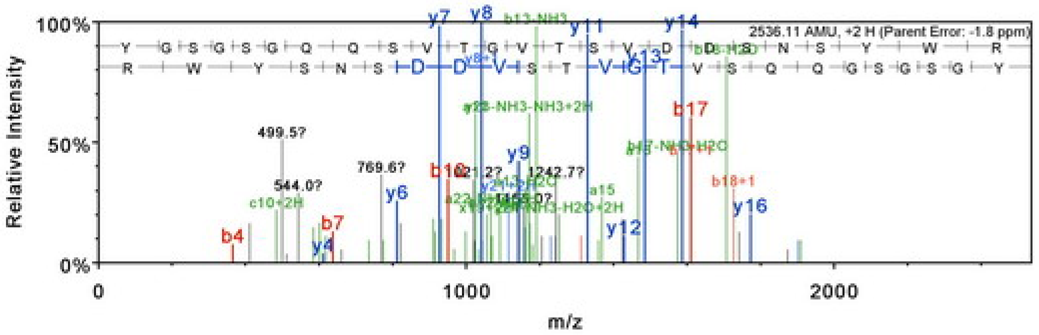

B	B Ions	B+2H	B-NH3	B-H2O	AA	Y Ions	Y+2H	Y-NH3	Y-H2O	Y
1	164.1				Y	2537.1	1269.1	2520.1	2519.1	24
2	221.1				G	2374.1	1187.5	2357.0	2356.0	23
3	308.1			290.1	S	2317.0	1159.0	2300.0	2299.0	22
4	365.2			347.1	G	2230.0	1115.5	2213.0	2212.0	21
5	452.2			434.2	S	2173.0	1087.0	2155.9	2155.0	20
6	509.2	255.1		491.2	G	2085.9	1043.5	2068.9	2067.9	19
7	637.3	319.1	620.2	619.3	Q	2028.9	1015.0	2011.9	2010.9	18
8	765.3	383.2	748.3	747.3	Q	1900.9	950.9	1883.8	1882.9	17
9	852.4	426.7	835.3	834.3	S	1772.8	886.9	1755.8	1754.8	16
10	951.4	476.2	934.4	933.4	V	1685.8	843.4	1668.8	1667.8	15
11	1052.5	526.7	1035.4	1034.5	T	1586.7	793.9	1569.7	1568.7	14
12	1109.5	555.3	1092.5	1091.5	G	1485.7	743.3	1468.6	1467.7	13
13	1208.5	604.8	1191.5	1190.5	V	1428.6	714.8	1411.6	1410.6	12
14	1309.6	655.3	1292.6	1291.6	T	1329.6	665.3	1312.5	1311.6	11
15	1396.6	698.8	1379.6	1378.6	S	1228.5	614.8	1211.5	1210.5	10
16	1495.7	748.4	1478.7	1477.7	V	1141.5	571.3	1124.5	1123.5	9
17	1610.7	805.9	1593.7	1592.7	D	1042.4	521.7	1025.4	1024.4	8
18	1725.8	863.4	1708.7	1707.8	D	927.4	464.2	910.4	909.4	7
19	1812.8	906.9	1795.8	1794.8	S	812.4	406.7	795.3	794.4	6
20	1926.8	963.9	1909.8	1908.8	N	725.3		708.3	707.3	5
21	2013.9	1007.4	1996.8	1995.9	S	611.3		594.3	593.3	4
22	2176.9	1089.0	2159.9	2158.9	Y	524.3		507.2		3
23	2363.0	1182.0	2346.0	2345.0	W	361.2		344.2		2
24	2537.1	1269.1	2520.1	2519.1	R	175.1		158.1		1

Gene symbol: Sdf2l1

Protein name: Stromal cell-derived factor 2-like protein 1 precursor

Protein accession numbers: IPI00227657

Peptide sequence: (K)YGSGSGQQSVTGVEESDDANSYWR(I)

Exclusive (unique to this protein): TRUE

SEQUEST XCorr score: 2.16 SEQUEST DCn score: 0.692

Fix modifications: None

Variable modifications: None

charge: 2 Observed m/z: 1290.0579

Actual minus calculated peptide mass (AMU): 0.01587

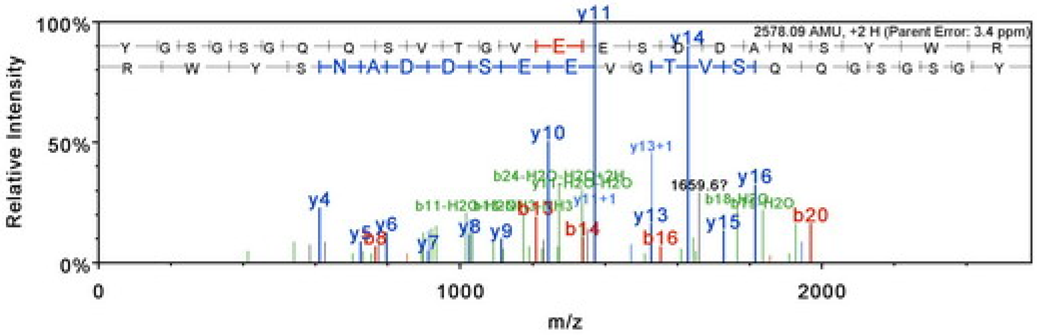

B	B Ions	B+2H	B-NH3	B-H2O	AA	Y Ions	Y+2H	Y-NH3	Y-H2O	Y
1	164.1				Y	2579.1	1290.0	2562.1	2561.1	24
2	221.1				G	2416.0	1208.5	2399.0	2398.0	23
3	308.1			290.1	S	2359.0	1180.0	2342.0	2341.0	22
4	365.2			347.1	G	2272.0	1136.5	2255.0	2254.0	21
5	452.2			434.2	S	2215.0	1108.0	2197.9	2196.9	20
6	509.2	255.1		491.2	G	2127.9	1064.5	2110.9	2109.9	19
7	637.3	319.1	620.2	619.3	Q	2070.9	1036.0	2053.9	2052.9	18
8	765.3	383.2	748.3	747.3	Q	1942.8	971.9	1925.8	1924.8	17
9	852.4	426.7	835.3	834.3	S	1814.8	907.9	1797.8	1796.8	16
10	951.4	476.2	934.4	933.4	V	1727.8	864.4	1710.7	1709.7	15
11	1052.5	526.7	1035.4	1034.5	T	1628.7	814.9	1611.7	1610.7	14
12	1109.5	555.3	1092.5	1091.5	G	1527.6	764.3	1510.6	1509.6	13
13	1208.5	604.8	1191.5	1190.5	V	1470.6	735.8	1453.6	1452.6	12
14	1337.6	669.3	1320.6	1319.6	E	1371.5	686.3	1354.5	1353.5	11
15	1466.6	733.8	1449.6	1448.6	E	1242.5	621.8	1225.5	1224.5	10
16	1553.7	777.3	1536.7	1535.7	S	1113.5	557.2	1096.4	1095.5	9
17	1668.7	834.9	1651.7	1650.7	D	1026.4	513.7	1009.4	1008.4	8
18	1783.7	892.4	1766.7	1765.7	D	911.4	456.2	894.4	893.4	7
19	1854.8	927.9	1837.7	1836.8	A	796.4	398.7	779.4	778.4	6
20	1968.8	984.9	1951.8	1950.8	N	725.3		708.3	707.3	5
21	2055.8	1028.4	2038.8	2037.8	S	611.3		594.3	593.3	4
22	2218.9	1110.0	2201.9	2200.9	Y	524.3		507.2		3
23	2405.0	1203.0	2388.0	2387.0	W	361.2		344.2		2
24	2579.1	1290.0	2562.1	2561.1	R	175.1		158.1		1

Gene symbol: Sec31a

Protein name: SEC31-like 1

Protein accession numbers: IPI00377592,IPI00850448

Peptide sequence: (R)TQACLCYICAGNVER(L)

Exclusive (unique to this protein): TRUE

SEQUEST XCorr score: 2.3 SEQUEST DCn score: 0.571

Fix modifications: C4: Carbamidomethyl (+57.02), C6: Carbamidomethyl (+57.02), C9: Carbamidomethyl (+57.02)Variable modifications: None

charge: 2 Observed m/z: 907.9009

Actual minus calculated peptide mass (AMU): -0.004761

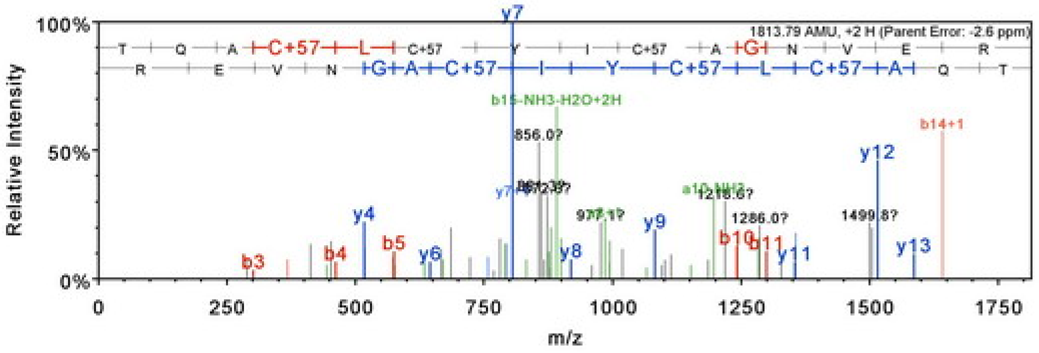

B	B Ions	B+2H	B-NH3	B-H2O	AA	Y Ions	Y+2H	Y-NH3	Y-H2O	Y
1	102.1			84.0	T	1814.8	907.9	1797.8	1796.8	15
2	230.1		213.1	212.1	Q	1713.8	857.4	1696.7	1695.7	14
3	301.2		284.1	283.1	A	1585.7	793.4	1568.7	1567.7	13
4	461.2		444.2	443.2	C+57	1514.7	757.8	1497.6	1496.6	12
5	574.3		557.2	556.3	L	1354.6	677.8	1337.6	1336.6	11
6	734.3	367.7	717.3	716.3	C+57	1241.5	621.3	1224.5	1223.5	10
7	897.4	449.2	880.3	879.4	Y	1081.5	541.3	1064.5	1063.5	9
8	1010.4	505.7	993.4	992.4	I	918.5	459.7	901.4	900.4	8
9	1170.5	585.7	1153.5	1152.5	C+57	805.4	403.2	788.3	787.4	7
10	1241.5	621.3	1224.5	1223.5	A	645.3	323.2	628.3	627.3	6
11	1298.5	649.8	1281.5	1280.5	G	574.3		557.3	556.3	5
12	1412.6	706.8	1395.5	1394.6	N	517.3		500.3	499.3	4
13	1511.6	756.3	1494.6	1493.6	V	403.2		386.2	385.2	3
14	1640.7	820.9	1623.7	1622.7	E	304.2		287.1	286.2	2
15	1814.8	907.9	1797.8	1796.8	R	175.1		158.1		1

Gene symbol: Sel1l

Protein name: Isoform 1 of Sel-1 homolog precursor

Protein accession numbers: IPI00131143,IPI00224252

Peptide sequence: (R)LVANHVASDISLTGGSVVQR(I)

Exclusive (unique to this protein): TRUE

SEQUEST XCorr score: 2.14 SEQUEST DCn score: 0.545

Fix modifications: None

Variable modifications: None

charge: 2 Observed m/z: 1012.0497

Actual minus calculated peptide mass (AMU): -0.0019551

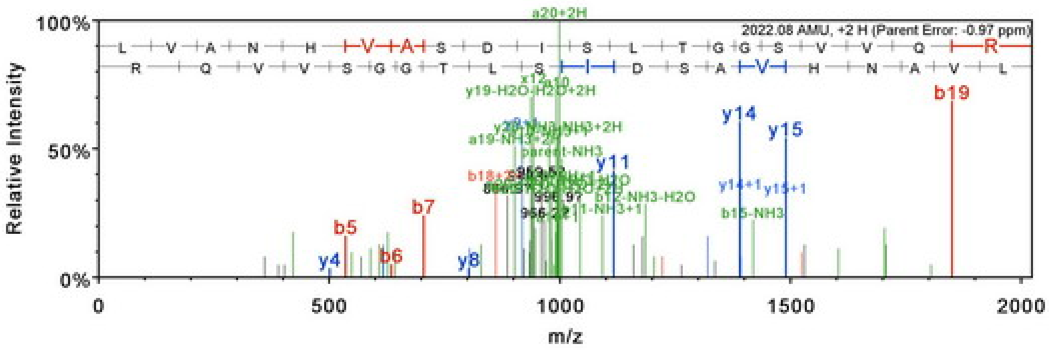

B	B Ions	B+2H	B-NH3	B-H2O	AA	Y Ions	Y+2H	Y-NH3	Y-H2O	Y
1	114.1				L	2023.1	1012.1	2006.1	2005.1	20
2	213.2				V	1910.0	955.5	1893.0	1892.0	19
3	284.2				A	1810.9	906.0	1793.9	1792.9	18
4	398.2		381.2		N	1739.9	870.5	1722.9	1721.9	17
5	535.3	268.1	518.3		H	1625.9	813.4	1608.8	1607.9	16
6	634.4	317.7	617.3		V	1488.8	744.9	1471.8	1470.8	15
7	705.4	353.2	688.4		A	1389.7	695.4	1372.7	1371.7	14
8	792.4	396.7	775.4	774.4	S	1318.7	659.9	1301.7	1300.7	13
9	907.5	454.2	890.4	889.5	D	1231.7	616.3	1214.6	1213.7	12
10	1020.6	510.8	1003.5	1002.5	I	1116.6	558.8	1099.6	1098.6	11
11	1107.6	554.3	1090.5	1089.6	S	1003.6	502.3	986.5	985.5	10
12	1220.7	610.8	1203.6	1202.7	L	916.5	458.8	899.5	898.5	9
13	1321.7	661.4	1304.7	1303.7	T	803.4	402.2	786.4	785.4	8
14	1378.7	689.9	1361.7	1360.7	G	702.4	351.7	685.4	684.4	7
15	1435.8	718.4	1418.7	1417.7	G	645.4	323.2	628.3	627.4	6
16	1522.8	761.9	1505.8	1504.8	S	588.4		571.3	570.3	5
17	1621.9	811.4	1604.8	1603.8	V	501.3		484.3		4
18	1720.9	861.0	1703.9	1702.9	V	402.3		385.2		3
19	1849.0	925.0	1832.0	1831.0	Q	303.2		286.2		2
20	2023.1	1012.1	2006.1	2005.1	R	175.1		158.1		1

Gene symbol: Sept2
Protein name: Septin-2
Protein accession numbers: IPI00114945
Peptide sequence: (R)LTVVDTPGYGDAINCR(D)
Exclusive (unique to this protein): TRUE
SEQUEST XCorr score: 2 SEQUEST DCn score: 0.564
Fix modifications: C15: Carbamidomethyl (+57.02)Variable modifications: None
charge: 2 Observed m/z: 875.9213
Actual minus calculated peptide mass (AMU): -0.008667

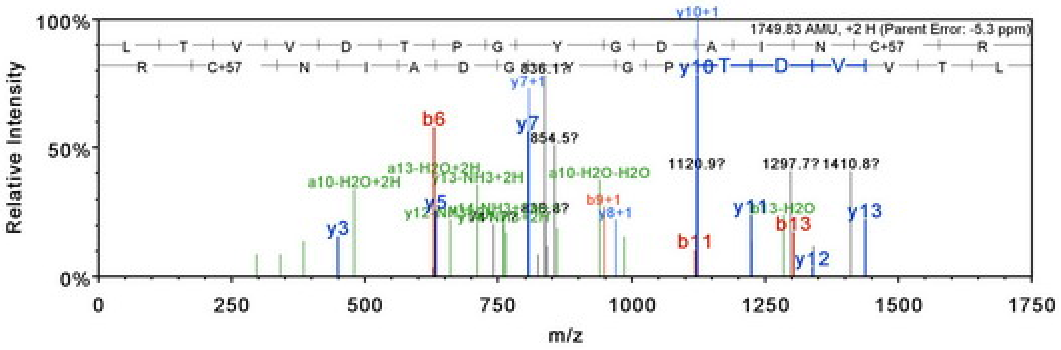

B	B Ions	B+2H	B-NH3	B-H2O	AA	Y Ions	Y+2H	Y-NH3	Y-H2O	Y
1	114.1				L	1750.8	875.9	1733.8	1732.8	16
2	215.1			197.1	T	1637.8	819.4	1620.7	1619.8	15
3	314.2			296.2	V	1536.7	768.9	1519.7	1518.7	14
4	413.3			395.3	V	1437.6	719.3	1420.6	1419.6	13
5	528.3			510.3	D	1338.6	669.8	1321.5	1320.6	12
6	629.4	315.2		611.3	T	1223.5	612.3	1206.5	1205.5	11
7	726.4	363.7		708.4	P	1122.5	561.8	1105.5	1104.5	10
8	783.4	392.2		765.4	G	1025.5	513.2	1008.4	1007.4	9
9	946.5	473.8		928.5	Y	968.4	484.7	951.4	950.4	8
10	1003.5	502.3		985.5	G	805.4	403.2	788.3	787.4	7
11	1118.5	559.8		1100.5	D	748.3	374.7	731.3	730.3	6
12	1189.6	595.3		1171.6	A	633.3		616.3		5
13	1302.7	651.8		1284.7	I	562.3		545.3		4
14	1416.7	708.9	1399.7	1398.7	N	449.2		432.2		3
15	1576.7	788.9	1559.7	1558.7	C+57	335.2		318.1		2
16	1750.8	875.9	1733.8	1732.8	R	175.1		158.1		1

Gene symbol: Sfrs3

Protein name: Isoform Long of Splicing factor, arginine/serine-rich 3

Protein accession numbers: IPI00129323,IPI00221826,IPI00346575,IPI00474970,IPI00756474,IPI00850997

Peptide sequence: (R)AFGYYGPLR(S)

Exclusive (unique to this protein): TRUE

SEQUEST XCorr score: 2.05 SEQUEST DCn score: 0.368

Fix modifications: None

Variable modifications: None

charge: 2 Observed m/z: 522.2685

Actual minus calculated peptide mass (AMU): -0.002319

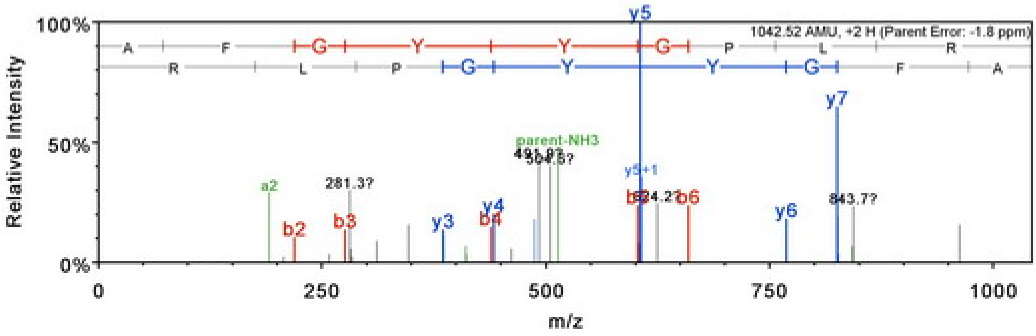

B	B Ions	B+2H	B-NH3	B-H2O	AA	Y Ions	Y+2H	Y-NH3	Y-H2O	Y
1	72.0				A	1043.5	522.3	1026.5		9
2	219.1				F	972.5	486.8	955.5		8
3	276.1				G	825.4	413.2	808.4		7
4	439.2				Y	768.4	384.7	751.4		6
5	602.3				Y	605.3		588.3		5
6	659.3	330.2			G	442.3		425.3		4
7	756.3	378.7			P	385.3		368.2		3
8	869.4	435.2			L	288.2		271.2		2
9	1043.5	522.3	1026.5		R	175.1		158.1		1

Gene symbol: Sgms2
Protein name: Phosphatidylcholine:ceramide cholinephosphotransferase 2
Protein accession numbers: IPI00187570
Peptide sequence: (K)LNGDSQAKIQR(I)
Exclusive (unique to this protein): TRUE
SEQUEST XCorr score: 2.08   SEQUEST DCn score: 0.355
Fix modifications: None
Variable modifications: None
charge: 2   Observed m/z: 615.3288
Actual minus calculated peptide mass (AMU): -0.01062

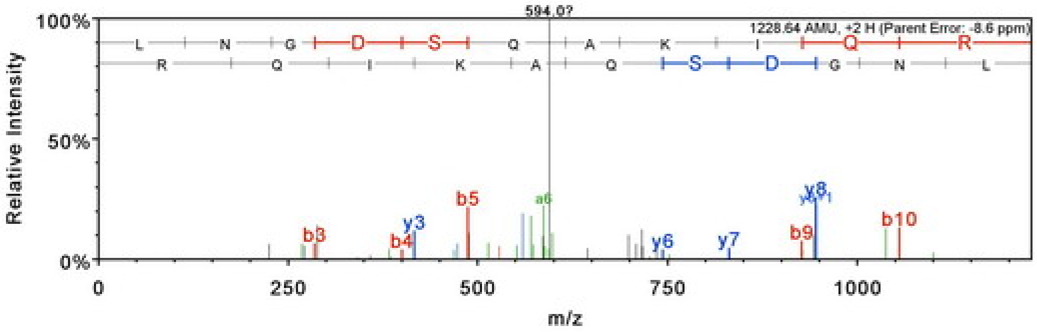

B	B Ions	B+2H	B-NH3	B-H2O	AA	Y Ions	Y+2H	Y-NH3	Y-H2O	Y
1	114.1				L	1229.7	615.3	1212.6	1211.7	11
2	228.1		211.1		N	1116.6	558.8	1099.5	1098.6	10
3	285.2		268.1		G	1002.5	501.8	985.5	984.5	9
4	400.2		383.2	382.2	D	945.5	473.3	928.5	927.5	8
5	487.2		470.2	469.2	S	830.5	415.8	813.5	812.5	7
6	615.3	308.1	598.3	597.3	Q	743.5	372.2	726.4		6
7	686.3	343.7	669.3	668.3	A	615.4	308.2	598.4		5
8	814.4	407.7	797.4	796.4	K	544.4	272.7	527.3		4
9	927.5	464.3	910.5	909.5	I	416.3		399.2		3
10	1055.5	528.3	1038.5	1037.5	Q	303.2		286.2		2
11	1229.7	615.3	1212.6	1211.7	R	175.1		158.1		1

Gene symbol: Ski
Protein name: Sloan-Kettering viral oncogene homolog
Protein accession numbers: IPI00462772,IPI00622896
Peptide sequence: (R)SLHQELEFLR(V)
Exclusive (unique to this protein): TRUE
SEQUEST XCorr score: 2.05   SEQUEST DCn score: 0.594
Fix modifications: None
Variable modifications: None
charge: 2   Observed m/z: 636.3404
Actual minus calculated peptide mass (AMU): -0.001831

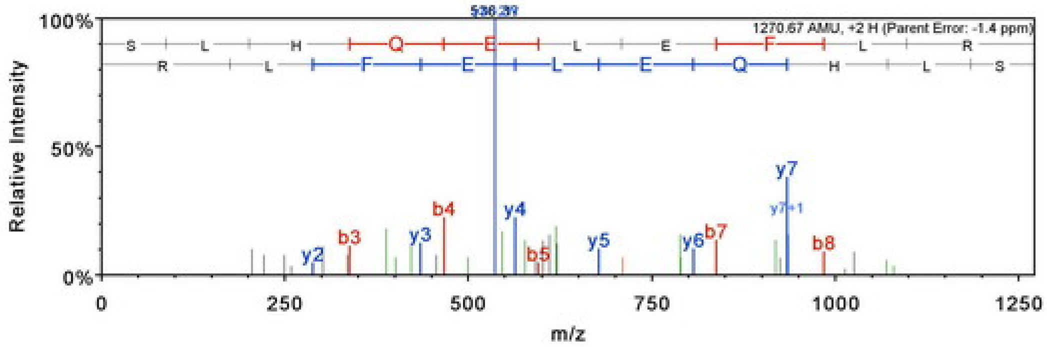

B	B Ions	B+2H	B-NH3	B-H2O	AA	Y Ions	Y+2H	Y-NH3	Y-H2O	Y
1	88.0			70.0	S	1271.7	636.3	1254.7	1253.7	10
2	201.1			183.1	L	1184.6	592.8	1167.6	1166.6	9
3	338.2	169.6		320.2	H	1071.6	536.3	1054.5	1053.5	8
4	466.2	233.6	449.2	448.2	Q	934.5	467.8	917.5	916.5	7
5	595.3	298.2	578.3	577.3	E	806.4	403.7	789.4	788.4	6
6	708.4	354.7	691.3	690.4	L	677.4		660.4	659.4	5
7	837.4	419.2	820.4	819.4	E	564.3		547.3	546.3	4
8	984.5	492.7	967.5	966.5	F	435.3		418.3		3
9	1097.6	549.3	1080.5	1079.5	L	288.2		271.2		2
10	1271.7	636.3	1254.7	1253.7	R	175.1		158.1		1

Gene symbol: Slc25a1

Protein name: NOD-derived CD11c +ve dendritic cells cDNA, RIKEN full-length enriched library, clone:F630026H16 product:solute carrier family 25 (mitochondrial carrier; citrate transporter), member 1, full insert sequence

Protein accession numbers: IPI00276926

Peptide sequence: (R)GLSSLLYGSIPK(A)

Exclusive (unique to this protein): TRUE

SEQUEST XCorr score: 2.37 SEQUEST DCn score: 0.549

Fix modifications: None

Variable modifications: None

charge: 2 Observed m/z: 617.8525

Actual minus calculated peptide mass (AMU): -0.007568

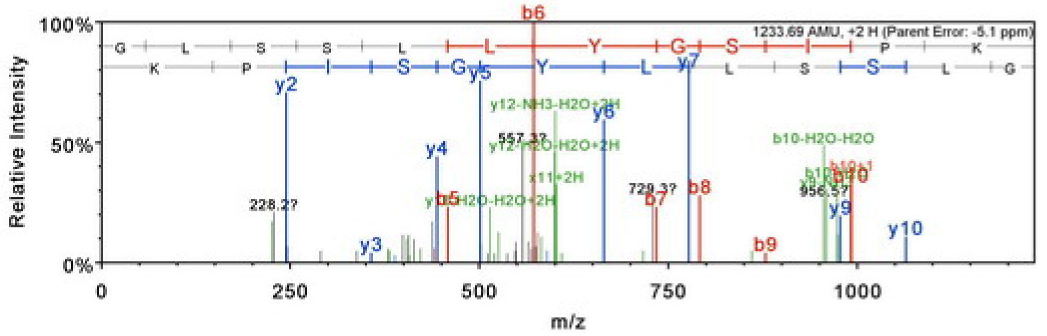

B	B Ions	B+2H	B-NH3	B-H2O	AA	Y Ions	Y+2H	Y-NH3	Y-H2O	Y
1	58.0				G	1234.7	617.9	1217.7	1216.7	12
2	171.1				L	1177.7	589.4	1160.7	1159.7	11
3	258.1			240.1	S	1064.6	532.8	1047.6	1046.6	10
4	345.2			327.2	S	977.6	489.3	960.5	959.6	9
5	458.3			440.3	L	890.5	445.8	873.5	872.5	8
6	571.4	286.2		553.3	L	777.5	389.2	760.4	759.4	7
7	734.4	367.7		716.4	Y	664.4	332.7	647.3	646.4	6
8	791.4	396.2		773.4	G	501.3		484.3	483.3	5
9	878.5	439.7		860.5	S	444.3		427.3	426.3	4
10	991.6	496.3		973.5	I	357.3		340.2		3
11	1088.6	544.8		1070.6	P	244.2		227.1		2
12	1234.7	617.9	1217.7	1216.7	K	147.1		130.1		1

Gene symbol: Slc25a22

Protein name: Mitochondrial glutamate carrier 1

Protein accession numbers: IPI00109275,IPI00756073

Peptide sequence: (R)GVNEDTYSGFLDCAR(K)

Exclusive (unique to this protein): TRUE

SEQUEST XCorr score: 2.53 SEQUEST DCn score: 0.65

Fix modifications: C13: Carbamidomethyl (+57.02)Variable modifications: None

charge: 2 Observed m/z: 852.3712

Actual minus calculated peptide mass (AMU): 0.0009766

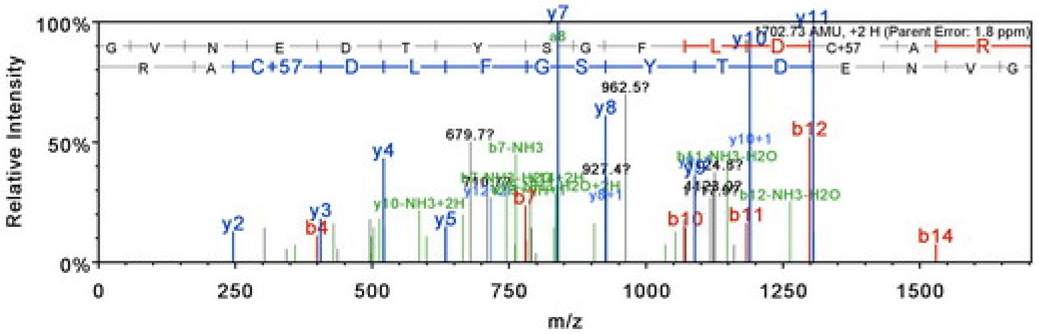

B	B Ions	B+2H	B-NH3	B-H2O	AA	Y Ions	Y+2H	Y-NH3	Y-H2O	Y
1	58.0				G	1703.7	852.4	1686.7	1685.7	15
2	157.1				V	1646.7	823.9	1629.7	1628.7	14
3	271.1		254.1		N	1547.6	774.3	1530.6	1529.6	13
4	400.2		383.2	382.2	E	1433.6	717.3	1416.6	1415.6	12
5	515.2		498.2	497.2	D	1304.6	652.8	1287.5	1286.5	11
6	616.3	308.6	599.2	598.3	T	1189.5	595.3	1172.5	1171.5	10
7	779.3	390.2	762.3	761.3	Y	1088.5	544.8	1071.5	1070.5	9
8	866.4	433.7	849.3	848.3	S	925.4	463.2	908.4	907.4	8
9	923.4	462.2	906.4	905.4	G	838.4	419.7	821.4	820.4	7
10	1070.4	535.7	1053.4	1052.4	F	781.4	391.2	764.3	763.4	6
11	1183.5	592.3	1166.5	1165.5	L	634.3		617.3	616.3	5
12	1298.5	649.8	1281.5	1280.5	D	521.2		504.2	503.2	4
13	1458.6	729.8	1441.6	1440.6	C+57	406.2		389.2		3
14	1529.6	765.3	1512.6	1511.6	A	246.2		229.1		2
15	1703.7	852.4	1686.7	1685.7	R	175.1		158.1		1

Gene symbol: Slc25a35
Protein name: Solute carrier family 25 member 35
Protein accession numbers: IPI00111884,IPI00648190
Peptide sequence: (R)MQLQGELQAPGTYQR(H)
Exclusive (unique to this protein): TRUE
SEQUEST XCorr score: 2.23 SEQUEST DCn score: 0.534
Fix modifications: None
Variable modifications: None
charge: 2 Observed m/z: 860.4302
Actual minus calculated peptide mass (AMU): 0.003418

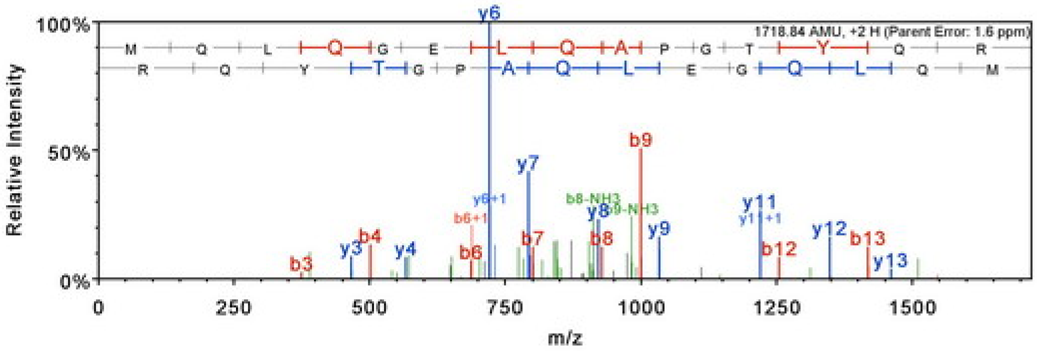

B	B Ions	B+2H	B-NH3	B-H2O	AA	Y Ions	Y+2H	Y-NH3	Y-H2O	Y
1	132.1				M	1719.9	860.4	1702.8	1701.8	15
2	260.1		243.1		Q	1588.8	794.9	1571.8	1570.8	14
3	373.2		356.2		L	1460.8	730.9	1443.7	1442.7	13
4	501.3		484.2		Q	1347.7	674.3	1330.6	1329.7	12
5	558.3		541.2		G	1219.6	610.3	1202.6	1201.6	11
6	687.3	344.2	670.3	669.3	E	1162.6	581.8	1145.6	1144.6	10
7	800.4	400.7	783.4	782.4	L	1033.5	517.3	1016.5	1015.5	9
8	928.5	464.7	911.4	910.5	Q	920.5	460.7	903.4	902.5	8
9	999.5	500.3	982.5	981.5	A	792.4	396.7	775.4	774.4	7
10	1096.5	548.8	1079.5	1078.5	P	721.4	361.2	704.3	703.4	6
11	1153.6	577.3	1136.5	1135.6	G	624.3		607.3	606.3	5
12	1254.6	627.8	1237.6	1236.6	T	567.3		550.3	549.3	4
13	1417.7	709.3	1400.7	1399.7	Y	466.2		449.2		3
14	1545.7	773.4	1528.7	1527.7	Q	303.2		286.2		2
15	1719.9	860.4	1702.8	1701.8	R	175.1		158.1		1

Gene symbol: Slc25a4
Protein name: ADP/ATP translocase 1
Protein accession numbers: IPI00115564,IPI00676622
Peptide sequence: (R)AAYFGVYDTAK(G)
Exclusive (unique to this protein): TRUE
SEQUEST XCorr score: 2.18 SEQUEST DCn score: 0.627
Fix modifications: None

Variable modifications: None

charge: 2 Observed m/z: 603.2962

Actual minus calculated peptide mass (AMU): 0.0002441

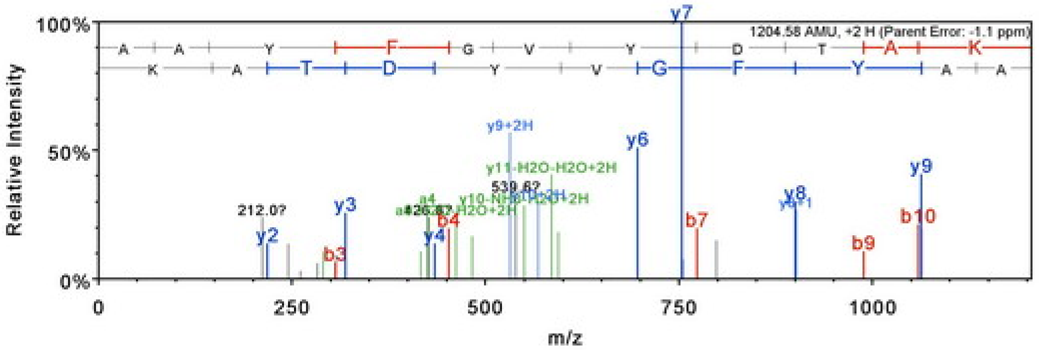

B	B Ions	B+2H	B-NH3	B-H2O	AA	Y Ions	Y+2H	Y-NH3	Y-H2O	Y
1	72.0				A	1205.6	603.3	1188.6	1187.6	11
2	143.1				A	1134.5	567.8	1117.5	1116.5	10
3	306.2				Y	1063.5	532.3	1046.5	1045.5	9
4	453.2				F	900.5	450.7	883.4	882.4	8
5	510.2				G	753.4	377.2	736.4	735.4	7
6	609.3	305.2			V	696.4	348.7	679.3	678.4	6
7	772.4	386.7			Y	597.3		580.3	579.3	5
8	887.4	444.2		869.4	D	434.2		417.2	416.2	4
9	988.4	494.7		970.4	T	319.2		302.2	301.2	3
10	1059.5	530.2		1041.5	A	218.2		201.1		2
11	1205.6	603.3	1188.6	1187.6	K	147.1		130.1		1

Gene symbol: Snrpd1

Protein name: Small nuclear ribonucleoprotein Sm D1

Protein accession numbers: IPI00322749

Peptide sequence: (K)NREPVQLETLSIR(G)

Exclusive (unique to this protein): TRUE

SEQUEST XCorr score: 2.29 SEQUEST DCn score: 0.416

Fix modifications: None

Variable modifications: None

charge: 2 Observed m/z: 777.9357

Actual minus calculated peptide mass (AMU): 0.003052

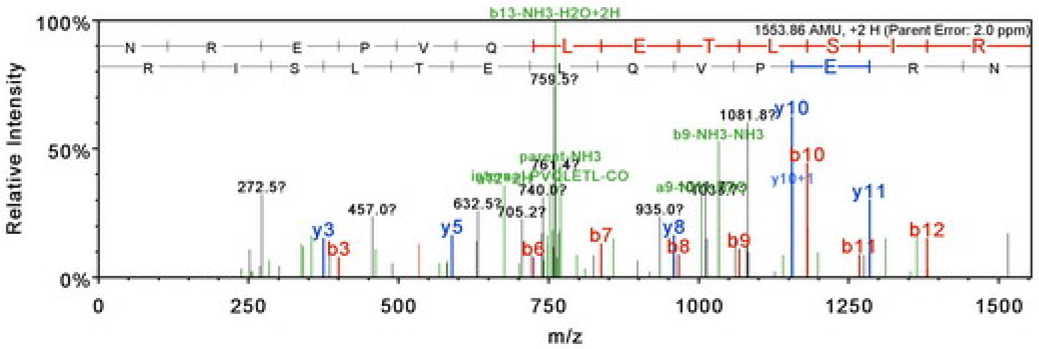

B	B Ions	B+2H	B-NH3	B-H2O	AA	Y Ions	Y+2H	Y-NH3	Y-H2O	Y
1	115.1		98.0		N	1554.9	777.9	1537.8	1536.9	13
2	271.1	136.1	254.1		R	1440.8	720.9	1423.8	1422.8	12
3	400.2	200.6	383.2	382.2	E	1284.7	642.9	1267.7	1266.7	11
4	497.3	249.1	480.2	479.2	P	1155.7	578.3	1138.7	1137.7	10
5	596.3	298.7	579.3	578.3	V	1058.6	529.8	1041.6	1040.6	9
6	724.4	362.7	707.4	706.4	Q	959.6	480.3	942.5	941.5	8
7	837.5	419.2	820.4	819.5	L	831.5	416.3	814.5	813.5	7
8	966.5	483.8	949.5	948.5	E	718.4	359.7	701.4	700.4	6
9	1067.5	534.3	1050.5	1049.5	T	589.4		572.3	571.4	5
10	1180.6	590.8	1163.6	1162.6	L	488.3		471.3	470.3	4
11	1267.7	634.3	1250.6	1249.7	S	375.2		358.2	357.2	3
12	1380.8	690.9	1363.7	1362.7	I	288.2		271.2		2
13	1554.9	777.9	1537.8	1536.9	R	175.1		158.1		1

Gene symbol: Snrpd3

Protein name: Small nuclear ribonucleoprotein Sm D3

Protein accession numbers: IPI00119224

Peptide sequence: (R)VAQLEQVVYIR(G)

Exclusive (unique to this protein): TRUE

SEQUEST XCorr score: 2.58 SEQUEST DCn score: 0.565

Fix modifications: None

Variable modifications: None

charge: 2 Observed m/z: 609.8461

Actual minus calculated peptide mass (AMU): -0.0003662

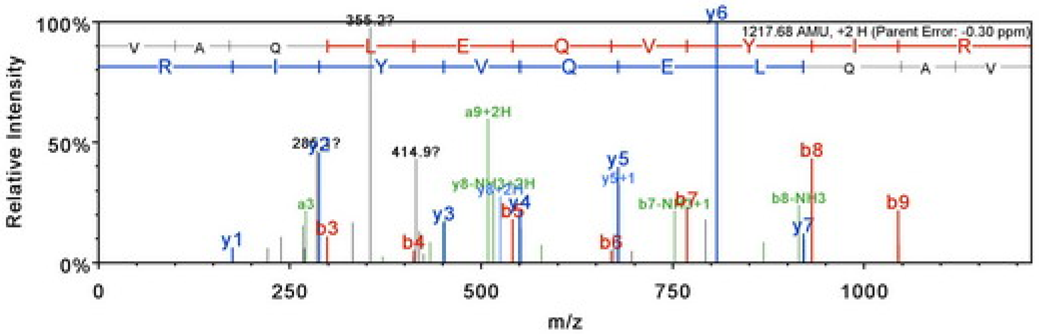

B	B Ions	B+2H	B-NH3	B-H2O	AA	Y Ions	Y+2H	Y-NH3	Y-H2O	Y
1	100.1				V	1218.7	609.9	1201.7	1200.7	10
2	171.1				A	1119.6	560.3	1102.6	1101.6	9
3	299.2		282.2		Q	1048.6	524.8	1031.5	1030.6	8
4	412.3		395.2		L	920.5	460.8	903.5	902.5	7
5	541.3		524.3	523.3	E	807.4	404.2	790.4	789.4	6
6	669.4	335.2	652.3	651.4	Q	678.4		661.4		5
7	768.4	384.7	751.4	750.4	V	550.3		533.3		4
8	931.5	466.3	914.5	913.5	Y	451.3		434.2		3
9	1044.6	522.8	1027.5	1026.6	I	288.2		271.2		2
10	1218.7	609.9	1201.7	1200.7	R	175.1		158.1		1

Gene symbol: Snx12

Protein name: 10 day old male pancreas cDNA, RIKEN full-length enriched library, clone:1810035A22 product:sorting nexin 12, full insert sequence

Protein accession numbers: IPI00468902,IPI00762269

Peptide sequence: (R)GDEGIFEESFIEER(R)

Exclusive (unique to this protein): TRUE

SEQUEST XCorr score: 2.05 SEQUEST DCn score: 0.707

Fix modifications: None

Variable modifications: None

charge: 2 Observed m/z: 828.8754

Actual minus calculated peptide mass (AMU): 0.003418

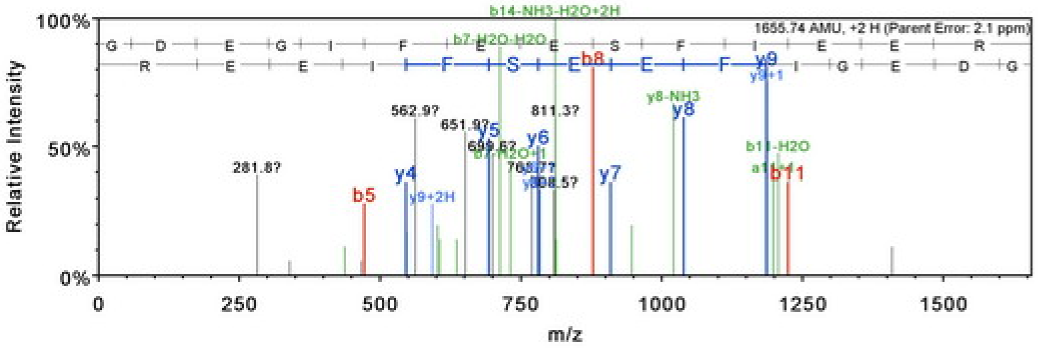

B	B Ions	B+2H	B-NH3	B-H2O	AA	Y Ions	Y+2H	Y-NH3	Y-H2O	Y
1	58.0				G	1656.7	828.9	1639.7	1638.7	14
2	173.1			155.1	D	1599.7	800.4	1582.7	1581.7	13
3	302.1			284.1	E	1484.7	742.9	1467.7	1466.7	12
4	359.1			341.1	G	1355.7	678.3	1338.6	1337.6	11
5	472.2			454.2	I	1298.6	649.8	1281.6	1280.6	10
6	619.3	310.1		601.3	F	1185.5	593.3	1168.5	1167.5	9
7	748.3	374.7		730.3	E	1038.5	519.7	1021.5	1020.5	8
8	877.4	439.2		859.4	E	909.4	455.2	892.4	891.4	7
9	964.4	482.7		946.4	S	780.4	390.7	763.4	762.4	6
10	1111.5	556.2		1093.5	F	693.4		676.3	675.4	5
11	1224.5	612.8		1206.5	I	546.3		529.3	528.3	4
12	1353.6	677.3		1335.6	E	433.2		416.2	415.2	3
13	1482.6	741.8		1464.6	E	304.2		287.1	286.2	2
14	1656.7	828.9	1639.7	1638.7	R	175.1		158.1		1

Gene symbol: Sod1

Protein name: Superoxide dismutase

Protein accession numbers: IPI00130589

Peptide sequence: (R)VISLSGEHSIIGR(T)

Exclusive (unique to this protein): TRUE

SEQUEST XCorr score: 2.98 SEQUEST DCn score: 0.607

Fix modifications: None

Variable modifications: None

charge: 2 Observed m/z: 684.3865

Actual minus calculated peptide mass (AMU): 0.0004883

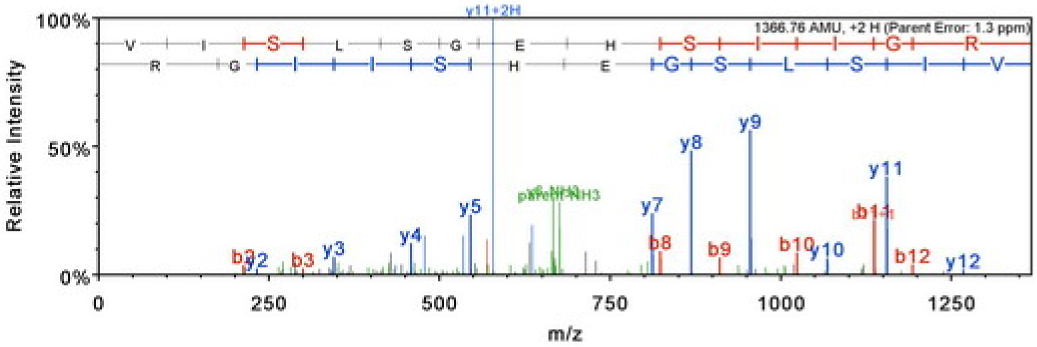

B	B Ions	B+2H	B-NH3	B-H2O	AA	Y Ions	Y+2H	Y-NH3	Y-H2O	Y
1	100.1				V	1367.8	684.4	1350.7	1349.8	13
2	213.2				I	1268.7	634.9	1251.7	1250.7	12
3	300.2			282.2	S	1155.6	578.3	1138.6	1137.6	11
4	413.3			395.3	L	1068.6	534.8	1051.5	1050.6	10
5	500.3			482.3	S	955.5	478.3	938.5	937.5	9
6	557.3	279.2		539.3	G	868.5	434.7	851.4	850.5	8
7	686.4	343.7		668.4	E	811.4	406.2	794.4	793.4	7
8	823.4	412.2		805.4	H	682.4	341.7	665.4	664.4	6
9	910.5	455.7		892.5	S	545.3		528.3	527.3	5
10	1023.6	512.3		1005.5	I	458.3		441.3		4
11	1136.6	568.8		1118.6	I	345.2		328.2		3
12	1193.7	597.3		1175.6	G	232.1		215.1		2
13	1367.8	684.4	1350.7	1349.8	R	175.1		158.1		1

Gene symbol: Sox15

Protein name: SOX-15 protein

Protein accession numbers: IPI00118219

Peptide sequence: (K)RPMNAFMVWSSVQR(R)

Exclusive (unique to this protein): TRUE

SEQUEST XCorr score: 2.15 SEQUEST DCn score: 0.292

Fix modifications: None

Variable modifications: M3: Oxidation (+16.00)

charge: 2 Observed m/z: 862.9185

Actual minus calculated peptide mass (AMU): -0.0070758

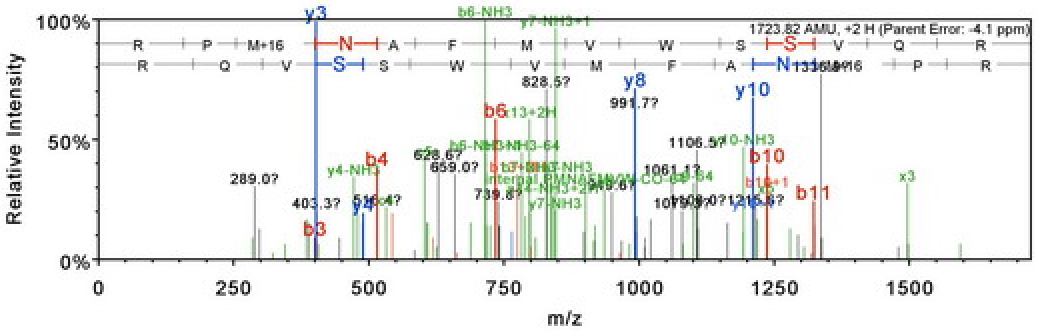

B	B Ions	B+2H	B-NH3	B-H2O	AA	Y Ions	Y+2H	Y-NH3	Y-H2O	Y
1	157.1	79.1	140.1		R	1724.8	862.9	1707.8	1706.8	14
2	254.2	127.6	237.1		P	1568.7	784.9	1551.7	1550.7	13
3	401.2	201.1	384.2		M+16	1471.7	736.4	1454.7	1453.7	12
4	515.2	258.1	498.2		N	1324.7	662.8	1307.6	1306.6	11
5	586.3	293.6	569.3		A	1210.6	605.8	1193.6	1192.6	10
6	733.4	367.2	716.3		F	1139.6	570.3	1122.5	1121.6	9
7	864.4	432.7	847.4		M	992.5	496.8	975.5	974.5	8
8	963.5	482.2	946.4		V	861.5	431.2	844.4	843.5	7
9	1149.5	575.3	1132.5		W	762.4	381.7	745.4	744.4	6
10	1236.6	618.8	1219.5	1218.6	S	576.3		559.3	558.3	5
11	1323.6	662.3	1306.6	1305.6	S	489.3		472.3	471.3	4
12	1422.7	711.8	1405.6	1404.7	V	402.3		385.2		3
13	1550.7	775.9	1533.7	1532.7	Q	303.2		286.2		2
14	1724.8	862.9	1707.8	1706.8	R	175.1		158.1		1

Gene symbol: Ssbp1

Protein name: single-stranded DNA binding protein 1 isoform 1

Protein accession numbers: IPI00408243

Peptide sequence: (R)QATTIIADNIIFLSDQTK(E)

Exclusive (unique to this protein): TRUE

SEQUEST XCorr score: 2.12 SEQUEST DCn score: 0.341

Fix modifications: None

Variable modifications: None

charge: 2 Observed m/z: 996.5334

Actual minus calculated peptide mass (AMU): -0.0067152

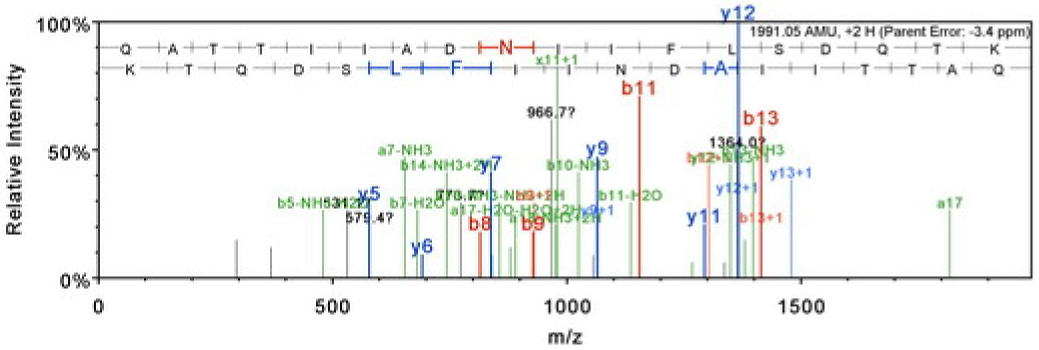

B	B Ions	B+2H	B-NH3	B-H2O	AA	Y Ions	Y+2H	Y-NH3	Y-H2O	Y
1	129.1		112.0		Q	1992.1	996.5	1975.0	1974.1	18
2	200.1		183.1		A	1864.0	932.5	1847.0	1846.0	17
3	301.2		284.1	283.1	T	1793.0	897.0	1775.9	1775.0	16
4	402.2		385.2	384.2	T	1691.9	846.5	1674.9	1673.9	15
5	515.3		498.3	497.3	I	1590.9	795.9	1573.9	1572.9	14
6	628.4	314.7	611.3	610.4	I	1477.8	739.4	1460.8	1459.8	13
7	699.4	350.2	682.4	681.4	A	1364.7	682.9	1347.7	1346.7	12
8	814.4	407.7	797.4	796.4	D	1293.7	647.3	1276.6	1275.7	11
9	928.5	464.7	911.5	910.5	N	1178.6	589.8	1161.6	1160.6	10
10	1041.6	521.3	1024.5	1023.6	I	1064.6	532.8	1047.6	1046.6	9
11	1154.6	577.8	1137.6	1136.6	I	951.5	476.3	934.5	933.5	8
12	1301.7	651.4	1284.7	1283.7	F	838.4	419.7	821.4	820.4	7
13	1414.8	707.9	1397.8	1396.8	L	691.4	346.2	674.3	673.4	6
14	1501.8	751.4	1484.8	1483.8	S	578.3		561.3	560.3	5
15	1616.9	808.9	1599.8	1598.8	D	491.3		474.2	473.2	4
16	1744.9	873.0	1727.9	1726.9	Q	376.2		359.2	358.2	3
17	1846.0	923.5	1828.9	1828.0	T	248.2		231.1	230.2	2
18	1992.1	996.5	1975.0	1974.1	K	147.1		130.1		1

Gene symbol: Ssr4

Protein name: 10 day old male pancreas cDNA, RIKEN full-length enriched library, clone:1810061C01 product:signal sequence receptor, delta, full insert sequence

Protein accession numbers: IPI00122346

Peptide sequence: (R)FFDEESYSLLR(K)

Exclusive (unique to this protein): TRUE

SEQUEST XCorr score: 2.48 SEQUEST DCn score: 0.649

Fix modifications: None

Variable modifications: None

charge: 2 Observed m/z: 703.3366

Actual minus calculated peptide mass (AMU): 0.001343

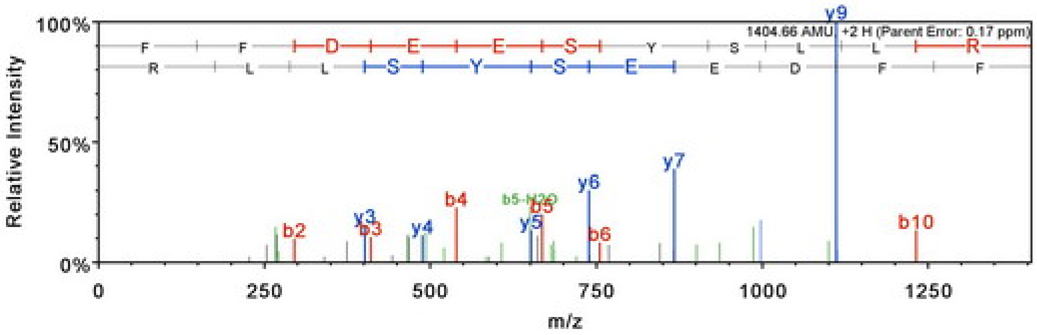

B	B Ions	B+2H	B-NH3	B-H2O	AA	Y Ions	Y+2H	Y-NH3	Y-H2O	Y
1	148.1				F	1405.7	703.3	1388.6	1387.7	11
2	295.1				F	1258.6	629.8	1241.6	1240.6	10
3	410.2			392.2	D	1111.5	556.3	1094.5	1093.5	9
4	539.2			521.2	E	996.5	498.8	979.5	978.5	8
5	668.3			650.3	E	867.5	434.2	850.4	849.5	7
6	755.3	378.2		737.3	S	738.4	369.7	721.4	720.4	6
7	918.4	459.7		900.3	Y	651.4		634.4	633.4	5
8	1005.4	503.2		987.4	S	488.3		471.3	470.3	4
9	1118.5	559.7		1100.5	L	401.3		384.3		3
10	1231.5	616.3		1213.5	L	288.2		271.2		2
11	1405.7	703.3	1388.6	1387.7	R	175.1		158.1		1

Gene symbol: Stip1

Protein name: Stress-induced-phosphoprotein 1

Protein accession numbers: IPI00121514

Peptide sequence: (R)LAYINPDLALEEK(N)

Exclusive (unique to this protein): TRUE

SEQUEST XCorr score: 2.47 SEQUEST DCn score: 0.469

Fix modifications: None

Variable modifications: None

charge: 2 Observed m/z: 744.8987

Actual minus calculated peptide mass (AMU): -0.005493

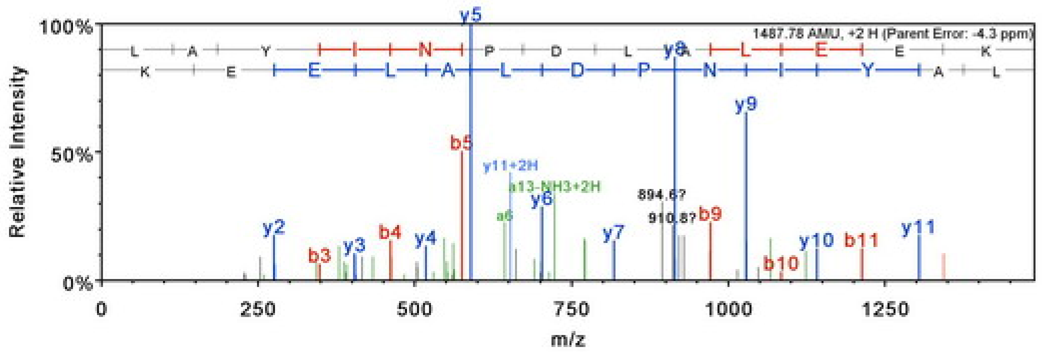

B	B Ions	B+2H	B-NH3	B-H2O	AA	Y Ions	Y+2H	Y-NH3	Y-H2O	Y
1	114.1				L	1488.8	744.9	1471.8	1470.8	13
2	185.1				A	1375.7	688.4	1358.7	1357.7	12
3	348.2				Y	1304.7	652.8	1287.7	1286.7	11
4	461.3				I	1141.6	571.3	1124.6	1123.6	10
5	575.3		558.3		N	1028.5	514.8	1011.5	1010.5	9
6	672.4	336.7	655.4		P	914.5	457.8	897.5	896.5	8
7	787.4	394.2	770.4	769.4	D	817.4	409.2	800.4	799.4	7
8	900.5	450.8	883.5	882.5	L	702.4	351.7	685.4	684.4	6
9	971.5	486.3	954.5	953.5	A	589.3		572.3	571.3	5
10	1084.6	542.8	1067.6	1066.6	L	518.3		501.3	500.3	4
11	1213.7	607.3	1196.6	1195.6	E	405.2		388.2	387.2	3
12	1342.7	671.9	1325.7	1324.7	E	276.2		259.1	258.1	2
13	1488.8	744.9	1471.8	1470.8	K	147.1		130.1		1

Gene symbol: Stx12

Protein name: Syntaxin-12

Protein accession numbers: IPI00111416

Peptide sequence: (R)DFNSIIQTCSGNIQR(I)

Exclusive (unique to this protein): TRUE

SEQUEST XCorr score: 2.41 SEQUEST DCn score: 0.433

Fix modifications: C9: Carbamidomethyl (+57.02)Variable modifications: None

charge: 2 Observed m/z: 876.9184

Actual minus calculated peptide mass (AMU): -0.005127

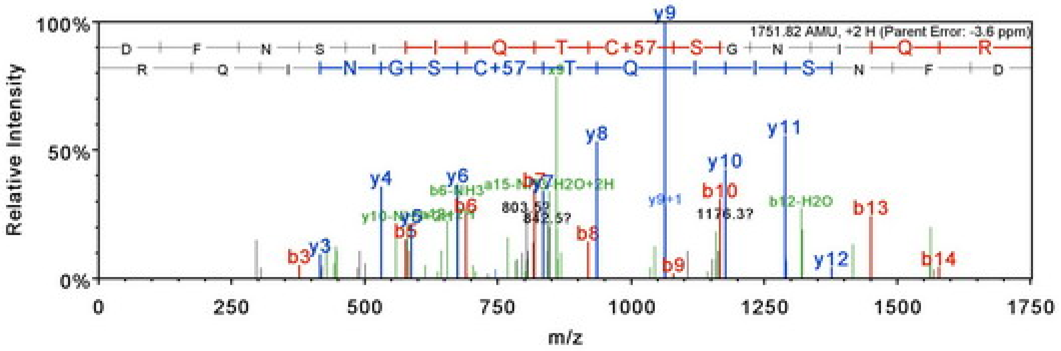

B	B Ions	B+2H	B-NH3	B-H2O	AA	Y Ions	Y+2H	Y-NH3	Y-H2O	Y
1	116.0			98.0	D	1752.8	876.9	1735.8	1734.8	15
2	263.1			245.1	F	1637.8	819.4	1620.8	1619.8	14
3	377.2		360.1	359.1	N	1490.7	745.9	1473.7	1472.7	13
4	464.2		447.2	446.2	S	1376.7	688.9	1359.7	1358.7	12
5	577.3		560.2	559.3	I	1289.7	645.3	1272.6	1271.7	11
6	690.4	345.7	673.3	672.3	I	1176.6	588.8	1159.5	1158.6	10
7	818.4	409.7	801.4	800.4	Q	1063.5	532.3	1046.5	1045.5	9
8	919.5	460.2	902.4	901.4	T	935.4	468.2	918.4	917.4	8
9	1079.5	540.3	1062.5	1061.5	C+57	834.4	417.7	817.4	816.4	7
10	1166.5	583.8	1149.5	1148.5	S	674.4	337.7	657.3	656.4	6
11	1223.5	612.3	1206.5	1205.5	G	587.3		570.3		5
12	1337.6	669.3	1320.5	1319.6	N	530.3		513.3		4
13	1450.7	725.8	1433.6	1432.7	I	416.3		399.2		3
14	1578.7	789.9	1561.7	1560.7	Q	303.2		286.2		2
15	1752.8	876.9	1735.8	1734.8	R	175.1		158.1		1

Gene symbol: Surf4

Protein name: Surfeit locus protein 4

Protein accession numbers: IPI00133249

Peptide sequence: (R)LCLISTFLEDGIR(M)

Exclusive (unique to this protein): TRUE

SEQUEST XCorr score: 2.21 SEQUEST DCn score: 0.64

Fix modifications: C2: Carbamidomethyl (+57.02)Variable modifications: None

charge: 2 Observed m/z: 768.9055

Actual minus calculated peptide mass (AMU): -0.006836

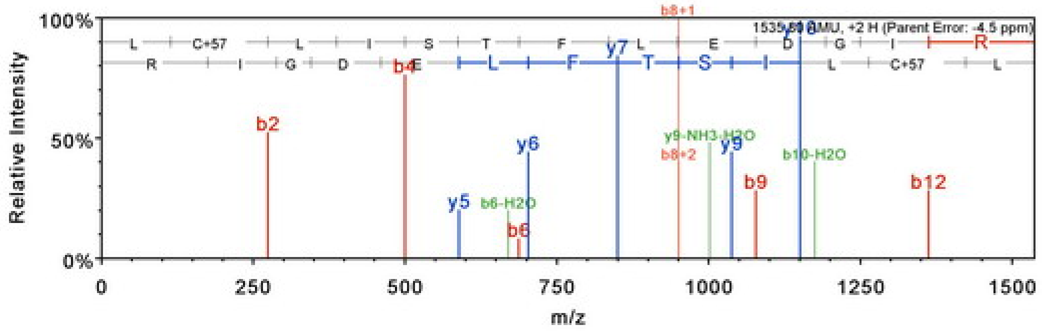

B	B Ions	B+2H	B-NH3	B-H2O	AA	Y Ions	Y+2H	Y-NH3	Y-H2O	Y
1	114.1				L	1536.8	768.9	1519.8	1518.8	13
2	274.1				C+57	1423.7	712.4	1406.7	1405.7	12
3	387.2				L	1263.7	632.4	1246.7	1245.7	11
4	500.3				I	1150.6	575.8	1133.6	1132.6	10
5	587.3			569.3	S	1037.5	519.3	1020.5	1019.5	9
6	688.4	344.7		670.4	T	950.5	475.8	933.5	932.5	8
7	835.4	418.2		817.4	F	849.5	425.2	832.4	831.4	7
8	948.5	474.8		930.5	L	702.4	351.7	685.4	684.4	6
9	1077.6	539.3		1059.6	E	589.3		572.3	571.3	5
10	1192.6	596.8		1174.6	D	460.3		443.2	442.2	4
11	1249.6	625.3		1231.6	G	345.2		328.2		3
12	1362.7	681.9		1344.7	I	288.2		271.2		2
13	1536.8	768.9	1519.8	1518.8	R	175.1		158.1		1

Gene symbol: Tatdn2

Protein name: Activated spleen cDNA, RIKEN full-length enriched library, clone:F830011G01 product:similar to Putative deoxyribonuclease KIAA0218

Protein accession numbers: IPI00622729,IPI00762372

Peptide sequence: (R)HPKAVAFGEMGLDYSHK(C)

Exclusive (unique to this protein): TRUE

SEQUEST XCorr score: 2.04 SEQUEST DCn score: 0.377

Fix modifications: None

Variable modifications: None

charge: 2 Observed m/z: 943.9716

Actual minus calculated peptide mass (AMU): 0.01285

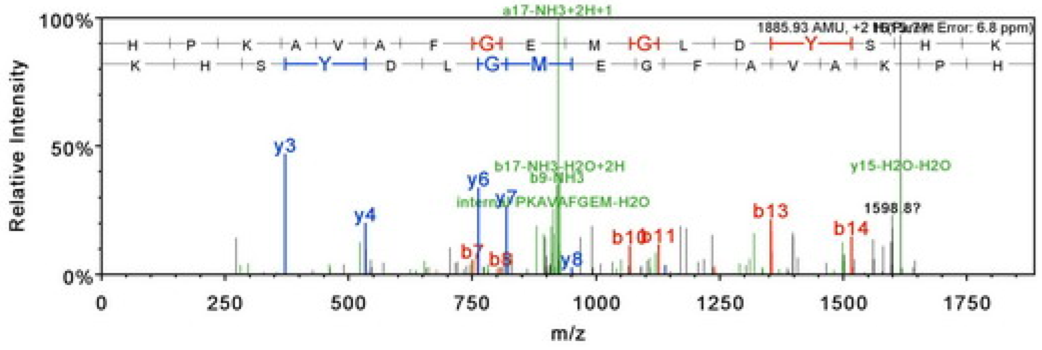

B	B Ions	B+2H	B-NH3	B-H2O	AA	Y Ions	Y+2H	Y-NH3	Y-H2O	Y
1	138.1	69.5			H	1886.9	944.0	1869.9	1868.9	17
2	235.1	118.1			P	1749.9	875.4	1732.8	1731.9	16
3	363.2	182.1	346.2		K	1652.8	826.9	1635.8	1634.8	15
4	434.3	217.6	417.2		A	1524.7	762.9	1507.7	1506.7	14
5	533.3	267.2	516.3		V	1453.7	727.3	1436.7	1435.7	13
6	604.4	302.7	587.3		A	1354.6	677.8	1337.6	1336.6	12
7	751.4	376.2	734.4		F	1283.6	642.3	1266.5	1265.6	11
8	808.5	404.7	791.4		G	1136.5	568.8	1119.5	1118.5	10
9	937.5	469.3	920.5	919.5	E	1079.5	540.3	1062.5	1061.5	9
10	1068.5	534.8	1051.5	1050.5	M	950.4	475.7	933.4	932.4	8
11	1125.5	563.3	1108.5	1107.5	G	819.4	410.2	802.4	801.4	7
12	1238.6	619.8	1221.6	1220.6	L	762.4	381.7	745.4	744.4	6
13	1353.7	677.3	1336.6	1335.7	D	649.3	325.2	632.3	631.3	5
14	1516.7	758.9	1499.7	1498.7	Y	534.3	267.6	517.2	516.3	4
15	1603.8	802.4	1586.7	1585.8	S	371.2	186.1	354.2	353.2	3
16	1740.8	870.9	1723.8	1722.8	H	284.2	142.6	267.1		2
17	1886.9	944.0	1869.9	1868.9	K	147.1		130.1		1

Gene symbol: Tbcc

Protein name: Tubulin-specific chaperone C

Protein accession numbers: IPI00122348

Peptide sequence: (R)QGQAALAQLQAVLTER(R)

Exclusive (unique to this protein): TRUE

SEQUEST XCorr score: 2.28 SEQUEST DCn score: 0.468

Fix modifications: None

Variable modifications: None

charge: 2 Observed m/z: 848.9725

Actual minus calculated peptide mass (AMU): 0.002319

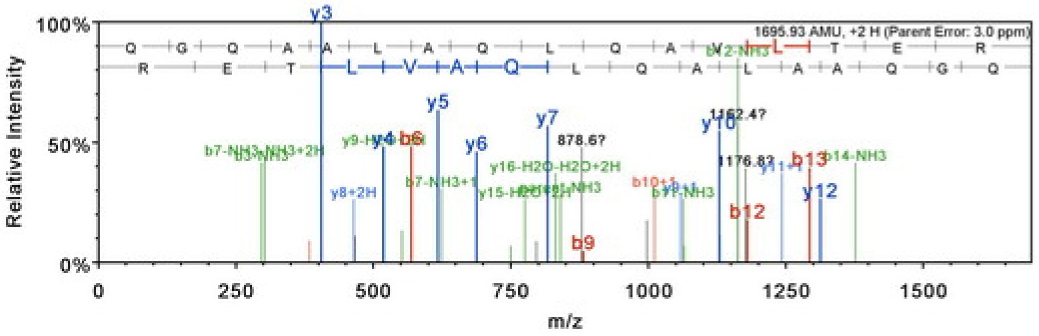

B	B Ions	B+2H	B-NH3	B-H2O	AA	Y Ions	Y+2H	Y-NH3	Y-H2O	Y
1	129.1		112.0		Q	1696.9	849.0	1679.9	1678.9	16
2	186.1		169.1		G	1568.9	784.9	1551.9	1550.9	15
3	314.2		297.1		Q	1511.9	756.4	1494.8	1493.8	14
4	385.2		368.2		A	1383.8	692.4	1366.8	1365.8	13
5	456.2		439.2		A	1312.8	656.9	1295.7	1294.8	12
6	569.3	285.2	552.3		L	1241.7	621.4	1224.7	1223.7	11
7	640.3	320.7	623.3		A	1128.6	564.8	1111.6	1110.6	10
8	768.4	384.7	751.4		Q	1057.6	529.3	1040.6	1039.6	9
9	881.5	441.3	864.5		L	929.5	465.3	912.5	911.5	8
10	1009.5	505.3	992.5		Q	816.5	408.7	799.4	798.5	7
11	1080.6	540.8	1063.5		A	688.4	344.7	671.4	670.4	6
12	1179.7	590.3	1162.6		V	617.4		600.3	599.4	5
13	1292.7	646.9	1275.7		L	518.3		501.3	500.3	4
14	1393.8	697.4	1376.8	1375.8	T	405.2		388.2	387.2	3
15	1522.8	761.9	1505.8	1504.8	E	304.2		287.1	286.2	2
16	1696.9	849.0	1679.9	1678.9	R	175.1		158.1		1

Gene symbol: Tceb2

Protein name: Transcription elongation factor B polypeptide 2

Protein accession numbers: IPI00131224

Peptide sequence: (R)PQAPATVGLAFR(A)

Exclusive (unique to this protein): TRUE

SEQUEST XCorr score: 2.12 SEQUEST DCn score: 0.749

Fix modifications: None

Variable modifications: None

charge: 2 Observed m/z: 614.3461

Actual minus calculated peptide mass (AMU): -0.0007324

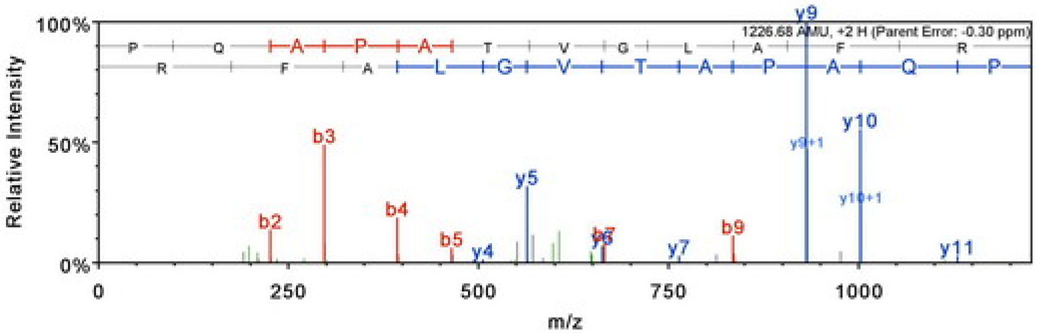

B	B Ions	B+2H	B-NH3	B-H2O	AA	Y Ions	Y+2H	Y-NH3	Y-H2O	Y
1	98.1				P	1227.7	614.4	1210.7	1209.7	12
2	226.1		209.1		Q	1130.6	565.8	1113.6	1112.6	11
3	297.2		280.1		A	1002.6	501.8	985.6	984.6	10
4	394.2		377.2		P	931.5	466.3	914.5	913.5	9
5	465.3		448.2		A	834.5	417.8	817.5	816.5	8
6	566.3	283.7	549.3	548.3	T	763.5	382.2	746.4	745.4	7
7	665.4	333.2	648.3	647.4	V	662.4	331.7	645.4		6
8	722.4	361.7	705.4	704.4	G	563.3		546.3		5
9	835.5	418.2	818.4	817.5	L	506.3		489.3		4
10	906.5	453.8	889.5	888.5	A	393.2		376.2		3
11	1053.6	527.3	1036.5	1035.6	F	322.2		305.2		2
12	1227.7	614.4	1210.7	1209.7	R	175.1		158.1		1

Gene symbol: Tcl1b1

Protein name: TCL1B1 protein

Protein accession numbers: IPI00116164

Peptide sequence: (R)NIYWATDGTHWR(L)

Exclusive (unique to this protein): TRUE

SEQUEST XCorr score: 2.86   SEQUEST DCn score: 0.591

Fix modifications: None

Variable modifications: None

charge: 2   Observed m/z: 760.3546

Actual minus calculated peptide mass (AMU): -0.006958

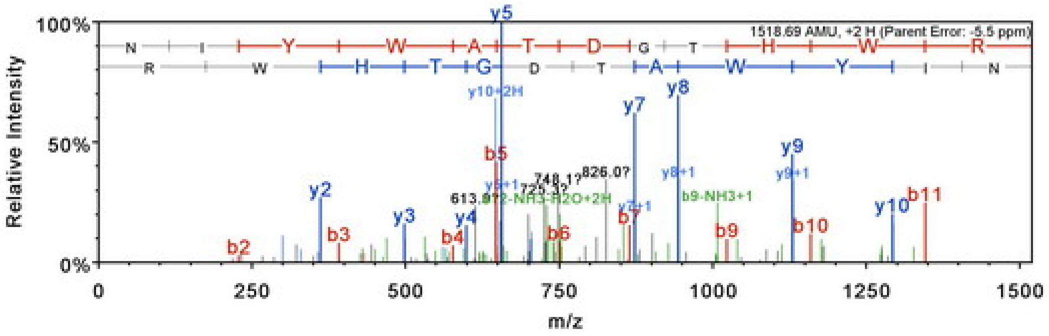

B	B Ions	B+2H	B-NH3	B-H2O	AA	Y Ions	Y+2H	Y-NH3	Y-H2O	Y
1	115.1		98.0		N	1519.7	760.4	1502.7	1501.7	12
2	228.1		211.1		I	1405.7	703.3	1388.6	1387.7	11
3	391.2		374.2		Y	1292.6	646.8	1275.5	1274.6	10
4	577.3		560.3		W	1129.5	565.3	1112.5	1111.5	9
5	648.3		631.3		A	943.4	472.2	926.4	925.4	8
6	749.4	375.2	732.3	731.4	T	872.4	436.7	855.4	854.4	7
7	864.4	432.7	847.4	846.4	D	771.4	386.2	754.3	753.3	6
8	921.4	461.2	904.4	903.4	G	656.3	328.7	639.3	638.3	5
9	1022.5	511.7	1005.4	1004.5	T	599.3	300.2	582.3	581.3	4
10	1159.5	580.3	1142.5	1141.5	H	498.3	249.6	481.2		3
11	1345.6	673.3	1328.6	1327.6	W	361.2		344.2		2
12	1519.7	760.4	1502.7	1501.7	R	175.1		158.1		1

Gene symbol: Tcl1b2

Protein name: TCL1B2 protein

Protein accession numbers: IPI00116165

Peptide sequence: (R)LLPQVLISTGPGFYEDEHHR(L)

Exclusive (unique to this protein): TRUE

SEQUEST XCorr score: 2.05 SEQUEST DCn score: 0.597

Fix modifications: None

Variable modifications: None

charge: 2 Observed m/z: 1154.5868

Actual minus calculated peptide mass (AMU): -0.0070758

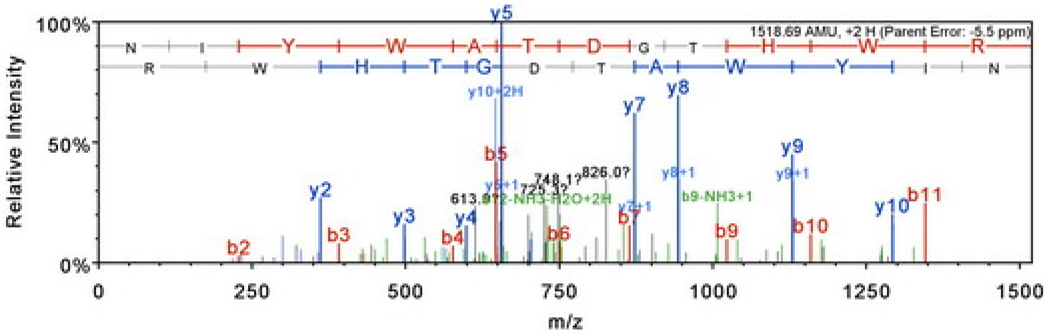

B	B Ions	B+2H	B-NH3	B-H2O	AA	Y Ions	Y+2H	Y-NH3	Y-H2O	Y
1	115.1		98.0		N	1519.7	760.4	1502.7	1501.7	12
2	228.1		211.1		I	1405.7	703.3	1388.6	1387.7	11
3	391.2		374.2		Y	1292.6	646.8	1275.5	1274.6	10
4	577.3		560.3		W	1129.5	565.3	1112.5	1111.5	9
5	648.3		631.3		A	943.4	472.2	926.4	925.4	8
6	749.4	375.2	732.3	731.4	T	872.4	436.7	855.4	854.4	7
7	864.4	432.7	847.4	846.4	D	771.4	386.2	754.3	753.3	6
8	921.4	461.2	904.4	903.4	G	656.3	328.7	639.3	638.3	5
9	1022.5	511.7	1005.4	1004.5	T	599.3	300.2	582.3	581.3	4
10	1159.5	580.3	1142.5	1141.5	H	498.3	249.6	481.2		3
11	1345.6	673.3	1328.6	1327.6	W	361.2		344.2		2
12	1519.7	760.4	1502.7	1501.7	R	175.1		158.1		1

Gene symbol: Them2

Protein name: Thioesterase superfamily member 2

Protein accession numbers: IPI00132958

Peptide sequence: (K)TLAFASVDLTNK(T)

Exclusive (unique to this protein): TRUE

SEQUEST XCorr score: 3.04 SEQUEST DCn score: 0.587

Fix modifications: None

Variable modifications: None

charge: 2 Observed m/z: 640.3446

Actual minus calculated peptide mass (AMU): -0.008423

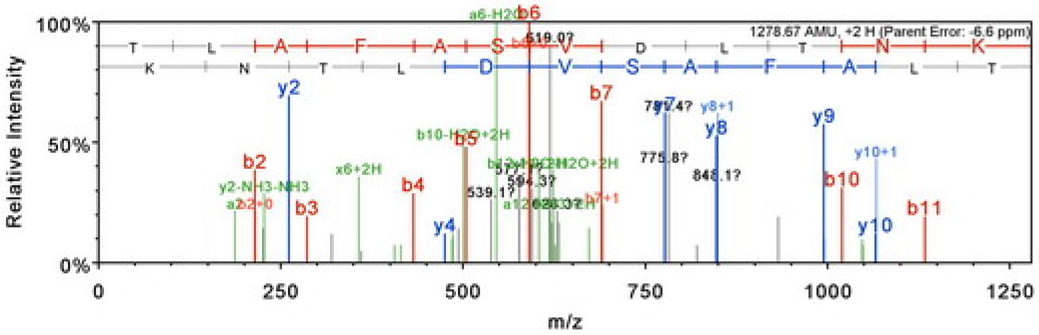

B	B Ions	B+2H	B-NH3	B-H2O	AA	Y Ions	Y+2H	Y-NH3	Y-H2O	Y
1	102.1			84.0	T	1279.7	640.4	1262.7	1261.7	12
2	215.1			197.1	L	1178.6	589.8	1161.6	1160.6	11
3	286.2			268.2	A	1065.6	533.3	1048.5	1047.5	10
4	433.3			415.2	F	994.5	497.8	977.5	976.5	9
5	504.3			486.3	A	847.5	424.2	830.4	829.4	8
6	591.3	296.2		573.3	S	776.4	388.7	759.4	758.4	7
7	690.4	345.7		672.4	V	689.4	345.2	672.4	671.4	6
8	805.4	403.2		787.4	D	590.3		573.3	572.3	5
9	918.5	459.8		900.5	L	475.3		458.3	457.3	4
10	1019.5	510.3		1001.5	T	362.2		345.2	344.2	3
11	1133.6	567.3	1116.6	1115.6	N	261.2		244.1		2
12	1279.7	640.4	1262.7	1261.7	K	147.1		130.1		1

Gene symbol: Timm44

Protein name: Import inner membrane translocase subunit TIM44, mitochondrial precursor

Protein accession numbers: IPI00135068

Peptide sequence: (K)ELDESVLGQTGPYR(R)

Exclusive (unique to this protein): TRUE

SEQUEST XCorr score: 2.02 SEQUEST DCn score: 0.557

Fix modifications: None

Variable modifications: None

charge: 2 Observed m/z: 782.3824

Actual minus calculated peptide mass (AMU): -0.008667

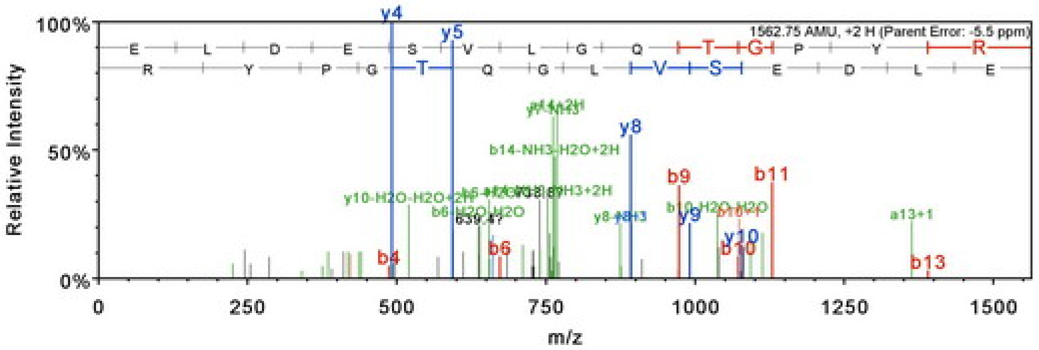

B	B Ions	B+2H	B-NH3	B-H2O	AA	Y Ions	Y+2H	Y-NH3	Y-H2O	Y
1	130.1			112.0	E	1563.8	782.4	1546.7	1545.8	14
2	243.1			225.1	L	1434.7	717.9	1417.7	1416.7	13
3	358.2			340.2	D	1321.6	661.3	1304.6	1303.6	12
4	487.2			469.2	E	1206.6	603.8	1189.6	1188.6	11
5	574.2			556.2	S	1077.6	539.3	1060.5	1059.6	10
6	673.3	337.2		655.3	V	990.5	495.8	973.5	972.5	9
7	786.4	393.7		768.4	L	891.5	446.2	874.4	873.5	8
8	843.4	422.2		825.4	G	778.4	389.7	761.4	760.4	7
9	971.5	486.2	954.4	953.5	Q	721.4	361.2	704.3	703.4	6
10	1072.5	536.8	1055.5	1054.5	T	593.3		576.3	575.3	5
11	1129.5	565.3	1112.5	1111.5	G	492.3		475.2		4
12	1226.6	613.8	1209.6	1208.6	P	435.2		418.2		3
13	1389.7	695.3	1372.6	1371.6	Y	338.2		321.2		2
14	1563.8	782.4	1546.7	1545.8	R	175.1		158.1		1

Gene symbol: Tmem109

Protein name: Transmembrane protein 109 precursor

Protein accession numbers: IPI00153101

Peptide sequence: (K)ETSADILTQIGR(S)

Exclusive (unique to this protein): TRUE

SEQUEST XCorr score: 2.17 SEQUEST DCn score: 0.482

Fix modifications: None

Variable modifications: None

charge: 2 Observed m/z: 652.3472

Actual minus calculated peptide mass (AMU): 0.0004883

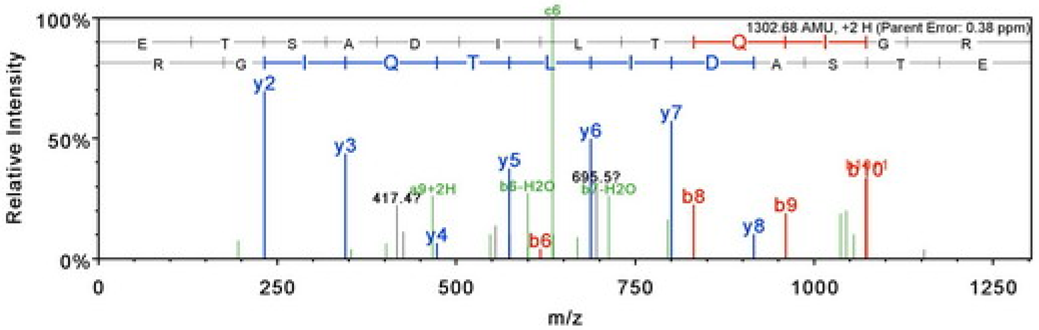

B	B Ions	B+2H	B-NH3	B-H2O	AA	Y Ions	Y+2H	Y-NH3	Y-H2O	Y
1	130.1			112.0	E	1303.7	652.4	1286.7	1285.7	12
2	231.1			213.1	T	1174.6	587.8	1157.6	1156.6	11
3	318.1			300.1	S	1073.6	537.3	1056.6	1055.6	10
4	389.2			371.2	A	986.6	493.8	969.5	968.6	9
5	504.2			486.2	D	915.5	458.3	898.5	897.5	8
6	617.3	309.1		599.3	I	800.5	400.8	783.5	782.5	7
7	730.4	365.7		712.4	L	687.4	344.2	670.4	669.4	6
8	831.4	416.2		813.4	T	574.3		557.3	556.3	5
9	959.5	480.2	942.4	941.5	Q	473.3		456.3		4
10	1072.5	536.8	1055.5	1054.5	I	345.2		328.2		3
11	1129.6	565.3	1112.5	1111.6	G	232.1		215.1		2
12	1303.7	652.4	1286.7	1285.7	R	175.1		158.1		1

Gene symbol: Tmem11
Protein name: Transmembrane protein 11
Protein accession numbers: IPI00222447
Peptide sequence: (R)LPLHTLTSSTPVVLVR(K)
Exclusive (unique to this protein): TRUE
SEQUEST XCorr score: 2.23 SEQUEST DCn score: 0.577
Fix modifications: None
Variable modifications: None
charge: 2 Observed m/z: 867.0162
Actual minus calculated peptide mass (AMU): -0.008057

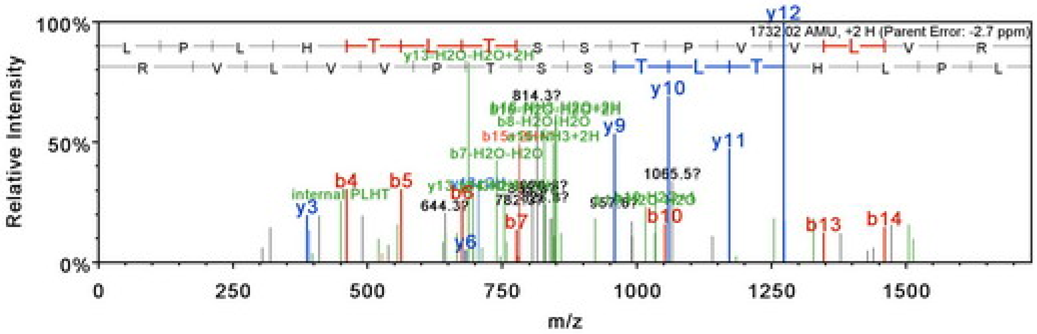

B	B Ions	B+2H	B-NH3	B-H2O	AA	Y Ions	Y+2H	Y-NH3	Y-H2O	Y
1	114.1				L	1733.0	867.0	1716.0	1715.0	16
2	211.1				P	1620.0	810.5	1602.9	1601.9	15
3	324.2				L	1522.9	762.0	1505.9	1504.9	14
4	461.3	231.2			H	1409.8	705.4	1392.8	1391.8	13
5	562.3	281.7		544.3	T	1272.8	636.9	1255.7	1254.7	12
6	675.4	338.2		657.4	L	1171.7	586.4	1154.7	1153.7	11
7	776.5	388.7		758.5	T	1058.6	529.8	1041.6	1040.6	10
8	863.5	432.3		845.5	S	957.6	479.3	940.6	939.6	9
9	950.5	475.8		932.5	S	870.5	435.8	853.5	852.5	8
10	1051.6	526.3		1033.6	T	783.5	392.3	766.5	765.5	7
11	1148.6	574.8		1130.6	P	682.5	341.7	665.4		6
12	1247.7	624.4		1229.7	V	585.4		568.4		5
13	1346.8	673.9		1328.8	V	486.3		469.3		4
14	1459.9	730.4		1441.8	L	387.3		370.3		3
15	1558.9	780.0		1540.9	V	274.2		257.2		2
16	1733.0	867.0	1716.0	1715.0	R	175.1		158.1		1

Gene symbol: Tmem14c
Protein name: Transmembrane protein 14C
Protein accession numbers: IPI00132768
Peptide sequence: (K)VGISLLSSPHP
Exclusive (unique to this protein): TRUE
SEQUEST XCorr score: 2.22 SEQUEST DCn score: 0.487
Fix modifications: None

Variable modifications: None

charge: 2 Observed m/z: 553.8102

Actual minus calculated peptide mass (AMU): -0.0085453

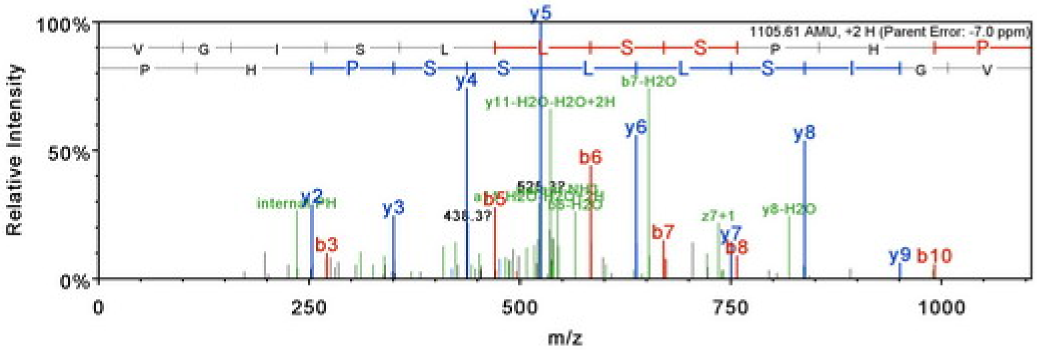

B	B Ions	B+2H	B-NH3	B-H2O	AA	Y Ions	Y+2H	Y-NH3	Y-H2O	Y
1	100.1				V	1106.6	553.8		1088.6	11
2	157.1				G	1007.6	504.3		989.5	10
3	270.2				I	950.5	475.8		932.5	9
4	357.2			339.2	S	837.5	419.2		819.4	8
5	470.3			452.3	L	750.4	375.7		732.4	7
6	583.4	292.2		565.4	L	637.3	319.2		619.3	6
7	670.4	335.7		652.4	S	524.3			506.2	5
8	757.5	379.2		739.4	S	437.2			419.2	4
9	854.5	427.8		836.5	P	350.2				3
10	991.6	496.3		973.6	H	253.1				2
11	1106.6	553.8		1088.6	P	116.1				1

Gene symbol: Tmem66

Protein name: transmembrane protein 66

Protein accession numbers: IPI00469002

Peptide sequence: (K)TVVSCEGYESSEDQYVLR(G)

Exclusive (unique to this protein): TRUE

SEQUEST XCorr score: 2.2 SEQUEST DCn score: 0.545

Fix modifications: C5: Carbamidomethyl (+57.02)Variable modifications: None

charge: 2 Observed m/z: 1060.9805

Actual minus calculated peptide mass (AMU): 0.008789

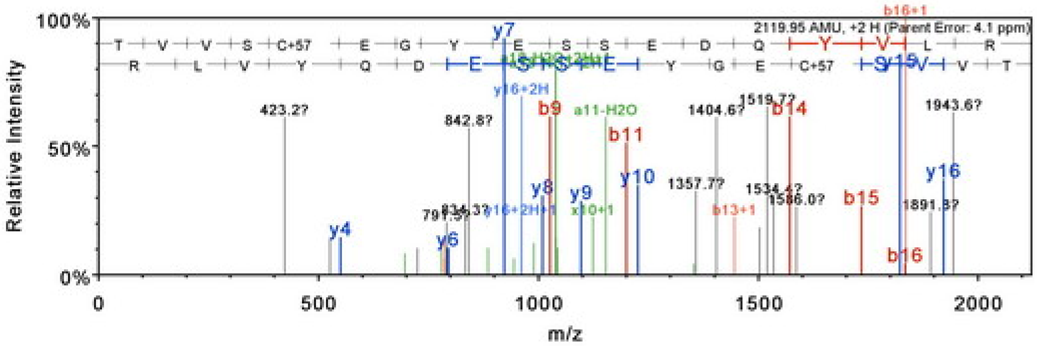

B	B Ions	B+2H	B-NH3	B-H2O	AA	Y Ions	Y+2H	Y-NH3	Y-H2O	Y
1	102.1			84.0	T	2120.9	1061.0	2103.9	2102.9	18
2	201.1			183.1	V	2019.9	1010.5	2002.9	2001.9	17
3	300.2			282.2	V	1920.8	960.9	1903.8	1902.8	16
4	387.2			369.2	S	1821.8	911.4	1804.7	1803.8	15
5	547.3			529.2	C+57	1734.7	867.9	1717.7	1716.7	14
6	676.3	338.7		658.3	E	1574.7	787.9	1557.7	1556.7	13
7	733.3	367.2		715.3	G	1445.7	723.3	1428.6	1427.6	12
8	896.4	448.7		878.4	Y	1388.6	694.8	1371.6	1370.6	11
9	1025.4	513.2		1007.4	E	1225.6	613.3	1208.5	1207.6	10
10	1112.5	556.7		1094.5	S	1096.5	548.8	1079.5	1078.5	9
11	1199.5	600.3		1181.5	S	1009.5	505.3	992.5	991.5	8
12	1328.5	664.8		1310.5	E	922.5	461.7	905.4	904.5	7
13	1443.6	722.3		1425.5	D	793.4	397.2	776.4	775.4	6
14	1571.6	786.3	1554.6	1553.6	Q	678.4		661.4		5
15	1734.7	867.8	1717.7	1716.7	Y	550.3		533.3		4
16	1833.8	917.4	1816.7	1815.7	V	387.3		370.3		3
17	1946.8	973.9	1929.8	1928.8	L	288.2		271.2		2
18	2120.9	1061.0	2103.9	2102.9	R	175.1		158.1		1

Gene symbol: Tom1l1

Protein name: Isoform 1 of TOM1-like protein 1

Protein accession numbers: IPI00124710,IPI00230596,IPI00649476

Peptide sequence: (K)MNVKVMTAILMENTPGSENHEDIELLR(K)

Exclusive (unique to this protein): TRUE

SEQUEST XCorr score: 2.59 SEQUEST DCn score: 0.348

Fix modifications: None

Variable modifications: M1: Oxidation (+16.00), M6: Oxidation (+16.00)

charge: 3 Observed m/z: 1039.5105

Actual minus calculated peptide mass (AMU): 0.0092755

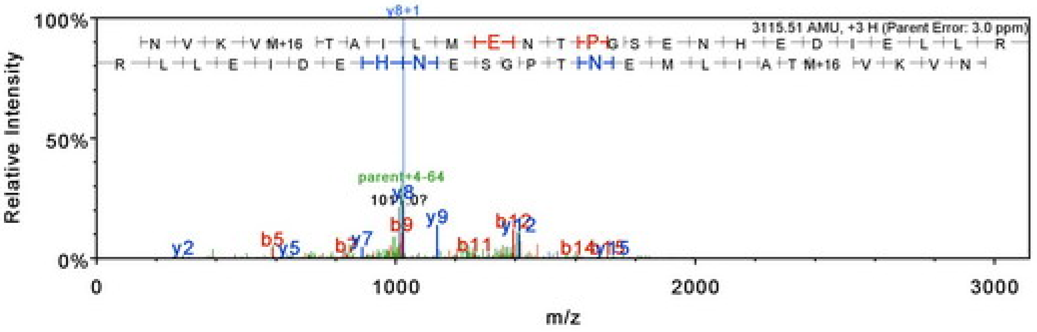

B	B Ions	B+2H	B-NH3	B-H2O	AA	Y Ions	Y+2H	Y-NH3	Y-H2O	Y
1	148.0				M+16	3116.5	1558.8	3099.5	3098.5	27
2	262.1		245.1		N	2969.5	1485.2	2952.4	2951.5	26
3	361.2		344.1		V	2855.4	1428.2	2838.4	2837.4	25
4	489.3	245.1	472.2		K	2756.4	1378.7	2739.3	2738.3	24
5	588.3	294.7	571.3		V	2628.3	1314.6	2611.2	2610.3	23
6	735.4	368.2	718.3		M+16	2529.2	1265.1	2512.2	2511.2	22
7	836.4	418.7	819.4	818.4	T	2382.2	1191.6	2365.1	2364.2	21
8	907.4	454.2	890.4	889.4	A	2281.1	1141.1	2264.1	2263.1	20
9	1020.5	510.8	1003.5	1002.5	I	2210.1	1105.5	2193.1	2192.1	19
10	1133.6	567.3	1116.6	1115.6	L	2097.0	1049.0	2080.0	2079.0	18
11	1264.7	632.8	1247.6	1246.6	M	1983.9	992.5	1966.9	1965.9	17
12	1393.7	697.4	1376.7	1375.7	E	1852.9	926.9	1835.8	1834.9	16
13	1507.7	754.4	1490.7	1489.7	N	1723.8	862.4	1706.8	1705.8	15
14	1608.8	804.9	1591.8	1590.8	T	1609.8	805.4	1592.8	1591.8	14
15	1705.8	853.4	1688.8	1687.8	P	1508.7	754.9	1491.7	1490.7	13
16	1762.9	881.9	1745.8	1744.8	G	1411.7	706.3	1394.7	1393.7	12
17	1849.9	925.5	1832.9	1831.9	S	1354.7	677.8	1337.6	1336.7	11
18	1978.9	990.0	1961.9	1960.9	E	1267.6	634.3	1250.6	1249.6	10
19	2093.0	1047.0	2075.9	2075.0	N	1138.6	569.8	1121.6	1120.6	9
20	2230.0	1115.5	2213.0	2212.0	H	1024.5	512.8	1007.5	1006.5	8
21	2359.1	1180.0	2342.1	2341.1	E	887.5	444.3	870.5	869.5	7
22	2474.1	1237.5	2457.1	2456.1	D	758.4	379.7	741.4	740.4	6
23	2587.2	1294.1	2570.2	2569.2	I	643.4		626.4	625.4	5
24	2716.2	1358.6	2699.2	2698.2	E	530.3		513.3	512.3	4
25	2829.3	1415.2	2812.3	2811.3	L	401.3		384.3		3
26	2942.4	1471.7	2925.4	2924.4	L	288.2		271.2		2
27	3116.5	1558.8	3099.5	3098.5	R	175.1		158.1		1

Gene symbol: Tpd52

Protein name: Tumor protein D52

Protein accession numbers: IPI00408626,IPI00473743,IPI00622606,IPI00622833,IPI00625441

Peptide sequence: (K)ASAAFSSVGSVITK(K)

Exclusive (unique to this protein): TRUE

SEQUEST XCorr score: 2.55 SEQUEST DCn score: 0.551

Fix modifications: None

Variable modifications: None

charge: 2 Observed m/z: 662.8588

Actual minus calculated peptide mass (AMU): -0.001465

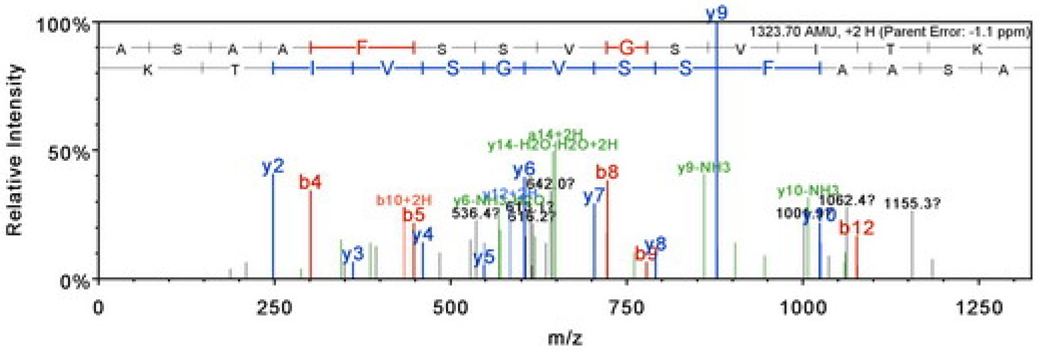

B	B Ions	B+2H	B-NH3	B-H2O	AA	Y Ions	Y+2H	Y-NH3	Y-H2O	Y
1	72.0				A	1324.7	662.9	1307.7	1306.7	14
2	159.1			141.1	S	1253.7	627.3	1236.7	1235.7	13
3	230.1			212.1	A	1166.6	583.8	1149.6	1148.6	12
4	301.2			283.1	A	1095.6	548.3	1078.6	1077.6	11
5	448.2			430.2	F	1024.6	512.8	1007.5	1006.6	10
6	535.3	268.1		517.2	S	877.5	439.3	860.5	859.5	9
7	622.3	311.7		604.3	S	790.5	395.7	773.4	772.5	8
8	721.4	361.2		703.3	V	703.4	352.2	686.4	685.4	7
9	778.4	389.7		760.4	G	604.4	302.7	587.3	586.4	6
10	865.4	433.2		847.4	S	547.4		530.3	529.3	5
11	964.5	482.7		946.5	V	460.3		443.3	442.3	4
12	1077.6	539.3		1059.5	I	361.3		344.2	343.2	3
13	1178.6	589.8		1160.6	T	248.2		231.1	230.2	2
14	1324.7	662.9	1307.7	1306.7	K	147.1		130.1		1

Gene symbol: Tpi1

Protein name: Triosephosphate isomerase

Protein accession numbers: IPI00467833

Peptide sequence: (K)DLGATWVVLGHSER(R)

Exclusive (unique to this protein): TRUE

SEQUEST XCorr score: 2.6 SEQUEST DCn score: 0.731

Fix modifications: None

Variable modifications: None

charge: 2 Observed m/z: 770.3953

Actual minus calculated peptide mass (AMU): -0.009399

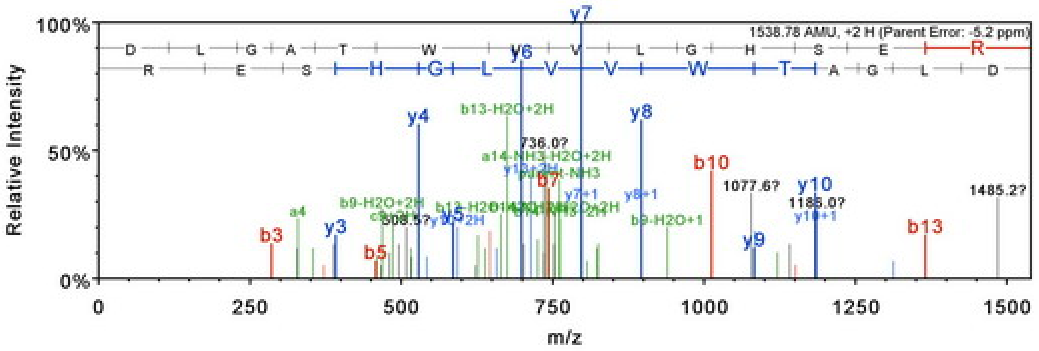

B	B Ions	B+2H	B-NH3	B-H2O	AA	Y Ions	Y+2H	Y-NH3	Y-H2O	Y
1	116.0			98.0	D	1539.8	770.4	1522.8	1521.8	14
2	229.1			211.1	L	1424.8	712.9	1407.7	1406.8	13
3	286.1			268.1	G	1311.7	656.3	1294.7	1293.7	12
4	357.2			339.2	A	1254.7	627.8	1237.6	1236.7	11
5	458.2			440.2	T	1183.6	592.3	1166.6	1165.6	10
6	644.3	322.7		626.3	W	1082.6	541.8	1065.5	1064.6	9
7	743.4	372.2		725.4	V	896.5	448.8	879.5	878.5	8
8	842.4	421.7		824.4	V	797.4	399.2	780.4	779.4	7
9	955.5	478.3		937.5	L	698.4	349.7	681.3	680.4	6
10	1012.6	506.8		994.5	G	585.3	293.1	568.3	567.3	5
11	1149.6	575.3		1131.6	H	528.3	264.6	511.2	510.2	4
12	1236.6	618.8		1218.6	S	391.2		374.2	373.2	3
13	1365.7	683.3		1347.7	E	304.2		287.1	286.2	2
14	1539.8	770.4	1522.8	1521.8	R	175.1		158.1		1

Gene symbol: Tsfm

Protein name: Elongation factor Ts, mitochondrial precursor

Protein accession numbers: IPI00113052

Peptide sequence: (K)GFLNSSELSELAAGPDR(E)

Exclusive (unique to this protein): TRUE

SEQUEST XCorr score: 2.4 SEQUEST DCn score: 0.577

Fix modifications: None

Variable modifications: None

charge: 2 Observed m/z: 881.9363

Actual minus calculated peptide mass (AMU): 0.0035358

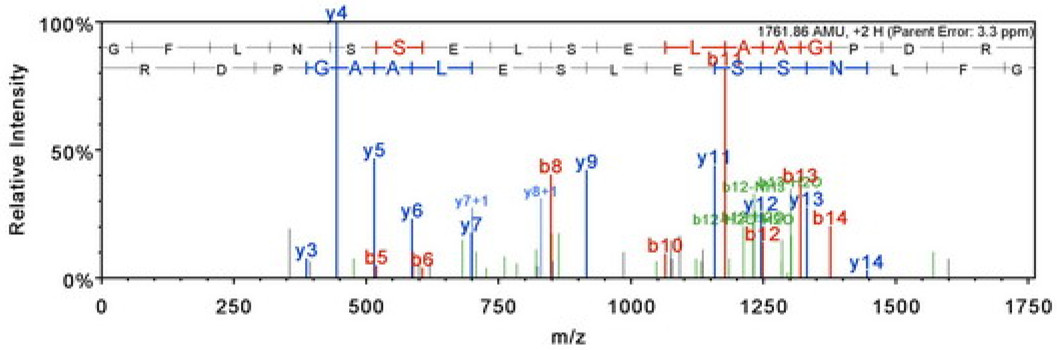

B	B Ions	B+2H	B-NH3	B-H2O	AA	Y Ions	Y+2H	Y-NH3	Y-H2O	Y
1	58.0				G	1762.9	881.9	1745.8	1744.9	17
2	205.1				F	1705.8	853.4	1688.8	1687.8	16
3	318.2				L	1558.8	779.9	1541.7	1540.8	15
4	432.2		415.2		N	1445.7	723.4	1428.7	1427.7	14
5	519.3		502.2	501.3	S	1331.6	666.3	1314.6	1313.6	13
6	606.3	303.7	589.3	588.3	S	1244.6	622.8	1227.6	1226.6	12
7	735.3	368.2	718.3	717.3	E	1157.6	579.3	1140.5	1139.6	11
8	848.4	424.7	831.4	830.4	L	1028.5	514.8	1011.5	1010.5	10
9	935.5	468.2	918.4	917.4	S	915.5	458.2	898.4	897.4	9
10	1064.5	532.8	1047.5	1046.5	E	828.4	414.7	811.4	810.4	8
11	1177.6	589.3	1160.5	1159.6	L	699.4	350.2	682.4	681.4	7
12	1248.6	624.8	1231.6	1230.6	A	586.3	293.7	569.3	568.3	6
13	1319.7	660.3	1302.6	1301.6	A	515.3		498.2	497.3	5
14	1376.7	688.8	1359.6	1358.7	G	444.2		427.2	426.2	4
15	1473.7	737.4	1456.7	1455.7	P	387.2		370.2	369.2	3
16	1588.8	794.9	1571.7	1570.7	D	290.2		273.1	272.1	2
17	1762.9	881.9	1745.8	1744.9	R	175.1		158.1		1

Gene symbol: Tufm

Protein name: Isoform 1 of Elongation factor Tu, mitochondrial precursor

Protein accession numbers: IPI00274407,IPI00460132,IPI00625588

Peptide sequence: (K)LLDAVDTYIPVPTR(D)

Exclusive (unique to this protein): TRUE

SEQUEST XCorr score: 2.23 SEQUEST DCn score: 0.45

Fix modifications: None

Variable modifications: None

charge: 2 Observed m/z: 786.9321

Actual minus calculated peptide mass (AMU): -0.00769

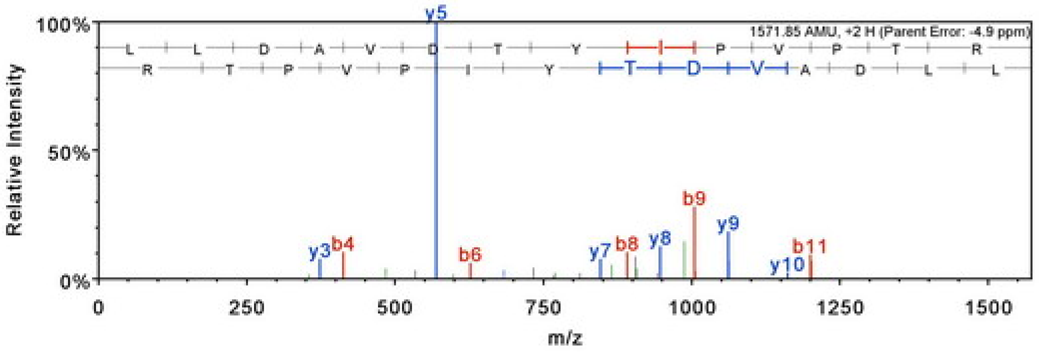

B	B Ions	B+2H	B-NH3	B-H2O	AA	Y Ions	Y+2H	Y-NH3	Y-H2O	Y
1	114.1				L	1572.9	786.9	1555.8	1554.9	14
2	227.2				L	1459.8	730.4	1442.8	1441.8	13
3	342.2			324.2	D	1346.7	673.9	1329.7	1328.7	12
4	413.2			395.2	A	1231.7	616.3	1214.6	1213.7	11
5	512.3			494.3	V	1160.6	580.8	1143.6	1142.6	10
6	627.3	314.2		609.3	D	1061.6	531.3	1044.5	1043.5	9
7	728.4	364.7		710.4	T	946.5	473.8	929.5	928.5	8
8	891.5	446.2		873.4	Y	845.5	423.3	828.5	827.5	7
9	1004.5	502.8		986.5	I	682.4	341.7	665.4	664.4	6
10	1101.6	551.3		1083.6	P	569.3		552.3	551.3	5
11	1200.7	600.8		1182.6	V	472.3		455.3	454.3	4
12	1297.7	649.4		1279.7	P	373.2		356.2	355.2	3
13	1398.8	699.9		1380.7	T	276.2		259.1	258.2	2
14	1572.9	786.9	1555.8	1554.9	R	175.1		158.1		1

Gene symbol: Txndc5

Protein name: Thioredoxin domain-containing protein 5 precursor

Protein accession numbers: IPI00163011

Peptide sequence: (R)DLDSLHSFVLR(Q)

Exclusive (unique to this protein): TRUE

SEQUEST XCorr score: 2.02 SEQUEST DCn score: 0.608

Fix modifications: None

Variable modifications: None

charge: 2 Observed m/z: 651.3428

Actual minus calculated peptide mass (AMU): -0.00769

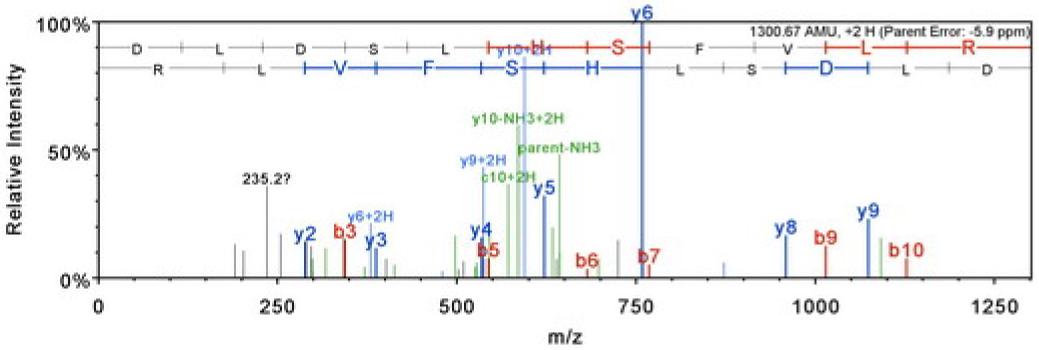

B	B Ions	B+2H	B-NH3	B-H2O	AA	Y Ions	Y+2H	Y-NH3	Y-H2O	Y
1	116.0			98.0	D	1301.7	651.4	1284.7	1283.7	11
2	229.1			211.1	L	1186.7	593.8	1169.6	1168.7	10
3	344.2			326.1	D	1073.6	537.3	1056.5	1055.6	9
4	431.2			413.2	S	958.6	479.8	941.5	940.5	8
5	544.3			526.3	L	871.5	436.3	854.5	853.5	7
6	681.3	341.2		663.3	H	758.4	379.7	741.4	740.4	6
7	768.4	384.7		750.3	S	621.4		604.4	603.4	5
8	915.4	458.2		897.4	F	534.3		517.3		4
9	1014.5	507.8		996.5	V	387.3		370.3		3
10	1127.6	564.3		1109.6	L	288.2		271.2		2
11	1301.7	651.4	1284.7	1283.7	R	175.1		158.1		1

Gene symbol: Txnl2
Protein name: Thioredoxin-like protein 2
Protein accession numbers: IPI00315550
Peptide sequence: (R)GDLVGGLDIVK(E)
Exclusive (unique to this protein): TRUE
SEQUEST XCorr score: 2.18 SEQUEST DCn score: 0.307
Fix modifications: None
Variable modifications: None
charge: 2 Observed m/z: 543.3113
Actual minus calculated peptide mass (AMU): -0.006226

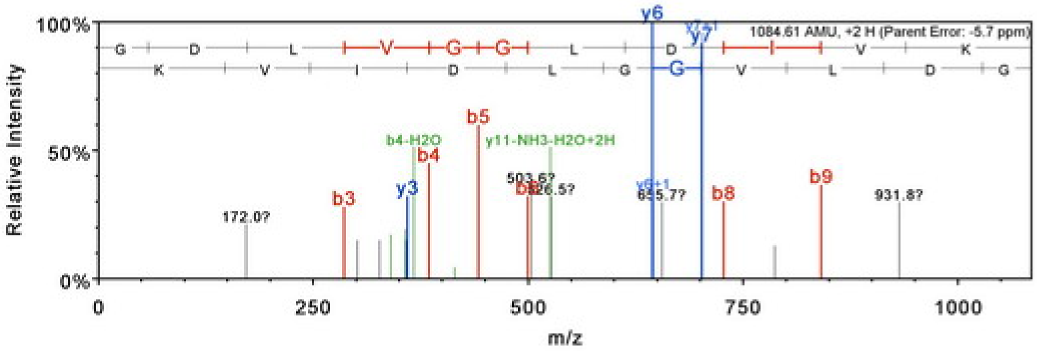

B	B Ions	B+2H	B-NH3	B-H2O	AA	Y Ions	Y+2H	Y-NH3	Y-H2O	Y
1	58.0				G	1085.6	543.3	1068.6	1067.6	11
2	173.1			155.1	D	1028.6	514.8	1011.6	1010.6	10
3	286.1			268.1	L	913.6	457.3	896.6	895.6	9
4	385.2			367.2	V	800.5	400.8	783.5	782.5	8
5	442.2			424.2	G	701.4	351.2	684.4	683.4	7
6	499.3	250.1		481.2	G	644.4	322.7	627.4	626.4	6
7	612.3	306.7		594.3	L	587.4		570.4	569.4	5
8	727.4	364.2		709.4	D	474.3		457.3	456.3	4
9	840.5	420.7		822.4	I	359.3		342.2		3
10	939.5	470.3		921.5	V	246.2		229.2		2
11	1085.6	543.3	1068.6	1067.6	K	147.1		130.1		1

Gene symbol: Ube1l2
Protein name: Ubiquitin-activating enzyme E1-like protein 2
Protein accession numbers: IPI00226815
Peptide sequence: (K)ALQLLHCFPLETR(L)
Exclusive (unique to this protein): TRUE
SEQUEST XCorr score: 2.3 SEQUEST DCn score: 0.652
Fix modifications: C7: Carbamidomethyl (+57.02)Variable modifications: None
charge: 2 Observed m/z: 799.4271
Actual minus calculated peptide mass (AMU): -0.0063456

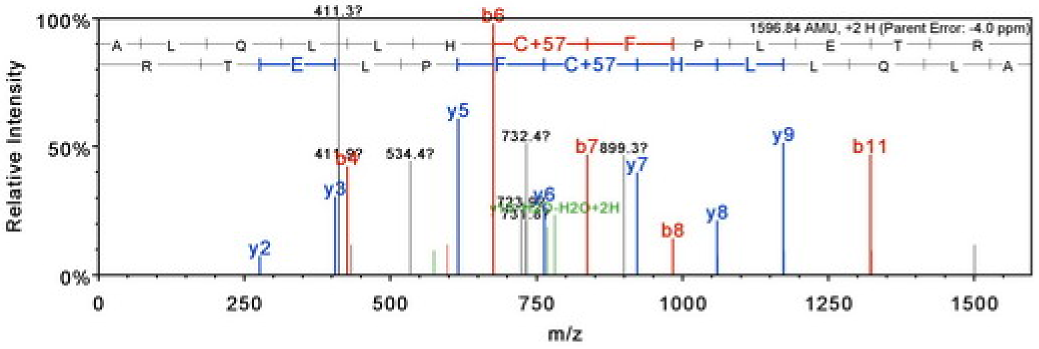

B	B Ions	B+2H	B-NH3	B-H2O	AA	Y Ions	Y+2H	Y-NH3	Y-H2O	Y
1	72.0				A	1597.9	799.4	1580.8	1579.8	13
2	185.1				L	1526.8	763.9	1509.8	1508.8	12
3	313.2		296.2		Q	1413.7	707.4	1396.7	1395.7	11
4	426.3		409.3		L	1285.7	643.3	1268.7	1267.7	10
5	539.4		522.3		L	1172.6	586.8	1155.6	1154.6	9
6	676.4	338.7	659.4		H	1059.5	530.3	1042.5	1041.5	8
7	836.5	418.7	819.4		C+57	922.5	461.7	905.4	904.4	7
8	983.5	492.3	966.5		F	762.4	381.7	745.4	744.4	6
9	1080.6	540.8	1063.5		P	615.4		598.3	597.3	5
10	1193.7	597.3	1176.6		L	518.3		501.3	500.3	4
11	1322.7	661.9	1305.7	1304.7	E	405.2		388.2	387.2	3
12	1423.7	712.4	1406.7	1405.7	T	276.2		259.1	258.2	2
13	1597.9	799.4	1580.8	1579.8	R	175.1		158.1		1

Gene symbol: Ube2g1

Protein name: Ubiquitin-conjugating enzyme E2 G1

Protein accession numbers: IPI00310850

Peptide sequence: (K)NPVEGFSAGLIDDNDLYR(W)

Exclusive (unique to this protein): TRUE

SEQUEST XCorr score: 2.3 SEQUEST DCn score: 0.581

Fix modifications: None

Variable modifications: None

charge: 2 Observed m/z: 997.973

Actual minus calculated peptide mass (AMU): -0.007935

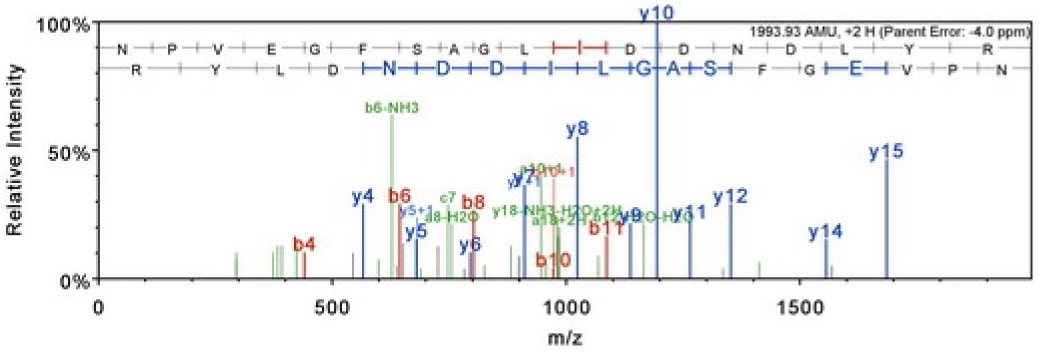

B	B Ions	B+2H	B-NH3	B-H2O	AA	Y Ions	Y+2H	Y-NH3	Y-H2O	Y
1	115.1		98.0		N	1995.0	998.0	1977.9	1976.9	18
2	212.1		195.1		P	1880.9	941.0	1863.9	1862.9	17
3	311.2		294.2		V	1783.9	892.4	1766.8	1765.8	16
4	440.2		423.2	422.2	E	1684.8	842.9	1667.8	1666.8	15
5	497.2		480.2	479.2	G	1555.7	778.4	1538.7	1537.7	14
6	644.3	322.7	627.3	626.3	F	1498.7	749.9	1481.7	1480.7	13
7	731.3	366.2	714.3	713.3	S	1351.7	676.3	1334.6	1333.6	12
8	802.4	401.7	785.4	784.4	A	1264.6	632.8	1247.6	1246.6	11
9	859.4	430.2	842.4	841.4	G	1193.6	597.3	1176.5	1175.6	10
10	972.5	486.7	955.5	954.5	L	1136.6	568.8	1119.5	1118.5	9
11	1085.6	543.3	1068.5	1067.5	I	1023.5	512.2	1006.5	1005.5	8
12	1200.6	600.8	1183.6	1182.6	D	910.4	455.7	893.4	892.4	7
13	1315.6	658.3	1298.6	1297.6	D	795.4	398.2	778.3	777.4	6
14	1429.7	715.3	1412.6	1411.7	N	680.3		663.3	662.3	5
15	1544.7	772.9	1527.7	1526.7	D	566.3		549.3	548.3	4
16	1657.8	829.4	1640.7	1639.8	L	451.3		434.2		3
17	1820.8	910.9	1803.8	1802.8	Y	338.2		321.2		2
18	1995.0	998.0	1977.9	1976.9	R	175.1		158.1		1

Gene symbol: Ube2n

Protein name: Ubiquitin-conjugating enzyme E2 N

Protein accession numbers: IPI00165854,IPI00850217

Peptide sequence: (R)LLAEPVPGIK(A)

Exclusive (unique to this protein): TRUE

SEQUEST XCorr score: 2.08 SEQUEST DCn score: 0.944

Fix modifications: None

Variable modifications: None

charge: 2 Observed m/z: 518.824

Actual minus calculated peptide mass (AMU): -0.0008545

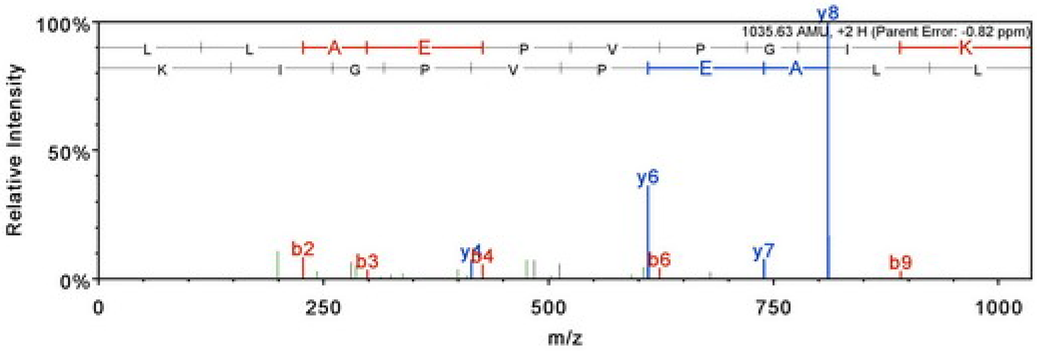

B	B Ions	B+2H	B-NH3	B-H2O	AA	Y Ions	Y+2H	Y-NH3	Y-H2O	Y
1	114.1				L	1036.6	518.8	1019.6	1018.6	10
2	227.2				L	923.6	462.3	906.5	905.6	9
3	298.2				A	810.5	405.7	793.5	792.5	8
4	427.3			409.3	E	739.4	370.2	722.4	721.4	7
5	524.3			506.3	P	610.4	305.7	593.4		6
6	623.4	312.2		605.4	V	513.3		496.3		5
7	720.4	360.7		702.4	P	414.3		397.3		4
8	777.5	389.2		759.4	G	317.2		300.2		3
9	890.5	445.8		872.5	I	260.2		243.2		2
10	1036.6	518.8	1019.6	1018.6	K	147.1		130.1		1

Gene symbol: Ube2s

Protein name: Ubiquitin-conjugating enzyme E2 S

Protein accession numbers: IPI00121891

Peptide sequence: (R)DWTAELGIR(H)

Exclusive (unique to this protein): TRUE

SEQUEST XCorr score: 2.08 SEQUEST DCn score: 0.405

Fix modifications: None

Variable modifications: None

charge: 2 Observed m/z: 530.7747

Actual minus calculated peptide mass (AMU): -0.001465

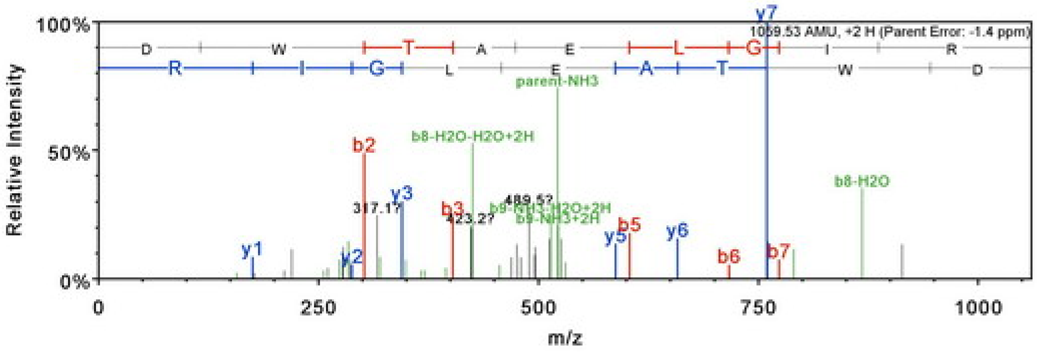

B	B Ions	B+2H	B-NH3	B-H2O	AA	Y Ions	Y+2H	Y-NH3	Y-H2O	Y
1	116.0			98.0	D	1060.5	530.8	1043.5	1042.5	9
2	302.1			284.1	W	945.5	473.3	928.5	927.5	8
3	403.2			385.2	T	759.4	380.2	742.4	741.4	7
4	474.2			456.2	A	658.4	329.7	641.4	640.4	6
5	603.2			585.2	E	587.4		570.3	569.3	5
6	716.3	358.7		698.3	L	458.3		441.3		4
7	773.4	387.2		755.3	G	345.2		328.2		3
8	886.4	443.7		868.4	I	288.2		271.2		2
9	1060.5	530.8	1043.5	1042.5	R	175.1		158.1		1

Gene symbol: Ubxd2

Protein name: UBX domain-containing protein 2

Protein accession numbers: IPI00165799,IPI00719854

Peptide sequence: (R)LLDLELAPSASVVLLPAGR(P)
Exclusive (unique to this protein): TRUE
SEQUEST XCorr score: 2.05 SEQUEST DCn score: 0.672
Fix modifications: None
Variable modifications: None
charge: 2 Observed m/z: 967.5669
Actual minus calculated peptide mass (AMU): -0.006958

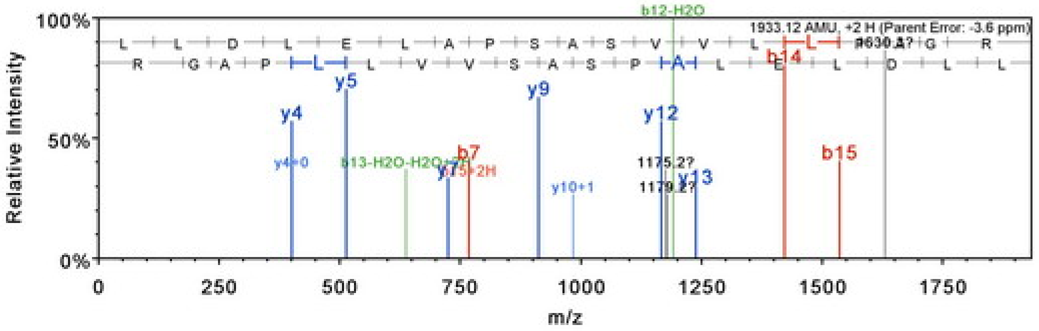

B	B Ions	B+2H	B-NH3	B-H2O	AA	Y Ions	Y+2H	Y-NH3	Y-H2O	Y
1	114.1				L	1934.1	967.6	1917.1	1916.1	19
2	227.2				L	1821.1	911.0	1804.0	1803.0	18
3	342.2			324.2	D	1708.0	854.5	1690.9	1690.0	17
4	455.3			437.3	L	1592.9	797.0	1575.9	1574.9	16
5	584.3			566.3	E	1479.9	740.4	1462.8	1461.8	15
6	697.4	349.2		679.4	L	1350.8	675.9	1333.8	1332.8	14
7	768.5	384.7		750.4	A	1237.7	619.4	1220.7	1219.7	13
8	865.5	433.3		847.5	P	1166.7	583.9	1149.7	1148.7	12
9	952.5	476.8		934.5	S	1069.6	535.3	1052.6	1051.6	11
10	1023.6	512.3		1005.6	A	982.6	491.8	965.6	964.6	10
11	1110.6	555.8		1092.6	S	911.6	456.3	894.5	893.6	9
12	1209.7	605.3		1191.7	V	824.5	412.8	807.5		8
13	1308.7	654.9		1290.7	V	725.5	363.2	708.4		7
14	1421.8	711.4		1403.8	L	626.4	313.7	609.4		6
15	1534.9	768.0		1516.9	L	513.3		496.3		5
16	1632.0	816.5		1614.0	P	400.2		383.2		4
17	1703.0	852.0		1685.0	A	303.2		286.2		3
18	1760.0	880.5		1742.0	G	232.1		215.1		2
19	1934.1	967.6	1917.1	1916.1	R	175.1		158.1		1

Gene symbol: Upp1
Protein name: Uridine phosphorylase 1
Protein accession numbers: IPI00323353
Peptide sequence: (K)AAVVCVTLLDR(L)
Exclusive (unique to this protein): TRUE
SEQUEST XCorr score: 2.42 SEQUEST DCn score: 0.584
Fix modifications: C5: Carbamidomethyl (+57.02)Variable modifications: None
charge: 2 Observed m/z: 608.8397
Actual minus calculated peptide mass (AMU): -0.0009766

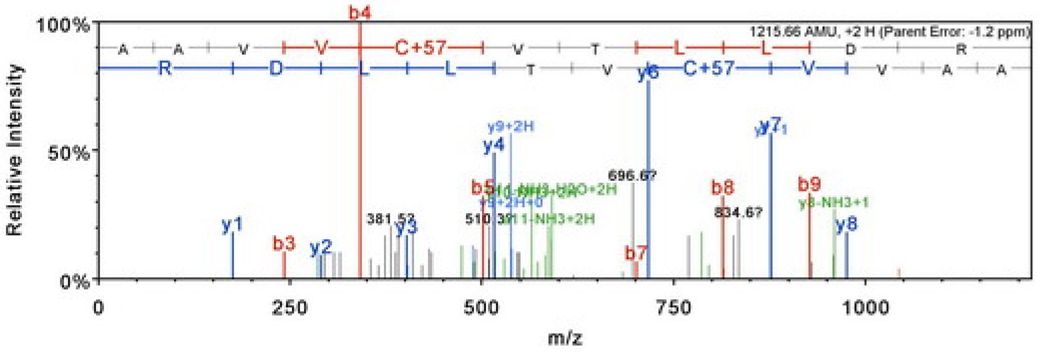

B	B Ions	B+2H	B-NH3	B-H2O	AA	Y Ions	Y+2H	Y-NH3	Y-H2O	Y
1	72.0				A	1216.7	608.8	1199.7	1198.7	11
2	143.1				A	1145.6	573.3	1128.6	1127.6	10
3	242.2				V	1074.6	537.8	1057.6	1056.6	9
4	341.2				V	975.5	488.3	958.5	957.5	8
5	501.3				C+57	876.5	438.7	859.4	858.5	7
6	600.3	300.7			V	716.4	358.7	699.4	698.4	6
7	701.4	351.2		683.4	T	617.4		600.3	599.4	5
8	814.5	407.7		796.4	L	516.3		499.3	498.3	4
9	927.5	464.3		909.5	L	403.2		386.2	385.2	3
10	1042.6	521.8		1024.5	D	290.2		273.1	272.1	2
11	1216.7	608.8	1199.7	1198.7	R	175.1		158.1		1

Gene symbol: Uqcrc1

Protein name: Ubiquinol-cytochrome-c reductase complex core protein 1, mitochondrial precursor

Protein accession numbers: IPI00111885,IPI00653598

Peptide sequence: (R)NALVSHLDGTTPVCEDIGR(S)

Exclusive (unique to this protein): TRUE

SEQUEST XCorr score: 2.56 SEQUEST DCn score: 0.509

Fix modifications: C14: Carbamidomethyl (+57.02)Variable modifications: None

charge: 3 Observed m/z: 685.3353

Actual minus calculated peptide mass (AMU): -0.007568

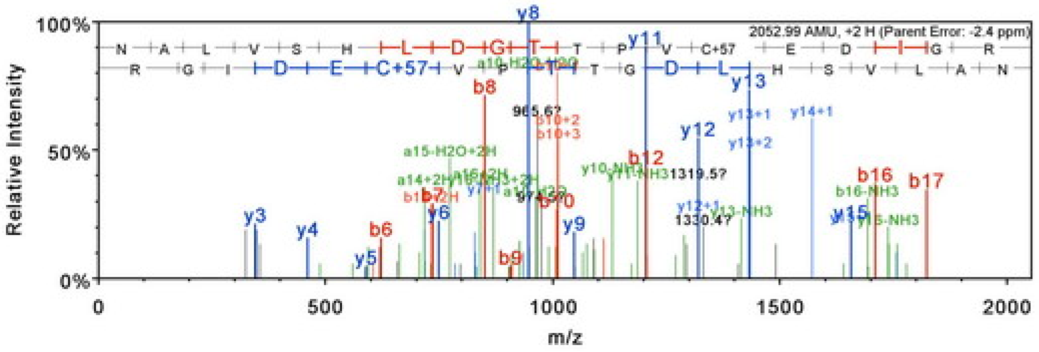

B	B Ions	B+2H	B-NH3	B-H2O	AA	Y Ions	Y+2H	Y-NH3	Y-H2O	Y
1	115.1		98.0		N	2054.0	1027.5	2037.0	2036.0	19
2	186.1		169.1		A	1940.0	970.5	1922.9	1921.9	18
3	299.2		282.2		L	1868.9	935.0	1851.9	1850.9	17
4	398.2		381.2		V	1755.8	878.4	1738.8	1737.8	16
5	485.3		468.3	467.3	S	1656.8	828.9	1639.7	1638.8	15
6	622.3	311.7	605.3	604.3	H	1569.7	785.4	1552.7	1551.7	14
7	735.4	368.2	718.4	717.4	L	1432.7	716.8	1415.7	1414.7	13
8	850.4	425.7	833.4	832.4	D	1319.6	660.3	1302.6	1301.6	12
9	907.5	454.2	890.4	889.5	G	1204.6	602.8	1187.5	1186.5	11
10	1008.5	504.8	991.5	990.5	T	1147.5	574.3	1130.5	1129.5	10
11	1109.6	555.3	1092.5	1091.5	T	1046.5	523.8	1029.5	1028.5	9
12	1206.6	603.8	1189.6	1188.6	P	945.5	473.2	928.4	927.4	8
13	1305.7	653.3	1288.7	1287.7	V	848.4	424.7	831.4	830.4	7
14	1465.7	733.4	1448.7	1447.7	C+57	749.3	375.2	732.3	731.3	6
15	1594.8	797.9	1577.7	1576.7	E	589.3		572.3	571.3	5
16	1709.8	855.4	1692.8	1691.8	D	460.3		443.2	442.2	4
17	1822.9	911.9	1805.8	1804.9	I	345.2		328.2		3
18	1879.9	940.5	1862.9	1861.9	G	232.1		215.1		2
19	2054.0	1027.5	2037.0	2036.0	R	175.1		158.1		1

Gene symbol: Uqcrq

Protein name: Ubiquinol-cytochrome c reductase complex ubiquinone-binding protein QP-C

Protein accession numbers: IPI00224210

Peptide sequence: (R)HVISYSLSPFEQR(A)

Exclusive (unique to this protein): TRUE

SEQUEST XCorr score: 2.66 SEQUEST DCn score: 0.553

Fix modifications: None

Variable modifications: None

charge: 2 Observed m/z: 781.8987

Actual minus calculated peptide mass (AMU): -0.0070758

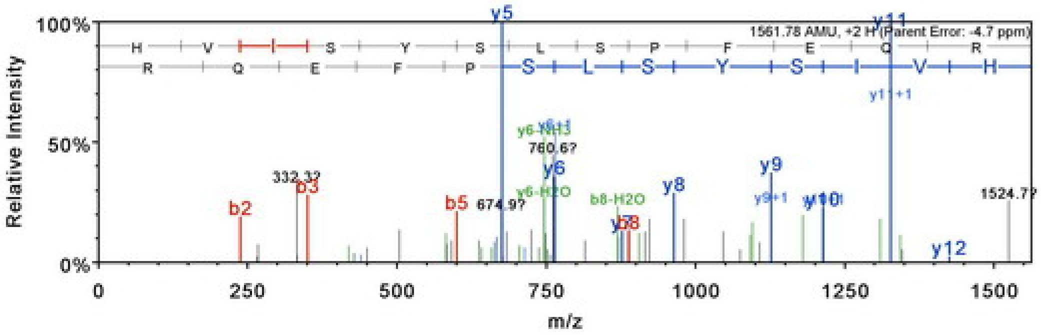

B	B Ions	B+2H	B-NH3	B-H2O	AA	Y Ions	Y+2H	Y-NH3	Y-H2O	Y
1	138.1	69.5			H	1562.8	781.9	1545.8	1544.8	13
2	237.1	119.1			V	1425.7	713.4	1408.7	1407.7	12
3	350.2	175.6			I	1326.7	663.8	1309.6	1308.7	11
4	437.3	219.1		419.2	S	1213.6	607.3	1196.6	1195.6	10
5	600.3	300.7		582.3	Y	1126.5	563.8	1109.5	1108.5	9
6	687.4	344.2		669.3	S	963.5	482.3	946.5	945.5	8
7	800.4	400.7		782.4	L	876.5	438.7	859.4	858.5	7
8	887.5	444.2		869.5	S	763.4	382.2	746.4	745.4	6
9	984.5	492.8		966.5	P	676.3		659.3	658.3	5
10	1131.6	566.3		1113.6	F	579.3		562.3	561.3	4
11	1260.6	630.8		1242.6	E	432.2		415.2	414.2	3
12	1388.7	694.9	1371.7	1370.7	Q	303.2		286.2		2
13	1562.8	781.9	1545.8	1544.8	R	175.1		158.1		1

Gene symbol: Usp15

Protein name: Isoform 1 of Ubiquitin carboxyl-terminal hydrolase 15

Protein accession numbers: IPI00154012,IPI00403342

Peptide sequence: (R)QDTFSGTGFFPLDR(E)

Exclusive (unique to this protein): TRUE

SEQUEST XCorr score: 2.19 SEQUEST DCn score: 0.609

Fix modifications: None

Variable modifications: None

charge: 2 Observed m/z: 794.3739

Actual minus calculated peptide mass (AMU): -0.004639

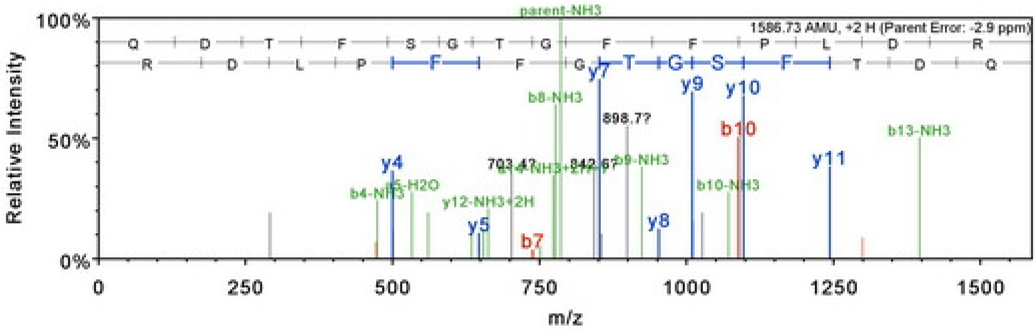

B	B Ions	B+2H	B-NH3	B-H2O	AA	Y Ions	Y+2H	Y-NH3	Y-H2O	Y
1	129.1		112.0		Q	1587.7	794.4	1570.7	1569.7	14
2	244.1		227.1	226.1	D	1459.7	730.4	1442.7	1441.7	13
3	345.1		328.1	327.1	T	1344.7	672.8	1327.6	1326.7	12
4	492.2		475.2	474.2	F	1243.6	622.3	1226.6	1225.6	11
5	579.2		562.2	561.2	S	1096.5	548.8	1079.5	1078.5	10
6	636.3	318.6	619.2	618.3	G	1009.5	505.3	992.5	991.5	9
7	737.3	369.2	720.3	719.3	T	952.5	476.8	935.5	934.5	8
8	794.3	397.7	777.3	776.3	G	851.4	426.2	834.4	833.4	7
9	941.4	471.2	924.4	923.4	F	794.4	397.7	777.4	776.4	6
10	1088.5	544.7	1071.4	1070.5	F	647.4		630.3	629.3	5
11	1185.5	593.3	1168.5	1167.5	P	500.3		483.3	482.3	4
12	1298.6	649.8	1281.6	1280.6	L	403.2		386.2	385.2	3
13	1413.6	707.3	1396.6	1395.6	D	290.2		273.1	272.1	2
14	1587.7	794.4	1570.7	1569.7	R	175.1		158.1		1

Gene symbol: Usp19

Protein name: 16 days embryo kidney cDNA, RIKEN full-length enriched library, clone:I920015N15 product:ubiquitin specific protease 19, full insert sequence

Protein accession numbers: IPI00420483,IPI00457619,IPI00762408

Peptide sequence: (R)LEDVDAAFTDTDCVVR(L)

Exclusive (unique to this protein): TRUE

SEQUEST XCorr score: 2.03 SEQUEST DCn score: 0.709

Fix modifications: C13: Carbamidomethyl (+57.02)Variable modifications: None

charge: 2 Observed m/z: 913.4135

Actual minus calculated peptide mass (AMU): -0.008789

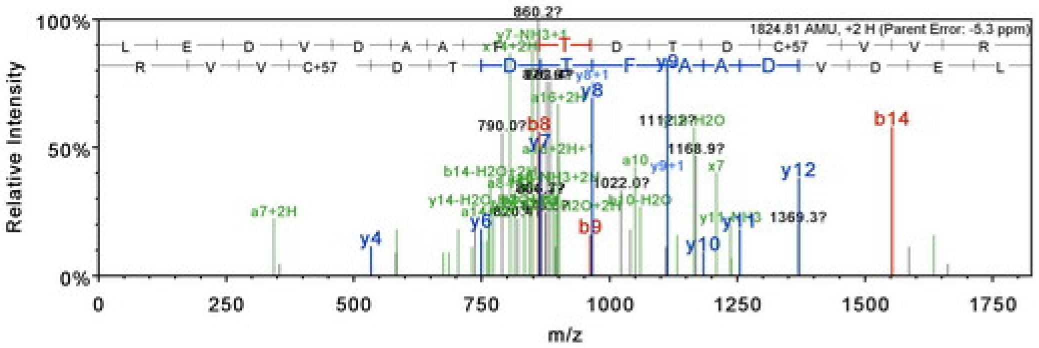

B	B Ions	B+2H	B-NH3	B-H2O	AA	Y Ions	Y+2H	Y-NH3	Y-H2O	Y
1	114.1				L	1825.8	913.4	1808.8	1807.8	16
2	243.1			225.1	E	1712.7	856.9	1695.7	1694.7	15
3	358.2			340.2	D	1583.7	792.4	1566.7	1565.7	14
4	457.2			439.2	V	1468.7	734.8	1451.7	1450.7	13
5	572.3			554.3	D	1369.6	685.3	1352.6	1351.6	12
6	643.3	322.2		625.3	A	1254.6	627.8	1237.5	1236.6	11
7	714.3	357.7		696.3	A	1183.5	592.3	1166.5	1165.5	10
8	861.4	431.2		843.4	F	1112.5	556.8	1095.5	1094.5	9
9	962.5	481.7		944.4	T	965.4	483.2	948.4	947.4	8
10	1077.5	539.2		1059.5	D	864.4	432.7	847.4	846.4	7
11	1178.5	589.8		1160.5	T	749.4	375.2	732.3	731.4	6
12	1293.5	647.3		1275.5	D	648.3		631.3	630.3	5
13	1453.6	727.3		1435.6	C+57	533.3		516.3		4
14	1552.7	776.8		1534.6	V	373.3		356.2		3
15	1651.7	826.4		1633.7	V	274.2		257.2		2
16	1825.8	913.4	1808.8	1807.8	R	175.1		158.1		1

Gene symbol: Usp9x

Protein name: Ubiquitin specific peptidase 9, X chromosome

Protein accession numbers: IPI00798468

Peptide sequence: (R)FSEYLLECPSAEVR(G)

Exclusive (unique to this protein): TRUE

SEQUEST XCorr score: 2.12 SEQUEST DCn score: 0.548

Fix modifications: C8: Carbamidomethyl (+57.02)Variable modifications: None

charge: 2 Observed m/z: 850.4008

Actual minus calculated peptide mass (AMU): -0.0063456

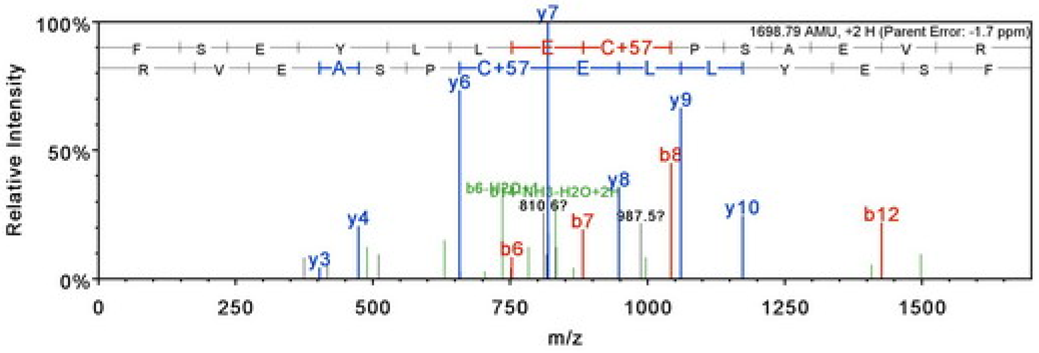

B	B Ions	B+2H	B-NH3	B-H2O	AA	Y Ions	Y+2H	Y-NH3	Y-H2O	Y
1	148.1				F	1699.8	850.4	1682.8	1681.8	14
2	235.1			217.1	S	1552.7	776.9	1535.7	1534.7	13
3	364.2			346.1	E	1465.7	733.4	1448.7	1447.7	12
4	527.2			509.2	Y	1336.7	668.8	1319.6	1318.7	11
5	640.3			622.3	L	1173.6	587.3	1156.6	1155.6	10
6	753.4	377.2		735.4	L	1060.5	530.8	1043.5	1042.5	9
7	882.4	441.7		864.4	E	947.4	474.2	930.4	929.4	8
8	1042.5	521.7		1024.4	C+57	818.4	409.7	801.4	800.4	7
9	1139.5	570.3		1121.5	P	658.4	329.7	641.3	640.3	6
10	1226.5	613.8		1208.5	S	561.3		544.3	543.3	5
11	1297.6	649.3		1279.6	A	474.3		457.2	456.3	4
12	1426.6	713.8		1408.6	E	403.2		386.2	385.2	3
13	1525.7	763.4		1507.7	V	274.2		257.2		2
14	1699.8	850.4	1682.8	1681.8	R	175.1		158.1		1

Gene symbol: Utp14b;Acsl3

Protein name: UTP14, U3 small nucleolar ribonucleoprotein, homolog B

Protein accession numbers: IPI00345617

Peptide sequence: (K)LLEAVSSLGR(K)

Exclusive (unique to this protein): TRUE

SEQUEST XCorr score: 2.04 SEQUEST DCn score: 0.37

Fix modifications: None

Variable modifications: None

charge: 2 Observed m/z: 522.8011

Actual minus calculated peptide mass (AMU): -0.011149

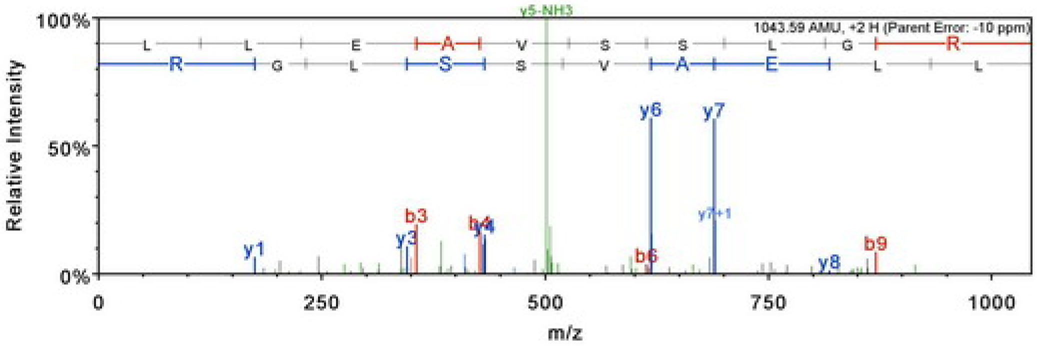

B	B Ions	B+2H	B-NH3	B-H2O	AA	Y Ions	Y+2H	Y-NH3	Y-H2O	Y
1	114.1				L	1044.6	522.8	1027.6	1026.6	10
2	227.2				L	931.5	466.3	914.5	913.5	9
3	356.2			338.2	E	818.4	409.7	801.4	800.4	8
4	427.3			409.3	A	689.4	345.2	672.4	671.4	7
5	526.3			508.3	V	618.4	309.7	601.3	600.4	6
6	613.4	307.2		595.4	S	519.3		502.3	501.3	5
7	700.4	350.7		682.4	S	432.3		415.2	414.3	4
8	813.5	407.2		795.5	L	345.2		328.2		3
9	870.5	435.8		852.5	G	232.1		215.1		2
10	1044.6	522.8	1027.6	1026.6	R	175.1		158.1		1

Gene symbol: Vat1

Protein name: Synaptic vesicle membrane protein VAT-1 homolog

Protein accession numbers: IPI00126072

Peptide sequence: (R)PAVPPAPGPGQLTLR(V)

Exclusive (unique to this protein): TRUE

SEQUEST XCorr score: 2.05 SEQUEST DCn score: 0.688

Fix modifications: None

Variable modifications: None

charge: 2 Observed m/z: 735.9225

Actual minus calculated peptide mass (AMU): -0.006226

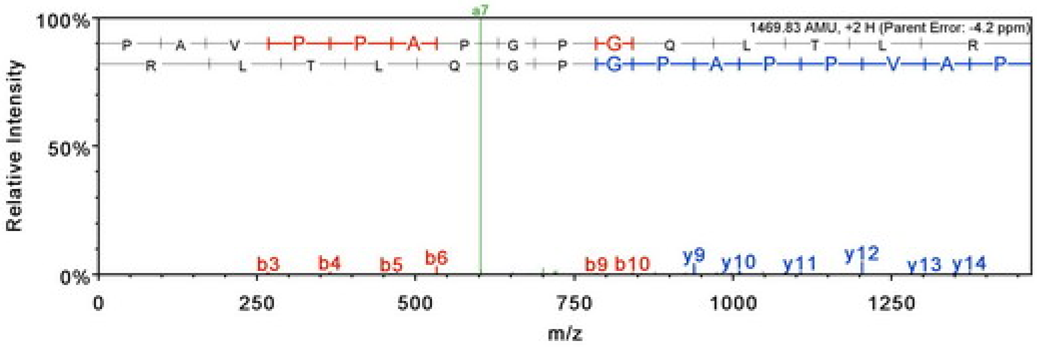

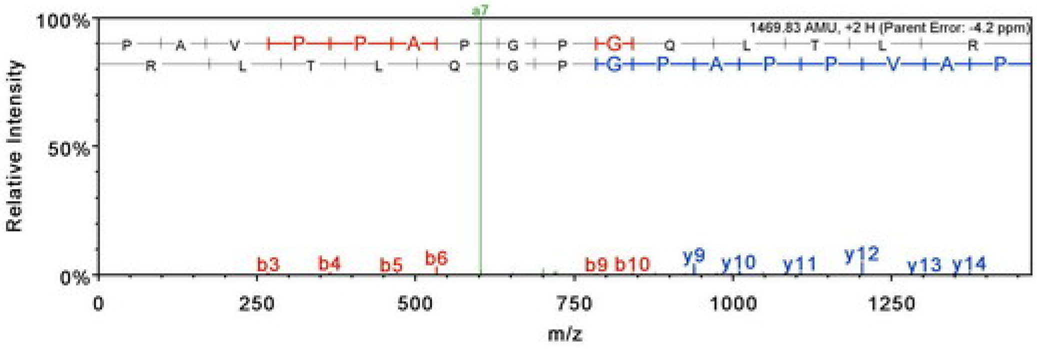

Gene symbol: Vdac1

Protein name: Isoform Pl-VDAC1 of Voltage-dependent anion-selective channel protein 1

Protein accession numbers: IPI00122549,IPI00230540

Peptide sequence: (R)WTEYGLTFTEK(W)

Exclusive (unique to this protein): TRUE

SEQUEST XCorr score: 2.35 SEQUEST DCn score: 0.544

Fix modifications: None

Variable modifications: None

charge: 2 Observed m/z: 687.8332

Actual minus calculated peptide mass (AMU): 0.0003662

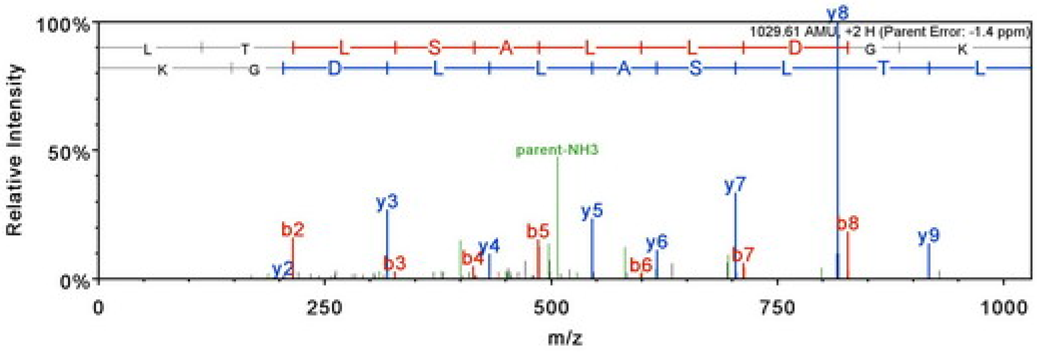

B	B Ions	B+2H	B-NH3	B-H2O	AA	Y Ions	Y+2H	Y-NH3	Y-H2O	Y
1	114.1				L	1030.6	515.8	1013.6	1012.6	10
2	215.1			197.1	T	917.5	459.3	900.5	899.5	9
3	328.2			310.2	L	816.5	408.8	799.5	798.5	8
4	415.3			397.3	S	703.4	352.2	686.4	685.4	7
5	486.3			468.3	A	616.4	308.7	599.3	598.4	6
6	599.4	300.2		581.4	L	545.3		528.3	527.3	5
7	712.5	356.7		694.5	L	432.3		415.2	414.2	4
8	827.5	414.3		809.5	D	319.2		302.1	301.2	3
9	884.5	442.8		866.5	G	204.1		187.1		2
10	1030.6	515.8	1013.6	1012.6	K	147.1		130.1		1

Gene symbol: Vkorc1

Protein name: Vitamin K epoxide reductase complex subunit 1

Protein accession numbers: IPI00133579

Peptide sequence: (R)ALCDVGTAISCSR(V)

Exclusive (unique to this protein): TRUE

SEQUEST XCorr score: 2.16 SEQUEST DCn score: 0.693

Fix modifications: C3: Carbamidomethyl (+57.02), C11: Carbamidomethyl (+57.02)Variable modifications: None

charge: 2 Observed m/z: 705.3284

Actual minus calculated peptide mass (AMU): -0.0028056

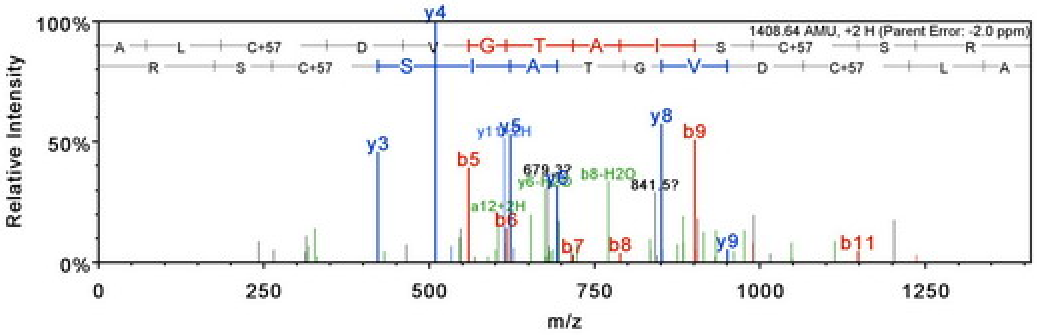

B	B Ions	B+2H	B-NH3	B-H2O	AA	Y Ions	Y+2H	Y-NH3	Y-H2O	Y
1	72.0				A	1409.7	705.3	1392.6	1391.6	13
2	185.1				L	1338.6	669.8	1321.6	1320.6	12
3	345.2				C+57	1225.5	613.3	1208.5	1207.5	11
4	460.2			442.2	D	1065.5	533.3	1048.5	1047.5	10
5	559.3			541.2	V	950.5	475.7	933.5	932.5	9
6	616.3	308.6		598.3	G	851.4	426.2	834.4	833.4	8
7	717.3	359.2		699.3	T	794.4	397.7	777.4	776.4	7
8	788.4	394.7		770.4	A	693.3	347.2	676.3	675.3	6
9	901.5	451.2		883.4	I	622.3		605.3	604.3	5
10	988.5	494.7		970.5	S	509.2		492.2	491.2	4
11	1148.5	574.8		1130.5	C+57	422.2		405.2	404.2	3
12	1235.5	618.3		1217.5	S	262.1		245.1	244.1	2
13	1409.7	705.3	1392.6	1391.6	R	175.1		158.1		1

Gene symbol: Vps26a

Protein name: Isoform 2 of Vacuolar protein sorting-associated protein 26A

Protein accession numbers: IPI00329942

Peptide sequence: (K)EITGIGPSTTTETETIAK(Y)

Exclusive (unique to this protein): TRUE

SEQUEST XCorr score: 2.55 SEQUEST DCn score: 0.736

Fix modifications: None

Variable modifications: None

charge: 2 Observed m/z: 924.9728

Actual minus calculated peptide mass (AMU): -0.006958

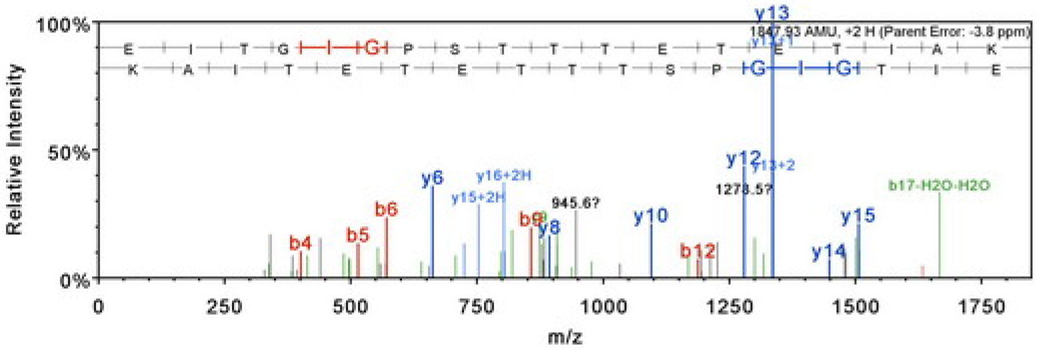

B	B Ions	B+2H	B-NH3	B-H2O	AA	Y Ions	Y+2H	Y-NH3	Y-H2O	Y
1	130.1			112.0	E	1848.9	925.0	1831.9	1830.9	18
2	243.1			225.1	I	1719.9	860.5	1702.9	1701.9	17
3	344.2			326.2	T	1606.8	803.9	1589.8	1588.8	16
4	401.2			383.2	G	1505.8	753.4	1488.7	1487.8	15
5	514.3			496.3	I	1448.8	724.9	1431.7	1430.7	14
6	571.3	286.2		553.3	G	1335.7	668.3	1318.6	1317.7	13
7	668.4	334.7		650.4	P	1278.6	639.8	1261.6	1260.6	12
8	755.4	378.2		737.4	S	1181.6	591.3	1164.6	1163.6	11
9	856.4	428.7		838.4	T	1094.6	547.8	1077.5	1076.5	10
10	957.5	479.3		939.5	T	993.5	497.3	976.5	975.5	9
11	1058.5	529.8		1040.5	T	892.5	446.7	875.4	874.5	8
12	1187.6	594.3		1169.6	E	791.4	396.2	774.4	773.4	7
13	1288.6	644.8		1270.6	T	662.4	331.7	645.4	644.4	6
14	1417.7	709.3		1399.7	E	561.3		544.3	543.3	5
15	1518.7	759.9		1500.7	T	432.3		415.3	414.3	4
16	1631.8	816.4		1613.8	I	331.2		314.2		3
17	1702.8	851.9		1684.8	A	218.2		201.1		2
18	1848.9	925.0	1831.9	1830.9	K	147.1		130.1		1

Gene symbol: Vps29

Protein name: Isoform 1 of Vacuolar protein sorting-associated protein 29

Protein accession numbers: IPI00136936,IPI00230513

Peptide sequence: (R)GDFDENLNYPEQK(V)

Exclusive (unique to this protein): TRUE

SEQUEST XCorr score: 2.07 SEQUEST DCn score: 0.473

Fix modifications: None

Variable modifications: None

charge: 2 Observed m/z: 784.8494

Actual minus calculated peptide mass (AMU): 0.003784

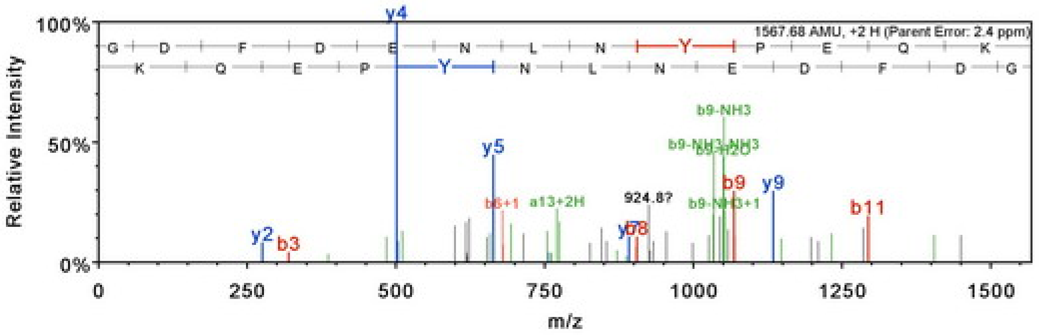

B	B Ions	B+2H	B-NH3	B-H2O	AA	Y Ions	Y+2H	Y-NH3	Y-H2O	Y
1	58.0				G	1568.7	784.9	1551.7	1550.7	13
2	173.1			155.1	D	1511.7	756.3	1494.6	1493.7	12
3	320.1			302.1	F	1396.6	698.8	1379.6	1378.6	11
4	435.2			417.1	D	1249.6	625.3	1232.5	1231.6	10
5	564.2			546.2	E	1134.5	567.8	1117.5	1116.5	9
6	678.2	339.6	661.2	660.2	N	1005.5	503.3	988.5	987.5	8
7	791.3	396.2	774.3	773.3	L	891.5	446.2	874.4	873.5	7
8	905.4	453.2	888.3	887.4	N	778.4	389.7	761.4	760.4	6
9	1068.4	534.7	1051.4	1050.4	Y	664.3		647.3	646.3	5
10	1165.5	583.2	1148.5	1147.5	P	501.3		484.2	483.3	4
11	1294.5	647.8	1277.5	1276.5	E	404.2		387.2	386.2	3
12	1422.6	711.8	1405.5	1404.6	Q	275.2		258.1		2
13	1568.7	784.9	1551.7	1550.7	K	147.1		130.1		1

Gene symbol: Vps4a

Protein name: Vacuolar protein sorting-associating protein 4A

Protein accession numbers: IPI00312172

Peptide sequence: (K)TEGYSGADISIIVR(D)

Exclusive (unique to this protein): TRUE

SEQUEST XCorr score: 2.36 SEQUEST DCn score: 0.188

Fix modifications: None

Variable modifications: None

charge: 2 Observed m/z: 740.8904

Actual minus calculated peptide mass (AMU): 0.0081757

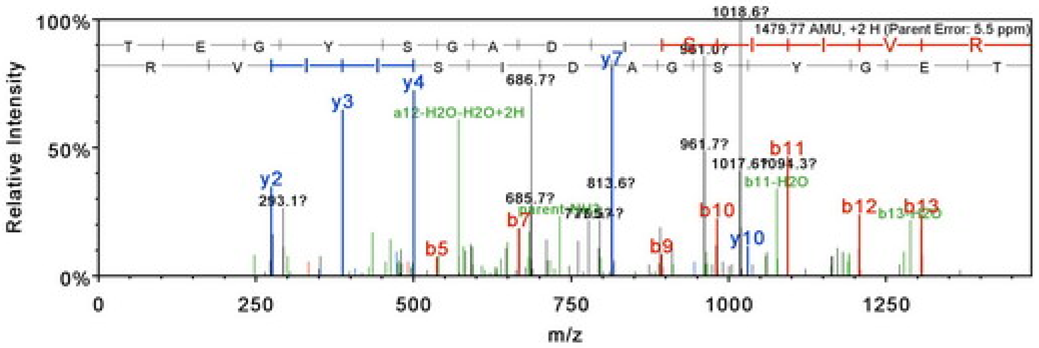

B	B Ions	B+2H	B-NH3	B-H2O	AA	Y Ions	Y+2H	Y-NH3	Y-H2O	Y
1	102.1			84.0	T	1480.8	740.9	1463.7	1462.8	14
2	231.1			213.1	E	1379.7	690.4	1362.7	1361.7	13
3	288.1			270.1	G	1250.7	625.8	1233.7	1232.7	12
4	451.2			433.2	Y	1193.7	597.3	1176.6	1175.6	11
5	538.2			520.2	S	1030.6	515.8	1013.6	1012.6	10
6	595.2	298.1		577.2	G	943.6	472.3	926.5	925.6	9
7	666.3	333.6		648.3	A	886.5	443.8	869.5	868.5	8
8	781.3	391.2		763.3	D	815.5	408.3	798.5	797.5	7
9	894.4	447.7		876.4	I	700.5	350.7	683.5	682.5	6
10	981.4	491.2		963.4	S	587.4		570.4	569.4	5
11	1094.5	547.8		1076.5	I	500.4		483.3		4
12	1207.6	604.3		1189.6	I	387.3		370.3		3
13	1306.7	653.8		1288.6	V	274.2		257.2		2
14	1480.8	740.9	1463.7	1462.8	R	175.1		158.1		1

Gene symbol: Ykt6

Protein name: Synaptobrevin homolog YKT6

Protein accession numbers: IPI00453771

Peptide sequence: (R)SDSLAGVVIADSEYPSR(V)

Exclusive (unique to this protein): TRUE

SEQUEST XCorr score: 2.96 SEQUEST DCn score: 0.538

Fix modifications: None

Variable modifications: None

charge: 2 Observed m/z: 883.4303

Actual minus calculated peptide mass (AMU): -0.008301

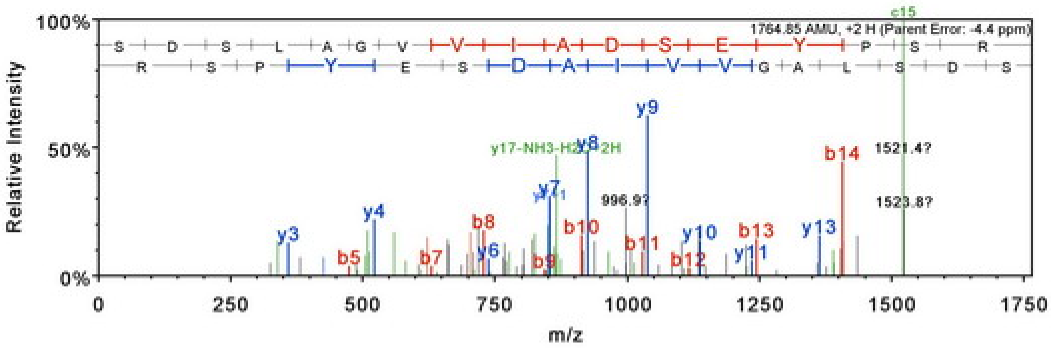

B	B Ions	B+2H	B-NH3	B-H2O	AA	Y Ions	Y+2H	Y-NH3	Y-H2O	Y
1	88.0			70.0	S	1765.9	883.4	1748.8	1747.9	17
2	203.1			185.1	D	1678.8	839.9	1661.8	1660.8	16
3	290.1			272.1	S	1563.8	782.4	1546.8	1545.8	15
4	403.2			385.2	L	1476.8	738.9	1459.7	1458.8	14
5	474.2			456.2	A	1363.7	682.4	1346.7	1345.7	13
6	531.2	266.1		513.2	G	1292.7	646.8	1275.6	1274.6	12
7	630.3	315.7		612.3	V	1235.6	618.3	1218.6	1217.6	11
8	729.4	365.2		711.4	V	1136.6	568.8	1119.5	1118.5	10
9	842.5	421.7		824.5	I	1037.5	519.3	1020.5	1019.5	9
10	913.5	457.3		895.5	A	924.4	462.7	907.4	906.4	8
11	1028.5	514.8		1010.5	D	853.4	427.2	836.3	835.4	7
12	1115.6	558.3		1097.5	S	738.3	369.7	721.3	720.3	6
13	1244.6	622.8		1226.6	E	651.3		634.3	633.3	5
14	1407.7	704.3		1389.7	Y	522.3		505.2	504.3	4
15	1504.7	752.9		1486.7	P	359.2		342.2	341.2	3
16	1591.8	796.4		1573.7	S	262.1		245.1	244.1	2
17	1765.9	883.4	1748.8	1747.9	R	175.1		158.1		1

Gene symbol: Zar1

Protein name: Zygote arrest 1

Protein accession numbers: IPI00280501

Peptide sequence: (R)SEEGDVQAAGQAGWEQQPPPEDR(N)

Exclusive (unique to this protein): TRUE

SEQUEST XCorr score: 2.47 SEQUEST DCn score: 0.742

Fix modifications: None

Variable modifications: None

charge: 2 Observed m/z: 1241.0485

Actual minus calculated peptide mass (AMU): -0.0031752

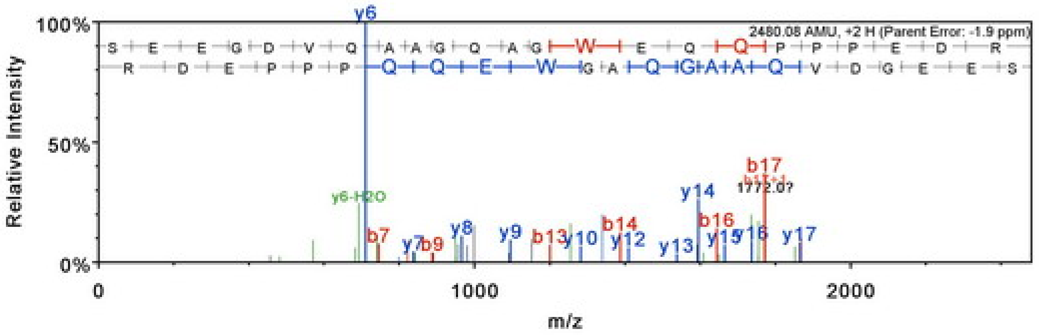

B	B Ions	B+2H	B-NH3	B-H2O	AA	Y Ions	Y+2H	Y-NH3	Y-H2O	Y
1	88.0			70.0	S	2481.1	1241.0	2464.1	2463.1	23
2	217.1			199.1	E	2394.1	1197.5	2377.0	2376.1	22
3	346.1			328.1	E	2265.0	1133.0	2248.0	2247.0	21
4	403.2			385.1	G	2136.0	1068.5	2118.9	2118.0	20
5	518.2			500.2	D	2078.9	1040.0	2061.9	2060.9	19
6	617.2	309.1		599.2	V	1963.9	982.5	1946.9	1945.9	18
7	745.3	373.2	728.3	727.3	Q	1864.9	932.9	1847.8	1846.9	17
8	816.3	408.7	799.3	798.3	A	1736.8	868.9	1719.8	1718.8	16
9	887.4	444.2	870.4	869.4	A	1665.8	833.4	1648.7	1647.8	15
10	944.4	472.7	927.4	926.4	G	1594.7	797.9	1577.7	1576.7	14
11	1072.5	536.7	1055.4	1054.4	Q	1537.7	769.4	1520.7	1519.7	13
12	1143.5	572.3	1126.5	1125.5	A	1409.7	705.3	1392.6	1391.6	12
13	1200.5	600.8	1183.5	1182.5	G	1338.6	669.8	1321.6	1320.6	11
14	1386.6	693.8	1369.6	1368.6	W	1281.6	641.3	1264.6	1263.6	10
15	1515.6	758.3	1498.6	1497.6	E	1095.5	548.3	1078.5	1077.5	9
16	1643.7	822.4	1626.7	1625.7	Q	966.5	483.7	949.4	948.5	8
17	1771.8	886.4	1754.7	1753.7	Q	838.4	419.7	821.4	820.4	7
18	1868.8	934.9	1851.8	1850.8	P	710.4	355.7	693.3	692.3	6
19	1965.9	983.4	1948.8	1947.9	P	613.3		596.3	595.3	5
20	2062.9	1032.0	2045.9	2044.9	P	516.2		499.2	498.2	4
21	2192.0	1096.5	2174.9	2173.9	E	419.2		402.2	401.2	3
22	2307.0	1154.0	2290.0	2289.0	D	290.2		273.1	272.1	2
23	2481.1	1241.0	2464.1	2463.1	R	175.1		158.1		1

Gene symbol: Zp1

Protein name: Zona pellucida sperm-binding protein 1 precursor

Protein accession numbers: IPI00310464

Peptide sequence: (K)TFSSYYQGSDYPLVR(L)

Exclusive (unique to this protein): TRUE

SEQUEST XCorr score: 2.25 SEQUEST DCn score: 0.603

Fix modifications: None

Variable modifications: None

charge: 2 Observed m/z: 891.9179

Actual minus calculated peptide mass (AMU): -0.005859

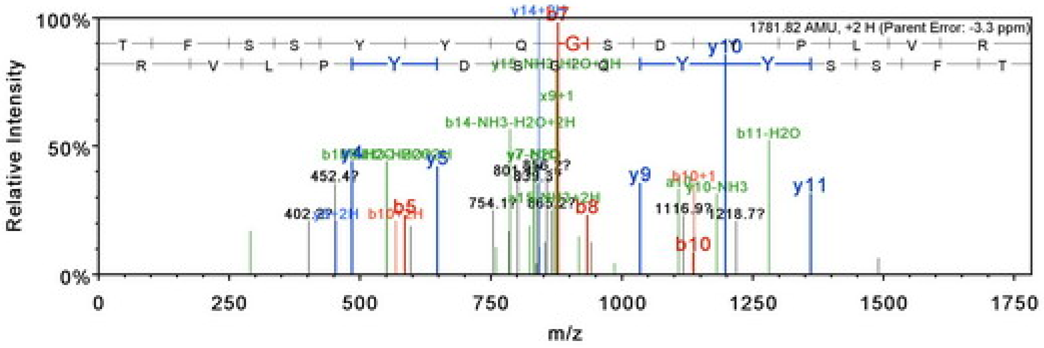

B	B Ions	B+2H	B-NH3	B-H2O	AA	Y Ions	Y+2H	Y-NH3	Y-H2O	Y
1	102.1			84.0	T	1782.8	891.9	1765.8	1764.8	15
2	249.1			231.1	F	1681.8	841.4	1664.8	1663.8	14
3	336.2			318.2	S	1534.7	767.9	1517.7	1516.7	13
4	423.2			405.2	S	1447.7	724.4	1430.7	1429.7	12
5	586.3			568.2	Y	1360.7	680.8	1343.6	1342.6	11
6	749.3	375.2		731.3	Y	1197.6	599.3	1180.6	1179.6	10
7	877.4	439.2	860.4	859.4	Q	1034.5	517.8	1017.5	1016.5	9
8	934.4	467.7	917.4	916.4	G	906.5	453.7	889.4	888.5	8
9	1021.4	511.2	1004.4	1003.4	S	849.5	425.2	832.4	831.4	7
10	1136.5	568.7	1119.4	1118.4	D	762.4	381.7	745.4	744.4	6
11	1299.5	650.3	1282.5	1281.5	Y	647.4		630.4		5
12	1396.6	698.8	1379.5	1378.6	P	484.3		467.3		4
13	1509.7	755.3	1492.6	1491.6	L	387.3		370.3		3
14	1608.7	804.9	1591.7	1590.7	V	274.2		257.2		2
15	1782.8	891.9	1765.8	1764.8	R	175.1		158.1		1

Gene symbol: Zp3

Protein name: Zona pellucida sperm-binding protein 3 precursor

Protein accession numbers: IPI00114128

Peptide sequence: (K)LVQPGDLTLGSEGCQPR(V)

Exclusive (unique to this protein): TRUE

SEQUEST XCorr score: 2.48 SEQUEST DCn score: 0.672

Fix modifications: C14: Carbamidomethyl (+57.02)Variable modifications: None

charge: 2 Observed m/z: 913.9554

Actual minus calculated peptide mass (AMU): -0.004517

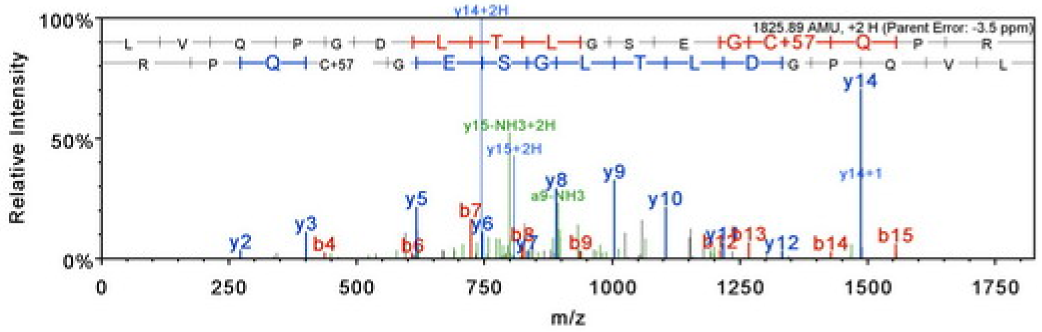

B	B Ions	B+2H	B-NH3	B-H2O	AA	Y Ions	Y+2H	Y-NH3	Y-H2O	Y
1	114.1				L	1826.9	914.0	1809.9	1808.9	17
2	213.2				V	1713.8	857.4	1696.8	1695.8	16
3	341.2		324.2		Q	1614.8	807.9	1597.7	1596.7	15
4	438.3		421.3		P	1486.7	743.9	1469.7	1468.7	14
5	495.3		478.3		G	1389.6	695.3	1372.6	1371.6	13
6	610.3	305.7	593.3	592.3	D	1332.6	666.8	1315.6	1314.6	12
7	723.4	362.2	706.4	705.4	L	1217.6	609.3	1200.6	1199.6	11
8	824.5	412.7	807.4	806.4	T	1104.5	552.8	1087.5	1086.5	10
9	937.5	469.3	920.5	919.5	L	1003.5	502.2	986.4	985.5	9
10	994.6	497.8	977.5	976.6	G	890.4	445.7	873.4	872.4	8
11	1081.6	541.3	1064.6	1063.6	S	833.4	417.2	816.3	815.4	7
12	1210.6	605.8	1193.6	1192.6	E	746.3	373.7	729.3	728.3	6
13	1267.7	634.3	1250.6	1249.6	G	617.3		600.3		5
14	1427.7	714.4	1410.7	1409.7	C+57	560.3		543.2		4
15	1555.7	778.4	1538.7	1537.7	Q	400.2		383.2		3
16	1652.8	826.9	1635.8	1634.8	P	272.2		255.2		2
17	1826.9	914.0	1809.9	1808.9	R	175.1		158.1		1
